# Intermolecular Aza-Wacker Coupling of Alkenes with Azoles by Photo-Aerobic Selenium-π-Acid Multicatalysis

**DOI:** 10.1021/acscatal.4c01327

**Published:** 2024-06-11

**Authors:** Tao Lei, Theresa Appleson, Alexander Breder

**Affiliations:** Institut für Organische Chemie, Universität Regensburg, Universitätstrasse 31, 93053 Regensburg, Germany

**Keywords:** aza-Wacker coupling, azoles, alkenes, selenium-π-acid catalysis, photoredox catalysis

## Abstract

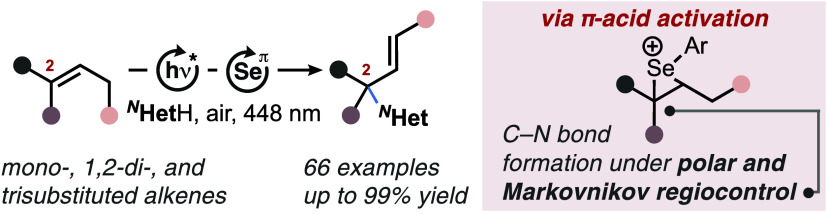

Herein, the intermolecular, photoaerobic aza-Wacker coupling of azoles with alkenes by means of dual and ternary selenium-π-acid multicatalysis is presented. The title method permits an expedited avenue toward a broad scope of *N*-allylated azoles and representative azinones under mild conditions with broad functional group tolerance, as is showcased in more than 60 examples including late-stage drug derivatizations. From a regiochemical perspective, the protocol is complementary to cognate photoredox catalytic olefin aminations, as they typically proceed through either allylic hydrogen atom abstraction or single electron oxidation of the alkene substrate. These methods predominantly result in C–N bond formations at the allylic periphery of the alkene or the less substituted position of the former π-bond (i.e., *anti*-Markovnikov selectivity). The current process, however, operates through a radical-polar crossover mechanism, which solely affects the selenium catalyst, thus allowing the alkene to be converted strictly through an ionic two-electron transfer regime under Markovnikov control. In addition, it is shown that the corresponding *N*-vinyl azoles can also be accessed by sequential or one-pot treatment of the allylic azoles with base, thus emphasizing the exquisite utility of this method.

## Introduction

**Scheme 1:**
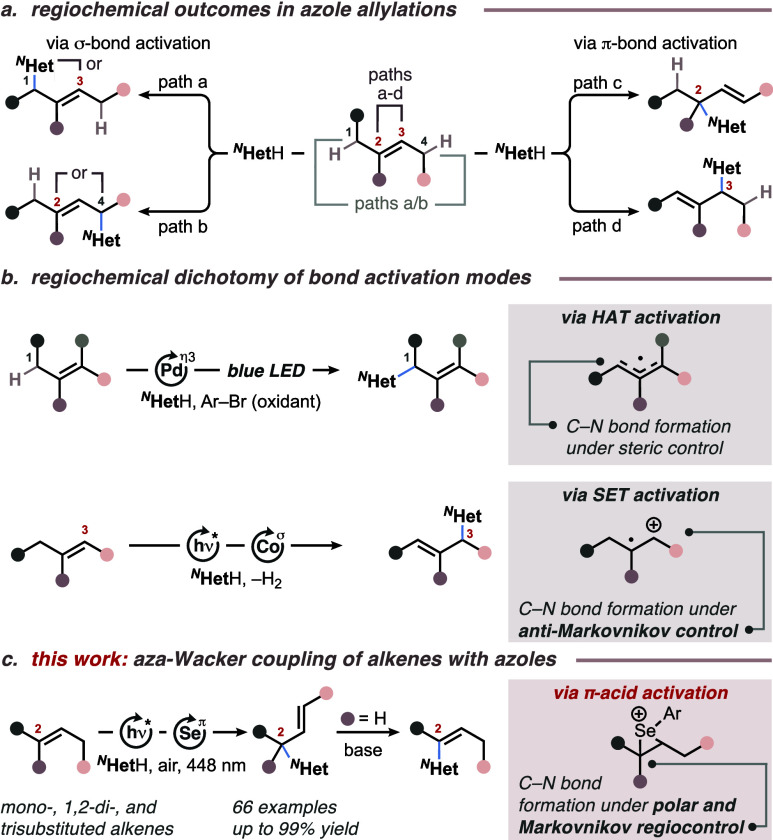
Activation Methods and Examples of Oxidative Azole Allylations. (a) Overview on Possible Regiochemical Outcomes in the *N*-Allylation of Azoles through C–H σ- and C–C π-Bond Activations. (b) Current Exemplary Strategies for the Regioselective *N*-Allylation of Azoles by Gevorgyan et al.^5d^ (Top) and Lei, Cai et al.^5e^ (Bottom). (c) This Work: Markovnikov-Selective, Photoaerobic Selenium-π-Acid Multicatalytic Aza-Wacker Coupling of Alkenes with Azoles HAT = hydrogen atom transfer, SET = single electron transfer, *^N^*HetH = *N*-heterocycle.

Azoles represent a structural motif frequently found in biologically and pharmaceutically active compounds.^1^ Due to the industrial significance of this heterocycle class, substantial efforts were invested into method development and strategical concepts for their bespoke and diversified syntheses.^2^ In this context,^3^ electrophilic allylations of azoles were shown to offer an expedited avenue toward valorized building blocks and synthetic intermediates for established pharmaceuticals, drug leads, and agrochemicals.^4^ The direct redox-coupling of 1,2-di- or higher substituted alkenes to azoles without any prefunctionalization^5^ of either reactant provides a streamlined, highly redox economic^6^ avenue to customized target structures. However, a severe challenge associated with such enterprises arises from regiochemical considerations. More concretely, nonpolar, internal alkenes offer up to 4 positions at which the C–N σ-bond coupling can take place, depending on the nature of the activation principle and substitution pattern around the alkene (Scheme 1a, paths a,b: C–N bond in position 1 or 4 with the double bond fixed within positions 2 and 3; paths c,d: C–N bond in position 2 or 3 with concomitant transposition of the double bond terminating at position 4 or 1, respectively).

C–H σ-bond activations via hydrogen atom transfer (HAT) can result in the functionalization of each position from 1 to 4 (Scheme 1a, path a or b), while direct electrophilic π-bond activations are restricted to C–N couplings in position 2 or 3 (Scheme 1a, path c or d). So far, only a small number of suitable synthetic methods have been reported that allow for the regioselective allylation of azoles. Sporadic early examples of such C–N couplings predicated on the use of, in part, corrosive oxidants, such as *N*-halosuccinimides, peroxides, and benzoquinones, were restricted to alkene substrates with specific substitution patterns (e.g., terminal or π-conjugated).^5a−5c^ In 2022, Gevorgyan et al. reported a Pd-photoredox hybrid-catalytic C–N coupling to construct a broad series of allylated amines, including two azoles, from simple alkenes via allylic HAT. The protocol relied on the use of customized aryl bromides as terminal oxidants to generate aryl radicals as key HAT acceptors (Scheme 1b, top). The regiochemical preference for C1 functionalization (Scheme 1a, path a) was governed by thermodynamic and steric substrate control.^5d^ A complementary approach was recently reported by Lei, Cai, and co-workers, who showed that *N*-allylation of azoles can be accomplished through dual photoredox/cobalt catalysis (Scheme 1b, bottom).^5e^ The protocol is mechanistically reminiscent of earlier work by Yoon et al. on Cu-mediated, photoredox catalytic allylations of N- and O-nucleophiles^7^ and accordingly shows strong *anti*-Markovnikov regioselectivity in preference of position 3 (Scheme 1a, path d). Despite the promising achievements made so far in the context of azole allylations, protocols that are generally suitable for 1,2-disubstituted alkenes and that display high electronic or, in the case of 1,1,2-trisubstituted alkenes, Markovnikov regioselectivity remain yet to be established.

A promising alternative to existing methods on azole allylations may lie in the implementation of selenium-π-acid catalysis.^8^ In the past, this technique has shown unique abilities to control regioselectivity via dynamic covalent π-bond activation, which has been successfully applied in the vinylic-,^9^ allylic-,^10,11^ and 1,2-difunctionalization^12^ of olefins with a vast panoply of nucleophiles. A seminal example of selenium-π-acid-catalyzed intermolecular C–N couplings between simple alkenes and *N*-fluorobenzenesulfonimide (NFSI) was already reported in 2013.^13^ In these reactions, the actual sulfonimide nucleophile was released in situ during the catalytic cycle. Subsequently, various conceptually related allylic and vinylic aminations were reported, including an interesting electrophilic *N*-vinylation of pyridines.^9d^ Most of these procedures predicate on the use of halogen oxidants and tolerate only oxidation-insensitive N-nucleophiles. A notable further development in this field was demonstrated by Michael et al., who elaborated a selenium-catalyzed ene reaction using hypervalent iodine reagents as terminal oxidants.^11c−11j,14^ The protocol displayed high substrate-based regiocontrol and was found suitable for mono- to tetrasubstituted alkenes but was specific to primary sulfonamide and carbamate coupling partners for the alkenes.

The abovementioned state of affairs in selenium-π-acid catalysis already indicates that its synthetic potential is far from being exhaustively exploited. For instance, our group has previously shown that the scope of suitable nucleophiles can be markedly enhanced when resorting to ambient air as a mild and compatible terminal oxidant.^15^ The implementation of air as a reactant was accomplished by the merger of photoredox and selenium-π-acid catalysis, enabling the coupling of various nucleophiles such as carboxylic acids,^15a,15b^ hydrogen phosphates,^15c^ alcohols,^15d^ and sulfonamides^15f,15g^ to simple alkenes through a dual catalytic radical-polar crossover mechanism.^16^ Against this background, we posited that this technique might also prove suitable for azole nucleophiles, provided that the envisioned heteroarenes would exhibit sufficient compatibility with our photoaerobic reaction conditions. As a result of these considerations, we present herein the first photoaerobic selenium-π-acid-catalyzed aza-Wacker coupling of alkenes with N-heterocycles, exemplified with a broad series of azoles and selected azinones (Scheme 1c). The title protocol shows strong, alkene-based electronic regiocontrol (including Markovnikov selectivity for mono- and trisubstituted alkenes) and high functional group tolerance, even in complex molecular architectures. In addition to allylations, the corresponding vinylation products can also be accessed in high yields through eventual exposure of the allylated azole intermediates to a base, which is a testament to the marked flexibility of our method.

## Results and Discussion

At the outset, 3 equiv of (*E*)-hex-3-enoic acid ethyl ester (**1a**) were exposed to 4-chloropyrazole (**2a**) in the presence of 5 mol % 2,4,6-tris(4-methoxyphenyl)pyrylium tetrafluoroborate (TAPT) and 10 mol % (PhSe)_2_ under air and 448 nm irradiation in dichloroethane (DCE), yielding product **3a** in 65% along with 11% vinylic isomer **3a’** (Table 1, entry 1, **3a**:**3a**’ = 6:1). Under these conditions, no electrophilic selenation of the azole core was observed.^17^ While chloroform also provided a total yield of 76% for isomers **3a** and **3a’** with a slightly lower isomeric ratio (entry 2, **3a**:**3a**’ = 5:1), other solvents such as acetonitrile, acetone, and toluene gave inferior results (entries 3–5). From previous studies on asymmetric aza-Wacker cyclizations we learned that disulfide cocatalysts can accelerate the overall reaction rate and suppress side reactivity.^15f^ Mechanistic investigations indicated that disulfides serve two purposes: (1) they function as electron–hole shuttles between the excited photoredox catalyst and the selenium cocatalyst, and (2) they accelerate the final step in the catalytic cycle, namely the β-elimination of the product from the selenium catalyst.^15g^ Accordingly, we added 5 mol % (4-ClPhS)_2_ to the reaction mixture and ran the reaction for 21 h on an 1.0 mmol scale (for scale-up results, see the Supporting Information), which resulted in a comparable total yield of 78% but with a markedly improved isomeric ratio of 13:1 (entry 6). A similar isomeric ratio improvement was observed when alkene **2a** was used as the limiting reagent in the presence of 3 equiv of pyrazole **1a** (entry 7, **3a**:**3a**’ = 14:1). The addition of the disulfide cocatalyst to the latter conditions further improved the isomeric ratio of the product to 25:1, albeit with a slight decrease in total yield (entry 8). Control experiments showed that TAPT, (PhSe)_2_, light, and air are crucial components in the reaction (entries 9–12).

**Table 1:**
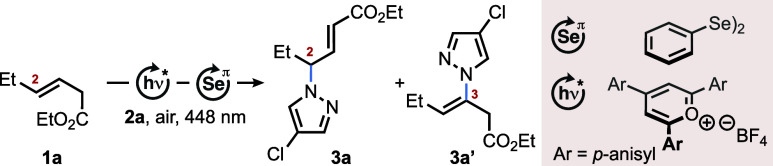
Optimization of Photoaerobic Aza-Wacker Coupling of Alkenes with Azoles^a^

entry	solvent	time	yield **3a**^b^	total yield	ratio **3a:3a’**
1	DCE	8 h	65%	76%	6:1
2	CHCl_3_	8 h	63%	76%	5:1
3	MeCN	8 h	20%	24%	5:1
4	Acetone	8 h	18%	21%	6:1
5	Toluene	8 h	4%	5%	4:1
6^c^^,^^d^	DCE	21 h	72%	78%	13:1
7^e^	DCE	21 h	68% (68%)	73%	14:1
8^c^^,^^e^	DCE	21 h	63%	66%	25:1
9^f^	DCE	8 h	0%	0%	
10^g^	DCE	8 h	0%	0%	
11^h^	DCE	8 h	0%	2%	0:1
12^i^	DCE	8 h	0%	0%	

a Conditions: 4-Chloropyrazole (**2a**, 0.30 mmol, 1.0 equiv), (*E*)-hex-3-enoate **1a** (3.0 equiv), TAPT (5 mol %), (PhSe)_2_ (10 mol %), solvent (4 mL), 448 nm irradiation, air, and 19 °C.

b Yield determined by ^1^H NMR spectroscopy using 1,4-dimethoxybenzene as an internal standard. Isolated yield in parentheses.

c (4*-*ClPhS)_2_ (5 mol %) added.

d Pyrazole **2a** (1.0 mmol, 1.0 equiv), alkene **1a** (3.0 equiv), and DCE (5 mL).

e Alkene **1a** (1.0 mmol, 1.0 equiv), pyrazole **2a** (3.0 equiv), and DCE (5 mL). Control experiments were performed under unaltered conditions except for the parameters indicated as follows:

f without irradiation.

g without (PhSe)_2_,

h without TAPT,

i N_2_ instead of air. For further details, see the Supporting Information.

**Scheme 2:**
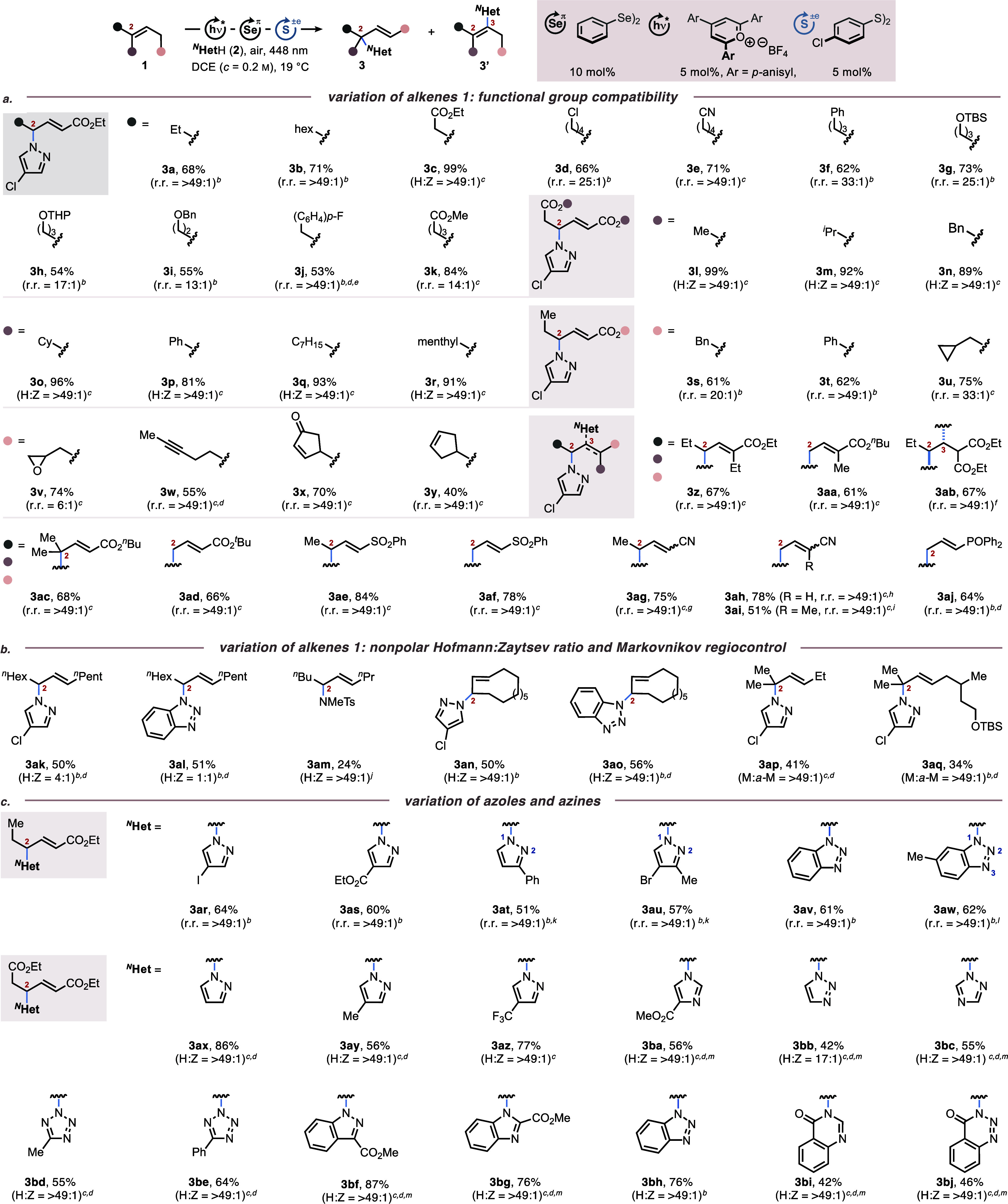
Substrate Scope of the Intermolecular Aza-Wacker Coupling of Alkenes with Azoles (a) Evaluation of Functional Group Tolerance, (b) Hofmann/Zaytsev and Markovnikov/*anti*-Markovnikov Regioselectivity, (c) N-Nucleophile Compatibility All reactions were carried out on a 1.0 mmol (1.0 equiv) scale with regards to the limiting compound, using 5 mol % TAPT and 10 mol % (PhSe)_2_, if not indicated otherwise. Yields and regioisomeric ratios (r.r. = isomer **3**: isomer **3**′) refer to isolated compounds. If isomer **3**′ was indetectable in the ^1^H NMR spectrum, r.r. is given as >49:1. For details, see the Supporting Information. Indicated concentration refers to a limiting compound. ^*b*^Alkene **1** (1.0 equiv), ^*N*^HetH **2** (3.0 equiv). ^*c*^Alkene **1** (3.0 equiv), ^*N*^HetH **2** (1.0 equiv). ^*d*^(4-ClPhS)_2_ (5 mol %) added. ^*e*^*E*/*Z* = 33:1. ^*f*^Alkene **1** (1.00 equiv), ^*N*^HetH **2** (6.50 equiv). ^*g*^*E*/*Z* = 6:1. ^*h*^*E*/*Z* = 1:1. ^*i*^*Z*-isomer. ^*j*^Alkene **1** (0.50 mmol), ^*N*^HetH **2** (5.00 equiv), TAPT (10 mol %), (PhSe)_2_ (20 mol %) in PhCl (5 mL). ^*k*^*N*^1^:*N*^2^ = 5:1. ^*l*^*N*^1^:*N*^3^ = 1:1. ^*m*^DCE:HFIP = 3:2 used as the solvent. H:Z = Hofmann:Zaitsev elimination ratio. M:*a*-M = Markovnikov/*anti*-Markovnikov ratio.

With an empirically optimized set of reaction conditions in hand, the substrate scope was examined next by initially varying the alkene coupling partner (Scheme 2). For this purpose, several, in part, acid-sensitive groups were incorporated into the backbone of target **3**. Installation of various polar groups next to position 2 (**3c**-**3k**) had no apparent negative impact on the yield, which ranged between 53 and 99%. Also, regioisomer **3** was consistently preferred over **3**′ in all of these reactions, with a minimum ratio of 7:1 prior to purification and 13:1 after it. These results already show that common functional groups such as halide, cyanide, arene, ether, acetal, and ester moieties are well tolerated. This positive trend continued to hold true for the diester series **3l** to **3r**, for which an average yield of 92% was obtained without any indication for the formation of isomer **3'**. To analyze the tolerance of the title protocol toward the presence of various π-bonds and strained rings, monoesters **1s** to **1y** (see the Supporting Information for details) were converted to their respective aza-Wacker coupling products **3s**-**y**. Particularly noteworthy from this series are the results of compounds **3w** to **3y**, as both the alkyne and the secondary *Z*-alkene group remained largely intact during the catalytic conversion of the coexisting *E*-alkene. We interpret this outcome, on the one hand, as a result of −I effects (**3x** and **3y**) exerted by the proximal oxygen atoms and, on the other hand, as a consequence of the high s-character of the C-atoms in the alkyne, which presumably destabilizes the corresponding selenirenium ion relative to the competing seleniranium ion from the alkene. This kind of chemoselectivity (i.e., alkene vs alkyne) has been observed previously^18^ in related selenium-π-acid-catalyzed reactions and bears testament to the exquisite utility of this methodology in polyfunctional settings.

Switching to other electron-withdrawing groups (e.g., malonyl, sulfonyl, nitrile, and phosphonate) or different degrees of olefin substitution within the substrates or products provided access to compounds **3z** to **3aj** in yields between 51 and 84% (avg. = 69%; median = 67%). These results were next contrasted with those obtained from alkenes being void of any electron-withdrawing groups (Scheme 2b). As anticipated, the yields of azole coupling products **3ak**-**aq** are on average 23 percentage points lower (34–56%) compared to the **3z**-**aj** series, even with the addition of cocatalyst (4-ClPhS)_2_. This observation correlates well with several mechanistic studies from our laboratories, which suggest that either the final step in the multicatalytic cycle, i.e., the β-elimination of the product from the selenium catalyst,^15,19^ or the preceding photooxidation of the selenium residue^15b^ can be rate limiting. The missing electronic influence of any electron-withdrawing groups (EWG) in the case of nonpolar alkenes **2ak**-**2ao** is suspected to slow down β-elimination considerably, opening pathways for probably yield-diminishing side reactions such as the Schenck-ene oxidation.^15,20^ Notably, the current method shows significant preference for the formation of Markovnikov products,^21^ which complements existing protocols on photoredox catalytic amination reactions of nonpolar alkenes proceeding through radical ionic π-bond activations, as they typically result in preferential formation of *anti*-Markovnikov regioisomers (Scheme 1a, path d).^5e,7^

**Scheme 3:**
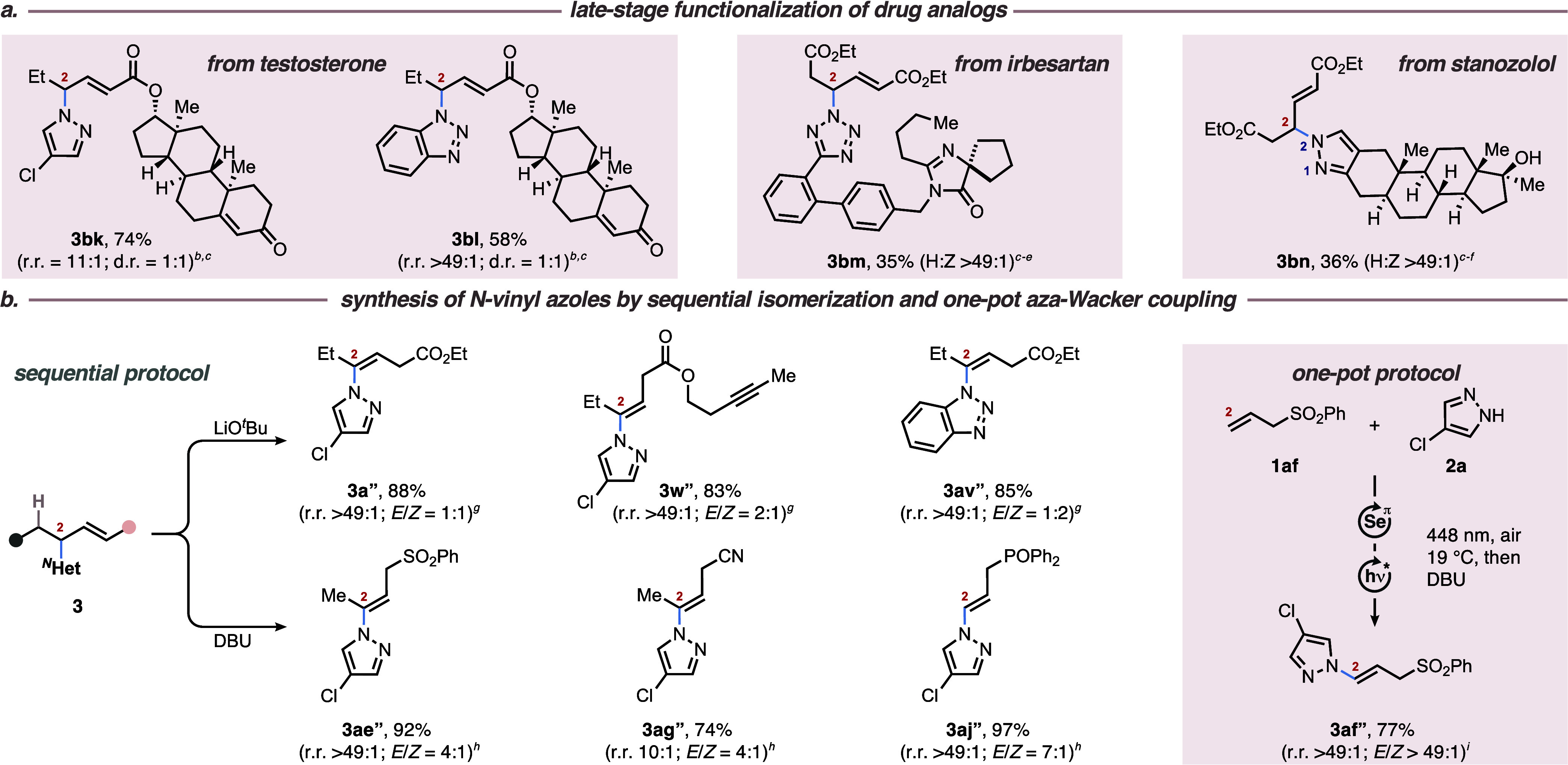
Application of the Intermolecular Aza-Wacker Coupling of Alkenes with Azoles by Photoaerobic Selenium-π-Acid Multicatalysis (a) Late-Stage Functionalization of Biologically Active Compounds and Drug Analogs (b) Sequential and One-Pot Synthesis of *N*-Vinylated Azoles (a) Reactions were carried out on a 1.00 mmol (1.00 equiv) scale with regards to the limiting compound, using 5 mol % TAPT and 10 mol % (PhSe)_2_ in DCE (0.2 m) under irradiation at 448 nm at 19 °C. Yields and regioisomeric ratios (r.r. = isomer **3**: isomer **3**′) refer to isolated compounds. If isomer **3**′ was indetectable in the ^1^H NMR spectrum, r.r. is given as >49:1. For details, see the Supporting Information. (b) Yields and regioisomeric ratios (r.r. = isomer **3”**: isomer **3**) refer to isolated compounds. If isomer **3** was indetectable in the ^1^H NMR spectrum, r.r. is given as >49:1. ^*b*^Alkene **1** (1.00 equiv), ^*N*^HetH **2** (3.00 equiv). ^*c*^(4-ClPhS)_2_ (5 mol %) added. ^*d*^Alkene **1** (3.00 equiv), ^*N*^HetH **2** (1.00 equiv). ^*e*^DCE/HFIP = 3:2 used as a solvent. ^*f*^*N*^2^:*N*^1^ = 5:1. ^*g*^**3** (1.00 equiv), THF (0.05 m), LiO^*t*^Bu (5.00 equiv), 0 °C, N_2_. ^*h*^**3** (1.00 equiv), DCE (0.05 m), DBU (2.00 equiv), r.t., N_2_. ^*i*^Coupling was conducted under conditions b, followed by solvent exchange in the same vessel to continue the reaction under conditions *h*. H:Z = Hofmann:Zaitsev elimination ratio.

At this stage, we tested an extended panoply of azoles and a few azinones as coupling partners to alkenes **1a** and diethyl (*E*)-hex-3-enedioate **1c** (Scheme 2c). Overall, our method provided for all tested heterocycles relatively consistent results with yields averaging around 59% (median = 61%) for the monoester series **3ar**-**aw** (derived from **1a**) and 63% (median = 56%) for the diester series **3ax**-**bj** (derived from **1c**). As in all cases before, functional groups such as halides, esters, and amides proved to be compatible with the reaction conditions. In addition, no sign of direct heteroarene selenation was observed.^17^ Given the robust functional group tolerance of the title procedure, we applied it to late-stage derivatizations of biologically active compounds and drug analogs (Scheme 3a). More concretely, testosteryl ester **1ap** was subjected to the title conditions and gave access to its pyrazolated and benzotriazolated (via *N*^1^) products **3bk** and **3bl** in 74% (r.r. = 11:1) and 58% yield (r.r. >49:1), respectively. Commercially available irbesartan (**2u**) and stanozolol (**2v**), each decorated with tetrazole and pyrazole moieties, respectively, were readily converted under optimized conditions to give derivatives **3bm** and **3bn** in 35 and 36% yields, respectively. As indicated earlier (Table 1), an attack of the azole in position 3 exclusively resulted in the formation of vinylic products **3**′, presumably because of the –I effect of the azole group (i.e., increased acidity of the C–H bond in position 3). We posited that exposure of coupling products **3** to basic conditions would result in complete isomerization toward vinylic isomers **3**″ (Scheme 3b). This turned out to be true since the exposure of coupling products **3a**, **3w**, and **3av** to LiO^*t*^Bu resulted in smooth isomerization into isomers **3a’’**, **3w’’**, and **3av’’** in yields beyond 80%, albeit with moderate to no *E*/*Z*-selectivity. Similar results were obtained when compounds **3ae**, **3ag**, and **3aj** were treated with DBU as a milder base (average yield = 88%, median 92%). Eventually, the latter conditions were combined with the aza-Wacker coupling in a two-step, one-pot sequence to afford vinylated pyrazole **3af’’** in 77% yield (Scheme 3b). The synthetic versatility of the aza-Wacker products was further demonstrated in exemplary Dieckmann cyclizations of **3c** and **3bg** using LiO^*t*^Bu as a base, which furnished corresponding cyclopent-2-enones **4a** and **4b** in 72 and 56% yields, respectively (see the Supporting Information for details).

**Scheme 4:**
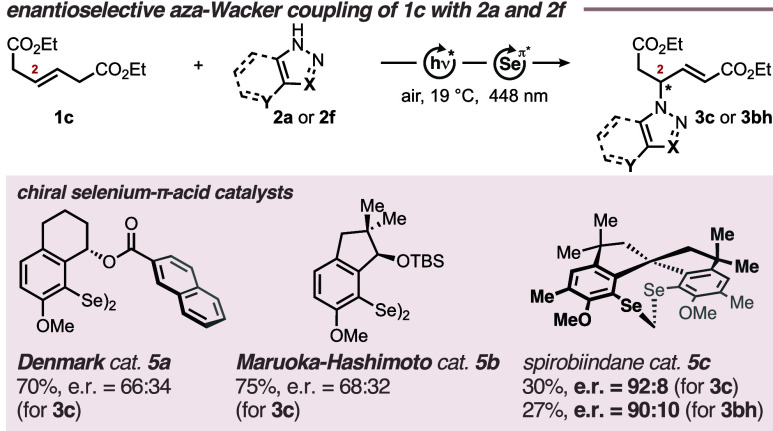
Asymmetric Intermolecular Aza-Wacker Coupling of Alkenes with Azoles by Photoaerobic Selenium-π-Acid Multicatalysis Yields refer to isolated compounds. **2** (0.30 mmol, 1.0 equiv), **1c** (3.0 equiv), TAPT (5 mol %), diselanes **5a–c** (10 mol %), DCE (4 mL), 8 h. e.r. = enantiomeric ratio. **2a**: X = C, Y = Cl. **2f**: X = N, Y = C.

Exemplarily, we also investigated the possibility of running the allylic aza-Wacker coupling enantioselectively using a set of chiral selenium-π-acid catalysts that were previously shown to provide high levels of stereoinduction in related transformations (Scheme 4; for details, see the Supporting Information).^15f,18,22^ For this purpose, alkene **1c** was used as the model substrate, which was exposed to chiral catalysts **5a**–**c** under the standard conditions determined in Table 1. Catalysts **5a**(22) and **5b**(18) furnished product **3c** in yields comparable to that of (PhSe)_2_, albeit with low levels of stereoinduction (e.r. = 66:34 and 68:32, respectively). On the contrary, catalyst **5c**(15f) provided compound **3c** in an e.r. of 92:8 on a 0.30 mmol scale, which remained virtually invariant when scaling up to 1.00 mmol. However, in both cases, the isolated yields did not exceed 30%. A similar outcome was observed with benzotriazole **2f** as the coupling partner, furnishing **3bh** in 27% yield and an e.r. of 90:10 on a 0.30 mmol scale. The sum of these results clearly shows that the allylic aza-Wacker coupling has indeed the potential to be further developed into an efficient enantioselective process. To reach this promising goal, further reaction optimization remains indispensable and will continue in our laboratories.

## Conclusions

In summary, we have presented a novel and operationally simple method for the aza-Wacker coupling of azoles and azinones with alkenes. The reaction proceeds through a radical-polar crossover mechanism,^16^ in which a diselane catalyst is sequentially oxidized to an oligomeric selenonium ion by a photoredox catalyst.^15b,15g,19^ The cationic selenium species functions as a potent π-acid, activating alkenes via seleniranium ion formation. Interception of these ions by azole and azinone nucleophiles, followed by eventual β-elimination, was shown to result in a broad product scope with excellent functional group tolerance. From a regiochemical perspective, our findings complement previous reports on cognate photoredox catalytic aminations of alkenes, as they typically result in the amination of the less substituted position 3 with *anti*-Markovnikov selectivity (Scheme 1, path d). Our conditions, however, show a preference for position 2 with Markovnikov selectivity in the case of trisubstituted alkenes. Therefore, we are optimiztic that our protocol will find ample application in the realm of organic synthesis and related disciplines as a complementary means to decorate N-heterocycles with carbon residues. Further efforts toward the design of enantioselective versions of our intermolecular aza-Wacker protocol are currently underway in our laboratories.

## Supporting Information Available

Materials and methods; conditions optimization and general experimental procedures; control experiments; characterization data of all products; and copies of ^1^H, ^13^C, ^19^F, ^31^P, and ^77^Se spectra of all products (PDF)

## References

[ref1] aLegraverend M.; Grierson D. S. The purines: Potent and versatile small molecule inhibitors and modulators of key biological targets. Bioorg. Med. Chem. 2006, 14, 3987–4006. 10.1016/j.bmc.2005.12.060. 16503144

[ref2] aBuzykin B. I. Formazans in the synthesis of heterocycles I. Synthesis of azoles (Review). Chem. Heterocycl. Compd. 2010, 46, 379–408. 10.1007/s10593-010-0523-0.

[ref3] aBanerjee D.; Jagadeesh R. V.; Junge K.; Junge H.; Beller M. Efficient and Convenient Palladium-Catalyzed Amination of Allylic Alcohols with N-Heterocycles. Angew. Chem., Int. Ed. 2012, 51, 11556–11560. 10.1002/anie.201206319. 23023833

[ref4] aSteffan R. J.; Matelan E.; Ashwell M. A.; Moore W. J.; Solvibile W. R.; Trybulski E.; Chadwick C. C.; Chippari S.; Kenney T.; Eckert A.; et al. Synthesis and activity of substituted 4-(indazol-3-yl)phenols as pathway-selective estrogen receptor ligands useful in the treatment of rheumatoid arthritis. J. Med. Chem. 2004, 47, 6435–6438. 10.1021/jm049194+. 15588074

[ref5] aWei Y.; Liang F.; Zhang X. N-bromoimide/DBU combination as a new strategy for intermolecular allylic amination. Org. Lett. 2013, 15, 5186–5189. 10.1021/ol402287n. 24102289

[ref6] Burns N. Z.; Baran P. S.; Hoffmann R. W. Redox Economy in Organic Synthesis. Angew. Chem., Int. Ed. 2009, 48, 2854–2867. 10.1002/anie.200806086. 19294720

[ref7] Reed N. L.; Lutovsky G. A.; Yoon T. P. Copper-Mediated Radical–Polar Crossover Enables Photocatalytic Oxidative Functionalization of Sterically Bulky Alkenes. J. Am. Chem. Soc. 2021, 143, 6065–6070. 10.1021/jacs.1c02747. 33856228 PMC8547160

[ref8] aOrtgies S.; Breder A. Oxidative Alkene Functionalizations via Selenium-π-Acid Catalysis. ACS Catal. 2017, 7, 5828–5840. 10.1021/acscatal.7b01216.

[ref9] aOrtgies S.; Breder A. Selenium-Catalyzed Oxidative C(sp(2))-H Amination of Alkenes Exemplified in the Expedient Synthesis of (Aza-)Indoles. Org. Lett. 2015, 17, 2748–2751. 10.1021/acs.orglett.5b01156. 25997578

[ref10] aChabaud B.; Sharpless B. Selenium-catalyzed nonradical chlorination of olefins with N-chlorosuccinimide. J. Org. Chem. 1979, 44, 4204–4208. 10.1021/jo01337a046.

[ref11] aGuo R.; Huang J.; Huang H.; Zhao X. Organoselenium-Catalyzed Synthesis of Oxygen- and Nitrogen-Containing Heterocycles. Org. Lett. 2016, 18, 504–507. 10.1021/acs.orglett.5b03543. 26794425

[ref12] aCresswell A. J.; Eey S. T.-C.; Denmark S. E. Catalytic, stereospecific syn-dichlorination of alkenes. Nat. Chem. 2015, 7, 146–152. 10.1038/nchem.2141. PMC4674811 25615668

[ref13] Trenner J.; Depken C.; Weber T.; Breder A. Direct oxidative allylic and vinylic amination of alkenes through selenium catalysis. Angew. Chem., Int. Ed. 2013, 52, 8952–8956. 10.1002/anie.201303662. 23843245

[ref14] Sharpless K. B.; Hori T.; Truesdale L. K.; Dietrich C. O. Allylic amination of olefins and acetylenes by imido selenium compounds. J. Am. Chem. Soc. 1976, 98, 269–271. 10.1021/ja00417a062.

[ref15] aOrtgies S.; Depken C.; Breder A. Oxidative Allylic Esterification of Alkenes by Cooperative Selenium-Catalysis Using Air as the Sole Oxidant. Org. Lett. 2016, 18, 2856–2859. 10.1021/acs.orglett.6b01130. 27257803

[ref16] Fletcher R. J.; Lampard C.; Murphy J. A.; Lewis N. Tetrathiafulvalene: a catalyst for sequential radical–polar reactions. J. Chem. Soc., Perkin Trans. 1995, 1, 623–633. 10.1039/P19950000623.

[ref17] aSaba S.; Rafique J.; Franco M. S.; Schneider A. R.; Espíndola L.; Silva D. O.; Braga A. L. Rose Bengal catalysed photo-induced selenylation of indoles, imidazoles and arenes: a metal free approach. Org. Biomol. Chem. 2018, 16, 880–885. 10.1039/C7OB03177G. 29340417

[ref18] Kawamata Y.; Hashimoto T.; Maruoka K. A Chiral Electrophilic Selenium Catalyst for Highly Enantioselective Oxidative Cyclization. J. Am. Chem. Soc. 2016, 138, 5206–5209. 10.1021/jacs.6b01462. 27064419

[ref19] Wilken M.; Ortgies S.; Breder A.; Siewert I. Mechanistic Studies on the Anodic Functionalization of Alkenes Catalyzed by Diselenides. ACS Catal. 2018, 8, 10901–10912. 10.1021/acscatal.8b01236.

[ref20] Schenck G. O. Zur Theorie der photosensibilisierten Reaktion mit molekularem Sauerstoff. Naturwissenschaften 1948, 35, 28–29. 10.1007/BF00626628.

[ref21] Makovnikov selectivity in this context refers to the formation of the C-N bond at the carbon atom that electronically stabilizes the positive charge best in the preceding seleniranium intermediate. For monosubstituted alkenes with electron withdrawing groups (EWG) in the allylic position (i.e., 1aa, 1ad, 1af, 1ah-aj) this means that the C–N bond formation occurs at the terminal C-atom, which is less affected by the −I-effect of the EWG. This definition is in accordance with the IUPAC definition of the Markovnikov selectivity, and has also been used in related contexts (e.g., *J. Org. Chem.***2006**, *71*, 7293–7306.).

[ref22] Tao Z.; Gilbert B. B.; Denmark S. E. Catalytic, Enantioselective syn-Diamination of Alkenes. J. Am. Chem. Soc. 2019, 141, 19161–19170. 10.1021/jacs.9b11261. 31742399 PMC6939451

