# Supplementary material for: Intermolecular Aza-Wacker Coupling of Alkenes with Azoles by Photo-Aerobic Selenium-π-Acid Multicatalysis
Source: ACS Catal. 2024 Jun 11;14(12):9586–93. doi: 10.1021/acscatal.4c01327 (PMC11197018; doi:10.1021/acscatal.4c01327)
Supplement: Supplementary file 1 — cs4c01327_si_001.pdf [file cs4c01327_si_001.pdf]

Supporting Information for

Intermolecular Aza-Wacker Coupling of Alkenes with Azoles by Photo-Aerobic Selenium-π-Acid Multicatalysis

*Tao Lei[1†], Theresa Appleson[1†], Alexander Breder[1]**

[1]Institut für Organische Chemie, Universität Regensburg, Universitätstrasse 31

93053 Regensburg (Germany)

Corresponding Author E-mail: alexander.breder@ur.de

†T.L. and T.A. contributed equally to this work.

Table of Contents

General Remarks 3
Synthesis of Substrates and catalysts 4
Optimization of the Photo-Aerobic Intermolecular *N*-allylation reaction 17
Investigation Toward the Isomerization of the Allylic Products 32
Initial Study of the Assymetric Intermolecular *N*-Allylation Reaction 35
Substrate Scope of the Internal *N*-Allylation Reaction 38
References 73
Characterization Data of Substrates and Products 74
HPLC Data of Chiral Products 199
Author Contributions 203

General Remarks

Commercially available reagents were used without further purification. Solvents were used in p.a. quality or dried according to common procedures if necessary. Purity is estimated to be ≥95% based on ^{1}H-NMR spectroscopic analysis. Irradiation experiments were performed using custom built temperature-controlled metal blocks and commercially available blue LED lights (LT 1013 royal-blue) operating at a constant current (700 mA) with an intensity maximum at λ = 448 nm and an output power of 414 – 520 mW. TLC: MACHEREY-NAGEL, TLC plates Alugram® Sil G/UV254. Visualization of TLC was enabled by exposure to UV light [UV] (λ = 254 nm); by treatment with permanganate stain [$KMnO_4$] (composition: 3 g potassium permanganate, 20 g potassium carbonate, 5 mL 5% aqueous NaOH, 300 mL H_2O) or *p*-anisaldehyde stain [*p*-anisaldehyde] (composition: 270 mL EtOH, 7,4 mL *p*-anisaldehyde, 10 mL H_2SO_4 conc., 3 mL AcOH). Chromatography: Separations were carried out manually using forced flow or via an automated flash column (ADVION, *puriFlash*® 5.050) on Acros Silica 60 (0.035-0.075 mm, 70-230 mesh ASTM). NMR spectra were recorded at 300 MHz (^{1}H) and 75 MHz (^{13}C) on a Bruker Avance 300 spectrometer or at 400 MHz (^{1}H), 101 MHz (^{13}C), 377 MHz (^{19}F), 162 MHz (^{31}P) on a Bruker Avance III HD 400, if not otherwise specified. Chemical shifts (δ) are given in parts per million (ppm) and referenced to the residual proton signal of the used solvent: $CDCl_3$ (δ = 7.26 ppm, ^{1}H; δ = 77.16 ppm, ^{13}C). Multiplicity (s = singlet, d = doublet, t = triplet, m = multiplet, and combinations of these). Isomeric ratios (*E*/*Z*) were determined by the ratio of ^{1}H NMR integrals of the isolated products. For ^{1}H NMR yield determination, the solvent of reaction mixture was removed under reduced pressure after reaction was completed. The internal standard was added, and the residue was taken up in $CDCl_3$. Melting point: A.KRÜSS melting point meter M5000. IR: Agilent Technologies Cary 630 FT-IR spectrometer. High resolution mass spectrometry (HRMS): Agilent Q-TOF 6540 UHD. High performance liquid chromatography (HPLC): Agilent 1260 Infinity using 4.6 mm × 25 cm Daicel CHIRALPAK IA-3, IC-3, ID-3, OD-3. Optical rotations: Jasco P-2000 polarimeter.

Synthesis of Substrates and catalysts

Scope of alkenes:

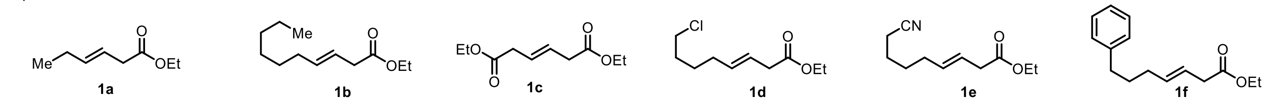

1a 1b 1c 1d 1e 1f

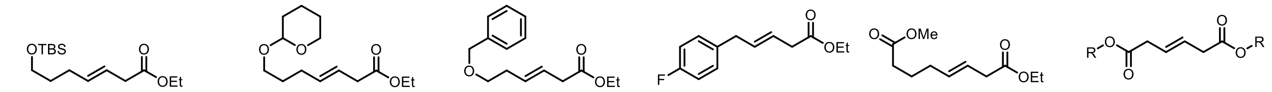

1g 1h 1i 1j 1k

1l, Me; **1m**, ^{i}Pr; **1n**, Bn; **1o**, Cy; **1p**, Ph; **1q**, C_7H_{15}

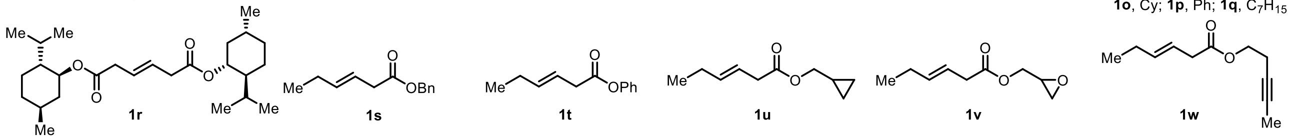

1r 1s 1t 1u 1v 1w

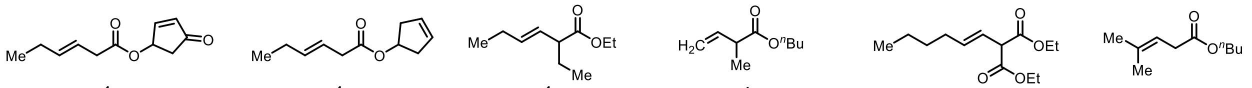

1x 1y 1z 1aa 1ab 1ac

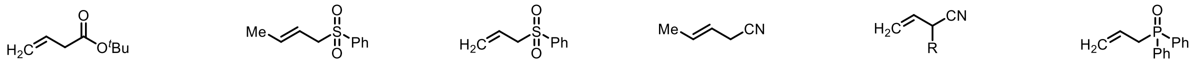

1ad 1ae 1af 1ag **1ah**, H; **1ai**, Me 1aj

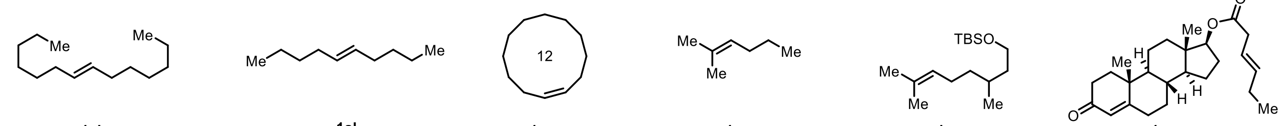

1ak 1al 1am 1an 1ao 1ap

Scope of *N*-nuclophiles:

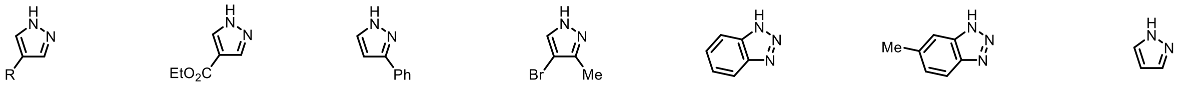

2a, Cl; **2b**, I 2c 2d 2e 2f 2g 2h

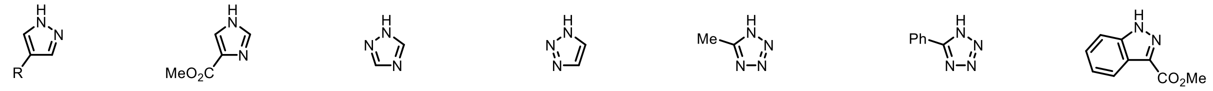

2i, Me; **2j**, CF_3 2k 2l 2m 2n 2o 2p

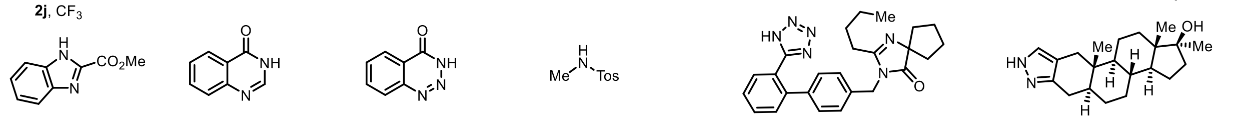

2q 2r 2s 2t 2u 2v

Scope of Se-catalysts:

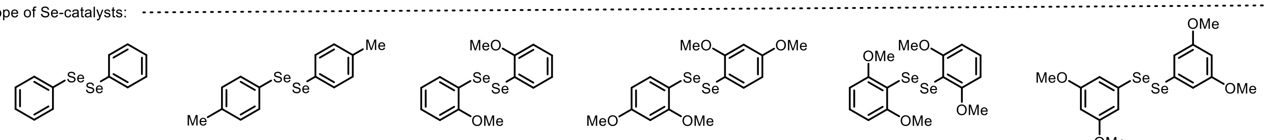

$(PhSe)_2$ $(4\text{-MePhSe})_2$ $(2\text{-MeOPhSe})_2$ $(2,4\text{-diMeOPhSe})_2$ $(2,6\text{-diMeOPhSe})_2$ $(3,5\text{-diMeOPhSe})_2$

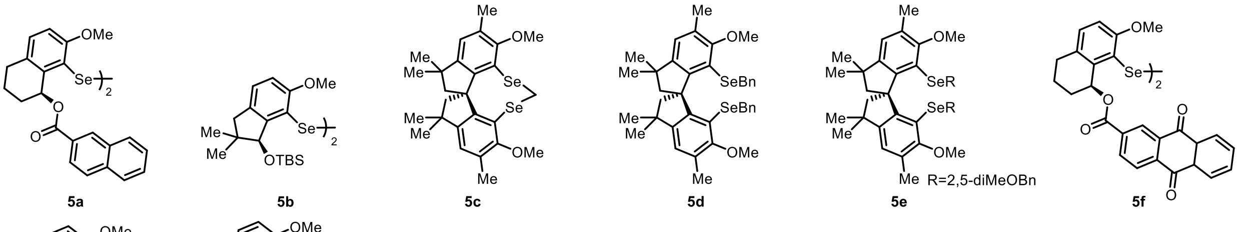

5a 5b 5c 5d 5e 5f

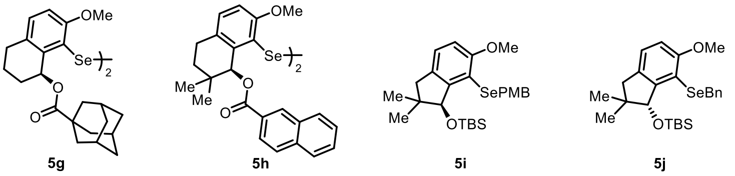

5g 5h 5i 5j

Ethyl (*E*)-hex-3-enoate (**1a**), diethyl (*E*)-hex-3-enedioate (**1c**), *tert*-butyl but-3-enoate (**1ad**), (*E*)-(but-2-en-1-ylsulfonyl)benzene (**1ae**), (allylsulfonyl)benzene (**1af**), pent-3-enenitrile (**1ag**), but-3-enenitrile (**1ah**), 2-methylbut-3-enenitrile (**1ai**), allyldiphenylphosphine oxide (**1aj**), (*E*)-7-tetradecene (**1ak**), (*E*)-5-decene (**1al**), cyclododecene (*E*/*Z* mixture, **1am**), 2-methylhex-2-ene (**1an**) were commercial. **1b** to **1ac**, **1ao**, **1ap** were synthesized according to following procedures. All *N*-nucleophiles were commercial. As for selenium catalysts, $(PhSe)_2$ was commercial. **1s** and **1t**,[1] $(4\text{-}MePhSe)_2$,[2] $(2\text{-}MeOPhSe)_2$,[2] $(2,4\text{-}dIMeOPhSe)_2$,[2] $(2,6\text{-}diMeOPhSe)_2$,[2] $(3,5\text{-}diMeOPhSe)_2$,[2] **5c** to **5e**,[3] **5a** and **5f** to **5h**,[4] **5b** and **5i** and **5j**[5] were synthesized according to literatures.

Ethyl (*E*)-dec-3-enoate (1b):

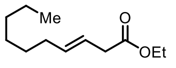

1b

(*E*)-dec-3-enoic acid (1.70 g, 10.0 mmol, 1.00 equiv.), DMAP (122 mg, 1.00 mmol, 10 mol%) and EtOH (921 g, 20.0 mmol, 2.00 equiv.) were dissolved in DCM (20 mL). At 0 °C, DCC (3.09 g, 15.0 mmol, 1.50 equiv.) in DCM (5 mL) was added and the resulting solution was stirred at 0 °C for 10 min and at r.t. overnight. AcOH (360 mg, 6.00 mmol, 0.60 equiv.) was added and stirring was continued at r.t. for 4 h. Then, hexane (30 mL) was added and the mixture was stirred at r.t. for another 10 min. After filtration via short silica column (eluted with Et_2O), the solvent was removed under reduced pressure. The residue was purified via column chromatography (PE/EtOAc, 60:1 to 50:1) to afford the target compound **1b** as a colorless oil (1.77 g, 8.90 mmol, 89%).

TLC R_f = 0.67 (PE/EtOAc, 10:1) [$KMnO_4$]. **IR** [cm^{-1}]: 2926, 2855, 1737, 1461, 1372, 1245, 1156, 1118, 1029, 969, 857, 727. **^{1}H NMR** (300 MHz, $CDCl_3$): δ 5.65 – 5.41 (m, 2H), 4.13 (q, *J* = 7.1 Hz, 2H), 3.11 – 2.91 (m, 2H), 2.12 – 1.92 (m, 2H), 1.37 – 1.15 (m, 11H), 0.87 (t, *J* = 6.7 Hz, 3H). **^{13}C NMR** (75 MHz, $CDCl_3$): δ 172.3, 134.9, 121.5, 60.5, 38.2, 32.5, 31.7, 29.1, 28.8, 22.6, 14.2, 14.1. **HRMS** (EI) calcd. for $[C_{12}H_{22}O_2]^{\cdot+}$ ($[M]^{\cdot+}$), m/z = 198.1614; found 198.1610.

Ethyl (*E*)-8-chlorooct-3-enoate (1d):

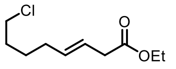

1d

Step 1: To a solution of oxalyl dichloride (3.81 g, 2.58 mL, 30.0 mmol, 1.50 equiv.) in dry DCM (40 mL) under a N_2 atmosphere DMSO (3.13 g, 2.84 mL, 40.0 mmol, 2.00 equiv.) in dry DCM (5 mL) was added carefully at -78 °C (note: strong gas release). The resulting mixture was continued to stir at -78 °C for 20 min. Then, 6-chlorohexan-1-ol (2.73 g, 20.0 mmol, 1.00 equiv.) in dry DCM (5 mL) was added dropwise and the solution was stirred at -78 °C for 30 min. After that, dry NEt_3 (6.07 g, 8.40 mL, 60.0 mmol, 3.00 equiv.) was added dropwise. The solution was stirred at -78 °C for 20 min and at r.t. overnight, before H_2O (50 mL) and DCM (200 mL) were added. The layers were separated, and the organic phase was washed with H_2O (3 x 100 mL), dried over Na_2SO_4, and filtered. The solvent was removed under reduced pressure and the residue was purified via column chromatography (pentane/EtOAc, 40:1 to 20:1) to afford crude 6-chlorohexanal.

Step 2: Piperidine (85.2 mg, 99 μL, 1.00 mmol, 5 mol%) and AcOH (60.1 mg, 57 μL, 1.00 mmol, 5 mol%) were added to a solution of malonic acid (6.24 g, 60.0 mmol, 3.00 equiv.) in DMSO (28 mL). After the mixture was heated to 65 °C, crude 6-chlorohexanal (1.00 equiv., from step 1) in DMSO (2 mL) was added slowly. The mixture was stirred at 75 °C for 24 h. After cooling to r.t., the reaction was quenched via the addition of H_2O (50 mL) and Et_2O (250 mL). The layers were separated, and the organic phase was washed with H_2O (5 x 50 mL), dried over Na_2SO_4, and filtered. The solvent was removed under reduced pressure and the residue was purified via column chromatography (PE/Et_2O, 1:1) to afford (*E*)-8-chlorooct-3-enoic acid as a lightly yellow oil (1.38 g, 7.80 mmol, 39%).

Step 3: (*E*)-8-chlorooct-3-enoic acid (883 mg, 5.00 mmol, 1.00 equiv., from step 2), DMAP (61.1 mg, 500 umol, 10 mol%) and EtOH (276 mg, 6.00 mmol, 1.20 equiv.) were dissolved in DCM (20 mL). At 0 °C, DCC (1.55 g, 7.50 mmol, 1.50 equiv.) in DCM (10 mL) was added. The resulting solution was stirred at 0 °C for 10 min and at r.t. overnight. AcOH (0.5 mL) and

EtOH (0.5 mL) were added and stirring was continued at r.t. for 30 min. Then, hexane (30 mL) was added, and the mixture was stirred at r.t. for another 10 min. After filtration via short silica column (eluted with Et_2O), the solvent was removed under reduced pressure. The residue was purified via column chromatography (PE/EtOAc, 60:1) to afford the target compound **1d** as a lightly yellow oil (934 mg, 4.56 mmol, 91% in the third step).

TLC R_f = 0.46 (PE/EtOAc, 10:1) [$KMnO_4$]. **IR** [cm^{-1}]: 2982, 2937, 2863, 1733, 1446, 1372, 1249, 1163, 1029, 969, 857, 723. **^{1}H NMR** (300 MHz, $CDCl_3$): δ 5.63 – 5.44 (m, 2H), 4.13 (q, J = 7.2 Hz, 2H), 3.52 (t, J = 6.7 Hz, 2H), 3.10 – 2.93 (m, 2H), 2.14 – 2.01 (m, 2H), 1.84 – 1.69 (m, 2H), 1.58 – 1.42 (m, 2H), 1.25 (t, J = 7.2 Hz, 3H). **^{13}C NMR** (75 MHz, $CDCl_3$): δ172.1, 133.8, 122.4, 60.6, 44.9, 38.1, 32.0, 31.7, 26.3, 14.2. **HRMS** (ESI) calcd. for $[C_{10}H_{18}ClO_2]^+$ ($[M+H]^+$), m/z = 205.0990; found 205.0991.

Ethyl (*E*)-8-cyanooct-3-enoate (1e):

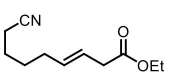

1e

Step 1: To a solution of oxalyl dichloride (3.81 g, 2.58 mL, 30.0 mmol, 1.50 equiv.) in dry DCM (40 mL) under a N_2 atmosphere DMSO (3.13 g, 2.84 mL, 40.0 mmol, 2.00 equiv.) in dry DCM (5 mL) was added carefully at -78 °C (note: strong gas release). The resulting mixture was continued to stir at -78 °C for 20 min. Then, 7-hydroxyheptanenitrile (2.54 g, 20.0 mmol, 1.00 equiv.) in dry DCM (5 mL) was added dropwise and the solution was stirred at -78 °C for 30 min. After that, dry NEt_3 (6.07 g, 8.40 mL, 60.0 mmol, 3.00 equiv.) was added dropwise. The solution was stirred at -78 °C for 20 min and at r.t. overnight, before H_2O (50 mL) and DCM (200 mL) were added. The layers were separated, and the organic phase was washed with H_2O (3 x 100 mL), dried over Na_2SO_4, and filtered. The solvent was removed under reduced pressure and the residue was purified via column chromatography (pentane/EtOAc, 40:1 to 20:1) to afford crude 7-oxoheptanenitrile.

Step 2: Piperidine (85.2 mg, 99 µL, 1.00 mmol, 5 mol%) and AcOH (60.1 g, 57 µL, 1.00 mmol, 5 mol%) were added to a solution of malonic acid (6.24 g, 60.0 mmol, 3.00 equiv.) in DMSO (28 mL). After the solution was heated to 65 °C, crude 7-oxoheptanenitrile (1.00 equiv., from step 1) in DMSO (2 mL) was added slowly. The mixture was stirred at 75 °C for 24 h. After cooling to r.t., the reaction was quenched via the addition of water (50 mL) and Et_2O (250 mL). The layers were separated, and the organic phase was washed with H_2O (5 x 50 mL), dried over Na_2SO_4, and filtered. The solvent was removed under reduced pressure and the residue was purified via column chromatography (PE/Et_2O, 1:1) to afford (*E*)-8-chlorooct-3-enoic acid as a lightly yellow oil (980 mg, 5.86 mmol, 29%).

Step 3: (*E)*-8-cyanooct-3-enoic acid (980 mg, 5.86 mmol, 1.00 equiv., from step 2), DMAP (71.6 mg, 586 µmol, 10 mol%) and EtOH (540 mg, 11.7 mmol, 2.00 equiv.) was dissolved in DCM (20 mL). At 0 °C, DCC (1.82 g, 8.79 mmol, 1.50 equiv.) in DCM (10 mL) was added. The resulting solution was stirred at 0 °C for 10 min and at r.t. overnight. AcOH (0.5 mL) and EtOH (0.5 mL) were added and stirring was continued at r.t. for 30 min. Then, hexane (30 mL) was added, and the mixture stirred at r.t. for another 10 min. After filtration via short silica column (eluted with Et_2O), the solvent was removed under reduced pressure. The residue was purified via column chromatography (PE/EtOAc, 30:1 to 10:1) to afford the target compound **1e** as a lightly yellow oil (995 mg, 5.10 mmol, 87% in the third step).

TLC R_f = 0.46 (PE/EtOAc, 5:1) [$KMnO_4$]. **IR** [cm^{-1}]: 2982, 2937, 2866, 2248, 1730, 1446, 1371, 1327, 1297,1249, 1178, 1100, 1029, 969, 857, 738. **^{1}H-NMR** (300 MHz, $CDCl_3$): δ 5.66 – 5.38 (m, 2H), 4.12 (q, J = 7.1 Hz, 2H), 3.09 – 2.92 (m, 2H), 2.33 (t, J = 6.9 Hz, 2H), 2.14 – 1.99 (m, 2H), 1.72 – 1.59 (m, 2H), 1.59 – 1.47 (m, 2H), 1.25 (t, J = 7.1 Hz, 3H). **^{13}C-NMR** (75 MHz, $CDCl_3$): δ 172.0, 133.2, 122.9, 119.7, 60.6, 38.0, 31.5, 28.0, 24.7, 17.0, 14.2. **HRMS** (ESI) calcd. for $[C_{11}H_{18}NO_2]^+$ ($[M+H]^+$), m/z = 196.1332; found 196.1333.

Ethyl (*E*)-7-phenylhept-3-enoate (1f):

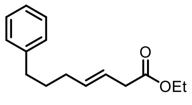

1f

Step 1: To a solution of oxalyl dichloride (3.81 g, 2.58 mL, 30.0 mmol, 2.46 equiv.) in dry DCM (40 mL) under a N_2 atmosphere DMSO (3.13 g, 2.84 mL, 40.0 mmol, 3.28 equiv.) in dry DCM (5 mL) was added carefully at -78 °C (note: strong gas release). The resulting mixture was continued to stir at -78 °C for 20 min. Then, 5-phenylpentan-1-ol (2.00 g, 12.2 mmol, 1.00 equiv.) in dry DCM (5 mL) was added dropwise and the solution was stirred at -78 °C for 30 min. After that, dry NEt_3 (6.07 g, 8.40 mL, 60.0 mmol, 4.92 equiv.) was added dropwise. The solution was stirred at -78 °C for 20 min and at r.t. overnight, before H_2O (50 mL) and DCM (200 mL) were added. The layers were separated, and the organic phase was washed with H_2O (3 x 100 mL), dried over Na_2SO_4, and filtered. The solvent was removed under reduced pressure and the residue was purified via column chromatography (Pentane/EtOAc, 40:1 to 20:1) to afford crude 5-phenylpentanal.

Step 2: To abovementioned crude 5-phenylpentanal (1.00 equiv., from step 1), 3-ethoxy-3-oxopropanoic acid (1.93 g, 1.73 mL, 14.6 mmol, 1.20 equiv.) and NEt_3 (1.48 g, 2.04 mL, 14.6 mmol, 1.20 equiv.) were added. The mixture was heated to 85 °C and stirred for 14 h. After the reaction was cooled down to r.t., H_2O (50 mL) and EtOAc (100 mL) were added. At 0 °C, 1 M HCl (aq., 50 mL) was added. The layers were separated, and the water phase was extracted with EtOAc (100 mL). The combined organic phases were washed with H_2O and brine, dried over Na_2SO_4, and filtered. The solvent was removed under reduced pressure and the residue was purified via column chromatography (PE/EtOAc, 60:1) to afford the target compound **1f** as a colorless oil (1.17 g, 3.50 mmol, 29%).

TLC R_f = 0.75 (PE/EtOAc, 5:1) [UV, $KMnO_4$]. **IR** [cm^{-1}]: 3027, 2982, 2930, 2855, 1733, 1603, 1495, 1454, 1368, 1245, 1174, 1029, 969, 857, 746, 701. **^{1}H-NMR** (300 MHz, $CDCl_3$): δ 7.38 – 7.32 (m, 2H), 7.29 – 7.21 (m, 3H), 5.74 – 5.55 (m, 2H), 4.22 (q, *J* = 7.2 Hz, 2H), 3.16 – 3.05 (m, 2H), 2.74 – 2.64 (m, 2H), 2.21 – 2.11 (m, 2H), 1.84 – 1.74 (m, 2H), 1.34 (t, *J* = 7.1 Hz, 3H). **^{13}C-NMR** (75 MHz, $CDCl_3$): δ 172.2, 142.4, 134.2, 128.5, 128.3, 125.7, 122.2, 60.6, 38.2, 35.3, 32.0, 30.8, 14.2. **HRMS** (ESI) calcd. for $[C_{15}H_{21}O_2]^+$ ($[M+H]^+$), m/z = 233.1536; found 233.1540.

Ethyl (*E*)-7-((*tert*-butyldimethylsilyl)oxy)hept-3-enoate (1g):

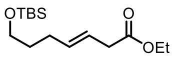

1g

Step 1: To a solution of oxalyl dichloride (3.56 g, 2.40 mL, 28.1 mmol, 1.50 equiv.) in dry DCM (40 mL) under a N_2 atmosphere DMSO (2.93 g, 2.65 mL, 37.4 mmol, 2.00 equiv.) in dry DCM (5 mL) was added carefully at -78 °C (note: strong gas release). The resulting mixture was continued to stir at -78 °C for 20 min. Then, 5-((*tert*-butyldimethylsilyl)oxy)pentan-1-ol (4.09 g, 18.7 mmol, 1.00 equiv.) in dry DCM (5 mL) was added dropwise and the solution was stirred at -78 °C for 30 min. After that, dry NEt_3 (5.68 g, 7.82 mL, 56.1 mmol, 3.00 equiv.) was added dropwise. The solution was stirred at -78 °C for 20 min and at r.t. overnight, before H_2O (50 mL) and DCM (200 mL) were added. The layers were separated, and the organic phase was washed with H_2O (2 x 100 mL), saturated NH_4Cl (aq., 100 mL), brine, dried over Na_2SO_4, and filtered. Removal of the solvent afforded crude 5-((*tert*-butyldimethylsilyl)oxy)pentanal without further purification.

Step 2: To abovementioned crude 5-((*tert*-butyldimethylsilyl)oxy)pentanal (1.00 equiv., from step 1), 3-ethoxy-3-oxopropanoic acid (3.36 g, 3.00 mL, 25.4 mmol, 1.36 equiv.) and NEt_3 (3.63 g, 5.00 mL, 49.4 mmol, 2.64 equiv.) were added. The mixture was heated to 90°C and stirred for 12 h. After the reaction was cooled down to r.t., H_2O (50 mL) and EtOAc (100 mL) were added. At 0 °C, 1 M HCl (aq., 50 mL) was added. The layers were separated, and the water phase was extracted with EtOAc (100 mL). The combined organic phases were washed with H_2O and brine, dried over Na_2SO_4, and filtered. The solvent was removed under reduced pressure and the residue was purified via column chromatography (PE/EtOAc, 100:1 to 60:1) to afford target compound **1g** as a colorless oil (2.69 g, 9.40 mmol, 50%).

TLC R$_f$ = 0.58 (PE/EtOAc, 10:1) [$KMnO_4$]. **IR** [cm^{-1}]: 2930, 2896, 2859, 1737, 1472, 1387, 1252, 1156, 1096, 1033, 969, 835, 775, 716, 664. **^{1}H-NMR** (300 MHz, $CDCl_3$): δ5.64 – 5.45 (m, 2H), 4.13 (q, J = 7.1 Hz, 2H), 3.60 (t, J = 6.4 Hz, 2H), 3.09 – 2.92 (m, 2H), 2.15 – 2.03 (m, 2H), 1.64 – 1.53 (m, 2H), 1.25 (t, J = 7.1 Hz, 3H), 0.88 (s, 9H), 0.04 (s, 6H). **^{13}C-NMR** (75 MHz, $CDCl_3$): δ 172.2, 134.2, 122.0, 62.5, 60.5, 38.2, 32.3, 28.8, 26.0, 18.4, 14.2, -5.3. **HRMS** (ESI) calcd. for $[C_{15}H_{31}O_3Si]^+$ ($[M+H]^+$), m/z = 287.2037; found 287.2037.

Ethyl (*E*)-7-((tetrahydro-2H-pyran-2-yl)oxy)hept-3-enoate (1h):

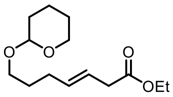

1h

Step 1: To a solution of oxalyl dichloride (3.64 g, 2.45 mL, 28.7 mmol, 1.50 equiv.) in dry DCM (40.0 mL) under a N_2 atmosphere DMSO (2.99 g, 2.71 mL, 38.2 mmol, 2.00 equiv.) in dry DCM (5 mL) was added carefully at -78 °C (note: strong gas release). This mixture was continued to stir at -78 °C for 20 min. Then, 5-((tetrahydro-2H-pyran-2-yl)oxy)pentan-1-ol (3.59 g, 19.1 mmol, 1.00 equiv.) in dry DCM (5.00 mL) was added dropwise and the solution was stirred at -78 °C for 30 min. After that, dry NEt_3 (5.80 g, 7.96 mL, 57.3 mmol, 3.00 equiv.) was added dropwise. The solution was stirred at -78 °C for 20 min and at r.t. overnight, before H_2O (50 mL) and DCM (200 mL) were added. The layers were separated, and the organic phase was washed with H_2O (2 x 100 mL), saturated NH_4Cl (aq. 100 mL), brine, dried over Na_2SO_4, and filtered. Removal of the solvent afforded crude 5-((tetrahydro-2H-pyran-2-yl)oxy)pentanal without further purification.

Step 2: To abovementioned crude 5-((tetrahydro-2H-pyran-2-yl)oxy)pentanal (1.00 equiv., from step 1), 3-ethoxy-3-oxopropanoic acid (3.03 g, 2.71 mL, 22.9 mmol, 1.20 equiv.) and NEt_3 (3.63 g, 5.00 mL, 49.4 mmol, 2.59 equiv.) were added. The mixture was heated to 90 °C and stirred for 12 h. After the reaction was cooled down to r.t., H_2O (50 mL) and EtOAc (100 mL) were added. At 0 °C, 1 M HCl (aq., 50 mL) was added. The layers were separated, and the water phase was extracted with EtOAc (100 mL). The combined organic phases were washed with H_2O and brine, dried over Na_2SO_4, and filtered. The solvent was removed under reduced pressure and the residue was purified via column chromatography (PE/EtOAc, 20:1 to 15:1) to afford target compound **1h** as a colorless oil (2.53 g, 9.86 mmol, 52%).

TLC R$_f$ = 0.54 (PE/EtOAc, 5:1) [$KMnO_4$]. **IR** [cm^{-1}]: 2941, 2870, 1737, 1443, 1368, 1252, 1156, 1077, 1021, 969, 902, 869, 813, 693. **^{1}H-NMR** (400 MHz, $CDCl_3$): δ 5.66 – 5.47 (m, 2H), 4.59 – 4.52 (m, 1H), 4.12 (q, J = 7.2 Hz, 2H), 3.85 (ddd, J = 11.2, 7.7, 3.2 Hz, 1H), 3.73 (dt, J = 9.5, 6.6 Hz, 1H), 3.53 – 3.44 (m, 1H), 3.38 (dt, J = 9.7, 6.6 Hz, 1H), 3.06 – 2.93 (m, 2H), 2.19 – 2.07 (m, 2H), 1.88 – 1.76 (m, 1H), 1.74 – 1.62 (m, 3H), 1.59 – 1.47 (m, 4H), 1.25 (td, J = 7.2, 0.6 Hz, 3H). **^{13}C-NMR** (101 MHz, $CDCl_3$): δ 172.1, 134.0, 122.1, 98.8, 66.9, 62.3, 60.5, 38.2, 30.7, 29.2, 25.5, 19.6, 14.2. **HRMS** (ESI) calcd. for $[C_{14}H_{25}O_4]^+$ ($[M+H]^+$), m/z = 257.1747; found 257.1745.

Ethyl (*E*)-6-(benzyloxy)hex-3-enoate (1i):

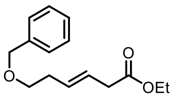

1i

(*E*)-6-(benzyloxy)hex-3-enoic acid[5] (955 mg, 4.33 mmol, 1.00 equiv.), DMAP (52.9 mg, 433 µmol, 10 mol%) and EtOH (399 mg, 8.66 mmol, 2.00 equiv.) were dissolved in DCM (20 mL). At 0 °C, DCC (1.34 g, 6.50 mmol, 1.50 equiv.) in DCM (5 mL) was added. The resulting solution was stirred at 0 °C for 10 min and at r.t. overnight. AcOH (156 mg, 149 µL, 2.60 mmol, 0.60 equiv.) was added and stirring was continued at r.t. for 1 h. Then, hexane (30 mL) was added, and the mixture was stirred at r.t. for another 10 min. After filtration via short silica column (eluted with Et_2O), the solvent was removed under reduced pressure. The residue was purified via column chromatography (PE/EtOAc, 50:1 to 24:1) to afford target compound **1i** as a colorless oil (788 mg, 3.17 mmol, 73%).

TLC R$_f$ = 0.33 (PE/EtOAc, 10:1) [UV, $KMnO_4$]. **IR** [cm^{-1}]: 3373, 2937, 2859, 2907, 2859, 1733, 1454, 1368, 1252, 1200, 1159, 1096, 1029, 969, 857, 809, 738, 697. **^{1}H-NMR** (300 MHz, $CDCl_3$): δ 7.39 – 7.24 (m, 5H), 5.72 – 5.50 (m, 2H), 4.51 (s, 2H), 4.13 (q, J = 7.3 Hz, 2H), 3.51 (t, J = 6.7 Hz, 2H), 3.09 – 3.00 (m, 2H), 2.42 – 2.33 (m, 2H), 1.26 (t, J = 7.1 Hz, 3H).

^{13}C-NMR (75 MHz, $CDCl_3$): δ 172.0, 138.4, 130.8, 128.4, 127.7, 127.6, 123.8, 72.9, 69.7, 60.6, 38.2, 33.0, 14.2. **HRMS** (ESI) calcd. for $[C_{15}H_{21}O_3]^+$ ($[M+H]^+$), m/z = 249.1485; found 249.1486.

Ethyl (*E*)-5-(4-fluorophenyl)pent-3-enoate (1j):

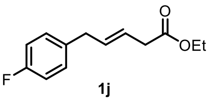

1j

(*E*)-5-(4-fluorophenyl)pent-3-enoic acid[5] (1.55 g, 8.00 mmol, 1.00 equiv.), DMAP (97.7 mg, 0.80 mmol, 10 mol%) and EtOH (737 mg, 934 µL, 16.0 mmol, 2.00 equiv.) were dissolved in DCM (20 mL). At 0 °C, DCC (2.48 g, 12.0 mmol, 1.50 equiv.) in DCM (5 mL) was added. The resulting solution was stirred at 0 °C for 10 min and at r.t. overnight. AcOH (288 mg, 274 µL, 4.80 mmol, 0.60 equiv.) was added and stirring was continued at r.t. for 1 h. Then, hexane (30 mL) was added, and the mixture was stirred at r.t. for another 10 min. After filtration via short silica column (eluted with Et_2O), the solvent was removed under reduced pressure. The residue was purified via column chromatography (PE/EtOAc, 50:1 to 40:1) to afford target compound **1j** as a colorless oil (1.74 g, 7.83 mmol, 98%).

TLC R_f = 0.47 (PE/EtOAc, 10:1) [UV, $KMnO_4$]. **IR** [cm^{-1}]: 3042, 2982, 2907, 1890, 1733, 1603, 1510, 1435, 1372, 1305, 1219, 1179, 1096, 1029, 969, 820, 708. **^{1}H NMR** (300 MHz, $CDCl_3$): δ 7.18 – 7.09 (m, 2H), 7.02 – 6.91 (m, 2H), 5.77 – 5.53 (m, 2H), 4.15 (q, J = 7.1 Hz, 2H), 3.35 (d, J = 5.8 Hz, 2H), 3.11 – 3.02 (m, 2H), 1.26 (t, J = 7.1 Hz, 3H). **^{13}C NMR** (75 MHz, $CDCl_3$): δ 171.9, 161.4 (d, J_{C-F} = 244.0 Hz), 135.7 (d, J_{C-F} = 2.9 Hz), 133.0, 129.9 (d, J_{C-F} = 7.7 Hz), 123.53, 115.2 (d, J_{C-F} = 21.4 Hz), 60.7, 38.1, 38.0, 14.2. **^{19}F NMR** (377 MHz, $CDCl_3$) δ -117.9. **HRMS** (EI) calcd. for $[C_{13}H_{15}O_2F]^{\cdot+}$ ($[M^{\cdot+}]$), m/z = 222.1051; found 222.1055.

1-Ethyl 8-methyl (*E*)-oct-3-enedioate (1k):

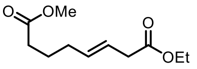

1k

Step 1: To a solution of oxalyl dichloride (3.81 g, 2.58 mL, 30.0 mmol, 1.50 equiv.) in dry DCM (40 mL) under a N_2 atmosphere DMSO (3.13 g, 2.84 mL, 40.0 mmol, 2.00 equiv.) in dry DCM (5 mL) was added carefully at -78 °C (note: strong gas release). The resulting mixture was continued to stir at -78 °C for 20 min. Then, methyl 6-hydroxyhexanoate (2.92 g, 20.0 mmol, 1.00 equiv.) in dry DCM (5 mL) was added dropwise and the solution was stirred at -78 °C for 30 min. After that, dry NEt_3 (6.07 g, 8.40 mL, 60.0 mmol, 3.00 equiv.) was added dropwise. The solution was stirred at -78 °C for 20 min and at r.t. overnight, before H_2O (50 mL) and DCM (250 mL) were added. The organic phase was washed with H_2O (3 x 50 mL), dried over Na_2SO_4, and filtered. The solvent was removed under reduced pressure and the residue was purified via column chromatography (pentane/EtOAc, 20:1 to 10:1) to afford crude methyl 7-oxoheptanoate.

Step 2: Piperidine (85.2 mg, 99 µL, 1.00 mmol, 5 mol%) and AcOH (60.1 g, 57 µL, 1.00 mmol, 5 mol%) were added to a solution of malonic acid (6.24 g, 60.0 mmol, 3.00 equiv.) in DMSO (28.0 mL). After the solution was heated to 65 °C, crude methyl 7-oxoheptanoate (1.00 equiv., from step 1) in DMSO (2 mL) was added slowly. The mixture was stirred at 75 °C for 24 h. After cooling to r.t., the reaction was quenched via the addition of water (50 mL) and Et_2O (250 mL). The layers were separated, and the organic phase was washed with H_2O (5 x 50 mL), dried over Na_2SO_4, and filtered. The solvent was removed under reduced pressure and the residue was purified via column chromatography (PE/Et_2O, 1:1) to get (*E*)-9-methoxy-9-oxonon-3-enoic acid as a colorless oil (1.48 mg, 7.39 mmol, 37%).

Step 3: (*E*)-9-methoxy-9-oxonon-3-enoic acid (1.48 mg, 7.39 mmol, 1.00 equiv., from step 2), DMAP (45.1 mg, 370 umol, 5 mol%) and EtOH (374.5 mg, 8.13 mmol, 1.10 equiv.) were dissolved in DCM (40.0 mL). At 0 °C, DCC (2.29 g, 11.1 mmol, 1.50 equiv.) in DCM (10 mL) was added. The resulting solution was stirred at 0 °C for 10 min and at r.t. overnight. AcOH (0.5 mL) and EtOH (0.5 mL) were added and stirring was continued at r.t. for 30 min. Then, hexane (30 mL) was added and the mixture was stirred at r.t. for another 10 min. After filtration via short silica column (eluted with Et_2O), the solvent was

removed under reduced pressure. The residue was purified via column chromatography (PE/EtOAc, 20:1 to 15:1) to afford the target compound **1k** as a colorless oil (1.61 mg, 7.51 mmol, 38% over three steps).

TLC R_f = 0.43 (PE/EtOAc, 5:1) [$KMnO_4$]. **IR** [cm^{-1}]: 2982, 2952, 1733, 1439, 1372, 1029, 1163, 1096, 1029, 969, 857, 787, 701. **^{1}H NMR** (300 MHz, $CDCl_3$): δ 5.62 – 5.42 (m, 2H), 4.12 (q, *J* = 7.1 Hz, 2H), 3.65 (s, 3H), 3.05 – 2.95 (m, 2H), 2.30 (t, *J* = 7.5 Hz, 2H), 2.11 – 2.01 (m, 2H), 1.77 – 1.63 (m, 2H), 1.24 (t, *J* = 7.1 Hz, 3H). **^{13}C NMR** (75 MHz, $CDCl_3$): δ 174.0, 172.0, 133.3, 122.9, 60.6, 51.5, 38.1, 33.3, 31.8, 24.3, 14.2. **HRMS** (ESI) calcd. for $[C_{11}H_{19}O_4]^+$ ($[M+H]^+$), m/z = 215.1278; found 215.1277.

Dimethyl (*E*)-hex-3-enedioate (1l):

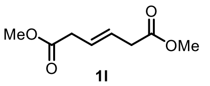

1-Ethyl-3-(3-dimethylaminopropyl)carbodiimide (5.75 g, 30.0 mmol, 3.00 equiv.), DMAP (122 mg, 1.00 mmol, 10 mol%) and MeOH (705 mg, 890 µL, 22.0 mmol, 2.20 equiv.) were added to a solution of (*E*)-hex-3-enedioic acid (1.44 g, 10.0 mmol, 1.00 equiv.) in DCM (20 mL) at 0 °C. The resulting mixture was stirred at 0 °C for 10 min and at r.t. for 4 h. Then, DCM (50 mL) was added, and the solution was washed with 1% HCl (aq.) (50 mL) and brine. The layers were separated, and the organic phase was dried over Na_2SO_4 and filtered. The solvent was removed under reduced pressure and the residue was purified via column chromatography (PE/EtOAc, 10:1 to 8:1) to afford target compound **1l** as a colorless oil (1.25 g, 7.26 mmol, 73%)

TLC R_f = 0.17 (PE/EtOAc, 10:1) [$KMnO_4$]. **IR** [cm^{-1}]: 3403, 3004, 2956, 1733, 1439, 1364, 1252, 1197, 1156, 1092, 973, 928, 891, 846, 716. **^{1}H NMR** (300 MHz, $CDCl_3$): δ 5.79 – 5.55 (m, 2H), 3.68 (s, 6H), 3.14 – 3.05 (m, 4H). **^{13}C NMR** (75 MHz, $CDCl_3$): δ 172.0, 125.9, 51.9, 37.7. **HRMS** (EI) calcd. for $[C_8H_{12}O_4]^{\cdot+}$ ($[M]^{\cdot+}$), m/z = 172.0730; found 172.0733.

Diisopropyl (E)-hex-3-enedioate (1m):

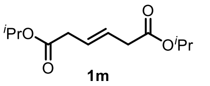

(*E*)-hex-3-enedioic acid (1.44 g, 10.0 mmol, 1.00 equiv.), DMAP (122 mg, 1.00 mmol, 10 mol%) and propan-2-ol (1.32 g, 22.0 mmol, 2.20 equiv.) were dissolved in DCM (20 mL). At 0 °C, DCC (4.33 g, 21.0 mmol, 2.10 equiv.) in DCM (5 mL) was added. The resulting solution was stirred at 0 °C for 10 min and at r.t. overnight. Then, hexane (25 mL) was added and stirring was continued at r.t. for 10 min. After filtration via short silica column (eluted with Et_2O), the solvent was removed under reduced pressure. The residue was purified via column chromatography (PE/EtOAc, 30:1 to 20:1) to afford target compound **1m** as a colorless solid (1.94 g, 8.48 mmol, 85%).

TLC R_f = 0.38 (PE/EtOAc, 10:1) [$KMnO_4$]. **Mp** 35.8 °C. **IR** [cm^{-1}]: 2982, 2937, 1730, 1469, 1409, 1372, 1256, 1178, 1103, 969, 902, 824, 731. **^{1}H NMR** (300 MHz, $CDCl_3$): δ 5.80 – 5.55 (m, 2H), 5.10 – 4.90 (m, 2H), 3.11 – 2.96 (m, 4H), 1.22 (d, *J* = 6.3 Hz, 12H). **^{13}C NMR** (75 MHz, $CDCl_3$): δ 171.2, 126.0, 68.0, 38.2, 21.8. **HRMS** (EI) calcd. for $[C_{12}H_{20}O_4]^{\cdot+}$ ($[M]^{\cdot+}$), m/z = 228.1356; found 228.1363.

Dibenzyl (*E*)-hex-3-enedioate (1n):

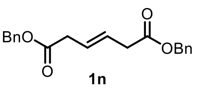

1-Ethyl-3-(3-dimethylaminopropyl)carbodiimide (5.75 g, 30.0 mmol, 3.00 equiv.), DMAP (122 mg, 1.00 mmol, 10 mol%) and BnOH (2.38 g, 22.0 mmol, 2.20 equiv.) were added to a solution of (*E*)-hex-3-enedioic acid (1.44 g, 10.0 mmol, 1.00 equiv.) in DCM (20 mL) at 0 °C. The resulting mixture was stirred at 0 °C for 10 min and at r.t. for 4 h. Then, DCM (50 mL) was added, and the solution was washed with 1% HCl (aq.) (50 mL) and brine. The layers were separated, and the organic phase was dried over Na_2SO_4 and filtered. The solvent was removed under reduced pressure and the residue was purified via column chromatography (PE/EtOAc, 30:1 to 15:1) to afford target compound **1n** as a colorless solid (2.72 g, 8.37 mmol, 83%).

TLC R_f = 0.29 (PE/EtOAc, 10:1) [UV]. **Mp** 40.8 °C. **IR** [cm^{-1}]: 3064, 3034, 2960, 1730, 1498, 1454, 1379, 1357, 1238, 1148, 1081, 969, 828, 738, 697. **^{1}H NMR** (300 MHz, $CDCl_3$): δ 7.43 – 7.28 (m, 10H), 5.81 – 5.64 (m, 2H), 5.12 (s, 4H), 3.18 – 3.11 (m, 4H). **^{13}C NMR** (75 MHz, $CDCl_3$): δ 171.4, 135.8, 128.6, 128.3, 126.0, 66.5, 37.9. **HRMS** (ESI) calcd. for $[C_{20}H_{21}O_4]^+$ ($[M+H]^+$), m/z = 325.1434; found 325.1430.

Dicyclohexyl (*E*)-hex-3-enedioate (1o):

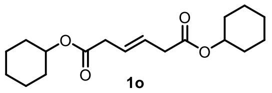
1o

(*E*)-hex-3-enedioic acid (1.44 g, 10.0 mmol, 1.00 equiv.), DMAP (122 mg, 1.00 mmol, 10 mol%) and cyclohexanol (2.20 g, 22.0 mmol, 2.20 equiv.) were dissolved in DCM (20 mL). At 0 °C, DCC (4.33 g, 21.0 mmol, 2.10 equiv.) in DCM (5 mL) was added. The resulting solution was stirred at 0 °C for 10 min and at r.t. overnight. Then, hexane (25 mL) was added and stirring was continued at r.t. for another 10 min. After filtration via short silica column (eluted with Et_2O), the solvent was removed under reduced pressure. The residue was purified via column chromatography (PE/EtOAc, 30:1 to 20:1) to afford target compound **1o** as a colorless solid (2.49 g, 8.09 mmol, 81%).

TLC R_f = 0.38 (PE/EtOAc, 10:1) [$KMnO_4$]. **Mp** 48.6 °C. **IR** [cm^{-1}]: 2937, 2859, 1722, 1446, 1405, 1364, 1271, 1193, 1144, 1036, 1014, 951, 917, 895, 801, 731. **^{1}H-NMR** (300 MHz, $CDCl_3$): δ 5.78 – 5.58 (m, 2H), 4.86 – 4.62 (m, 2H), 3.12 – 2.98 (m, 4H), 1.89 – 1.65 (m, 8H), 1.59 – 1.47 (m, 2H), 1.47 – 1.20 (m, 10H). **^{13}C-NMR** (75 MHz, $CDCl_3$): δ 171.1, 126.0, 72.9, 38.3, 31.6, 25.4, 23.7. **HRMS** (ESI) calcd. for $[C_{18}H_{29}O_4]^+$ ($[M+H]^+$), m/z = 309.2060; found 309.2064.

Diphenyl (*E*)-hex-3-enedioate (1p):

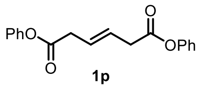
1p

(*E*)-hex-3-enedioic acid (1.44 g, 10.0 mmol, 1.00 equiv.), DMAP (122 mg, 1.00 mmol, 10 mol%) and cyclohexanol (2.20 g, 22.0 mmol, 2.20 equiv.) were dissolved in DCM (20 mL). At 0 °C, DCC (4.33 g, 21.0 mmol, 2.10 equiv.) in DCM (5 mL) was added. The resulting solution was stirred at 0 °C for 10 min and at r.t. overnight. Then, hexane (25 mL) was added and stirring was continued at r.t. for another 10 min. After filtration via short silica column (eluted with DCM), the solvent was removed under reduced pressure. The residue was purified via column chromatography (PE/EtOAc, 20:1 to 15:1, then DCM) to get the crude target compound. Further recrystallization with DCM/hexane afforded pure compound **1p** as a colorless solid (2.42 g, 8.17 mmol, 82%).

TLC R_f = 0.25 (PE/EtOAc, 10:1) [UV]. **Mp** 119.7 °C. **IR** [cm^{-1}]: 3068, 3038, 2892, 1737, 1588, 1484, 1416, 1379, 1279, 1200, 1133, 992, 931, 898, 813, 768, 719, 690. **^{1}H NMR** (300 MHz, $CDCl_3$): δ 7.44 – 7.33 (m, 4H), 7.28 – 7.20 (m, 2H), 7.17 – 7.05 (m, 4H), 6.02 – 5.84 (m, 2H), 3.48 – 3.32 (m, 4H). **^{13}C NMR** (75 MHz, $CDCl_3$): δ 169.9, 150.6, 129.5, 126.0, 121.5, 37.9. **HRMS** (EI) calcd. for $[C_{18}H_{16}O_4]^{\cdot+}$ ($[M]^{\cdot+}$), m/z = 296.1043; found 294.1050.

Diheptyl (*E*)-hex-3-enedioate (1q):

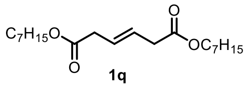
1q

1-Ethyl-3-(3-dimethylaminopropyl)carbodiimide (5.75 g, 30.0 mmol, 3.00 equiv.), DMAP (122 mg, 1.00 mmol, 10 mol%) and heptan-1-ol (2.56 g, 22.0 mmol, 2.20 equiv.) were added to a solution of (*E*)-hex-3-enedioic acid (1.44 g, 1.00 mmol, 1.00 equiv.) in DCM (20 mL) at 0 °C. The resulting mixture was stirred at 0 °C for 10 min then at r.t. for 4 h. Then, DCM (50 mL) was added, and the solution was washed with 1% HCl (aq.) (50 mL) and brine. The layers were separated, and the organic phase was dried over Na_2SO_4 and filtered. The solvent was removed under reduced pressure and the residue was purified via column chromatography (PE/EtOAc, 30:1 to 20:1) to get target compound **1q** as a colorless oil (2.51 g, 7.37 mmol, 74%).

TLC R_f = 0.31 (PE/EtOAc, 20:1) [$KMnO_4$]. **Mp** 342 °C. **IR** [cm^{-1}]: 2930, 2859, 1737, 1465, 1357, 1241, 1152, 1066, 1003, 969, 861, 723. **^{1}H NMR** (300 MHz, $CDCl_3$): δ5.77 – 5.60 (m, 2H), 4.06 (t, J = 6.7 Hz, 4H), 3.15 – 3.00 (m, 4H), 1.67 – 1.55

(m, 4H), 1.37 – 1.19 (m, 16H), 0.92 – 0.83 (m, 6H). **^{13}C NMR** (75 MHz, $CDCl_3$): δ 171.7, 126.0, 64.9, 37.9, 31.7, 28.9, 28.6, 25.8, 22.6, 14.1. **HRMS** (EI) calcd. for $[C_{20}H_{36}O_4]^{\cdot+}$ ($[M]^{\cdot+}$), m/z = 340.2608; found 340.2611.

1-((1R,2S,5R)-2-isopropyl-5-methylcyclohexyl) 6-((1S,2R,5S)-2-isopropyl-5-methylcyclohexyl) (*E*)-hex-3-enedioate (1r):

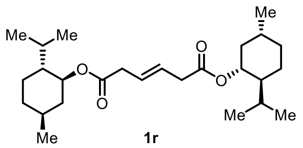
1r

(*E*)-hex-3-enedioic acid (1.44 g, 10.0 mmol, 1.00 equiv.), DMAP (122 mg, 1.00 mmol, 10 mol%) and L-menthol (2.20 g, 22.0 mmol, 2.20 equiv.) were dissolved in DCM (20 mL). At 0 °C, DCC (4.33 g, 21.0 mmol, 2.10 equiv.) in DCM (5 mL) was added. The resulting solution was stirred at 0 °C for 10 min and at r.t. overnight. Then, hexane (20 mL) was added and stirring was continued at r.t. for 10 min. After filtration via short silica column (eluted with Et_2O), the solvent was removed under reduced pressure. The residue was purified via column chromatography (PE/EtOAc, 60:1 to 50:1) to afford target compound **1r** as a colorless solid (2.50 g, 5.94 mmol, 59%).

TLC R_f = 0.64 (PE/EtOAc, 10:1) [$KMnO_4$]. **Mp** 43.6 °C. **IR** [cm^{-1}]: 2952, 2926, 2870, 1722, 1454, 1387, 1275, 1159, 1096, 1040, 984, 876, 842, 779, 731. **^{1}H NMR** (300 MHz, $CDCl_3$): δ 5.78 – 5.58 (m, 2H), 4.67 (td, *J* = 10.9, 4.5 Hz, 2H), 3.16 – 2.96 (m, 4H), 2.04 – 1.92 (m, 2H), 1.90 – 1.76 (m, 2H), 1.71 – 1.59 (m, 4H), 1.57 – 1.30 (m, 4H), 1.14 – 0.81 (m, 18H), 0.74 (d, *J* = 7.0 Hz, 6H). **^{13}C NMR** (75 MHz, $CDCl_3$): δ 171.2, 126.0, 74.5, 47.0, 40.9, 38.2, 34.2, 31.4, 26.3, 23.5, 22.0, 20.8, 16.4. **HRMS** (ESI) calcd. for $[C_{26}H_{45}O_4]^+$ ($[M+H]^+$), m/z = 421.3312; found 421.3304.

Cyclopropylmethyl (*E*)-hex-3-enoate (1u):

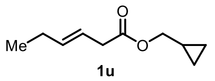
1u

(*E*)-hex-3-enoic acid (1.60 g, 14.0 mmol, 1.17 equiv.), DMAP (61.1 mg, 0.50 mmol, 4 mol%) and cyclopropylmethanol (865 mg, 12.0 mmol, 1.00 equiv.) were dissolved in DCM (20 mL). At 0 °C, DCC (2.48 g, 12.0 mmol, 1.00 equiv.) in DCM (5 mL) was added. The resulting solution was stirred at 0 °C for 10 min and at r.t. overnight. 1-methylpiperidin-4-ol (346 mg, 3.00 mmol, 0.25 equiv.) was added and stirring was continued at r.t. for 1 h. Then, hexane (30 mL) was added, and the mixture was stirred at r.t. for another 10 min. After filtration via short silica column (eluted with Et_2O), the solvent was removed under reduced pressure. The residue was purified via column chromatography (PE/EtOAc, 100:1 to 50:1) to afford target compound **1u** as a colorless oil (1.69 g, 10.1 mmol, 84%).

TLC R_f = 0.51 (PE/EtOAc, 20:1) [$KMnO_4$]. **IR** [cm^{-1}]: 3086, 3008, 2967, 1733, 1461, 1357, 1316, 1282, 1238, 1156, 1021, 969, 831, 805. **^{1}H NMR** (300 MHz, $CDCl_3$): δ 5.69 – 5.44 (m, 2H), 3.90 (d, *J* = 7.3 Hz, 2H), 3.10 – 2.99 (m, 2H), 2.12 – 1.97 (m, 2H), 1.20 – 1.04 (m, 1H), 0.98 (t, *J* = 7.5 Hz, 3H), 0.60 – 0.49 (m, 2H), 0.31 – 0.21 (m, 2H). **^{13}C NMR** (75 MHz, $CDCl_3$): δ 172.4, 136.3, 120.7, 69.3, 38.1, 25.5, 13.5, 9.8, 3.2. **HRMS** (EI) calcd. for $[C_{10}H_{16}O_2]^{\cdot+}$ ($[M]^{\cdot+}$), m/z = 168.1145; found 168.1141.

Oxiran-2-ylmethyl (*E*)-hex-3-enoate (1v):

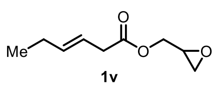
1v

(*E*)-hex-3-enoic acid (1.60 g, 14.0 mmol, 1.17 equiv.), DMAP (61.1 mg, 0.50 mmol, 4 mol%) and oxiran-2-ylmethanol (889 mg, 12.0 mmol, 1.00 equiv.) were dissolved in DCM (20 mL). At 0 °C, DCC (2.48 g, 12.0 mmol, 1.00 equiv.) in DCM (5 mL) was added. The resulting solution was stirred at 0 °C for 10 min and at r.t. overnight. 1-methylpiperidin-4-ol (346 mg, 3.00 mmol, 0.25 equiv.) was added and stirring was continued at r.t. for 1 h. Then, hexane (30 mL) was added and the mixture was stirred at r.t. for another 10 min. After filtration via short silica column (eluted with Et_2O), the solvent was removed under reduced pressure. The residue was purified via column chromatography (PE/EtOAc, 40:1 to 12:1) to afford target compound **1v** as a colorless oil (808 mg, 4.75 mmol, 40%).

TLC R_f = 0.46 (PE:EtOAc, 5:1) [$KMnO_4$]. **IR** [cm^{-1}]: 2967, 1737, 1457, 1361, 1316, 1238, 1156, 1081, 1014, 969, 910, 857, 764, 697. **^{1}H NMR** (300 MHz, $CDCl_3$): δ 5.70 – 5.42 (m, 2H), 4.42 (dd, *J* = 12.3, 3.1 Hz, 1H), 3.92 (dd, *J* = 12.2, 6.3 Hz, 1H), 3.27 – 3.15 (m, 1H), 3.10 – 3.02 (m,, 2H), 2.89 – 2.78 (m, 1H), 2.64 (dd, *J* = 4.8, 2.6 Hz, 1H), 2.12 – 1.96 (m, 2H), 0.98 (t, *J* = 7.5 Hz, 3H). **^{13}C NMR** (75 MHz, $CDCl_3$): δ 172.0, 136.7, 120.2, 65.0, 49.4, 44.7, 37.8, 25.5, 13.4. **HRMS** (EI) calcd. for $[C_9H_{14}O_3]^{\cdot+}$ ($[M]^{\cdot+}$), m/z = 170.0938; found 170.0936.

Pent-3-yn-1-yl (*E*)-hex-3-enoate (1w):

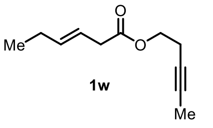

1w

(*E*)-hex-3-enoic acid (1.60 g, 14.0 mmol, 1.40 equiv.), DMAP (61.1 mg, 0.50 mmol, 5 mol%) and pent-3-yn-1-ol (841 mg, 10.0 mmol, 1.00 equiv.) was dissolved in DCM (20 mL). At 0 °C, DCC (2.48 g, 12.0 mmol, 1.20 equiv.) in DCM (5 mL) was added. The resulting solution was stirred at 0 °C for 10 min and at r.t. overnight. 1-methylpiperidin-4-ol (346 mg, 3.00 mmol, 0.30 equiv.) was added and stirring was continued at r.t. for 1 h. Then, hexane (30 mL) was added, and the mixture was stirred at r.t. for another 10 min. After filtration via short silica column (eluted with Et_2O), the solvent was removed under reduced pressure. The residue was purified via column chromatography (PE/EtOAc, 100:1 to 50:1) to afford target compound **1w** as a colorless oil (1.77 g, 9.81 mmol, 98%).

TLC R_f = 0.46 (PE/EtOAc, 5:1) [$KMnO_4$]. **IR** [cm^{-1}]: 2967, 2922, 1737, 1457, 1320, 1238, 1156, 1074, 1018, 969, 816. **^{1}H NMR** (300 MHz, $CDCl_3$): δ 5.68 – 5.43 (m, 2H), 4.13 (t, *J* = 7.0 Hz, 2H), 3.10 – 2.96 (m, 2H), 2.52 – 2.37 (m, 2H), 2.13 – 1.95 (m, 2H), 1.77 (t, *J* = 2.5 Hz, 3H), 0.98 (t, *J* = 7.4 Hz, 3H). **^{13}C NMR** (75 MHz, $CDCl_3$): δ 172.0, 136.4, 120.5, 77.3, 74.7, 62.9, 37.9, 25.5, 19.2, 13.4, 3.5. **HRMS** (EI) calcd. for $[C_{11}H_{16}O_2]^{\cdot+}$ ($[M]^{\cdot+}$), m/z = 180.1145; found 180.1145.

4-Oxocyclopent-2-en-1-yl (*E*)-hex-3-enoate (1x):

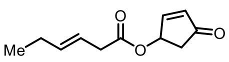

1x

(*E*)-hex-3-enoic acid (1.37 g, 12.0 mmol, 1.20 equiv.), DMAP (61.1 mg, 0.50 mmol, 5 mol%) and 4-hydroxycyclopent-2-en-1-one (981 mg, 10.0 mmol, 1.00 equiv.) was dissolved in DCM (20 mL). At 0 °C, DCC (2.48 g, 12.0 mmol, 1.20 equiv.) in DCM (5 mL) was added. The resulting solution was stirred at 0 °C for 10 min and at r.t. overnight. 1-methylpiperidin-4-ol (461 mg, 4.00 mmol, 0.40 equiv.) was added and stirring was continued at r.t. for 1 h. Then, hexane (30 mL) was added, and the mixture was stirred at r.t. for another 10 min. After filtration via short silica column (eluted with Et_2O), the solution was concentrated. The residue was purified via column chromatography (PE/EtOAc, 20:1) to afford target compound **1x** as a colorless oil (914 g, 4.71 mmol, 47%).

TLC R_f = 0.46 (PE/EtOAc, 5:1) [$KMnO_4$]. **IR** [cm^{-1}]: 2967, 2937, 1722, 1592, 1461, 1405, 1359, 1238, 1152, 1100, 1051, 1014, 969, 857, 828, 794. **^{1}H NMR** (300 MHz, $CDCl_3$): δ 7.56 (dd, *J* = 5.7, 2.4 Hz, 1H), 6.32 (dd, *J* = 5.7, 1.3 Hz, 1H), 5.92 – 5.78 (m, 1H), 5.68 – 5.54 (m, 1H), 5.53 – 5.41 (m, 1H), 3.04 (dd, *J* = 6.7, 1.1 Hz, 2H), 2.82 (dd, *J* = 18.7, 6.4 Hz, 1H), 2.31 (dd, *J* = 18.8, 2.2 Hz, 1H), 2.11 – 1.97 (m, 2H), 0.97 (t, *J* = 7.5 Hz, 3H). **^{13}C NMR** (75 MHz, $CDCl_3$): δ 204.9, 171.7, 159.0, 137.1, 119.8, 72.0, 41.0, 37.9, 25.5, 13.4. **HRMS** (EI) calcd. for $[C_{11}H_{14}O_3]^{\cdot+}$ ($[M]^{\cdot+}$), m/z = 194.0938; found 194.0943.

Cyclopent-3-en-1-yl (*E*)-hex-3-enoate (1y):

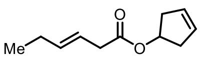

1y

(*E*)-hex-3-enoic acid (1.37 g, 12.0 mmol, 1.20 equiv.), DMAP (61.1 mg, 0.50 mmol, 5 mol%) and cyclopent-3-en-1-ol (841 mg, 10.0 mmol, 1.00 equiv.) was dissolved in DCM (20 mL). At 0 °C, DCC (2.48 g, 12.0 mmol, 1.20 equiv.) in DCM (5 mL) was added. The resulting solution was stirred at 0 °C for 10 min and at r.t. overnight. 1-methylpiperidin-4-ol (461 mg, 4.00 mmol, 0.40 equiv.) was added and stirring was continued at r.t. for 1 h. Then, hexane (30 mL) was added, and the mixture was stirred at r.t. for another 10 min. After filtration via short silica column (eluted with Et_2O), the solvent was removed under reduced pressure. The residue was purified via column chromatography (PE/EtOAc, 100:1 to 60:1) to afford target compound **1y** as a colorless oil (1.85 g, 10.3 mmol, >99%).

TLC R$_f$ = 0.53 (PE/EtOAc, 20:1) [$KMnO_4$]. **IR** [cm^{-1}]: 3064, 2967, 2937, 2848, 1733, 1618, 1461, 1431, 1353, 1320, 1256, 1163, 1118, 1025, 969, 880, 842, 671. **^{1}H NMR** (300 MHz, $CDCl_3$): δ 5.75 – 5.66 (m, 2H), 5.64 – 5.43 (m, 2H), 5.37 (tt, *J* = 7.0, 2.5 Hz, 1H), 3.03 – 2.91 (m, 2H), 2.81 – 2.65 (m, 2H), 2.44 – 2.31 (m, 2H), 2.10 – 1.96 (m, 2H), 0.97 (t, *J* = 7.5 Hz, 3H). **^{13}C NMR** (75 MHz, $CDCl_3$): δ 172.2, 136.3, 128.3, 120.6, 74.3, 39.7, 38.3, 25.5, 13.5. **HRMS** (EI) calcd. for $[C_{11}H_{16}O_2]^{\cdot+}$ ($[M]^{\cdot+}$), m/z = 180.1145; found 180.1147.

Ethyl (*E*)-2-ethylhex-3-enoate (1z)[6]:

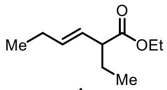

1z

LDA (2.0 M in THF, 11.0 mL, 22.0 mmol, 1.10 equiv.) was dissolved in dry THF (30 mL) under a N_2 atmosphere. At -78 °C, hexamethylphosphoric triamide (3.94 g, 3.83 mL, 22.0 mmol, 1.10 equiv.) was added. The resulting mixture was stirred at -78 °C for 10 min. Then, ethyl (*E*)-hex-3-enoate (2.84 g, 3.20 mL, 20.0 mmol, 1.00 equiv.) in dry THF (5 mL) was added dropwise and the solution reacted at -78 °C for 30 min. After that, iodoethane (15.6 g, 8.00 mL, 100 mmol, 5.00 equiv.) was added quickly. Stirring was continued at -78 °C for 20 min and at r.t. overnight. H_2O (50 mL) was added to quench the reaction, before THF was removed under reduced pressure (35 °C, 200 mbar). Et_2O (100 mL) and 1 M HCl (aq., 50 mL) were added, the layers were seperated and the aqueous phase was extracted with Et_2O (100 mL). The combined organic phases were washed with 1.0 M HCl (3 x 50 mL), dried over Na_2SO_4, filtered and removed under reduced pressure (35 °C, 200 mbar). The residue was purified via column chromatography (PE/EtOAc, 100:1 to 60:1) to afford target compound **1z** as a colorless oil (2.87 g, 16.9 mmol, 84%).

TLC R$_f$ = 0.39 (PE/EtOAc, 20:1) [$KMnO_4$]. **IR** [cm^{-1}]: 2967, 2878, 1733, 1461, 1372, 1301, 1260, 1223, 1170, 1074, 1029, 969, 861, 835, 779. **^{1}H NMR** (300 MHz, $CDCl_3$): δ 5.57 (dt, *J* = 15.5, 6.2 Hz, 1H), 5.39 (ddt, *J* = 15.4, 8.6, 1.4 Hz, 1H), 4.12 (q, *J* = 7.1 Hz, 2H), 2.83 (dd, *J* = 15.6, 7.4 Hz, 1H), 2.08 – 1.96 (m, 2H), 1.82 – 1.68 (m, 1H), 1.60 – 1.45 (m, 1H), 1.24 (t, *J* = 7.1 Hz, 3H), 0.97 (t, *J* = 7.5 Hz, 3H), 0.87 (t, *J* = 7.4 Hz, 3H). **^{13}C NMR** (75 MHz, $CDCl_3$): δ 174.8, 134.9, 126.6, 60.3, 51.0, 25.9, 25.5, 14.2, 13.6, 11.6. **HRMS** (EI) calcd. for $[C_{10}H_{18}O_2]^{\cdot+}$ ($[M]^{\cdot+}$), m/z = 170.1301; found 170.1298.

Butyl 2-methylbut-3-enoate (1aa):

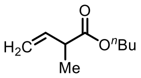

1aa

2-methylbut-3-enoic acid (2.00 g, 20.0 mmol, 1.00 equiv.), DMAP (122 mg, 1.00 mmol, 5 mol%) and butan-1-ol (1.63 g, 22.0 mmol, 1.10 equiv.) were dissolved in DCM (20 mL). At 0 °C, DCC (4.54 g, 22.0 mmol, 1.10 equiv.) in DCM (10 mL) was added. The resulting solution was stirred at 0 °C for 10 min and at r.t. overnight. Tosylphenylalanine (1.28 g, 4.00 mmol, 0.20 equiv.) was added and stirring was continued at r.t. for 1 h. Then, hexane (30 mL) was added, and the mixture was stirred at r.t. for another 10 min. After filtration via short silica column (eluted with Et_2O), the solvent was removed under reduced pressure. The residue was purified via column chromatography (PE/EtOAc, 100:1 to 60:1) to afford target compound **1aa** as a colorless oil (2.25 g, 14.4 mmol, 72%).

TLC R$_f$ = 0.46 (PE/EtOAc, 20:1) [$KMnO_4$]. **IR** [cm^{-1}]: 2963, 2878, 1733, 1640, 1461, 1379, 1327, 1245, 1178, 1077, 992, 917, 842, 779, 738. **^{1}H NMR** (300 MHz, $CDCl_3$): δ 5.92 (ddd, *J* = 17.4, 10.2, 7.4 Hz, 1H), 5.18 – 5.04 (m, 2H), 4.08 (t, *J* = 6.7 Hz, 2H), 3.24 – 3.03 (m, 1H), 1.67 – 1.55 (m, 2H), 1.44 – 1.30 (m, 2H), 1.26 (d, *J* = 7.0 Hz, 3H), 0.92 (t, *J* = 7.3 Hz, 3H). **^{13}C NMR** (75 MHz, $CDCl_3$): δ 174.6, 137.3, 115.8, 64.5, 43.8, 30.7, 19.1, 16.7, 13.7. **HRMS** (EI) calcd. for $[C_9H_{16}O_2]^{\cdot+}$ ($[M]^{\cdot+}$), m/z = 156.1145; found 156.1141.

Diethyl (*E*)-2-(hex-1-en-1-yl)malonate (1ab)[7]:

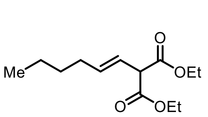

1ab

Step 1: To a DEAN-STARK apparatus, diethyl malonate (8.00 g, 50.0 mmol, 1.00 equiv.), piperidine (639 mg, 0.74 mL, 7.50 mmol, 15 mol%), AcOH (901 mg, 0.86 mL, 15.0 mmol, 0.30 equiv.) and toluene (30 mL) were added. The resulting solution was stirred at 160 °C for 10 min. Then, hexanal (6.00 g, 60.0 mmol, 1.20 equiv.) in 10 mL toluene was added slowly over a period of 30 min and stirring was

continued at 160 °C for another 16 h. After the mixture was cooled down, the solvent was removed under reduced pressure. H_2O (50 mL) and EtOAc (100 mL) were added. The layers were separated, and the aqueous phase was extracted with EtOAc (3 x 100 mL). The combined organic phases were washed with brine, dried over Na_2SO_4, and filtered. The solvent was removed under reduced pressure and the residue was purified via column chromatography (PE/EtOAc, 60:1) to afford diethyl 2-hexylidenemalonate as a colorless oil (2.73 g, 11.3 mmol, 23%).

Step 2: Diethyl 2-hexylidenemalonate (1.21 g, 5.00 mmol, 1.00 equiv., from step 1) was further dissolved in dry THF (10 mL) and added to LiO^{t}Bu (2.2 M in THF, 6.00 mL, 13.2 mmol, 2.64 equiv.). The mixture was stirred at r.t. for 12 h., before saturated NH_4Cl (aq., 20 mL) was added to quench the reaction. THF was removed under reduced pressure (35 °C, 200 mbar). EtOAc (100 mL) was added, the phases were separated, and the aqueous phase was then again extracted with EtOAc (50 mL). The combined organic phases were dried over Na_2SO_4 and filtered. The solvent was removed under reduced pressure and the residue was purified via column chromatography (PE/EtOAc, 60:1 to 40:1) to afford target compound **1ab** as a colorless oil (1.03 g, 4.27 mmol, 85% in the second step).

TLC R_f = 0.50 (PE/EtOAc, 10:1) [$KMnO_4$]. **IR** [cm^{-1}]: 2960, 2930, 2874, 1733, 1465, 1368, 1297, 1267, 1208, 1174, 1148, 1029, 969, 865, 783, 738, 697. **^{1}H NMR** (300 MHz, $CDCl_3$): δ 5.70 – 5.54 (m, 2H), 4.14 (q, *J* = 7.1 Hz, 4H), 3.98 – 3.84 (m, 1H), 2.09 – 1.94 (m, 2H), 1.35 – 1.18 (m, 10H), 0.83 (t, *J* = 7.1 Hz, 3H). **^{13}C NMR** (75 MHz, $CDCl_3$): δ 168.5, 137.0, 121.3, 61.5, 55.8, 32.1, 31.0, 22.1, 14.0, 13.9. **HRMS** (ESI) calcd. for $[C_{13}H_{23}O_4]^+$ ($[M+H]^+$), m/z = 243.1591; found 243.1596.

Butyl 4-methylpent-3-enoate (1ac):

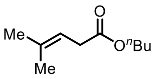

1ac

Step 1: (*E*)-4-methylpent-2-enoic acid (1.92 g, 2.00 mL, 16.8 mmol, 1.00 equiv.) was dissolved in 6 M KOH (aq., 40 mL) under N_2 atomsphere and the resulting solution was stirred at 105 °C for 48 h. After the temperature was cooled down, the reaction mixture was acidified to pH = 1 with 2 M HCl (aq.) and further extracted with DCM (3 x 50 mL). The combined organic phases were dried over Na_2SO_4 and filtered. The solvent was removed under reuced pressure to afford 4-methylpent-3-enoic acid[8] (1.67 g, 14.6 mmol), which was used in the next step without further purifitcation.

Step 2: 4-methylpent-3-enoic acid (1.67 g, 14.6 mmol, 1.00 equiv., from step 1), DMAP (89.3 mg, 0.73 mmol, 5 mol%) and butan-1-ol (1.19 g, 16.1 mmol, 1.10 equiv.) were dissolved in DCM (20 mL). At 0 °C, DCC (3.32 g, 16.1 mmol, 1.10 equiv.) in DCM (10 mL) was added. The resulting solution was stirred at 0 °C for 10 min and at r.t. overnight. Tosylphenylalanine (933 mg, 2.92 mmol, 0.20 equiv.) was added and stirring was continued at r.t. for 1 h. Then, hexane (30 mL) was added, and the mixture was stirred at r.t. for another 10 min. After filtration via short silica column (eluted with Et_2O), the solvent was removed under reduced pressure. The residue was purified via column chromatography (PE/EtOAc, 100:1 to 60:1) to afford target compound **1ac** as a colorless oil (2.34 g, 13.7 mmol, 82% over two steps, containing 10% butyl (*E*)-4-methylpent-2-enoate).

TLC R_f = 0.40 (PE/EtOAc, 20:1) [$KMnO_4$]. **IR** [cm^{-1}]: 2960, 2933, 2874, 1737, 1454, 1379, 1312, 1256, 1159, 1062, 1029, 969, 835, 790, 738. **^{1}H NMR** (300 MHz, $CDCl_3$): δ 5.36 – 5.23 (m, 1H), 4.07 (td, *J* = 6.7, 1.3 Hz, 2H), 3.01 (d, *J* = 6.6 Hz, 2H), 1.73 (s, 3H), 1.68 – 1.53 (m, 5H), 1.44 – 1.29 (m, 2H), 0.92 (td, *J* = 7.3, 1.2 Hz, 3H). **^{13}C NMR** (75 MHz, $CDCl_3$): δ 172.6, 135.4, 116.0, 64.4, 33.9, 30.7, 25.7, 19.1, 18.0, 13.7. **HRMS** (EI) calcd. for $[C_{10}H_{18}O_2]^{\cdot+}$ ($[M]^{\cdot+}$), m/z = 170.1301; found 170.1302.

***tert*-Butyl((3,7-dimethyloct-6-en-1-yl)oxy)dimethylsilane (1ao):**

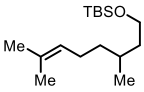

1ao

3,7-Dimethyloct-6-en-1-ol (15.6 g, 100 mmol, 1.00 equiv.) and 1H-imidazole (18.1 g, 120.0 mmol, 1.20 equiv.) were dissolved in DCM (200.0 mL). At 0 °C, TBSCl was added portionwise and the resulting solution was stirred at r.t. for 3 h. Then, saturated NH_4Cl (aq., 100 mL) was added, the layers were separated, and the aqueous phase was washed with DCM (3 x 50 mL). The combined organic phases were dried over Na_2SO_4, filtered and the solvent was removed under reduced pressure. The residue was purified via column chromatography (PE/EtOAc, 200:1 to 100:1) to afford target compound **1ao** as a colorless oil (17.9 g, 66.1 mmol, 66%).

TLC R_f = 0.23 (PE/EtOAc, 200:1) [$KMnO_4$]. **IR** [cm^{-1}]: 2956, 2859, 1741, 1461, 1379, 1252, 1096, 1006, 939, 898, 835, 775, 734, 664. **^{1}H-NMR** (300 MHz, $CDCl_3$): δ 5.18 – 4.99 (m, 1H), 3.74 – 3.55 (m, 2H), 2.08 – 1.85 (m, 2H), 1.68 (d, *J* = 1.0 Hz, 3H), 1.62 – 1.46 (m, 5H), 1.40 – 1.26 (m, 2H), 1.22 – 1.07 (m, 1H), 0.91 – 0.86 (m, 12H), 0.05 (s, 6H). **^{13}C-NMR** (75 MHz, $CDCl_3$): δ 131.1, 124.9, 61.5, 40.0, 37.2, 29.1, 26.0, 25.7, 25.5, 19.6, 18.4, 17.7, -5.2. **HRMS** (ESI) calcd. for $[C_{16}H_{35}OSi]^+$ ($[M+H]^+$), m/z = 271.2452; found 271.2451.

(8R,9S,10R,13S,14S,17S)-10,13-dimethyl-3-oxo-2,3,6,7,8,9,10,11,12,13,14,15,16,17-tetradecahydro-1H-cyclopenta[a]phenanthren-17-yl (*E*)-hex-3-enoate (1ap):

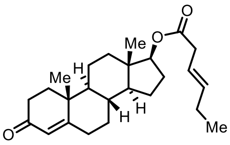

1ap

(*E*)-hex-3-enoic acid (1.44 g, 5.00 mmol, 1.00 equiv.), DMAP (30.5 mg, 0.25 mmol, 5 mol%) and Testosteron (720.7 mg, 5.00 mmol, 1.00 equiv.) were dissolved in DCM (20 mL). At 0 °C, DCC (1.08 g, 5.25 mmol, 1.05 equiv.) in DCM (5 mL) was added. The resulting solution was stirred at 0 °C for 10 min and at r.t. for 15 h. TLC-analysis showed residue of Testosteron still present in the reaction mixture. As a result, further DCC (102 mg, 0.49 mmol, 0.1 equiv.) in DCM (5 mL) was added at r.t. and the mixture was stirred for another 1 h. Then, hexane (30 mL) was added and stirring was continued at r.t. for 10 min. After filtration via short silica column (eluted with Et_2O), the solvent was removed under reduced pressure. The residue was purified via column chromatography (PE/EtOAc, 20:1 to 6:1) to afford target compound **1ap** as a colorless solid (1.91 g, 4.96 mmol, 99%).

TLC R_f = 0.58 (PE/EtOAc, 2:1) [UV, $KMnO_4$]. **Mp** 60.3 °C. **IR** [cm^{-1}]: 2971, 2937, 2907, 2848, 1730, 1662, 1614, 1450, 1401, 1293, 1230, 1174, 1036, 962, 865, 816, 678. **^{1}H NMR** (300 MHz, $CDCl_3$): δ 5.67 (d, *J* = 1.6 Hz, 1H), 5.61 – 5.37 (m, 2H), 4.56 (dd, *J* = 9.2, 7.8 Hz, 1H), 3.00 – 2.88 (m, 2H), 2.45 – 1.91 (m, 8H), 1.85 – 0.99 (m, 16H), 0.96 – 0.90 (m, 3H), 0.78 (s, 3H). **^{13}C NMR** (75 MHz, $CDCl_3$): δ 199.5, 172.3, 171.0, 136.2, 124.0, 120.8, 82.5, 53.7, 50.2, 42.6, 38.6, 38.3, 36.6, 35.7, 35.4, 34.0, 32.8, 31.5, 27.5, 25.5, 23.5, 20.5, 17.4, 13.5, 12.0. **HRMS** (ESI) calcd. for $[C_{25}H_{37}O_3]^+$ ($[M+H]^+$), m/z = 385.2737; found 385.2744.

Optimization of the Photo-Aerobic Intermolecular *N*-allylation reaction

General operation for the optimization:

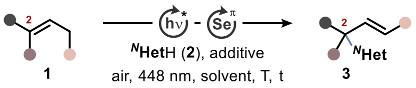

The stated alkene (**1**), *N*-nucleophile (**2**), photoredox catalyst, Se-catalyst, additive and solvent were added. The reaction mixture was vigorously stirred at the given temperature under irradiation at 448 nm and open to air for the given time. After reaction, the solvent was removed under reduced pressure. ^{1}H NMR yield was measured with internal standard 1,4-dimethoxybenzene and the isolated yield was obtained via further purification by column chromatography.

Table S1. Reaction optimization of the model reaction.

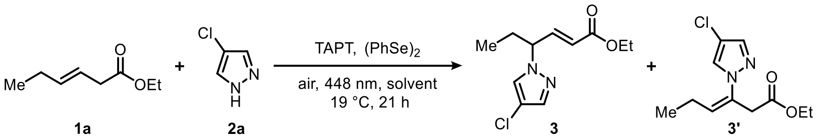

entry	1a	2a	TAPT	$(PhSe)_2$	solvent	t	T	^{1}H-NMR yield (total)	ratio (3:3')
**1**	3.0 equiv.	0.30 mmol	5 mol%	10 mol%	DCE	8 h	19 °C	76%	6:1
**2**	1.0 equiv.	0.30 mmol	5 mol%	10 mol%	DCE	8 h	19 °C	60%	5:1
**3**	0.30 mmol	3.0 equiv.	5 mol%	10 mol%	DCE	8 h	19 °C	71%	7:1
**4**	3.0 equiv.	0.30 mmol	5 mol%	10 mol%	CH_3CN	8 h	19 °C	24%	5:1
**5**	3.0 equiv.	0.30 mmol	5 mol%	10 mol%	$CHCl_3$	8 h	19 °C	76%	5:1
**6**	3.0 equiv.	0.30 mmol	5 mol%	10 mol%	Acetone	8 h	19 °C	21%	6:1
**7**	3.0 equiv.	0.30 mmol	5 mol%	10 mol%	Toluene	8 h	19 °C	5%	4:1
**8**[a]	3.0 equiv.	0.30 mmol	5 mol%	10 mol%	DCE	8 h	19 °C	76%	6:1
**9**[b]	3.0 equiv.	0.30 mmol	5 mol%	10 mol%	DCE	8 h	19 °C	69%	6:1
**10**	3.0 equiv.	0.30 mmol	10 mol%	10 mol%	DCE	8 h	19 °C	75%	6:1
**11**	3.0 equiv.	0.30 mmol	5 mol%	20 mol%	DCE	8 h	19 °C	73%	6:1
**12**	3.0 equiv.	0.30 mmol	10 mol%	20 mol%	DCE	8 h	19 °C	75%	7:1
**13**	3.0 equiv.	0.30 mmol	5 mol%	10 mol%	DCE	21 h	19 °C	83%	5:1
**14**[c]	3.0 equiv.	0.30 mmol	5 mol%	10 mol%	DCE	21 h	19 °C	76%	6:1
**15**[d]	3.0 equiv.	0.30 mmol	5 mol%	10 mol%	DCE	21 h	19 °C	78%	7:1
**16**	3.0 equiv.	0.30 mmol	5 mol%	10 mol%	DCE	6 h	19 °C	74%	6:1
**17**	3.0 equiv.	0.30 mmol	5 mol%	10 mol%	DCE	6 h	55 °C	67%	7:1
**18**	3.0 equiv.	0.30 mmol	--	10 mol%	DCE	8 h	19 °C	2%	0:1
**19**	3.0 equiv.	0.30 mmol	5 mol%	--	DCE	8 h	19 °C	0%	--
**20**[e]	3.0 equiv.	0.30 mmol	5 mol%	10 mol%	DCE	8 h	19 °C	0%	--
**21**[f]	3.0 equiv.	0.30 mmol	5 mol%	10 mol%	DCE	8 h	19 °C	0%	--

Conditions: **1a**, **2a**, 2,4,6-tris(4-methoxyphenyl)pyrylium tetrafluoroborate (TAPT), $(PhSe)_2$, solvent (4 mL), open to air, blue light irradiation. ^{1}H NMR total yield and the ratio of the two isomers (**3**:**3'**) were determined with 1,4-dimethoxybenzene as the internal standard. [a]DCE (2 mL) was used. [b]DCE (8 mL) was used. [c]Addition of second 5 mol% TAPT after 8 h. [d]Addition of second TAPT (5 mol%) and $(PhSe)_2$ (10 mol%) after 8 h. [e]N_2 atmosphere. [f]No light.

Scheme S1. Reaction optimization of model reaction with different additives.

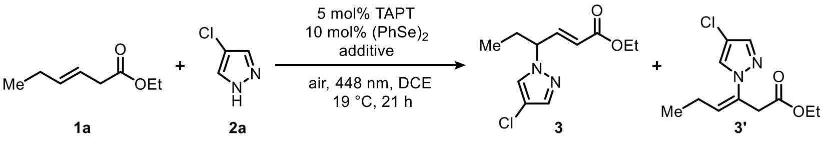

--	3 Å MS	Me_6Si_2	TEMPO	NaF	Li_2CO_3	KPF_6	CaF_2	Na_2HPO_4
6 h, 74%, 6:1;<br>8 h, 76%, 6:1<br>21 h, 83%, 5:1	8 h, 57%, 13:1[a]	21 h, 8%, 1:0[b]	21 h, 0%, --[c]	6 h, 59%, 4:1[b]	6 h, 57%, 4:1[b]	6 h, 56%, 5:1[b]	6 h, 75%, 6:1[b]<br>6 h, 76%, 5:1[d]	6 h, 30%, 4:1[b]
TFA	TfOH	$AlCl_3$	$Zn(OTf)_2$	$Yb(OTf)_3$	$(4\text{-ClPhS})_2$	$Sc(OTf)_3$		
8 h, 37%, 6:1[b]	8 h, 0%, --[b]<br>8 h, 0%, --[b,e]	8 h, 36%, 5:1[f]	8 h, 73%, 6:1[g]	21 h, 80%, 6:1[g]	21 h, 74%, 13:1[h]	8 h, 61%, 60:1[f]<br>8 h, 78%, 6:1[g]	21 h, 60%, 1:0[f]<br>21 h, 79%, 7:1[g]<br>21 h, 66%, 1:0[f,i]	
With 5 mol% $(4\text{-ClPhS})_2$								
$Sc(OTf)_3$	2-Nitrobenzaldehyde	$CaSO_4$	NaBArF	$Yb(OTf)_3$	CH_3COOH	HFIP	CH_3OH	2,6-diMePy
8 h, 58%, 1:0[f]	8 h, 72%, 7:1[j]<br>21 h, 68%, 7:1[j]; 77%, 6:1[f]	21 h, 75%, 8:1[k]	21 h, 72%, 8:1[l]	21 h, 83%, 6:1[g]	21 h, 74%, 11:1[b]	21 h, 74%, 10:1[b]	21 h, 67%, 13:1[b]	21 h, 2%, --[k]

Conditions: **1a** (3.0 equiv.), **2a** (0.30 mmol, 1.0 equiv.), TAPT (5 mol%), $(PhSe)_2$ (10 mol%), additive, DCE (4 mL), open to air, blue light irradiation, 21 h. ^{1}H NMR total yield and the ratio of the two isomers (**3**:**3'**) were determined with 1,4-dimethoxybenzene as the internal standard. [a]9.14 mg of additive. [b]1.00 equiv. of additive. [c]20 mol% of additive. [d]4.0 equiv. of additive. [e]10 mol% of $AlCl_3$ added. [f]10 mol% of additive. [g]2.5 mol% of additive. [h]5 mol% of additive. [i]4 Å MS (9.00 mg) added. [j]25 mol% of additive. [k]1.22 equiv. of additive. [l]4 mol% of additive. MS = molecular sieve. TEMPO = 2,2,6,6-Tetramethylpiperidinyloxyl. NaBArF = Sodium tetrakis[3,5-bis(trifluoromethyl)phenyl]borate.

Scheme S2. Reaction optimization of model reaction with different selenium catalysts.

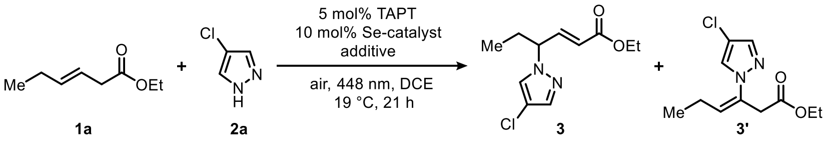

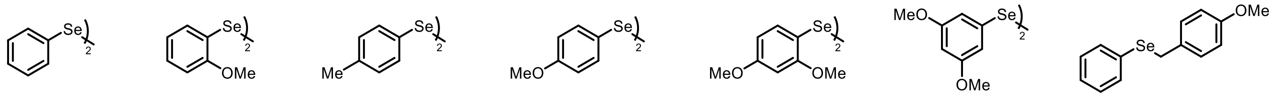

83%, 5:1	85%, 3:1	79%, 5:1	79%, 5:1	72%, 6:1	74%, 5:1	57%, 9:1[a]
With 5 mol% $(4\text{-ClPhS})_2$						
74%, 13:1	80%, 7:1	76%, 7:1	74%, 10:1	72%, 8:1	68%, 11:1	--

Conditions: **1a** (3.0 equiv.), **2a** (0.30 mmol, 1.0 equiv.), TAPT (5 mol%), Se-catalyst (10 mol%), additive, DCE (4 mL), open to air, blue light irradiation, 21 h. ^{1}H NMR total yield and the ratio of the two isomers (**3**:**3'**) were determined with 1,4-dimethoxybenzene as the internal standard. [a]20 mol% selenium catalyst was used.

Scheme S3. Reaction optimization of model reaction with different photocatalysts.

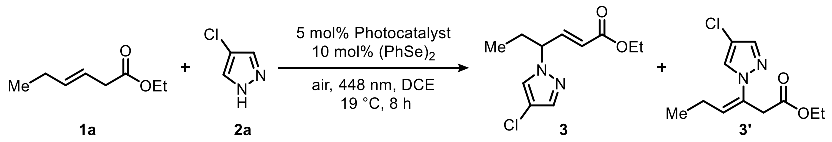

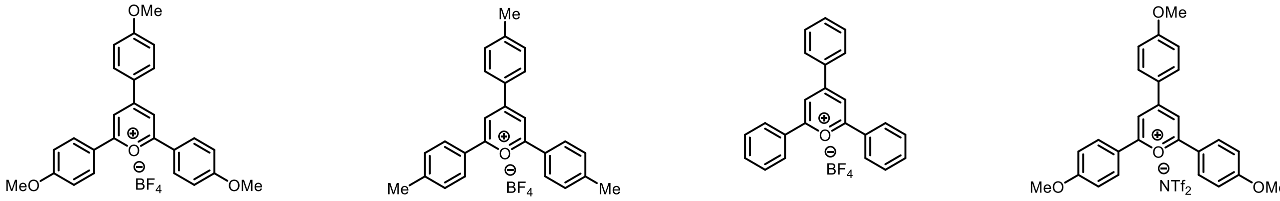

TAPT	(Tol)$_3$Pyr	(Ph)$_3$Pyr	TAP(NTf$_2$)
76%, 6:1	35%, 35:1	13%, 6:1	in 4 mL THF[a]: 12%, 5:1<br>in 4 mL TMO[a]: 19%, 3:1

Conditions: **1a** (3.0 equiv.), **2a** (0.30 mmol, 1.0 equiv.), TAPT (5 mol%), $(PhSe)_2$ (10 mol%), DCE (4 mL), open to air, blue light, 8 h. ^{1}H NMR total yield and the ratio of the two isomers (**3**:**3'**) were determined with 1,4-dimethoxybenzene as the internal standard. TMO = Trimethyl orthoformate. [a]21 h.

Table S2. Scale-up of the model reaction.

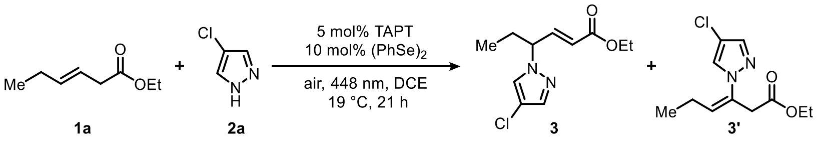

entry	1a	2a	concentration	^{1}H-NMR yield 3	^{1}H-NMR yield 3'	^{1}H-NMR yield (total)	ratio (3:3')
**1**[a]	3.0 equiv.	0.30 mmol	0.075 M	68%	15%	83%	5:1
**2**[a]	3.0 equiv.	1.0 mmol	0.075 M	16%	0%	16%	1:0
**3**[a]	3.0 equiv.	1.0 mmol	0.2 M	31%	3%	34%	10:1
**4**[a]	3.0 equiv.	0.50 mmol	0.1 M	60%	9%	69%	7:1
**5**[b]	3.0 equiv.	0.50 mmol	0.075 M	67%	15%	82%	4:1
**6**[b]	3.0 equiv.	1.0 mmol	0.075 M	62%	10%	72%	6:1
**7**[b]	3.0 equiv.	1.0 mmol	0.2 M	68% (68%)	15% (13%)	83% (81%)	5:1 (>49:1)[d]
**8**[b,c]	3.0 equiv.	1.0 mmol	0.2 M	72%	6%	78%	13:1
**9**[b]	1.0 mmol	3.0 equiv.	0.2 M	68% (68%)	5% (3%)	73% (71%)	14:1 (>49:1)[d]
**10**[b,c]	1.0 mmol	3.0 equiv.	0.2 M	63%	2.5%	66%	25:1

Conditions: **1a**, **2a**, TAPT (5 mol%), $(PhSe)_2$ (10 mol%), DCE, open to air, blue light irradiation, 21 h. Isolated yield in parentheses. ^{1}H NMR yield and the ratio of the two isomers (**1**:**1'**) were determined with 1,4-dimethoxybenzene as the internal standard. [a]40 mL vial. [b]100 mL round bottom flask. [c]$(4\text{-ClPhS})_2$ (5 mol%) was added. [d]The ratio referrs to the purity of the isolated allylic product.

Kinetic study of the model reaction

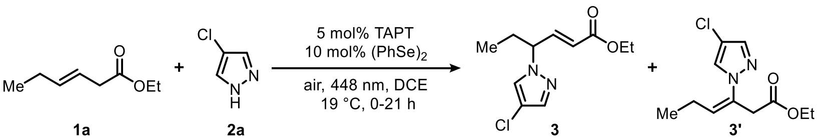

Ethyl (*E*)-hex-3-enoate **1a** (3.0 equiv.), 4-chloro-1H-pyrazole **2a** (0.30 mmol, 1.0 equiv.), TAPT (5 mol%), $(PhSe)_2$ (10 mol%) and DCE (4 mL) were added into a 40 mL vial. The reaction mixture was vigorously stirred at 19 °C under irradiation at 448 nm and open to air. For the given time, the reaction was stopped, and the solvent was removed under reduced pressure. ^{1}H NMR total yield and the ratio of the two isomers (**3**:**3'**) were determined with 1,4-dimethoxybenzene as the internal standard. The average of two reactions for each entry is given as follow:

entry	time	^{1}H-NMR yield 3	^{1}H-NMR yield 3'	sum	Ratio (3:3')
**1**	2 h	25%	4%	29%	6:1
**2**	4 h	57%	8%	65%	7:1
**3**	6 h	63%	11%	74%	6:1
**4**	8 h	65%	11%	76%	6:1
**5**	16 h	68%	11%	79%	6:1
**6**	21 h	68%	15%	83%	5:1

Figure S1. Kinetic study of the model reaction.

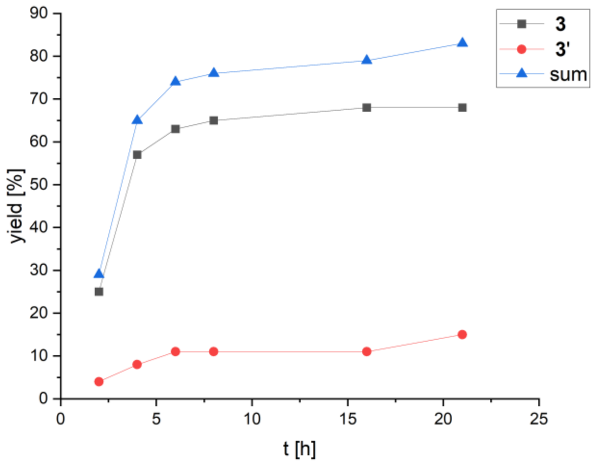

Table 3. Stoichiometry, solvent and concentration optimization in the reaction between (*E*)-dec-5-ene (**1al**) and *N*,4-dimethylbenzenesulfonamide (**2t**)

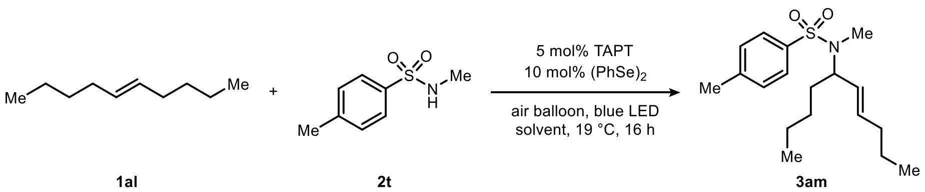

entry	1al	2t	solvent	^{1}H-NMR yield	entry	1al	2t	solvent	^{1}H-NMR yield
**1**[a]	0.50 mmol	2.0 equiv.	o-xylene (0.1 M)	4%	**19**	0.50 mmol	5.0 equiv.	EtOAc (0.05 M)	7%
**2**[a]	0.50 mmol	5.0 equiv.	o-xylene (0.1 M)	6%	**20**	0.50 mmol	5.0 equiv.	EtOAc (0.20 M)	13%
**3**[a]	0.50 mmol	10. equiv.	o-xylene (0.1 M)	2%	**21**	0.50 mmol	5.0 equiv.	EtOAc (0.50 M)	10%
**4**	0.50 mmol	5.0 equiv.	o-xylene (0.1 M)	3%	**22**	0.50 mmol	5.0 equiv.	PhCl (0.025 M)	10%
**5**[b]	0.50 mmol	5.0 equiv.	THF (0.1 M)	18%	**23**[b]	0.50 mmol	5.0 equiv.	PhCl (0.05 M)	19%
**6**	0.50 mmol	5.0 equiv.	CH_3CN (0.1 M)	6%	**24**	0.50 mmol	5.0 equiv.	PhCl (0.20 M)	13%
**7**	0.50 mmol	5.0 equiv.	DCM (0.1 M)	14%	**25**	0.50 mmol	5.0 equiv.	PhCl (0.50 M)	3%
**8**	0.50 mmol	5.0 equiv.	Acetone (0.1 M)	15%	**26**	0.50 mmol	5.0 equiv.	Toluene (0.05 M)	5%
**9**	0.50 mmol	5.0 equiv.	$C_2H_4Cl_2$ (0.1 M)	15%	**27**	0.50 mmol	5.0 equiv.	Toluene (0.20 M)	0%
**10**	0.50 mmol	5.0 equiv.	HFIP (0.1 M)	7%	**28**	0.50 mmol	5.0 equiv.	Toluene (0.50 M)	0%
**11**	0.50 mmol	5.0 equiv.	DMSO (0.1 M)	0%	**29**	0.50 mmol	5.0 equiv.	THF (0.05 M)	14%
**12**	0.50 mmol	5.0 equiv.	MeOH (0.1 M)	0%	**30**	0.50 mmol	5.0 equiv.	THF (0.20 M)	12%
**13**	0.50 mmol	5.0 equiv.	$CHCl_3$ (0.1 M)	13%	**31**	0.50 mmol	5.0 equiv.	THF (0.50 M)	10%
**14**	0.50 mmol	5.0 equiv.	1,4-dioxane (0.1 M)	18%	**32**[c]	5.0 equiv.	0.10 mmol	EtOAc (0.02 M)	8%
**15**[b]	0.50 mmol	5.0 equiv.	PhCl (0.1 M)	19%	**33**[b,c]	5.0 equiv.	0.10 mmol	PhCl (0.02 M)	37%
**16**[b]	0.50 mmol	5.0 equiv.	EtOAc (0.1 M)	21%	**34**[c]	5.0 equiv.	0.10 mmol	Toluene (0.02 M)	0%
**17**[b]	0.50 mmol	5.0 equiv.	Toluene (0.1 M)	7%	**35**[b,c]	5.0 equiv.	0.50 mmol	PhCl (0.10 M)	12%
**18**	0.50 mmol	5.0 equiv.	EtOH (0.1 M)	0%			-		

Conditions: **1al**, **2t**, TAPT (5 mol%), $(PhSe)_2$ (10 mol%), solvent, air balloon, irradiation with blue LEDs (λ_{max} = 448 or 440-460 nm, 472 or 425 mW respectively), 16 h. [a]TAPT (10 mol%) and $(PhSe)_2$ (5 mol%) were used. ^{1}H NMR yield of **56** was measured with 1,1,2-trichloroethylene as the internal standard. [b]Average of two reactions. [c]TAPT (25 mol%) and $(PhSe)_2$ (50 mol%) were used.

Table S4. Variation of catalysts and redox mediator.

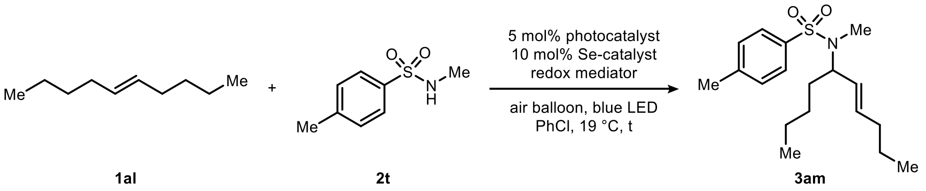

entry	photocatalyst	Se-catalyst	redox mediator	time	^{1}H-NMR yield
**1**	TAPT	$(PhSe)_2$	--	8 h	21%
**2**	Acridine	$(PhSe)_2$	--	8 h	0%
**3**	TAPT	(2,6-diMeOPhSe)$_2$	--	8 h	13%
**4**	Acridine	(2,6-diMeOPhSe)$_2$	--	8 h	4%
**5**	TAPT	(2-MeOPhSe)$_2$	--	8 h	9%
**6**	Acridine	(2-MeOPhSe)$_2$	--	8 h	0%
**7**	TAPT	Mono-Se	--	8 h	21%
**8**[a]	Hybrid-Se	--	--	6 h	0%
**9**[a]	Hybrid-Se	--	--	24 h	0%
**10**	TAPT	$(PhSe)_2$	1,1'-biphenyl	8 h	24%
**11**	TAPT	$(PhSe)_2$	naphthalene	8 h	17%
**12**	TAPT	$(PhSe)_2$	phenanthrene	8 h	7%
**13**	TAPT	(2,6-diMeOPhSe)$_2$	1,1'-biphenyl	8 h	2%
**14**	TAPT	(2,6-diMeOPhSe)$_2$	naphthalene	8 h	19%
**15**	TAPT	(2,6-diMeOPhSe)$_2$	phenanthrene	8 h	12%

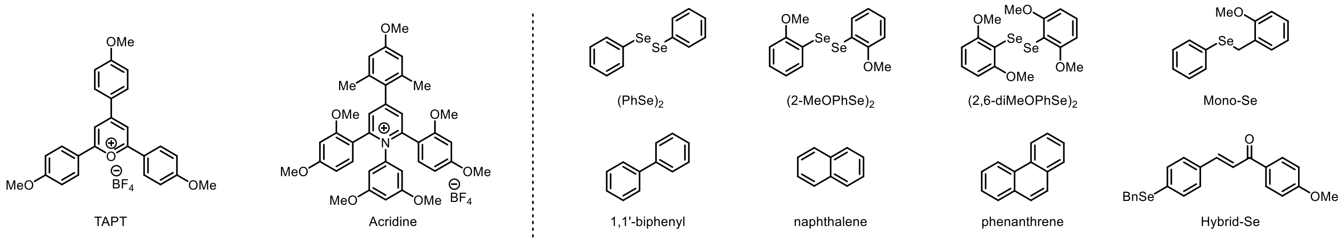

Reaction condition: **1al** (0.50 mmol, 1.0 equiv.), **2t** (5.0 equiv.), photocatalyst (5 mol%), Se-catalyst (10 mol%), PhCl (0.1 M), air balloon, irradiation with blue LEDs (λ_{max} = 448 or 440-460 nm, 472 or 425 mW respectively). ^{1}H NMR yield of **3am** was measured with 1,1,2-trichloroethylene as the internal standard. [a]20 mol% was used.

Kinetic study of the allylation reacton of (*E*)-dec-5-ene and *N*,4-dimethylbenzenesulfonamide with (PhSe)₂

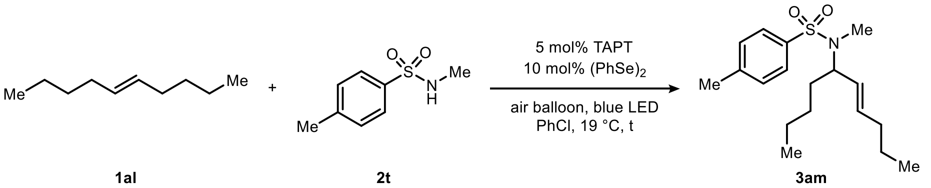

(*E*)-dec-5-ene **1al** (0.50 mmol, 1.0 equiv.), *N*,4-dimethylbenzenesulfonamide **2t** (5.0 equiv.), TAPT (5 mol%), $(PhSe)_2$ (10 mol%) and PhCl (5 mL) were added into a 40 mL vial. The reaction mixture was vigorously stirred at 19 °C under irradiation with blue LEDs (λ_{max} = 448 or 440-460 nm, 472 or 425 mW respectively) and equipped with an air balloon. For the given time, the reaction was stopped, and the solvent was removed under reduced pressure. ^{1}H NMR yield of **3am** was detected with 1,1,2-trichloroethylene as the internal standard. The average of two reactions for each entry is given as follow:

**entry**	**1**	**2**	**3**	**4**	**5**	**6**	**7**	**8**
**time [h]**	1	2	3	4	5	6	8	16
**^{1}H-NMR yield**	0%	0%	2%	2%	2%	10%	21%	19%

Kinetic study of the allylation reacton (*E*)-dec-5-ene and *N*,4-dimethylbenzenesulfonamide with Mono-Se

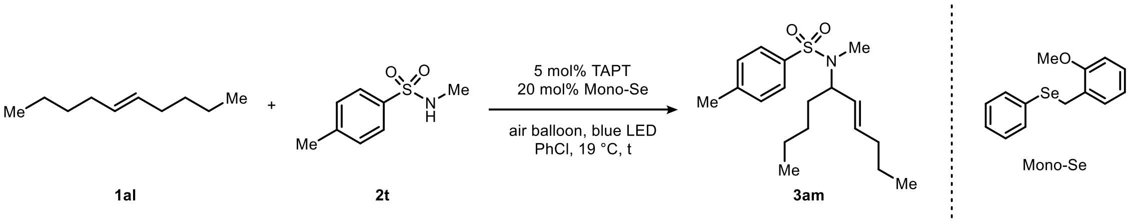

(*E*)-dec-5-ene **1al** (0.50 mmol, 1.0 equiv.), *N*,4-dimethylbenzenesulfonamide **2t** (5.0 equiv.), TAPT (5 mol%), Mono-Se (20 mol%) and PhCl (5 mL) were added into a 40 mL vial. The reaction mixture was vigorously stirred at 19 °C under irradiation with blue LEDs (λ_{max} = 448 or 440-460 nm, 472 or 425 mW respectively) and equipped with an air balloon. For the given time, the reaction was stoppe, and the solvent was removed under reduced pressure. ^{1}H NMR yield of **3am** was detected with 1,1,2-trichloroethylene as the internal standard. The average of two reactions for each entry is given as follow:

**entry**	**1**	**2**	**3**	**4**	**5**	**6**	**7**	**8**	**9**
**time [h]**	1	2	3	4	5	6	8	10	16
**^{1}H-NMR yield**	0%	3%	6%	8%	16%	18%	21%	19%	9%

Figure 2. Kinetic study with $(PhSe)_2$ (left) and Mono-Se (right).

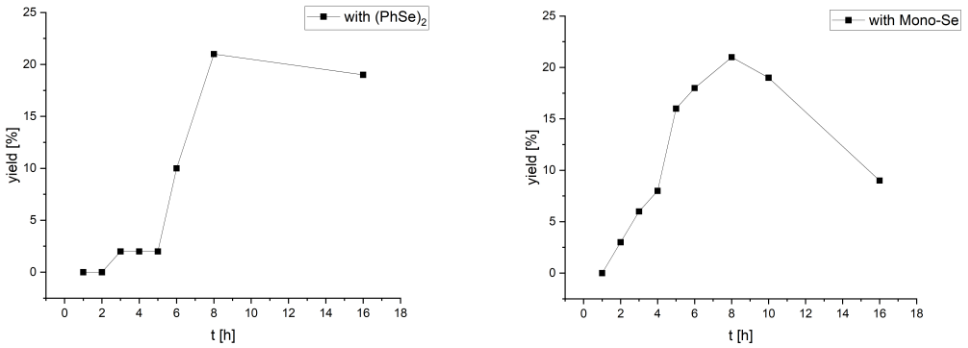

Table S5. Optimization of catalyst loading and reaction time.

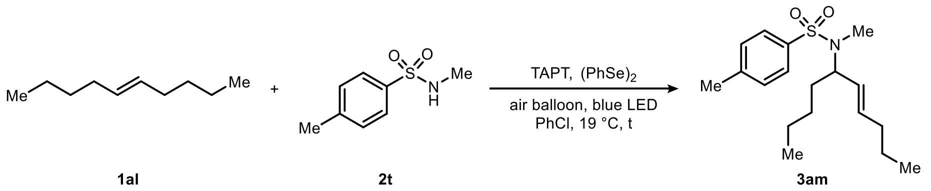

Entry	TAPT [mol%]	$(PhSe)_2$ [mol%]	Time	^{1}H NMR Yield	Entry	TAPT [mol%]	$(PhSe)_2$ [mol%]	Time	^{1}H-NMR Yield
**1**	5	10	6 h	10%	**10**	25	50	8 h	0%
**2**	5	20	6 h	4%	**11**	5	10	16 h	19%
**3**	5	50	6 h	0%	**12**	5	20	16 h	27%
**4**	10	10	6 h	5%	**13**	5	50	16 h	3%
**5**	5	10	8 h	21%	**14**	10	10	16 h	19%
**6**	5	20	8 h	18%	**15**	10	20	16 h	21%
**7**	10	10	8 h	23%	**16**	25	50	16 h	0%
**8**	10	20	8 h	28% (24%)	**17**	5	20	24 h	7%
**9**	20	10	8 h	20%	**18**	10	20	24 h	28%

Conditions: **1al** (0.50 mmol, 1.0 equiv.), **2t** (5.0 equiv.), TAPT, $(PhSe)_2$, PhCl (0.1 M) air balloon, irradiation with blue LEDs (λ_{max} = 448 or 440-460 nm, 472 or 425 mW respectively). Average of two reactions given. ^{1}H NMR yield of **3am** was measured with 1,1,2-trichloroethylene as the internal standard. Isolated yield in parentheses.

Figure S3. Optimization of catalyst loading and reaction time.

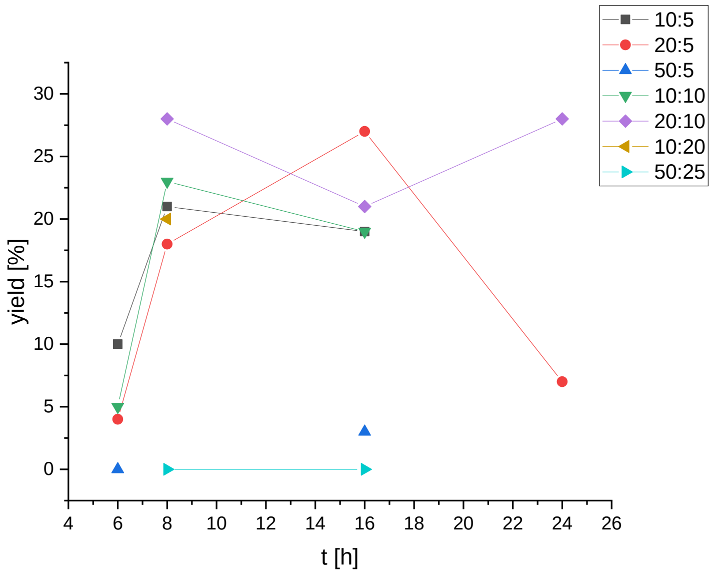

Table S6. Reaction optimization with different catalysts and additives.

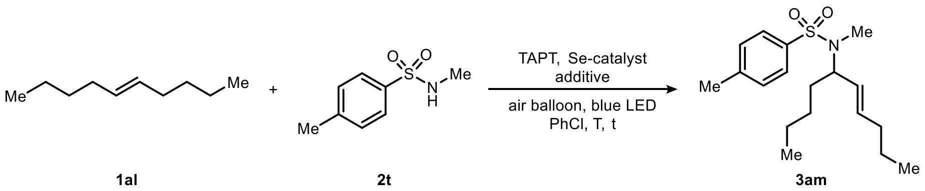

entry	TAPT	Se-catalyst	additive	time	temperature	^{1}H-NMR yield
**1**	5 mol%	Mono-Se, 20 mol%	Li_2CO_3, 4.00 equiv.	6 h	19 °C	0%
**2**	5 mol%	Mono-Se, 20 mol%	Na_2CO_3, 4.00 equiv.	6 h	19 °C	0%
**3**	5 mol%	Mono-Se, 20 mol%	K_2CO_3, 4.00 equiv.	6 h	19 °C	0%
**4**	5 mol%	Mono-Se, 20 mol%	$CsCO_3$, 4.00 equiv.	6 h	19 °C	0%
**5**	5 mol%	Mono-Se, 20 mol%	$NaHCO_3$, 4.00 equiv.	6 h	19 °C	0%
**6**	5 mol%	Mono-Se, 20 mol%	$KHCO_3$, 4.00 equiv.	6 h	19 °C	5%
**7**	5 mol%	Mono-Se, 20 mol%	Na_2HPO_4, 4.00 equiv.	6 h	19 °C	0%
**8**	5 mol%	Mono-Se, 20 mol%	K_2HPO_4, 4.00 equiv.	6 h	19 °C	0%
**9**	5 mol%	Mono-Se, 20 mol%	CaF_2, 4.00 equiv.	6 h	19 °C	21%
**10**	5 mol%	Mono-Se, 20 mol%	CsF, 1.50 equiv.	6 h	19 °C	5%
**11**	10 mol%	$(PhSe)_2$, 20 mol%	$(4\text{-ClPhS})_2$, 0.20 equiv.	4 h	19 °C	6%
**12**	10 mol%	$(PhSe)_2$, 20 mol%	$(4\text{-ClPhS})_2$, 0.20 equiv.	8 h	19 °C	21%
**13**[a]	10 mol%	$(PhSe)_2$, 20 mol%	$(4\text{-ClPhS})_2$, 0.20 equiv.	8 h	19 °C	0%
**14**	10 mol%	$(PhSe)_2$, 20 mol%	$(4\text{-ClPhS})_2$, 0.20 equiv.	16 h	19 °C	19%
**15**	10 mol%	$(PhSe)_2$, 20 mol%	2-Nitrobenzaldehyde, 0.25 equiv.	8 h	19 °C	6%
**16**	10 mol%	$(PhSe)_2$, 20 mol%	2-Nitrobenzaldehyde, 0.25 equiv.	24 h	19 °C	22%
**17**	10 mol%	$(PhSe)_2$, 20 mol%	$(4\text{-ClPhS})_2$, 0.20 equiv. and 2-Nitrobenzaldehyde, 0.25 equiv.	8 h	19 °C	20% (20%)
**18**	10 mol%	$(PhSe)_2$, 20 mol%	$(4\text{-ClPhS})_2$, 0.20 equiv. and 2-Nitrobenzaldehyde, 0.25 equiv.	16 h	19 °C	23%
**19**	10 mol%	$(PhSe)_2$, 20 mol%	--	8 h	19 °C	28% (24%)
**20**	10 mol%	$(PhSe)_2$, 20 mol%	--	8 h	30 °C	26%
**21**	10 mol%	$(PhSe)_2$, 20 mol%	--	8 h	50 °C	17%
**22**	10 mol%	$(PhSe)_2$, 10 mol%	--	8 h	19 °C	23%
**23**[b]	5 mol%	Mono-Se, 20 mol%	--	12 h	19 °C	6%
**24**[c]	5 mol%	Mono-Se, 20 mol%	--	12 h	19 °C	24%
**25**[d]	5 mol%	Mono-Se, 20 mol%	--	12 h	19 °C	14%
**26**	10 mol%	--	--	8 h	19 °C	0%
**27**	--	$(PhSe)_2$, 10 mol%	--	8 h	19 °C	0%

**28**[e]	10 mol%	$(PhSe)_2$, 10 mol%	--	8 h	19 °C	0%
**29**[f]	5 mol%	$(PhSe)_2$, 10 mol%	--	8 h	19 °C	0%

Conditions: **1al** (0.50 mmol, 1.0 equiv.), **2t** (5.0 equiv.), TAPT, $(PhSe)_2$, PhCl (0.1 M), air balloon, irradiation with blue LEDs (λ_{max} = 448 or 440-460 nm, 472 or 425 mW respectively). Average of two reactions, ^1H NMR yield of **3am** was measured with 1,1,2-trichloroethylene as the internal standard. Isolated yields in parentheses. [a]**1al** (5.00 equiv.), **2t** (0.50 mmol, 1.00 equiv.). [b]Second addition of Mono-Se (20 mol%) after 6 h. [c]Second addition of TAPT (5 mol%) after 6 h. [d]Second addition of TAPT (5 mol%) and $(PhSe)_2$ (20 mol%) after 6 h. [e]Either no light or N_2-atmosphere. [f]O_2-atmosphere, with molecular sieve (4 Å, 15 mg).

Table S7. Analysis of Substrate decomposition.

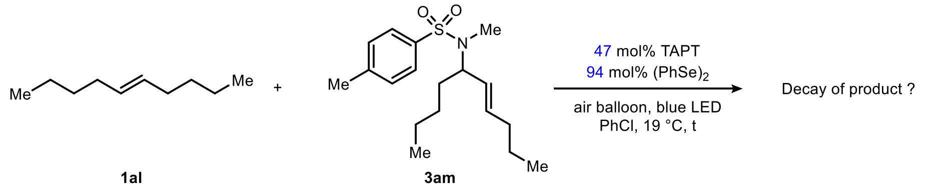

Entry	3am	Time	Residue of 3am
**1**	52.7 µmol	16 h	87%
**2**	52.6 µmol	40 h	82%
**3**	52.8 µmol	112 h	21%

Reaction condition: **1al** (47.2 equiv.), **3am** (0.05 mmol, 1.0 equiv.) TAPT (47 mol%), $(PhSe)_2$ (94 mol%), PhCl (10.6 mM), air balloon, irradiation with blue LEDs (λ_{max} = 448 or 440-460 nm, 472 or 425 mW respectively). Residue of **3am** was determined via ^1H-NMR analysis and 1,1,2-trichloroethylene as the internal standard.

Table S8. Reaction optimization of the allylation reaction of cyclododecene (**1am**) and 1H-benzo[d][1,2,3]triazole (**2f**).

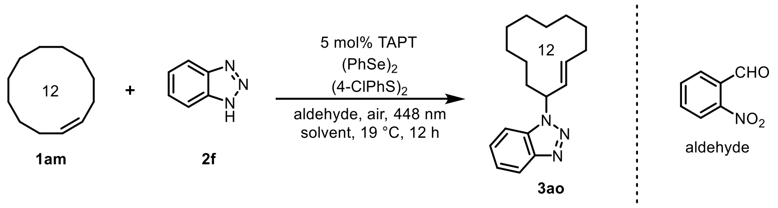

entry	1am	2f	(PhSe)2	(4-ClPhS)2	2-nitrobenzaldehyde	solvent	^{1}H-NMR yield
**1**	0.30 mmol	3.00 equiv.	15 mol%	5 mol%	0.25 equiv.	DCE	56%
**2**	0.30 mmol	3.00 equiv.	15 mol%	5 mol%	--	DCE	55%
**3**	0.30 mmol	3.00 equiv.	15 mol%	5 mol%	1.00 equiv.	DCE	49%
**4**	0.30 mmol	3.00 equiv.	10 mol%	--	0.25 equiv.	DCE	40%
**5**	0.30 mmol	3.00 equiv.	10 mol%	5 mol%	0.25 equiv.	DCE	53%
**6**[a]	0.30 mmol	3.00 equiv.	10 mol%	5 mol%	0.25 equiv.	DCE	55%
**7**[b]	0.30 mmol	4.50 equiv.	15 mol%	7.5 mol%	0.38 equiv.	DCE	61%
**8**	3.00 equiv.	0.30 mmol	10 mol%	5 mol%	0.25 equiv.	DCE	37%
**9**[c]	0.30 mmol	3.00 equiv.	10 mol%	5 mol%	0.25 equiv.	DCE	54%
**10**	0.30 mmol	3.00 equiv.	10 mol%	5 mol%	0.25 equiv.	CH_3CN	32%
**11**	0.30 mmol	3.00 equiv.	10 mol%	5 mol%	0.25 equiv.	Acetone	<10%
**12**	0.30 mmol	3.00 equiv.	10 mol%	5 mol%	0.25 equiv.	$CHCl_3$	52%
**13**[d]	0.30 mmol	3.00 equiv.	10 mol%	5 mol%	0.25 equiv.	o-xylene	<10%
**14**[d]	0.30 mmol	3.00 equiv.	10 mol%	5 mol%	0.25 equiv.	1-chloro-2-methylbenzene	<10%
**15**[d]	0.30 mmol	3.00 equiv.	10 mol%	5 mol%	0.25 equiv.	(trifluoromethyl) benzene	<10%
**16**[e]	0.30 mmol	3.00 equiv.	10 mol%	5 mol%	0.25 equiv.	DCE/Tolune	52%
**17**[e]	0.30 mmol	3.00 equiv.	10 mol%	5 mol%	0.25 equiv.	DCE/o-xylene	48%
**18**[e]	0.30 mmol	3.00 equiv.	10 mol%	5 mol%	0.25 equiv.	DCE/1-chloro-2-methylbenzene	53%
**19**[e]	0.30 mmol	3.00 equiv.	10 mol%	5 mol%	0.25 equiv.	DCE/ (trifluoromethyl) benzene	53%

Conditions: **1am**, **2f**, TAPT (5 mol%), $(PhSe)_2$, $(4\text{-}ClPhS)_2$, 2-Nitrobenzaldehyde, solvent (4 mL), open to air, blue light irradiation for 12 h. ^{1}H NMR yield of **3ao** was determined with 1,4-dimethoxybenzene as the internal standard. [a]10 mol% TAPT was used. [b]7.5 mol% TAPT was used. [c]2 mL DCE was used. [d]2 mL solvent was used. [e]Ratio of the two solvents was 3:1.

Table S9. Optimization with different catalysts and additives.

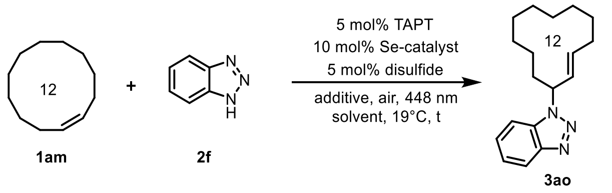

entry	Se-catalyst	disulfide	additive	time	^{1}H-NMR yield
**1**	$(PhSe)_2$	$(4\text{-}ClPhS)_2$	2-Nitrobenzaldehyde, 0.25 equiv.	12 h	53%
**2**	$(PhSe)_2$	--	2-Nitrobenzaldeyde, 0.25 equiv.	12 h	40%
**2**	$(PhSe)_2$	$(4\text{-}ClPhS)_2$	NaF, 1.00 equiv.	12 h	52%
**3**	$(PhSe)_2$	$(4\text{-}ClPhS)_2$	SiO_2, 13.0 mg	12 h	54%
**4**	$(PhSe)_2$	$(4\text{-}MeOPhS)_2$	2-Nitrobenzaldeyde, 0.25 equiv.	12 h	51%
**5**	$(PhSe)_2$	dibenzo[c,e][1,2]dithiine	2-Nitrobenzaldeyde, 0.25 equiv.	12 h	49%
**6**	$(4\text{-}MePhSe)_2$	$(4\text{-}ClPhS)_2$	2-Nitrobenzaldeyde, 0.25 equiv.	7 h	59%
**7**	$(2\text{-}MeOPhSe)_2$	$(4\text{-}ClPhS)_2$	2-Nitrobenzaldeyde, 0.25 equiv.	7 h	57%
**8**	$(2,4\text{-}diMeOPhSe)_2$	$(4\text{-}ClPhS)_2$	2-Nitrobenzaldeyde, 0.25 equiv.	12 h	42%
**9**	$(3,5\text{-}diMeOPhSe)_2$	$(4\text{-}ClPhS)_2$	2-Nitrobenzaldeyde, 0.25 equiv.	12 h	33%
**10**[a]	$(4\text{-}MePhSe)_2$	$(4\text{-}ClPhS)_2$	2-Nitrobenzaldeyde, 0.25 equiv.	12 h	49%
**11**[b]	$(4\text{-}MePhSe)_2$	$(4\text{-}ClPhS)_2$	2-Nitrobenzaldeyde, 0.25 equiv.	26 h	57%
**12**[c]	$(PhSe)_2$	$(4\text{-}ClPhS)_2$	--	21 h	52%

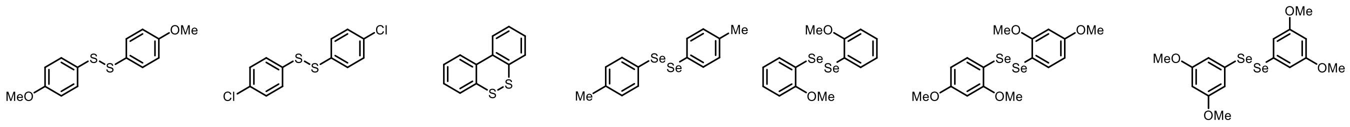

$(4\text{-}MeOPhS)_2$ $(4\text{-}ClPhS)_2$ dibenzo[c,e][1,2]dithiine $(4\text{-}MePhSe)_2$ $(2\text{-}MeOPhSe)_2$ $(2,4\text{-}diMeOPhSe)_2$ $(3,5\text{-}diMeOPhSe)_2$

Conditions: **1am** (0.30 mmol, 1.00 equiv.), **2f** (3.00 equiv.), TAPT (5 mol%), Se-catalyst (10 mol%), disulfide (5 mol%), additive, DCE (4 mL), open to air, blue light irradiation. ^{1}H NMR yield of **60** was determined with 1,4-dimethoxybenzene as the internal standard. [a]10 mol% $(4\text{-}ClPhS)_2$ was used. [b]1.00 mmol reaction. [c]**1am** (0.96 mmol, 1.00 equiv.), **2f** (3.00 mmol, 3.13 equiv.).

Investigation Toward the Isomerization of the Allylic Products

Table S10. Optimization of the isomerization of *N*-allylic compound **3a**.

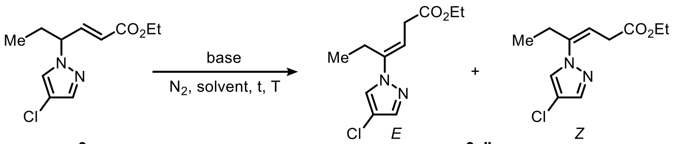

3a **3a''**

entry	3a	base	solvent	t	T	yield (3a'')	*E* : *Z*	residue of 3a	3a'':3a
**1**	0.1 mmol	TEA	DCE	2 h	r.t.	<5%	--	>95%	--
**2**	0.1 mmol	2,6-lutidine	DCE	2 h	r.t.	<5%	--	>95%	--
**3**	0.1 mmol	DBU	DCE	2 h	r.t.	65%	2:1	17%	4:1
**4**	0.1 mmol	TBD	DCE	2 h	r.t.	62%	2:1	17%	4:1
**5**	0.1 mmol	TMG	DCE	18 h	r.t.	57%	2:1	34%	2:1
**6**	0.1 mmol	DBU	DCE	18 h	r.t.	74%	2:1	20%	4:1
**7**	0.1 mmol	DBU	CH_3CN	2 h	r.t.	66%	2:1	21%	3:1
**8**	0.1 mmol	DBU	Acetone	2 h	r.t.	61%	2:1	23%	3:1
**9**	0.1 mmol	DBU	THF	2 h	r.t.	67%	2:1	20%	3:1
**10**	0.1 mmol	DBU	THF	24 h	r.t.	69%	2:1	24%	3:1
**11**	0.1 mmol	DBU	DCE	2 h	60 °C	73%	2:1	20%	4:1
**12**	0.1 mmol	DBU	CH_3CN	2 h	60 °C	67%	2:1	21%	3:1
**13**	0.1 mmol	DBU	Acetone	2 h	60 °C	63%	2:1	23%	3:1
**14**	0.1 mmol	DBU	THF	2 h	60 °C	61%	2:1	18%	3:1
**15**	0.1 mmol	DBU	THF	2 h	-95 °C	<5%	--	>95%	--
**16**[a]	0.1 mmol	LiO^{t}Bu	THF	2 h	r.t.	57%	1:1	7%	8:1
**17**[a]	**0.1 mmol**	**LiO^{t}Bu**	**THF**	**1 h**	**0 °C**	**87%**	**1:1**	**4%**	**25:1**
**18**[b]	0.1 mmol	LiO^{t}Bu	THF	1 h	0 °C	62%	2:1	3%	19:1
**19**[a]	0.2 mmol	LiO^{t}Bu	THF	1 h	0 °C	86% (88%)	1:1 (1:1)	2%	49:1 (>49:1)

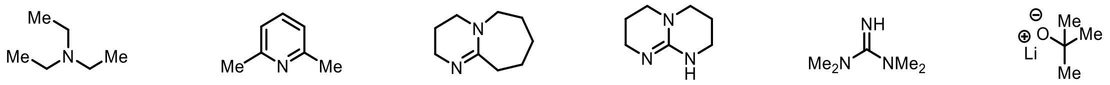

TEA 2,6-lutidine DBU TBD TMG LiO^{t}Bu

Conditions: **3c** (0.10 mmol, 1.00 equiv.), base (2.00 equiv.), solvent (2 mL), N_2, r.t.. After reaction, AcOH (0.05 mL) was added to quench the reaction. ^{1}H NMR yield and the ratio were determined with 1,4-dimethoxybenzene as the internal standard. Isolated yield and ratio are given in parentheses [a]5.00 equiv. LiO^{t}Bu was used, saturated NH_4Cl (aq., 0.5 mL) was added to quench the reaction. [b]3.00 equiv. LiO^{t}Bu was used, saturated NH_4Cl (aq., 0.5 mL) was added to quench the reaction.

Table S11. Isomerization of *N*-allylic compounds with different electron-withdrawing groups.

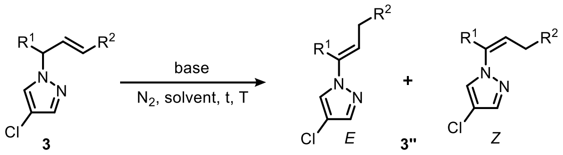

entry	substrate	base	solvent	time	temperature	product	^{1}H-NMR yield	*E* : *Z*	residue of 3	3'':3
**1**	**3ae**	DBU	DCE	5 h	r.t.	**3ae''**	73%	7:1	0%	>49:1
**2**	R^1=Me; R^2=SO_2Ph	LiO^{t}Bu	THF	1 h	0 °C	**3ae''**	90% (92%)	4:1 (4:1)	0%	>49:1
**3**	**3ag**	DBU	DCE	5 h	r.t.	**3ag''**	86% (74%)	4:1 (*E*)	7%	10:1 (10:1)
**4**	R^1=Me; R^2=CN	LiO^{t}Bu	THF	1 h	0 °C	**3ag''**	0%	--	--	--
**5**	**3aj**	DBU	DCE	5 h	r.t.	**3aj''**	94% (97%)	7:1 (7:1)	0%	>49:1
**6**	R^1=H; R^2=PO(Ph)$_2$	LiO^{t}Bu	THF	1 h	0 °C	**3aj''**	0%	--	--	--

Conditions: **3c** (0.20 mmol, 1.00 equiv.), base (with DBU: 2.00 equiv.; with LiO^{t}Bu: 5.00 equiv.), solvent (4 mL), N_2. After reaction, AcOH (0.05 mL) was added to quench the reaction with DBU, saturated NH_4Cl (aq., 1.00 mL) was added to quench the reaction with LiO^{t}Bu. ^{1}H NMR yield and the ratio were determined with 1,4-dimethoxybenzene as the internal standard. Isolated yield and ratio are given in parentheses

Table S12. Isomerization of *N*-allylic compound **3c.**

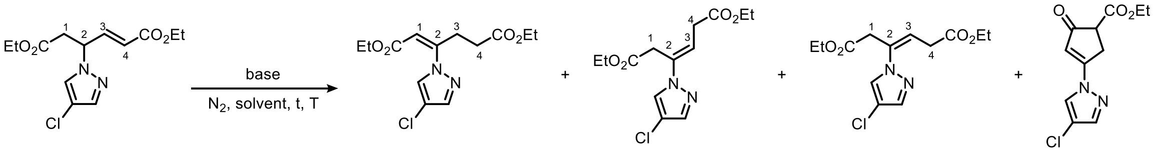

3c **3c''**, C12 **3c''**, C23, *E* **3c''**, C23, *Z* **4a**

entry	3c	base	solvent	time	temperature	^{1}H-NMR yield of 3c''		^{1}H-NMR Yield 4a	Residue 3c
						C12	C23 (*E*:*Z*)		
**1**	0.1 mmol	DBU	DCE	5 h	60 °C	52%	34% (3:1)	--	10%
**2**	0.2 mmol	DBU	DCE	18 h	60 °C	52%	38% (4:1)	--	10%
**3**	0.1 mmol	TBD	DCE	5 h	60 °C	51%	32% (3:1)	--	9%
**4**	0.1 mmol	TMG	DCE	5 h	60 °C	17%	61% (3:1)	--	17%
**5**[a]	0.2 mmol	LiO*t*Bu	THF	1 h	0 °C	--	--	74% (72%)	--

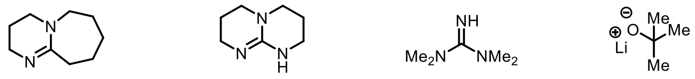

DBU TBD TMG LiO*t*Bu

Conditions: **3c** (0.10 mmol, 1.00 equiv.), base (2.00 equiv.), solvent (2 mL), N_2. After reaction, AcOH (0.05 mL) was added to quench the reaction. ^{1}H NMR yield and the ratio were determined with 1,4-dimethoxybenzene as the internal standard. Isolated yield and ratio are given in parentheses. [a]5.00 equiv. LiO*t*Bu was used, saturated NH_4Cl (aq., 0.5 mL) was added to quench the reaction.

Initial Study of the Assymetric Intermolecular *N*-Allylation Reaction

Scheme S4. Chiral reaction of ethyl (E)-hex-3-enoate 1a and 4-chloro-1H-pyrazole 2a with different chiral Se-catalysts.

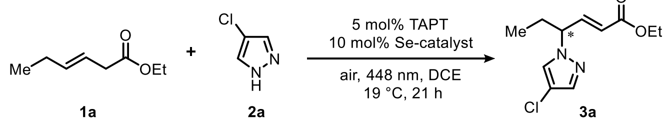

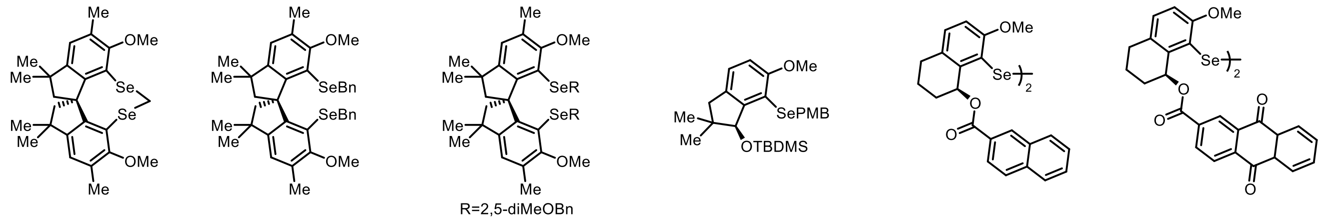

5c, 24%, 67:33 e.r.[a] | **5d**, 22%, 66:34 e.r.[a] | **5e**, 13%%, 70:30 e.r. | **5i**, 40%, 72:28 e.r.[b] | **5a**, 44%, 63:37 e.r. | **5f**, 3%, -- e.r.[c]

Conditions: **1a** (3.0 equiv.), **2a** (0.30 mmol, 1.0 equiv.), TAPT (5 mol%), Se-catalyst (10 mol%), DCE (4 mL), open to air, blue light irradiation for 21 h. ^{1}H NMR yield of **3a** was determined with 1,4-dimethoxybenzene as the internal standard. [a]TAPT (10 mol%) was used. [b]**5i** (20 mol%) was used. [c]**5f** (5 mol%) was used. Without TAPT.

Table S13. Reaction optimization of chiral reaction of ethyl (*E*)-hex-3-enoate (**1a**) and 4-chloro-1H-pyrazole (**2a**).

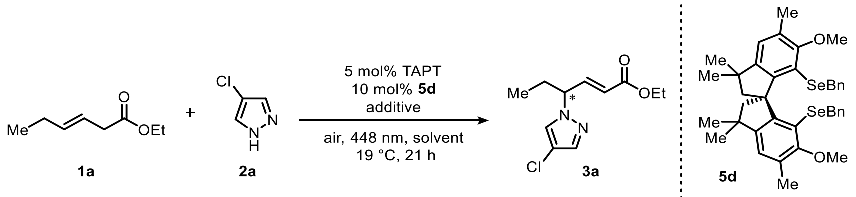

Entry	1a	2a	Additive	Solvent	Temperature	^{1}H-NMR Yield	e.r.
**1**[a]	3.0 equiv.	0.30 mmol	$Sc(OTf)_3$ 2.5 mol%	DCE	19 °C	23%	66:34
**2**[a]	0.30 mmol	3.0 equiv.	$Sc(OTf)_3$ 10 mol%	DCE	19 °C	26%	69:31
**3**[a]	3.0 equiv.	0.30 mmol	$Zn(OTf)_2$ 2.5 mol%	DCE	19 °C	22%	65:35
**4**	3.0 equiv.	0.30 mmol	$(4\text{-ClPhS})_2$ 5.0 mol%	DCE	19 °C	22%	63:37
**5**	3.0 equiv.	0.30 mmol	--	Acetone	19 °C	4%	--
**6**	3.0 equiv.	0.30 mmol	--	CH_3CN	19 °C	15%	--
**7**	3.0 equiv.	0.30 mmol	--	Toluene	19 °C	6%	--
**8**	3.0 equiv.	0.30 mmol	--	*o*-Xylene	19 °C	6%	--
**9**	0.30 mmol	3.0 equiv.	--	DCE	19 °C	22%	63:37
**10**[b]	3.0 equiv.	0.30 mmol	--	DCE	19 °C	18%	65:35
**11**	3.0 equiv.	0.30 mmol	--	DCE	-3 °C	15%	61:39

Condition: **1a**, **2a**, TAPT (5 mol%), **5d** (10 mol%), DCE (4 mL), open to air, blue light irradiation for 21 h. ^{1}H NMR yield of **3a** was determined with 1,4-dimethoxybenzene as the internal standard. [a]TAPT (10 mol%) was used. [b]DCE (2 mL) was used.

Scheme S5. Chiral reaction of diethyl (*E*)-hex-3-enedioate (**1c**) and 4-chloro-1H-pyrazole (**2a**) with different chiral Se-catalysts.

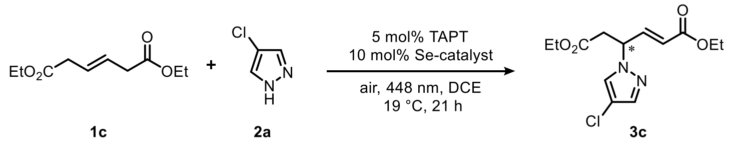

1c **2a** **3c**

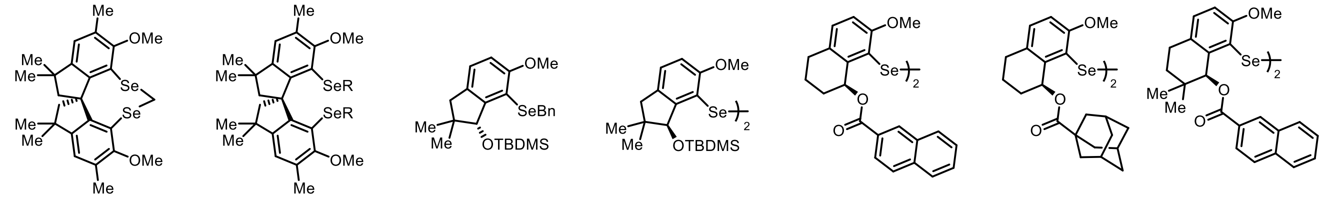

5c, 35%, 88:12 e.r.; **5d**, R=Bn, 44%, 84:26 e.r.; **5e**, R=2,5-diMeOBn, 29%, 89:11 e.r.; **5j**, 64%, 24:76 e.r.[a]; **5b**, 75%, 68:32 e.r.; **5a**, 70%, 66:34 e.r.; **5g**, 71%, 45:55 e.r.; **5h**, 66%, 61:39 e.r.

Conditions: **1c** (0.9 mmol), **2a** (0.30 mmol, 1.0 equiv.), TAPT (5 mol%), Se-catalyst (10 mol%), DCE (4 mL), open to air, blue light irradiation for 21 h. ^{1}H NMR yield of **3c** was determined with 1,4-dimethoxybenzene as the internal standard. [a]**5j** (20 mol%) was used.

Table S14. Reaction optimization of the chiral reaction of diethyl (*E*)-hex-3-enedioate (**1c**) and 4-chloro-1H-pyrazole (**2a**).

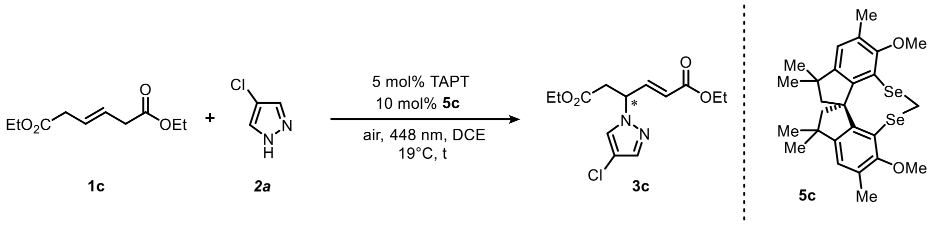

Entry	Solvent	Additive	Temperature	Time	^{1}H-NMR Yield	e.r.
**1**	DCE	--	19 °C	21 h	35%	88:12
**2**	DCE	--	19 °C	8 h	32%	92:8
**3**	Acetone	--	19 °C	8 h	7%	92:8
**4**	HFIP	--	19 °C	8 h	<6%	--
**5**	$CHCl_3$	--	19 °C	8 h	17%	90:10
**6**	CH_3CN	--	19 °C	8 h	14%	92:8
**7**	CH_3CN	$(4\text{-ClPhS})_2$, 5 mol%	19 °C	8 h	27%	94:6
**8**	DCE	$(4\text{-ClPhS})_2$, 5 mol%	19 °C	8 h	33%	91:9
**9**	DCE	$(4\text{-ClPhS})_2$, 10 mol%	19 °C	8 h	29%	91:9
**10**	DCE	$(^t\text{BuS})_2$, 5 mol%	19 °C	8 h	33%	92:8
**11**	DCE	$(4\text{-MeOPhS})_2$, 5 mol%	19 °C	8 h	26%	92:8
**12**	DCE	Me_6Si_2, 1.00 equiv.	19 °C	8 h	0%	--
**13**	DCE	$Sc(OTf)_3$, 10 mol%	19 °C	8 h	30%	87:13
**14**	DCE	$ZnCl_2$, 10 mol%	19 °C	8 h	23%	92:8
**15**	DCE	SiO_2, 1.00 equiv.	19 °C	8 h	30%	93:7
**16**[a]	DCE	--	19 °C	8 h	19%	88:12
**17**	DCE	--	0 °C	8 h	26%	93:17
**18**[b]	DCE	--	19 °C	8 h	29%	94:6

Conditions: **1c** (0.9 mmol, 3.00 equiv.), **2a** (0.3 mmol, 1.00 equiv.), TAPT (5 mol%), **5c** (10 mol%), DCE (4 mL), open to air, blue light irradiation. ^{1}H NMR yield of **3c** was determined with 1,4-dimethoxybenzene as the internal standard. [a]**1a** (0.3 mmol, 1.00 equiv.), **1b** (0.9 mmol, 3.00 equiv.). [b]**5c** (20 mol%).

Substrate Scope of the Internal *N*-Allylation Reaction

General procedure A for the racemic reaction:

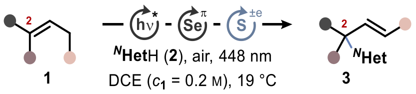

Substrates (alkene and amine), TAPT (5 mol%), $(PhSe)_2$ (10 mol%) and solvent (DCE, DCE/HFIP = 3:2, or PhCl) were added into a 100 mL round bottom flask. In some cases, additive $(4\text{-}ClPhS)_2$ (5 mol%) was used. The flask was equipped with a septum including two cannulas. The reaction mixture was then vigorously stirred at 19 °C under irradiation at 448 nm for the given time. After the reaction was finished, the solvent was removed under reduced pressure. The ^{1}H NMR-yield of allylic product and the ratio of allylic and vinylic isomer was determined with internal standard 1,4-dimethoxybenzene. If no obvious signal for the vinylic isomer in the crude ^{1}H-NMR was detected, no total yield is given, and the ratio is given as >10:1. Further purification via column chromatography provided the target compound.

Ethyl (*E*)-4-(4-chloro-1H-pyrazol-1-yl)hex-2-enoate (3a):

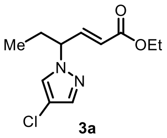

3a

According to general procedure A: Ethyl (*E*)-hex-3-enoate (**1a**) (142 mg, 1.00 mmol, 1.00 equiv.), 4-chloro-1H-pyrazole (**2a**) (308 mg, 3.00 mmol, 3.00 equiv.), TAPT (24.3 mg, 50.0 µmol, 5 mol%), $(PhSe)_2$ (31.6 mg, 101 µmol, 10 mol%) and DCE (5 mL), 21 h. After purification (PE/EtOAc, 9:1), the product was isolated as a colorless oil (166 mg, 684 µmol, 68%; allylic/vinylic isomer >49:1). Crude ^{1}H NMR analysis: total yield: 73%; allylic isomer: 68%; allylic/vinylic isomer: 14:1.

According to general procedure A: Ethyl (*E*)-hex-3-enoate (**1a**) (142 mg, 1.00 mmol, 1.00 equiv.), 4-chloro-1H-pyrazole (**2a**) (308 mg, 3.00 mmol, 3.00 equiv.), TAPT (24.3 mg, 50.0 µmol, 5 mol%), $(PhSe)_2$ (31.2 mg, 100 µmol, 10 mol%), $(4\text{-}ClPhS)_2$ (14.4 mg, 50.0 µmol, 5 mol%) and DCE (5 mL), 21 h. Crude ^{1}H NMR analysis: total yield: 66%; allylic isomer: 63%; allylic/vinylic isomer: 25:1.

According to general procedure A: Ethyl (*Z*)-hex-3-enoate (***Z*-1a**) (142 mg, 1.00 mmol, 1.00 equiv.), 4-chloro-1H-pyrazole (**2a**) (308 mg, 3.00 mmol, 3.00 equiv.), TAPT (24.3 mg, 50.0 µmol, 5 mol%), $(PhSe)_2$ (31.2 mg, 100 µmol, 10 mol%) and DCE (5 mL), 21 h. After purification (PE/EtOAc, 9:1), the product was isolated as a lightly yellow oil (123 mg, 507 mmol, 51%; allylic/vinylic isomer >49:1). Crude ^{1}H NMR analysis: total yield: 49%; allylic isomer: 47%; allylic/vinylic isomer: 24:1.

TLC: R_f = 0.51 (PE/EtOAc, 4:1) [UV, $KMnO_4$]. IR [cm^{-1}]: 3131, 2878, 2974, 2937, 1778, 1715, 1659, 1435, 1368, 1312, 1271, 1182, 1036, 969, 824, 790, 742. **^{1}H NMR** (300 MHz, $CDCl_3$): δ 7.46 – 7.41 (m, 1H), 7.38 (d, *J* = 0.8 Hz, 1H), 7.05 – 6.90 (m, 1H), 5.73 – 5.62 (m, 1H), 4.68 (dtd, *J* = 8.8, 6.1, 1.4 Hz, 1H), 4.24 – 4.07 (m, 2H), 2.14 – 1.83 (m, 2H), 1.23 (tdd, *J* = 7.1, 2.7, 1.3 Hz, 3H), 0.90 – 0.76 (m, 3H). **^{13}C NMR** (75 MHz, $CDCl_3$): δ 165.7, 145.1, 138.0, 126.3, 122.9, 110.2, 65.2, 60.7, 27.2, 14.2, 10.5. **HRMS** (EI) calcd. for $[C_{11}H_{15}ClN_2O_2]^{\cdot+}$ ($[M]^{\cdot+}$), m/z = 242.0817; found 242.0811.

Vinylic isomer: Ethyl (*Z*)-3-(4-chloro-1H-pyrazol-1-yl)hex-3-enoate (3a')

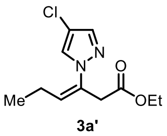

3a'

TLC: R_f = 0.57 (PE/EtOAc, 8:1) [UV]. IR [cm^{-1}]: 3138, 2974, 2363, 1737, 1681, 1431, 1372, 1341, 1178, 1252, 1029, 969, 842, 790. **^{1}H NMR** (400 MHz, $CDCl_3$): 7.57 – 7.45 (m, 2H), 5.51 (t, *J* = 7.3 Hz, 1H), 4.08 (q, *J* = 7.1 Hz, 2H), 3.52 (d, *J* = 0.5 Hz, 2H), 2.22 – 2.01 (m, 2H), 1.19 (t, *J* = 7.1 Hz, 3H), 1.02 (t, *J* = 7.5 Hz, 3H). **^{13}C NMR** (101 MHz, $CDCl_3$): δ 170.2, 138.5, 131.6, 129.6, 128.9, 110.3, 61.1, 41.0, 21.0, 14.2, 14.0. **HRMS** (EI) calcd. for $[C_{11}H_{15}ClN_2O_2]^{\cdot+}$ ($[M]^{\cdot+}$), m/z = 242.0817; found 242.0820.

Ethyl (*E*)-4-(4-chloro-1H-pyrazol-1-yl)dec-2-enoate (3b):

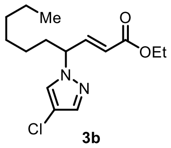
3b

According to general procedure A: Ethyl (*E*)-dec-3-enoate (**1b**) (198 mg, 1.00 mmol, 1.00 equiv.), 4-chloro-1H-pyrazole (**2a**) (308 mg, 3.00 mmol, 3.00 equiv.), TAPT (24.3 mg, 50.0 µmol, 5 mol%), $(PhSe)_2$ (31.2 mg, 100 µmol, 10 mol%) and DCE (5 mL), 21 h. After purification (PE/EtOAc, 30:1 to 20:1), the product was isolated as a lightly yellow oil (212 mg, 710 µmol, 71%; allylic/vinylic isomer >49:1). Crude ^{1}H NMR analysis: total yield: 69%; allylic isomer: 66%, allylic/vinylic isomer: 25:1.

According to general procedure A: Ethyl (*E*)-dec-3-enoate (**1b**) (198 mg, 1.00 mmol, 1.00 equiv.), 4-chloro-1H-pyrazole (**2a**) (308 mg, 3.00 mmol, 3.00 equiv.), TAPT (24.3 mg, 50.0 µmol, 5 mol%), $(PhSe)_2$ (31.2 mg, 100 µmol, 10 mol%), $(4\text{-ClPhS})_2$ (11.4 mg, 50.0 µmol, 5 mol%) and DCE (5 mL), 21 h. Crude ^{1}H NMR analysis: total yield: 69%; allylic isomer: 66%; allylic/vinylic isomer: 25:1.

TLC: R_f = 0.44 (PE/EtOAc, 10:1) [UV, $KMnO_4$]. IR [cm^{-1}]: 3131, 2930, 2859, 1718, 1659, 1525, 1465, 1435, 1368, 1312, 1271, 1178, 1234, 1178, 1115, 1036, 969, 839, 790, 727. **^{1}H NMR** (300 MHz, $CDCl_3$): δ 7.42 (d, *J* = 0.5 Hz, 1H), 7.37 (d, *J* = 0.7 Hz, 1H), 6.96 (dd, *J* = 15.7, 6.1 Hz, 1H), 5.65 (dd, *J* = 15.6, 1.5 Hz, 1H), 4.83 – 4.69 (m, 1H), 4.13 (q, *J* = 7.1 Hz, 2H), 2.10 – 1.94 (m, 1H), 1.92 – 1.75 (m, 1H), 1.30 – 1.14 (m, 11H), 0.81 (t, *J* = 6.7 Hz, 3H). **^{13}C NMR** (75 MHz, $CDCl_3$): δ 165.7, 145.4, 137.9, 126.2, 122.7, 110.2, 63.7, 60.7, 33.9, 31.5, 28.7, 25.8, 22.5, 14.2, 14.0. **HRMS** (ESI) calcd. for $[C_{15}H_{24}ClN_2O_2]^+$ ($[M+H]^+$), m/z = 299.1521; found 299.1525.

Diethyl (*E*)-4-(4-chloro-1H-pyrazol-1-yl)hex-2-enedioate (3c):

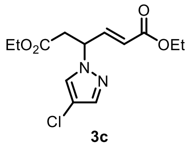
3c

According to general procedure A: Diethyl (*E*)-hex-3-enedioate (**1c**) (601 mg, 3.00 mmol, 3.00 equiv.), 4-chloro-1H-pyrazole (**2a**) (103 mg, 1.00 mmol, 1.00 equiv.), TAPT (24.3 mg, 50.0 µmol, 5 mol%), $(PhSe)_2$ (31.2 mg, 100 µmol, 10 mol%) and DCE (5 mL), 21 h. After purification (PE/EtOAc, 15:1), the product was isolated as a colorless oil (308 mg, 1.02 mmol, >99%; allylic/vinylic isomer >49:1. Crude ^{1}H NMR analysis: total yield: 99%; allylic isomer: 99%; allylic/vinylic isomer >10:1.

TLC: R_f = 0.23 (PE/EtOAc, 5:1) [UV]. IR [cm^{-1}]: 3131, 2982, 2937, 1718, 1662, 1372, 1312, 1271, 1178, 1025, 969, 857. **^{1}H NMR** (300 MHz, $CDCl_3$): δ 7.54 – 7.39 (m, 2H), 7.01 (dd, *J* = 15.6, 5.9 Hz, 1H), 5.71 (dd, *J* = 15.6, 1.5 Hz, 1H), 5.33 (dtd, *J* = 8.5, 5.8, 1.6 Hz, 1H), 4.24 – 4.08 (m, 4H), 3.22 (dd, *J* = 16.6, 8.8 Hz, 1H), 2.91 (dd, *J* = 16.6, 5.6 Hz, 1H), 1.27 (t, *J* = 7.1 Hz, 3H), 1.21 (t, *J* = 7.1 Hz, 3H). **^{13}C NMR** (101 MHz, $CDCl_3$): δ 169.5, 165.4, 143.6, 138.5, 127.4, 123.6, 110.4, 61.3, 60.9, 59.3, 38.2, 14.2, 14.2. **HRMS** (APCI) calcd. for $[C_{13}H_{18}ClN_2O_4]^+$ ($[M+H]^+$), m/z = 301.0950; found 301.0953.

Ethyl (*E*)-8-chloro-4-(4-chloro-1H-pyrazol-1-yl)oct-2-enoate (3d):

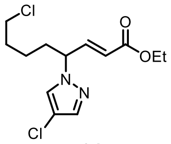
3d

According to general procedure A: Ethyl (*E*)-8-chlorooct-3-enoate (**1d**) (205 mg, 1.00 mmol, 1.00 equiv.), 4-chloro-1H-pyrazole (**2a**) (308 mg, 3.00 mmol, 3.00 equiv.), TAPT (24.3 mg, 50.0 µmol, 5 mol%), $(PhSe)_2$ (31.2 mg, 100 µmol, 10 mol%) and DCE (5 mL), 21 h. After purification (PE/EtOAc, 20:1 to 12:1), the product was isolated as a colorless oil (201 mg, 660 µmol, 66%; allylic/vinylic isomer 25:1). Crude ^{1}H NMR analysis: total yield: 71%; allylic isomer: 66%; allylic/vinylic isomer: 11:1.

TLC: R_f = 0.34 (PE/EtOAc, 5:1) [UV, $KMnO_4$]. IR [cm^{-1}]: 3131, 2982, 2941, 1870, 1715, 1659, 1525, 1435, 1368, 1312, 1271, 1182, 1096, 1036, 969, 842, 790, 768, 731. **^{1}H NMR** (300 MHz, $CDCl_3$): δ 7.43 (d, *J* = 0.5 Hz, 1H), 7.38 (d, *J* = 0.7 Hz, 1H), 6.95 (dd, *J* = 15.7, 6.1 Hz, 1H), 5.67 (dd, *J* = 15.6, 1.5 Hz, 1H), 4.82 – 4.71 (m, 1H), 4.13 (q, *J* = 7.1 Hz, 2H), 3.49 – 3.41 (m, 2H), 2.16 – 1.99 (m, 1H), 1.97 – 1.82 (m, 1H), 1.80 – 1.67 (m, 2H), 1.53 – 1.26 (m, 2H), 1.22 (t, *J* = 7.1 Hz, 3H). **^{13}C**

NMR (75 MHz, $CDCl_3$): δ 165.6, 144.9, 138.1, 126.4, 123.0, 110.3, 63.4, 60.8, 44.4, 33.1, 31.8, 23.2, 14.2. **HRMS** (EI) calcd. for $[C_{13}H_{18}Cl_2N_2O_4]^{\cdot+}$ ($[M]^{\cdot+}$), m/z = 304.0740; found 304.0734.

Ethyl (*E*)-4-(4-chloro-1H-pyrazol-1-yl)-8-cyanooct-2-enoate (3e):

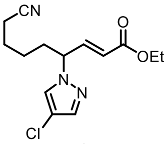

3e

According to general procedure A: Ethyl (*E*)-8-cyanooct-3-enoate (**1e**) (586 mg, 3.00 mmol, 3.00 equiv.), 4-chloro-1H-pyrazole (**2a**) (103 mg, 1.00 mmol, 1.00 equiv.), TAPT (24.3 mg, 50.0 µmol, 5 mol%), $(PhSe)_2$ (31.2 mg, 100 µmol, 10 mol%) and DCE (5 mL), 21 h. After purification (PE/EtOAc, 20:1 to 5:1), the product was isolated as a colorless oil (209 mg, 705 µmol, 71%; allylic/vinylic isomer >49:1). Crude ^{1}H NMR analysis: yield of allylic isomer is 75%; allylic/vinylic isomer: >9:1.

According to general procedure A: Ethyl (*E*)-8-cyanooct-3-enoate (**1e**) (195 mg, 1.00 mmol, 1.00 equiv.), 4-chloro-1H-pyrazole (**2a**) (308 mg, 3.00 mmol, 3.00 equiv.), TAPT (24.3 mg, 50.0 µmol, 5 mol%), $(PhSe)_2$ (31.2 mg, 100 µmol, 10 mol%) and DCE (5 mL), 21 h. Crude ^{1}H NMR analysis: total yield: 64%; allylic isomer: 60%; allylic/vinylic isomer: 15:1.

TLC: R_f = 0.44 (PE/EtOAc, 2:1) [UV, $KMnO_4$]. IR [cm^{-1}]: 3127, 2982, 2937, 2870, 2248, 1715, 1659, 1525, 1431, 1368, 1312, 1275, 1230, 1185, 1096, 1036, 969, 913, 842, 794, 731. **^{1}H NMR** (300 MHz, $CDCl_3$): δ 7.41 (d, *J* = 0.5 Hz, 1H), 7.38 (d, *J* = 0.7 Hz, 1H), 6.92 (dd, *J* = 15.6, 6.2 Hz, 1H), 5.65 (dd, *J* = 15.7, 1.4 Hz, 1H), 4.81 – 4.67 (m, 1H), 4.11 (q, *J* = 7.1 Hz, 2H), 2.32 – 2.20 (m, 2H), 2.15 – 1.99 (m, 1H), 1.97 – 1.80 (m, 1H), 1.69 – 1.49 (m, 2H), 1.46 – 1.17 (m, 5H). **^{13}C NMR** (75 MHz, $CDCl_3$): δ 165.5, 144.7, 138.2, 126.6, 123.1, 119.4, 110.3, 63.2, 60.8, 33.0, 25.0, 24.84, 17.0, 14.2. **HRMS** (EI) calcd. for $[C_{14}H_{18}ClN_3O_2]^{\cdot+}$ ($[M]^{\cdot+}$), m/z = 295.1082; found 295.1090.

Ethyl (*E*)-4-(4-chloro-1H-pyrazol-1-yl)-7-phenylhept-2-enoate (3f):

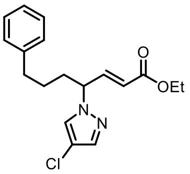

3f

According to general procedure A: Ethyl (*E*)-7-phenylhept-3-enoate (**1f**) (232 mg, 1.00 mmol, 1.00 equiv.), 4-chloro-1H-pyrazole (**2a**) (308 mg, 3.00 mmol, 3.00 equiv.), $(PhSe)_2$ (31.2 mg, 100 µmol, 10 mol%) and DCE (5 mL), 21 h. After purification (PE/EtOAc, 20:1 to 15:1), the product was isolated as a colorless oil (208 mg, 621 µmol, 62%; allylic:vinylic isomer 33:1). Crude ^{1}H NMR analysis: total yield: 68%; allylic isomer: 65%; allylic/vinylic isomer: 20:1.

TLC: R_f = 0.46 (PE/EtOAc, 5:1) [UV, $KMnO_4$]. IR [cm^{-1}]: 3127, 3027, 2982, 2937, 2863, 1715, 1659, 1495, 1435, 1368, 1312, 1271, 1230, 1182, 1096, 1036, 969, 842, 790, 749, 701. **^{1}H NMR** (300 MHz, $CDCl_3$): δ 7.47 (d, *J* = 0.5 Hz, 1H), 7.33 (d, *J* = 0.5 Hz, 1H), 7.31 – 7.23 (m, 2H), 7.23 – 7.09 (m, 3H), 6.98 (dd, *J* = 15.7, 6.1 Hz, 1H), 5.68 (dd, *J* = 15.6, 1.5 Hz, 1H), 4.84 – 4.73 (m,, 1H), 4.18 (q, *J* = 7.1 Hz, 2H), 2.62 (t, *J* = 7.5 Hz, 2H), 2.18 – 2.01 (m, 1H), 2.00 – 1.85 (m, 1H), 1.69 – 1.47 (m, 2H), 1.26 (t, *J* = 7.1 Hz, 3H). **^{13}C NMR** (75 MHz, $CDCl_3$): δ 165.7, 145.2, 141.3, 138.0, 128.5, 128.4, 126.3, 126.1, 122.8, 110.3, 63.6, 60.8, 35.2, 33.3, 27.5, 14.2. **HRMS** (ESI) calcd. for $[C_{18}H_{22}ClN_2O_2]^+$ ($[M+H]^+$), m/z = 333.1364; found 333.1370.

Ethyl (*E*)-7-((*tert*-butyldimethylsilyl)oxy)-4-(4-chloro-1H-pyrazol-1-yl)hept-2-enoate (3g):

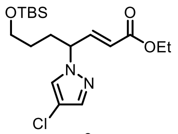

3g

According to general procedure A: Ethyl (*E*)-7-((*tert*-butyldimethylsilyl)oxy)hept-3-enoate (**1g**) (287 mg, 1.00 mmol, 1.00 equiv.), 4-chloro-1H-pyrazole (**2a**) (308 mg, 3.00 mmol, 3.00 equiv.), TAPT (24.3 mg, 50.0 µmol, 5 mol%), $(PhSe)_2$ (31.2 mg, 100 µmol, 10 mol%) and DCE (5 mL), 21 h. After purification (PE/EtOAc, 25:1 to 15:1), the product was isolated as a colorless oil (282 mg, 729 µmol, 73% allylic/vinylic isomer 25:1). Crude ^{1}H NMR analysis: total yield: 78%; allylic isomer: 73%, allylic/vinylic isomer: 17:1.

TLC: R_f = 0.65 (PE/EtOAc, 5:1) [UV, $KMnO_4$]. IR [cm^{-1}]: 3131, 2952, 2896, 2859, 1722, 1662, 1469, 1435, 1387, 1312, 1256, 1178, 1096, 1036, 969, 835, 775, 716. **^{1}H NMR** (300 MHz, $CDCl_3$): δ 7.43 (d, *J* = 0.4 Hz, 1H), 7.39 (d, *J* = 0.7 Hz, 1H), 6.98 (dd, *J* = 15.7, 6.0 Hz, 1H), 5.66 (dd, *J* = 15.7, 1.6 Hz, 1H), 4.92 – 4.75 (m, 1H), 4.14 (q, *J* = 7.1 Hz, 2H), 3.62 – 3.52 (m,

2H), 2.15 – 1.93 (m, 2H), 1.50 – 1.32 (m, 2H), 1.23 (t, *J* = 7.1 Hz, 3H), 0.85 (s, 9H), -0.00 (s, 6H). **^{13}C NMR** (75 MHz, $CDCl_3$): δ 165.7, 145.4, 138.0, 126.3, 122.7, 110.2, 63.4, 62.1, 60.7, 30.5, 28.8, 25.9, 18.3, 14.2, -5.4. **HRMS** (ESI) calcd. for $[C_{18}H_{32}ClN_2O_3]^+$ ($[M+H]^+$), m/z = 387.1865; found 387.187301.0953.

Ethyl (*E*)-4-(4-chloro-1H-pyrazol-1-yl)-7-((tetrahydro-2H-pyran-2-yl)oxy)hept-2-enoate (3h):

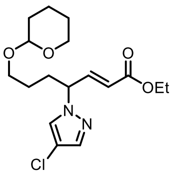

3h

According to general procedure A: Ethyl (*E*)-7-((tetrahydro-2H-pyran-2-yl)oxy)hept-3-enoate (**1h**) (256 mg, 1.00 mmol, 1.00 equiv.), 4-chloro-1H-pyrazole (**2a**) (308 mg, 3.00 mmol, 3.00 equiv.), TAPT (24.3 mg, 50.0 µmol, 5 mol%), $(PhSe)_2$ (31.2 mg, 100 µmol, 10 mol%) and DCE (5 mL), 21 h. After purification (DCM/CH_3CN, 10:1 to 5:1), the product was isolated as a colorless oil (192 mg, 538 µmol, 54%; allylic:vinylic isomer 17:1). Crude ^{1}H NMR analysis: total yield: 52%; allylic isomer: 50%, allylic/vinylic isomer: 25:1.

According to general procedure A: Ethyl (*E*)-7-((tetrahydro-2H-pyran-2-yl)oxy)hept-3-enoate (**1h**) (769 mg, 3.00 mmol, 3.00 equiv.), 4-chloro-1H-pyrazole (**2a**) (103 mg, 1.00 mmol, 1.00 equiv.), TAPT (24.3 mg, 50.0 µmol, 5 mol%), $(PhSe)_2$ (31.2 mg, 100 µmol, 10 mol%) and DCE (5 mL), 21 h. Crude ^{1}H NMR analysis: yield of allylic isomer: 30%; the signal of vinylic isomer is overlapping with others.

TLC: R_f = 0.49 (PE/EtOAc, 2:1) [UV, $KMnO_4$]. IR [cm^{-1}]: 3124, 2941, 2870, 1718, 1659, 1525, 1439, 1368, 1312, 1271, 1178, 1118, 1074, 1033, 969, 902, 869, 734. **^{1}H NMR** (300 MHz, $CDCl_3$): δ 7.41 (s, 1H), 7.39 (s, 1H), 6.96 (ddd, *J* = 15.6, 6.0, 0.7 Hz, 1H), 5.66 (dd, *J* = 15.8, 1.5 Hz, 1H), 4.91 – 4.77 (m, 1H), 4.48 (dd, *J* = 7.0, 3.6 Hz, 1H), 4.12 (q, *J* = 7.1 Hz, 2H), 3.82 – 3.63 (m, 2H), 3.49 – 3.39 (m, 1H), 3.38 – 3.25 (m, 1H), 2.18 – 1.93 (m, 2H), 1.80 – 1.61 (m, 2H), 1.55 – 1.40 (m, 6H), 1.21 (t, *J* = 7.1 Hz, 3H). **^{13}C NMR** (75 MHz, $CDCl_3$): δ 165.7, 145.3, 138.0, 126.4, 122.8, 110.2, 110.2, 99.0, 66.6, 66.5, 63.4, 63.4, 62.5, 62.4, 60.7, 31.0, 30.8, 30.7, 26.0, 25.4, 19.7, 19.6, 14.2. **HRMS** (ESI) calcd. for $[C_{17}H_{26}ClN_2O_4]^+$ ($[M+H]^+$), m/z = 357.1576; found 357.1578.

Ethyl (*E*)-6-(benzyloxy)-4-(4-chloro-1H-pyrazol-1-yl)hex-2-enoate (3i):

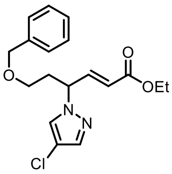

3i

According to general procedure A: Ethyl (*E*)-6-(benzyloxy)hex-3-enoate (**1i**) (248 mg, 1.00 mmol, 1.00 equiv.), 4-chloro-1H-pyrazole (**2a**) (308 mg, 3.00 mmol, 3.00 equiv.), TAPT (24.3 mg, 50.0 µmol, 5 mol%), $(PhSe)_2$ (31.2 mg, 100 µmol, 10 mol%) and DCE (5 mL), 21 h. After purification (PE/EtOAc, 20:1 to 15:1), the product was isolated as a lightly yellow oil (191 mg, 549 µmol, 55%; allylic/vinylic isomer 13:1). Crude ^{1}H NMR analysis: total yield: 64%; allylic isomer: 56%; allylic/vinylic isomer: 7:1.

According to general procedure A: Ethyl (*E*)-6-(benzyloxy)hex-3-enoate (**1i**) (248 mg, 1.00 mmol, 1.00 equiv.), 4-chloro-1H-pyrazole (**2a**) (308 mg, 3.00 mmol, 3.00 equiv.), TAPT (24.3 mg, 50.0 µmol, 5 mol%), $(PhSe)_2$ (31.2 mg, 100 µmol, 10 mol%), $(4\text{-}ClPhS)_2$ (14.4 mg, 50.0 µmol, 5 mol%) and DCE (5 mL), 21 h. Crude ^{1}H NMR analysis: total yield: 50%; allylic isomer: 47%; allylic/vinylic isomer: 14:1.

TLC: R_f = 0.41 (PE/EtOAc, 5:1) [UV, $KMnO_4$]. IR [cm^{-1}]: 3131, 2982, 2863, 1718, 1659, 1454, 1368, 1312, 1267, 1226, 1179, 1096, 1029, 969, 842, 798, 738, 701. **^{1}H NMR** (300 MHz, $CDCl_3$): δ 7.47 (s, 1H), 7.39 – 7.24 (m, 6H), 7.02 (dd, *J* = 15.6, 6.0 Hz, 1H), 5.69 (dd, *J* = 15.8, 1.5 Hz, 1H), 5.14 – 5.02 (m, 1H), 4.48 – 4.34 (m, 2H), 4.17 (q, *J* = 7.1 Hz, 2H), 3.47 (dt, *J* = 9.7, 4.9 Hz, 1H), 3.17 (td, *J* = 9.3, 4.0 Hz, 1H), 2.39 – 2.27 (m, 1H), 2.27 – 2.14 (m, 1H), 1.26 (t, *J* = 7.1 Hz, 3H). **^{13}C NMR** (75 MHz, $CDCl_3$): δ 165.7, 145.2, 138.3, 137.9, 128.5, 127.9, 127.3, 122.9, 109.9, 73.2, 65.3, 60.7, 60.0, 34.0, 14.2. **HRMS** (ESI) calcd. for $[C_{18}H_{22}ClN_2O_3]^+$ ($[M+H]^+$), m/z = 349.1313; found 349.1321.

Ethyl (*E*)-4-(4-chloro-1H-pyrazol-1-yl)-5-(4-fluorophenyl)pent-2-enoate (3j):

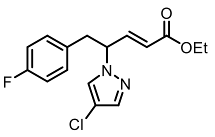

3j

According to general procedure A: Ethyl (*E*)-5-(4-fluorophenyl)pent-3-enoate (**1j**) (222 mg, 1.00 mmol, 1.00 equiv.), 4-chloro-1H-pyrazole (**2a**) (308 mg, 3.00 mmol, 3.00 equiv.), TAPT (24.3 mg, 50.0 µmol, 5 mol%), $(PhSe)_2$ (31.2 mg, 100 µmol, 10 mol%), $(4\text{-}ClPhS)_2$ and DCE (5.0 mL), 21 h. After purification (PE/EtOAc, 30:1 to 15:1), the product was isolated as a colorless oil (171 mg, 53.0 µmol, 53%, *E*/*Z* = 33:1; allylic/vinylic isomer >49:1). Crude ^{1}H NMR analysis: yield of allylic isomer: 52%, *E*/*Z* = 8:1, allylic/vinylic isomer: >10:1.

According to general procedure A: Ethyl (*E*)-5-(4-fluorophenyl)pent-3-enoate (**1j**) (222 mg, 1.00 mmol, 1.00 equiv.), 4-chloro-1H-pyrazole (**2a**) (308 mg, 3.00 mmol, 3.00 equiv.), TAPT (24.3 mg, 50.0 µmol, 5 mol%), $(PhSe)_2$ (31.2 mg, 100 µmol, 10 mol%) and DCE (5 mL), 21 h. Crude ^{1}H NMR analysis: yield of allylic isomer: 37%, *E*/*Z* = 13:1; allylic/vinylic isomer: >10:1.

TLC: R_f = 0.44 (PE/EtOAc, 5:1) [UV, $KMnO_4$]. IR [cm^{-1}]:3130, 2982, 1715, 1659, 1603, 1435, 1312, 1267, 1223, 1182, 1096, 1036, 969, 924, 835, 734. **^{1}H NMR** (400 MHz, $CDCl_3$, *E*-isomer): δ 7.53 (s, 1H), 7.26 (s, 1H), 7.12 (dd, *J* = 15.6, 6.1 Hz, 1H), 7.05 – 6.90 (m, 4H), 5.77 (dd, *J* = 15.8, 1.5 Hz, 1H), 5.04 – 4.93 (m, 1H), 4.23 (q, *J* = 7.2 Hz, 2H), 3.43 (dd, *J* = 13.9, 9.2 Hz, 1H), 3.21 (dd, *J* = 14.0, 5.8 Hz, 1H), 1.31 (t, *J* = 7.2 Hz, 3H). **^{13}C NMR** (101 MHz, $CDCl_3$, *E*-isomer): δ 165.5, 161.9 (d, $J_{C\text{-}F}$ = 245.8 Hz), 144.1, 138.4, 131.9 (d, $J_{C\text{-}F}$ = 3.0 Hz), 130.5 (d, $J_{C\text{-}F}$ = 7.8 Hz), 127.1, 123.5, 115.5 (d, $J_{C\text{-}F}$ = 21.7 Hz), 110.1, 65.1 (d, $J_{C\text{-}F}$ = 0.9 Hz), 60.8, 39.5, 14.2. **^{19}F NMR** (377 MHz, $CDCl_3$, *E*- and *Z*-isomer) δ -110.9, -115.8. **HRMS** (EI) calcd. for $[C_{16}H_{16}N_2O_2FCl]^{\cdot+}$ ($[M]^{\cdot+}$), m/z = 322.0879; found 322.0875.

1-Ethyl 8-methyl (*E*)-4-(4-chloro-1H-pyrazol-1-yl)oct-2-enedioate (3k):

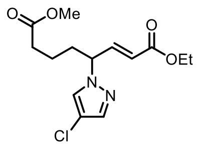

3k

According to general procedure A: 1-Ethyl 8-methyl (*E*)-oct-3-enedioate (**1k**) (643 mg, 3.00 mmol, 3.00 equiv.), 4-chloro-1H-pyrazole (**2a**) (103 mg, 1.00 mmol, 1.00 equiv.), TAPT (24.3 mg, 50.0 µmol, 5 mol%), $(PhSe)_2$ (31.2 mg, 100 µmol, 10 mol%) and DCE (5 mL), 21 h. After purification (PE/EtOAc, 20:1-6:1), the product was isolated as a colorless oil (263 mg, 835 µmol, 84%; allylic/vinylic isomer 14:1). Crude ^{1}H NMR analysis: total yield: 93%; allylic isomer: 84%; allylic/vinylic isomer: 10:1.

According to general procedure A: 1-Ethyl 8-methyl (*E*)-oct-3-enedioate (**1k**) (214 mg, 1.00 mmol, 1.00 equiv.), 4-chloro-1H-pyrazole (**2a**) (308 mg, 3.00 mmol, 3.00 equiv.), TAPT (24.3 mg, 50.0 µmol, 5 mol%), $(PhSe)_2$ (31.2 mg, 100 µmol, 10 mol%) and DCE (5 mL), 21 h. Crude ^{1}H NMR analysis: total yield: 54%; allylic isomer: 50%; allylic/vinylic isomer: 11:1.

TLC: R_f = 0.14 (PE/EtOAc, 5:1) [UV, $KMnO_4$]. IR [cm^{-1}]: 3131, 2952, 1722, 1659, 1525, 1435, 1368, 1312, 1267, 1174, 1036, 969, 842, 798, 734. **^{1}H NMR** (300 MHz, $CDCl_3$): δ 7.39 (s, 2H), 6.92 (dd, *J* = 15.7, 6.1 Hz, 1H), 5.64 (dd, *J* = 15.7, 1.4 Hz, 1H), 4.87 – 4.63 (m, 1H), 4.10 (q, *J* = 7.1 Hz, 2H), 3.58 (s, 3H), 2.26 (t, *J* = 7.3 Hz, 2H), 2.14 – 1.97 (m, 1H), 1.97 – 1.82 (m, 1H), 1.63 – 1.36 (m, 2H), 1.19 (t, *J* = 7.1 Hz, 3H). **^{13}C NMR** (75 MHz, $CDCl_3$): δ 173.2, 165.5, 144.9, 138.0, 126.5, 122.9, 110.2, 63.2, 60.7, 51.6, 33.0, 21.2, 14.1. **HRMS** (ESI) calcd. for $[C_{14}H_{20}ClN_2O_4]^+$ ($[M+H]^+$), m/z = 315.1106; found 315.1110.

Dimethyl (*E*)-4-(4-chloro-1H-pyrazol-1-yl)hex-2-enedioate (3l):

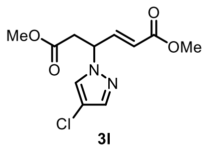

3l

According to general procedure A: Dimethyl (*E*)-hex-3-enedioate (**1l**) (517 mg, 3.00 mmol, 3.00 equiv.), 4-chloro-1H-pyrazole (**2a**) (103 mg, 1.00 mmol, 1.00 equiv.), TAPT (24.3 mg, 50.0 µmol, 5 mol%), $(PhSe)_2$ (31.2 mg, 100 µmol, 10 mol%) and DCE (5 mL), 21 h. After purification (PE/EtOAc, 10:1 to 5:1), the product was isolated as a lightly yellow oil (283 mg, 1.04 mmol, >99%; allylic/vinylic isomer >49:1). Crude ^{1}H NMR analysis: yield of allylic isomer: 97%; allylic/vinylic isomer: >10:1.

TLC: R_f = 0.16 (PE/EtOAc, 5:1) [UV, $KMnO_4$]. IR [cm^{-1}]: 3131, 3001, 2956, 1722, 1662, 1439, 1368, 1312, 1275, 1200, 1159, 1036, 969, 917, 846, 798, 731. **^{1}H NMR** (300 MHz, $CDCl_3$): δ 7.45 (s, 2H), 7.00 (dd, *J* = 15.7, 6.0 Hz, 1H), 5.70 (dd, *J* = 15.6, 1.5 Hz, 1H), 5.38 – 5.24 (m, 1H), 3.70 (s, 3H), 3.65 (s, 3H), 3.23 (dd, *J* = 16.8, 8.6 Hz, 1H), 2.91 (dd, *J* = 16.7, 5.6 Hz, 1H). **^{13}C NMR** (75 MHz, $CDCl_3$): δ 170.0, 165.8, 143.9, 138.6, 127.5, 123.2, 110.4, 59.2, 52.3, 51.9, 37.9. **HRMS** (ESI) calcd. for $[C_{11}H_{14}ClN_2O_4]^+$ ($[M+H]^+$), m/z = 273.0637; found 273.0640.

Diisopropyl (*E*)-4-(4-chloro-1H-pyrazol-1-yl)hex-2-enedioate (3m):

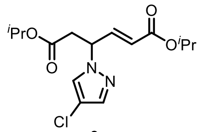

3m

According to general procedure A: Diisopropyl (E)-hex-3-enedioate (**1m**) (865 mg, 3.00 mmol, 3.00 equiv.), 4-chloro-1H-pyrazole (**2a**) (103 mg, 1.00 mmol, 1.00 equiv.), TAPT (24.3 mg, 50.0 µmol, 5 mol%), $(PhSe)_2$ (31.2 mg, 100 µmol, 10 mol%) and DCE (5 mL), 21 h. After purification (PE/EtOAc, 20:1 to 10:1), the product was isolated as a lightly yellow oil (303 mg, 922 µmol, 92%; allylic/vinylic isomer >49:1). Crude ^{1}H NMR analysis: yield of allylic isomer: 93%; allylic/vinylic isomer: >10:1.

TLC: R_f = 0.33 (PE/EtOAc, 5:1) [UV, $KMnO_4$]. IR [cm^{-1}]: 3131, 2982, 2937, 1715, 1662, 1528, 1469, 1375, 1308, 1275, 1178, 1103, 969, 910, 824, 734. **^{1}H NMR** (300 MHz, $CDCl_3$): δ 7.49 – 7.37 (m, 2H), 6.95 (dd, *J* = 15.6, 5.9 Hz, 1H), 5.66 (dd, *J* = 15.7, 1.6 Hz, 1H), 5.37 – 5.21 (m, 1H), 5.12 – 4.80 (m, 2H), 3.14 (dd, *J* = 16.3, 8.8 Hz, 1H), 2.85 (dd, *J* = 16.4, 5.7 Hz, 1H), 1.20 (d, *J* = 6.3 Hz, 6H), 1.14 (t, *J* = 6.5 Hz, 6H). **^{13}C NMR** (75 MHz, $CDCl_3$): δ 168.9, 164.9, 143.3, 138.4, 127.4, 124.0, 110.3, 68.8, 68.3, 59.4, 38.6, 21.8, 21.6. **HRMS** (ESI) calcd. for $[C_{15}H_{22}ClN_2O_4]^+$ ($[M+H]^+$), m/z = 329.1263; found 329.1269.

Dibenzyl (*E*)-4-(4-chloro-1H-pyrazol-1-yl)hex-2-enedioate (3n):

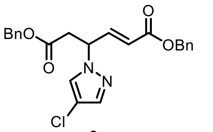

3n

According to general procedure A: Dibenzyl (*E*)-hex-3-enedioate (**1n**) (973 mg, 3.00 mmol, 3.00 equiv.), 4-chloro-1H-pyrazole (**2a**) (103 mg, 1.00 mmol, 1.00 equiv.), TAPT (24.3 mg, 50.0 µmol, 5 mol%), $(PhSe)_2$ (31.2 mg, 100 µmol, 10 mol%) and DCE (5mL), 21 h. After purification (PE/EtOAc, 30:1 to 15:1), the product was isolated as a colorless solid (376 mg, 885 µmol, 89%; allylic/vinylic isomer >49:1). Crude ^{1}H NMR analysis: yield of allylic isomer: 92%; allylic/vinylic isomer >10:1.

TLC: R_f = 0.25 (PE/EtOAc, 5:1) [UV, $KMnO_4$]. **Mp** 67.9 °C. IR [cm^{-1}]: 3142, 2945, 1707, 1655, 1498, 1439, 1383, 1245, 1193, 1148, 988, 850, 798, 731. **^{1}H NMR** (300 MHz, $CDCl_3$): δ 7.46 (s, 1H), 7.40 (d, *J* = 0.4 Hz, 1H), 7.39 – 7.22 (m, 10H), 7.05 (dd, *J* = 15.6, 5.9 Hz, 1H), 5.75 (dd, *J* = 15.6, 1.5 Hz, 1H), 5.38 – 5.29 (m, 1H), 5.20 – 5.05 (m, 4H), 3.30 (dd, *J* = 16.6, 8.9 Hz, 1H), 2.95 (dd, *J* = 16.6, 5.5 Hz, 1H). **^{13}C NMR** (75 MHz, $CDCl_3$): δ 169.3, 165.2, 144.2, 138.6, 135.5, 135.2, 128.7, 128.5, 128.3, 127.5, 123.3, 110.5, 67.1, 66.8, 59.3, 38.1. **HRMS** (ESI) calcd. for $[C_{23}H_{22}ClN_2O_4]^+$ ($[M+H]^+$), m/z = 425.1263; found 425.1266.

Dicyclohexyl (*E*)-4-(4-chloro-1H-pyrazol-1-yl)hex-2-enedioate (3o):

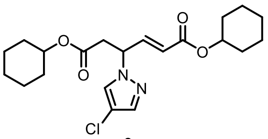

3o

According to general procedure A: Dicyclohexyl (*E*)-hex-3-enedioate (**1o**) (925 mg, 3.00 mmol, 3.00 equiv.), 4-chloro-1H-pyrazole (**2a**) (103 mg, 1.00 mmol, 1.00 equiv.), TAPT (24.3 mg, 50.0 µmol, 5 mol%), $(PhSe)_2$ (31.2 mg, 100 µmol, 10 mol%) and DCE (5 mL), 21 h. After purification (PE/EtOAc, 30:1 to 15:1), the product was isolated as a colourless solid (392 mg, 958 µmol, 96%; allylic/vinylic isomer >49:1). Crude ^{1}H NMR analysis: yield of allylic isomer: 92%; allylic/vinylic isomer: >10:1.

TLC: R_f = 0.36 (PE/EtOAc, 5:1) [UV, $KMnO_4$]. **Mp** 82.5 °C. IR [cm^{-1}]: 3109, 2937, 2859, 1711, 1662, 1454, 1379, 1267, 1230, 1170, 1144, 1010, 965, 932, 887, 768, 727. **^{1}H NMR** (300 MHz, $CDCl_3$): δ 7.43 (s, 2H), 6.96 (dd, *J* = 15.6, 5.9 Hz, 1H), 5.68 (dd, *J* = 15.6, 1.5 Hz, 1H), 5.41 – 5.16 (m, 1H), 4.84 – 4.61 (m, 2H), 3.26 – 3.07 (m, 1H), 2.88 (dd, *J* = 16.3, 5.8 Hz, 1H), 1.90 – 1.56 (m, 8H), 1.56 – 1.42 (m, 2H), 1.41 – 1.11 (m, 10H). **^{13}C NMR** (75 MHz, $CDCl_3$): δ 168.9, 164.9, 143.3, 138.4, 127.3, 124.1, 110.3, 73.7, 73.2, 59.5, 38.6, 31.5, 31.4, 25.3, 25.2, 23.7, 23.6. **HRMS** (ESI) calcd. for $[C_{21}H_{30}ClN_2O_4]^+$ ($[M+H]^+$), m/z = 409.1889; found 409.1892.

Diphenyl (*E*)-4-(4-chloro-1H-pyrazol-1-yl)hex-2-enedioate (3p):

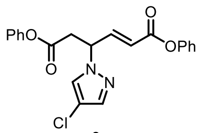

3p

According to general procedure A: Diphenyl (*E*)-hex-3-enedioate (**1p**) (889 mg, 3.00 mmol, 3.00 equiv.), 4-chloro-1H-pyrazole (**2a**) (103 mg, 1.00 mmol, 1.00 equiv.), TAPT (24.3 mg, 50.0 μmol, 5 mol%), $(PhSe)_2$ (31.2 mg, 100 μmol, 10 mol%) and DCE (5 mL), 21 h. After purification (PE/EtOAc, 30:1 to 15:1), the product was isolated as a colorless solid (321 mg, 808 μmol, 81%; allylic/vinylic isomer >49:1). Crude ^{1}H NMR analysis: yield of allylic isomer: 90%; allylic/vinylic isomer: >10:1.

TLC: R_f = 0.25 (PE/EtOAc, 5:1) [UV, $KMnO_4$]. **Mp** 115.0 °C. IR [cm^{-1}]: 3120, 3049, 1737, 1655, 1592, 1484, 1416, 1364, 1301, 1271, 1189, 1144, 882, 965, 936, 867, 686. **^{1}H NMR** (300 MHz, $CDCl_3$): δ 7.52 (s, 1H), 7.49 (d, *J* = 0.5 Hz, 1H), 7.40 – 7.28 (m, 4H), 7.25 – 7.15 (m, 3H), 7.11 – 7.03 (m, 2H), 7.00 – 6.93 (m, 2H), 5.93 (dd, *J* = 15.7, 1.6 Hz, 1H), 5.51 – 5.36 (m, 1H), 3.51 (dd, *J* = 16.8, 9.0 Hz, 1H), 3.18 (dd, *J* = 16.8, 5.4 Hz, 1H). **^{13}C NMR** (75 MHz, $CDCl_3$): δ 168.2, 163.8, 150.4, 150.2, 145.4, 138.9, 129.6, 127.8, 126.3, 126.1, 123.2, 121.4, 121.3, 110.8, 59.2, 38.2. **HRMS** (ESI) calcd. for $[C_{21}H_{18}ClN_2O_4]^+$ ($[M+H]^+$), m/z = 397.0950; found 397.0956.

Diheptyl (*E*)-4-(4-chloro-1H-pyrazol-1-yl)hex-2-enedioate (3q):

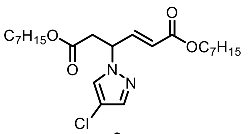

3q

According to general procedure A: Diheptyl (*E*)-hex-3-enedioate (**1q**) (1.02 g, 3.00 mmol, 3.00 equiv.), 4-chloro-1H-pyrazole (**2a**) (103 mg, 1.00 mmol, 1.00 equiv.), TAPT (24.3 mg, 50.0 μmol, 5 mol%), $(PhSe)_2$ (31.2 mg, 100 μmol, 10 mol%) and DCE (5 mL), 21 h. After purification (PE/EtOAc, 30:1 to 15:1), the product was isolated as a lightly yellow oil (409 mg, 927 μmol, 93%; allylic/vinylic isomer >49:1). Crude ^{1}H NMR analysis: yield of allylic isomer: 98%; allylic/vinylic isomer: >10:1.

TLC: R_f = 0.57 (PE/EtOAc, 5:1) [UV, $KMnO_4$]. IR [cm^{-1}]: 3131, 2930, 2859, 1722, 1662, 1465, 1387, 1361, 1312, 1271, 1174, 1066, 969, 842, 790, 734. **^{1}H NMR** (300 MHz, $CDCl_3$): δ 7.44 (s, 2H), 6.98 (dd, *J* = 15.8, 5.9 Hz, 1H), 5.69 (dd, *J* = 15.7, 1.6 Hz, 1H), 5.38 – 5.23 (m, 1H), 4.08 (t, *J* = 6.7 Hz, 2H), 4.02 (t, *J* = 6.7 Hz, 2H), 3.20 (dd, *J* = 16.5, 8.9 Hz, 1H), 2.89 (dd, *J* = 16.6, 5.6 Hz, 1H), 1.66 – 1.47 (m, 4H), 1.33 – 1.18 (m, 16H), 0.84 (dt, *J* = 6.7, 4.3 Hz, 6H). **^{13}C NMR** (75 MHz, $CDCl_3$): δ 169.5, 165.5, 143.6, 138.5, 127.4, 123.5, 110.3, 65.4, 65.1, 59.3, 38.2, 31.7, 28.9, 28.8, 28.5, 25.8, 25.7, 22.6, 14.0. **HRMS** (ESI) calcd. for $[C_{23}H_{38}ClN_2O_4]^+$ ($[M+H]^+$), m/z = 441.2515; found 441.2518.

1-((1R,2S,5R)-2-isopropyl-5-methylcyclohexyl) 6-((1S,2R,5S)-2-isopropyl-5-methylcyclohexyl) (*E*)-4-(4-chloro-1H-pyrazol-1-yl)hex-2-enedioate (3r):

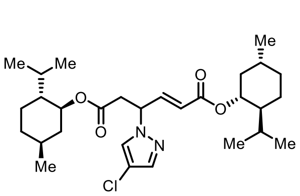

3r

According to general procedure A: 1-((1R,2S,5R)-2-Isopropyl-5-methylcyclohexyl) 6-((1S,2R,5S)-2-isopropyl-5-methylcyclohexyl) (*E*)-hex-3-enedioate (**1r**) (1.26 g, 3.00 mmol, 3.00 equiv.), 4-chloro-1H-pyrazole (**2a**) (103 mg, 1.00 mmol, 1.00 equiv.), TAPT (24.3 mg, 50.0 μmol, 5 mol%), $(PhSe)_2$ (31.2 mg, 100 μmol, 10 mol%) and DCE (5 mL), 21 h. After purification (PE/EtOAc, 60:1 to 40:1), the product was isolated as a colorless solid (474 mg, 910 μmol, 91%; in which allylic/vinylic isomer >49:1). Crude ^{1}H NMR analysis: yield of allylic isomer: 93%; allylic/vinylic isomer >10:1.

TLC: R_f = 0.41 (PE/EtOAc, 10:1) [UV, $KMnO_4$]. **Mp** 117.1 °C. IR [cm^{-1}]: 3124, 2956, 2866, 1730, 1659, 1457, 1371, 1267, 1234, 1190, 1152, 1103, 1040, 984, 876, 842, 738. **^{1}H NMR** (300 MHz, $CDCl_3$): δ 7.44 (dd, *J* = 5.2, 2.2 Hz, 2H), 6.99 (dd, *J* = 5.8, 4.2 Hz, 0.47H), 6.94 (dd, *J* = 5.8, 4.2 Hz, 0.52H), 5.75 – 5.61 (m, 1H), 5.40 – 5.24 (m, 1H), 4.81 – 4.53 (m, 2H), 3.26 – 3.10 (m, 1H), 2.95 – 2.79 (m, 1H), 2.00 – 1.90 (m, 1H), 1.89 – 1.74 (m, 2H), 1.70 – 1.22 (m, 9H+peak of water), 1.06 – 0.91 (m, 3H), 0.90 – 0.77 (m, 15H), 0.73 – 0.69 (m, 3H), 0.68 – 0.55 (m, 3H). **^{13}C NMR** (75 MHz, $CDCl_3$): δ 169.1, 169.0, 165.0,

143.4, 138.5, 138.4, 127.5, 127.3, 123.9, 123.8, 110.36, 110.2, 75.3, 75.2, 74.7, 59.7, 59.5, 47.0, 46.9, 46.8, 40.8, 40.7, 40.6, 38.7, 38.6, 34.2, 34.1, 31.4, 31.3, 26.1, 26.0, 23.4, 23.3, 23.2, 22.0, 20.8, 20.7, 16.3, 16.2, 16.1, 16.0. **HRMS** (ESI) calcd. for $[C_{29}H_{46}ClN_2O_4]^+$ ($[M+H]^+$), m/z = 521.3141; found 521.315.

Benzyl (*E*)-4-(4-chloro-1H-pyrazol-1-yl)hex-2-enoate (3s):

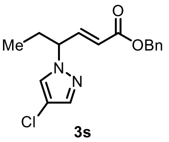

3s

According to general procedure A: Benzyl (*E*)-hex-3-enoate (**1s**) (204 mg, 1.00 mmol, 1.00 equiv.), 4-chloro-1H-pyrazole (**2a**) (308 mg, 3.00 mmol, 3.00 equiv.), TAPT (22.5 mg, 46.2 µmol, 5 mol%), $(PhSe)_2$ (31.2 mg, 100 µmol, 10 mol%) and DCE (5 mL), 21 h. After purification (PE/EtOAc, 9:1), the product was isolated as a colorless oil (185 mg, 608 µmol, 61%; allylic/vinylic isomer 20:1). Crude ^{1}H NMR analysis: total yield is 59%, allylic isomer: 55%, allylic/vinylic isomer: 16:1.

TLC: R_f = 0.23 (PE/EtOAc, 4:1) [UV, $KMnO_4$]. IR [cm^{-1}]: 3131, 3064, 3034, 2971, 2878, 1718, 1659, 1457, 1383, 1271, 1163, 969, 842, 697. **^{1}H NMR** (300 MHz, $CDCl_3$): δ 7.47 (s, 1H), 7.39 (s, 1H), 7.38 – 7.30 (m, 5H), 7.06 (dd, *J* = 15.7, 6.0 Hz, 1H), 5.75 (dd, *J* = 15.6, 1.5 Hz, 1H), 5.17 (s, 2H), 4.77 – 4.62 (m, 1H), 2.17 – 1.83 (m, 2H), 0.88 (t, *J* = 7.3 Hz, 3H). **^{13}C NMR** (75 MHz, $CDCl_3$): δ 165.5, 145.8, 138.1, 135.6, 128.6, 128.6, 128.4, 128.3, 126.3, 122.5, 110.3, 66.6, 65.2, 27.2, 10.5. **HRMS** (EI) calcd. for $[C_{16}H_{17}ClN_2O_2]^{\cdot+}$ ($[M]^{\cdot+}$), m/z = 304.0973; found 304.0968.

Phenyl (*E*)-4-(4-chloro-1H-pyrazol-1-yl)hex-2-enoate (3t):

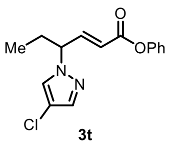

3t

According to general procedure A: Phenyl (*E*)-hex-3-enoate (**1t**) (190 mg, 1.00 mmol, 1.00 equiv.), 4-chloro-1H-pyrazole (**2a**) (308 mg, 3.00 mmol, 3.00 equiv.), TAPT (24.4 mg, 50.1 µmol, 5 mol%), $(PhSe)_2$ (31.2 mg, 100 µmol, 10 mol%) and DCE (5 mL), 21 h. After purification (PE/EtOAc, 95:5), the product was isolated as a lightly yellow oil (179 mg, 616 µmol, 62%; allylic/vinylic isomer >49:1). Crude ^{1}H NMR analysis: total yield: 65%; allylic isomer: 58%; allylic/vinylic isomer: 8:1.

TLC: R_f = 0.56 (PE/EtOAc, 4:1) [UV, $KMnO_4$]. IR [cm^{-1}]: 3131, 2974, 2937, 1737, 1656, 1595, 1490, 1312, 1193, 1163, 973, 824, 690. **^{1}H NMR** (400 MHz, $CDCl_3$):δ 7.52 (s, 1H), 7.46 (s, 1H), 7.41 – 7.35 (m, 2H), 7.26 – 7.17 (m, 2H), 7.12 – 7.06 (m, 2H), 5.90 (dd, *J* = 15.7, 1.6 Hz, 1H), 4.80 (dtd, *J* = 7.3, 5.9, 1.3 Hz, 1H), 2.22 – 2.09 (m, 1H), 2.09 – 1.97 (m, 1H), 0.95 (t, *J* = 7.3 Hz, 3H). **^{13}C NMR** (101 MHz, $CDCl_3$): δ 164.1, 150.5, 147.3, 138.2, 129.5, 126.4, 126.0, 122.1, 121.5, 110.4, 65.2, 27.2, 10.6. **HRMS** (ESI) calcd. for $[C_{15}H_{15}ClN_2O_4]^+$ ($[M+H]^+$), m/z = 291.0895; found 291.0895.

Cyclopropylmethyl (*E*)-4-(4-chloro-1H-pyrazol-1-yl)hex-2-enoate (3u):

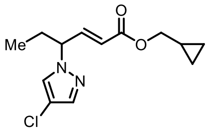

3u

According to general procedure A: Cyclopropylmethyl (*E*)-hex-3-enoate (**1u**) (505 mg, 3.00 mmol, 3.00 equiv.), 4-chloro-1H-pyrazole (**2a**) (103 mg, 1.00 mmol, 1.00 equiv.), TAPT (24.3 mg, 50.0 µmol, 5 mol%), $(PhSe)_2$ (31.2 mg, 100 µmol, 10 mol%) and DCE (5 mL), 21 h. After purification (PE/EtOAc, 40:1 to 15:1), the product was isolated as a colorless oil (201 mg, 746 µmol, 75%; allylic/vinylic isomer 33:1). Crude ^{1}H NMR analysis: total yield: 82%; allylic isomer: 72%; allylic/vinylic isomer: 7:1.

According to general procedure A: Cyclopropylmethyl (*E*)-hex-3-enoate (**1u**) (505 mg, 3.00 mmol, 3.00 equiv.), 4-chloro-1H-pyrazole (**2a**) (103 mg, 1.00 mmol, 1.00 equiv.), TAPT (24.3 mg, 50.0 µmol, 5 mol%), $(PhSe)_2$ (31.2 mg, 100 µmol, 10 mol%), $(4\text{-}ClPhS)_2$ (14.4 mg, 50.0 µmol, 5 mol%) and DCE (5 mL), 21 h. Crude ^{1}H NMR analysis: total yield: 76%; allylic isomer: 70%, allylic/vinylic isomer: 13:1.

TLC: R_f = 0.42 (PE/EtOAc, 5:1) [UV, $KMnO_4$]. IR [cm^{-1}]: 3086, 2971, 2881, 1715, 1659, 1461, 1431, 1349, 1308, 1274, 1170, 1025, 969, 828, 790, 716. **^{1}H NMR** (400 MHz, $CDCl_3$): δ 7.45 (s, 1H), 7.39 (s, 1H), 7.00 (dd, *J* = 15.6, 6.1 Hz, 1H), 5.72 (dd, *J* = 15.7, 1.6 Hz, 1H), 4.76 – 4.65 (m, 1H), 3.93 (d, *J* = 7.3 Hz, 2H), 2.13 – 1.90 (m, 2H), 1.16 – 1.05 (m, 1H), 0.88

(t, J = 7.3 Hz, 3H), 0.57 – 0.51 (m, 2H), 0.28 – 0.22 (m, 2H). **^{13}C NMR** (101 MHz, $CDCl_3$): δ 165.8, 145.2, 138.0, 126.3, 122.9, 110.2, 69.6, 65.2, 27.2, 10.5, 9.7, 3.3. **HRMS** (EI) calcd. for $[C_{13}H_{17}ClN_2O_2]^{\cdot+}$ ($[M]^{\cdot+}$), m/z = 268.0973; found 268.0974.

Oxiran-2-ylmethyl (*E*)-4-(4-chloro-1H-pyrazol-1-yl)hex-2-enoate (3v):

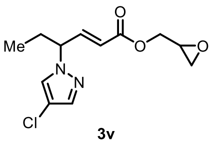

3v

According to general procedure A: Oxiran-2-ylmethyl (*E*)-hex-3-enoate (**1v**) (511 mg, 3.00 mmol, 3.00 equiv.), 4-chloro-1H-pyrazole (**2a**) (103 mg, 1.00 mmol, 1.00 equiv.), TAPT (24.3 mg, 50.0 µmol, 5 mol%), $(PhSe)_2$ (31.2 mg, 100 µmol, 10 mol%) and DCE (5 mL), 21 h. After purification (PE/EtOAc, 10:1 to 5:1), the product was isolated as a colorless oil (200 mg, 739 µmol, 74%; allylic/vinylic isomer 6:1). Crude ^{1}H NMR analysis: total yield: 88%; allylic isomer: 73%; allylic/vinylic isomer: 5:1.

According to general procedure A: Oxiran-2-ylmethyl (*E*)-hex-3-enoate (**1v**) (170 mg, 1.00 mmol, 1.00 equiv.), 4-chloro-1H-pyrazole (**2a**) (308 mg, 3.00 mmol, 3.00 equiv.), TAPT (24.3 mg, 50.0 µmol, 5 mol%), $(PhSe)_2$ (31.2 mg, 100 µmol, 10 mol%) and DCE (5 mL), 21 h. Crude ^{1}H NMR analysis: total yield: 62%; allylic isomer: 60%; allylic/vinylic isomer: 25:1.

TLC: R_f = 0.38 (PE/EtOAc, 2:1) [UV, $KMnO_4$]. IR [cm^{-1}]: 3131, 2971, 2937, 2878, 1722, 1659, 1451, 1387, 1308, 1275, 1178, 1021, 969, 910, 842. **^{1}H NMR** (300 MHz, $CDCl_3$): δ 7.45 (d, J = 0.4 Hz, 1H), 7.39 (d, J = 0.5 Hz, 1H), 7.04 (dd, J = 15.7, 6.0 Hz, 1H), 5.72 (dd, J = 15.7, 1.6 Hz, 1H), 4.77 – 4.64 (m, 1H), 4.48 – 4.39 (m, 1H), 3.99 – 3.89 (m, 1H), 3.24 – 3.15 (m, 1H), 2.87 – 2.80 (m, 1H), 2.65 – 2.58 (m, 1H), 2.15 – 1.86 (m, 2H), 0.88 (t, J = 7.3 Hz, 3H). **^{13}C NMR** (75 MHz, $CDCl_3$): δ 165.3, 146.3, 138.1, 126.3, 122.0, 110.3, 65.4, 65.3, 65.2, 49.2, 44.7, 27.2, 10.5. **HRMS** (EI) calcd. for $[C_{12}H_{15}ClN_2O_3]^{\cdot+}$ ($[M]^{\cdot+}$), m/z = 270.0766; found 270.0772.

Pent-3-yn-1-yl (*E*)-4-(4-chloro-1H-pyrazol-1-yl)hex-2-enoate (3w):

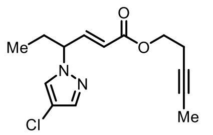

3w

According to general procedure A: Pent-3-yn-1-yl (*E*)-hex-3-enoate (**1w**) (541 mg, 3.00 mmol, 3.00 equiv.), 4-chloro-1H-pyrazole (**2a**) (103 mg, 1.00 mmol, 1.00 equiv.), TAPT (24.3 mg, 50.0 µmol, 5 mol%), $(PhSe)_2$ (31.2 mg, 100 µmol, 10 mol%) and DCE (5 mL), 21 h. After purification (PE/EtOAc, 40:1 to 15:1), the product was isolated as a colorless oil (202 mg, 720 µmol, 72%; allylic/vinylic isomer 6:1). Crude ^{1}H NMR analysis: total yield is 85%; allylic isomer: 76%; allylic/vinylic isomer: 9:1.

According to general procedure A: Pent-3-yn-1-yl (*E*)-hex-3-enoate (**1w**) (541 mg, 3.00 mmol, 3.00 equiv.), 4-chloro-1H-pyrazole (**2a**) (103 mg, 1.00 mmol, 1.00 equiv.), TAPT (24.3 mg, 50.0 µmol, 5 mol%), $(PhSe)_2$ (31.2 mg, 100 µmol, 10 mol%), $(4\text{-}ClPhS)_2$ (14.4 mg, 50.0 µmol, 5 mol%) and DCE (5 mL), 21 h. After purification (PE/EtOAc, 40:1 to 15:1), the product was isolated as a colorless oil (154 mg, 547 µmol, 55%; allylic/vinylic isomer >49:1). Crude ^{1}H NMR analysis: total yield: 63%; allylic isomer: 60%; allylic/vinylic isomer: 20:1.

TLC: R_f = 0.37 (PE/EtOAc, 5:1) [UV, $KMnO_4$]. IR [cm^{-1}]: 3127, 2971, 2922, 1718, 1659, 1525, 1435, 1387, 1312, 1271, 1178, 1074, 1021, 969, 824, 790, 742. **^{1}H NMR** (300 MHz, $CDCl_3$): δ 7.43 (d, J = 0.5 Hz, 1H), 7.38 (d, J = 0.7 Hz, 1H), 6.99 (dd, J = 15.6, 6.0 Hz, 1H), 5.69 (dd, J = 15.7, 1.6 Hz, 1H), 4.74 – 4.60 (m, 1H), 4.13 (t, J = 7.0 Hz, 2H), 2.48 – 2.38 (m, 2H), 2.12 – 1.84 (m, 2H), 1.72 (t, J = 2.5 Hz, 3H), 0.85 (t, J = 7.4 Hz, 3H). **^{13}C NMR** (75 MHz, $CDCl_3$): δ 165.4, 145.7, 138.0, 126.3, 122.5, 110.2, 77.4, 74.5, 65.1, 63.1, 27.2, 19.2, 10.5, 3.4. **HRMS** (EI) calcd. for $[C_{14}H_{17}ClN_2O_2]^{\cdot+}$ ($[M]^{\cdot+}$), m/z = 280.0971; found 280.0966.

4-Oxocyclopent-2-en-1-yl (*E*)-4-(4-chloro-1H-pyrazol-1-yl)hex-2-enoate (3x):

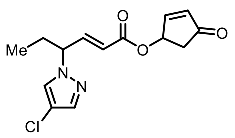

3x

According to general procedure A: 4-Oxocyclopent-2-en-1-yl (*E*)-hex-3-enoate (**1x**) (583 mg, 3.00 mmol, 3.00 equiv.), 4-chloro-1H-pyrazole (**2a**) (103 mg, 1.00 mmol, 1.00 equiv.), TAPT (24.3 mg, 50.0 μmol, 5 mol%), $(PhSe)_2$ (31.2 mg, 100 μmol, 10 mol%) and DCE (5 mL), 21 h. After purification (DCM/CH_3CN, 40:1 to 20:1), the product was isolated as a cololess oil (207 mg, 701 μmol, 70%; allylic/vinylic isomer >49:1). Crude ^{1}H NMR analysis: total yield: 76%; allylic isomer: 66%; allylic/vinylic isomer: 7:1.

TLC: R_f = 0.33 (PE/EtOAc, 2:1) [UV, $KMnO_4$]. IR [cm^{-1}]: 3127, 2971, 2937, 2878, 1715, 1655, 1592, 1525, 1461, 1435, 1387, 1331, 1267, 1163, 1100, 1021, 969, 828, 794. **^{1}H NMR** (300 MHz, $CDCl_3$): δ7.54 (dd, *J* = 5.7, 2.4 Hz, 1H), 7.45 (s, 1H), 7.39 (s, 1H), 7.04 (dd, *J* = 15.8, 5.9 Hz, 1H), 6.31 (dd, *J* = 5.7, 1.3 Hz, 1H), 5.93 – 5.81 (m, 1H), 5.73 – 5.59 (m, 1H), 4.78 – 4.61 (m, 1H), 2.81 (dd, *J* = 18.8, 6.3 Hz, 1H), 2.31 (dd, *J* = 18.7, 2.2 Hz, 1H), 2.16 – 1.86 (m, 2H), 0.87 (t, *J* = 7.3 Hz, 3H). **^{13}C NMR** (75 MHz, $CDCl_3$): δ 204.7, 165.0, 158.7, 146.8, 138.2, 137.2, 126.4, 121.8, 110.3, 72.2, 65.1, 40.9, 27.1, 10.5. **HRMS** (EI) calcd. for $[C_{14}H_{15}ClN_2O_3]^{\cdot+}$ ($[M]^{\cdot+}$), m/z = 294.0766; found 294.0758.

Cyclopent-3-en-1-yl (*E*)-4-(4-chloro-1H-pyrazol-1-yl)hex-2-enoate (3y):

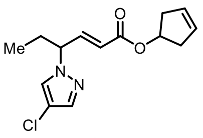

3y

According to general procedure A: Cyclopent-3-en-1-yl (*E*)-hex-3-enoate (**1y**) (541 mg, 3.00 mmol, 3.00 equiv.), 4-chloro-1H-pyrazole (**2a**) (103 mg, 1.00 mmol, 1.00 equiv.), TAPT (24.3 mg, 50.0 μmol, 5 mol%), $(PhSe)_2$ (31.2 mg, 100 μmol, 10 mol%) and DCE (5 mL), 21 h. After purification (PE/EtOAc, 40:1 to 20:1), the product was isolated as a colorless oil (112 mg, 400 μmol, 40%; allylic/vinylic isomer >49:1). Crude ^{1}H NMR analysis: total yield: 46%, allylic isomer: 43%, allylic/vinylic isomer: 13:1.

TLC: R_f = 0.38 (PE/EtOAc, 5:1) [UV, $KMnO_4$]. IR [cm^{-1}]: 3131, 3064, 2971, 2933, 2848, 1715, 1659, 1431, 1364, 1312, 1275, 1238, 1178, 1025, 969, 842, 790, 731, 675. **^{1}H NMR** (300 MHz, $CDCl_3$): δ 7.46 (d, *J* = 0.5 Hz, 1H), 7.39 (d, *J* = 0.7 Hz, 1H), 6.98 (dd, *J* = 15.7, 6.0 Hz, 1H), 5.77 – 5.62 (m, 3H), 5.40 (tt, *J* = 7.0, 2.4 Hz, 1H), 4.76 – 4.62 (m, 1H), 2.81 – 2.65 (m, 2H), 2.45 – 2.34 (m, 2H), 2.15 – 1.87 (m, 2H), 0.88 (t, *J* = 7.3 Hz, 3H). **^{13}C NMR** (75 MHz, $CDCl_3$): δ 165.6, 145.2, 138.0, 128.3, 126.3, 123.0, 110.3, 74.7, 65.2, 39.7, 27.2, 10.5. **HRMS** (EI) calcd. for $[C_{14}H_{17}ClN_2O_2]^{\cdot+}$ ($[M]^{\cdot+}$), m/z = 280.0973; found 280.0975.

Ethyl (*E*)-4-(4-chloro-1H-pyrazol-1-yl)-2-ethylhex-2-enoate (3z):

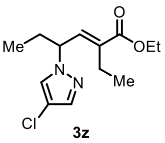

3z

According to to general procedure A: Ethyl (*E*)-2-ethylhex-3-enoate (**1z**) (511 mg, 3.00 mmol, 3.00 equiv.), 4-chloro-1H-pyrazole (**2a**) (103 mg, 1.00 mmol, 1.00 equiv.), TAPT (24.3 mg, 50.0 μmol, 5 mol%), $(PhSe)_2$ (31.2 mg, 100 μmol, 10 mol%) and DCE (5 mL), 21 h. After purification (PE/EtOAc, 100:1 to 40:1), the product was isolated as a lightly yellow oil (182 mg, 671 μmol, 67%; allylic/vinylic isomer >49:1). Crude ^{1}H NMR analysis: yield of allylic isomer: 66%, allylic/vinylic isomer >10:1.

TLC: R_f = 0.33 (PE/EtOAc, 10:1) [UV, $KMnO_4$]. IR [cm^{-1}]: 3131, 2974, 2937, 2878, 1711, 1651, 1525, 1461, 1387, 1290, 1230, 1174, 1148, 1096, 1044, 969, 924, 835, 787. **^{1}H NMR** (400 MHz, $CDCl_3$): δ 7.39 (s, 1H), 7.38 (s, 1H), 6.72 (d, *J* = 9.5 Hz, 1H), 4.93 – 4.72 (m, 1H), 4.20 – 4.11 (m, 2H), 2.40 – 2.29 (m, 2H), 2.12 – 1.95 (m, 1H), 1.88 – 1.75 (m, 1H), 1.25 (t, *J* = 7.2 Hz, 3H), 0.97 (t, *J* = 7.5 Hz, 3H), 0.83 (t, *J* = 7.3 Hz, 3H). **^{13}C NMR** (101 MHz, $CDCl_3$): δ 166.9, 137.5, 137.3, 137.0, 125.7, 109.9, 62.0, 60.9, 28.5, 20.6, 14.2, 13.9, 10.2. **HRMS** (ESI) calcd. for $[C_{13}H_{20}ClN_2O_2]^+$ ($[M+H]^+$), m/z = 271.1208; found 271.1210.

Butyl (*E*)-4-(4-chloro-1H-pyrazol-1-yl)-2-methylbut-2-enoate (3aa):

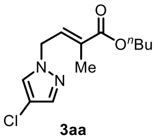
3aa

According to general procedure A: Butyl 2-methylbut-3-enoate (**1aa**) (469 mg, 3.00 mmol, 3.00 equiv.), 4-chloro-1H-pyrazole (**2a**) (103 mg, 1.00 mmol, 1.00 equiv.), TAPT (24.3 mg, 50.0 μmol, 5 mol%), $(PhSe)_2$ (31.2 mg, 100 μmol, 10 mol%) and DCE (5 mL), 21 h. After purification (PE/EtOAc, 40:1 to 20:1), the product was isolated as a lightly yellow oil (157 mg, 612 μmol, 61%; allylic/vinylic isomer >49:1). Crude ^{1}H NMR analysis: yield of allylic isomer: 63%; allylic/vinylic isomer: >10:1.

TLC: R_f = 0.23 (PE/EtOAc, 10:1) [UV, $KMnO_4$]. IR [cm^{-1}]: 3131, 2960, 2937, 2874, 1711, 1655, 1528, 1446, 1387, 1312, 1252, 1200, 1163, 1126, 1077, 1021, 969, 839, 772, 723. **^{1}H NMR** (400 MHz, $CDCl_3$): δ 7.39 (s, 1H), 7.36 (s, 1H), 6.81 – 6.70 (m, 1H), 4.82 (dd, J = 6.6, 0.9 Hz, 2H), 4.10 (t, J = 6.6 Hz, 2H), 1.91 (d, J = 1.1 Hz, 3H), 1.65 – 1.55 (m, 2H), 1.40 – 1.29 (m, 2H), 0.89 (t, J = 7.4 Hz, 3H). **^{13}C NMR** (101 MHz, $CDCl_3$): δ 167.0, 138.1, 133.6, 131.7, 127.0, 110.4, 64.9, 50.5, 30.6, 19.2, 13.7, 12.8. **HRMS** (EI) calcd. for $[C_{12}H_{17}ClN_2O_2]^{\cdot+}$ ($[M]^{\cdot+}$), m/z = 256.0973; found 256.0970.

Diethyl 2-((1R,2R)-1,2-bis(4-chloro-1H-pyrazol-1-yl)hexyl)malonate (3ab):

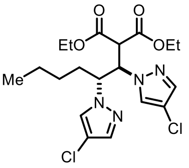
3ab

According to general procedure A: Diethyl (*E*)-2-(hex-1-en-1-yl)malonate (**1ab**) (242 mg, 1.00 mmol, 1.00 equiv.), 4-chloro-1H-pyrazole (**2a**) (667 mg, 6.50 mmol, 6.50 equiv.), TAPT (24.3 mg, 50.0 μmol, 5 mol%), $(PhSe)_2$ (31.2 mg, 100 μmol, 10 mol%) and DCE (5 mL), 21 h. After purification (PE/EtOAc, 10:1), the product was isolated as a colorless solid (298 mg, 668 μmol, 67%; allylic/vinylic isomer >49:1). Crude ^{1}H NMR analysis: yield of allylic isomer: 69%; allylic/vinylic isomer >10:1.

TLC: R_f = 0.36 (PE/EtOAc, 5:1) [UV, $KMnO_4$]. **Mp** 96.9 °C. IR [cm^{-1}]: 3124, 2963, 2930, 2859, 1752, 1528, 1446, 1375, 1346, 1297, 1178, 1148, 1021, 969, 865. **^{1}H NMR** (300 MHz, $CDCl_3$): δ 7.39 (s, 1H), 7.35 (s, 1H), 7.18 (d, J = 0.5 Hz, 1H), 7.08 (d, J = 0.5 Hz, 1H), 5.18 (dd, J = 8.9, 6.4 Hz, 1H), 4.91 – 4.77 (m, 1H), 4.29 – 4.17 (m, 2H), 4.00 (q, J = 7.1 Hz, 2H), 3.91 (d, J = 9.1 Hz, 1H), 1.79 – 1.65 (m, 2H), 1.30 – 1.20 (m, 5H), 1.13 – 0.91 (m, 5H), 0.80 (t, J = 7.1 Hz, 3H). **^{13}C NMR** (75 MHz, $CDCl_3$): δ 166.6, 166.0, 138.3, 138.1, 129.2, 128.1, 109.9, 109.7, 64.1, 62.2, 62.1, 53.7, 30.6, 27.9, 22.1, 13.9, 13.8. **HRMS** (ESI) calcd. for $[C_{19}H_{27}Cl_2N_2O_4]^+$ ($[M+H]^+$), m/z = 445.1404; found 445.1411.

Ethyl (*E*)-4-(4-chloro-1H-pyrazol-1-yl)-4-methylpent-2-enoate (3ac):

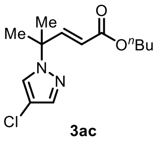
3ac

According to general procedure A: Butyl 4-methylpent-3-enoate (**1ac**) (511 mg, 3.00 mmol, 3.00 equiv.), 4-chloro-1H-pyrazole (**2a**) (103 mg, 1.00 mmol, 1.00 equiv.), TAPT (24.3 mg, 50.0 μmol, 5 mol%), $(PhSe)_2$ (31.2 mg, 100 μmol, 10 mol%) and DCE (5 mL), 21 h. After purification (PE/EtOAc, 40:1), the product was isolated as a colorless oil (185 mg, 683 μmol, 68%; allylic/vinylic isomer >49:1). Crude ^{1}H NMR analysis: yield of allylic isomer: 65%, allylic/vinylic isomer: >10:1.

TLC: R_f = 0.24 (PE/EtOAc, 10:1) [UV, $KMnO_4$]. IR [cm^{-1}]: 3142, 2960, 2874, 1715, 1659, 1525, 1461, 1387, 1290, 1185, 1066, 1025, 969, 839, 790, 723. **^{1}H NMR** (300 MHz, $CDCl_3$): δ 7.52 – 7.40 (m, 2H), 7.01 (d, J = 15.8 Hz, 1H), 5.59 (d, J = 15.8 Hz, 1H), 4.10 (t, J = 6.7 Hz, 2H), 1.70 (s, 6H), 1.65 – 1.55 (m, 2H), 1.42 – 1.28 (m, 2H), 0.90 (t, J = 7.3 Hz, 3H). **^{13}C NMR** (75 MHz, $CDCl_3$): δ 166.1, 150.8, 137.7, 125.0, 120.2, 110.0, 64.7, 62.0, 30.6, 27.0, 19.1, 13.7. **HRMS** (EI) calcd. for $[C_{13}H_{19}ClN_2O_2]^{\cdot+}$ ($[M]^{\cdot+}$), m/z = 270.1130; found 270.1138.

***tert*-Butyl (*E*)-4-(4-chloro-1H-pyrazol-1-yl)but-2-enoate (3ad):**

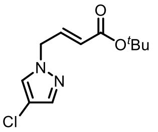
3ad

According to general procedure A: *tert*-Butyl but-3-enoate (**1ad**) (427 mg, 3.00 mmol, 3.00 equiv.), 4-chloro-1H-pyrazole (**2a**) (103 mg, 1.00 mmol, 1.00 equiv.), TAPT (24.3 mg, 50.0 μmol, 5 mol%), $(PhSe)_2$ (31.2 mg, 100 μmol, 10 mol%) and DCE (5 mL), 21 h. After purification (PE/EtOAc, 30:1 to 12:1), the product was

isolated as a lightly yellow oil (161 mg, 663 µmol, 66%; allylic/vinylic isomer >49:1). Crude ^{1}H NMR analysis: yield of allylic isomer: 62%; allylic/vinylic isomer: >10:1.

TLC: R_f = 0.29 (PE/EtOAc, 5:1) [UV, $KMnO_4$]. IR [cm^{-1}]: 3131, 2978, 2933, 1711, 1659, 1446, 1368, 1312, 1219, 1148, 1081, 969, 842, 794, 749, 675. **^{1}H NMR** (300 MHz, $CDCl_3$): δ 7.42 (s, 1H), 7.37 (s, 1H), 6.84 (dt, *J* = 15.6, 5.4 Hz, 1H), 5.63 (dt, *J* = 15.6, 1.7 Hz, 1H), 4.78 (dd, *J* = 5.4, 1.7 Hz, 2H), 1.42 (s, 9H). **^{13}C NMR** (75 MHz, $CDCl_3$): δ 164.7, 139.8, 138.3, 127.5, 125.7, 110.6, 81.0, 53.1, 28.0. **HRMS** (EI) calcd. for $[C_{11}H_{15}ClN_2O_2]^{\cdot+}$ ($[M+H]^{\cdot+}$), m/z = 242.0817; found 242.0812.

(*E*)-4-chloro-1-(4-(phenylsulfonyl)but-3-en-2-yl)-1H-pyrazole (3ae):

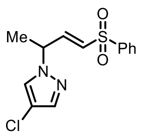

3ae

According to general procedure A: (*E*)-(But-2-en-1-ylsulfonyl)benzene (**1ae**) (589 mg, 3.00 mmol, 3.00 equiv.), 4-chloro-1H-pyrazole (**2a**) (103 mg, 1.00 mmol, 1.00 equiv.), TAPT (24.3 mg, 50.0 µmol, 5 mol%), $(PhSe)_2$ (31.2 mg, 100 µmol, 10 mol%) and DCE (5 mL), 21 h. After purification (PE/EtOAc, 6:1 to 4:1 to afford crude product, then DCM/CH_3CN, 40:1 to 20:1), the product was isolated as a colorless oil (250 mg, 843 µmol, 84%; allylic/vinylic isomer > 49:1). Crude ^{1}H NMR analysis: yield of allylic isomer: 80%; allylic/vinylic isomer: >10:1.

TLC: R_f = 0.33 (PE/EtOAc, 2:1) [UV, $KMnO_4$]. IR [cm^{-1}]: 3131, 3056, 2989, 2937, 1633, 1584, 1446, 1387, 1308, 1204, 1144, 1085, 969, 835, 753, 723, 686. **^{1}H NMR** (300 MHz, $CDCl_3$): δ 7.86 – 7.77 (m, 2H), 7.65 – 7.56 (m, 1H), 7.55 – 7.47 (m, 2H), 7.43 – 7.36 (m, 2H), 7.04 (dd, *J* = 15.1, 4.9 Hz, 1H), 6.13 (dd, *J* = 15.1, 1.6 Hz, 1H), 5.14 – 4.97 (m, 1H), 1.64 (d, *J* = 7.1 Hz, 3H). **^{13}C NMR** (75 MHz, $CDCl_3$): δ 144.5, 139.6, 138.3, 133.8, 131.6, 129.5, 127.8, 125.9, 110.6, 58.0, 19.3. **HRMS** (ESI) calcd. for $[C_{13}H_{14}ClN_2O_2S]^+$ ($[M+H]^+$), m/z = 297.0459; found 297.0464.

(*E*)-4-chloro-1-(3-(phenylsulfonyl)allyl)-1H-pyrazole (3af):

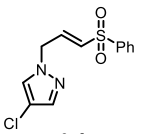

3af

According to general procedure A: (Allylsulfonyl)benzene (**1af**) (547 mg, 3.00 mmol, 3.00 equiv.), 4-chloro-1H-pyrazole (**2a**) (103 mg, 1.00 mmol, 1.00 equiv.), TAPT (24.3 mg, 50.0 µmol, 5 mol%), $(PhSe)_2$ (31.2 mg, 100 µmol, 10 mol%) and DCE (5 mL), 21 h. After purification (PE/EtOAc, 6:1 to 4:1 to afford crude product, then DCM/CH_3CN, 40:1 to 20:1), the product was isolated as a colorless solid (219 mg, 776 µmol, 78%; allylic/vinylic isomer >49:1). Crude ^{1}H NMR analysis: yield of allylic isomer: 78%; allylic/vinylic isomer >10:1.

TLC: R_f = 0.24 (PE/EtOAc, 2:1) [UV, $KMnO_4$]. **Mp** 67.1 °C. IR [cm^{-1}]: 3124, 3068, 1640, 1446, 1383, 1353, 1297, 1144, 1085, 969, 842, 790, 749, 716, 682. **^{1}H NMR** (300 MHz, $CDCl_3$): δ 7.89 – 7.76 (m, 2H), 7.66 – 7.57 (m, 1H), 7.56 – 7.47 (m, 2H), 7.45 – 7.34 (m, 2H), 7.04 (dt, *J* = 15.2, 4.6 Hz, 1H), 6.17 (dt, *J* = 15.2, 1.9 Hz, 1H), 4.89 (dd, *J* = 4.6, 1.9 Hz, 2H). **^{13}C NMR** (75 MHz, $CDCl_3$): δ 140.0, 139.5, 138.9, 133.9, 132.7, 129.5, 127.8, 110.9, 52.1. **HRMS** (ESI) calcd. for $[C_{12}H_{12}ClN_2O_4S]^+$ ($[M+H]^+$), m/z = 283.0303; found 283.0306.

4-(4-Chloro-1H-pyrazol-1-yl)pent-2-enenitrile (3ag):

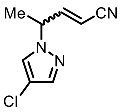

3ag

According to general procedure A: Pent-3-enenitrile (**1ag**) (243 mg, 3.00 mmol, 3.00 equiv.), 4-chloro-1H-pyrazole (**2a**) (103 mg, 1.00 mmol, 1.00 equiv.), TAPT (24.3 mg, 50.0 µmol, 5 mol%), $(PhSe)_2$ (31.2 mg, 100 µmol, 10 mol%) and DCE (5 mL), 21 h. After purification (PE/EtOAc, 6:1 to afford crude product, then DCM/CH_3CN, 40:1), the product was isolated as a colorless oil (137 mg, 752 µmol, 75%; allylic/vinylic isomer >49:1; *E*/*Z* = 6:1). Crude ^{1}H NMR analysis: yield of allylic isomer: 80%, *E*/*Z* = 4:1; allylic/vinylic isomer >10:1.

TLC: R_f = 0.54 (PE/EtOAc, 2:1) [UV, $KMnO_4$]. IR [cm^{-1}]: 3131, 3056, 2989, 2941, 2229, 1640, 1528, 1435, 1387, 1316, 1249, 1174, 1081, 1021, 969, 842, 790, 701. **^{1}H NMR** (300 MHz, $CDCl_3$): δ 7.50 – 7.38 (m, 2H), 6.79 (dd, *J* = 16.3, 5.4 Hz, 0.86H), 6.69 (dd, *J* = 11.0, 8.9 Hz, 0.14H), 5.48 (dd, *J* = 11.0, 0.9 Hz, 0.15H), 5.34 – 5.22 (m, 0.15H), 5.18 (dd, *J* = 16.4, 1.7 Hz, 0.86H), 5.05 – 4.93 (m, 0.87H), 1.68 (dd, *J* = 9.9, 7.0 Hz, 3H). **^{13}C NMR** (75 MHz, $CDCl_3$): δ 152.8, 152.2, 138.5, 126.2, 125.9, 116.2, 114.6, 110.8, 110.5, 101.4, 100.9, 58.9, 57.9, 20.0, 19.1. **HRMS** (EI) calcd. for $[C_8H_8ClN_3]^{\cdot+}$ ($[M]^{\cdot+}$), m/z = 181.0401; found 181.0399.

4-(4-Chloro-1H-pyrazol-1-yl)but-2-enenitrile (3ah):

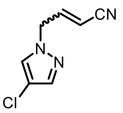

3ah

According to general procedure A: But-3-enenitrile (**1ah**) (201 mg, 3.00 mmol, 3.00 equiv.), 4-chloro-1H-pyrazole (**2a**) (103 mg, 1.00 mmol, 1.00 equiv.), TAPT (24.3 mg, 50.0 µmol, 5 mol%), $(PhSe)_2$ (31.2 mg, 100 µmol, 10 mol%) and DCE (5 mL), 21 h. After purification (PE/EtOAc, 10:1 to 5:1), the product was isolated as a lightly yellow oil (131 mg, 782 µmol, 78%; allylic/vinylic isomer >49:1; *E*/*Z* = 1:1). Crude ^{1}H NMR analysis: yield of allylic isomer: 78%, *E*/*Z* = 1:1; allylic/vinylic isomer >10:1.

TLC: R_f = 0.25 (PE/EtOAc, 2:1) [UV, $KMnO_4$]. IR [cm^{-1}]: 3131, 2930, 2229, 1640, 1528, 1443, 1383, 1316, 1264, 1197, 1159, 1122, 969, 842, 798. **^{1}H NMR** (300 MHz, $CDCl_3$): δ 7.49 – 7.36 (m, 1.95H), 6.77 (dt, *J* = 16.3, 4.9 Hz, 0.55H), 6.64 (dt, *J* = 11.1, 6.7 Hz, 0.44H), 5.57 (dt, *J* = 11.0, 1.6 Hz, 0.44H), 5.21 (dt, *J* = 16.3, 2.0 Hz, 0.54H), 4.98 (dd, *J* = 6.6, 1.6 Hz, 0.88H), 4.82 (dd, *J* = 5.0, 2.0 Hz, 1.12H). **^{13}C NMR** (75 MHz, $CDCl_3$): δ 148.0, 146.9, 139.0, 138.8, 127.8, 127.5, 116.1, 114.6, 111.1, 110.9, 102.9, 102.6, 53.2, 52.0. **HRMS** (EI) calcd. for $[C_7H_6ClN_3]^{\cdot+}$ ($[M]^{\cdot+}$), m/z = 167.0245; found 167.0245.

(*Z*)-4-(4-chloro-1H-pyrazol-1-yl)-2-methylbut-2-enenitrile (3ai):

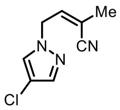

3ai

According to general procedure A: 2-Methylbut-3-enenitrile (**1ai**) (>70% purity, 348 mg, 3.00 mmol, 3.00 equiv.), 4-chloro-1H-pyrazole (**2a**) (103 mg, 1.00 mmol, 1.00 equiv.), TAPT (24.3 mg, 50.0 µmol, 5 mol%), $(PhSe)_2$ (31.2 mg, 100 µmol, 10 mol%) and DCE (5 mL), 21 h. After purification (PE/EtOAc, 15:1 to 10:1), the product was isolated as a colorless oil (92.7 mg, 510 µmol, 51%, *Z* isomer; allylic/vinylic isomer >49:1). Crude ^{1}H NMR analysis: yield of allylic isomer: 53%; allylic/vinylic isomer: >10:1.

TLC: R_f = 0.29 (PE/EtOAc, 5:1) [UV, $KMnO_4$]. IR [cm^{-1}]: 3131, 2930, 2222, 1528, 1439, 1387, 1312, 1223, 1178, 1144, 1081, 1018, 973, 842, 794, 734. **^{1}H NMR** (300 MHz, $CDCl_3$): δ 7.45 – 7.36 (m, 1H), 6.33 (tq, *J* = 6.9, 1.6 Hz, 0H), 4.95 – 4.88 (m, 1H), 2.00 (dd, *J* = 2.9, 1.4 Hz, 1H). **^{13}C NMR** (75 MHz, $CDCl_3$): δ 140.2, 138.6, 127.3, 116.7, 113.8, 110.7, 52.2, 20.2. **HRMS** (EI) calcd. for $[C_8H_8ClN_3]^{\cdot+}$ ($[M]^{\cdot+}$), m/z = 181.0401; found 181.0404.

(*E*)-(3-(4-chloro-1H-pyrazol-1-yl)prop-1-en-1-yl)diphenylphosphine oxide (3aj):

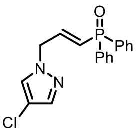

3aj

According to general procedure A: Allyldiphenylphosphine oxide (**1aj**) (242 mg, 1.00 mmol, 1.00 equiv.), 4-chloro-1H-pyrazole (**2a**) (308 mg, 3.00 mmol, 3.00 equiv.), TAPT (24.3 mg, 50.0 µmol, 5 mol%), $(PhSe)_2$ (31.2 mg, 100 µmol, 10 mol%), $(4\text{-}ClPhS)_2$ (14.4 mg, 50.0 µmol, 5 mol%) and DCE (5 mL), 21 h. After purification (EtOAc), the product was isolated as a colorless solid (220 mg, 643 µmol, 64%; allylic/vinylic isomer >49:1). Crude ^{1}H NMR analysis: yield of allylic isomer: 62%; allylic/vinylic isomer: >10:1.

TLC: R_f = 0.17 (EtOAc) [UV, $KMnO_4$]. **Mp** 120.6 °C. IR [cm^{-1}]: 3131, 3053, 3004, 2922, 1711, 1633, 1592, 1484, 1439, 1383, 1349, 1297, 1249, 1182, 1118, 1006, 965, 865, 835, 798, 727, 693. **^{1}H NMR** (300 MHz, $CDCl_3$): δ 7.66 – 7.54 (m, 4H), 7.52 – 7.35 (m, 8H), 6.77 (ddt, *J* = 18.9, 17.0, 4.7 Hz, 1H), 6.11 (ddt, *J* = 22.0, 16.9, 1.8 Hz, 1H), 4.92 – 4.79 (m, 2H). **^{13}C NMR** (75 MHz, $CDCl_3$): δ 144.8 (d, $J_{C\text{-}P}$ = 3.7 Hz), 138.5, 132.1 (d, $J_{C\text{-}P}$ = 2.6 Hz), 132.1 (d, $J_{C\text{-}P}$ = 106.2 Hz), 131.2 (d, $J_{C\text{-}P}$ = 10.3 Hz), 128.7 (d, $J_{C\text{-}P}$ = 12.5 Hz), 127.7, 124.9 (d, $J_{C\text{-}P}$ = 99.9 Hz), 110.6, 54.6 (d, $J_{C\text{-}P}$ = 18.4 Hz). **^{31}P NMR** (162 MHz, $CDCl_3$) δ 23.6. **HRMS** (EI) calcd. for $[C_{18}H_{16}PClN_2O]^{\cdot+}$ ($[M]^{\cdot+}$), m/z = 342.0683; found 342.0678.

(*E*)-4-chloro-1-(tetradec-8-en-7-yl)-1H-pyrazole and (*Z*)-4-chloro-1-(tetradec-7-en-7-yl)-1H-pyrazole (3ak):

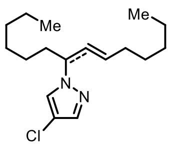

3ak

According to general procedure A: (*E*)-7-Tetradecene (**1ak**) (142 mg, 1.00 mmol, 1.00 equiv.), 4-chloro-1H-pyrazole (**2a**) (308 mg, 3.00 mmol, 3.00 equiv.), TAPT (24.3 mg, 50.0 µmol, 5 mol%), $(PhSe)_2$ (31.2 mg, 100 µmol, 10 mol%), $(4\text{-}ClPhS)_2$ (14.4 mg, 50.0 µmol, 5 mol%) and DCE (5 mL), 8 h. After purification (PE/EtOAc, 200:1 to 150:1 to afford the crude product, then PE/DCM, 10:1 to 5:1), the product

was isolated as a colorless oil (147 mg, 496 µmol, 50%; allylic/vinylic isomer 4:1). Crude ^{1}H NMR analysis: total yield: 49%; allylic isomer: 39%; allylic/vinylic isomer: 4:1).

TLC: R_f = 0.71 (PE/EtOAc, 10:1) [UV, $KMnO_4$]. IR [cm^{-1}]: 3120, 2926, 2866, 1741, 1670, 1525, 1461, 1424, 1383, 1312, 1234, 1163, 1111, 969, 895, 835, 787, 727. **^{1}H NMR** (300 MHz, $CDCl_3$) δ 7.52 (d, J = 0.5 Hz, 0.21H), 7.41 (d, J = 0.7 Hz, 0.77H), 7.38 – 7.35 (m, 1.00H), 5.68 – 5.50 (m, 1.57H), 5.37 (t, J = 7.3 Hz, 0.23H), 4.97 – 4.86 (m, 0.06H), 4.62 – 4.50 (m, 0.72H), 2.44 (t, J = 5.8 Hz, 0.47H), 2.09 – 1.69 (m, 3.50H), 1.48 – 1.14 (m, 14.58H), 0.99 – 0.75 (m, 6.07H). **^{13}C NMR** (75 MHz, $CDCl_3$) δ 138.9, 138.0, 137.1, 134.6, 128.6, 128.1, 125.6, 124.8, 109.7, 109.4, 65.3, 35.7, 35.0, 32.2, 31.60, 31.5, 31.4, 29.5, 28.9, 28.6, 28.5, 27.1, 27.0, 25.9, 22.6, 22.5, 14.0. **HRMS** (EI) calcd. for $[C_{17}H_{29}ClN_2]^{\cdot+}$ ($[M]^{\cdot+}$), m/z = 296.2014; found 296.2004.

(*E*)-1-(tetradec-8-en-7-yl)-1H-benzo[d][1,2,3]triazole (3al) and (*Z*)-1-(tetradec-7-en-7-yl)-1H-benzo[d][1,2,3]triazole (3al'):

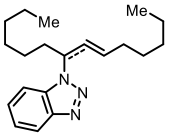

3al

According to general procedure A: (*E*)-7-Tetradecene (**1ak**) (196 mg, 1.00 mmol, 1.00 equiv.), 1H-benzo[d][1,2,3]triazole (**2f**) (357 mg, 3.00 mmol, 3.00 equiv.), TAPT (24.3 mg, 50.0 µmol, 5 mol%), $(PhSe)_2$ (31.2 mg, 100 µmol, 10 mol%), $(4\text{-}ClPhS)_2$ (14.4 mg, 50.0 µmol, 5 mol%) and DCE (5 mL), 8 h. After purification (PE/EtOAc, 100:1 to 40:1), the product was isolated as a colorless oil (160 mg, 510 µmol, 51%; allylic/vinylic isomer 1:1). Further purification afforded small amounts of the isolated isomers for analysis. Crude ^{1}H NMR analysis: total yield: 60%; allylic isomer: 30%; allylic/vinylic isomer: 1:1.

Allylic isomer (3al): TLC: R_f = 0.50 (PE/EtOAc, 5:1) [UV, $KMnO_4$]. IR [cm^{-1}]: 3064, 2926, 2855, 1614, 1491, 1454, 1379, 1271, 1238, 1156, 1066, 1003, 969, 783, 746. **^{1}H NMR** (300 MHz, $CDCl_3$): δ 8.10 – 8.00 (m, 1H), 7.58 – 7.49 (m, 1H), 7.47 – 7.39 (m, 1H), 7.37 – 7.30 (m, 1H), 5.86 – 5.57 (m, 2H), 5.40 – 5.10 (m, 1H), 2.41 – 1.93 (m, 4H), 1.49 – 1.06 (m, 14H), 0.96 – 0.70 (m, 6H). **^{13}C NMR** (75 MHz, $CDCl_3$): δ 146.2, 134.8, 132.3, 127.7, 126.8, 123.8, 120.0, 110.2, 62.4, 34.2, 32.1, 31.6, 31.3, 28.8, 28.6, 26.1, 22.5, 22.4, 14.0. **HRMS** (EI) calcd. for $[C_{20}H_{31}N_3]^{\cdot+}$ ($[M]^{\cdot+}$), m/z = 313.2513; found 313.2515.

Vinylic isomer (3al'): TLC: R_f = 0.57 (PE/EtOAc, 5:1) [UV, $KMnO_4$]. IR [cm^{-1}]: 3064, 2926, 2855, 1737, 1677, 1614, 1454, 1402, 1275, 1238, 1167, 1111, 1062, 999, 783, 746. **^{1}H NMR** (300 MHz, $CDCl_3$): δ 8.11 – 8.01 (m, 1H), 7.51 – 7.43 (m, 2H), 7.40 – 7.31 (m, 2H), 5.81 (t, J = 7.4 Hz, 1H), 2.58 (t, J = 6.5 Hz, 2H), 1.72 (q, J = 7.3 Hz, 2H), 1.37 – 1.01 (m, 16H), 0.86 – 0.67 (m, 6H). **^{13}C NMR** (75 MHz, $CDCl_3$): δ 145.3, 135.1, 133.3, 129.6, 127.6, 123.8, 120.0 110.1, 36.2, 31.4, 29.0, 28.7, 28.5, 27.4, 27.0, 22.5, 14.0. **HRMS** (EI) calcd. for $[C_{20}H_{31}N_3]^{\cdot+}$ ($[M]^{\cdot+}$), m/z = 313.2513; found 313.2515.

(*E*)-*N*-(dec-6-en-5-yl)-N,4-dimethylbenzenesolfonamide (3am):

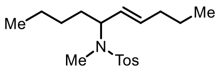

3am

(*E*)-Dec-5-ene (**1al**) (70.2 mg, 95 µL, 500 µmol, 1.00 equiv.), *N*,4-dimethylbenzenesulfonamide (**2t**) (463 mg, 2.50 mmol, 5.00 equiv.), TAPT (24.4 mg, 50.1 µmol, 10 mol%), $(PhSe)_2$ (31.2 mg, 100 µmol, 20 mol%) and PhCl (5 mL) were added into a 40 mL vial. The vial was then equipped with a balloon filled with air and the resulting mixture was vigorously stirred at 19 °C and irradiated for 8 h. Afterwards, the reaction mixture was transferred into a round bottom flask using DCM and the solvent was removed under reduced pressure. After purification (PE/EtOAc, 95:5 to 90:10), the product was isolated as a lightly yellow oil (39.0 mg, 121 µmol, 24%; allylic/vinylic isomer >49:1. Crude ^{1}H NMR analysis: yield of allylic isomer is 30%, allylic/vinylic isomer >10:1.

TLC R_f = 0.39 (PE/EtOAc, 9:1) [UV, *p*-anisaldehyde]. **IR** [cm^{-1}] 2960, 2870, 1718, 1599, 1494, 1461, 1334, 1156, 1088, 969, 921, 813. **^{1}H NMR** (300 MHz, $CDCl_3$) δ 7.71 – 7.60 (m, 2H), 7.30 – 7.21 (m, 2H+peak of $CHCl_3$), 5.46 – 5.32 (m,1H), 5.14 – 5.00 (m, 1H), 4.34 (quint, J = 7.0 Hz, 1H), 2.65 (d, J = 1.2 Hz, 3H), 2.41 (s, 3H), 1.93 – 1.76 (m, 2H), 1.50 – 1.38 (m, 2H), 1.39 – 1.09 (m, 6H), 0.98 – 0.70 (m, 6H). **^{13}C NMR** (101 MHz, $CDCl_3$): δ 142.8, 142.7, 137.3, 137.3, 134.2, 134.0, 129.4,

127.4, 127.3, 126.9, 126.6, 58.7, 58.4, 34.4, 34.3, 32.0, 31.8, 31.2, 28.5, 28.5, 28.4, 22.4, 22.1, 22.1, 21.5, 21.5, 19.4, 14.0, 13.9, 13.8, 13.6. **HRMS** (ESI) calcd. for $[C_{18}H_{30}NO_2S]^+$ ($[M+H]^+$), m/z = 324.1994, found 324.1992.

(*E*)-4-chloro-1-(cyclododec-2-en-1-yl)-1H-pyrazole (3an):

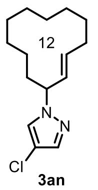

3an

According to general procedure A: Cyclododecene (**1am**) (160 mg, 964 µmol, 1.00 equiv.), 4-chloro-1H-pyrazole (**2a**) (308 mg, 3.01 mmol, 3.12 equiv.), TAPT (24.4 mg, 50.1 µmol, 5 mol%), $(PhSe)_2$ (31.3 mg, 100 µmol, 10 mol%), $(4\text{-ClPhS})_2$ (14.4 mg, 50.1 µmol, 5 mol%) and DCE (5 mL), 21 h. After purification (PE/EtOAc, 30:1), the product was isolated as a colorless solid (128 mg, 478 µmol, 50%; allylic/vinylic isomer: >49:1). Crude ^{1}H NMR analysis: yield of allylic isomer: 53%; allylic/vinylic isomer: >5:1.

TLC: R_f = 0.50 (PE/EtOAc, 10:1) [UV, $KMnO_4$]. **Mp** 49.8°C. IR [cm^{-1}]: 2926, 2855, 1461, 1387, 1308, 1167, 969, 835. **^{1}H°NMR** (400 MHz, $CDCl_3$): δ 7.51 – 7.37 (m, 2H), 5.77 (ddd, J = 14.9, 10.4, 4.3 Hz, 2H), 5.58 (ddd, J = 15.2, 9.4, 1.3 Hz, 2H), 4.56 (ddd, J = 11.2, 9.4, 3.9 Hz, 1H), 2.32 – 2.22 (m, 1H), 2.18 – 2.06 (m, 1H), 2.07 – 1.94 (m, 1H), 1.86 – 1.72 (m, 1H), 1.69 – 1.56 (m, 2H), 1.54 – 1.43 (m, 1H), 1.42 – 1.19 (m, 11H). **^{13}C NMR** (101 MHz, $CDCl_3$): δ 137.1, 136.6, 128.5, 125.3, 109.4, 65.9, 33.1, 31.9, 25.9, 25.9, 25.0, 24.6, 24.6, 24.6, 23.1. **HRMS** (ESI) calcd. for $[C_{15}H_{24}ClN_2]^+$ ($[M+H]^+$), m/z = 267.1623; found 267.1626.

(*E*)-1-(cyclododec-2-en-1-yl)-1H-benzo[d][1,2,3]triazole (3ao):

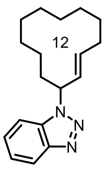

3ao

According to general procedure A: Cyclododecene (**1am**) (160 mg, 964 mmol, 1.00 equiv.), 1H-benzo[d][1,2,3]triazole (**2f**) (357 mg, 3.00 mmol, 3.11 equiv.), TAPT (24.4 mg, 50.1 µmol, 5 mol%), $(PhSe)_2$ (31.3 mg, 100 µmol, 10 mol%), $(4\text{-ClPhS})_2$ (14.4 mg, 50.1 µmol, 5 mol%) and DCE (5 mL), 21 h. After purification (PE/EtOAc, 30:1 to 15:1), the product was isolated as a colorless solid (153 mg, 539 µmol, 56%; allylic/vinyl isomer >49:1). Crude ^{1}H NMR analysis: yield of allylic isomer: 52%; allylic/vinylic isomer: >5:1.

TLC: R_f = 0.62 (PE/EtOAc, 5:1) [UV, $KMnO_4$]. **Mp** 84.2 °C. IR [cm^{-1}]: 2926, 2855, 1614, 1454, 1267, 1238, 1159, 1074, 977, 746. **^{1}H NMR** (400 MHz, $CDCl_3$): δ 8.05 (d, J = 8.4 Hz, 1H), 7.56 (d, J = 8.3 Hz, 1H), 7.45 (t, J = 7.6 Hz, 1H), 7.38 – 7.32 (m, 1H), 5.94 – 5.80 (m, 2H), 5.24 – 5.10 (m, 1H), 2.39 – 2.29 (m, 1H), 2.28 – 2.14 (m, 2H), 2.06 – 1.95 (m, 1H), 1.81 – 1.58 (m, 3H), 1.58 – 1.25 (m, 11H). **^{13}C NMR** (101 MHz, $CDCl_3$): δ 146.2, 136.6, 132.2, 128.0, 126.8, 123.7, 120.0, 110.0, 63.1, 32.5, 31.7, 25.9, 25.8, 25.0, 24.7, 24.6, 24.3, 23.4. **HRMS** (ESI) calcd. for $[C_{18}H_{26}N_3]^+$ ($[M+H]^+$), m/z = 284.2121; found 284.2128.

(*E*)-4-chloro-1-(2-methylhex-3-en-2-yl)-1H-pyrazole (3ap):

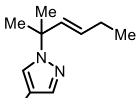

3ap

According to general procedure A: 2-Methylhex-2-ene (**1an**) (295 mg, 3.00 mmol, 3.00 equiv.), 4-chloro-1H-pyrazole (**2a**) (103 mg, 1.00 mmol, 1.00 equiv.), TAPT (24.4 mg, 50.1 µmol, 5 mol%), $(PhSe)_2$ (31.2 mg, 100 µmol, 10 mol%), $(4\text{-ClPhS})_2$ (14.4 mg, 50.1 µmol, 5 mol%) and DCE (5 mL), 21 h. After purification (PE/EtOAc, flash column), the product was isolated as a lightly yellow oil (82 mg, 413 µmol, 41%; allylic/vinylic isomer >49:1). Crude ^{1}H NMR analysis: yield of allylic isomer: 46%; allylic/vinylic isomer: >10:1.

TLC: R_f = 0.51 (PE/EtOAc, 95:5) [UV, $KMnO_4$]. IR [cm^{-1}]: 3146, 3027, 2967, 2937, 2878, 1461, 1387, 1327, 1271, 1167, 969, 835, 790. **^{1}H NMR** (300 MHz, $CDCl_3$): δ 7.51 – 7.36 (m, 2H), 5.76 – 5.45 (m, 2H), 2.19 – 1.99 (m, 2H), 1.62 (s, 6H), 1.00 (t, J = 7.5 Hz, 3H). **^{13}C NMR** (101 MHz, $CDCl_3$): δ 137.2, 133.6, 132.2, 125.1, 109.2, 62.3, 27.9, 25.4, 13.5. **HRMS** (EI) calcd. for $[C_{10}H_{15}ClN_2]^{\cdot+}$ ($[M+H]^{\cdot+}$), m/z = 198.0918; found 198.0917.

(*E*)-1-(8-((*tert*-butyldimethylsilyl)oxy)-2,6-dimethyloct-3-en-2-yl)-4-chloro-1H-pyrazole (3aq):

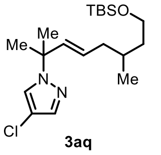

3aq

According to general procedure A: *tert*-Butyl((3,7-dimethyloct-6-en-1-yl)oxy)dimethylsilane (**1ao**) (271 mg, 1.00 mmol, 1.00 equiv.), 4-chloro-1H-pyrazole (**2a**) (308 mg, 3.00 mmol, 3.00 equiv.), TAPT (24.3 mg, 50.0 µmol, 5 mol%), $(PhSe)_2$ (31.2 mg, 100 µmol, 10 mol%), $(4\text{-}ClPhS)_2$ (14.4 mg, 50.0 µmol, 5 mol%) and DCE (5 mL), 8 h. After purification (PE/EtOAc, 40:1), the product was isolated as a lightly yellow oil (126 mg, 340 µmol, 34%; allylic/vinylic isomer >49:1). Crude ^{1}H NMR analysis: yield of allylic isomer: 46%; allylic:/vinylic isomer: >10:1.

TLC: R_f = 0.46 (PE/EtOAc, 10:1) [UV, $KMnO_4$]. IR [cm^{-1}]: 2956, 2859, 1730, 1461, 1387, 1252, 1156, 1088, 969, 895, 835, 775, 731. **^{1}H NMR** (300 MHz, $CDCl_3$): δ 7.49 – 7.37 (m, 2H), 5.68 (d, *J* = 15.6 Hz, 1H), 5.54 (dt, *J* = 15.6, 6.9 Hz, 1H), 3.70 – 3.56 (m, 2H), 2.15 – 2.04 (m, 1H), 1.98 – 1.86 (m, 1H), 1.75 – 1.47 (m, 8H), 1.39 – 1.25 (m, 1H), 0.90 – 0.85 (m, 12H), 0.04 (s, 6H). **^{13}C NMR** (75 MHz, $CDCl_3$): δ 137.0, 135.8, 129.0, 125.1, 109.1, 62.2, 61.2, 39.7, 39.4, 29.6, 27.8, 26.0, 19.6, 18.3, -5.3. **HRMS** (EI) calcd. for $[C_{19}H_{35}ClN_2OSi]^{\cdot+}$ ($[M]^{\cdot+}$), m/z = 370.2202; found 370.2198.

Ethyl (*E*)-4-(4-iodo-1H-pyrazol-1-yl)hex-2-enoate (3ar):

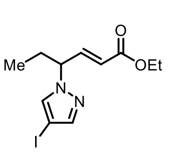

3ar

According to general procedure A: Ethyl (*E*)-hex-3-enoate (**1a**) (143 mg, 1.01 mmol, 1.00 equiv.), 4-iodo-1H-pyrazole (**2b**) (582 mg, 3.00 mmol, 3.00 equiv.), TAPT (24.4 mg, 50.1 µmol, 5 mol%), $(PhSe)_2$ (31.3 mg, 100 µmol, 10 mol%) and DCE (5 mL), 21 h. After purification (PE/EtOAc, 95:5), the product was isolated as a lightly yellow oil (214 mg, 640 µmol, 64%; in which allylic/vinylic isomer >49:1. Crude ^{1}H NMR analysis: total yield: 66%; allylic isomer: 64%; allylic/vinylic isomer: 40:1.

According to general procedure A: Ethyl (*E*)-hex-3-enoate (**1a**) (582 mg, 1.00 mmol, 1.00 equiv.), 4-iodo-1H-pyrazole (307.6 mg, 3.00 mmol, 3.00 equiv.), TAPT (24.3 mg, 50.0 µmol, 5 mol%), $(PhSe)_2$ (31.2 mg, 100 µmol, 10 mol%), $(4\text{-}ClPhS)_2$ (14.4 mg, 50.0 µmol, 5 mol%) and DCE (5 mL), 21 h. Crude ^{1}H NMR analysis: total yield: 67%, allylic isomer: 64%, allylic/vinylic isomer: 25:1.

TLC: R_f = 0.55 (PE/EtOAc, 4:1) [UV, $KMnO_4$]. IR [cm^{-1}]: 3124, 2974, 2937, 1715, 1659, 1428, 1368, 1308, 1271, 1182, 1036, 977, 939, 850. **^{1}H NMR** (300 MHz, $CDCl_3$): δ 7.55 (s, 1H), 7.48 – 7.38 (m, 1H), 7.00 (dd, *J* = 15.7, 6.1 Hz, 1H), 5.70 (dd, *J* = 15.8, 1.5 Hz, 1H), 4.76 (dtd, *J* = 7.5, 6.2, 1.5 Hz, 1H), 4.17 (q, *J* = 7.1 Hz, 2H), 2.18 – 1.89 (m, 2H), 1.26 (t, *J* = 7.1 Hz, 3H), 0.88 (t, *J* = 7.3 Hz, 3H). **^{13}C NMR** (101 MHz, $CDCl_3$): δ 165.7, 145.1, 144.6, 132.7, 123.0, 65.0, 60.8, 56.4, 27.3, 14.2, 10.5. **HRMS** (EI) calcd. for $[C_{11}H_{15}IN_2O_2]^{\cdot+}$ ($[M]^{\cdot+}$), m/z = 334.0173; found 334.0169.

Ethyl (*E*)-1-(6-ethoxy-6-oxohex-4-en-3-yl)-1H-pyrazole-4-carboxylate (3as):

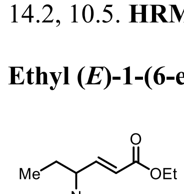

3as

According to general procedure A: Ethyl (*E*)-hex-3-enoate (**1a**) (143 mg, 1.01 mmol, 1.00 equiv.), ethyl 1H-pyrazole-4-carboxylate (***2c***) (422 mg, 3.01 mmol, 2.99 equiv.), TAPT (24.4 mg, 50.2 µmol, 5 mol%), $(PhSe)_2$ (31.2 mg, 100 µmol, 10 mol%) and DCE (5 mL), 21 h. After purification (PE/EtOAc/DCM, 90:10:5), the product was isolated as a colorless oil (169 mg, 603 µmol, 60%; allylic/vinylic isomer >49:1). Crude ^{1}H NMR analysis: total yield: 74%; allylic isomer: 62%; allylic/vinylic isomer: 5:1.

According to general procedure A: Ethyl (*E*)-hex-3-enoate (142 mg, 1.00 mmol, 1.00 equiv.), ethyl 1H-pyrazole-4-carboxylate (420 mg, 3.00 mmol, 3.00 equiv.), TAPT (24.3 mg, 50.0 µmol, 5 mol%), $(PhSe)_2$ (31.2 mg, 100 µmol, 10 mol%), $(4\text{-}ClPhS)_2$ (14.4 mg, 50.0 µmol, 5 mol%) and DCE (5.0 mL), 21 h. Crude ^{1}H NMR analysis: total yield is 74%, yield of allylic isomer is 64%, allylic: vinyl isomer = 6:1.

TLC: R_f = 0.31 (PE/EtOAc, 4:1) [UV, $KMnO_4$]. IR [cm^{-1}]: 3124, 2978, 2937, 1715, 1662, 1554, 1230, 1174, 1126, 1025, 977, 768. **^{1}H NMR** (300 MHz, $CDCl_3$): δ (300 MHz, $CDCl_3$) δ 8.00 – 7.86 (m, 2H), 7.03 (dd, *J* = 15.8, 6.2 Hz, 1H), 5.72 (dd, *J* = 15.6, 1.5 Hz, 1H), 4.77 (dtd, *J* = 7.5, 6.0, 1.4 Hz, 1H), 4.29 (q, *J* = 7.1 Hz, 2H), 4.18 (q, *J* = 7.1 Hz, 2H), 2.24 – 1.90 (m, 2H), 1.34 (t, *J* = 7.1 Hz, 3H), 1.26 (t, *J* = 7.1 Hz, 3H), 0.89 (t, *J* = 7.4 Hz, 3H). **^{13}C NMR** (75 MHz, $CDCl_3$): δ 165.6, 162.9, 144.7, 141.3, 131.8, 123.2, 115.3, 65.1, 60.8, 60.3, 27.3, 14.4, 14.2, 10.5. **HRMS** (ESI) calcd. for $[C_{14}H_{21}N_2O_4]^+$ ($[M+H]^+$), m/z = 281.1496; found 281.1504.

Ethyl (*E*)-4-(3-phenyl-1H-pyrazol-1-yl)hex-2-enoate (*N*1-isomer) and ethyl (*E*)-4-(5-phenyl-1H-pyrazol-1-yl)hex-2-enoate (*N*2-isomer) (3at):

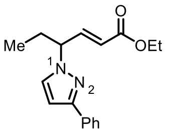

3at (*N*1-isomer)

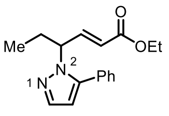

3at (*N*2-isomer)

According to general procedure A: Ethyl (*E*)-hex-3-enoate (**1a**) (143 mg, 1.01 mmol, 1.00 equiv.), 3-phenyl-1H-pyrazole (***2d***) (432 mg, 3.00 mmol, 2.98 equiv.), TAPT (24.5 mg, 50.3 µmol, 5 mol%), $(PhSe)_2$ (31.2 mg, 99.9 µmol, 10 mol%) and DCE (5 mL), 21 h. After purification (hexane/EtOAc, flash column), the product was isolated as a colorless oil (145 mg, 510 µmol, 51%, *N*1:*N*2 = 5:1; allylic/vinylic isomer >49:1. Crude ^{1}H NMR analysis: yield of allylic isomer: 53%; allylic/vinylic isomer: >10:1.

According to general procedure A: Ethyl (*E*)-hex-3-enoate (142.2 mg, 1.00 mmol, 1.00 equiv.), 3-phenyl-1H-pyrazole (432.5 mg, 3.00 mmol, 3.00 equiv.), TAPT (24.3 mg, 50.0 µmol, 5 mol%), $(PhSe)_2$ (31.2 mg, 100 µmol, 10 mol%), $(4\text{-}ClPhS)_2$ (14.4 mg, 50.0 µmol, 5 mol%) and DCE (5 mL), 21 h. Crude ^{1}H NMR analysis: yield of allylic isomer: 54%; allylic/vinylic isomer: >10:1.

TLC: R_f = 0.51 (PE/EtOAc, 4:1) [UV, $KMnO_4$]. IR [cm^{-1}]: 2974, 2937, 1715, 1659, 1498, 1457, 1368, 1308, 1271, 1233, 1177, 1036, 977, 749, 693. **^{1}H NMR** (400 MHz, $CDCl_3$): δ 7.86 – 7.78 (m, 1.68H), 7.71 – 7.60 (m, 0.15H), 7.57 – 7.24 (m, 3.03H), 7.34 – 7.27 (m, 1.15H), 7.20 – 7.06 (m, 1.01H), 6.60 (d, *J* = 2.4 Hz, 0.84H), 6.29 (d, *J* = 1.8 Hz, 0.15H), 5.76 (dd, *J* = 15.7, 1.5 Hz, 0.82H), 5.67 (dd, *J* = 15.7, 1.6 Hz, 0.16H), 4.92 – 4.80 (m, 0.86H), 4.79 – 4.69 (m, 0.16H), 4.26 – 4.11 (m, 2.03H), 2.28 – 2.10 (m, 1.07H), 2.09 – 1.86 (m, 1.06H), 1.30 – 1.25 (m, 3.02H), 0.94 (t, *J* = 7.4 Hz, 2.57H), 0.73 (t, *J* = 7.3 Hz, 0.45H). **^{13}C NMR** (101 MHz, $CDCl_3$): δ 166.1, 166.0, 151.5, 146.9, 146.0, 144.7, 139.6, 133.5, 130.6, 129.3, 129.1, 128.8, 128.7, 128.6, 127.6, 125.7, 122.6, 122.2, 105.9, 103.0, 64.7, 60.8, 60.6, 27.7, 27.5, 14.2, 10.6. **HRMS** (EI) calcd. for $[C_{17}H_{20}N_2O_2]^{\cdot+}$ ($[M]^{\cdot+}$), m/z = 284.1519; found 284.1514 and 284.1517.

Ethyl (*E*)-4-(4-bromo-3-methyl-1H-pyrazol-1-yl)hex-2-enoate (*N*1-isomer) and ethyl (E)-4-(4-bromo-5-methyl-1H-pyrazol-1-yl)hex-2-enoate (*N*2-isomer) (3au):

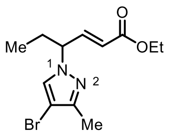

3au (*N*1-isomer)

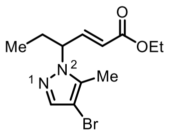

3au (*N*2-isomer)

According to general procedure A: Ethyl (*E*)-hex-3-enoate (**1a**) (142 mg, 1.00 mmol, 1.00 equiv.), 4-bromo-3-methyl-1H-pyrazole (**2e**) (484 mg, 3.01 mmol, 3.01 equiv.), TAPT (24.3 mg, 50.0 µmol, 5 mol%), $(PhSe)_2$ (31.4 mg, 101 µmol, 10 mol%) and DCE (5 mL), 21 h. After purification (PE/EtOAc, flash column), the product was isolated as a lightly yellow oil (172 mg, 571 µmol, 57%, *N*1/*N*2 = 5:1; allylic/vinylic isomer >49:1). Crude ^{1}H NMR analysis: yield of allylic isomer: 58%; allylic/vinylic isomer >10:1.

According to general procedure A: ethyl (*E*)-hex-3-enoate (142 mg, 1.00 mmol, 1.00 equiv.), 4-bromo-3-methyl-1H-pyrazole (483 mg, 3.00 mmol, 3.00 equiv.), TAPT (24.3 mg, 50.0 µmol, 5 mol%), $(PhSe)_2$ (31.2 mg, 100 µmol, 10 mol%), $(4\text{-}ClPhS)_2$ (14.4 mg, 50.0 µmol, 5 mol%) and DCE (5 mL), 21 h. Crude ^{1}H NMR analysis: yield of allylic isomer: 63%; allylic/vinylic isomer: >10:1.

TLC: R_f = 0.50 (PE/EtOAc, 4:1) [UV, $KMnO_4$]. IR [cm^{-1}]: 3127, 1974, 2933, 2876, 1718, 1659, 1368, 1312, 1274, 1238, 1178, 1059, 980, 861, 828. **^{1}H NMR** (400 MHz, $CDCl_3$): δ 7.48 (s, 0.17H), 7.35 (s, 0.83H), 7.07 – 6.91 (m, 1H), 5.71 (dd, *J* = 15.7, 1.5 Hz, 0.82H), 5.58 (dd, *J* = 15.8, 1.6 Hz, 0.17H), 4.70 – 4.60 (m, 1.02H), 4.23 – 4.11 (m, 2.01H), 2.25 – 2.22 (m,

3.01H), 2.13 – 1.84 (m, 1.05H), 2.00 – 1.88 (m, 1.05H), 1.29 – 1.24 (m, 3.08H), 0.92 – 0.84 (m, 3.10H). **^{13}C NMR** (101 MHz, $CDCl_3$): δ 165.9, 147.7, 145.7, 145.5, 139.4, 137.2, 128.8, 122.9, 122.4, 94.1, 93.7, 77.2, 62.1, 60.8, 27.3, 26.9, 14.3, 12.1, 10.9, 10.7, 9.8. **HRMS** (EI) calcd. for $[C_{12}H_{17}BrN_2O_2]^{\cdot+}$ ($[M]^{\cdot+}$), m/z = 300.0468; found 300.0462 and 300.0462.

Ethyl (*E*)-4-(1H-benzo[d][1,2,3]triazol-1-yl)hex-2-enoate (3av):

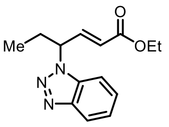

3av

According to general procedure A: Ethyl (*E*)-hex-3-enoate (**1a**) (142 mg, 1.00 mmol, 1.00 equiv.), 1H-benzo[d][1,2,3]triazole (**2f**) (358 mg, 3.01 mmol, 3.00 equiv.), TAPT (24.3 mg, 50.0 µmol, 5 mol%), $(PhSe)_2$ (31.2 mg, 100 µmol, 10 mol%) and DCE (5 mL), 21 h. After purification (PE/EtOAc, 9:1), the product was isolated as a lightly yellow oil (157 mg, 605 µmol, 61%; allylic/vinylic isomer >49:1). Crude ^{1}H NMR analysis: total yield: 74%, yield of allylic isomer is 62%; allylic/vinylic isomer = 4:1.

According to general procedure A: Ethyl (*E*)-hex-3-enoate (**1a**) (142 mg, 1.00 mmol, 1.00 equiv.), 1H-benzo[d][1,2,3]triazole (**2f**) (357 mg, 3.00 mmol, 3.00 equiv.), TAPT (24.3 mg, 50.0 µmol, 5 mol%), $(PhSe)_2$ (31.2 mg, 100 µmol, 10 mol%), $(4\text{-}ClPhS)_2$ (14.4 mg, 50.0 µmol, 5 mol%) and DCE (5 mL), 21 h. Crude ^{1}H NMR analysis: total yield: 76%, allylic isomer: 64%; allylic/vinylic isomer: 5:1.

TLC: R_f = 0.31 (PE/EtOAc, 4:1) [UV, $KMnO_4$]. IR [cm^{-1}]: 3064, 2974, 2937, 2876, 1718, 1659, 1454, 1271, 1238, 1185, 1036, 977, 746. **^{1}H NMR** (300 MHz, $CDCl_3$): δ 8.09 (d, *J* = 8.3 Hz, 1H), 7.50 – 7.47 (m, 2H), 7.44 – 7.33 (m, 1H), 7.20 (dd, *J* = 15.8, 6.0 Hz, 1H), 5.76 (dd, *J* = 15.8, 1.5 Hz, 1H), 5.46 – 5.30 (m, 1H), 4.16 (q, *J* = 7.1 Hz, 2H), 2.51 – 2.19 (m, 1H), 2.34 – 2.21 (m, 1H), 1.24 (t, *J* = 7.1 Hz, 3H), 0.91 (t, *J* = 7.4 Hz, 3H). **^{13}C NMR** (101 MHz, $CDCl_3$): δ 165.5, 146.2, 143.8, 132.5, 127.5, 124.2, 123.5, 120.3, 109.6, 62.1, 60.9, 26.8, 14.1, 10.7. **HRMS** (ESI) calcd. for $[C_{14}H_{18}N_3O_2]^+$ ($[M+H]^+$), m/z = 260.1394; found 260.1399.

Ethyl (*E*)-4-(5-methyl-1H-benzo[d][1,2,3]triazol-1-yl)hex-2-enoate (*N*1-isomer) and ethyl (*E*)-4-(6-methyl-1H-benzo[d][1,2,3]triazol-1-yl)hex-2-enoate (*N*3-isomer) (3aw):

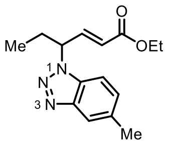

3aw (*N*1-isomer)

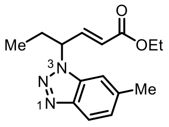

3aw (*N*3-isomer)

According to general procedure A: Ethyl (*E*)-hex-3-enoate (**1a**) (142 mg, 1.00 mmol, 1.00 equiv.), 6-methyl-1H-benzo[d][1,2,3]triazole (**2g**) (401 mg, 3.01 mmol, 3.01 equiv.), TAPT (24.3 mg, 49.9 µmol, 5 mol%), $(PhSe)_2$ (31.3 mg, 100 µmol, 10 mol%) and DCE (5 mL), 21 h. After purification (PE/EtOAc, 95:5 to 9:1), the product was isolated as a lightly yellow oil (169 mg, 618 µmol, 62%, *N*1:*N*3 = 1:1; allylic/vinylic isomer >49:1). Crude ^{1}H NMR analysis: total yield: 79%; yield of allylic isomer: 61%; allylic/vinylic isomer = 3:1.

According to general procedure A: Ethyl (*E*)-hex-3-enoate (**1a**) (142 mg, 1.00 mmol, 1.00 equiv.), 6-methyl-1H-benzo[d][1,2,3]triazole (**2g**) (400 mg, 3.00 mmol, 3.00 equiv.), TAPT (24.3 mg, 50.0 µmol, 5 mol%), $(PhSe)_2$ (31.2 mg, 100 µmol, 10 mol%), $(4\text{-}ClPhS)_2$ (14.4 mg, 50.0 µmol, 5 mol%) and DCE (5 mL), 21 h. Crude ^{1}H NMR analysis: total yield: 72%; allylic isomer: 61%; allylic/vinylic isomer: 5:1.

TLC: R_f = 0.28 (PE/EtOAc, 4:1) [UV, $KMnO_4$]. IR [cm^{-1}]: 2974, 2933, 1718, 1662, 1461, 1368, 1312, 1275, 1238, 1185, 1040, 980, 805. **^{1}H NMR** (400 MHz, $CDCl_3$): δ 7.97 (d, *J* = 8.4 Hz, 0.43H), 7.87 (s, 0.57H), 7.39 (d, *J* = 8.6 Hz, 0.58H), 7.33 (d, *J* = 8.6 Hz, 0.57H), 7.27 – 7.21 (m, 1.31H), 7.19 (dd, *J* = 6.0, 3.2 Hz, 0.53H), 5.77 (dd, *J* = 15.8, 1.7 Hz, 0.97H), 5.44 – 5.33 (m, 1.00H), 4.23 – 4.14 (m, 2.03H), 2.58 – 2.51 (m, 3.08H), 2.50 – 2.36 (m, 1.09H), 2.34 – 2.20 (m, 1.05H), 1.29 – 1.24 (m, 3.33H), 0.96 – 0.89 (m, 3.09H). **^{13}C NMR** (101 MHz, $CDCl_3$): δ 165.6, 165.5, 146.6, 144.7, 143.9, 143.8, 138.2, 134.4, 133.0, 131.0, 129.7, 126.5, 123.5, 123.4, 119.7, 119.1, 109.2, 108.8, 62.1, 61.9, 60.8, 26.8, 22.0, 21.4, 14.1, 10.8, 10.7 **HRMS** (EI) calcd. for $[C_{15}H_{19}N_3O_2]^{\cdot+}$ ($[M]^{\cdot+}$), m/z = 273.1472; found 273.1468 and 273.1472.

Diethyl (*E*)-4-(1H-pyrazol-1-yl)hex-2-enedioate (3ax):

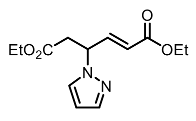

3ax

According to general procedure A: Diethyl (*E*)-hex-3-enedioate (**1a**) (604 mg, 3.02 mmol, 3.01 equiv.), 1H-pyrazole (**2h**) (68.2 mg, 1.00 mmol, 1.00 equiv.), TAPT (24.3 mg, 50.0 µmol, 5 mol%), $(PhSe)_2$ (31.4 mg, 101 µmol, 10 mol%), $(4\text{-}ClPhS)_2$ (14.5 mg, 50.3 µmol, 5 mol%) and DCE (5 mL), 21 h. After purification (PE/EtOAc, flash column), the product was isolated as a lightly yellow oil (228 mg, 857 µmol, 86%; allylic/vinylic isomer >49:1). Crude ^{1}H NMR analysis: yield of allylic isomer: 90%; allylic/vinylic isomer: >10:1.

TLC: R_f = 0.33 (PE/EtOAc, 4:1) [UV, $KMnO_4$]. IR [cm^{-1}]: 3124, 2982, 2937, 1722, 1662, 1398k, 1271, 1185, 1096, 1044, 980, 752. **^{1}H NMR** (400 MHz, $CDCl_3$): 7.56 (d, *J* = 1.7 Hz, 1H), 7.45 (d, *J* = 2.3 Hz, 1H), 7.06 (dd, *J* = 15.7, 5.8 Hz, 1H), 6.27 (t, *J* = 2.1 Hz, 1H), 5.69 (dd, *J* = 15.7, 1.6 Hz, 1H), 5.41 (dtd, *J* = 7.5, 5.9, 1.5 Hz, 1H), 4.20 – 4.06 (m, 4H), 3.24 (dd, *J* = 16.5, 8.5 Hz, 1H), 2.95 (dd, *J* = 16.5, 6.0 Hz, 1H), 1.25 (t, *J* = 7.1 Hz, 3H), 1.19 (t, *J* = 7.1 Hz, 3H) **^{13}C NMR** (101 MHz, $CDCl_3$): δ 169.8, 165.7, 144.5, 140.2, 129.4, 123.4, 105.9, 61.2, 60.9, 58.8, 38.8, 14.3, 14.2. **HRMS** (ESI) calcd. for $[C_{13}H_{19}N_2O_4]^+$ ($[M+H]^+$), m/z = 267.1339; found 267.1340.

Diethyl (*E*)-4-(4-methyl-1H-pyrazol-1-yl)hex-2-enedioate (3ay):

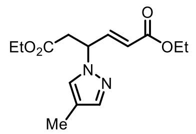

3ay

According to general procedure A: Diethyl (*E*)-hex-3-enedioate (**1c**) (603 mg, 3.01 mmol, 3.01 equiv.), 4-methyl-1H-pyrazole (**2i**) (77 µL, 82.1 mg, 1.00 mmol, 1.00 equiv.),TAPT (24.4 mg, 50.2 µmol, 5 mol%), $(PhSe)_2$ (31.2 mg, 100 µmol, 10 mol%),$(4\text{-}ClPhS)_2$ (14.4 mg, 50.0 µmol, 5 mol%) and DCE (5 mL), 21 h. After purification (PE/EtOAc, flash column), the product was isolated as a lightly yellow oil (157 mg, 560 µmol, 56%; allylic/vinylic isomer >49:1). Crude ^{1}H NMR analysis: yield of allylic isomer: 56%; allylic/vinylic isomer: >10:1.

TLC: R_f = 0.31 (PE/EtOAc, 4:1) [UV, $KMnO_4$]. IR [cm^{-1}]: 3090, 2982, 2937, 1722, 1662, 1372, 1312, 1271, 1159, 1029, 984, 861. **^{1}H NMR** (400 MHz, $CDCl_3$): δ 7.36 (s, 1H), 7.22 (s, 1H), 7.03 (dd, *J* = 15.7, 5.8 Hz, 1H), 5.69 (dd, *J* = 15.7, 1.6 Hz, 1H), 5.39 – 5.26 (m, 1H), 4.23 – 4.06 (m, 4H), 3.22 (dd, *J* = 16.4, 8.3 Hz, 1H), 2.92 (dd, *J* = 16.4, 6.0 Hz, 1H), 2.06 (s, 3H), 1.26 (t, *J* = 7.1 Hz, 3H), 1.20 (t, *J* = 7.2 Hz, 3H). **^{13}C NMR** (101 MHz, $CDCl_3$): δ 169.8, 165.7, 144.6, 140.4, 128.1, 123.1, 116.4, 61.1, 60.7, 58.5, 38.5, 14.2, 14.1, 8.8. **HRMS** (EI) calcd. for $[C_{14}H_{20}N_2O_4]^{\cdot+}$ ($[M]^{\cdot+}$), m/z = 280.1418; found 280.1414.

Diethyl (*E*)-4-(4-(trifluoromethyl)-1H-pyrazol-1-yl)hex-2-enedioate (3az):

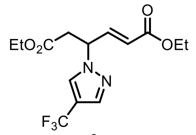

3az

According to general procedure A: Diethyl (*E*)-hex-3-enedioate (**1c**) (604 mg, 3.02 mmol, 2.99 equiv.), 4-(trifluoromethyl)-1H-pyrazole (**2j**) (137 mg, 1.01 mmol, 1.00 equiv.), TAPT (24.4 mg, 50.2 µmol, 5 mol%), $(PhSe)_2$ (31.2 mg, 100 µmol, 10 mol%) and DCE (5 mL), 21 h. After purification (PE/EtOAc, 90:10), the product was isolated as a lightly yellow oil (258 mg, 772 µmol, 77%; allylic/vinylic isomer >49:1). Crude ^{1}H NMR analysis: yield of allylic isomer: 89%; allylic/vinylic isomer: >10:1.

TLC: R_f = 0.32 (PE/EtOAc, 4:1) [UV, $KMnO_4$]. IR [cm^{-1}]: 3124, 2986, 1722, 1662, 1580, 1409, 1372, 1238, 1189, 1118, 1029, 969, 869, 686. **^{1}H NMR** (400 MHz, $CDCl_3$): δ 7.81 – 7.70 (m, 2H), 7.03 (dd, *J* = 15.7, 6.2 Hz, 1H), 5.77 (dd, *J* = 15.7, 1.5 Hz, 1H), 5.44 – 5.35 (m, 1H), 4.28 – 4.03 (m, 4H), 3.25 (dd, *J* = 16.7, 8.8 Hz, 1H), 2.94 (dd, *J* = 16.7, 5.5 Hz, 1H), 1.27 (t, *J* = 7.1 Hz, 3H), 1.19 (t, *J* = 7.1 Hz, 3H). **^{13}C NMR** (101 MHz, $CDCl_3$): δ 169.4, 165.4, 143.1, 137.8 (q, $J_{C\text{-}F}$ = 2.7 Hz), 129.1 (q, $J_{C\text{-}F}$ = 3.6 Hz), 124.2, 122.5 (q, $J_{C\text{-}F}$ = 267.1 Hz), 114.0 (q, $J_{C\text{-}F}$ = 38.6 Hz), 61.5, 61.1, 59.5, 38.6, 14.3, 14.1. **^{19}F NMR** (376 MHz, $CDCl_3$) δ -57.0. **HRMS** (ESI) calcd. for $[C_{14}H_{18}F_3N_2O_4]^+$ ($[M+H]^+$), m/z = 335.1213; found 335.1216.

Diethyl (*E*)-4-(4-(methoxycarbonyl)-1H-imidazol-1-yl)hex-2-enedioate (3ba):

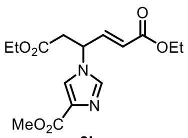

3ba

According to general procedure A: Diethyl (*E*)-hex-3-enedioate (**1c**) (601 mg, 3.00 mmol, 3.00 equiv.), methyl 1H-imidazole-4-carboxylate (**2k**) (126 mg, 1.00 mmol, 1.00 equiv.), TAPT (24.3 mg, 50.0 µmol, 5 mol%), $(PhSe)_2$ (31.2 mg, 100 µmol, 10 mol%), $(4\text{-ClPhS})_2$ (14.4 mg, 50.0 µmol, 5 mol%), DCE (3 mL) and HFIP (2 mL), 48 h. After purification (PE/EtOAc, 2:1 to 1:1), the product was isolated as a lightly yellow oil (183 mg, 564 µmol, 56%; allylic/vinyl isomer >49:1). Crude ^{1}H NMR analysis: yield of allylic isomer: 62%; allylic/vinylic isomer: >10:1.

According to general procedure A: Diethyl (*E*)-hex-3-enedioate (**1c**) (601 mg, 3.00 mmol, 3.00 equiv.), methyl 1H-imidazole-4-carboxylate (**2k**) (126 mg, 1.00 mmol, 1.00 equiv.), TAPT (24.3 mg, 50.0 µmol, 5 mol%), $(PhSe)_2$ (31.2 mg, 100 µmol, 10 mol%) and DCE (5.0 mL), 21 h. Crude ^{1}H NMR analysis: yield of allylic isomer: 5%; allylic/vinylic isomer: >10:1.

TLC: R_f = 0.23 (PE/EtOAc, 1:1) [UV, $KMnO_4$]. IR [cm^{-1}]: 124, 2982, 1711, 1662, 1539, 1465, 1364, 1308, 1267, 1215, 1182, 1111, 1025, 977, 917, 805, 768, 716. **^{1}H NMR** (300 MHz, $CDCl_3$): δ 7.80 – 7.64 (m, 2H), 7.04 (dd, *J* = 15.7, 5.6 Hz, 1H), 6.21 (td, *J* = 7.4, 1.5 Hz, 1H), 5.67 (dd, *J* = 15.6, 1.8 Hz, 1H), 4.18 – 4.03 (m, 4H), 3.81 (s, 3H), 3.13 – 2.91 (m, 2H), 1.22 (t, *J* = 7.2 Hz, 3H), 1.15 (t, *J* = 7.1 Hz, 3H). **^{13}C NMR** (75 MHz, $CDCl_3$): δ 168.9, 165.3, 160.5, 143.8, 140.3, 138.1, 123.5, 122.2, 61.3, 60.9, 53.5, 51.7, 38.9, 14.1, 14.0. **HRMS** (EI) calcd. for $[C_{15}H_{20}N_2O_6]^{\cdot+}$ ($[M]^{\cdot+}$), m/z = 324.1316; found 324.1314.

Diethyl (*E*)-4-(1H-1,2,3-triazol-1-yl)hex-2-enedioate (3bb):

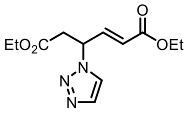

3bb

According to general procedure A: Diethyl (*E*)-hex-3-enedioate (**1c**) (601 mg, 3.00 mmol, 3.00 equiv.), 1H-1,2,3-triazole (**2m**) (69.1 mg, 1.00 mmol, 1.00 equiv.), TAPT (24.3 mg, 50.0 µmol, 5 mol%), (PhSe), $(4\text{-ClPhS})_2$ (14.4 mg, 50.0 µmol, 5 mol%), DCE (3 mL) and HFIP (2 mL), 48 h. After purification (PE/EtOAc, 4:1 to 2:1), the product was isolated as a lightly yellow oil (113 mg, 423 µmol, 42%; allylic/vinyl isomer 17:1). Crude ^{1}H NMR analysis: yield of allylic isomer: 48%; allylic/vinylic isomer: >10:1.

TLC: R_f = 0.12 (PE/EtOAc, 2:1) [UV, $KMnO_4$]. IR [cm^{-1}]: 3131, 2982, 2937, 1718, 1662, 1450, 1372, 1312, 1271, 1185, 1111, 1021, 977, 861, 790, 719. **^{1}H NMR** (300 MHz, $CDCl_3$): δ 7.67 (d, *J* = 1.0 Hz, 1H), 7.62 (d, *J* = 1.0 Hz, 1H), 7.02 (dd, *J* = 15.6, 6.3 Hz, 1H), 5.74 – 5.60 (m, 2H), 4.16 – 4.01 (m, 4H), 3.29 (dd, *J* = 16.8, 8.4 Hz, 1H), 3.01 (dd, *J* = 16.9, 5.8 Hz, 1H), 1.20 (t, *J* = 7.1 Hz, 3H), 1.14 (t, *J* = 7.1 Hz, 3H). **^{13}C NMR** (75 MHz, $CDCl_3$): δ 169.1, 165.2, 142.5, 133.8, 124.3, 123.7, 61.4, 61.0, 57.7, 38.6, 14.1, 14.0. **HRMS** (ESI) calcd. for $[C_{12}H_{18}N_3O_4]^+$ ($[M+H]^+$), m/z = 268.1292; found 268.1297.

Diethyl (*E*)-4-(1H-1,2,4-triazol-1-yl)hex-2-enedioate (3bc):

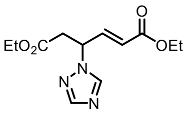

3bc

According to general procedure A: Diethyl (*E*)-hex-3-enedioate (**1c**) (601 mg, 3.00 mmol, 3.00 equiv.), 1H-1,2,4-triazole (**2l**) (69.1 mg, 1.00 mmol, 1.00 equiv.), TAPT (24.3 mg, 50.0 µmol, 5 mol%), $(PhSe)_2$ (31.2 mg, 100 µmol, 10 mol%), $(4\text{-ClPhS})_2$ (14.4 mg, 50.0 µmol, 5 mol%), DCE (3 mL) and HFIP (2 mL), 48 h. After purification (PE/EtOAc, 9:1), the product was isolated as a lightly yellow oil (148 mg, 553 µmol, 55%; allylic/vinylic isomer >49:1). Crude ^{1}H NMR analysis: yield of allylic isomer: 59%; allylic/vinylic isomer: >10:1.

According to general procedure A: diethyl (*E*)-hex-3-enedioate (600.7 mg, 3.00 mmol, 3.00 equiv.), 1H-1,2,4-triazole (69.1 mg, 1.00 mmol, 1.00 equiv.), TAPT (24.3 mg, 50.0 µmol, 5 mol%), $(PhSe)_2$ (31.2 mg, 100 µmol, 10 mol%) and DCE (5.0 mL), 21 h. yield of allylic isomer is 35%, allylic: vinyl isomer: >10:1.

TLC: R_f = 0.14 (PE/EtOAc, 1:1) [UV, $KMnO_4$]. IR [cm^{-1}]: 3124, 2982, 2937, 1718, 1662, 1506, 1446, 1372, 1312, 1275, 1234, 1163, 1141, 1021, 980, 861, 813, 719, 678. **^{1}H NMR** (300 MHz, $CDCl_3$): δ 8.16 (s, 1H), 7.95 (s, 1H), 7.02 (dd, *J* = 15.6, 6.2 Hz, 1H), 5.78 (dd, *J* = 15.7, 1.6 Hz, 1H), 5.58 – 5.37 (m, 1H), 4.20 – 4.03 (m, 4H), 3.20 (dd, *J* = 16.9, 8.9 Hz, 1H), 2.93

(dd, J = 16.8, 5.3 Hz, 1H), 1.24 (t, J = 7.1 Hz, 3H), 1.17 (t, J = 7.1 Hz, 3H). **^{13}C NMR** (75 MHz, $CDCl_3$): δ 169.2, 165.2, 152.4, 143.4, 142.5, 124.2, 61.4, 61.0, 56.7, 38.3, 14.1, 14.0. **HRMS** (APCI) calcd. for $[C_{12}H_{17}N_3O_4]^{\cdot+}$ ($[M]^{\cdot+}$), m/z = 267.1214; found 267.1211.

Diethyl (*E*)-4-(5-methyl-2H-tetrazol-2-yl)hex-2-enedioate (3bd):

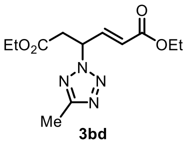

3bd

According to general procedure A: Diethyl (*E*)-hex-3-enedioate (**1c**) (601 mg, 3.00 mmol, 3.00 equiv.), 5-methyl-1H-tetrazole (**2n**) (84.0 mg, 1.00 mmol, 1.00 equiv.), TAPT (24.4 mg, 50.1 µmol, 5 mol%), $(PhSe)_2$ (31.4 mg, 100 µmol, 10 mol%), $(4\text{-}ClPhS)_2$ (14.4 mg, 50.0 µmol, 5 mol%) and DCE (5.0 mL), 21 h. After purification (PE/EtOAc, flash column), the product was isolated as a lightly yellow oil (154 mg, 547 µmol, 55%; allylic/vinylic isomer >49:1). Crude ^{1}H NMR analysis: yield of allylic isomer: 58%; allylic/vinylic isomer: >10:1.

TLC: R_f = 0.29 (PE/EtOAc, 4/1) [UV, $KMnO_4$]. IR [cm^{-1}]: 2982, 2941, 1722, 1662, 1506, 1372, 1312, 1271, 1185, 1025, 977. **^{1}H NMR** (400 MHz, $CDCl_3$): δ 7.01 (dd, J = 15.7, 6.7 Hz, 1H), 5.93 (dtd, J = 8.1, 6.5, 1.3 Hz, 1H), 5.81 (dd, J = 15.7, 1.4 Hz, 1H), 4.31 – 4.03 (m, 5H), 3.34 (dd, J = 16.8, 8.3 Hz, 1H), 3.07 (dd, J = 16.8, 6.3 Hz, 1H), 2.53 (s, 3H), 1.26 (t, J = 7.1 Hz, 3H), 1.20 (t, J = 7.1 Hz, 3H). **^{13}C NMR** (101 MHz, $CDCl_3$): δ 168.6, 165.1, 163.4, 141.2, 125.1, 61.6, 61.1, 60.4, 38.2, 14.2, 14.1, 11.1. **HRMS** (ESI) calcd. for $[C_{12}H_{19}N_4O_4]^+$ ($[M+H]^+$), m/z = 283.1401; found 283.1402.

Diethyl (*E*)-4-(5-phenyl-2H-tetrazol-2-yl)hex-2-enedioate (3be):

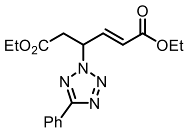

3be

According to general procedure A: Diethyl (*E*)-hex-3-enedioate (**1c**) (600 mg, 3.00 mmol, 3.00 equiv.), 5-phenyl-1H-tetrazole (**2o**) (146 mg, 1.00 mmol, 1.00 equiv.), TAPT (24.3 mg, 50.0 µmol, 5 mol%), $(PhSe)_2$ (31.4 mg, 100 µmol, 10 mol%), $(4\text{-}ClPhS)_2$ (14.5 mg, 50.4 µmol, 5 mol%) and DCE (5 mL), 21 h. After purification (PE/EtOAc, flash column), the product was isolated as a lightly yellow oil (220 mg, 639 µmol, 64%; allylic/vinylic isomer >49:1). Crude ^{1}H NMR analysis: yield of allylic isomer: 71%; allylic/vinylic isomer: >10:1.

TLC: R_f = 0.35 (PE/EtOAc, 4:1) [UV, $KMnO_4$]. IR [cm^{-1}]: 2982, 1722, 1662, 1450, 1372, 1316, 1271, 1185, 1025, 977, 734, 693. **^{1}H NMR** (400 MHz, $CDCl_3$): δ 8.27 – 8.05 (m, 2H), 7.57 – 7.40 (m, 3H), 7.09 (dd, J = 15.7, 6.7 Hz, 1H), 6.05 (dtd, J = 8.0, 6.5, 1.3 Hz, 1H), 5.89 (dd, J = 15.7, 1.4 Hz, 1H), 4.27 – 4.05 (m, 4H), 3.44 (dd, J = 16.8, 8.3 Hz, 1H), 3.16 (dd, J = 16.8, 6.3 Hz, 1H), 1.27 (t, J = 7.1 Hz, 3H), 1.21 (t, J = 7.1 Hz, 3H). **^{13}C NMR** (101 MHz, $CDCl_3$): δ 168.6, 165.5, 165.1, 141.1, 130.6, 129.0, 127.2, 127.1, 125.2, 61.7, 61.2, 60.8, 38.3, 14.3, 14.2. **HRMS** (ESI) calcd. for $[C_{17}H_{21}N_4O_4]^+$ ($[M+H]^+$), m/z = 345.1557; found 345.1562.

Diethyl (*E*)-4-(3-(methoxycarbonyl)-1H-indazol-1-yl)hex-2-enedioate (3bf):

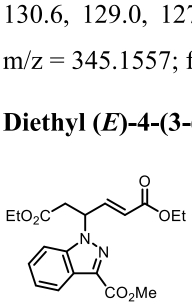

3bf

According to general procedure A: Diethyl (*E*)-hex-3-enedioate (**1c**) (601 mg, 3.00 mmol, 3.00 equiv.), methyl 1H-indazole-3-carboxylate (**2p**) (176 mg, 1.00 mmol, 1.00 equiv.), TAPT (24.3 mg, 50.0 µmol, 5 mol%), $(PhSe)_2$ (31.2 mg, 100 µmol, 10 mol%), $(4\text{-}ClPhS)_2$ (14.4 mg, 50.0 µmol, 5 mol%), DCE (3 mL) and HFIP (2 mL), 48 h. After purification (PE/EtOAc, 20:1 to 12:1), the product was isolated as a lightly yellow solid (327 mg, 874 µmol, 87%; allylic/vinylic isomer >49:1). Crude ^{1}H NMR analysis: yield of allylic isomer: 93%; allylic/vinylic isomer: >10:1.

TLC: R_f = 0.25 (PE/EtOAc, 5:1) [UV, $KMnO_4$]. **Mp** 70.5 °C. IR [cm^{-1}]: 2982, 1715, 1662, 1461, 1357, 1312, 1260, 1197, 1088, 1059, 917, 857, 779, 734, 664. **^{1}H NMR** (300 MHz, $CDCl_3$): δ 8.02 – 7.94 (m, 1H), 7.81 – 7.70 (m, 1H), 7.35 – 7.19 (m, 2H), 7.12 (dd, J = 15.6, 6.1 Hz, 1H), 7.04 – 6.92 (m, 1H), 5.65 (dd, J = 15.6, 1.4 Hz, 1H), 4.19 – 3.92 (m, 7H), 3.56 (dd, J = 16.9, 9.3 Hz, 1H), 3.07 (dd, J = 16.9, 5.4 Hz, 1H), 1.17 (t, J = 7.1 Hz, 3H), 1.07 (t, J = 7.1 Hz, 3H). **^{13}C NMR** (75 MHz,

$CDCl_3$): δ 169.5, 165.5, 160.5, 147.7, 144.4, 126.7, 125.3, 124.3, 123.3, 123.3, 121.6, 118.5, 60.9, 60.6, 58.1, 52.1, 38.3, 14.1, 13.9. **HRMS** (EI) calcd. for $[C_{19}H_{22}N_2O_6]^{\cdot+}$ ($[M]^{\cdot+}$), m/z = 374.1472; found 374.1467.

Diethyl (*E*)-4-(2-(methoxycarbonyl)-1H-benzo[d]imidazol-1-yl)hex-2-enedioate (3bg):

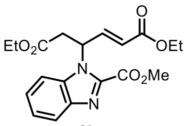

3bg

According to general procedure A: Diethyl (*E*)-hex-3-enedioate (**1c**) (601 mg, 3.00 mmol, 3.00 equiv.), methyl 1H-benzo[d]imidazole-2-carboxylate (**2q**) (176 mg, 1.00 mmol, 1.00 equiv.), TAPT (24.3 mg, 50.0 µmol, 5 mol%), $(PhSe)_2$ (31.2 mg, 100 µmol, 10 mol%), $(4\text{-}ClPhS)_2$ (14.4 mg, 50.0 µmol, 5 mol%), DCE (3 mL) and HFIP (2 mL), 48 h. After purification (PE/EtOAc, 5:1 to 2:1), the product was isolated as a colorless oil (285 mg, 761 µmol, 76%; allylic/vinylic isomer >49:1). Crude ^{1}H NMR analysis: yield of allylic isomer: 80%; allylic/vinyl isomer: >10:1.

TLC: R_f = 0.15 (PE/EtOAc, 2:1) [UV, $KMnO_4$]. IR [cm^{-1}]: 2982, 1715, 1659, 1491, 1439, 1390, 1312, 1260, 1178, 1025, 973, 947, 857, 742. **^{1}H NMR** (300 MHz, $CDCl_3$): δ 7.88 – 7.77 (m, 1H), 7.41 – 7.22 (m, 3H), 7.15 (dd, *J* = 15.8, 4.5 Hz, 1H), 6.91 – 6.77 (m, 1H), 5.74 (dd, *J* = 15.8, 2.2 Hz, 1H), 4.14 – 4.02 (m, 2H), 3.99 – 3.85 (m, 5H), 3.21 (dd, *J* = 16.6, 9.1 Hz, 1H), 3.08 (dd, *J* = 16.7, 5.8 Hz, 1H), 1.15 (t, *J* = 7.1 Hz, 3H), 0.99 (t, *J* = 7.1 Hz, 3H). **^{13}C NMR** (75 MHz, $CDCl_3$): δ 169.3, 165.4, 160.5, 143.1, 142.0, 140.9, 134.1, 125.8, 124.0, 123.7, 122.5, 112.5, 61.3, 60.9, 53.2, 37.2, 14.1, 13.9. **HRMS** (ESI) calcd. for $[C_{19}H_{22}N_2O_6]^+$ ($[M+H]^+$), m/z = 375.1551; found 375.1555.

Diethyl (*E*)-4-(1H-benzo[d][1,2,3]triazol-1-yl)hex-2-enedioate (3bh):

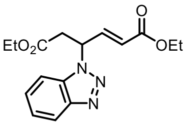

3bh

According to general procedure A: Diethyl (*E*)-hex-3-enedioate (**1c**) (602 mg, 3.01 mmol, 2.98 equiv.), 1H-benzo[d][1,2,3]triazole (**2f**) (120 mg, 1.01 mmol, 1.00 equiv.), TAPT (24.5 mg, 50.4 µmol, 5 mol%), $(PhSe)_2$ (31.4 mg, 101 µmol, 10 mol%), DCE (5 mL), 21 h. After purification (PE/EtOAc, 4:1), the product was isolated as a lightly yellow oil (242 mg, 763 µmol, 76%; allylic/vinylic isomer >49:1). Crude ^{1}H NMR analysis: yield of allylic isomer: 77%; allylic/vinylic isomer: >10:1.

According to general procedure A: Diethyl (*E*)-hex-3-enedioate (**1c**) (202 mg, 1.01 mmol, 1.00 equiv.), 1H-benzo[d][1,2,3]triazole (**2f**) (358 mg, 3.01 mmol, 2.98 equiv.), TAPT (24.5 mg, 50.3 µmol, 5 mol%), $(PhSe)_2$ (31.2 mg, 100 µmol, 10 mol%), DCE (5 mL), 21 h. After purification (PE/EtOAc, 4:1), the product was isolated as a lightly yellow oil (174 mg, 548 µmol, 55%; allylic/vinylic isomer >49:1). Crude ^{1}H NMR analysis: yield of allylic isomer: 52%; allylic/vinylic isomer: >10:1.

TLC: R_f = 0.25 (PE/EtOAc, 5:1) [UV, $KMnO_4$]. IR [cm^{-1}]: 3064, 2982, 2937, 1722, 1662, 1454, 1472, 1275, 1185, 1025, 749. **^{1}H NMR** (400 MHz, $CDCl_3$): δ 8.08 (d, *J* = 8.4 Hz, 1H), 7.62 – 7.46 (m, 2H), 7.43 – 7.33 (m, 1H), 7.14 (dd, *J* = 15.7, 6.0 Hz, 1H), 5.94 (dtd, *J* = 7.5, 6.0, 1.5 Hz, 1H), 5.72 (dd, *J* = 15.7, 1.5 Hz, 1H), 4.26 – 3.99 (m, 4H), 3.57 (dd, *J* = 16.8, 8.6 Hz, 1H), 3.24 (dd, *J* = 16.8, 6.0 Hz, 1H), 1.23 (t, *J* = 7.1 Hz, 3H), 1.14 (t, *J* = 7.1 Hz, 3H). **^{13}C NMR** (101 MHz, $CDCl_3$): δ 169.3, 165.2, 146.0, 142.6, 132.8, 127.8, 124.3, 124.1, 120.2, 109.4, 61.40, 60.9, 55.8, 38.2, 14.1, 14.0. **HRMS** (ESI) calcd. for $[C_{16}H_{20}N_3O_4]^+$ ($[M+H]^+$), m/z = 318.1448; found 318.1450.

Diethyl (*E*)-4-(4-oxoquinazolin-3(4H)-yl)hex-2-enedioate (3bi):

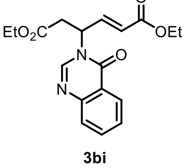

3bi

According to general procedure A: Diethyl (*E*)-hex-3-enedioate (**1c**) (601 mg, 3.00 mmol, 3.00 equiv.), quinazolin-4(3H)-one (**2r**) (146 mg, 1.00 mmol, 1.00 equiv.), TAPT (24.3 mg, 50.0 µmol, 5 mol%), $(PhSe)_2$ (31.2 mg, 100 µmol, 10 mol%), $(4\text{-}ClPhS)_2$ (14.4 mg, 50.0 µmol, 5 mol%), DCE (3 mL) and HFIP (2 mL), 48 h. After purification (PE/EtOAc, 5:1 to 2:1), the product was isolated as a lightly yellow oil (146 mg, 425 µmol, 42%; allylic/vinylic isomer >49:1). Crude ^{1}H NMR analysis: yield of allylic isomer: 45%; allylic/vinylic isomer: >10:1.

TLC: R_f = 0.16 (PE/EtOAc, 2:1) [UV, $KMnO_4$]. IR [cm^{-1}]: 3068, 2982, 2937, 1722, 1670, 1607, 1476, 1416, 1372, 1316, 1245, 1159, 1096, 1025, 917, 869, 775, 731, 701. **^{1}H NMR** (300 MHz, $CDCl_3$): δ 8.30 – 8.14 (m, 1H), 8.03 (s, 1H), 7.76 – 7.59 (m, 2H), 7.52 – 7.38 (m, 1H), 7.14 (dd, *J* = 15.8, 5.6 Hz, 1H), 5.92 (dd, *J* = 15.8, 1.8 Hz, 1H), 5.58 (dtd, *J* = 7.7, 5.8, 1.8 Hz, 1H), 4.13 (q, *J* = 7.1 Hz, 2H), 4.08 – 3.99 (m, 2H), 3.26 (dd, *J* = 16.9, 8.4 Hz, 1H), 3.02 (dd, *J* = 16.7, 5.9 Hz, 1H), 1.21 (t, *J* = 7.1 Hz, 3H), 1.11 (t, *J* = 7.1 Hz, 3H). **^{13}C NMR** (75 MHz, $CDCl_3$): δ 169.6, 165.3, 160.5, 147.5, 145.4, 142.8, 134.6, 127.5, 126.8, 124.1, 122.0, 61.3, 60.9, 54.6, 36.7, 14.1, 14.0. **HRMS** (EI) calcd. for $[C_{18}H_{20}N_2O_5]^{\cdot+}$ ($[M]^{\cdot+}$), m/z = 344.1367; found 344.1365.

Diethyl (E)-4-(4-oxobenzo[d][1,2,3]triazin-3(4H)-yl)hex-2-enedioate (3bj):

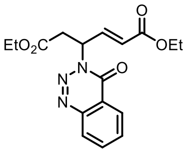

3bj

According to general procedure A: Diethyl (*E*)-hex-3-enedioate (**1c**) (601 mg, 3.00 mmol, 3.00 equiv.), benzo[d][1,2,3]triazin-4(3H)-one (**2s**) (147 mg, 1.00 mmol, 1.00 equiv.), TAPT (24.3 mg, 50.0 μmol, 5 mol%), $(PhSe)_2$ (31.2 mg, 100 μmol, 10 mol%), $(4\text{-}ClPhS)_2$ (14.4 mg, 50.0 μmol, 5 mol%), DCE (3 mL) and HFIP (2 mL), 48 h. After purification (PE/EtOAc, 2:1 to 0:1 to afford the crude product, then $CHCl_3$/THF, 10:1), the product was isolated as a lightly yellow oil (158 mg, 458 μmol, 46%; allylic/vinylic isomer >49:1). Crude ^{1}H NMR analysis: yield of allylic isomer: 55%; allylic/vinylic isomer: >10:1.

TLC: R_f = 0.75 ($CHCl_3$/THF, 10:1) [UV, $KMnO_4$]. IR [cm^{-1}]: 3071, 2982, 1722, 1685, 1610, 1461, 1372, 1297, 1267, 1178, 1096, 1070, 1025, 977, 939, 857, 779, 730, 686. **^{1}H NMR** (300 MHz, $CDCl_3$): δ 8.31 (ddd, *J* = 8.0, 1.5, 0.5 Hz, 1H), 8.12 (ddd, *J* = 8.1, 1.1, 0.5 Hz, 1H), 7.93 (ddd, *J* = 8.2, 7.3, 1.4 Hz, 1H), 7.78 (ddd, *J* = 8.0, 7.3, 1.2 Hz, 1H), 7.08 (dd, *J* = 15.8, 6.6 Hz, 1H), 6.30 – 6.13 (m, 1H), 5.94 (dd, *J* = 15.8, 1.4 Hz, 1H), 4.18 – 3.99 (m, 4H), 3.36 (dd, *J* = 16.6, 8.9 Hz, 1H), 3.10 (dd, *J* = 16.6, 6.0 Hz, 1H), 1.21 (t, *J* = 7.1 Hz, 3H), 1.12 (t, *J* = 7.1 Hz, 3H). **^{13}C NMR** (75 MHz, $CDCl_3$): δ 169.4, 165.5, 154.9, 143.7, 143.1, 135.2, 132.7, 128.4, 125.4, 124.2, 119.5, 61.1, 60.8, 54.5, 37.3, 14.2, 14.0. **HRMS** (ESI) calcd. for $[C_{17}H_{20}N_3O_5]^+$ ($[M+H]^+$), m/z = 346.1397; found 346.1403.

(8R,9S,10R,13S,14S,17S)-10,13-dimethyl-3-oxo-2,3,6,7,8,9,10,11,12,13,14,15,16,17-tetradecahydro-1H-cyclopenta[a]phenanthren-17-yl (E)-4-(4-chloro-1H-pyrazol-1-yl)hex-2-enoate (3bk):

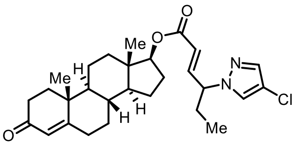

3bk

According to general procedure A: (8*R*,9*S*,10*R*,13*S*,14*S*,17*S*)-10,13-dimethyl-3-oxo-2,3,6,7,8,9,10,11,12,13,14,15,16,17-tetradecahydro-1H-cyclopenta[a]phenanthren-17-yl (*E*)-hex-3-enoate (**1ap**) (385 mg, 1.00 mmol, 1.00 equiv.), 4-chloro-1H-pyrazole (**2a**) (308 mg, 3.00 mmol, 3.00 equiv.), TAPT (24.3 mg, 50.0 μmol, 5 mol%), $(PhSe)_2$ (31.2 mg, 100 μmol, 10 mol%), $(4\text{-}ClPhS)_2$ (14.4 mg, 50.0 μmol, 5 mol%) and DCE (5 mL), 21 h. After purification (DCM/THF, 40:1 to 20:1), the product was isolated as a colorless oil (359 mg, 740 μmol, 74%; allylic/vinylic isomer 11:1). Further purification of the product with second column afforded the analytic data of purely allylic compound. Crude ^{1}H NMR analysis: yield of allylic isomer: 76%; allylic/vinyl isomer: >10:1.

TLC: R_f = 0.54 (DCM/THF, 20:1) [UV, $KMnO_4$]. IR [cm^{-1}]: 3124, 2937, 2878, 1715, 1662, 1618, 1435, 1387, 1312, 1275, 1230, 1182, 1133, 1044, 969, 910, 865, 835, 783. **^{1}H NMR** (300 MHz, $CDCl_3$): δ 7.44 (d, *J* = 0.5 Hz, 1H), 7.39 (s, 1H), 6.95 (ddd, *J* = 15.6, 6.1, 0.9 Hz, 1H), 5.76 – 5.63 (m, 2H), 4.76 – 4.56 (m, 2H), 2.46 – 1.88 (m, 8H), 1.86 – 1.46 (m, 7H), 1.40 – 0.93 (m, 9H), 0.89 – 0.83 (m, 3H), 0.80 (s, 3H). **^{13}C NMR** (75 MHz, $CDCl_3$): δ 199.4, 170.9, 165.7, 145.0, 138.0, 126.3, 123.9, 123.0, 122.9, 110.2, 82.9, 82.8, 65.2, 53.7, 50.2, 42.6, 38.6, 36.6, 35.7, 35.4, 33.9, 32.7, 31.5, 27.5, 27.2, 23.5, 20.5, 17.4, 12.1, 10.5. **HRMS** (ESI) calcd. for $[C_{28}H_{38}ClN_2O_3]^+$ ($[M+H]^+$), m/z = 485.2565; found 485.2573.

(8*R*,9*S*,10*R*,13*S*,14*S*,17*S*)-10,13-Dimethyl-3-oxo-2,3,6,7,8,9,10,11,12,13,14,15,16,17-tetradecahydro-1H-cyclopenta[a]phenanthren-17-yl (*E*)-4-(1H-benzo[d][1,2,3]triazol-1-yl)hex-2-enoate (3bl):

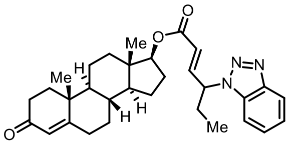

3bl

According to general procedure A: (8*R*,9*S*,10*R*,13*S*,14*S*,17*S*)-10,13-dimethyl-3-oxo-2,3,6,7,8,9,10,11,12,13,14,15,16,17-tetradecahydro-1H-cyclopenta[a]phenanthren-17-yl (*E*)-hex-3-enoate (**1ap**) (385 mg, 1.00 mmol, 1.00 equiv.), 1H-benzo[d][1,2,3]triazole (**2f**) (357 mg, 3.00 mmol, 3.00 equiv.), TAPT (24.3 mg, 50.0 μmol, 5 mol%), $(PhSe)_2$ (31.2 mg, 100 μmol, 10 mol%), $(4\text{-ClPhS})_2$ (14.4 mg, 50.0 μmol, 5 mol%) and DCE (5 mL), 21 h. After purification (PE/THF, 5:1), the product was isolated as a colorless solid (291 mg, 580 μmol, 58%; allylic/vinylic isomer >49:1). Crude ^{1}H NMR analysis: yield of allylic isomer: 62%; allylic/vinylic isomer: >10:1.

TLC: R_f = 0.23 (PE/EtOAc, 2:1) [UV, $KMnO_4$]. IR [cm^{-1}]: 3056, 2937, 2878, 1718, 1666, 1614, 1491, 1454, 1379, 1353, 1312, 1271, 1234, 1182, 1133, 1066, 1040, 977, 865, 824, 783, 734. **^{1}H NMR** (300 MHz, $CDCl_3$): δ 8.17 – 8.01 (m, 1H), 7.52 – 7.45 (m, 2H), 7.44 – 7.36 (m, 1H), 7.18 (dd, *J* = 15.7, 6.0 Hz, 1H), 5.83 – 5.73 (m, 1H), 5.73 – 5.68 (m, 1H), 5.50 – 5.32 (m, 1H), 4.69 – 4.55 (m, 1H), 2.52 – 1.94 (m, 8H), 1.88 – 1.46 (m, 7H), 1.41 – 0.93 (m, 9H), 0.93 – 0.87 (m, 3H), 0.82 – 0.73 (m, 3H). **^{13}C NMR** (75 MHz, $CDCl_3$): δ 199.4, 170.9, 165.5, 146.1, 143.7, 132.6, 127.6, 124.2, 123.9, 123.6, 120.3, 109.6, 83.0, 62.0, 53.6, 50.2, 42.7, 42.6, 38.6, 36.6, 35.7, 35.4, 33.9, 32.7, 31.5, 27.5, 27.0, 23.5, 20.5, 17.4, 12.1, 10.8. **HRMS** (ESI) calcd. for $[C_{31}H_{40}ClN_3O_3]^+$ ($[M+H]^+$), m/z = 502.3064; found 502.3064.

Diethyl (*E*)-4-(5-(4'-((2-butyl-4-oxo-1,3-diazaspiro[4.4]non-1-en-3-yl)methyl)-[1,1'-biphenyl]-2-yl)-2H-tetrazol-2-yl)hex-2-enedioate (3bm):

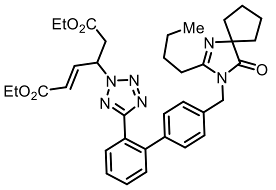

3bm

According to general procedure A: diethyl (*E*)-hex-3-enedioate (**1c**) (601 mg, 3.00 mmol, 3.00 equiv.), Irbesartan (**2u**) (429 mg, 1.00 mmol, 1.00 equiv.), TAPT (24.3 mg, 50.0 μmol, 5 mol%), $(PhSe)_2$ (31.2 mg, 100 μmol, 10 mol%), $(4\text{-ClPhS})_2$ (14.4 mg, 50.0 μmol, 5 mol%), DCE (3 mL) and HFIP (2 mL), 48 h. After purification (PE/EtOAc, 2:1), the product was isolated as a colorless oil (219 mg, 349 μmol, 35%; allylic/vinylic isomer >49:1). Crude ^{1}H NMR analysis: yield of allylic isomer: 53%; the ratio of allylic and vinylic isomer could not be detected.

TLC: R_f = 0.07 (PE/EtOAc, 2:1) [UV, $KMnO_4$]. IR [cm^{-1}]: 2960, 2870, 1722, 1662, 1633, 1513, 1461, 1439, 1394, 1342, 1267, 1178, 1096, 1025, 977, 913, 861, 813, 731. **^{1}H NMR** (300 MHz, $CDCl_3$): δ 7.90 – 7.81 (m, 1H), 7.58 – 7.43 (m, 2H), 7.42 – 7.37 (m, 1H), 7.16 – 7.04 (m, 4H), 6.90 (dd, *J* = 15.8, 6.6 Hz, 1H), 5.95 – 5.82 (m, 1H), 5.71 (dd, *J* = 15.6, 1.4 Hz, 1H), 4.68 (s, 2H), 4.29 – 4.04 (m, 4H), 3.15 (dd, *J* = 16.7, 8.2 Hz, 1H), 2.95 (dd, *J* = 16.8, 6.4 Hz, 1H), 2.40 – 2.25 (m, 2H), 2.04 – 1.73 (m, 8H), 1.64 – 1.49 (m, 2H), 1.41 – 1.23 (m, 5H), 1.19 (t, *J* = 7.2 Hz, 3H), 0.85 (t, *J* = 7.3 Hz, 3H). **^{13}C NMR** (75 MHz, $CDCl_3$): δ 186.7, 168.3, 165.4, 164.9, 161.9, 141.5, 141.0, 140.4, 135.5, 130.8, 130.3, 130.3, 129.7, 127.7, 126.5, 125.8, 124.9, 61.5, 61.0, 60.4, 43.3, 38.0, 37.4, 28.8, 27.8, 26.1, 22.3, 14.2, 14.1, 13.8. **HRMS** (ESI) calcd. for $[C_{35}H_{43}N_6O_5]^+$ ($[M+H]^+$), m/z = 627.7289; found 627.3297.

Diethyl (*E*)-4-((1S,3aS,3bR,5aS,10aS,10bS,12aS)-1-hydroxy-1,10a,12a-trimethyl-2,3,3a,3b,4,5,5a,6,10,10a,10b,11,12,12a-tetradecahydrocyclopenta[5,6]naphtho[1,2-f]indazol-8(1H)-yl)hex-2-enedioate (*N*1 isomer) and diethyl (*E*)-4-((1S,3aS,3bR,5aS,10aS,10bS,12aS)-1-hydroxy-1,10a,12a-trimethyl-2,3,3a,3b,4,5,5a,6,10,10a,10b,11,12,12a-tetradecahydrocyclopenta[5,6]naphtho[1,2-f]indazol-7(1H)-yl)hex-2-enedioate (*N*2 isomer) (3bn):

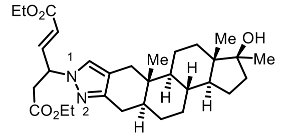

3bn (*N*1-isomer)

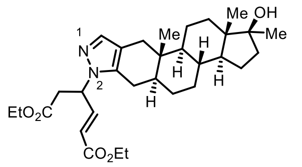

3bn (*N*2-isomer)

According to general procedure A: Diethyl (*E*)-hex-3-enedioate (**1c**) (600 mg, 3.00 mmol, 3.00 equiv.), Stanozolol (**2v**) (329 mg, 1.00 mmol, 1.00 equiv.), TAPT (24.3 mg, 50.1 µmol, 5 mol%), $(PhSe)_2$ (31.4 mg, 101 µmol, 10 mol%), $(4\text{-}ClPhS)_2$ (14.3 mg, 49.9 µmol, 5 mol%), DCE (3 mL) and HFIP (2 mL), 48 h. After purification (PE/EtOAc, flash column), the product was isolated as a colourless oil in three fractions: F1 (188 mg, 347 µmol, 36%, N1), F2 (29.8 mg, 56.6 µmol, 6%, *N*1:*N*2 = 1:6) and F3 (34.8 mg, 66.1 µmol, 7%, *N*1:*N*2 = 1:1). Total: 252 mg, 479 µmol, 48%, *N*1:*N*2 = 5:1; allylic/vinylic isomer >49:1, 1:1 d.r. Crude ^1H NMR analysis: yield of allylic isomer: 51%; the ratio of allylic and vinylic isomer could not be detected.

***N*1 isomer: TLC:** R_f = 0.39 (DCM/CH_3CN, 10:1) [UV]. IR [cm^{-1}]: 2922, 2851, 2251, 1718, 1372, 1271, 1156, 1096, 1029, 980, 910, 727. **^{1}H NMR** (400 MHz, $CDCl_3$): δ7.08 (s, 1H), 7.02 (dd, *J* = 15.7, 6.0 Hz, 1H), 5.72 (ddd, *J* = 15.8, 4.3, 1.4 Hz, 1H), 5.34 – 5.19 (m, 1H), 4.21 – 3.98 (m, 4H), 3.15 (ddd, *J* = 16.3, 7.9, 3.7 Hz, 1H), 2.89 (ddd, *J* = 16.3, 6.6, 2.6 Hz, 1H), 2.67 – 2.47 (m, 2H), 2.28 – 2.16 (m, 1H), 2.05 (d, *J* = 15.2 Hz, 1H), 1.85 – 1.65 (m, 3H), 1.64 – 1.27 (m, 10H), 1.27 – 1.21 (m, 4H), 1.21 – 1.13 (m, 7H), 0.94 – 0.77 (m, 5H), 0.74 – 0.70 (m, 3H). **^{13}C NMR** (101 MHz, $CDCl_3$): δ 169.9, 165.8, 148.7, 148.6, 144.9, 126.9, 126.7, 123.1, 123.0, 115.6, 81.7, 61.0, 60.7, 58.5, 53.9, 53.8, 50.6, 45.4, 42.6, 42.5, 39.0, 38.8, 36.7, 36.3, 34.8, 31.7, 31.5, 29.3, 27.6, 25.8, 23.3, 20.8, 14.2, 14.1, 13.9, 11.6, 11.5. **HRMS** (ESI) calcd. for $[C_{31}H_{47}N_2O_5]^+$ ($[M+H]^+$), m/z = 527.3479; found 527.3488.

***N*2 isomer (containing little *N*1 isomer): TLC:** R_f = 0.26 (DCM/CH_3CN, 10:1) [UV]. IR [cm^{-1}]: 3511, 2922, 1722, 1659, 1446, 1372, 1308, 1271, 1182, 1029, 936. **^{1}H NMR** (400 MHz, $CDCl_3$): δ 7.28 (s, 0.80H), 7.12 (s, 0.12H), 7.08 – 6.93 (m, 1.00H), 5.75 (dd, *J* = 15.6, 4.2 Hz, 0.13H), 5.60 (d, *J* = 15.6 Hz, 0.37H), 5.47 (d, *J* = 15.6 Hz, 0.49H), 5.35 – 5.14 (m, 0.99H), 4.21 – 4.00 (m, 4.00H), 3.44 – 3.28 (m, 0.89H), 3.18 (dd, *J* = 16.2, 6.5 Hz, 0.17H), 2.98 – 2.86 (m, 1.02H), 2.66 – 2.48 (m, 1.69H), 2.43 (dd, *J* = 16.2, 4.9 Hz, 0.42H), 2.34 – 2.18 (m, 0.57H), 2.14 – 2.01 (m, 1.53H), 1.88 – 1.10 (m, 24.00H+peak of water), 0.98 – 0.76 (m, 5.15H), 0.76 – 0.66 (m, 3.02H). **^{13}C NMR** (101 MHz, $CDCl_3$): δ 170.2, 170.1, 165.8, 145.1, 144.9, 138.1, 138.1, 137.6, 122.8, 122.6, 115.5, 115.3, 81.7, 61.0, 61.0, 60.7, 55.0, 54.9, 53.8, 53.7, 50.5, 45.4, 42.2, 42.1, 39.0, 38.1, 37.9, 36.6, 36.4, 35.0, 31.6, 31.4, 29.7, 29.2, 25.8, 25.7, 25.6, 23.3, 20.8, 14.2, 14.1, 13.9, 11.5, 11.4. **HRMS** (ESI) calcd. for $[C_{31}H_{47}N_2O_5]^+$ ($[M+H]^+$), m/z = 527.3479; found 527.3489 and 527.3488.

General procedure for the isomerization of the N-allylic products:

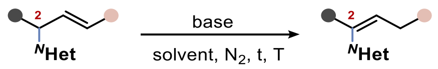

3 3''

General procedure B: *N*-allylic product (1.00 equiv.) was dissolved in THF (0.05 M). At 0 °C, LiO*t*Bu (2.2 M in THF, 5.00 equiv.) was added dropwise under N_2 atmosphere. The resulting mixture was then continued to react at 0 °C for 1 h. After the reaction was finished, saturated NH_4Cl (aq., 1 mL) was added at 0 °C to quench the excess LiO*t*Bu, followed by the addition of H_2O (20 mL) and DCM (20 mL). The two phases were separated, and the aqueous phase was extracted with DCM (2 x 20 mL). The organic phases were combined and the solvent was removed under reduced pressure. The ^{1}H NMR-yield of the residue was determined with internal standard 1,4-dimethoxybenzene. Further purification via column chromatography provided the target compound.

General procedure C: *N*-allylic product (1.00 equiv.) was dissolved in DCE (0.05 M). At r.t., DBU (or TMG) (2.00 equiv.) was added under N_2 atmosphere. The resulting mixture was then continued to react at the given temperature. After the reaction was finished, AcOH (0.05 mL) was added to quench the reaction. The solvent was removed under reduced pressure. The ^{1}H NMR-yield of the residue was determined with internal standard 1,4-dimethoxybenzene. Further purification via column chromatography provided the target compound.

Ethyl 4-(4-chloro-1H-pyrazol-1-yl)hex-3-enoate (3a''):

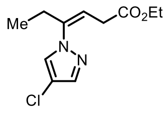

3a''

According to general procedure B: Ethyl (*E*)-4-(4-chloro-1H-pyrazol-1-yl)hex-2-enoate (**3a**) (48.5 mg, 200 μmol, 1.00 equiv.), LiO*t*Bu (2.2 M in THF, 455 μL, 5.00 equiv.), THF (4 mL), 0 °C, 1 h. After purification (PE/EtOAc, 30:1 to 15:1), the product was isolated as a colorless oil (40.5 mg, 167 μmol, 88%, *E*/*Z* = 1:1; vinylic/allylic isomer >49:1,). Crude ^{1}H NMR analysis: yield of vinylic isomer: 86%; *E*/*Z* = 1:1; vinylic/allylic isomer: 49:1.

TLC: R_f = 0.58 (PE/EtOAc, 5:1) [UV, $KMnO_4$]. IR [cm^{-1}]: 3135, 2978 2937, 1733, 1677, 1528, 1461, 1420, 1338, 1301, 1256, 1170, 1096, 1029, 969, 842, 790, 734. **^{1}H NMR** (300 MHz, $CDCl_3$): δ 7.63 (d, *J* = 0.7 Hz, 0.57H), 7.56 – 7.47 (m, 1.38H), 5.84 (t, *J* = 7.5 Hz, 0.57H), 5.60 (tt, *J* = 7.4, 1.2 Hz, 0.41H), 4.21 – 4.09 (m, 2.02H), 3.21 (d, *J* = 7.5 Hz, 1.14H), 3.10 (dt, *J* = 7.4, 1.2 Hz, 0.83H), 2.65 (q, *J* = 7.5 Hz, 1.20H), 2.52 (qd, *J* = 7.3, 1.2 Hz, 0.88H), 1.30 – 1.22 (m, 3.19H), 1.07 – 0.97 (m, 3.00H).. **^{13}C NMR** (75 MHz, $CDCl_3$): δ 171.2, 171.0, 142.8, 142.7, 138.6, 138.5, 127.8, 125.5, 114.4, 111.1, 110.6, 109.9, 61.1, 61.0, 32.9, 32.7, 28.8, 21.6, 14.2, 12.2, 11.6. **HRMS** (APCI) calcd. for $[C_{11}H_{16}ClN_2O_2]^+$ ($[M+H]^+$), m/z = 243.0895; found 243.0895 and 243.0896.

Pent-3-yn-1-yl 4-(4-chloro-1H-pyrazol-1-yl)hex-3-enoate (3w''):

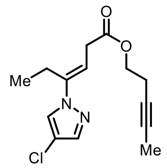

3w''

According to general procedure B: Pent-3-yn-1-yl (*E*)-4-(4-chloro-1H-pyrazol-1-yl)hex-2-enoate (**3w**) (56.2 mg, 200 μmol, 1.00 equiv.), LiO*t*Bu (2.2 M in THF, 455 μL, 5.00 equiv.), THF (4 mL), 0 °C, 1 h. After purification (PE/EtOAc, 30:1 to 15:1), the product was isolated as a colorless oil (46.4 mg, 165 μmol, 83%, *E*/*Z* = 2:1; vinylic/allylic isomer >49:1,). Crude ^{1}H NMR analysis: yield of vinylic isomer: 83%; *E*/*Z* = 2:1; vinylic/allylic: isomer: 47:1.

TLC: R_f = 0.54 (PE/EtOAc, 5:1) [UV, $KMnO_4$]. IR [cm^{-1}]: 3135, 2971, 2922, 1737, 1677, 1528, 1461, 1424, 1338, 1301, 1245, 1167, 1014, 969, 895, 842, 794. **^{1}H NMR** (300 MHz, $CDCl_3$): δ 7.64 (d, *J* = 0.7 Hz, 0.63H), 7.56 – 7.46 (m, 1.31H), 5.83 (t, *J* = 7.5 Hz, 0.61H), 5.60 (tt, *J* = 7.4, 1.2 Hz, 0.36H), 4.15 (q, *J* = 6.8 Hz, 2.00H), 3.23 (d, *J* = 7.5 Hz, 1.26H), 3.13 (dt, *J* = 7.3, 1.0 Hz, 0.73H), 2.65 (q, *J* = 7.5 Hz, 1.27H), 2.57 – 2.41 (m, 2.84H), 1.79 – 1.72 (m, 3.07H), 1.07 – 0.96 (m, 3.02H).

^{13}C NMR (75 MHz, $CDCl_3$): δ 171.0, 170.7, 142.9, 142.8, 138.6, 127.8, 125.6, 114.1, 111.1, 110.6, 109.7, 74.6, 63.4, 63.3, 32.8, 32.6, 28.8, 21.6, 19.2, 12.2, 11.6, 3.5. **HRMS** (APCI) calcd. for $[C_{14}H_{18}ClN_2O_2]^+$ ($[M+H]^+$), m/z = 281.1051; found 281.1053 and 281.1055.

Ethyl 4-(1H-benzo[d][1,2,3]triazol-1-yl)hex-3-enoate (3av''):

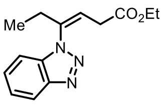

3av''

According to general procedure B: Ethyl (*E*)-4-(1H-benzo[d][1,2,3]triazol-1-yl)hex-2-enoate (**3av**) (51.9 mg, 200 μmol, 1.00 equiv.), LiO*t*Bu (2.2 M in THF, 455 μL, 5.00 equiv.), THF (4 mL), 0 °C, 1 h. After purification (PE/EtOAc, 30:1 to 12:1), the product was isolated as a colorless oil (43.9 mg, 169 μmol, 85%, *E*/*Z* = 1:2; vinylic/allylic isomer >49:1,). Crude ^{1}H NMR analysis: yield of vinylic isomer: 84%; *E*/*Z* = 1:1; vinylic/allylic: isomer: 46:1.

TLC: R_f = 0.33 (PE/EtOAc, 5:1) [UV, $KMnO_4$]. IR [cm^{-1}]: 2956, 2859, 1730, 1461, 1387, 1252, 1156, 1088, 969, 895, 835, 775, 746, 731. **^{1}H NMR** (300 MHz, $CDCl_3$): δ 8.13 – 8.02 (m, 0.97H), 7.75 – 7.65 (m, 0.40H), 7.55 – 7.44 (m, 1.00H), 7.43 – 7.33 (m, 1.60H), 6.13 – 5.96 (m, 0.97H), 4.22 (q, *J* = 7.1 Hz, 0.79H), 4.07 (q, *J* = 7.1 Hz, 1.21H), 3.39 (d, *J* = 7.5 Hz, 0.76H), 2.95 – 2.77 (m, 2.01H), 2.68 (qd, *J* = 7.4, 1.4 Hz, 1.26H), 1.31 (t, *J* = 7.1 Hz, 1.20H), 1.17 (t, *J* = 7.1 Hz, 1.94H), 1.05 – 0.95 (m, 3.00H). **^{13}C NMR** (75 MHz, $CDCl_3$): δ 170.8, 170.6, 145.9, 145.4, 140.5, 139.4, 133.1, 132.9, 128.0, 127.9, 124.2, 120.2, 120.0, 116.4, 110.9, 109.9, 61.3, 61.0, 32.8, 32.7, 29.3, 23.6, 14.2, 14.1, 11.9, 11.6. **HRMS** (APCI) calcd. for $[C_{14}H_{18}N_3O_2]^+$ ($[M+H]^+$), m/z = 260.1394; found 260.1394 and 260.1397.

4-Chloro-1-(4-(phenylsulfonyl)but-2-en-2-yl)-1H-pyrazole (3ae''):

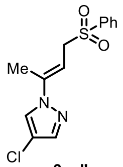

3ae''

According to general procedure C: (E)-4-chloro-1-(4-(phenylsulfonyl)but-3-en-2-yl)-1H-pyrazole (**3ae**) (35.8 mg, 120 μmol, 1.00 equiv.), DBU (36.5 mg, 33.8 μL, 2.00 equiv.), DCE (2.4 mL), r.t., 5 h. After purification (PE/EtOAc, 10:1 to 6:1), the product was isolated as a colorless solid (33.0 mg, 110 μmol, 93%, *E*/*Z* = 4:1; vinylic/allylic isomer >49:1,). Crude ^{1}H NMR analysis: yield of vinylic isomer: 90%; *E*/*Z* = 4:1; vinylic/allylic: isomer: >49:1.

TLC: R_f = 0.33 (PE/EtOAc, 2:1) [UV, $KMnO_4$]. **Mp** 111.9 °C. IR [cm^{-1}]: 3146, 3056, 3001, 2952, 2926, 1673, 1584, 1480, 1446, 1409, 1349, 1301, 1152, 1085, 1025, 965, 872, 794, 716, 690. **^{1}H NMR** (300 MHz, $CDCl_3$): δ 7.93 – 7.77 (m, 2.00H), 7.72 – 7.47 (m, 4.70H), 7.46 – 7.41 (m, 0.24H), 7.31 – 7.27 (m, 0.24H), 5.84 (td, *J* = 8.4, 1.1 Hz, 0.79H), 5.37 (td, *J* = 8.1, 1.3 Hz, 0.20H), 4.25 (dd, *J* = 8.0, 1.0 Hz, 0.40H), 3.95 (d, *J* = 8.5 Hz, 1.59H), 2.15 (d, *J* = 1.1 Hz, 0.62H), 1.93 (s, 2.38H).. **^{13}C NMR** (75 MHz, $CDCl_3$): δ 141.3, 139.7, 139.3, 138.9, 138.6, 138.4, 134.1, 133.8, 129.4, 129.1, 128.4, 128.2, 126.9, 124.8, 112.1, 107.1, 102.4, 55.6, 55.0, 22.2, 14.0. **HRMS** (ESI) calcd. for $[C_{13}H_{14}ClN_2O_2S]^+$ ($[M+H]^+$), m/z = 297.0459; found 297.0461 and 297.0464.

(*E*)-4-(4-Chloro-1H-pyrazol-1-yl)pent-3-enenitrile (3ag''):

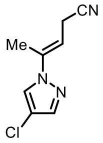

3ag''

According to general procedure C: 4-(4-Chloro-1H-pyrazol-1-yl)pent-2-enenitrile (**3ag**, *E*/*Z* = 6:1) (36.3 mg, 200 μmol, 1.00 equiv.), DBU (60.9 mg, 56.4 μL, 2.00 equiv.), DCE (4 mL), r.t., 5 h. After purification (PE/EtOAc, 10:1 to 6:1), the product was isolated as a colorless oil (26.8 mg, 148 μmol, 74%, *E*; vinylic/allylic isomer: 10:1). Crude ^{1}H NMR analysis: yield of vinylic isomer: 86%; *E*/*Z* = 4:1; vinylic/allylic: isomer: 10:1.

TLC: R_f = 0.47 (PE/EtOAc, 2:1) [UV, $KMnO_4$]. IR [cm^{-1}]: 3138, 2930, 2251, 1677, 1532, 1409, 1338, 1252, 1182, 1092, 969, 936, 846, 790, 686. **^{1}H NMR** (300 MHz, $CDCl_3$): δ 7.65 (d, *J* = 0.5 Hz, 1H), 7.53 (s, 1H), 5.95 – 5.85 (m, 1H), 3.25 (ddd, *J* = 7.3, 1.7, 0.8 Hz, 2H), 2.24 (dd, *J* = 1.9, 0.9 Hz, 3H). **^{13}C NMR** (75 MHz, $CDCl_3$): δ 139.2, 138.7, 124.8, 117.1, 112.0, 104.5, 15.8, 14.3. **HRMS** (APCI) calcd. for $[C_8H_9ClN_3]^+$ ($[M+H]^+$), m/z = 182.0480; found 182.0475.

(3-(4-Chloro-1H-pyrazol-1-yl)allyl)diphenylphosphine oxide (3aj''):

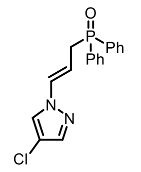

3aj''

According to general procedure C: (*E*)-(3-(4-chloro-1H-pyrazol-1-yl)prop-1-en-1-yl)diphenylphosphine oxide (**36**) (68.6 mg, 200 µmol, 1.00 equiv.), DBU (60.9 mg, 56.4 µL, 2.00 equiv.), DCE (4 mL), r.t., 5 h. After purification (PE/EtOAc, 5:1, then EtOAc), the product was isolated as a colorless solid (66.4 mg, 194 µmol, 97%, *E*/*Z* = 7:1; vinylic/allylic isomer >49:1,). Crude ^{1}H NMR analysis: yield of vinylic isomer: 94%; *E*/*Z* = 7:1; vinylic/allylic: isomer: >49:1. The data of the *E* isomer is given.

TLC: R_f = 0.31 (EtOAc) [UV, $KMnO_4$]. **Mp** 197.5 °C. IR [cm^{-1}]: 3053, 3109, 2937, 2873, 1670, 1592, 1528, 1487, 1435, 1387, 1331, 1215, 1174, 1122, 1074, 973, 850, 828, 783, 719, 693. **^{1}H NMR** (300 MHz, $CDCl_3$): δ 7.62 – 7.50 (m, 4H), 7.47 – 7.31 (m, 8H), 6.73 (ddt, *J* = 18.9, 17.0, 4.7 Hz, 1H), 6.06 (ddt, *J* = 22.0, 16.9, 1.8 Hz, 1H), 4.88 – 4.75 (m, 2H). **^{13}C NMR** (75 MHz, $CDCl_3$): δ 139.2, 132.2 (d, J_{C-P} = 2.6 Hz), 132.0 (d, J_{C-P} = 99.5 Hz), 131.0 (d, J_{C-P} = 9.2 Hz), 128.8 (d, J_{C-P} = 11.8 Hz), 125.2, 111.9, 106.6 (d, J_{C-P} = 9.6 Hz), 31.8 (d, J_{C-P} = 68.9 Hz). **^{31}P NMR** (162 MHz, $CDCl_3$) δ 30.2. **HRMS** (ESI) calcd. for $[C_{18}H_{17}ClN_2OP]^+$ ($[M+H]^+$), m/z = 343.0762; found 343.076 and 343.076.

Diethyl (*E*)-3-(4-chloro-1H-pyrazol-1-yl)hex-2-enedioate (C_{12}) and diethyl (*E*)-3-(4-chloro-1H-pyrazol-1-yl)hex-3-enedioate (C_{23}) (3c''):

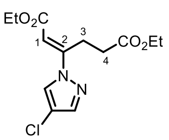

3c'' (C_{12})

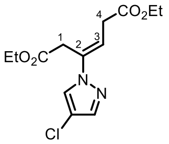

3c'' (C_{23})

According to general procedure C: Diethyl (E)-4-(4-chloro-1H-pyrazol-1-yl)hex-2-enedioate (2) (60.2 mg, 200 µmol, 1.00 equiv.), DBU (60.9 mg, 56.4 µL, 2.00 equiv.), DCE (4 mL), 60 °C, 18 h. After purification (DCM), two isomers were isolated separately as colorless oils (C_{12} isomer: 30.2 mg, 100 µmol, 50%, *E*; C_{23} isomer: 16.5 mg, 54.9 µmol, 27%, *E*). Crude ^{1}H NMR analysis: yield of vinylic isomer: 52% of C_{12} isomer and 38% of C_{23} isomer (E/Z = 4:1); vinylic/allylic isomer: 12:1.

C_{12} isomer: **TLC:** R_f = 0.48 (PE/EtOAc, 5:1) [UV, $KMnO_4$]. IR [cm^{-1}]: 3138, 2982, 1707, 1640, 1539, 1439, 1372, 1346, 1260, 1156, 1040, 969, 917, 857, 798, 731. **^{1}H NMR** (400 MHz, $CDCl_3$): δ 7.83 (s, 1H), 7.60 (s, 1H), 6.23 (s, 1H), 4.21 (q, *J* = 7.2 Hz, 2H), 4.12 (q, *J* = 7.2 Hz, 2H), 3.59 – 3.48 (m, 2H), 2.64 – 2.56 (m, 2H), 1.30 (t, *J* = 7.2 Hz, 3H), 1.24 (t, *J* = 7.2 Hz, 3H). **^{13}C NMR** (101 MHz, $CDCl_3$): δ 172.0, 165.8, 152.3, 140.8, 125.3, 113.7, 106.4, 60.7, 60.5, 32.6, 23.8, 14.2. **HRMS** (APCI) calcd. for $[C_{13}H_{18}ClN_2O_4]^+$ ($[M+H]^+$), m/z = 301.0950; found 301.0953.

C_{23} isomer: **TLC:** R_f = 0.27 (PE/EtOAc, 5:1) [UV, $KMnO_4$]. IR [cm^{-1}]: 3138, 2982, 1730, 1677, 1528, 1431, 1372, 1252, 1163, 1029, 969, 846, 790, 784. **^{1}H NMR** (400 MHz, $CDCl_3$): δ 7.74 (d, *J* = 0.7 Hz, 1H), 7.49 (s, 1H), 6.05 (t, *J* = 7.4 Hz, 1H), 4.21 – 4.15 (m, 2H), 4.15 – 4.09 (m, 2H), 3.72 (s, 2H), 3.24 (d, *J* = 7.3 Hz, 2H), 1.28 (t, *J* = 7.2 Hz, 3H), 1.21 (t, *J* = 7.2 Hz, 3H). **^{13}C NMR** (75 MHz, $CDCl_3$): δ 170.3, 169.0, 138.7, 134.6, 125.2, 112.8, 111.7, 61.3, 61.2, 34.2, 33.0, 14.2, 14.1. **HRMS** (APCI) calcd. for $[C_{13}H_{18}ClN_2O_4]^+$ ($[M+H]^+$), m/z = 301.0950; found 301.0952.

One pot reaction for the regio-selective synthesis of N-vinylic product:

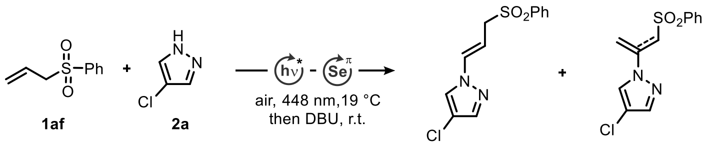

(Allylsulfonyl)benzene (**1af**) (547 mg, 3.00 mmol, 3.00 equiv.), 4-chloro-1H-pyrazole (**2a**) (103 mg, 1.00 mmol, 1.00 equiv.), TAPT (24.3 mg, 50.0 µmol, 5 mol%), $(PhSe)_2$ (31.2 mg, 100 µmol, 10 mol%) and DCE (5 mL) were added into a 100 mL round bottom flask. The flask was equipped with a septum including two cannulas. The reaction mixture was then vigorously stirred at 19 °C under irradiation at 448 nm for 21 h. After the reaction was finished, the solvent was removed under reduced pressure. Then, the flask was evacuated and then flushed with N_2 for three times. The residue was dissoved in DCE (20 mL). At r.t., DBU (305 mg, 28.2 µL, 2.00 equiv.) was added under N_2 atmosphere and the resulting mixture was contuned to react at r.t. for 23 h. After the reaction was finished, AcOH (0.5 mL) was added to quench the reaction. The solvent was removed under reduced pressure. The ^{1}H NMR-yield of the residue was determined with internal standard 1,4-dimethoxybenzene. Further purification via column chromatography (PE/EtOAc, 10:1 to 5:1), the product was isolated as a colorless solid (218.3 mg, 772 µmol, 77%).

(*E*)-4-Chloro-1-(3-(phenylsulfonyl)prop-1-en-1-yl)-1H-pyrazole (3af''):

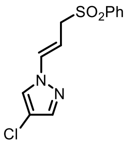

3af''

TLC: R_f = 0.31 (PE/EtOAc, 2:1) [UV, $KMnO_4$]. **Mp** 114.2 °C. IR [cm^{-1}]: 3131, 3068, 2993, 2926, 1670, 1584, 1439, 1387, 1342, 1297, 1226, 1197, 1137, 1081, 965, 895, 783, 731. **^{1}H NMR** (300 MHz, $CDCl_3$): δ 7.91 – 7.81 (m, 2H), 7.69 – 7.59 (m, 1H), 7.57 – 7.43 (m, 4H), 6.79 (d, *J* = 14.1 Hz, 1H), 5.92 (dt, *J* = 14.1, 8.1 Hz, 1H), 3.88 (dd, *J* = 8.0, 1.1 Hz, 2H). **^{13}C NMR** (75 MHz, $CDCl_3$): δ 140.1, 138.2, 134.1, 133.7, 129.4, 128.4, 125.8, 112.6, 103.1, 57.3. **HRMS** (ESI) calcd. for $[C_{12}H_{12}ClN_2O_2S]^+$ ($[M+H]^+$), m/z = 283.0303; found 283.0308.

General procedure for the cyclization of N-allylic 1,6-diester:

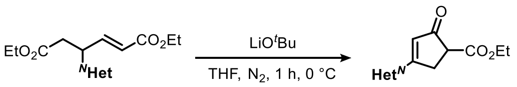

3 4

General procedure D: *N*-allylic product (1.00 equiv.) was dissoved in THF (0.05 M). At 0 °C, LiO^{t}Bu (2.2 M in THF, 5.00 equiv.) was added dropwise under N_2 atmosphere. The resulting mixture was then contuned to react at 0 °C for 1 h. After the reaction was finished, saturated NH_4Cl (aq., 1 mL) was added at 0 °C to quench the excess LiO^{t}Bu, followed by the addition of H_2O (20 mL) and DCM (20 mL). The two phases were separated, and the queous phase was extracted with DCM (2 x 20 mL). The organic phases were combined, and the solvent was removed under reduced pressure. The ^{1}H NMR-yield of the residue was determined with internal standard 1,4-dimethoxybenzene. Further purification via column chromatography provided the target compound.

Ethyl 4-(4-chloro-1H-pyrazol-1-yl)-2-oxocyclopent-3-ene-1-carboxylate (4a):

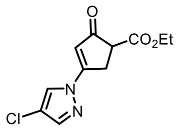

4a

According to general procedure D: Diethyl (*E*)-4-(4-chloro-1H-pyrazol-1-yl)hex-2-enedioate (**3c**) (60.2 mg, 200 μmol, 1.00 equiv.), LiO^{t}Bu (2.2 M in THF, 455 μL, 5.00 equiv.), THF (4 mL), 0 °C, 1 h. After purification (DCM), the product was isolated as a colorless solid (36.5 mg, 143 μmol, 72%; allylic/vinylic isomer >49:1). Crude ^{1}H NMR yield: 74%.

TLC: R_f = 0.17 (PE/EtOAc, 5:1) [UV, $KMnO_4$]. **Mp** 90.4 °C. IR [cm^{-1}]: 3097, 2986, 2945, 1692, 1625, 1472, 1416, 1361, 1219, 1159, 1081, 995, 962, 887, 854, 690. **^{1}H NMR** (400 MHz, $CDCl_3$): δ 7.86 (s, 1H), 7.73 (s, 1H), 6.15 (t, *J* = 1.6 Hz, 1H), 4.25 (q, *J* = 7.2 Hz, 2H), 3.65 (dd, *J* = 7.4, 2.8 Hz, 1H), 3.56 (ddd, *J* = 18.2, 2.8, 1.7 Hz, 1H), 3.33 (ddd, *J* = 18.2, 7.5, 1.6 Hz, 1H), 1.31 (t, *J* = 7.2 Hz, 3H). **^{13}C NMR** (101 MHz, $CDCl_3$): δ 197.8, 168.3, 166.9, 143.1, 126.4, 115.9, 112.3, 62.1, 51.6, 30.4, 14.2. **HRMS** (APCI) calcd. for $[C_{11}H_{12}ClN_2O_3]^+$ ($[M+H]^+$), m/z = 255.0531; found 255.0532.

Ethyl 4-(1H-benzo[d][1,2,3]triazol-1-yl)-2-oxocyclopent-3-ene-1-carboxylate (4b):

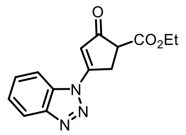

4b

According to general procedure D: Diethyl (*E*)-4-(1H-benzo[d][1,2,3]triazol-1-yl)hex-2-enedioate (**3bh**) (63.5 mg, 200 μmol, 1.00 equiv.), and LiO^{t}Bu (2.2 M in THF, 455 μL, 5.00 equiv.), THF (4 mL), 0 °C, 1 h. After purification (DCM), the product was isolated as a white solid (30.4 mg, 112 μmol, 56%). Crude ^{1}H NMR yield: 55%.

TLC: R_f = 0.08 (PE/EtOAc, 5:1) [UV, $KMnO_4$]. **Mp** 168.1 °C. IR [cm^{-1}]: 3116, 2989, 2926, 2117, 1730, 1692, 1614, 1484, 1454, 1428, 1372, 1323, 1215, 1170, 1036, 1010, 924, 846, 753. **^{1}H NMR** (400 MHz, $CDCl_3$): δ 8.20 (d, *J* = 8.4 Hz, 1H), 7.77 (d, *J* = 8.4 Hz, 1H), 7.73 – 7.66 (m, 1H), 7.62 – 7.48 (m, 1H), 6.49 (t, *J* = 1.6 Hz, 1H), 4.28 (q, *J* = 7.2 Hz, 2H), 4.01 (ddd, *J* = 18.0, 2.7, 1.6 Hz, 1H), 3.82 (ddd, *J* = 18.2, 7.4, 1.6 Hz, 1H), 3.74 (dd, *J* = 7.3, 2.8 Hz, 1H), 1.33 (t, *J* = 7.1 Hz, 3H). **^{13}C NMR** (101 MHz, $CDCl_3$): δ 198.3, 168.2, 164.7, 147.3, 131.5, 130.3, 126.1, 121.4, 112.5, 111.7, 62.1, 50.9, 32.2, 14.2. **HRMS** (ESI) calcd. for $[C_{14}H_{14}N_3O_3]^+$ ($[M+H]^+$), m/z = 272.1030; found 272.1032.

General procedure E for chiral reaction:

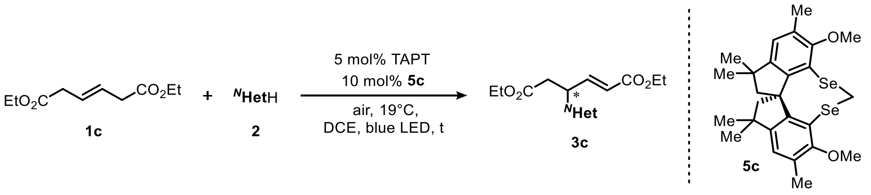

Substrates (alkene and amine), TAPT (5 mol%), chiral selenium catalyst **5c** (10 mol%) and DCE were added into a 40 mL vial or a 100 mL round bottom flask. The vial/flask was equipped with a septum including two cannulas. The reaction mixture was then vigorously stirred at 19 °C under irradiation at 448 nm for the given time. After the reaction was finished, the solvent was removed under reduced pressure. The ^{1}H NMR-yield of the residue was determined with internal standard 1,4-dimethoxybenzene. Further purification via column chromatography provided the target compound.

Diethyl (*E*)-4-(4-chloro-1H-pyrazol-1-yl)hex-2-enedioate (3c):

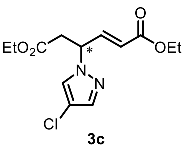

According to general procedure E: 40 mL vial, diethyl (*E*)-hex-3-enedioate (**1a**) (182 mg, 909 µmol, 3.03 equiv.), 4-chloro-1H-pyrazole (**2a**) (30.8 mg, 300 µmol, 1.00 equiv.), TAPT (7.36 mg, 15.1 µmol, 5 mol%), **5c** (16.1 mg, 30.1 µmol, 10 mol%) and DCE (4 mL), 8 h. After purification (PE/EtOAc, 9:1), the product (**2a**) was isolated as a colorless oil (22.0 mg, 90.7 µmol, 30%, 92:8 e.r.). Crude ^{1}H NMR analysis: allylic isomer: 32%; allylic/vinylic isomer >10:1. The NMR data are in accordance with the racemic product. **HPLC** (IC-3, hexane/^{i}PrOH 90:10, flow rate 0.5 ml/min, 25 °C) t_R = 29.805 min (minor), 31.770 min (major).

According to general procedure E: 100 mL round bottom flask, diethyl (*E*)-hex-3-enedioate (**1a**) (601 mg, 3.00 mmol, 3.00 equiv.), 4-chloro-1H-pyrazole (**2a**) (103 mg, 1.00 mmol, 1.00 equiv.), TAPT (24.3 mg, 50.0 µmol, 5 mol%), **5c** (53.5 mg, 100 µmol, 10 mol%) and DCE (5 mL), 21 h. After purification (PE/EtOAc, 9:1), the product (**2b**) was isolated as a yellowish oil (85.9 mg, 286 µmol, 29%, 91:9 e.r.; allylic/vinylic isomer 11:1). Crude ^{1}H NMR analysis: allylic isomer: 31%; allylic/vinylic isomer >10:1. The NMR data are in accordance with the racemic product. **HPLC** (IC-3, hexane/^{i}PrOH 90:10, flow rate 0.5 ml/min, 25 °C) t_R = 30.522 min (minor), 32.470 min (major). **Optical rotation** $[\alpha]_D^{25}$ = -13.6 (*c* 0.95, $CHCl_3$).

Diethyl (*E*)-4-(1H-benzo[d][1,2,3]triazol-1-yl)hex-2-enedioate (3bh):

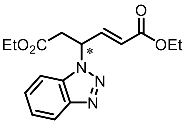

According to general procedure E: 40 mL vial, diethyl (*E*)-hex-3-enedioate (**1a**) (181 mg, 904 µmol, 3.00 equiv.), 1H-benzo[d][1,2,3]triazole (**2f**) (35.9 mg, 301 µmol, 1.00 equiv.), TAPT (7.35 mg, 15.1 µmol, 5 mol%), **5c** (16.1 mg, 30.0 µmol, 10 mol%) and DCE (4 mL), 8 h. After purification (PE/EtOAc, 9:1 to 8:2), the product (**51a**) was isolated as a yellowish oil (26.0 mg, 81.9 µmol, 27%, 90:10 e.r.; allylic/vinylic isomer >49:1). Crude ^{1}H NMR analysis: allylic isomer: 31%; allylic/vinylic isomer >10:1. The NMR data are in accordance with the racemic product. **HPLC** (IB-3, hexane/^{i}PrOH 90:10, flow rate 0.8 ml/min, 25 °C) t_R = 15.885 min (minor), 18.719 min (major).

According to general procedure E: 100 mL round bottom flask, diethyl (*E*)-hex-3-enedioate (**1a**) (601 mg, 3.00 mmol, 3.00 equiv.), 1H-benzo[d][1,2,3]triazole (**2f**) (119 mg, 1.00 mmol, 1.00 equiv.), TAPT (24.3 mg, 50.0 µmol, 5 mol%), **5c** (53.5 mg, 100 µmol, 10 mol%) and DCE (5 mL), 21 h. After purification (PE/EtOAc, 9:1 to 8:2), the product (**51b**) was isolated as a yellowish oil (75.0 mg, 236 µmol, 24%, 92:8 e.r.; allylic/vinylic isomer >49:1). Crude ^{1}H NMR analysis: allylic isomer: 29%; allylic/vinylic isomer >10:1. The NMR data are in accordance with the racemic product. **HPLC** (IB-3, hexane/^{i}PrOH 90:10, flow rate 0.8 ml/min, 25 °C) t_R = 15.817 min (minor), 18.540 min (major). **Optical rotation** $[\alpha]_D^{25}$ = 2.74 (*c* 0.95, $CHCl_3$).

References

(1) Ortgies, S.; Depken, C.; Breder, A. Oxidative Allylic Esterification of Alkenes by Cooperative Selenium-Catalysis Using Air as the Sole Oxidant. *Org. Lett.* **2016**, *18*, 2856–2859.

(2) Wilken, M.; Ortgies, S.; Breder, A.; Siewert, I. Mechanistic Studies on the Anodic Functionalization of Alkenes Catalyzed by Diselenides. *ACS Catal.* **2018**, *8*, 10901–10912.

(3) Lei, T.; Graf, S.; Schöll, C.; Krätzschmar, F.; Gregori, B.; Appleson, T.; Breder, A. Asymmetric Photoaerobic Lactonization and Aza-Wacker Cyclization of Alkenes Enabled by Ternary Selenium–Sulfur Multicatalysis. *ACS Catal.* **2023**, *13*, 16240–16248.

(4) Tao, Z.; Gilbert, B. B.; Denmark, S. E. Catalytic, Enantioselective syn-Diamination of Alkenes. *J. Am. Chem. Soc.* **2019**, *141*, 19161–19170.

(5) Kawamata, Y.; Hashimoto, T.; Maruoka, K. A Chiral Electrophilic Selenium Catalyst for Highly Enantioselective Oxidative Cyclization. *J. Am. Chem. Soc.* **2016**, *138*, 5206–5209.

(6) Van der Veen, Reinout H; Cerfontain, H. Temperature-dependent alkylation of gamma-phenyl beta, gamma-unsaturated acid and ester systems in hexamethylphosphoric triamide-tetrahydrofuran solutions using lithium diisopropylamide. *J. Org. Chem.* **1985**, *50*, 342–346.

(7) Jang, W. J.; Song, S. B.; Lee, S.; Yoo, S. Y.; Chung, Y. K.; Huh, J.; Yun, J. LiOtBu-promoted stereoselective deconjugation of α,β-unsaturated diesters probed using density functional theory. *Org. Chem. Front.* **2020**, *7*, 3427–3433.

(8) Rakete, S.; Berger, R.; Böhme, S.; Glomb, M. A. Oxidation of isohumulones induces the formation of carboxylic acids by hydrolytic cleavage. *J. Agric. Food Chem.* **2014**, *62*, 7541–7549.

Characterization Data of Substrates and Products

Ethyl (*E*)-dec-3-enoate (1b) (^{1}H NMR: 300 MHz, ^{13}C NMR: 75 MHz, CDCl$_3$):

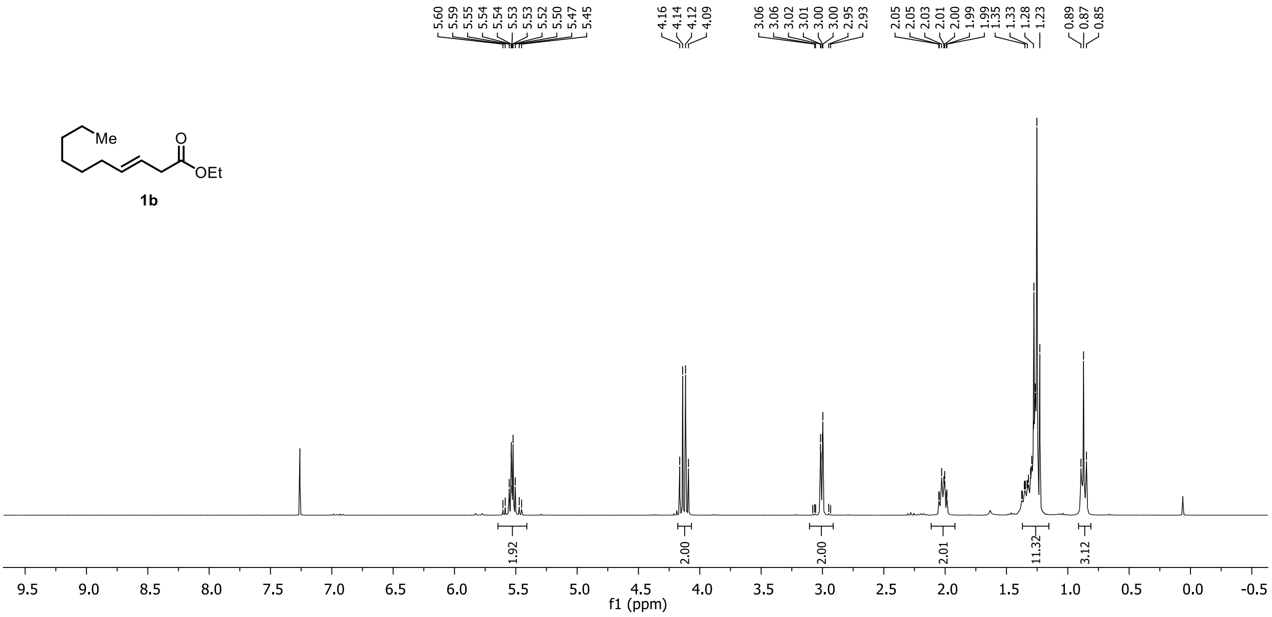

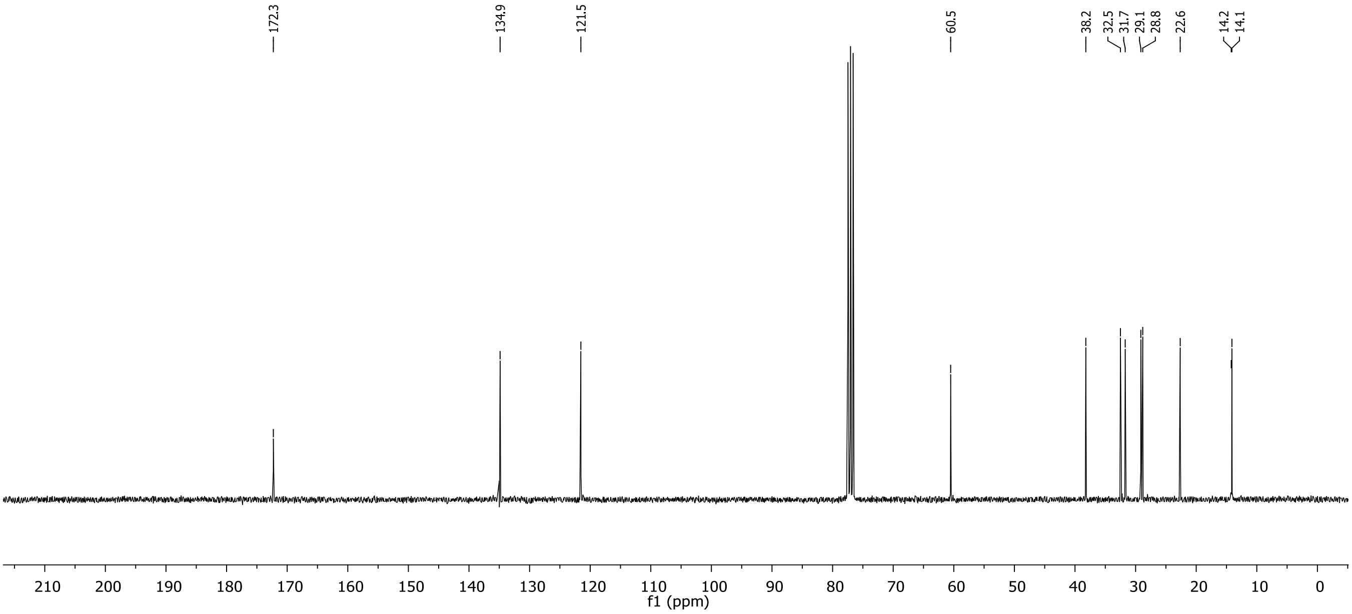

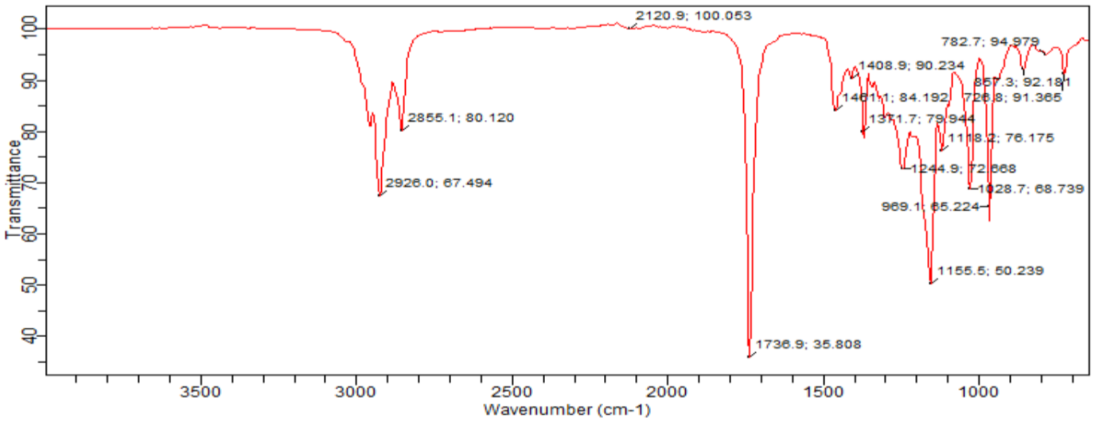

Ethyl (*E*)-8-chlorooct-3-enoate (1d) (^{1}H NMR: 300 MHz, ^{13}C NMR: 75 MHz, $CDCl_3$):

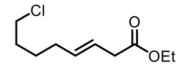

1d

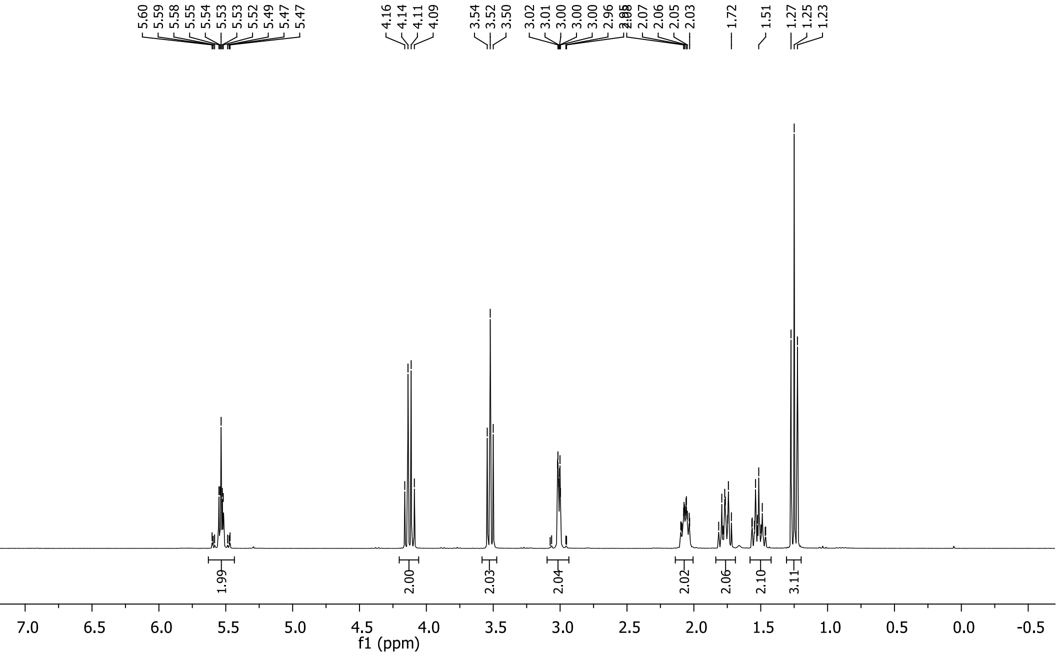

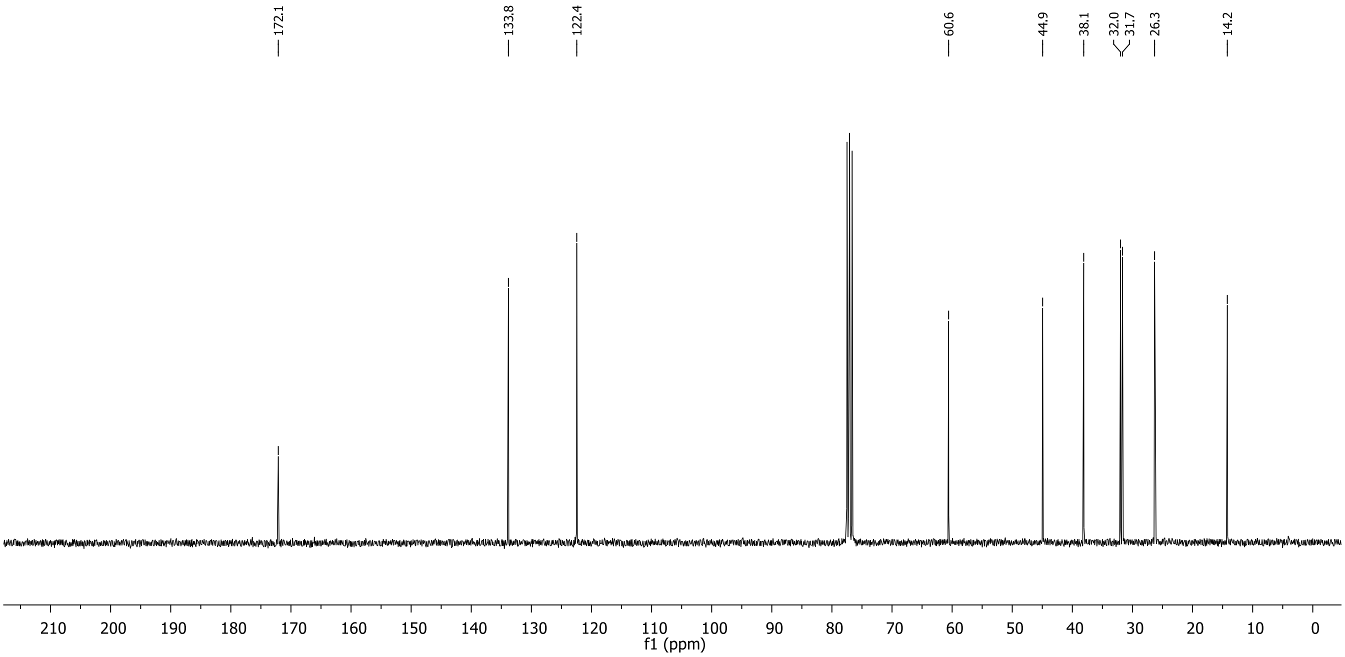

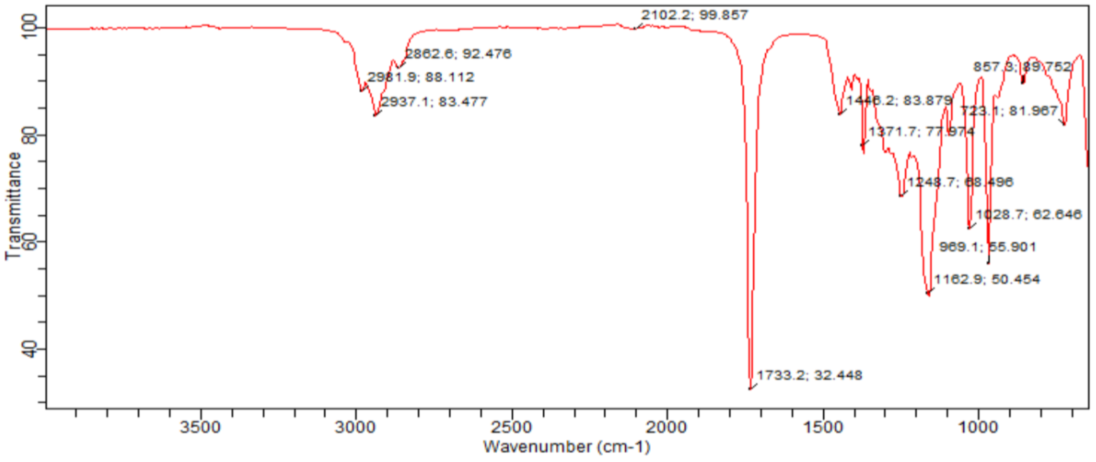

Ethyl (*E*)-8-cyanooct-3-enoate (1e) (^{1}H NMR: 300 MHz, ^{13}C NMR: 75 MHz, $CDCl_3$):

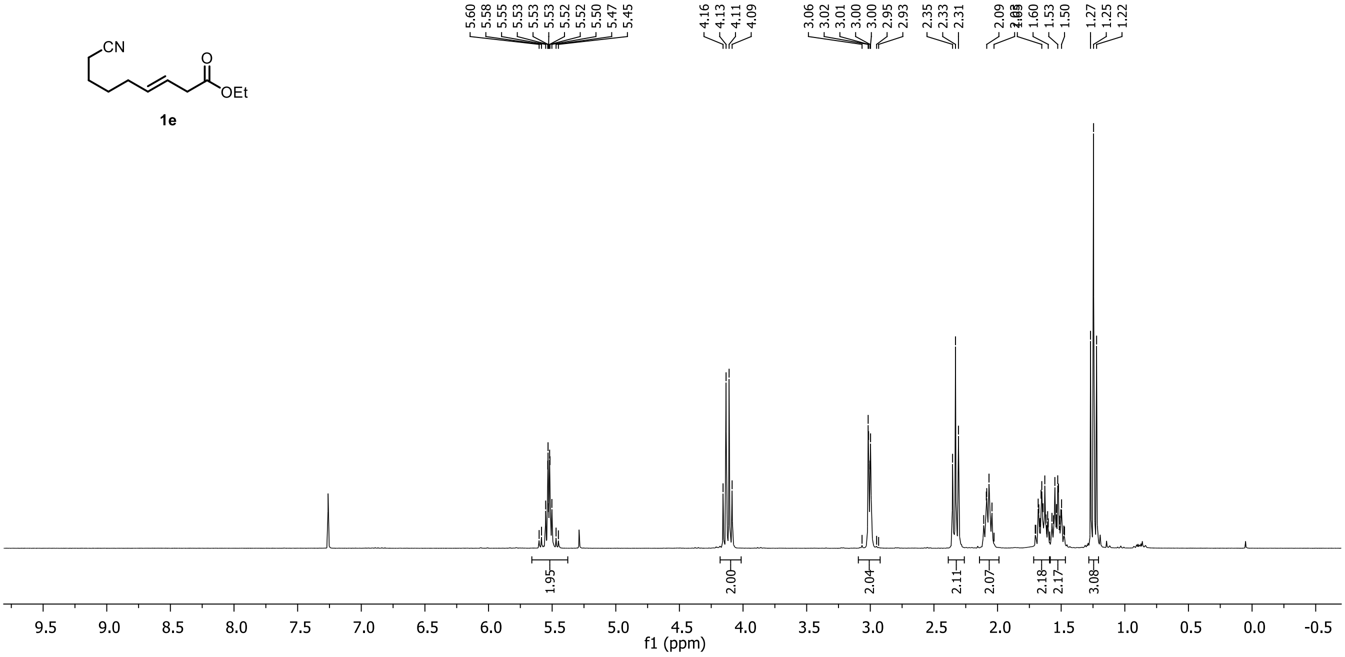

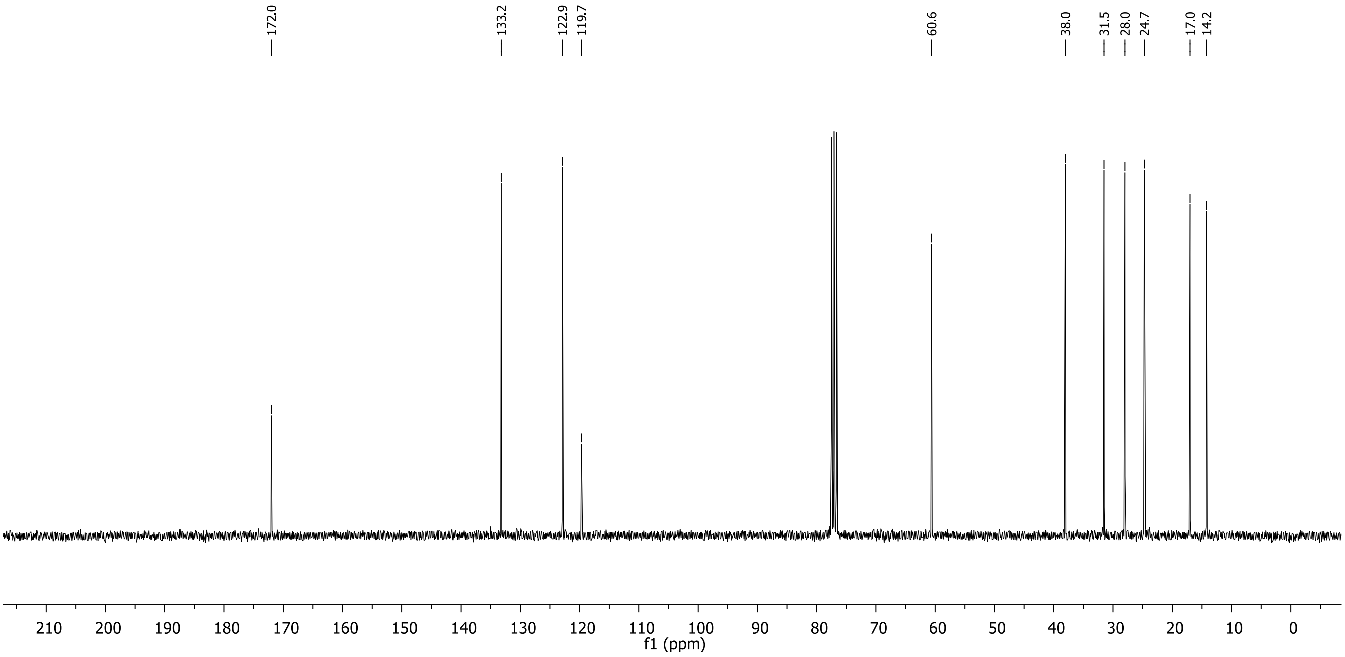

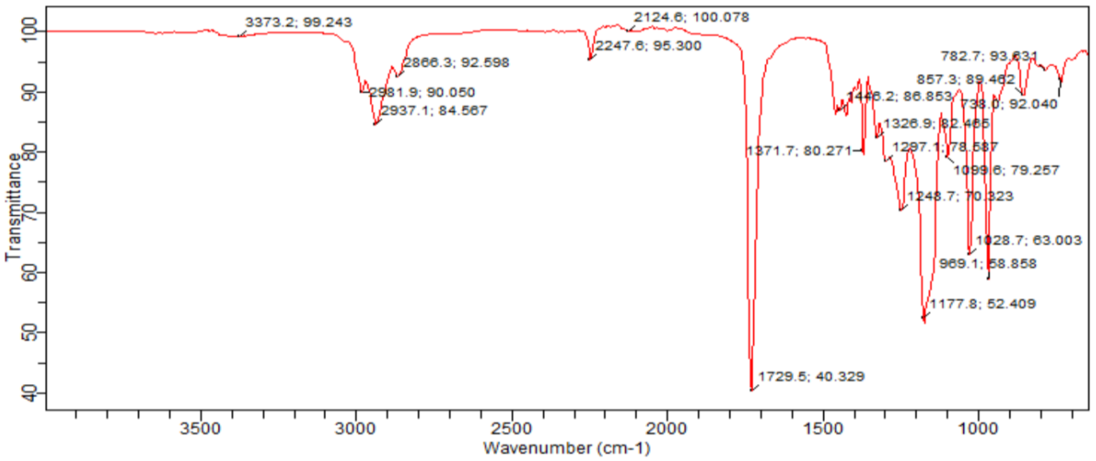

Ethyl (*E*)-7-phenylhept-3-enoate (1f) (^{1}H NMR: 300 MHz, ^{13}C NMR: 75 MHz, $CDCl_3$):

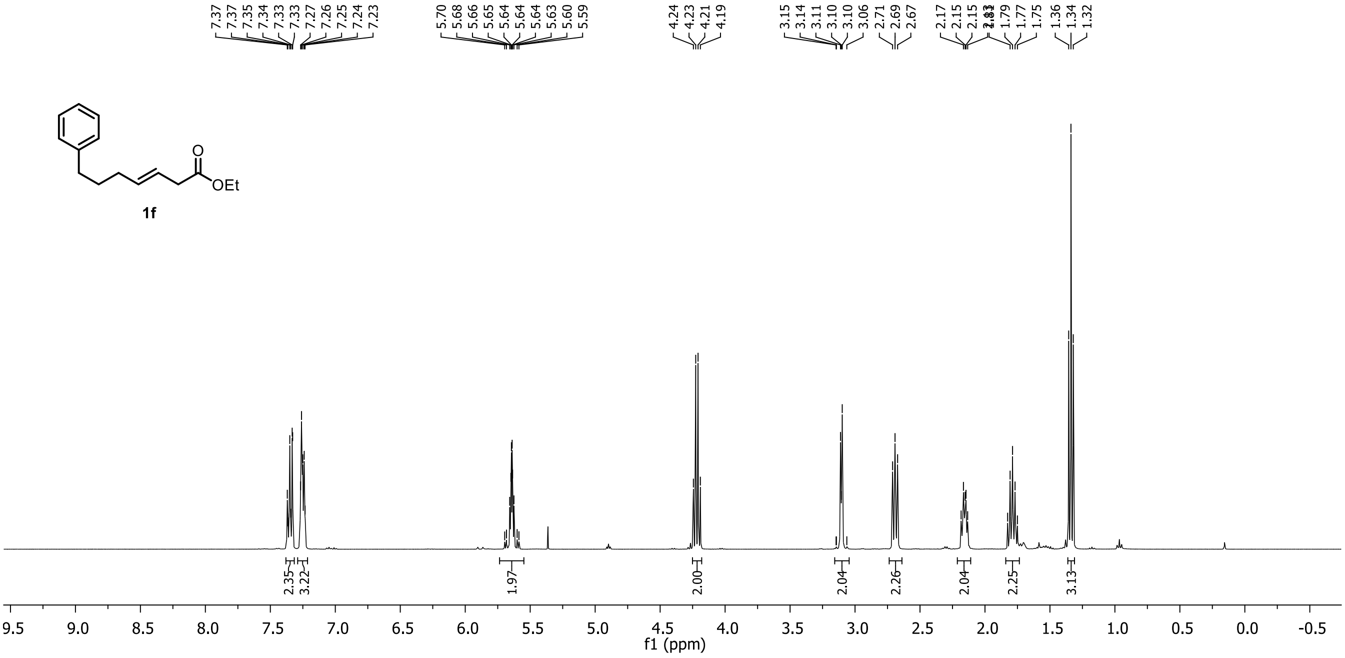

1f

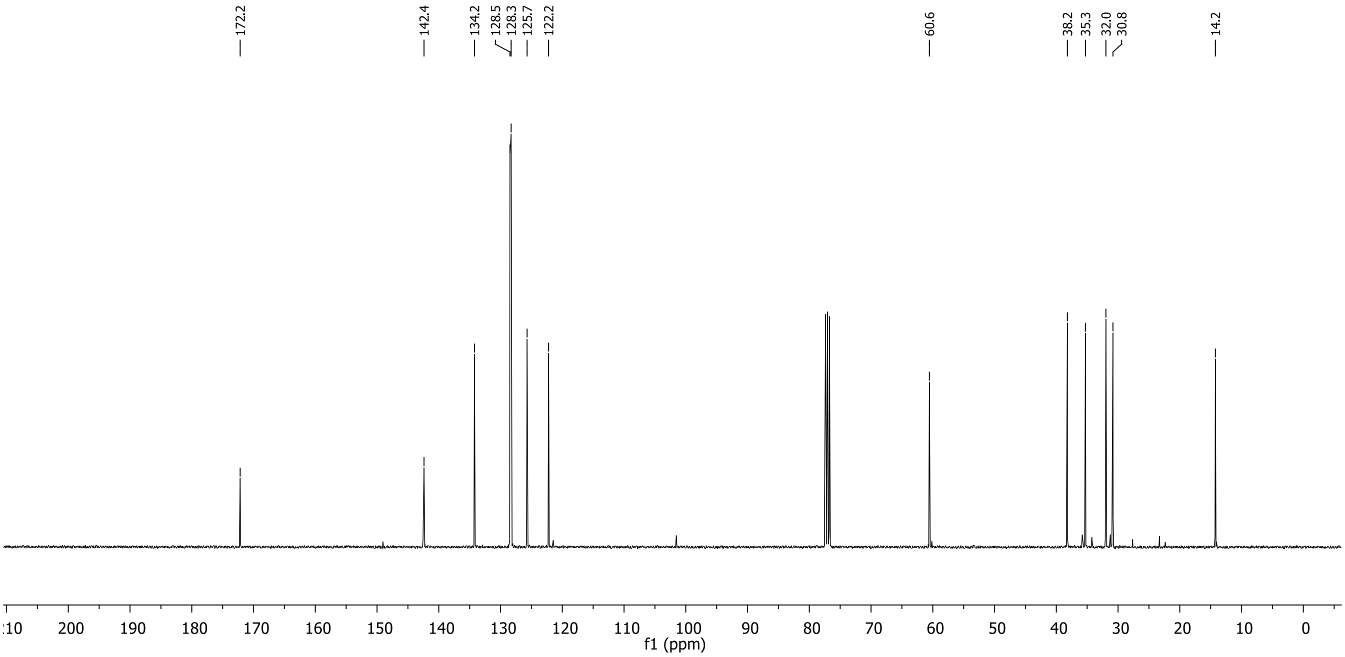

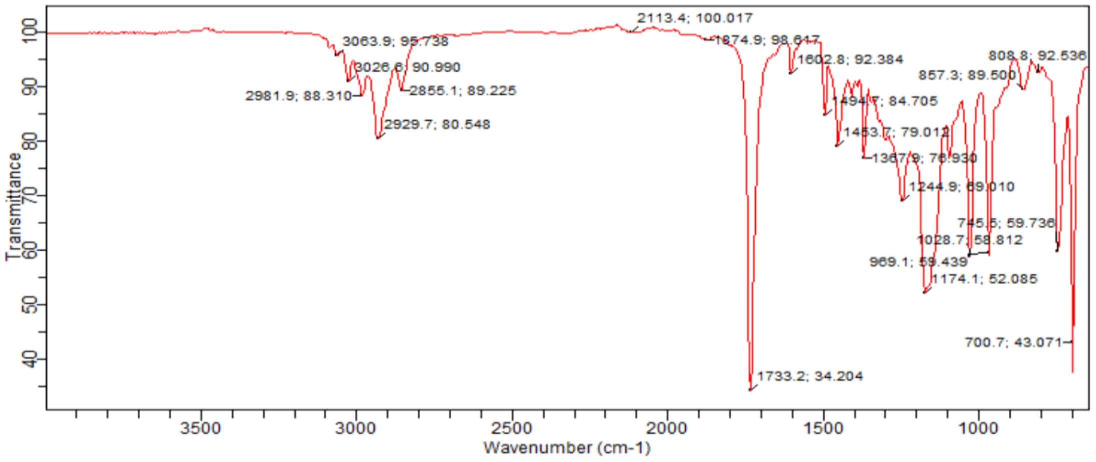

Ethyl (*E*)-7-((*tert*-butyldimethylsilyl)oxy)hept-3-enoate (1g) (^{1}H NMR: 300 MHz, ^{13}C NMR: 75 MHz, $CDCl_3$):

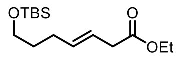

1g

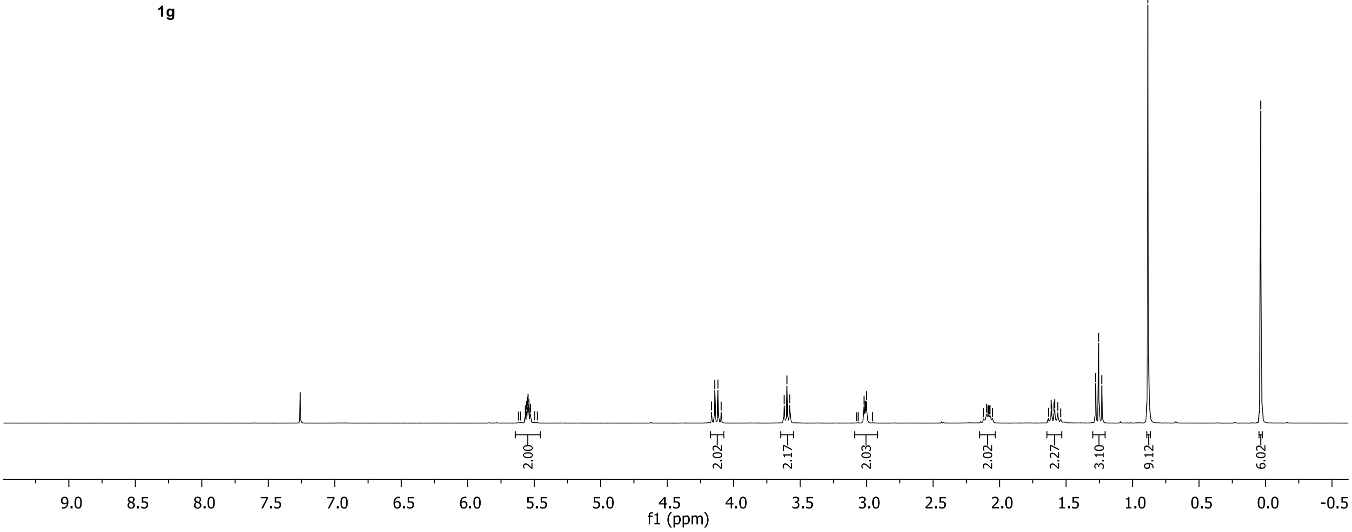

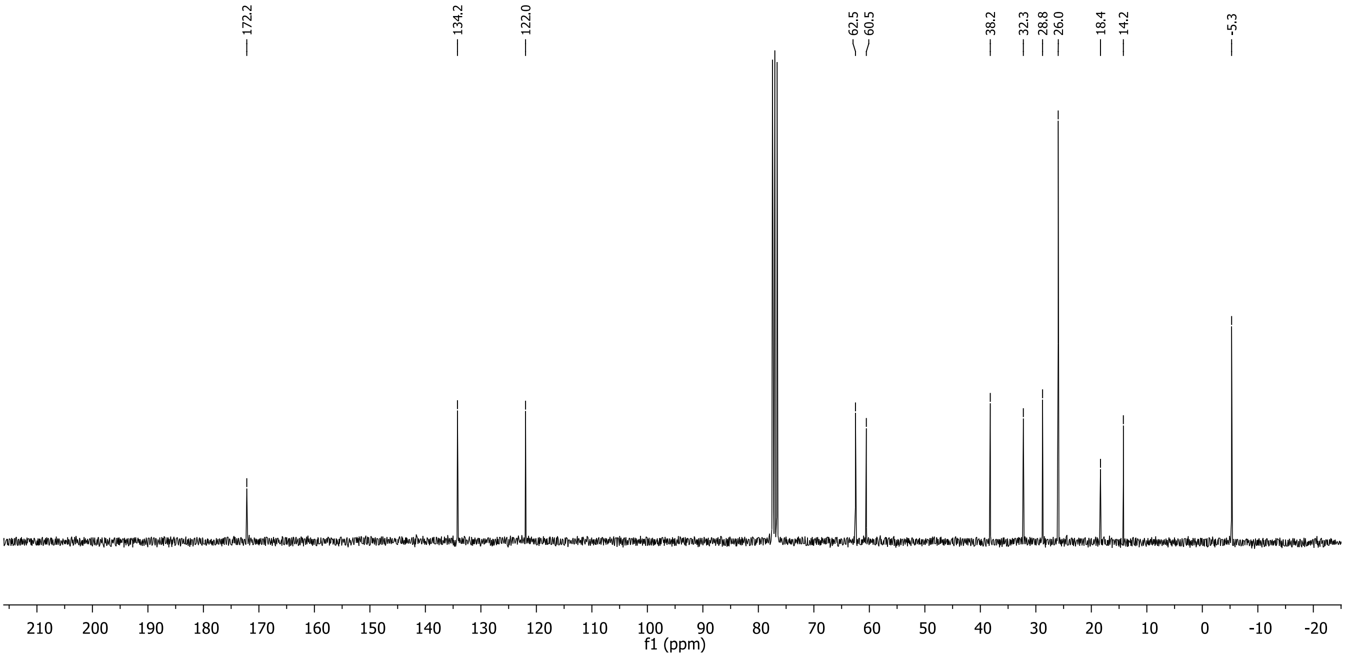

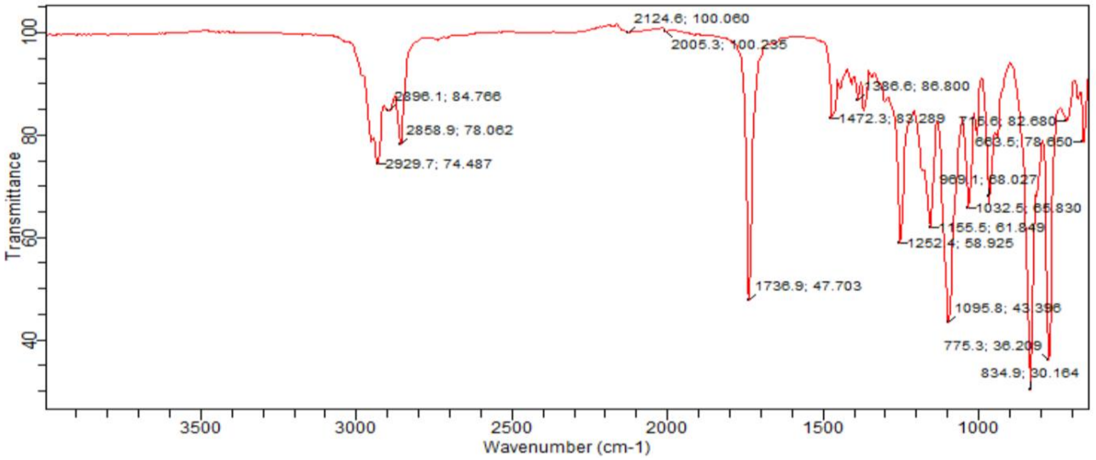

Ethyl (*E*)-7-((tetrahydro-2H-pyran-2-yl)oxy)hept-3-enoate (1h) (^{1}H NMR: 400 MHz, ^{13}C NMR: 101 MHz, CDCl$_3$):

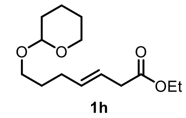

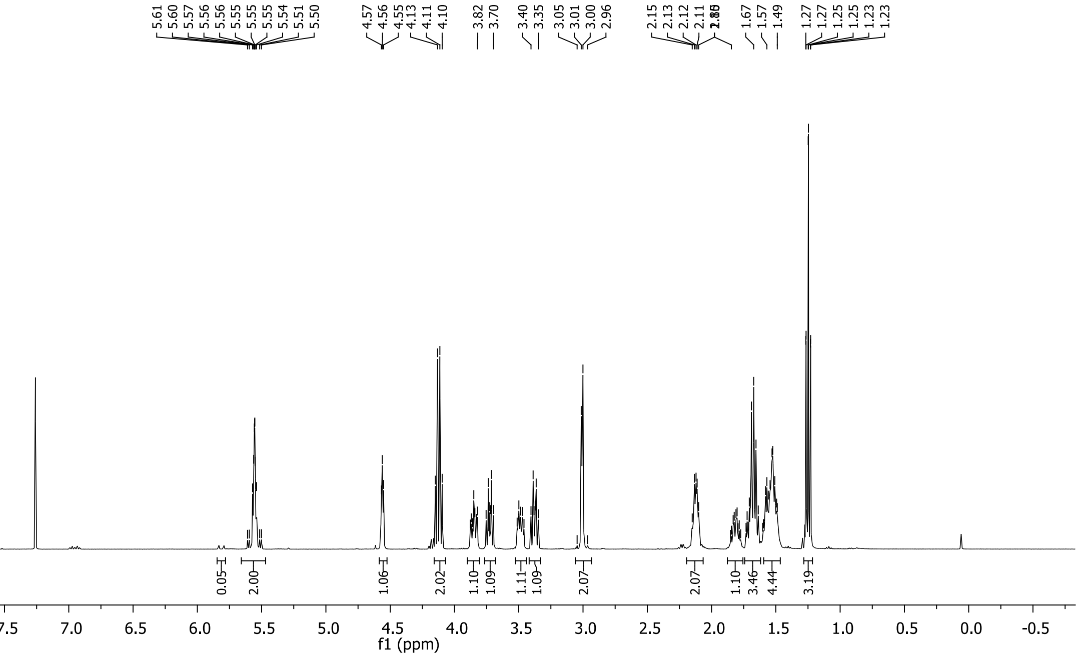

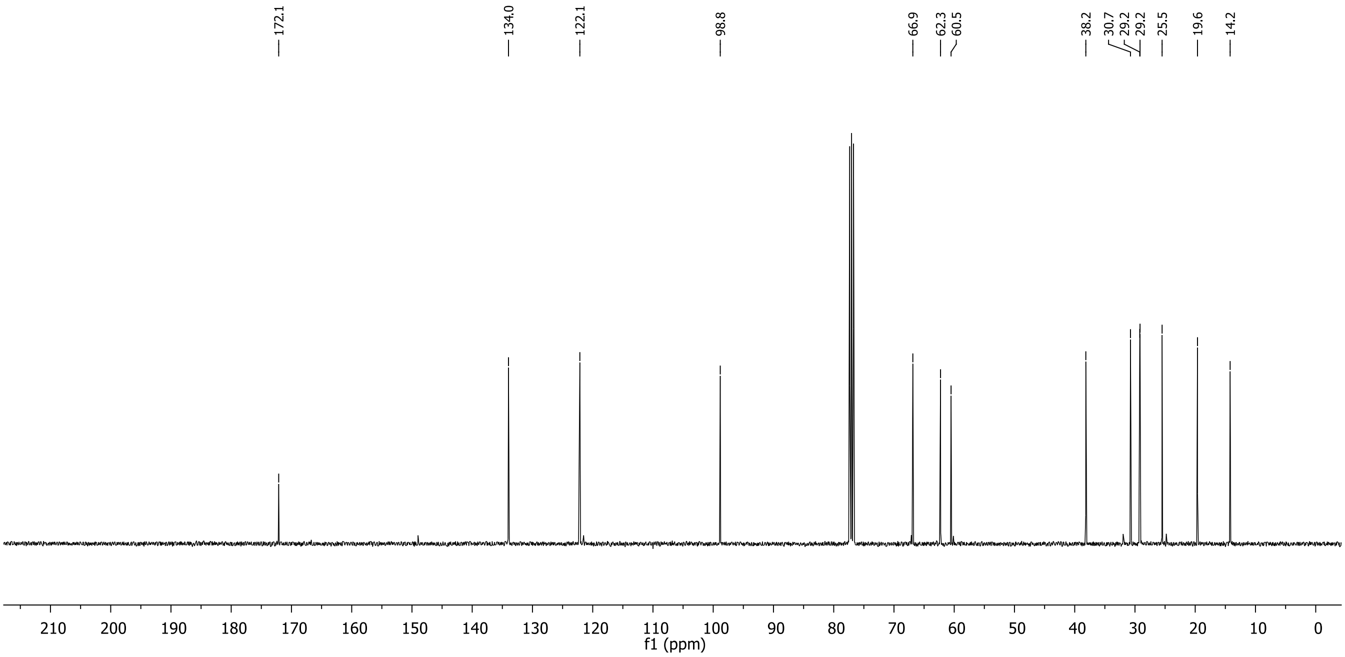

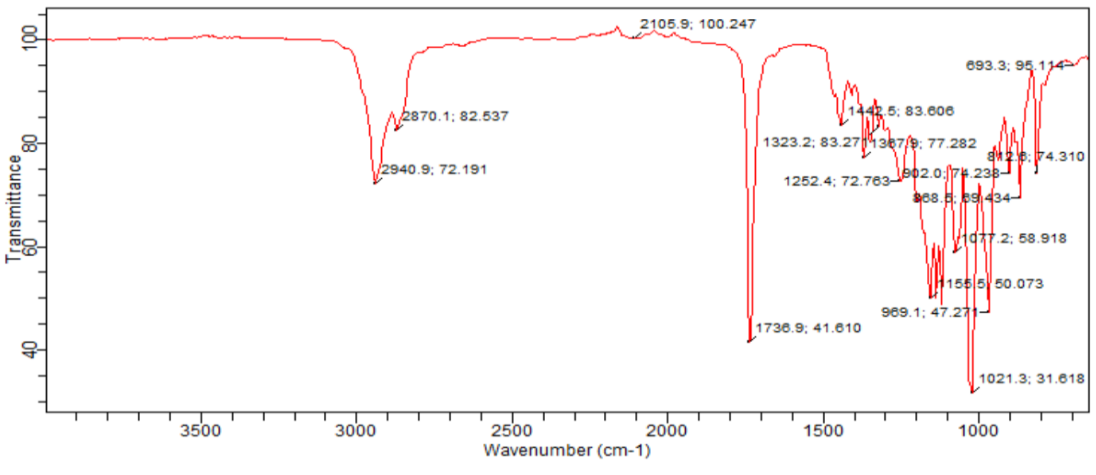

Ethyl (*E*)-6-(benzyloxy)hex-3-enoate (1i):

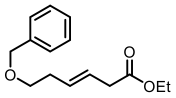

1i

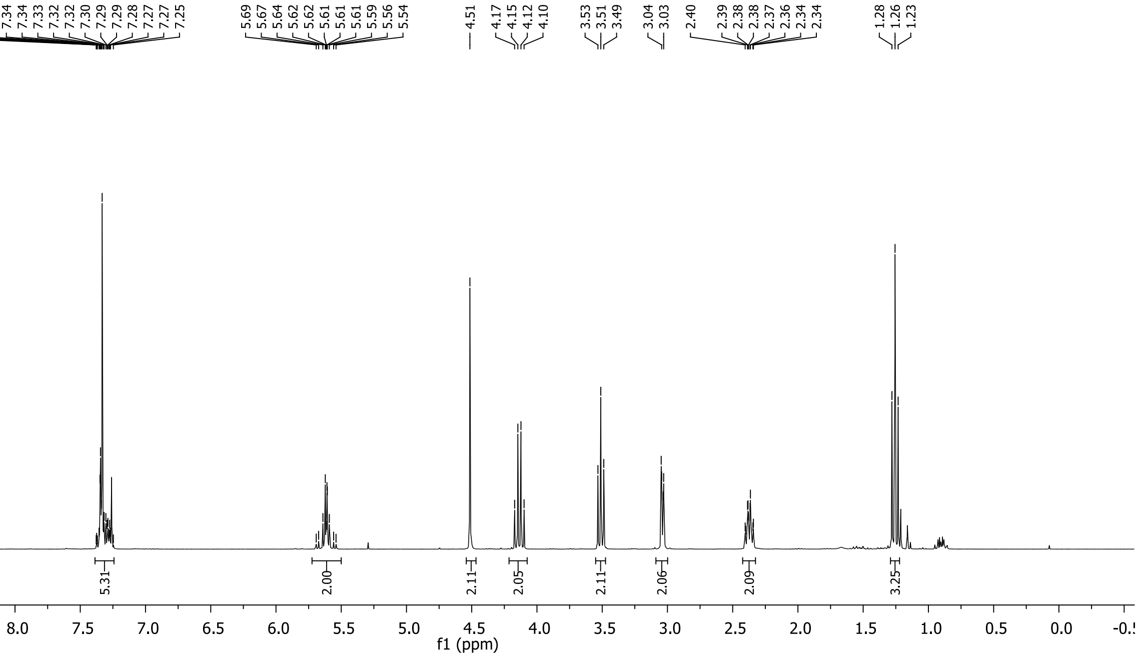

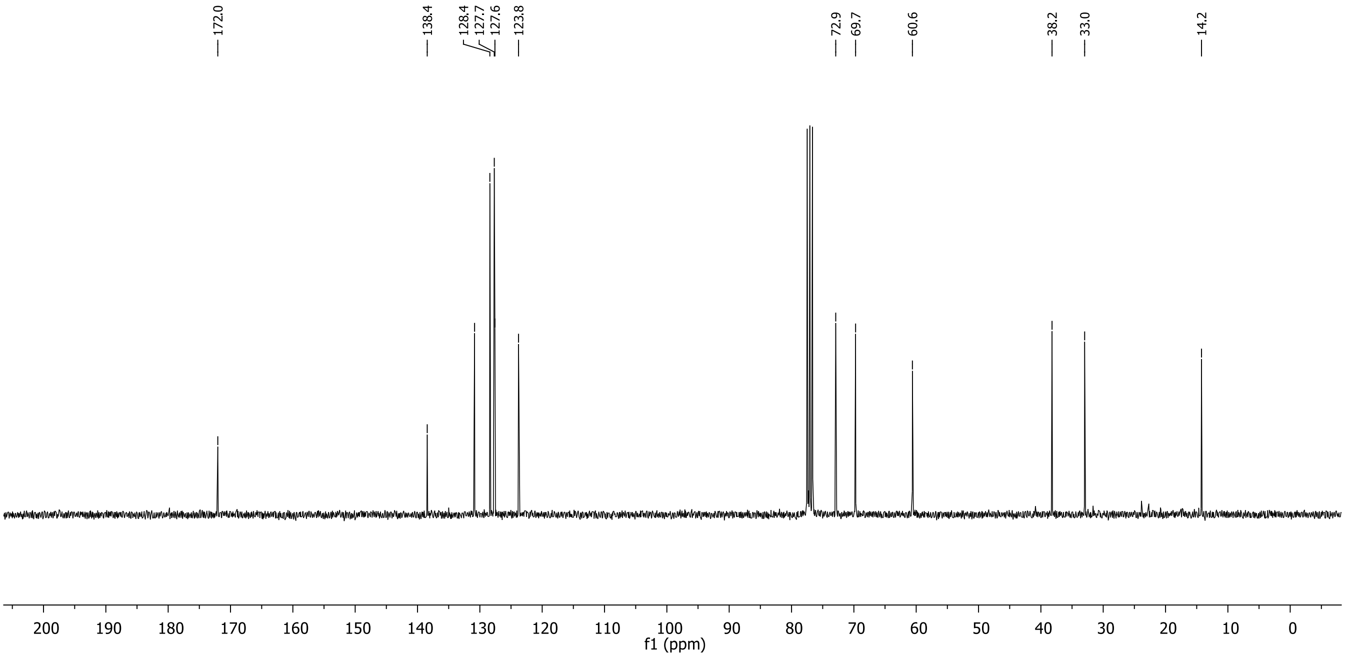

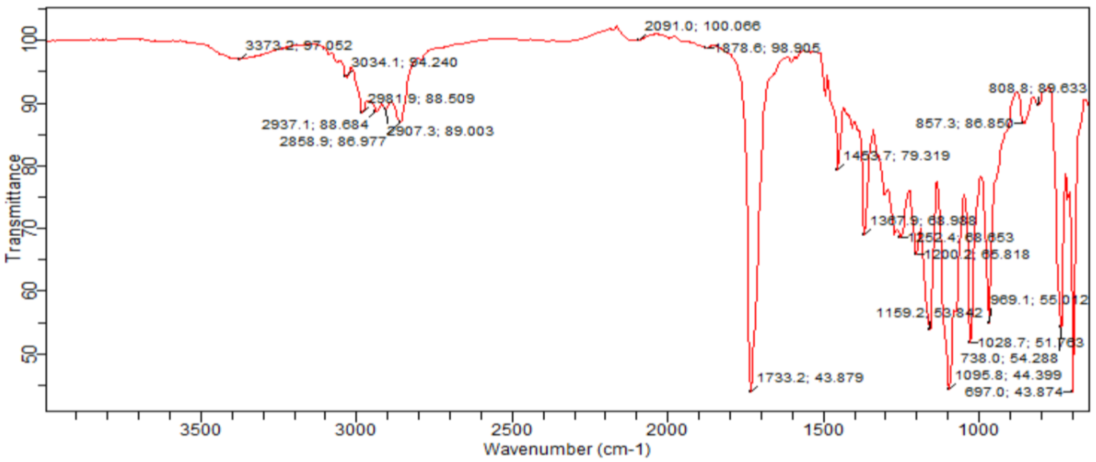

Ethyl (*E*)-5-(4-fluorophenyl)pent-3-enoate (1j) (^{1}H NMR: 300 MHz, ^{13}C NMR: 75 MHz, $CDCl_3$):

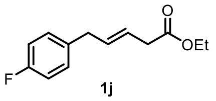

1j

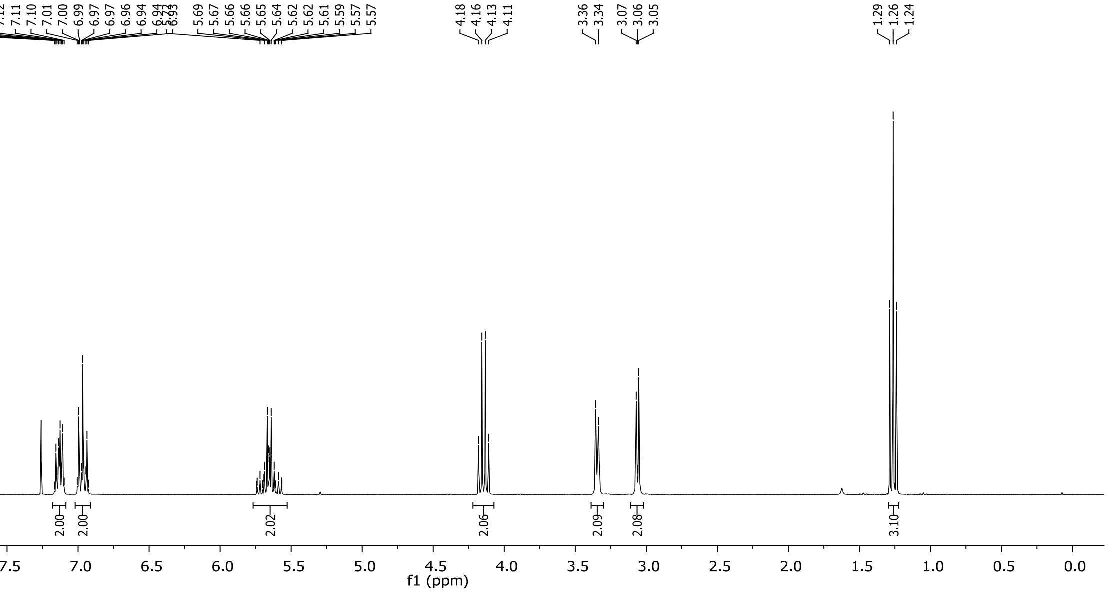

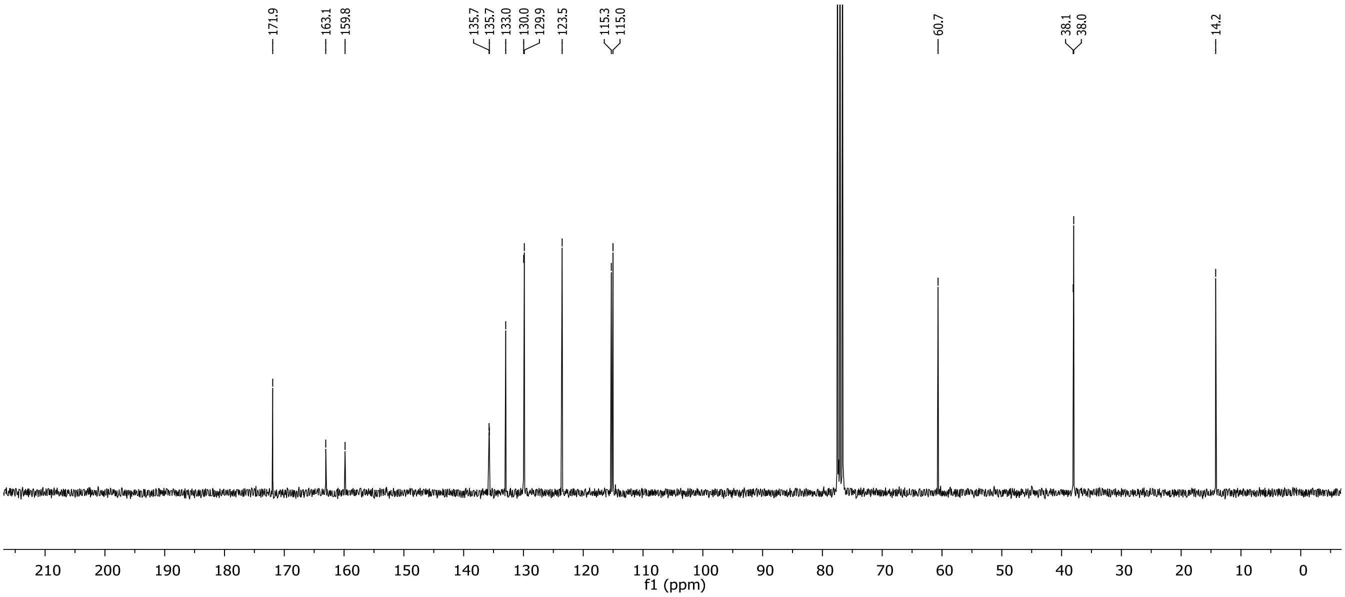

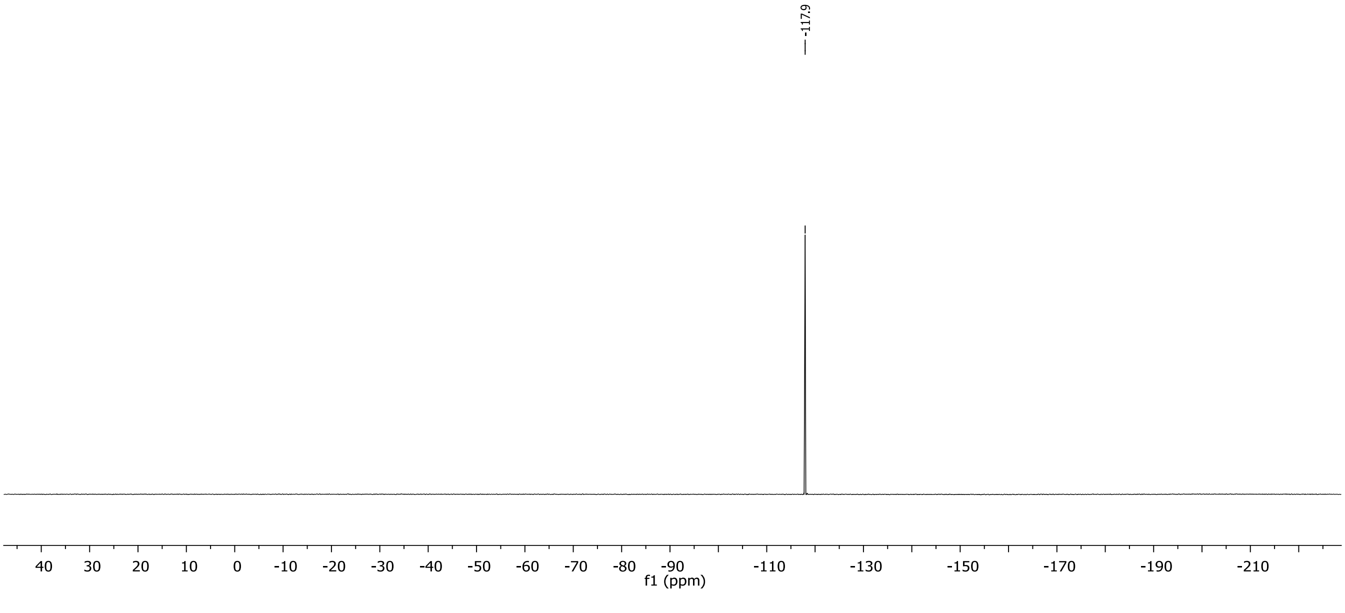

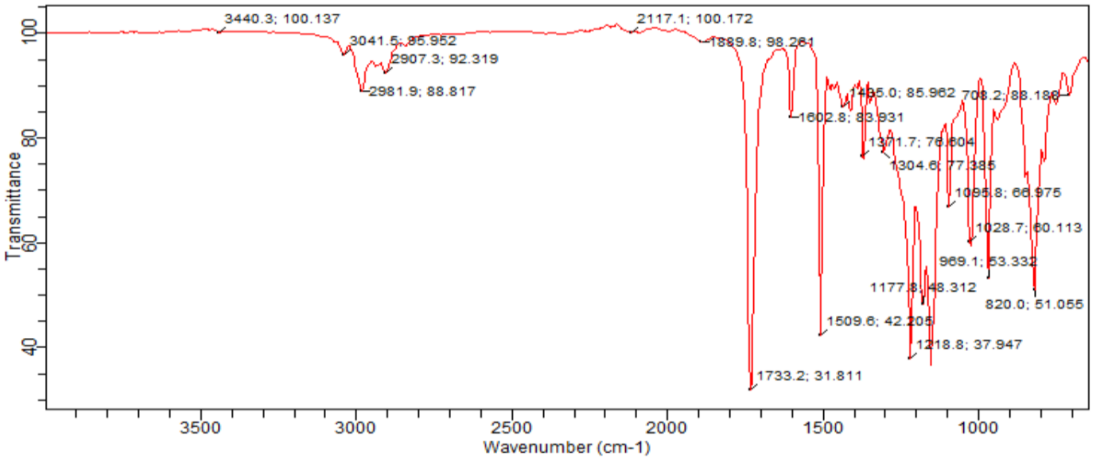

3440.3; 100.137
2117.1; 100.172
3041.5; 95.952
2907.3; 92.319
2981.9; 88.817
1889.8; 98.261
1602.8; 83.931
1495.0; 85.962
708.2; 88.180
1371.7; 76.604
1304.6; 77.385
1095.8; 66.975
1028.7; 60.113
969.1; 53.332
1177.8; 48.312
820.0; 51.055
1509.6; 42.205
1218.8; 37.947
1733.2; 31.811
Transmittance
Wavenumber (cm-1)

1-Ethyl 8-methyl (*E*)-oct-3-enedioate (1k) (^{1}H NMR: 300 MHz, ^{13}C NMR: 75 MHz, $CDCl_3$):

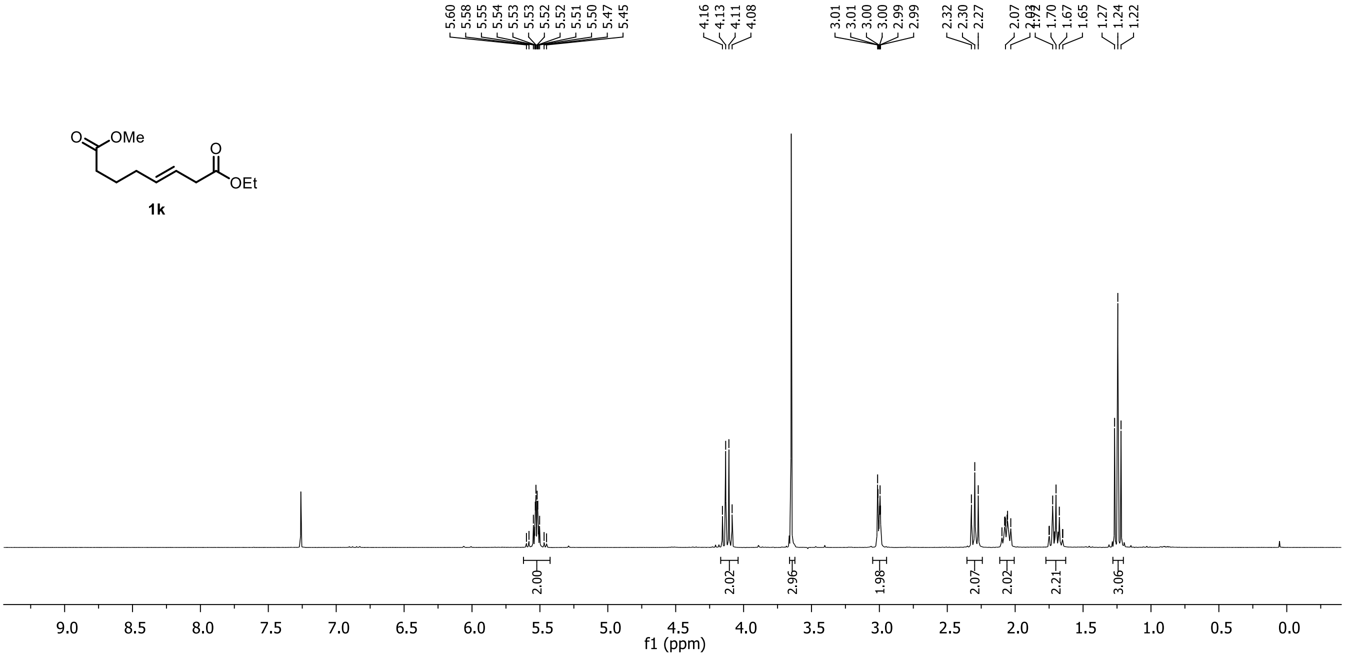

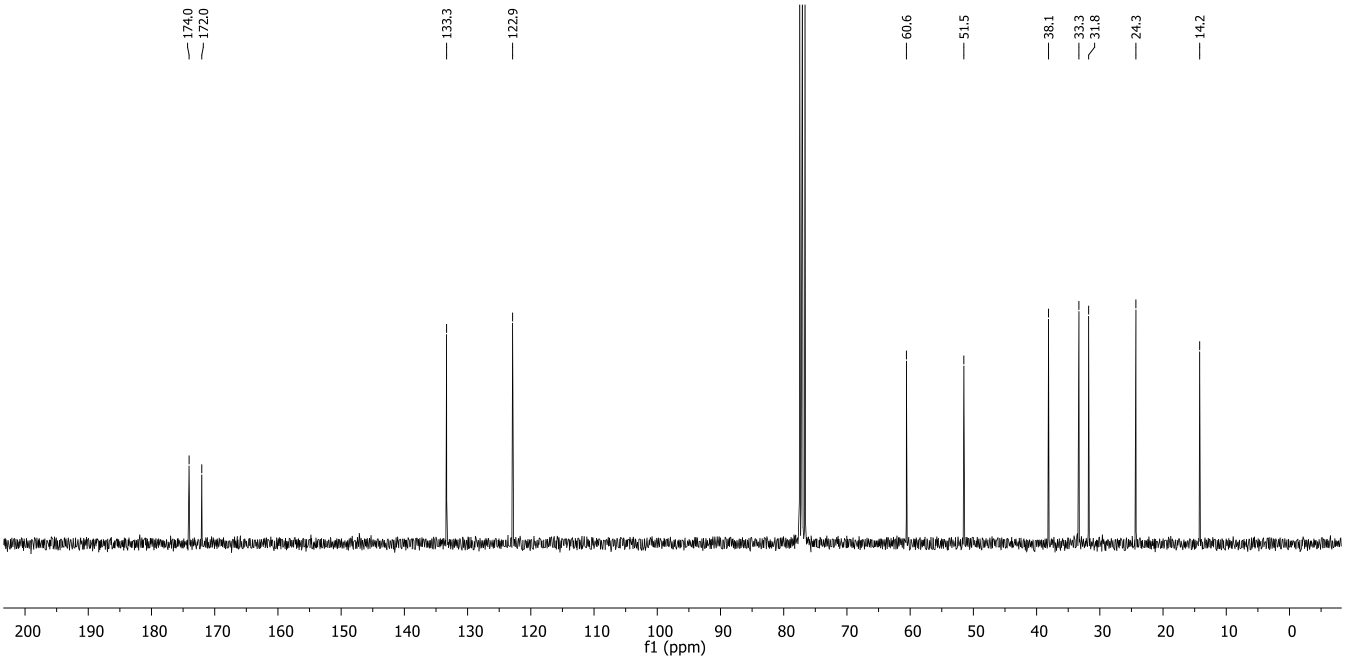

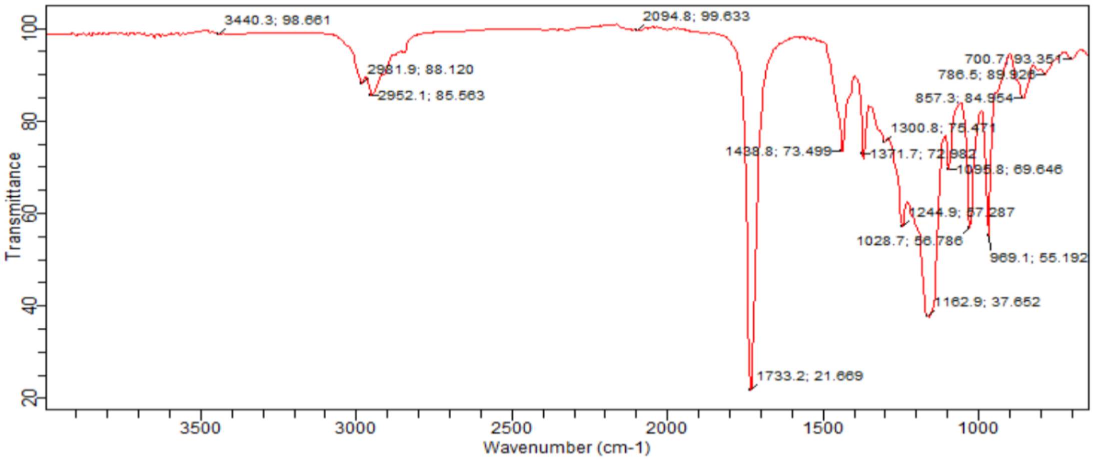

Dimethyl (*E*)-hex-3-enedioate (1l) (^{1}H NMR: 300 MHz, ^{13}C NMR: 75 MHz, CDCl$_3$):

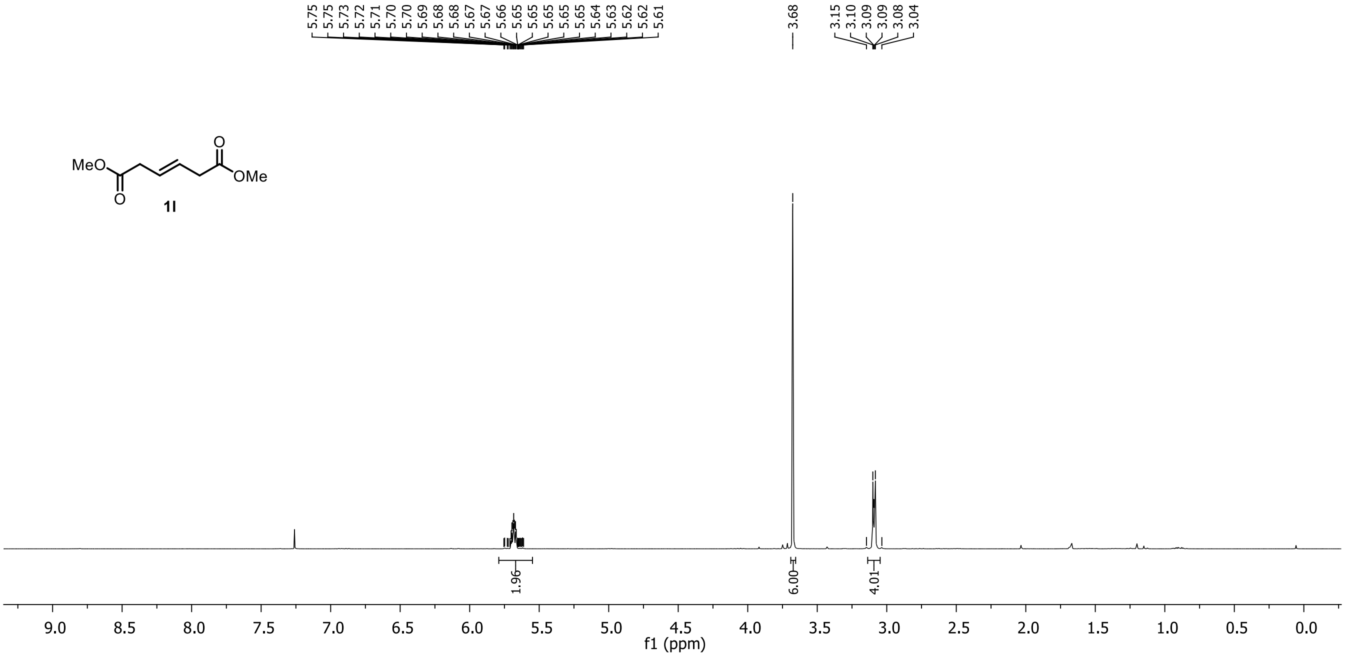

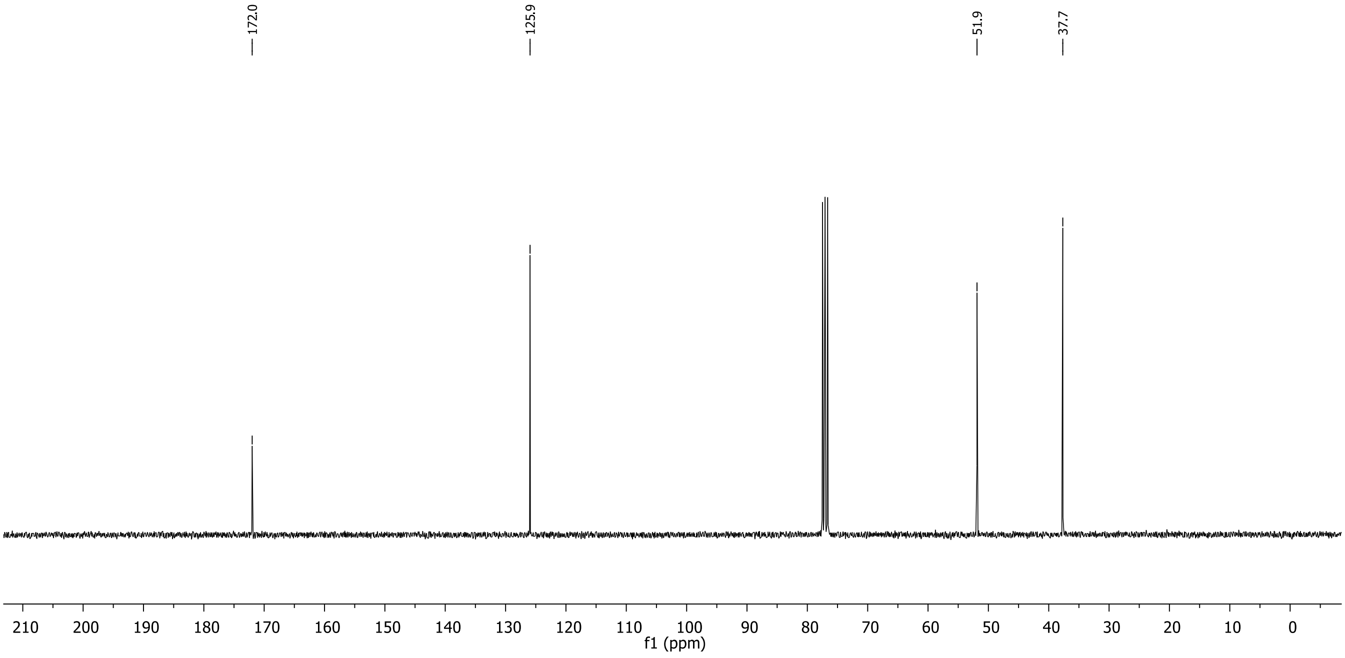

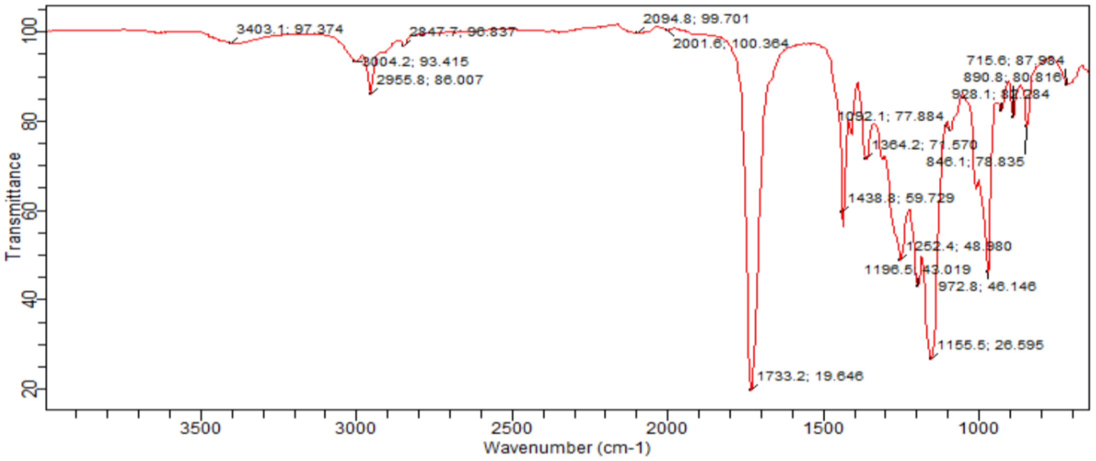

Diisopropyl (E)-hex-3-enedioate (1m) (^{1}H NMR: 300 MHz, ^{13}C NMR: 75 MHz, CDCl$_3$):

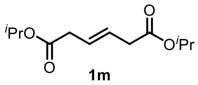

1m

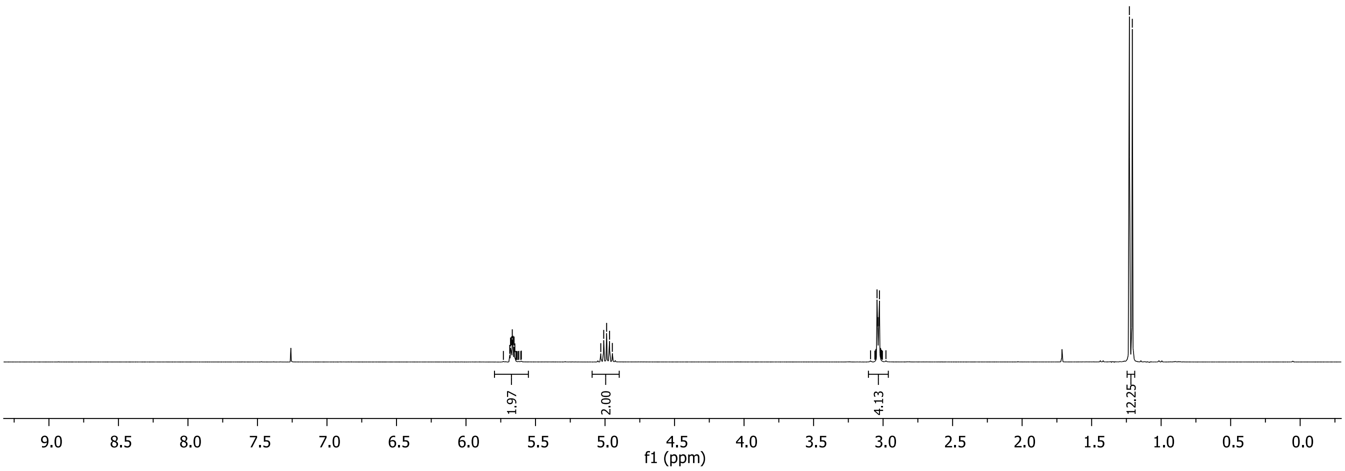

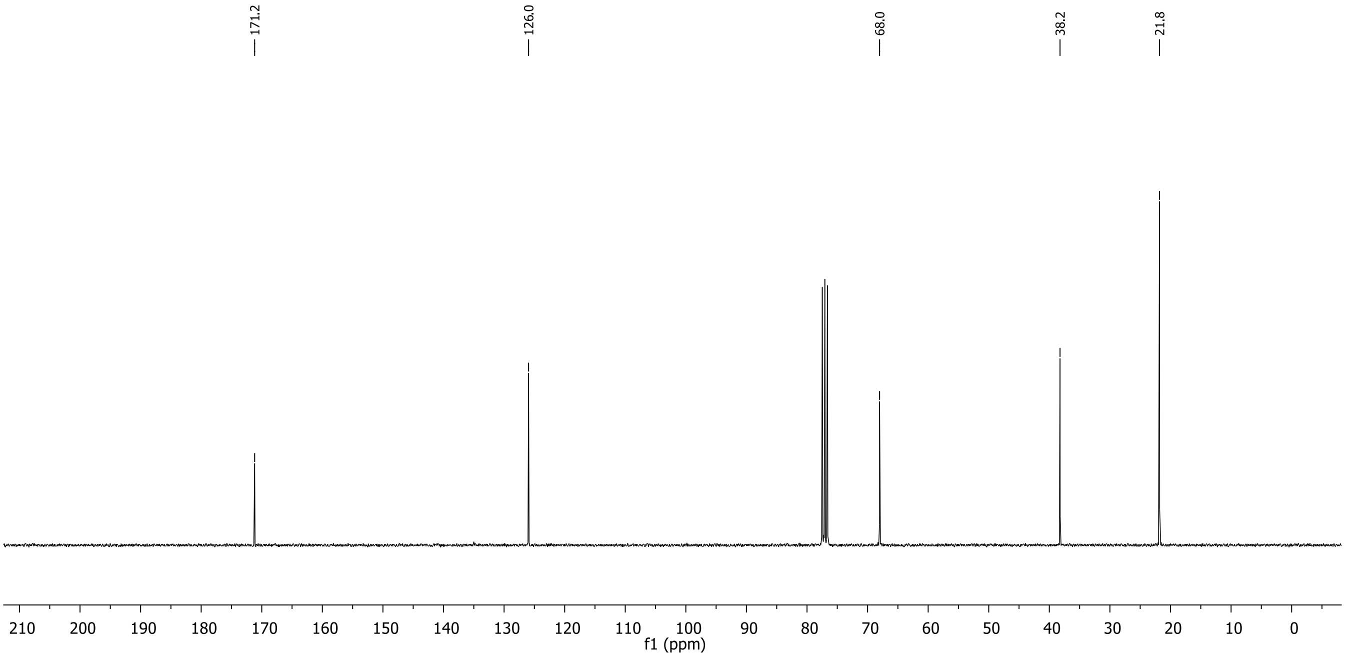

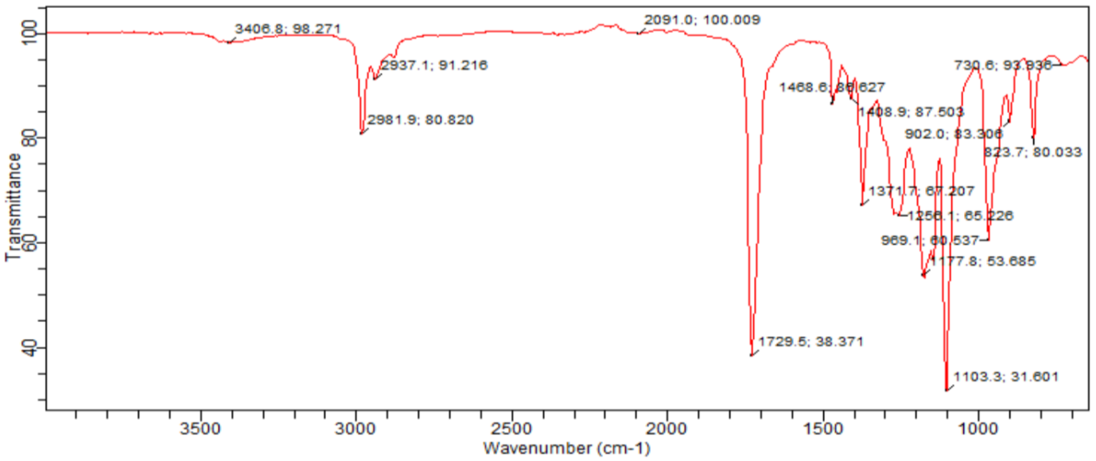

Dibenzyl (*E*)-hex-3-enedioate (1n) (^{1}H NMR: 300 MHz, ^{13}C NMR: 75 MHz, $CDCl_3$):

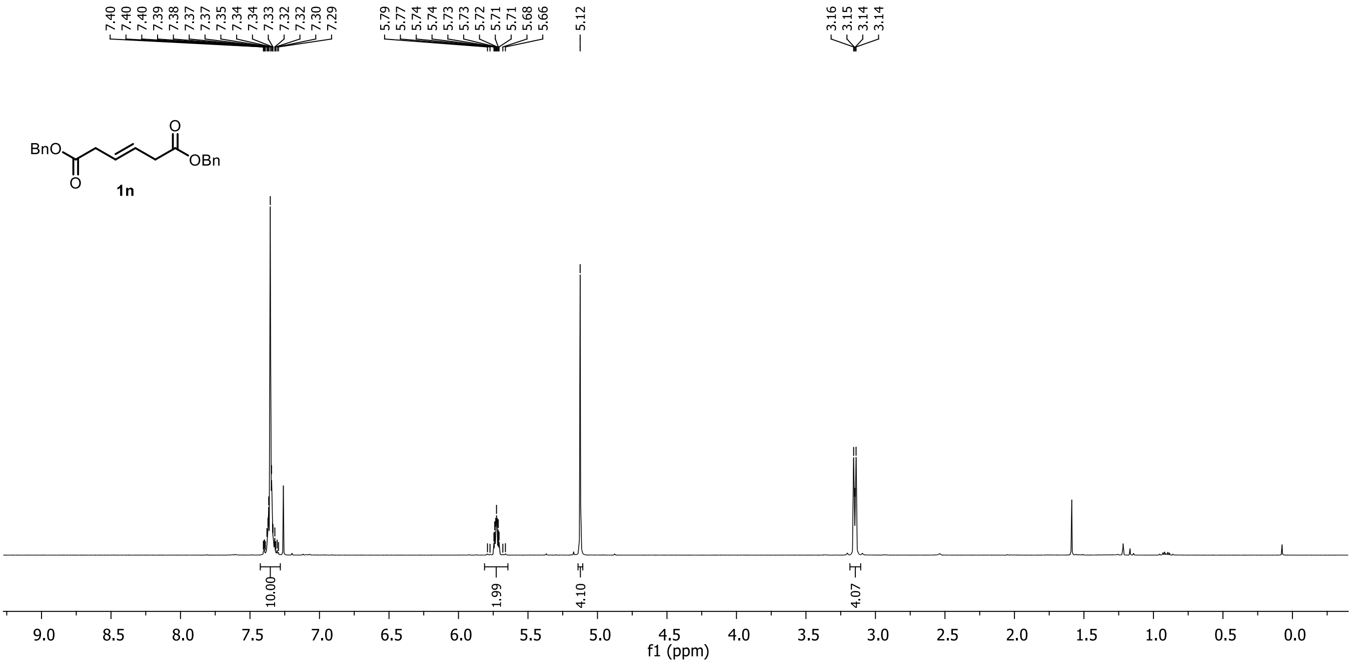

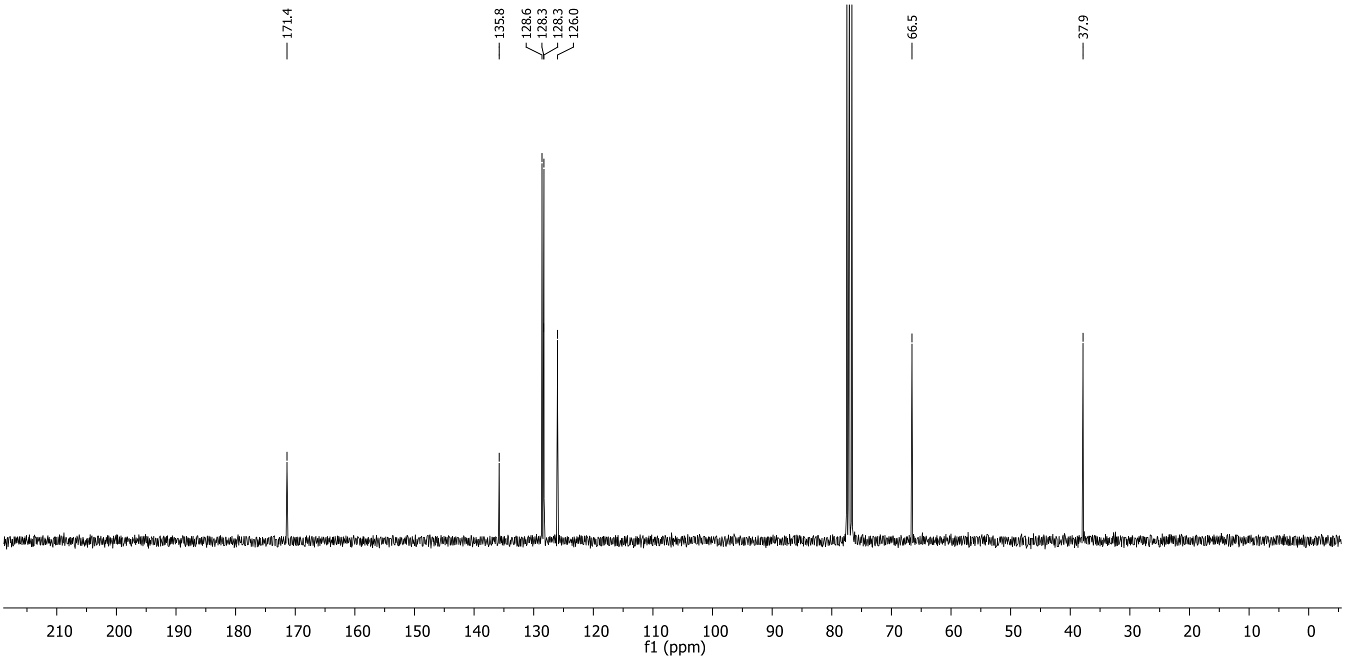

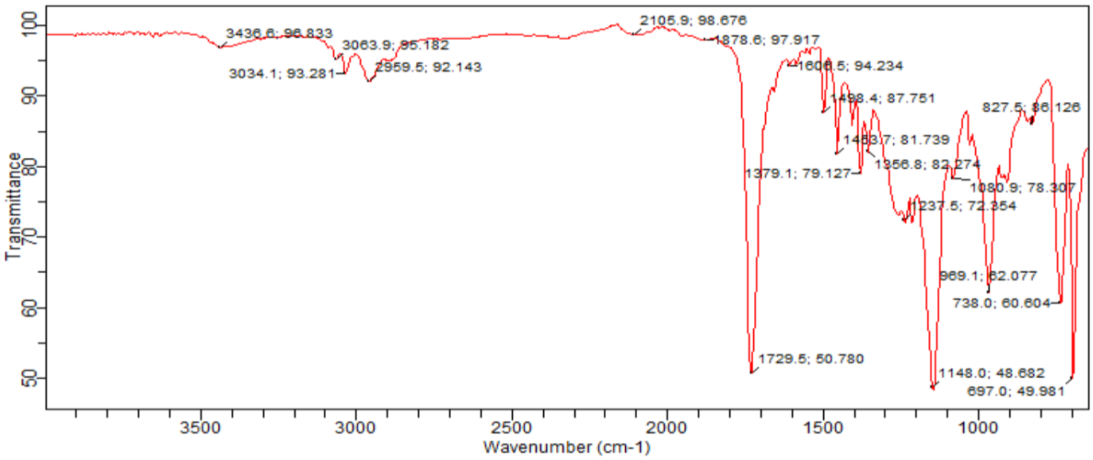

Dicyclohexyl (*E*)-hex-3-enedioate (1o) (^{1}H NMR: 300 MHz, ^{13}C NMR: 75 MHz, $CDCl_3$):

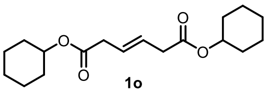

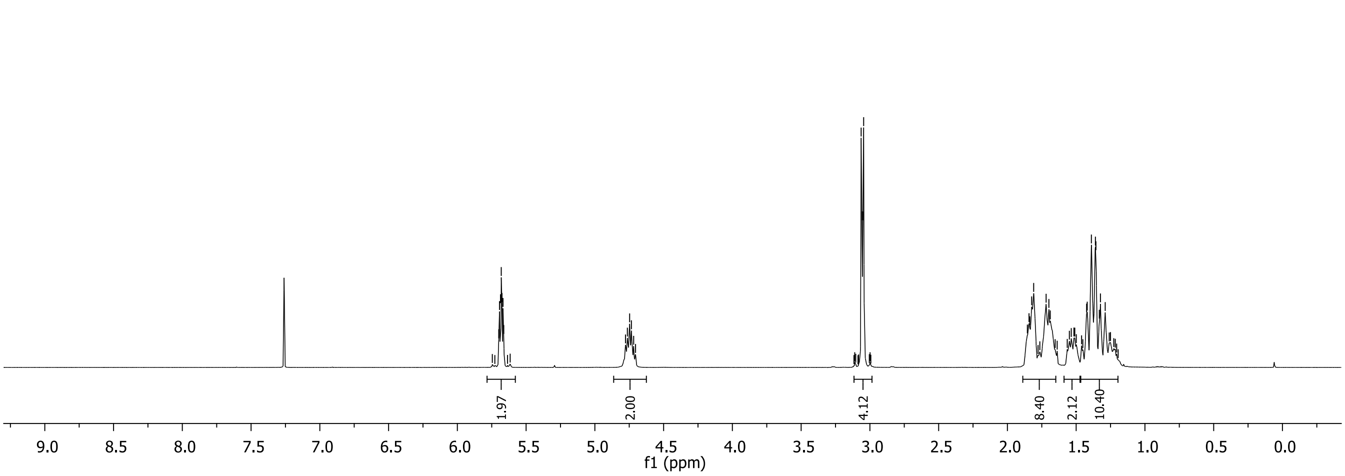

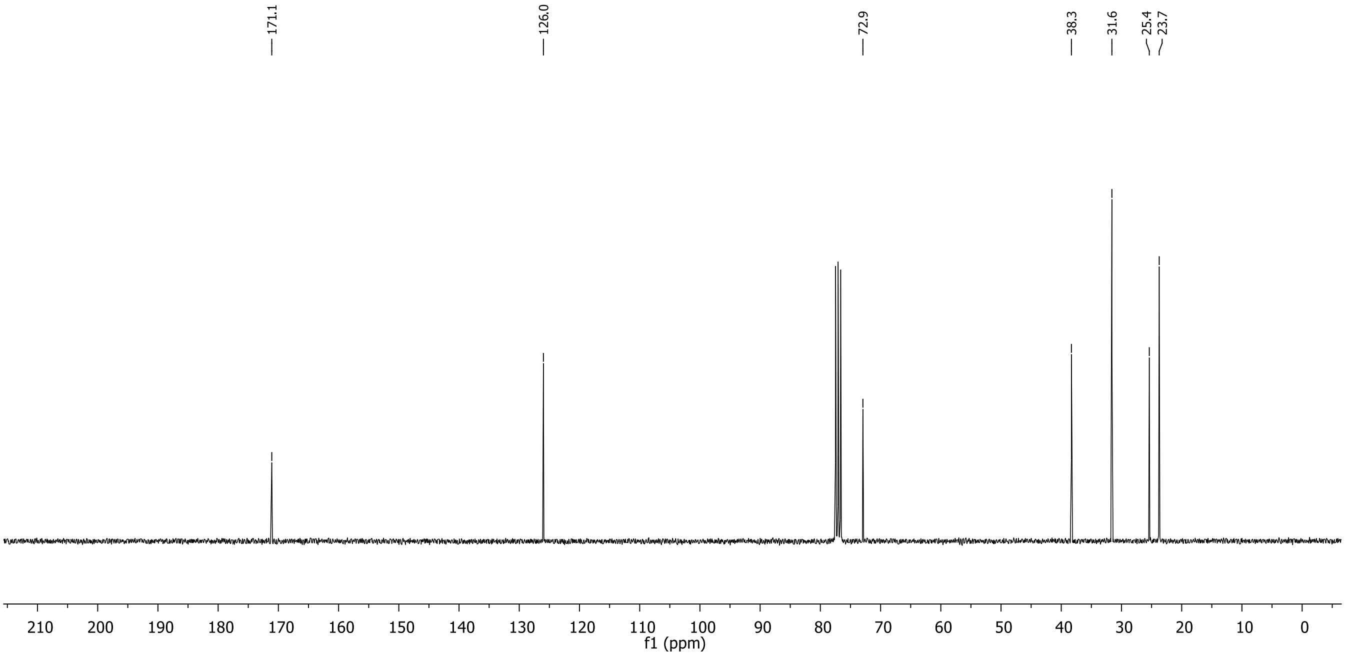

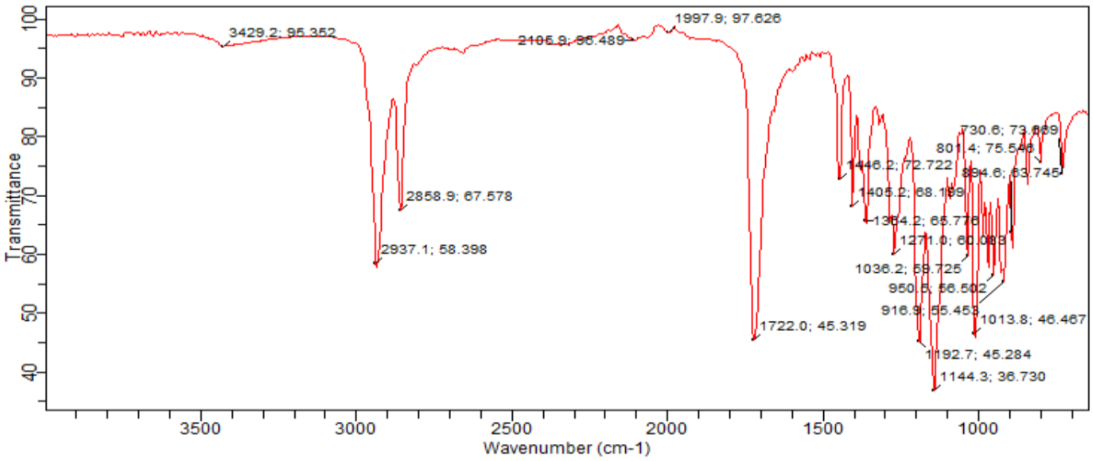

Diphenyl (*E*)-hex-3-enedioate (1p) (^{1}H NMR: 300 MHz, ^{13}C NMR: 75 MHz, $CDCl_3$):

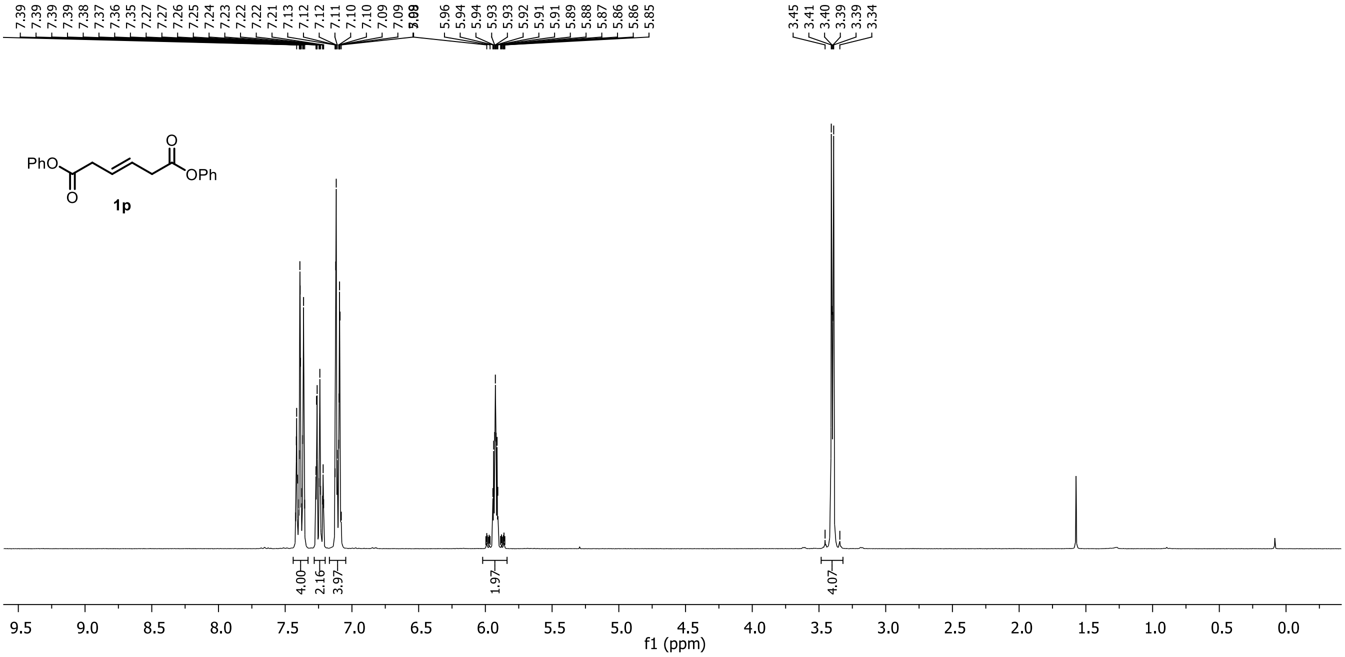

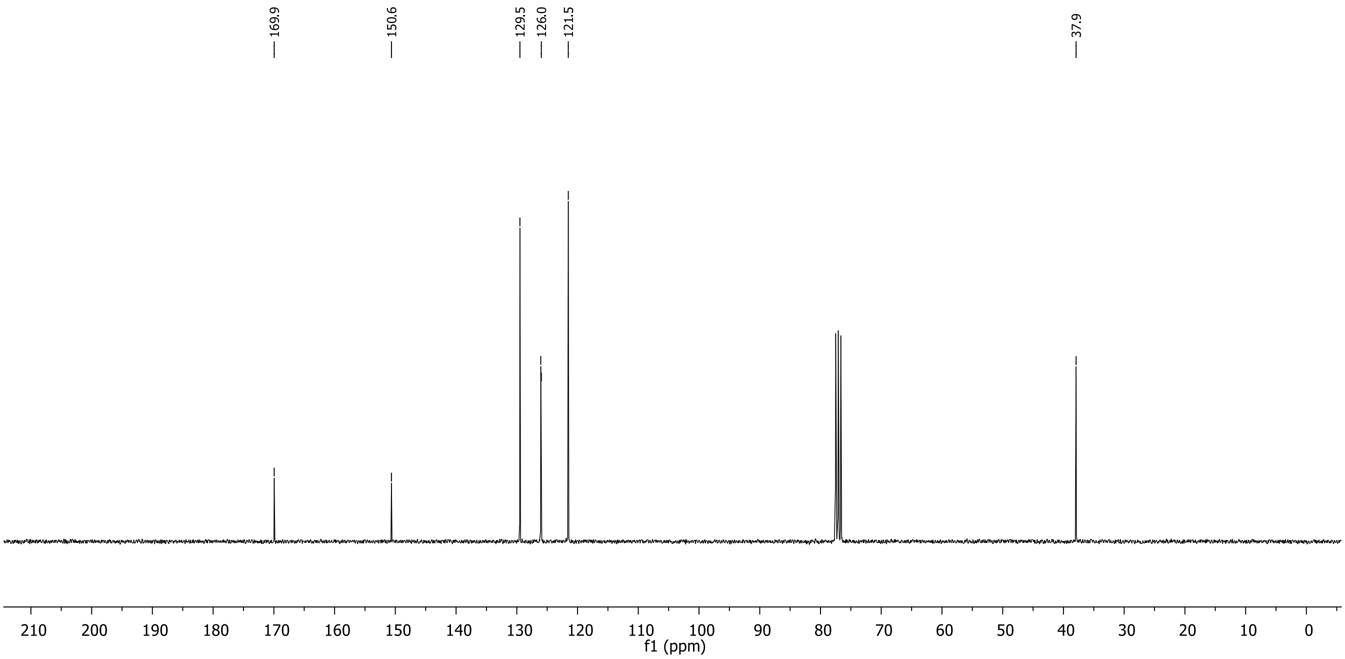

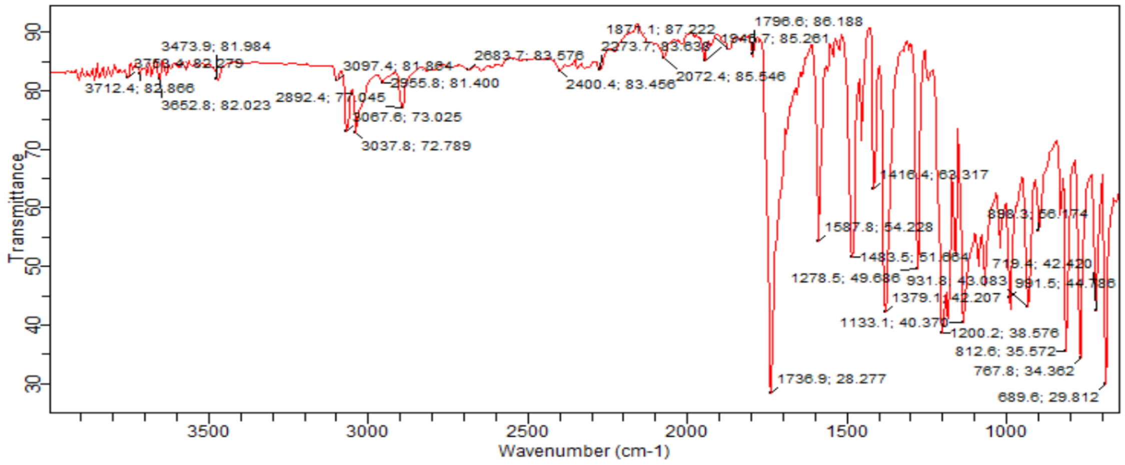

Diheptyl (*E*)-hex-3-enedioate (1q) (^{1}H NMR: 300 MHz, ^{13}C NMR: 75 MHz, $CDCl_3$):

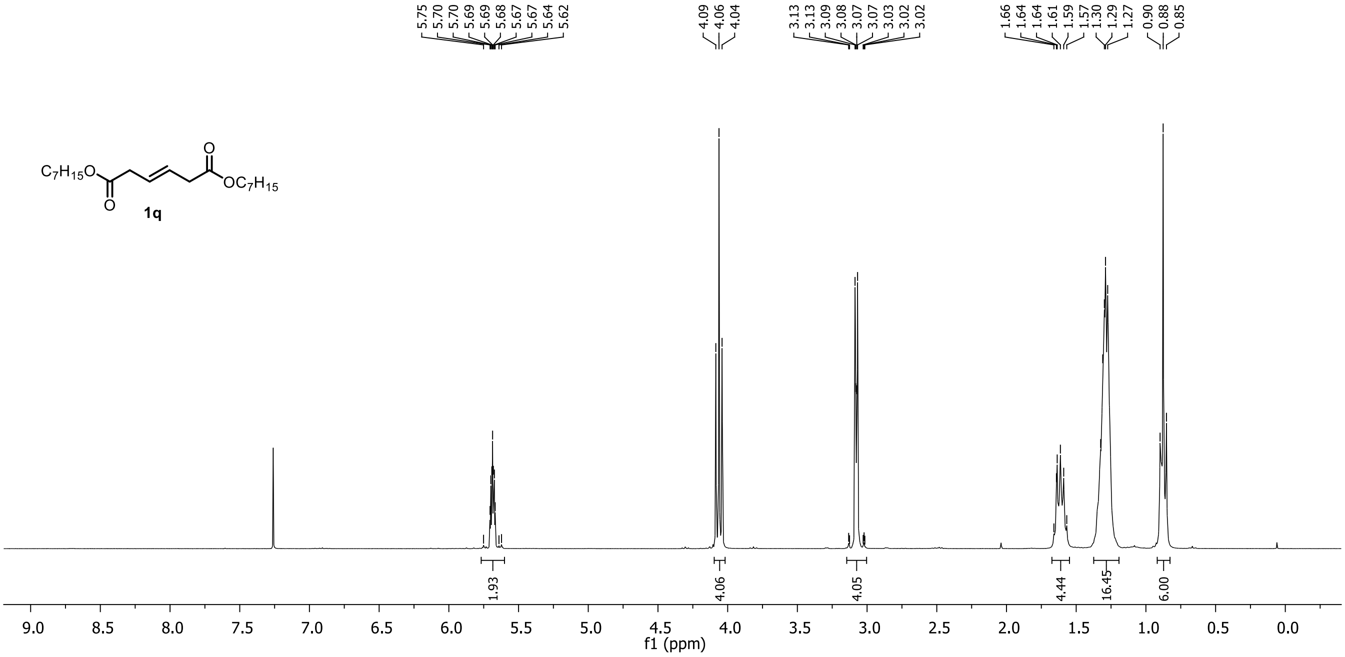

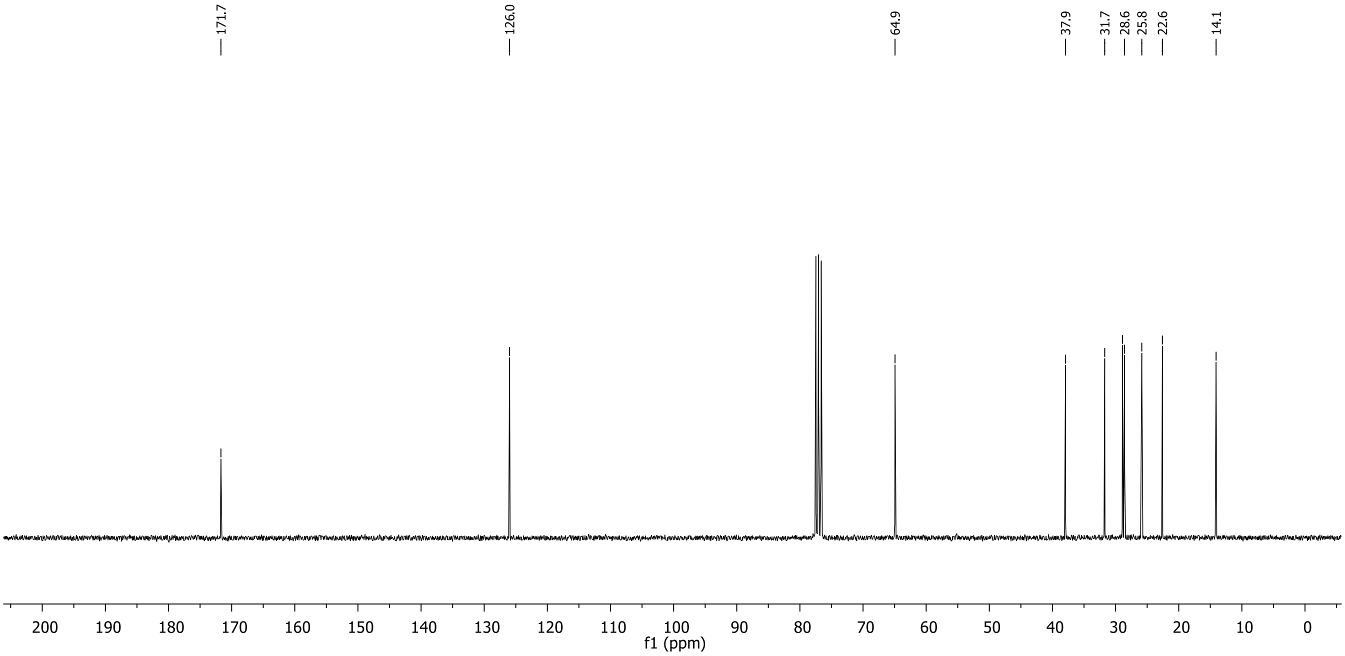

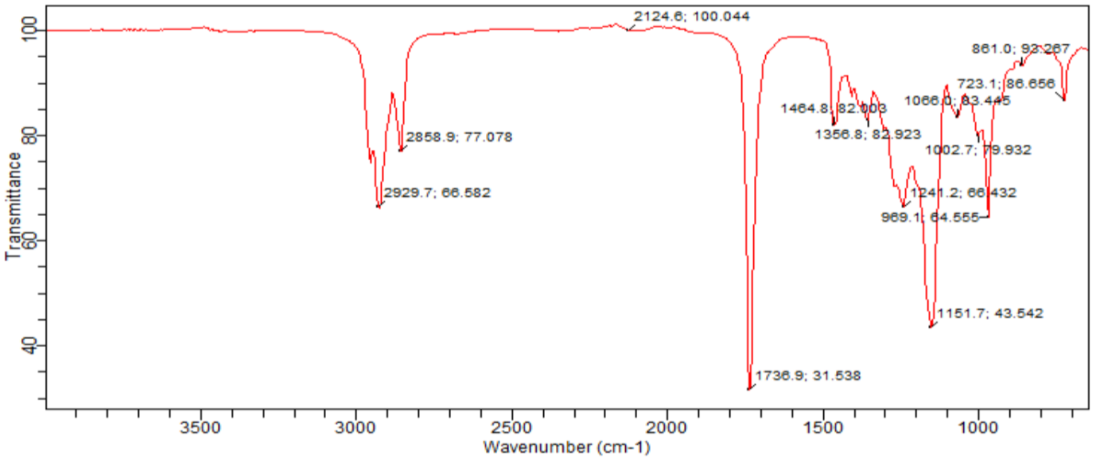

1-((1R,2S,5R)-2-isopropyl-5-methylcyclohexyl) 6-((1S,2R,5S)-2-isopropyl-5-methylcyclohexyl) (*E*)-hex-3-enedioate (1r) (^{1}H NMR: 300 MHz, ^{13}C NMR: 75 MHz, CDCl$_3$):

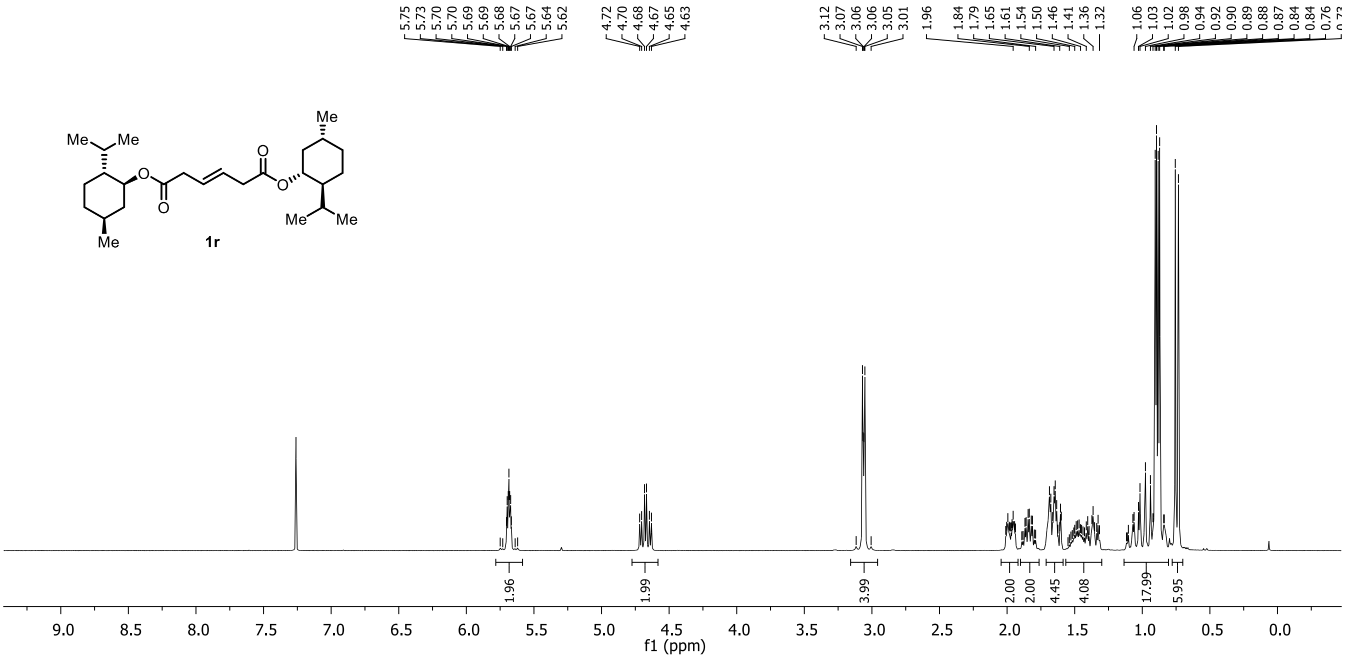

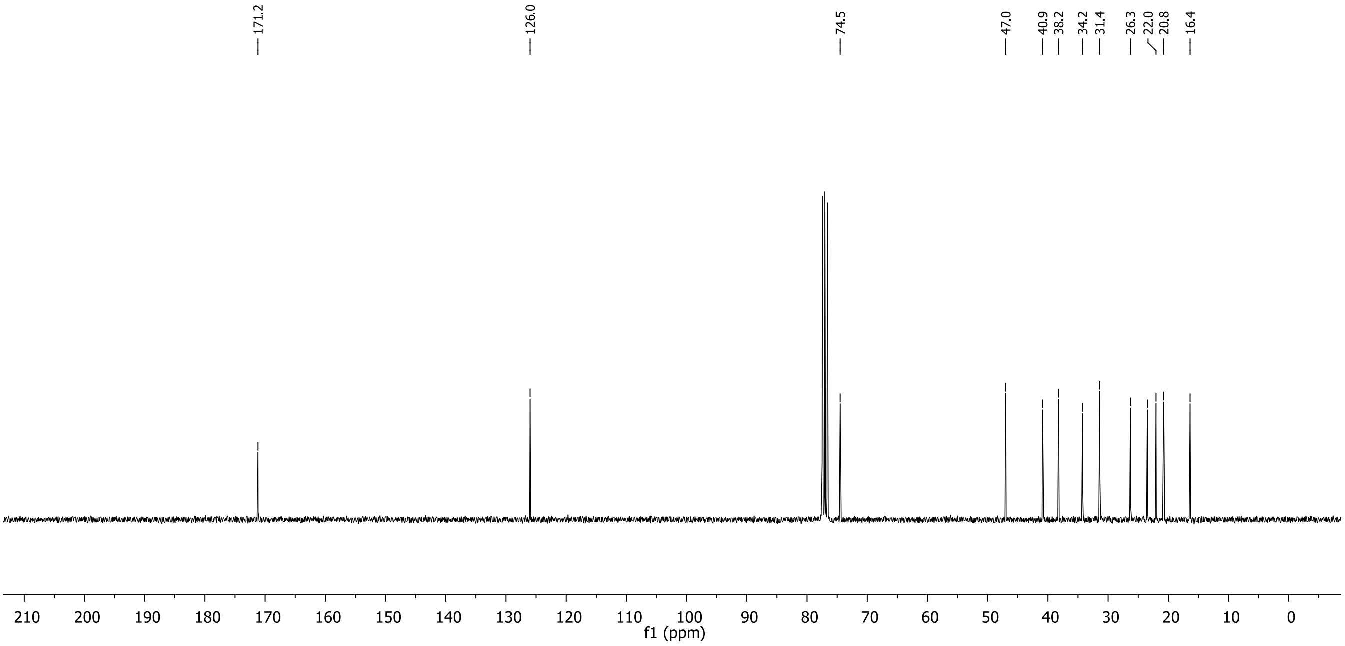

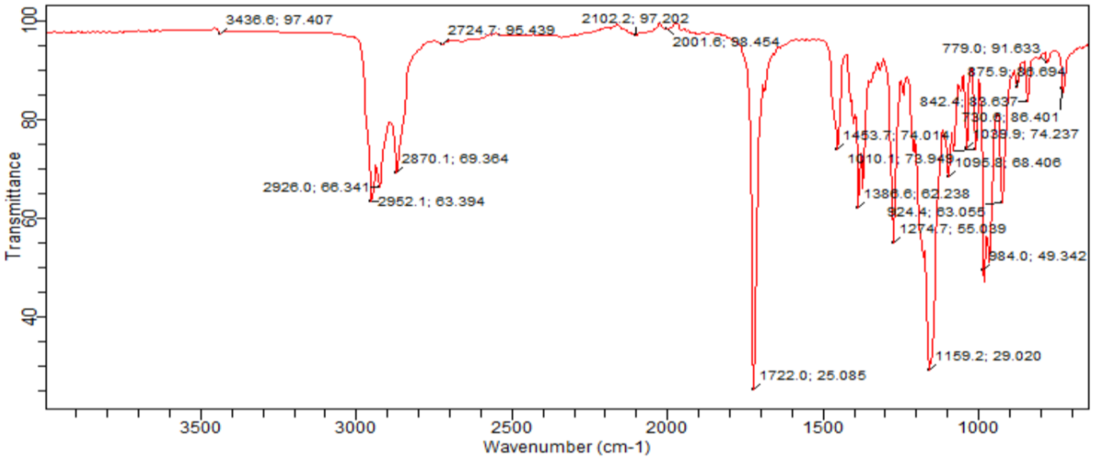

Cyclopropylmethyl (*E*)-hex-3-enoate (1u) (^{1}H NMR: 300 MHz, ^{13}C NMR: 75 MHz, CDCl$_3$):

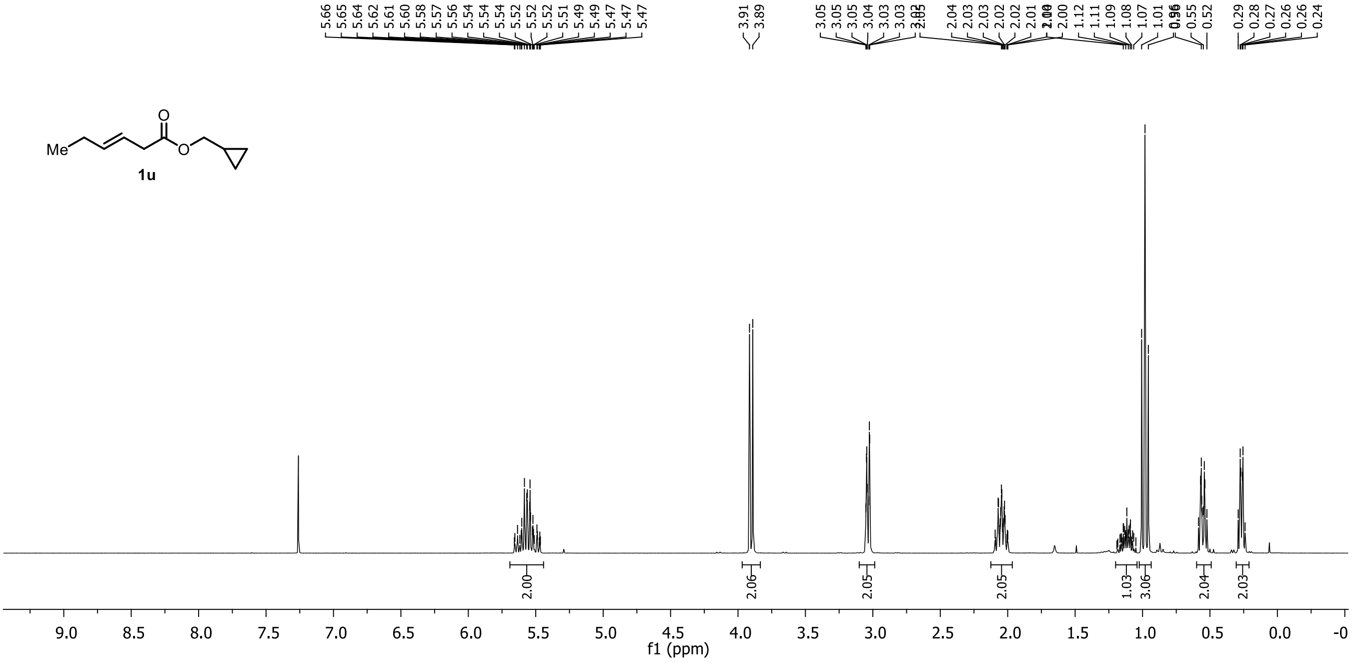

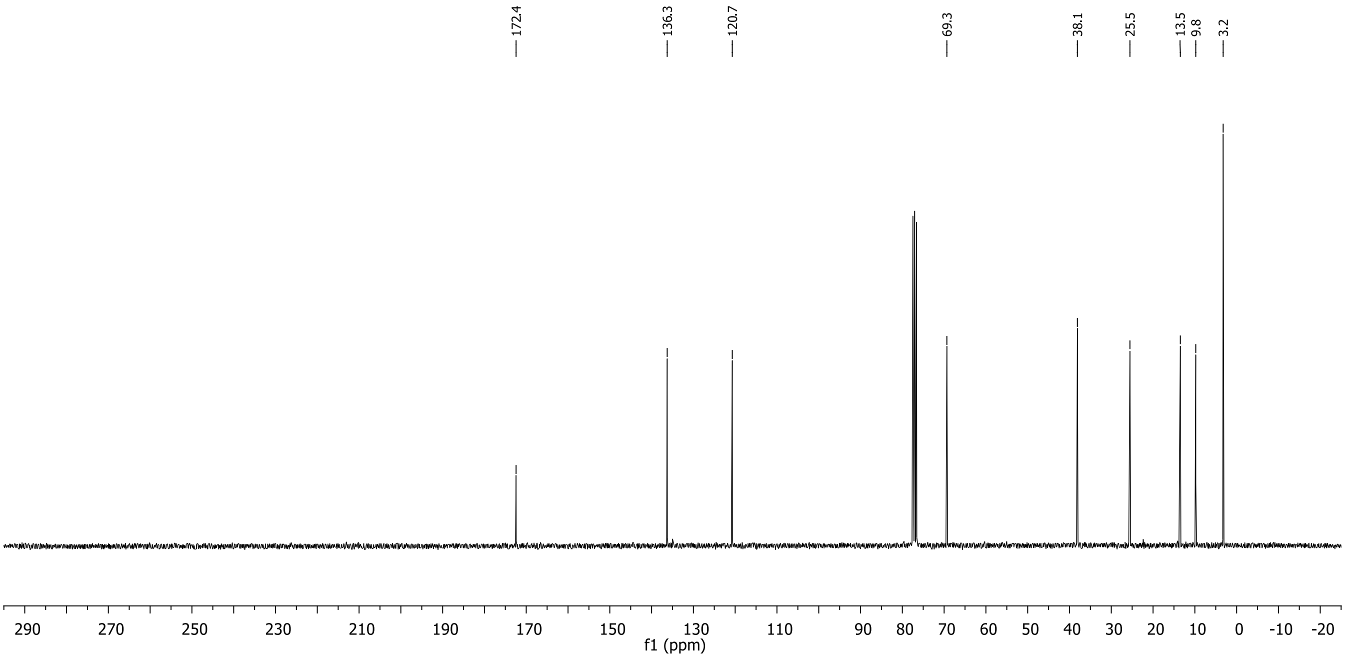

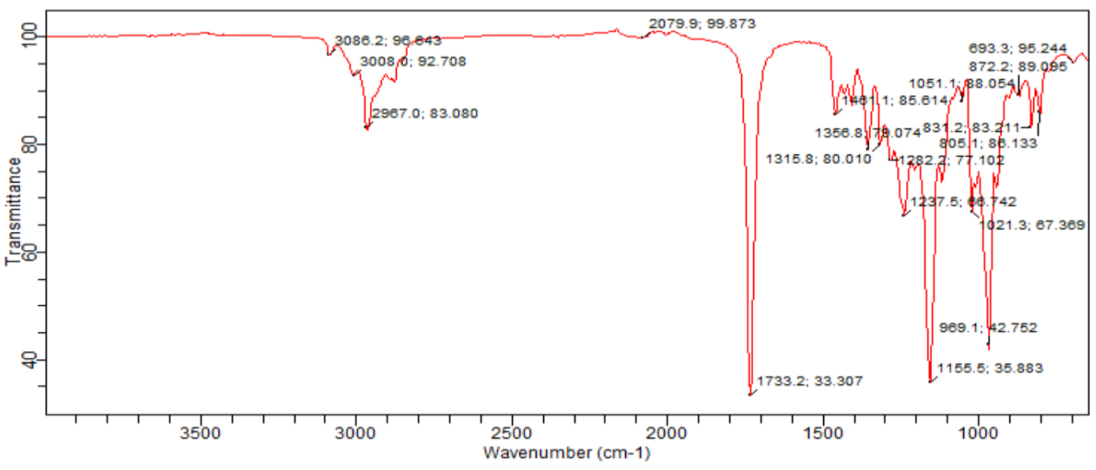

Oxiran-2-ylmethyl (*E*)-hex-3-enoate (1v) (^{1}H NMR: 300 MHz, ^{13}C NMR: 75 MHz, $CDCl_3$):

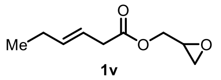

1v

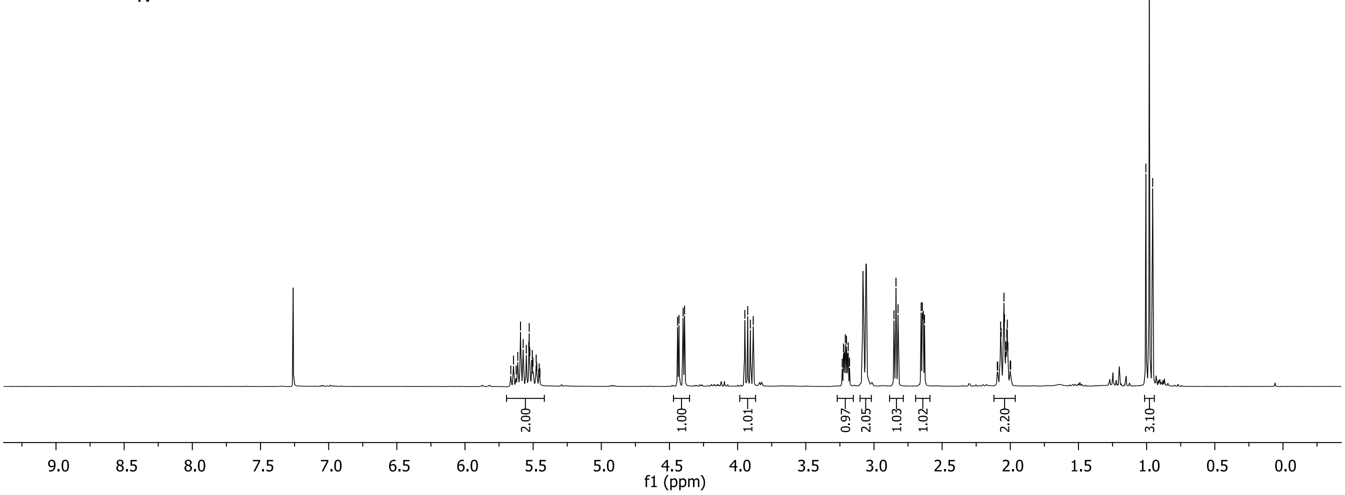

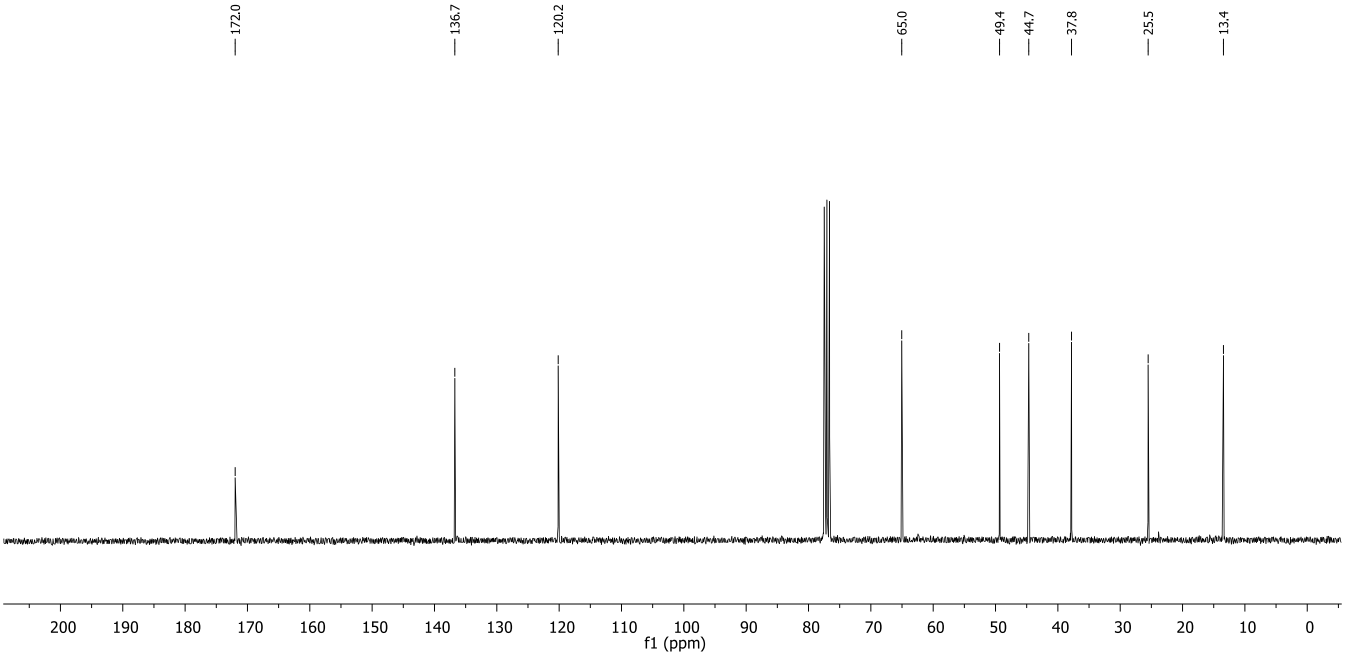

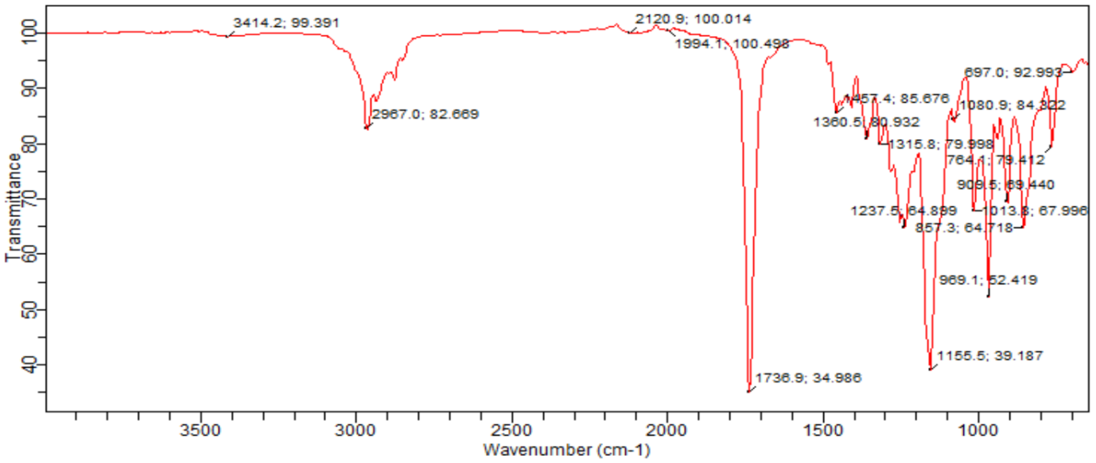

Pent-3-yn-1-yl (*E*)-hex-3-enoate (1w) (^{1}H NMR: 300 MHz, ^{13}C NMR: 75 MHz, CDCl$_3$):

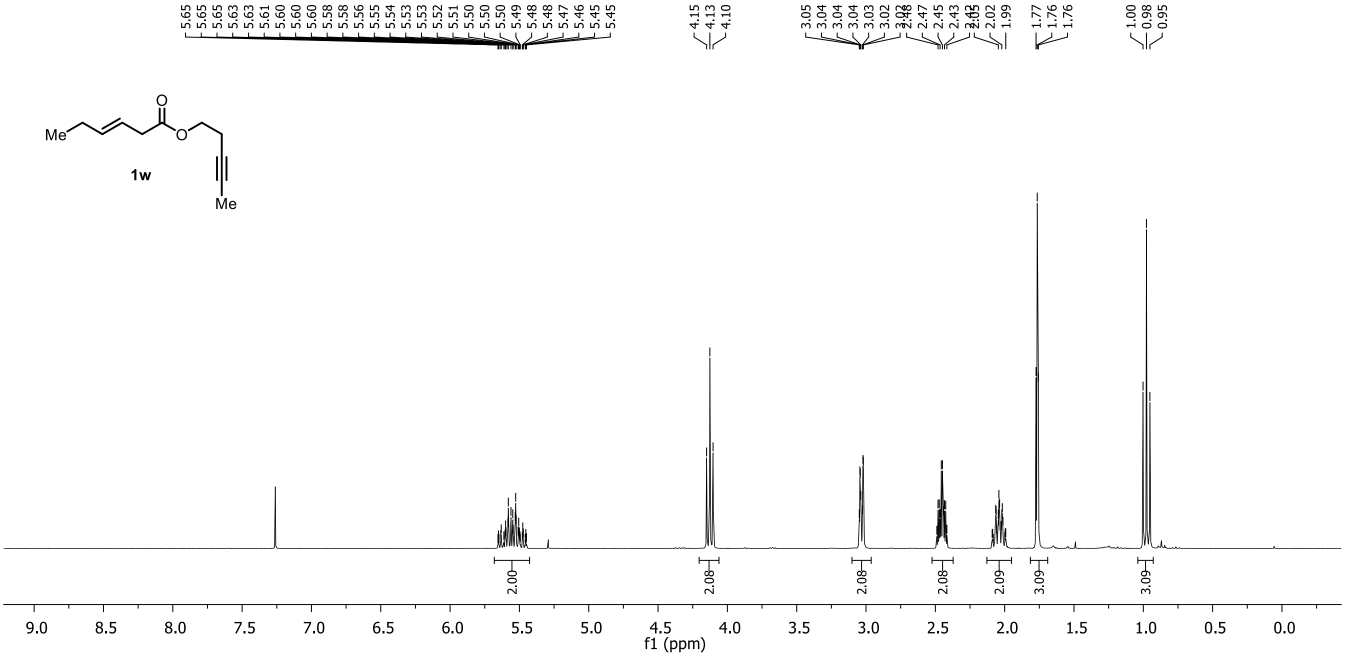

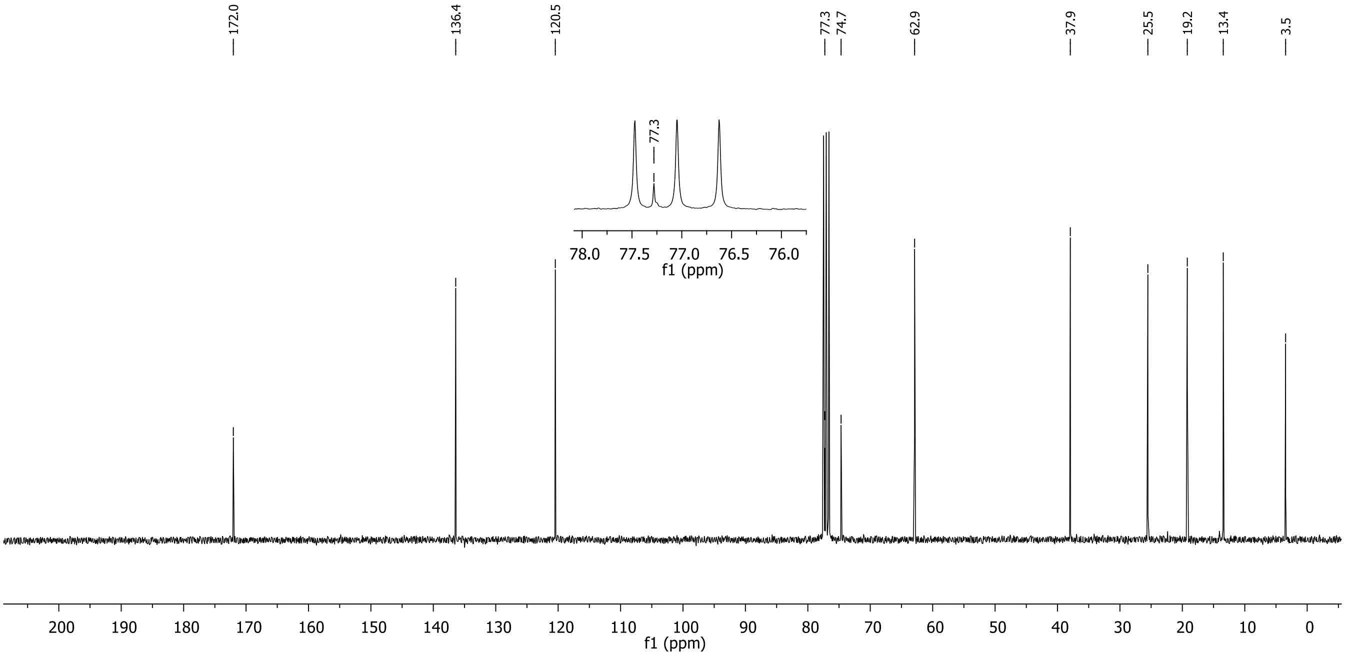

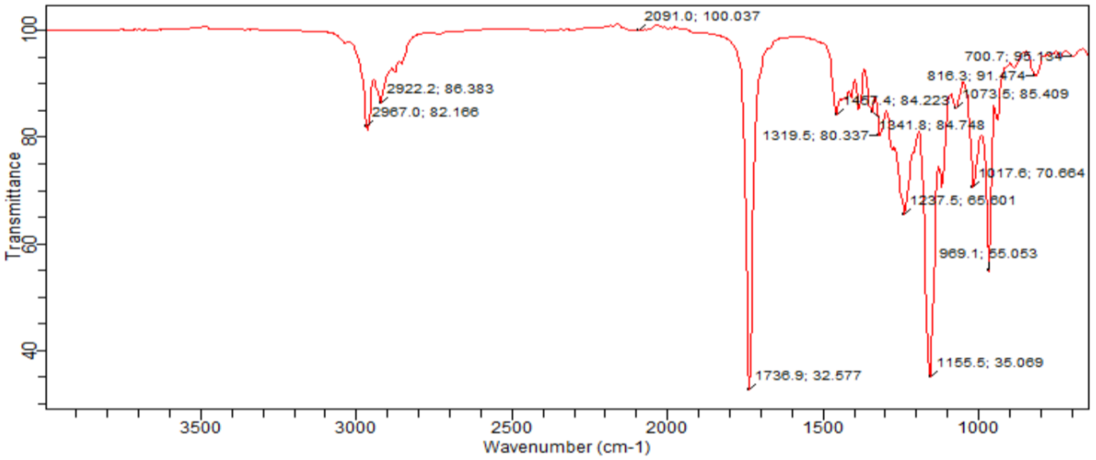

4-Oxocyclopent-2-en-1-yl (*E*)-hex-3-enoate (1x) (^{1}H NMR: 300 MHz, ^{13}C NMR: 75 MHz, $CDCl_3$):

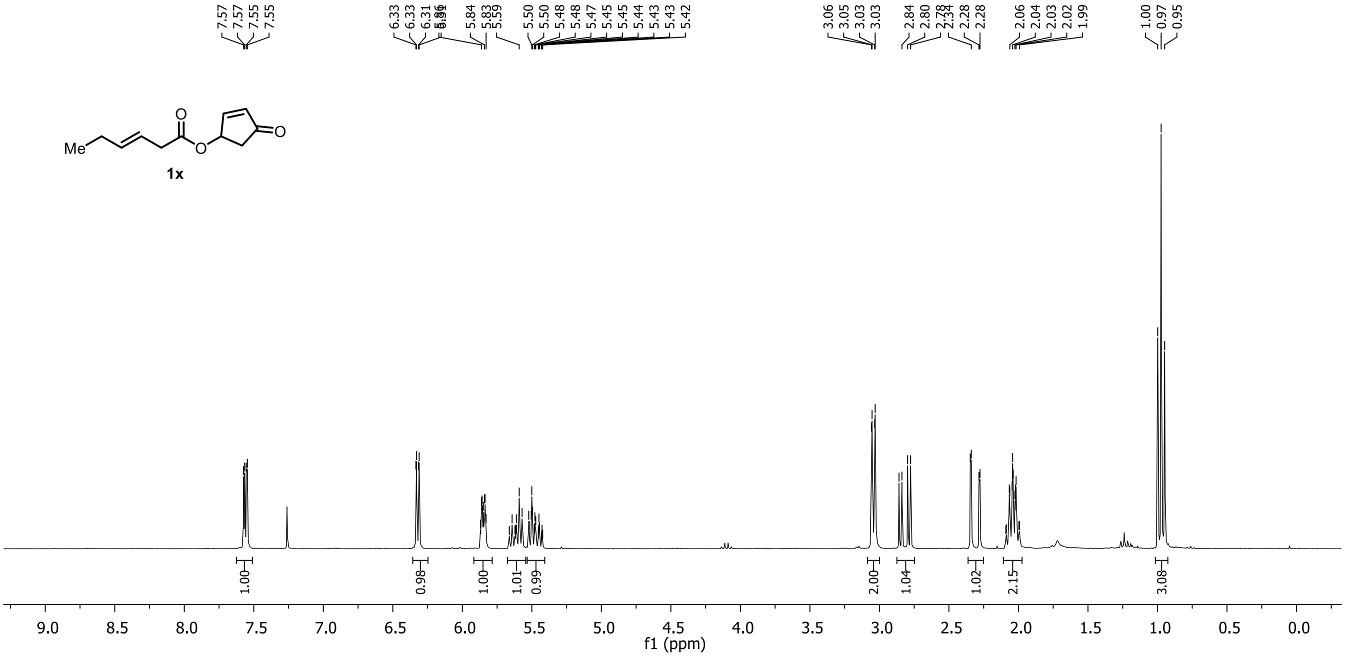

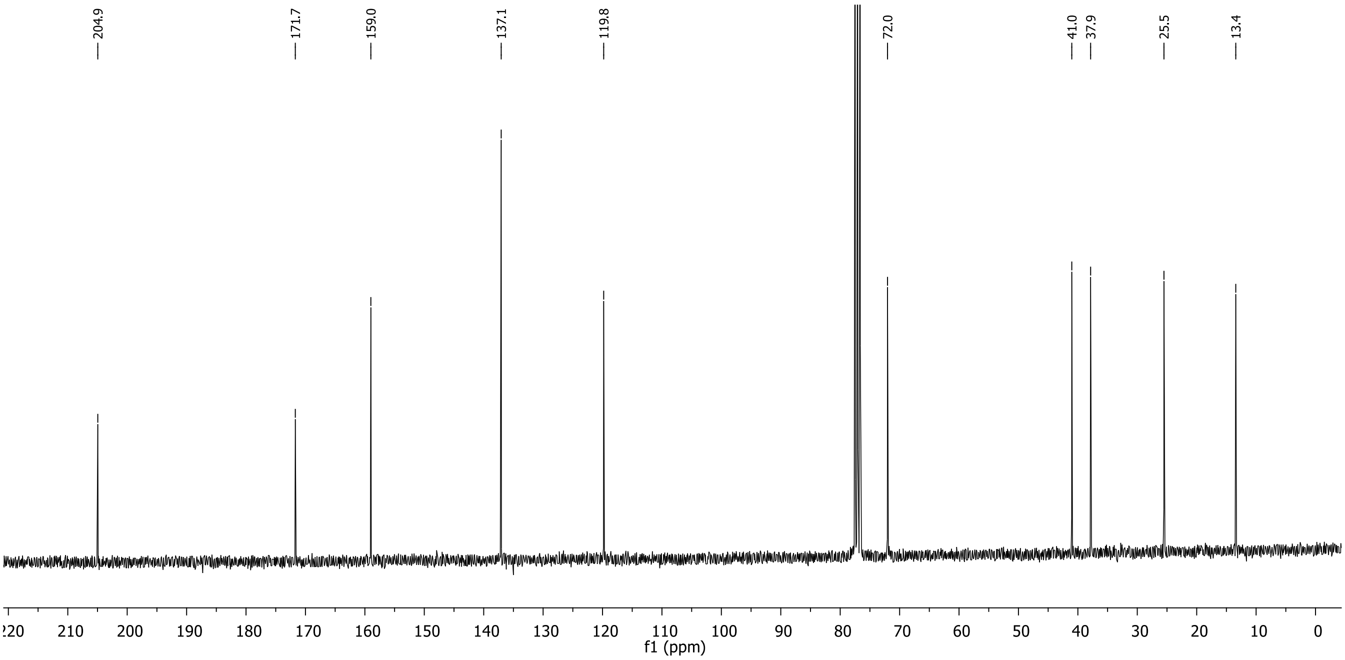

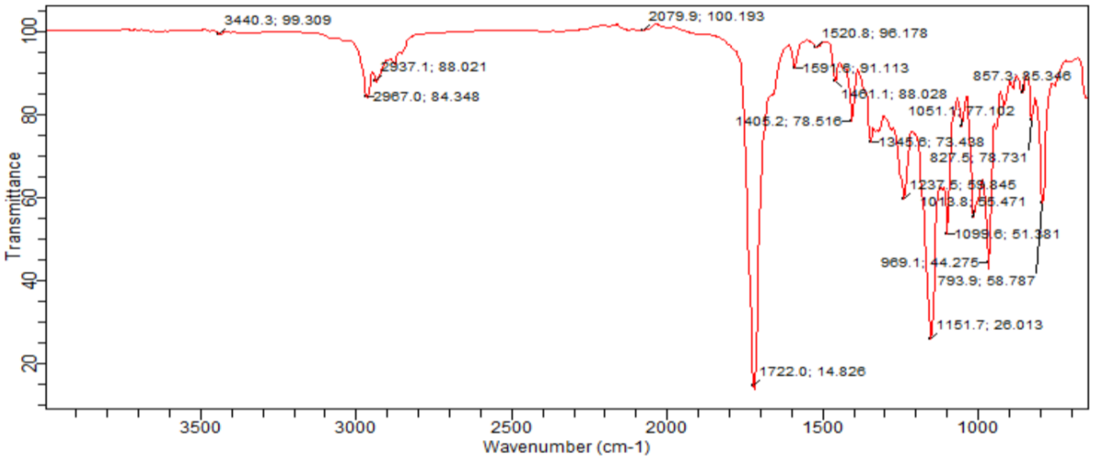

Cyclopent-3-en-1-yl (*E*)-hex-3-enoate (1y) (^{1}H NMR: 300 MHz, ^{13}C NMR: 75 MHz, CDCl₃):

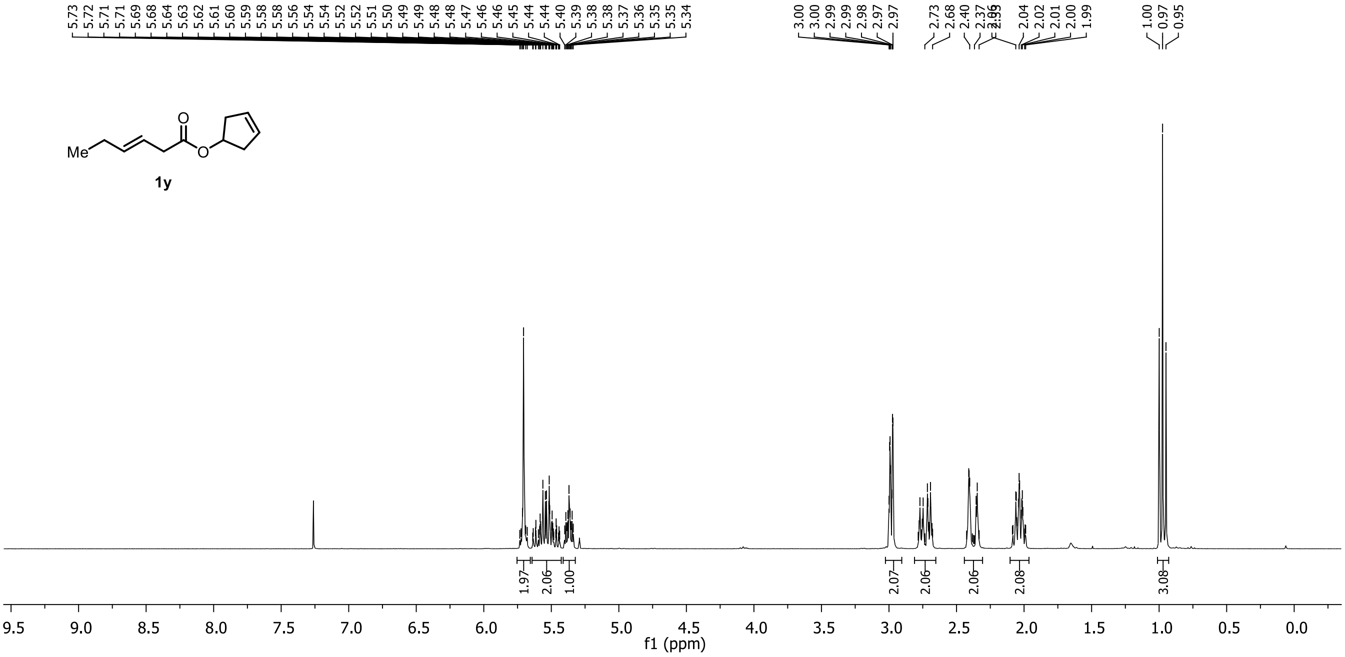

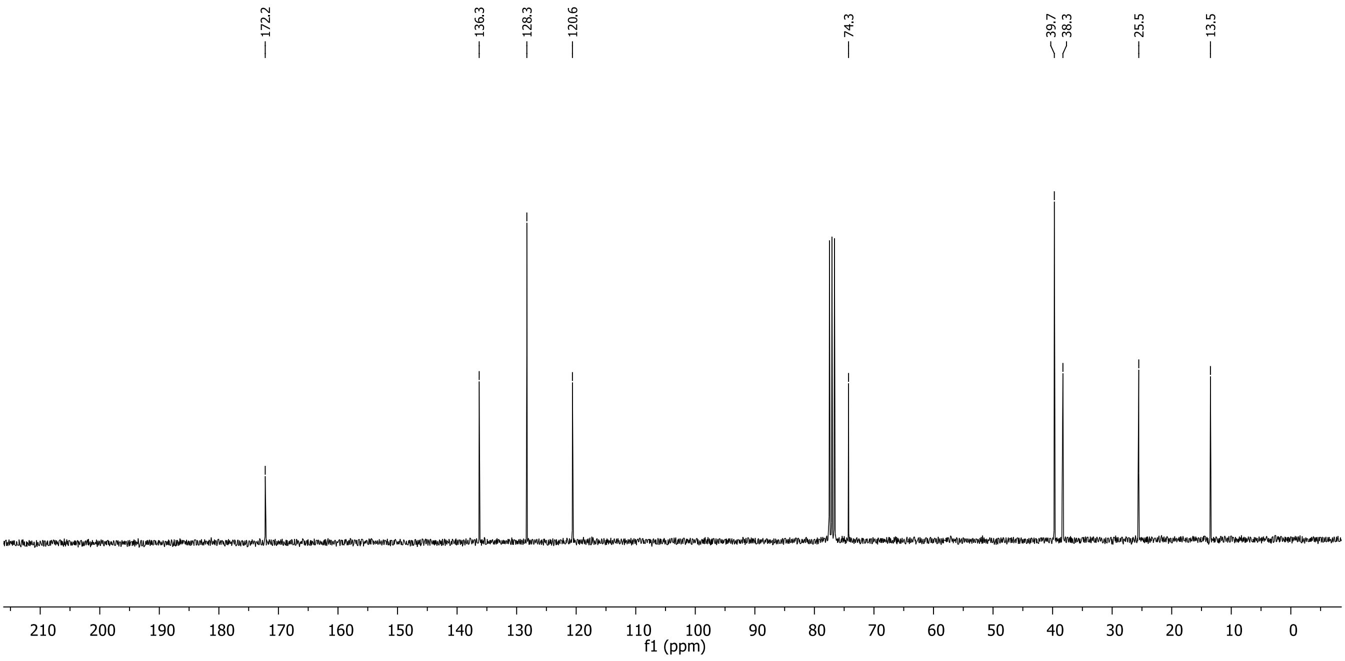

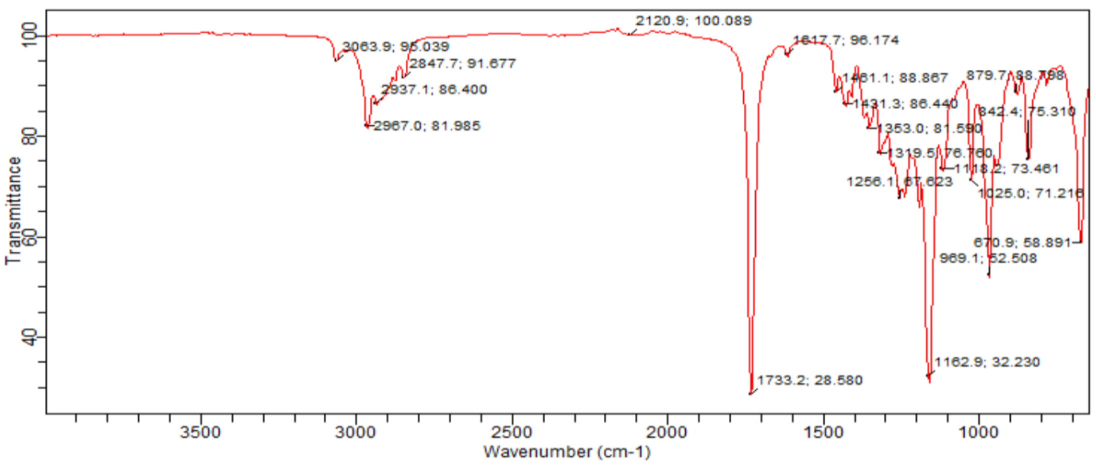

Ethyl (*E*)-2-ethylhex-3-enoate (1z) (^{1}H NMR: 300 MHz, ^{13}C NMR: 75 MHz, CDCl$_3$):

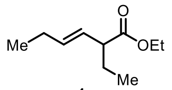

1z

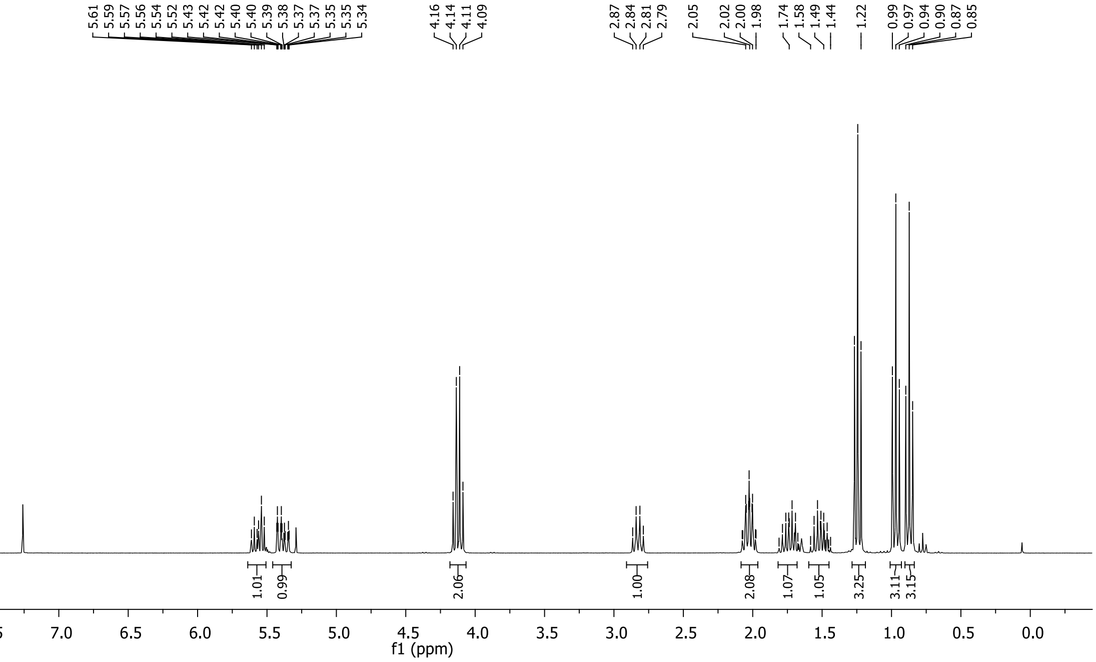

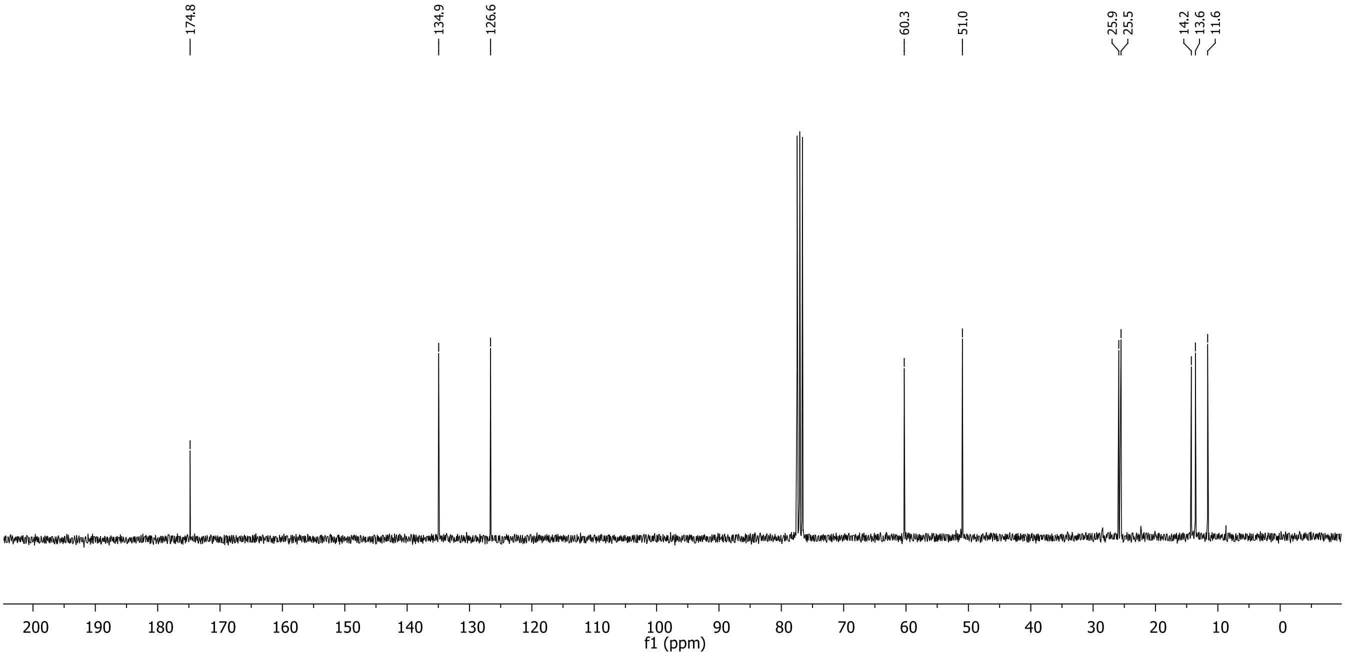

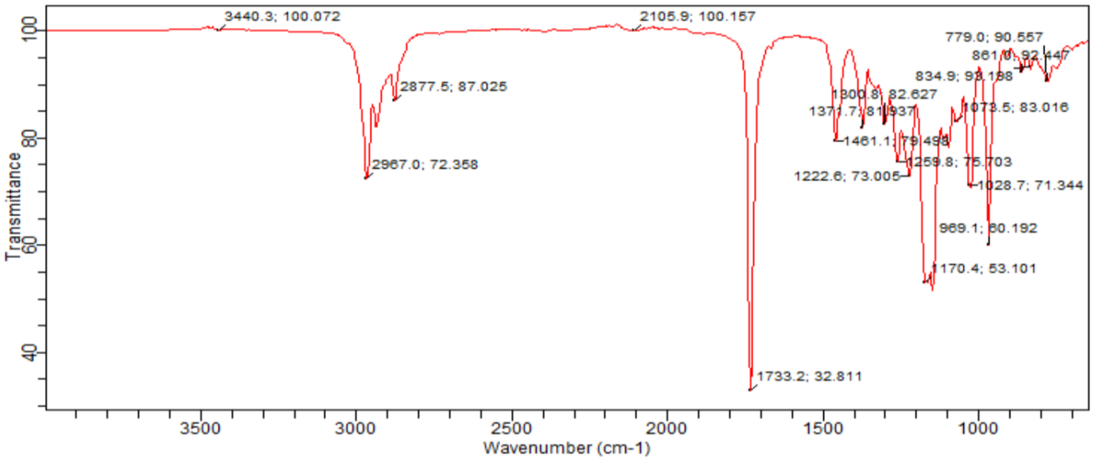

Butyl 2-methylbut-3-enoate (1aa) (^{1}H NMR: 300 MHz, ^{13}C NMR: 75 MHz, $CDCl_3$):

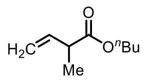

1aa

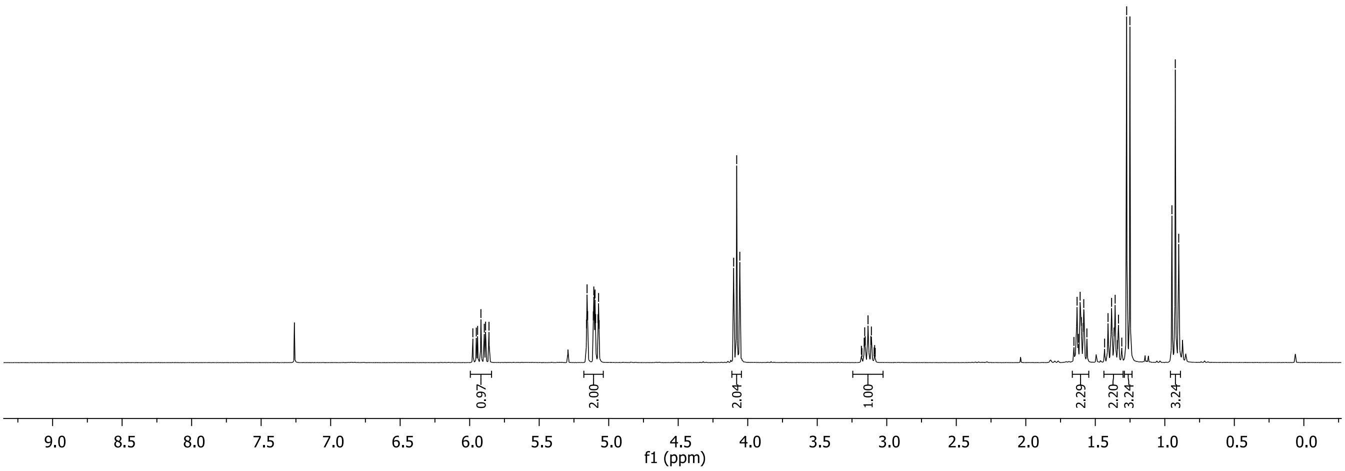

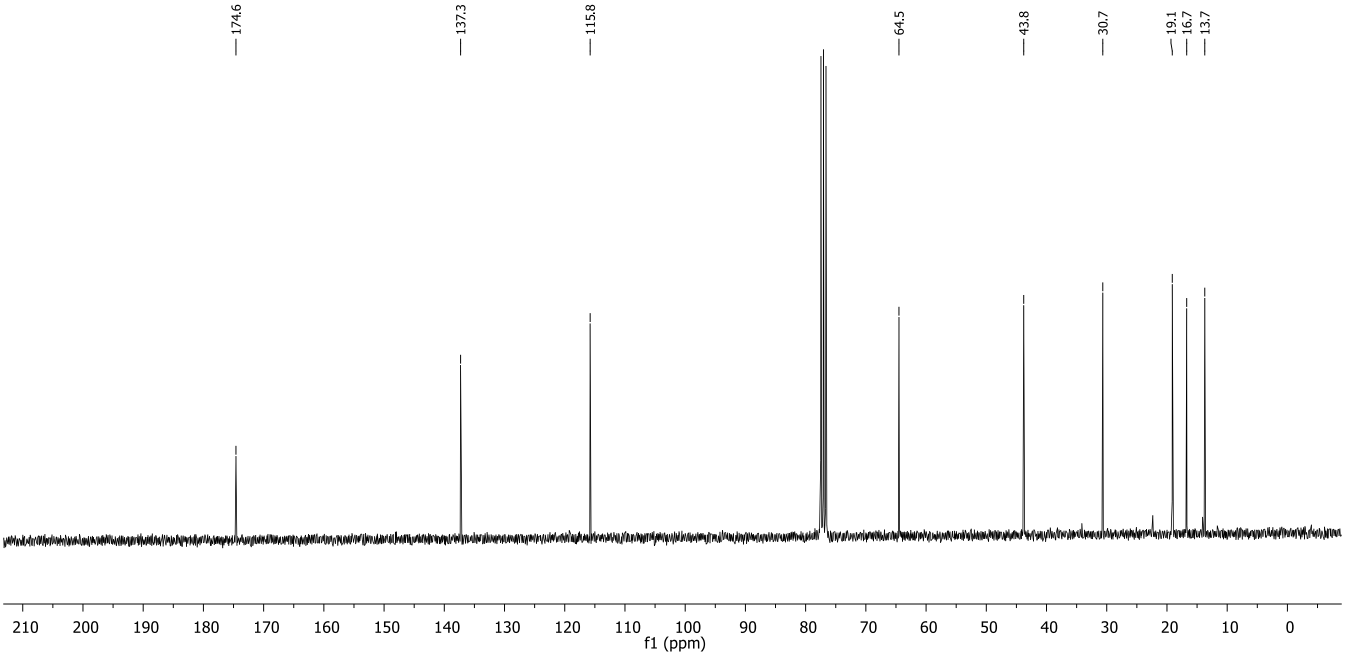

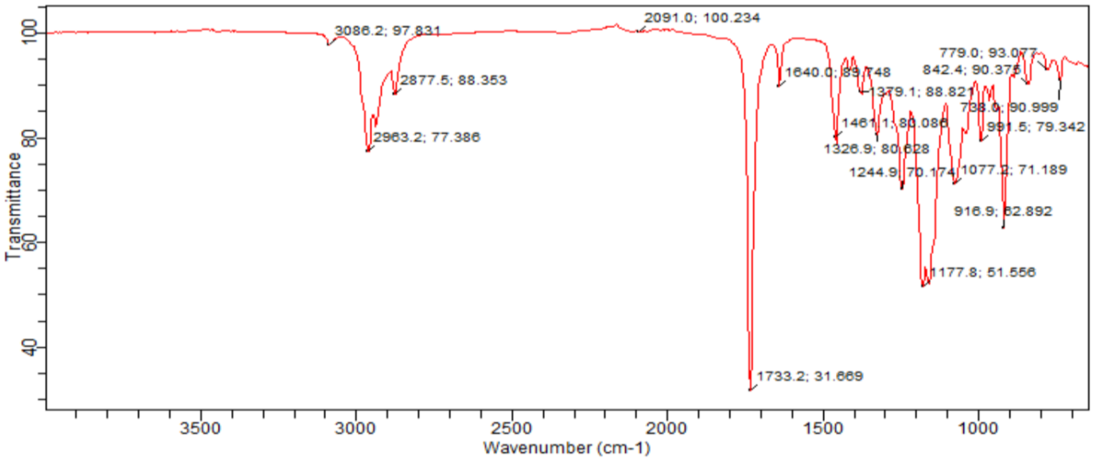

Diethyl (*E*)-2-(hex-1-en-1-yl)malonate (1ab):

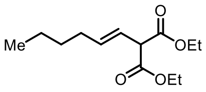

1ab

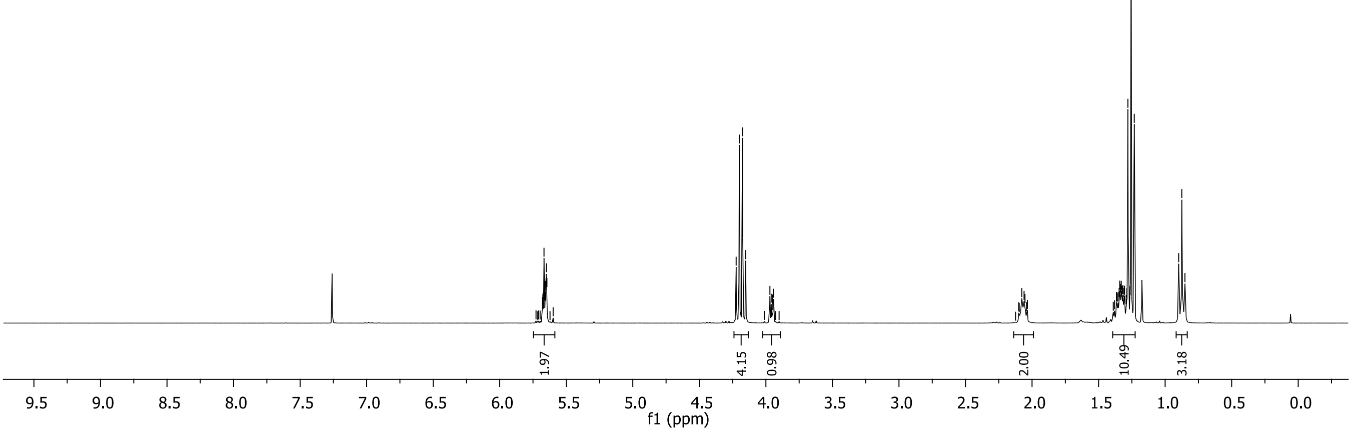

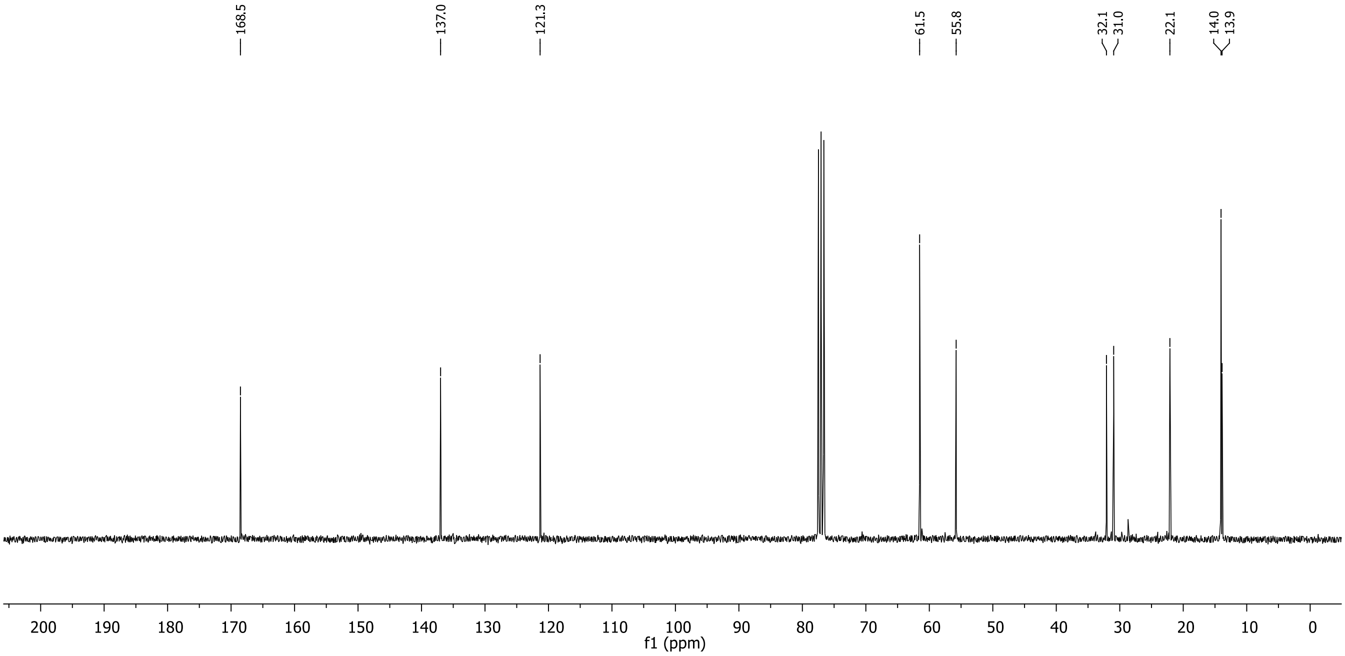

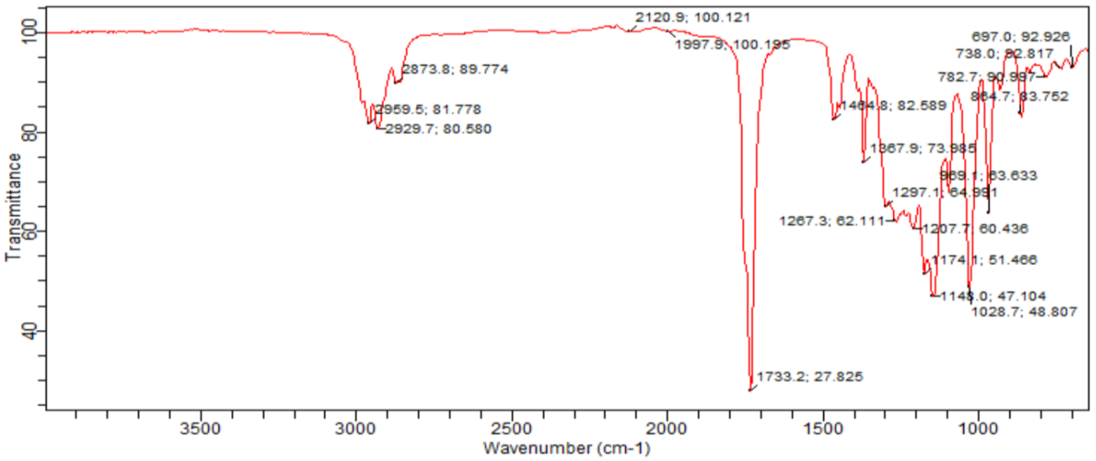

Butyl 4-methylpent-3-enoate (1ac) (^{1}H NMR: 300 MHz, ^{13}C NMR: 75 MHz, CDCl₃):

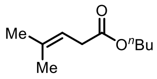

1ac

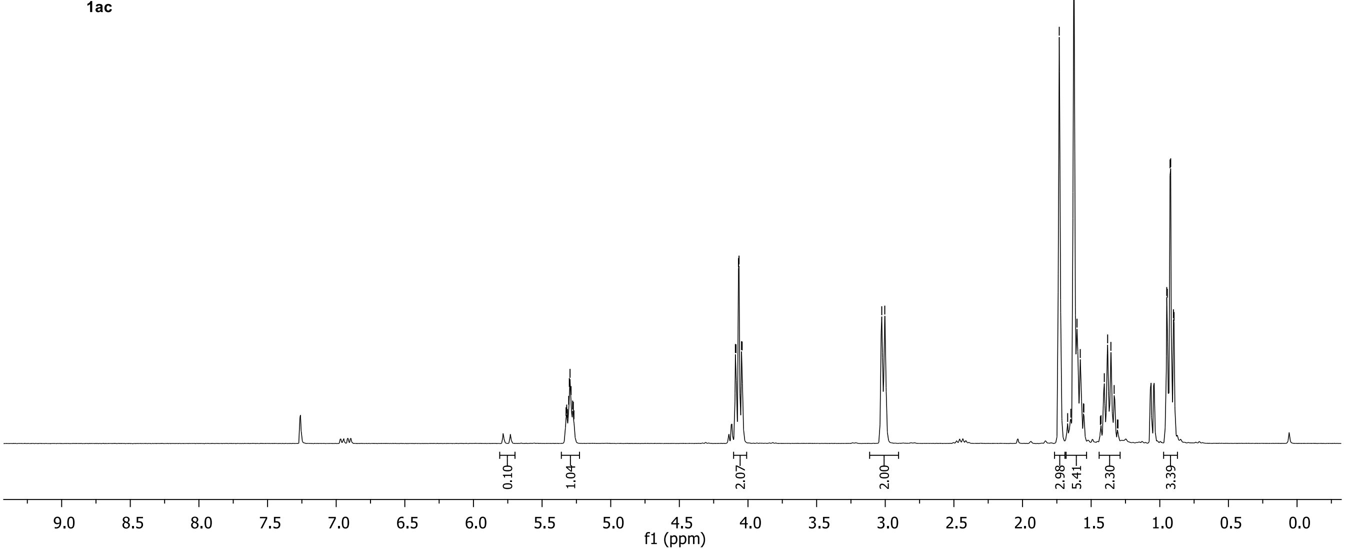

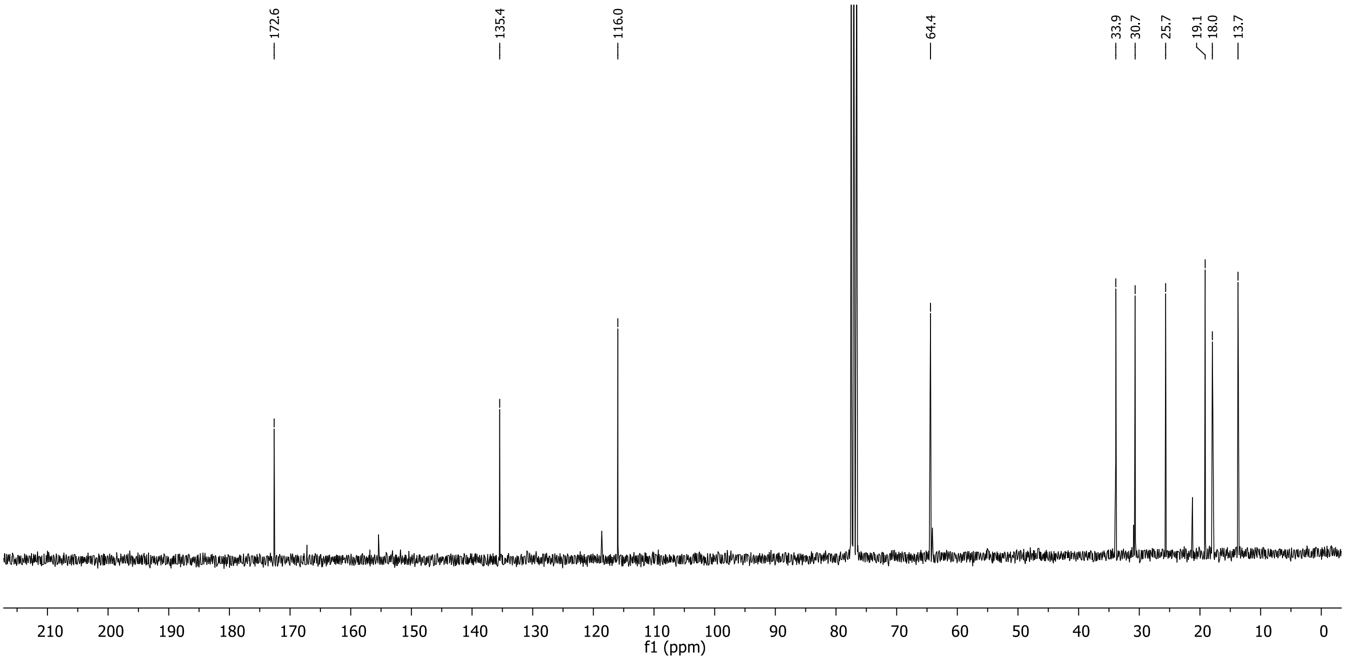

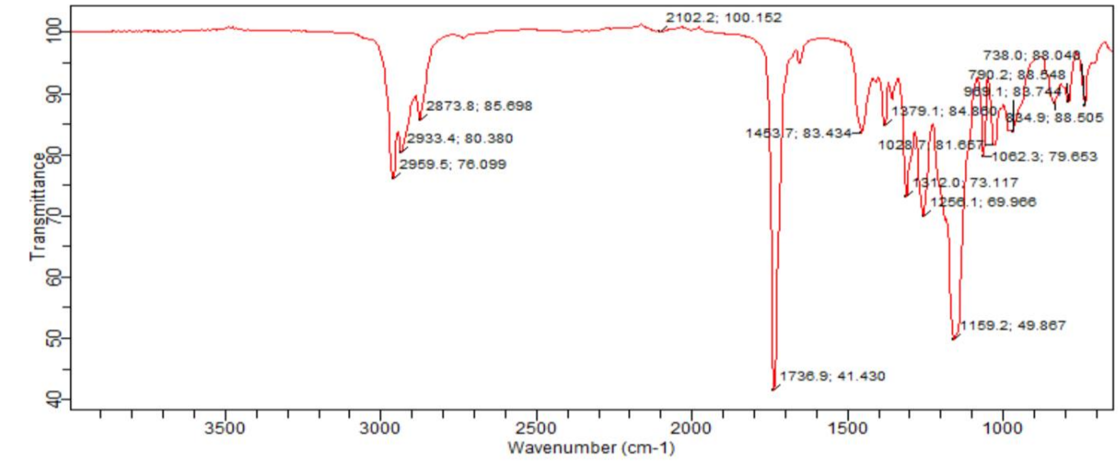

***tert*-Butyl((3,7-dimethyloct-6-en-1-yl)oxy)dimethylsilane (1ao) (^{1}H NMR: 300 MHz, ^{13}C NMR: 75 MHz, $CDCl_3$):**

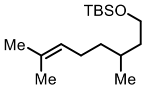

1ao

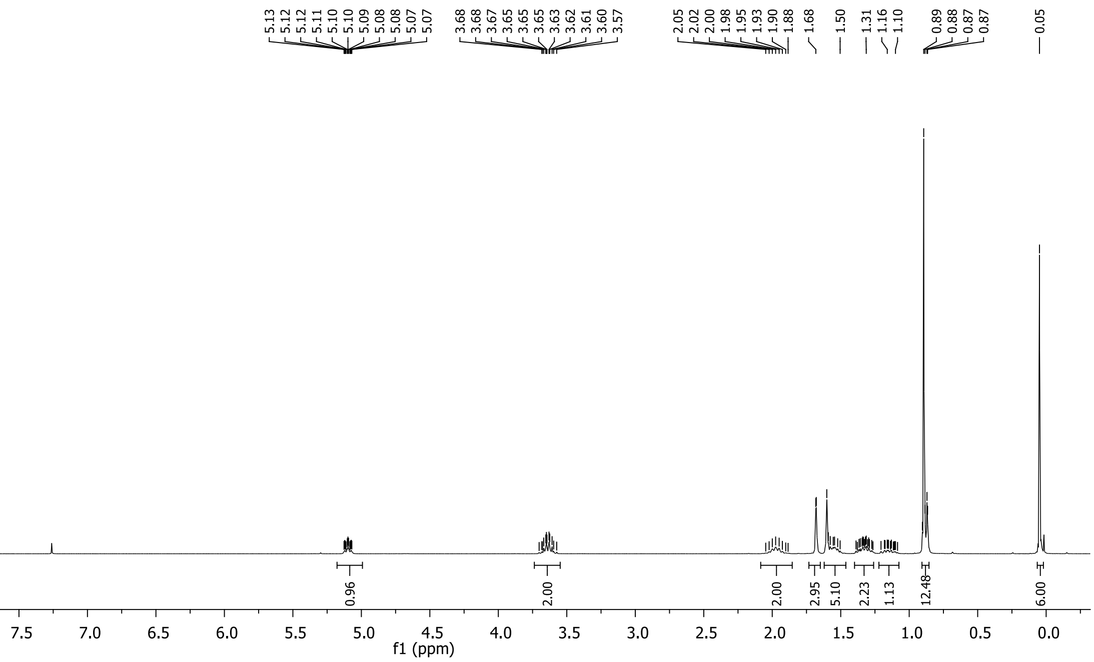

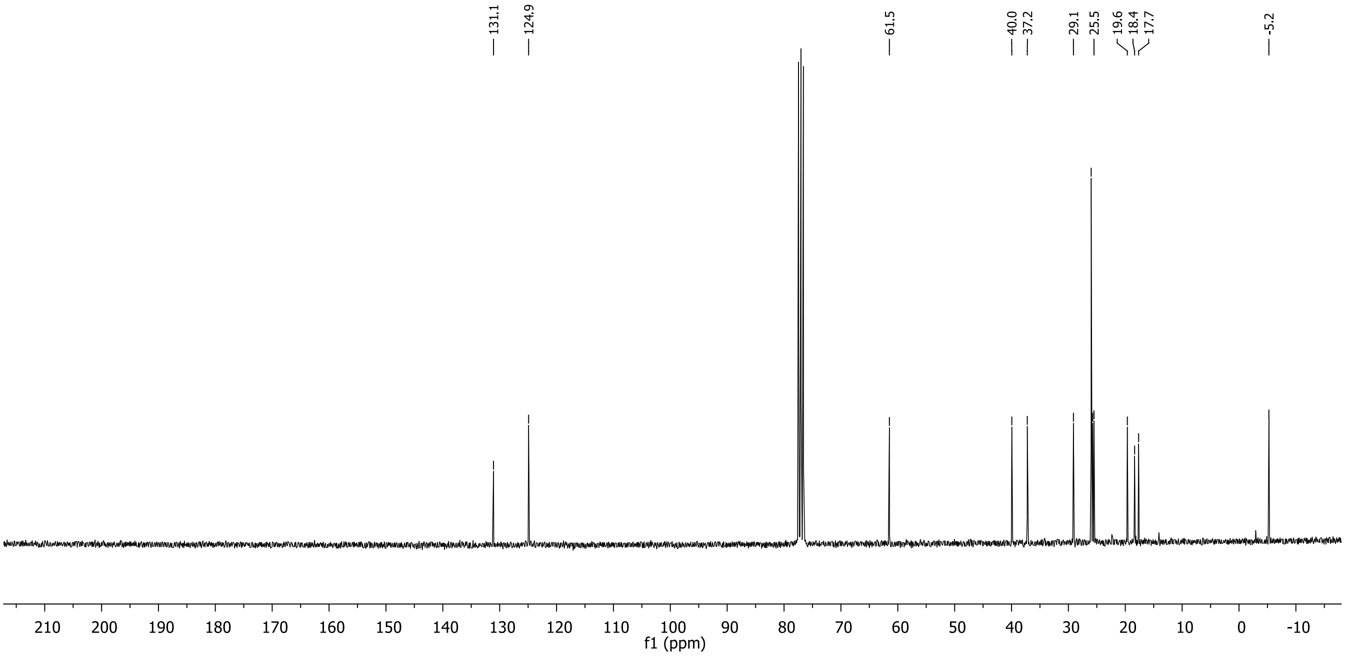

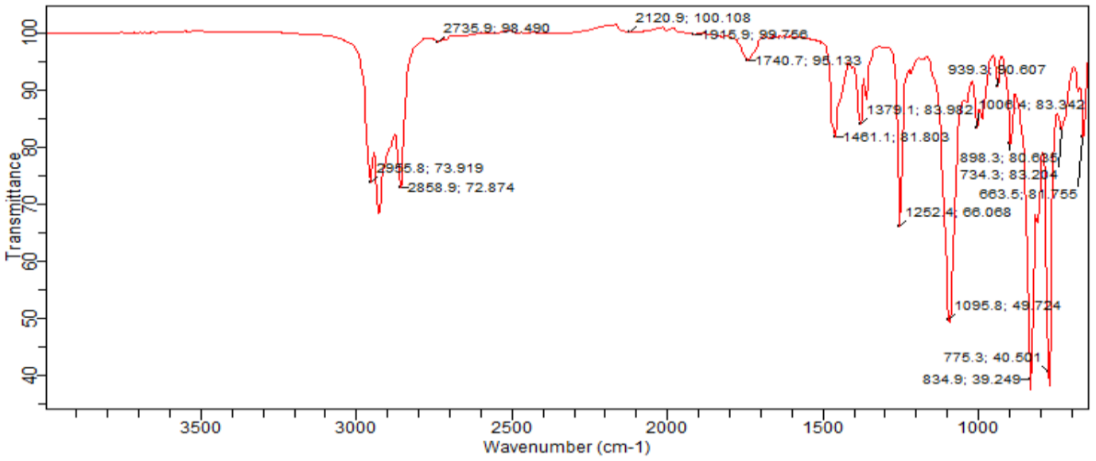

(8R,9S,10R,13S,14S,17S)-10,13-dimethyl-3-oxo-2,3,6,7,8,9,10,11,12,13,14,15,16,17-tetradecahydro-1H-cyclopenta[a]phenanthren-17-yl (*E*)-hex-3-enoate (1ap) (^{1}H NMR: 300 MHz, ^{13}C NMR: 75 MHz, $CDCl_3$):

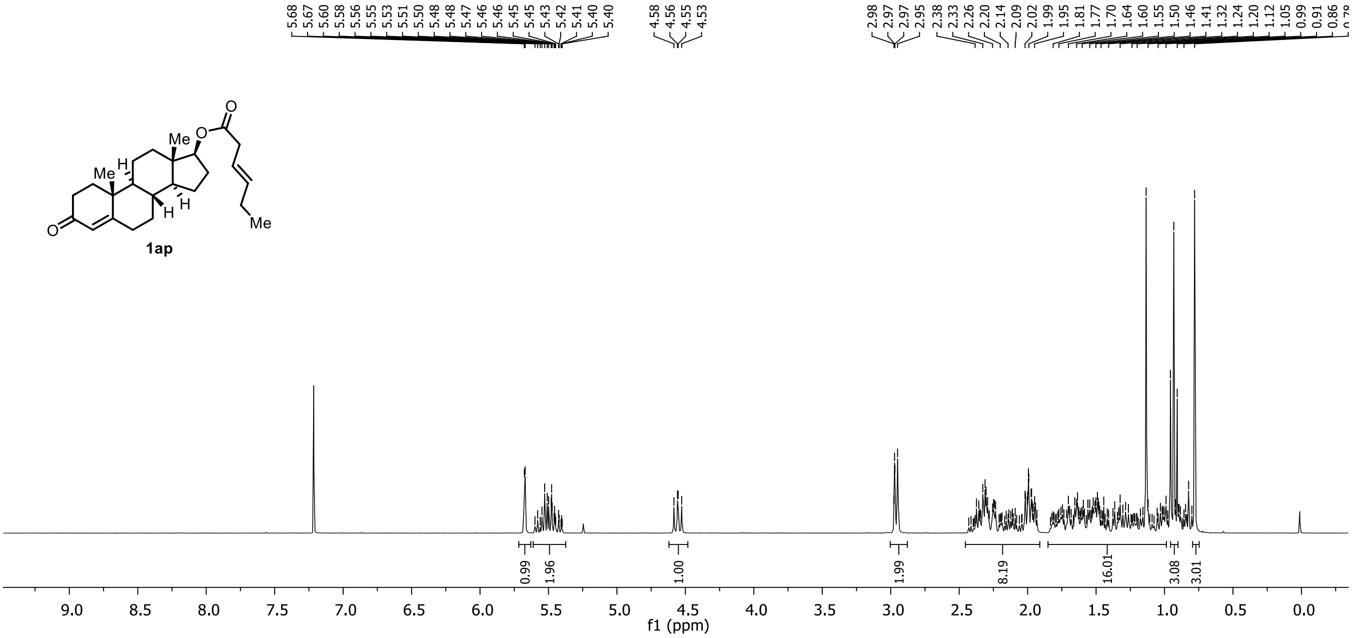

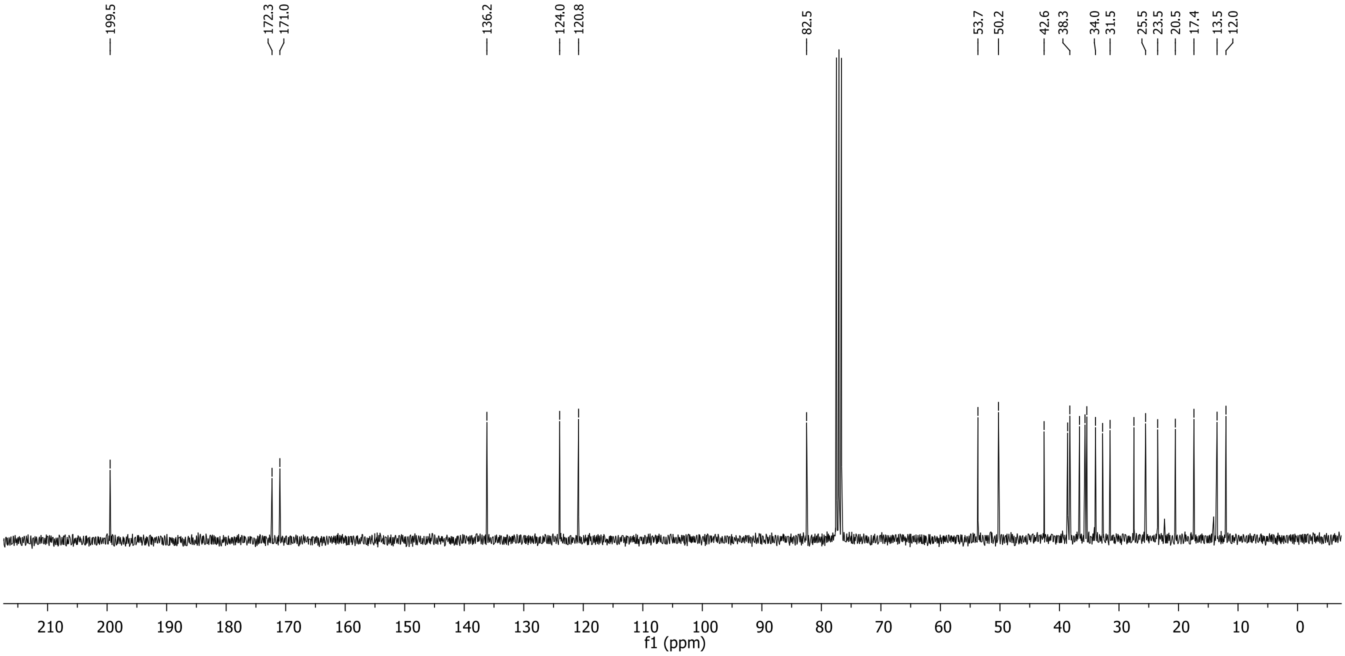

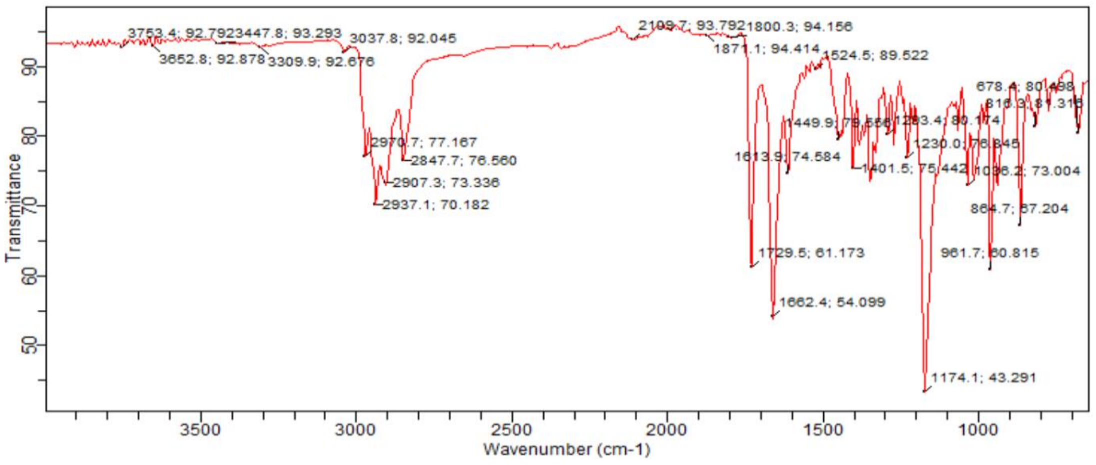

Ethyl (*E*)-4-(4-chloro-1H-pyrazol-1-yl)hex-2-enoate (3a) (^{1}H NMR: 300 MHz, ^{13}C NMR: 75 MHz, $CDCl_3$):

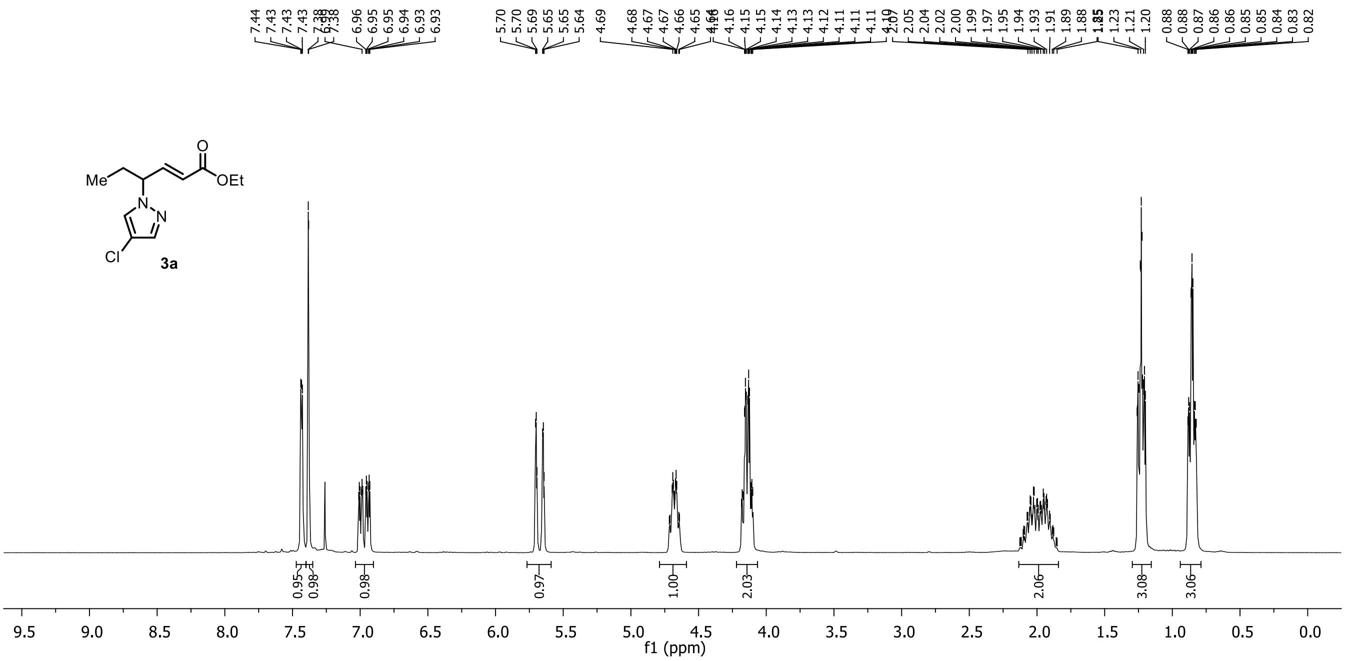

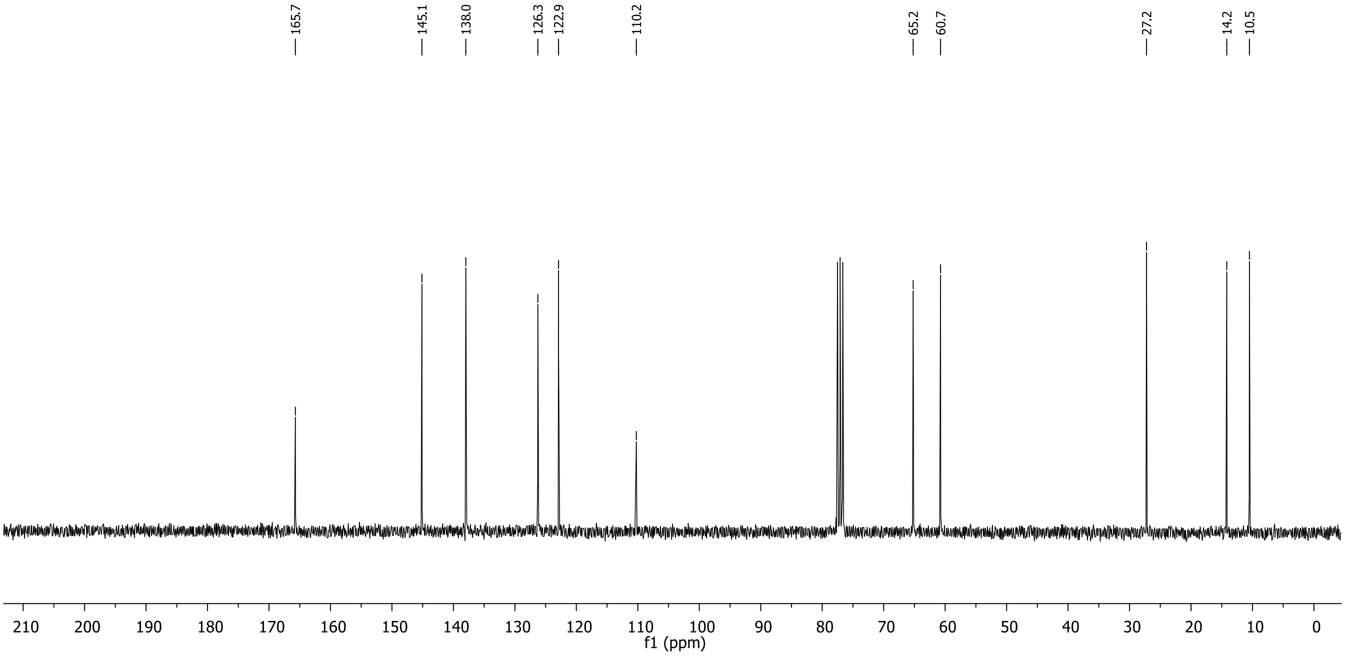

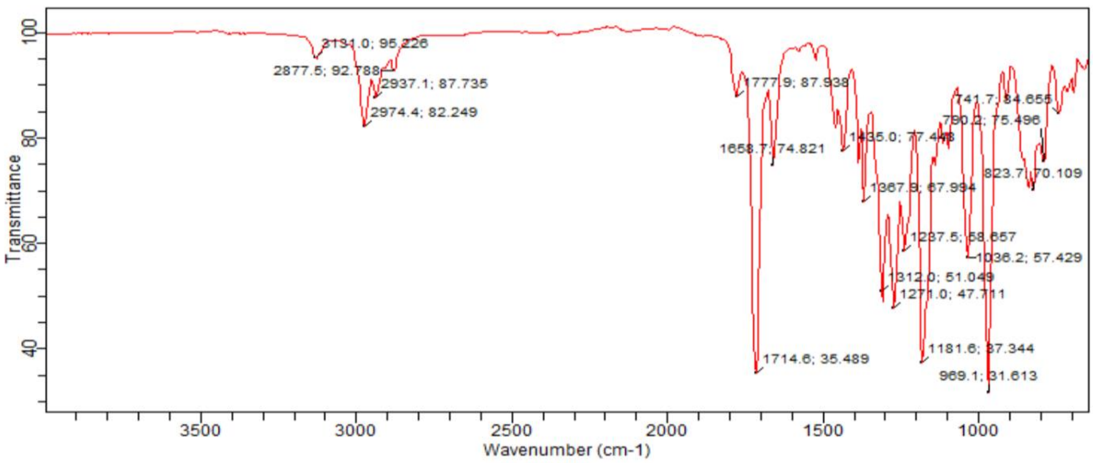

Ethyl (*Z*)-3-(4-chloro-1H-pyrazol-1-yl)hex-3-enoate (3a') (^{1}H NMR: 400 MHz, ^{13}C NMR: 101 MHz, $CDCl_3$):

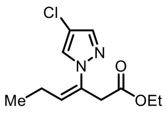

3a'

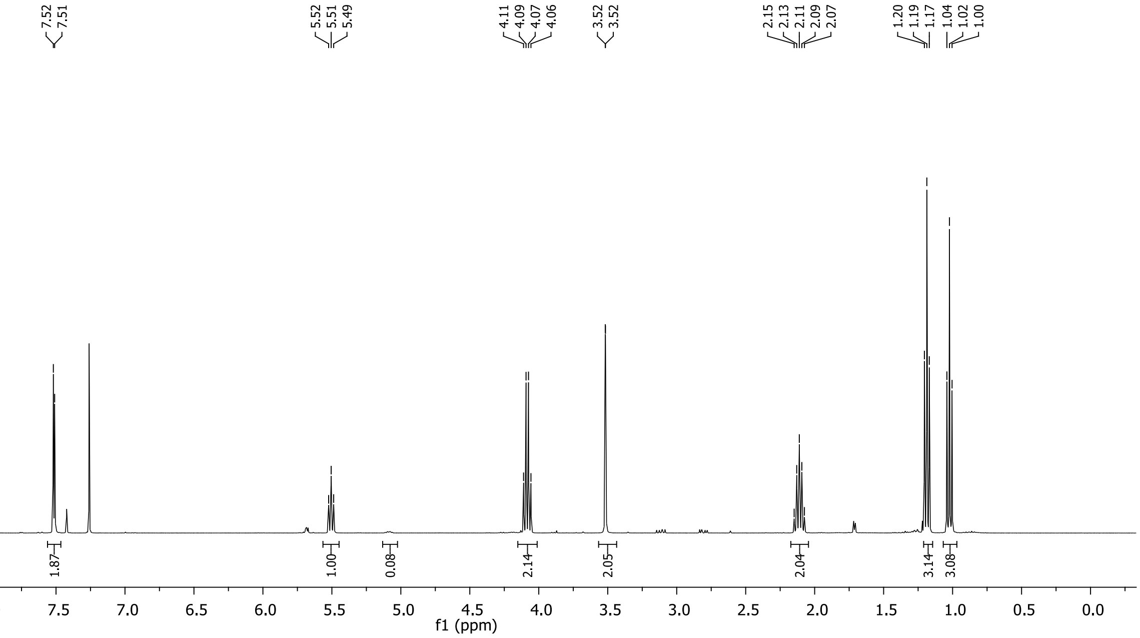

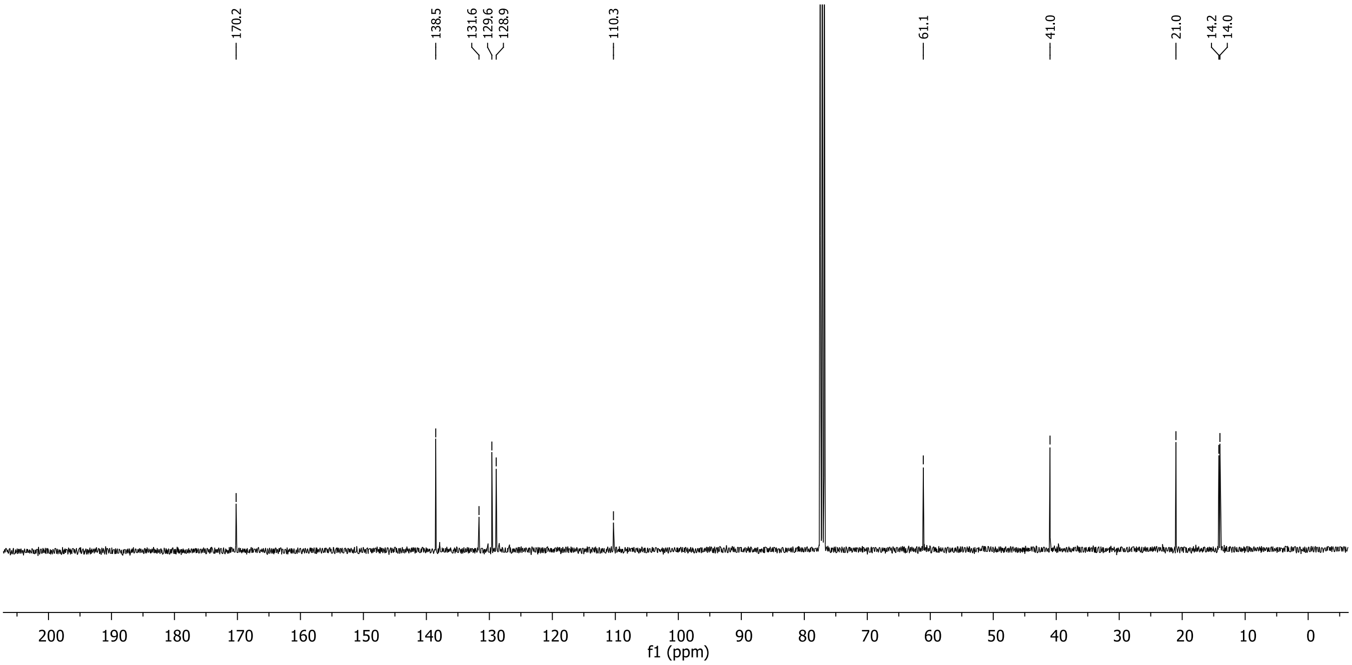

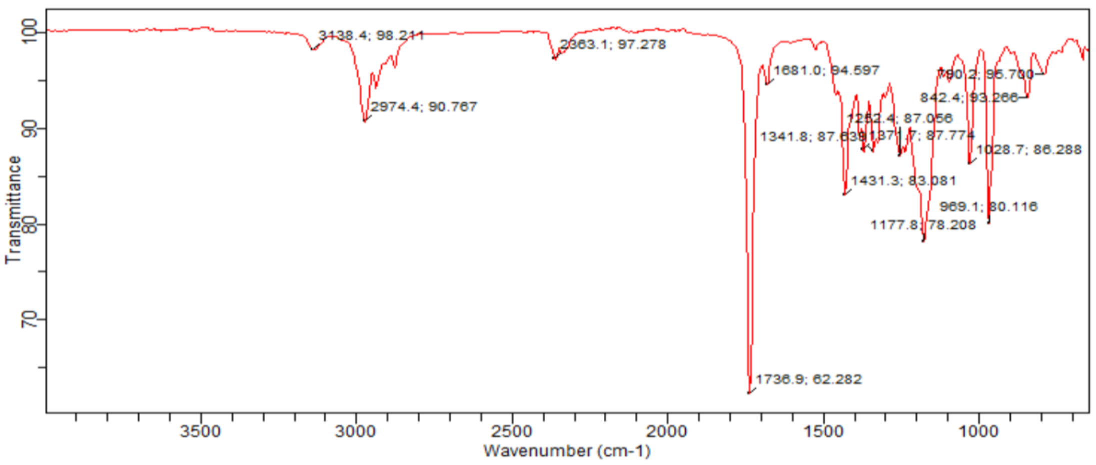

Ethyl (*E*)-4-(4-chloro-1H-pyrazol-1-yl)dec-2-enoate (3b) (^{1}H NMR: 300 MHz, ^{13}C NMR: 75 MHz, CDCl₃):

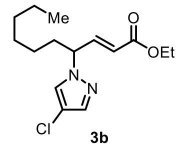

3b

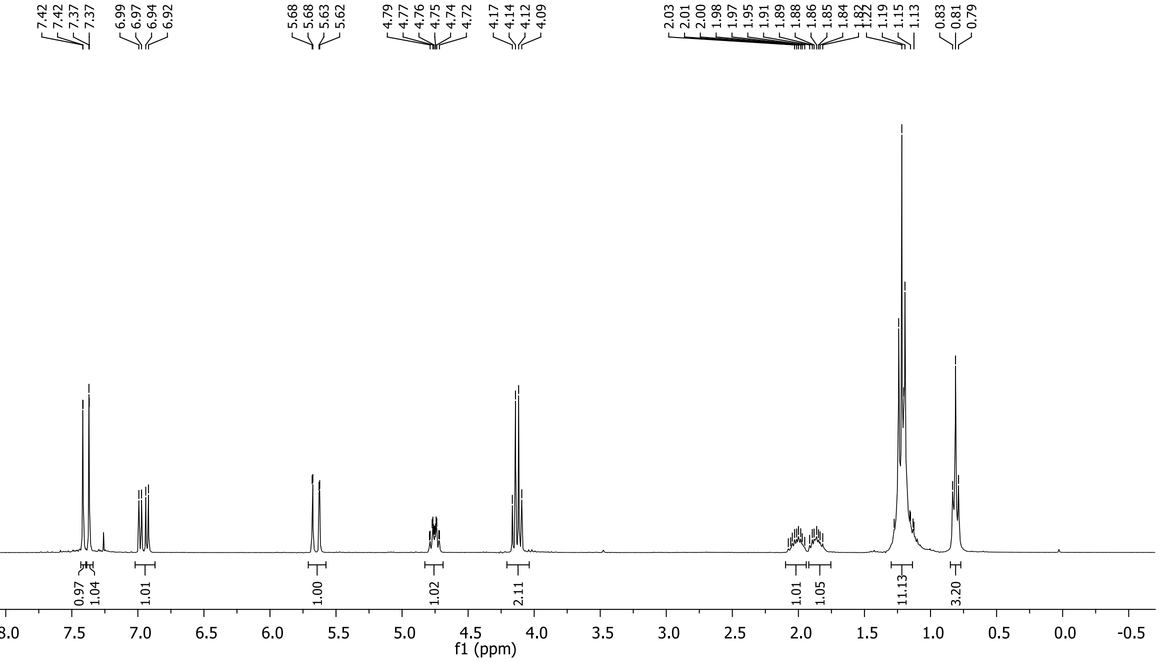

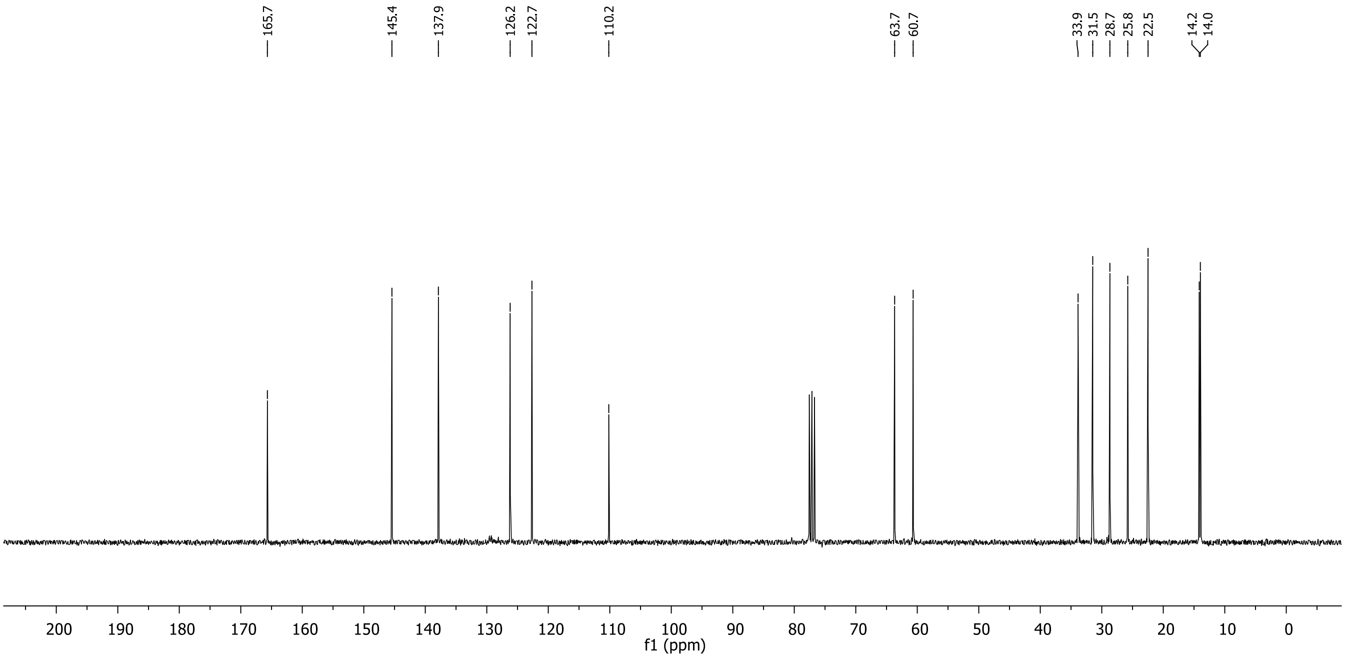

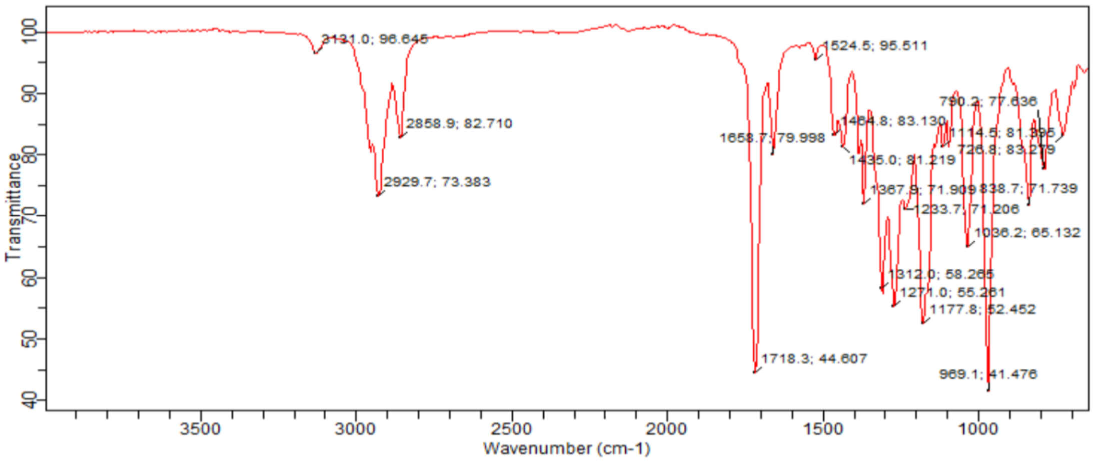

Diethyl (*E*)-4-(4-chloro-1H-pyrazol-1-yl)hex-2-enedioate (3c) (^{1}H NMR: 300 MHz, ^{13}C NMR: 101 MHz, CDCl₃):

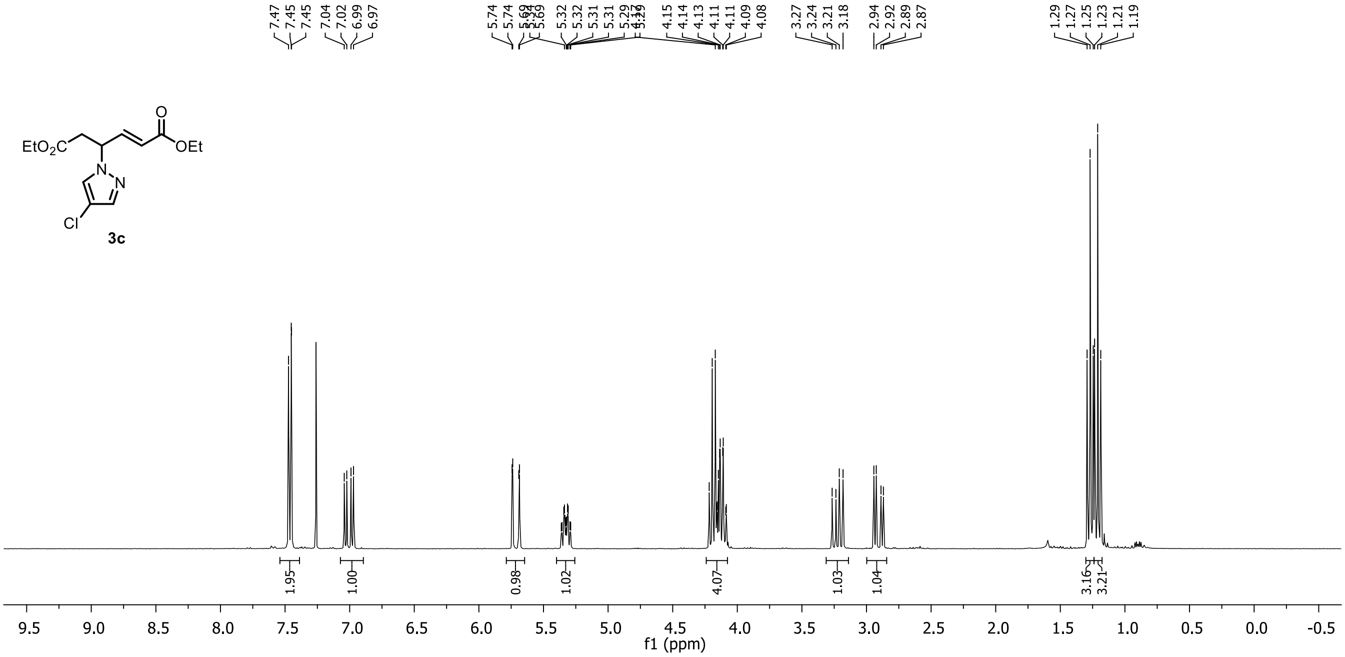

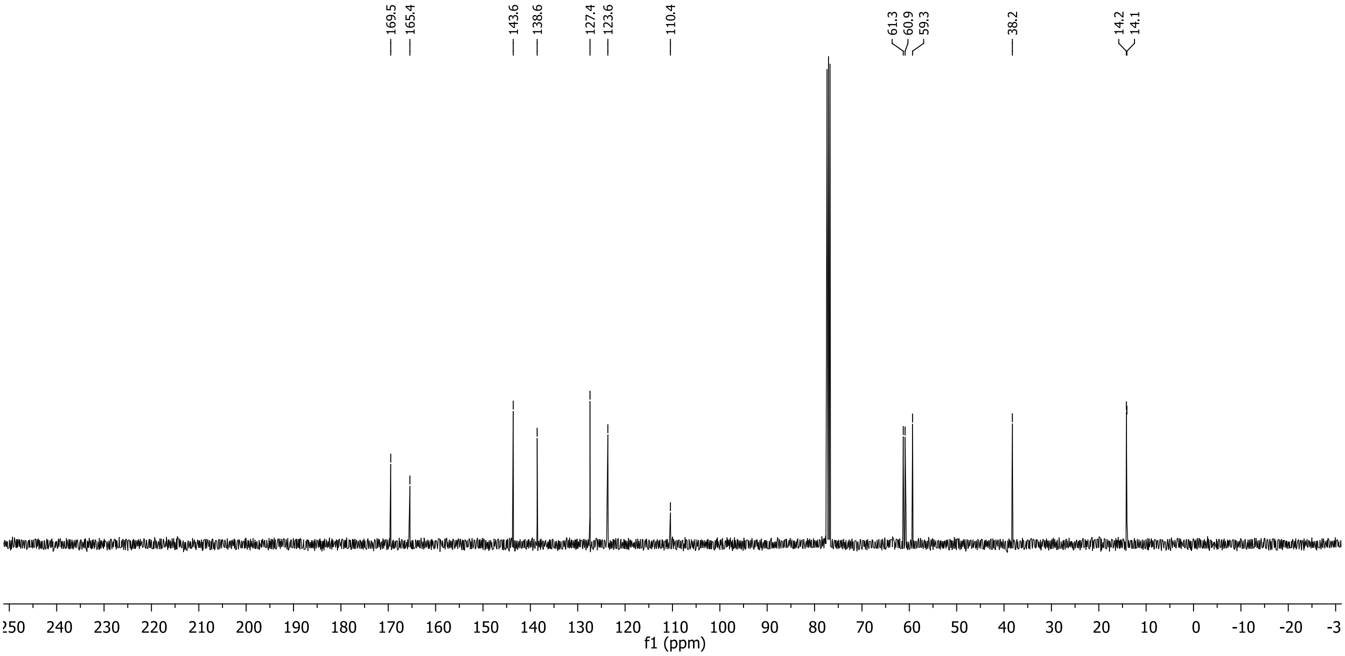

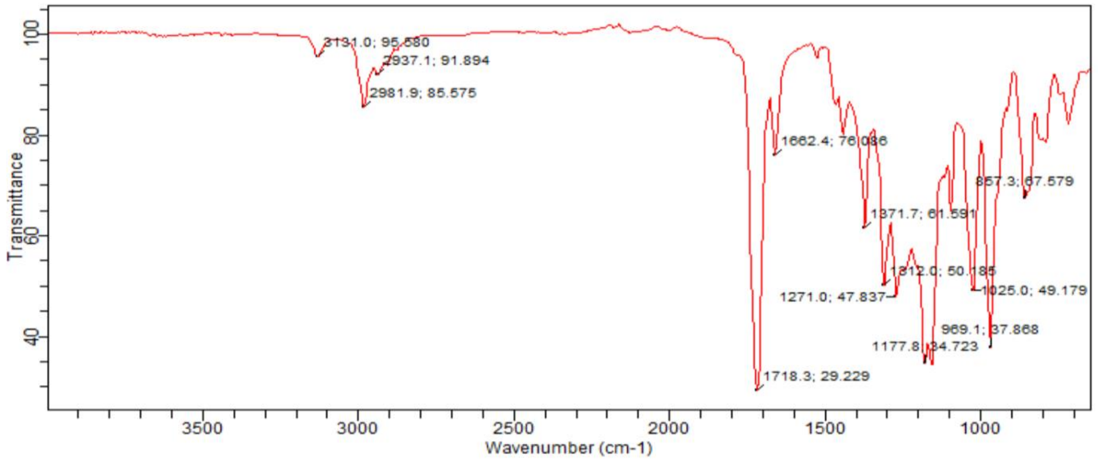

Ethyl (*E*)-8-chloro-4-(4-chloro-1H-pyrazol-1-yl)oct-2-enoate (3d) (^{1}H NMR: 300 MHz, ^{13}C NMR: 75 MHz, $CDCl_3$):

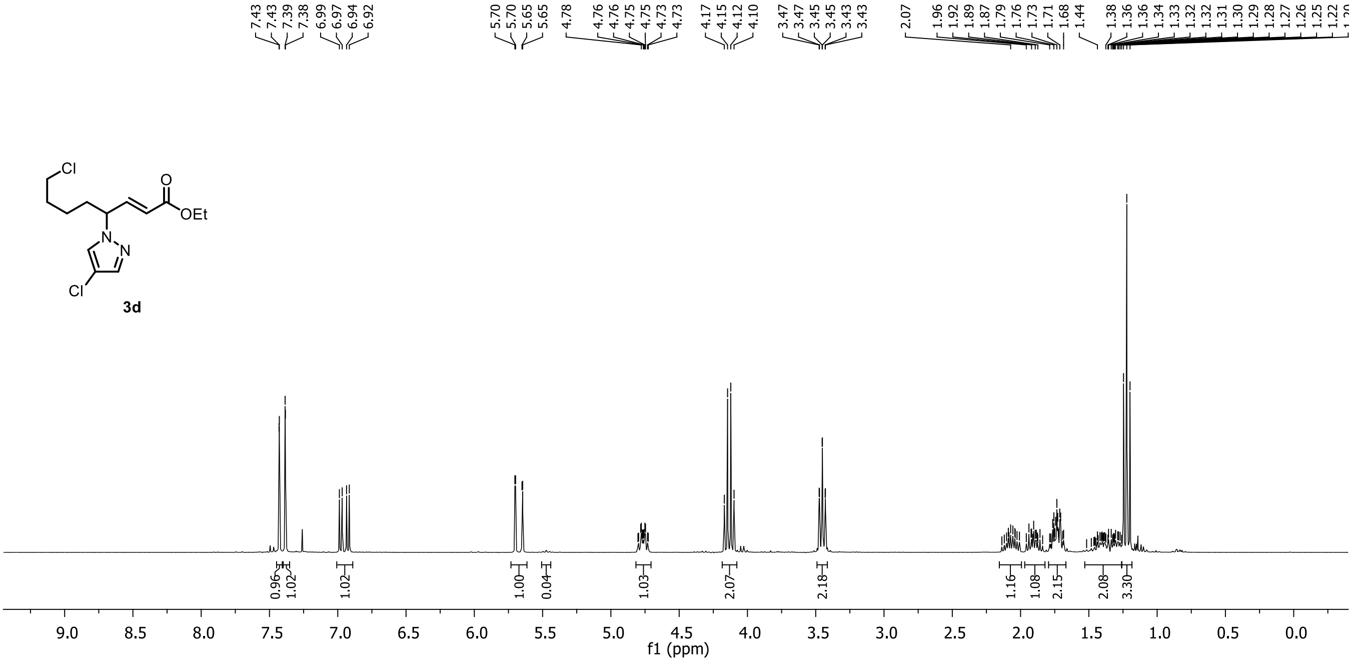

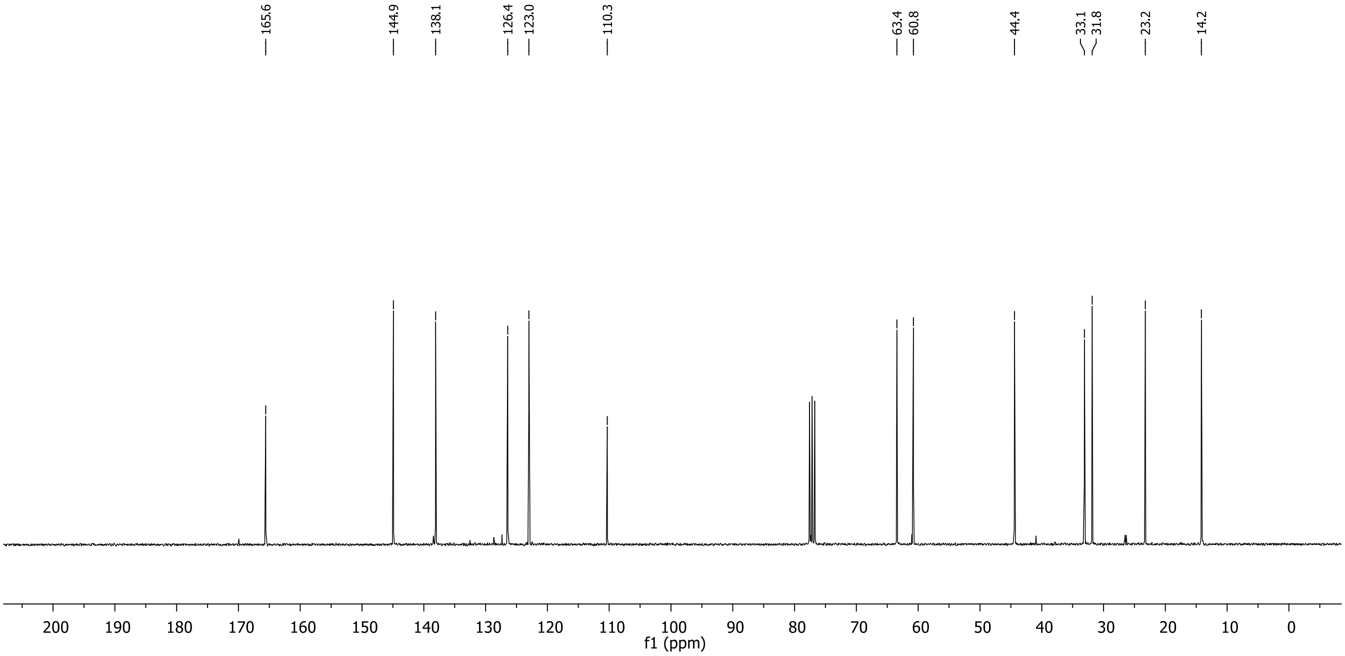

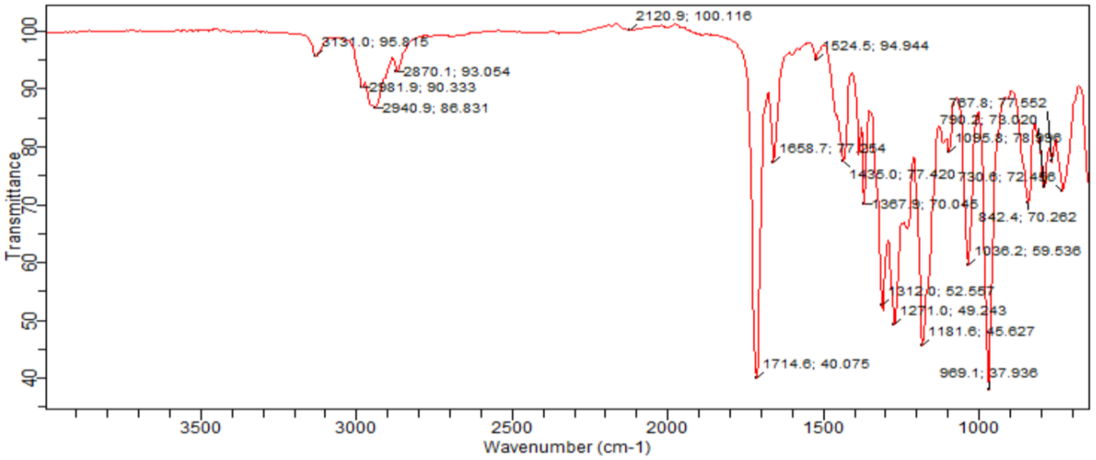

Ethyl (*E*)-4-(4-chloro-1H-pyrazol-1-yl)-8-cyanooct-2-enoate (3e) (^{1}H NMR: 300 MHz, ^{13}C NMR: 75 MHz, CDCl$_3$):

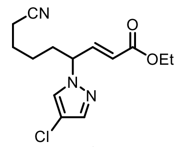

3e

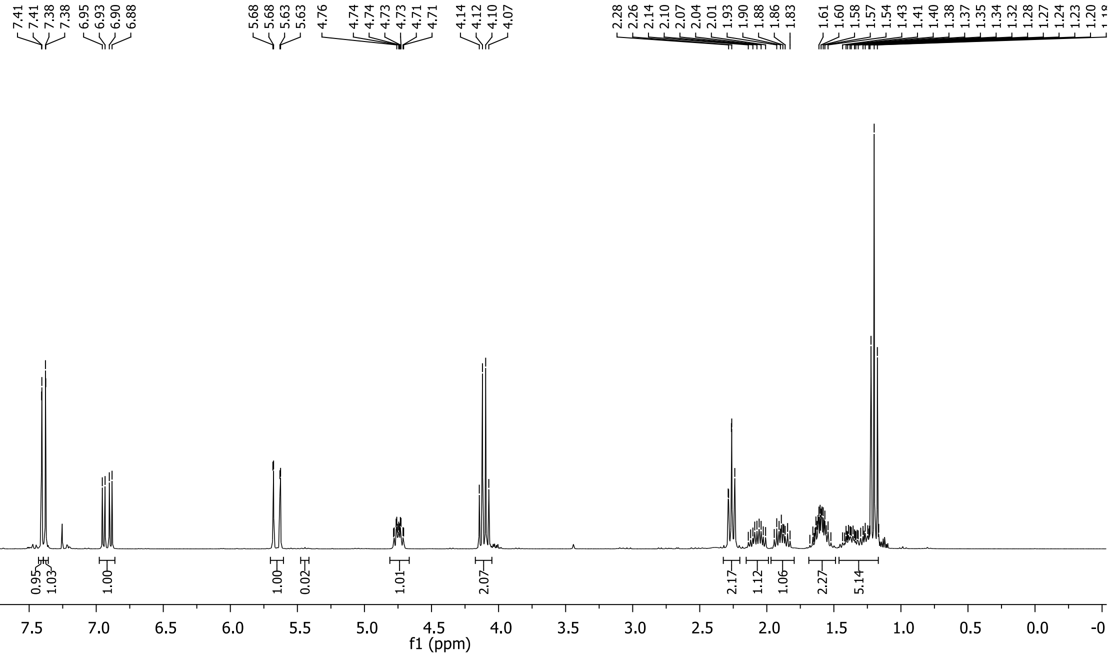

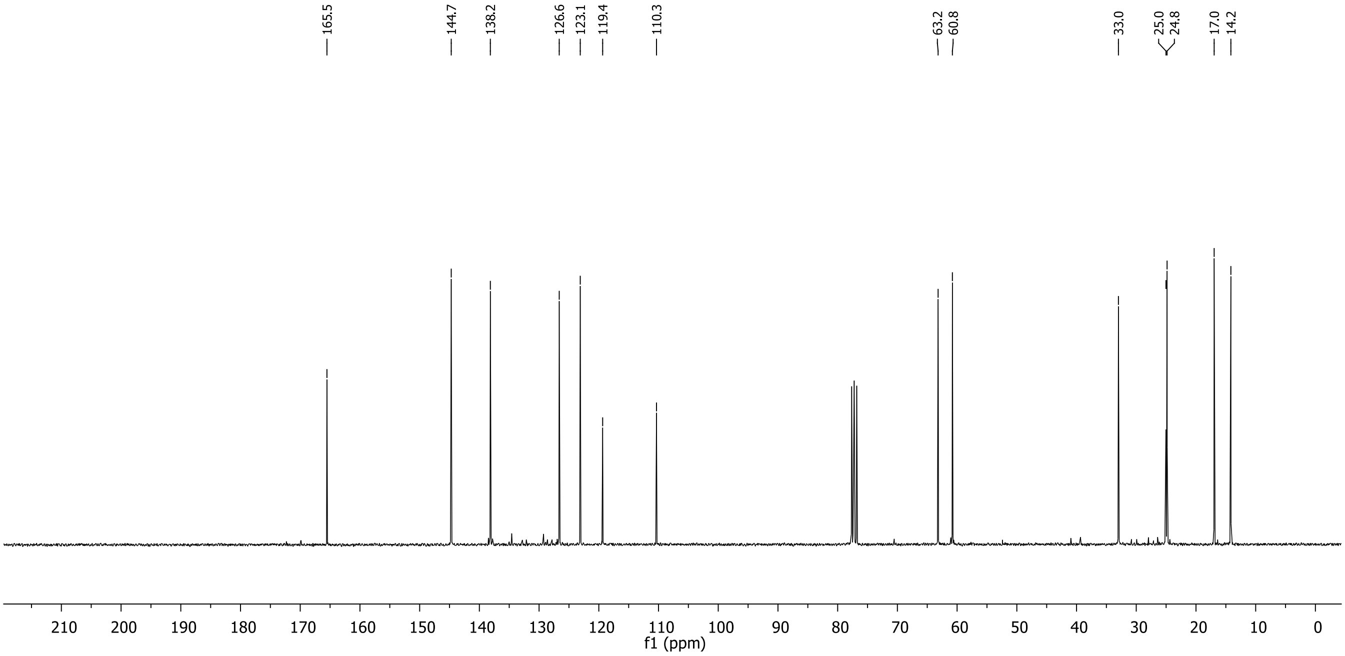

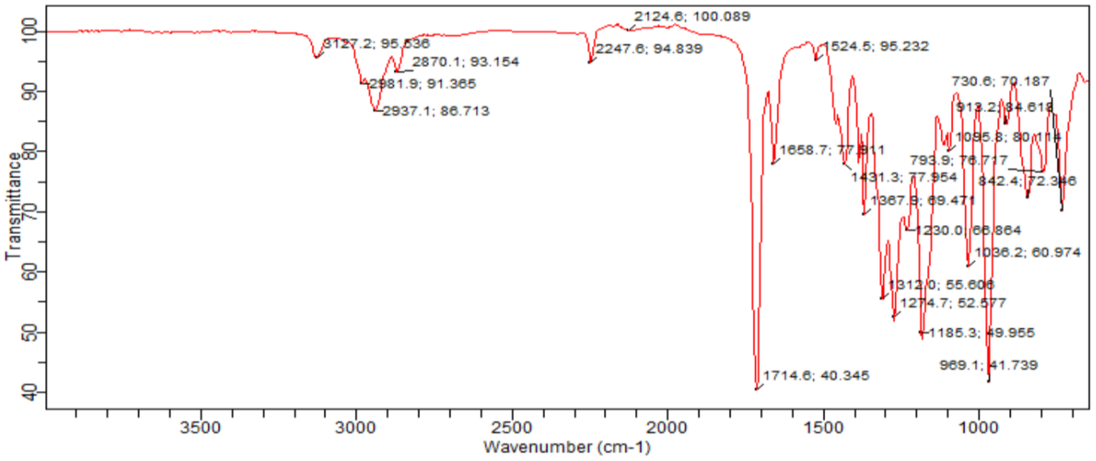

Ethyl (*E*)-4-(4-chloro-1H-pyrazol-1-yl)-7-phenylhept-2-enoate (3f) (^{1}H NMR: 300 MHz, ^{13}C NMR: 75 MHz, $CDCl_3$):

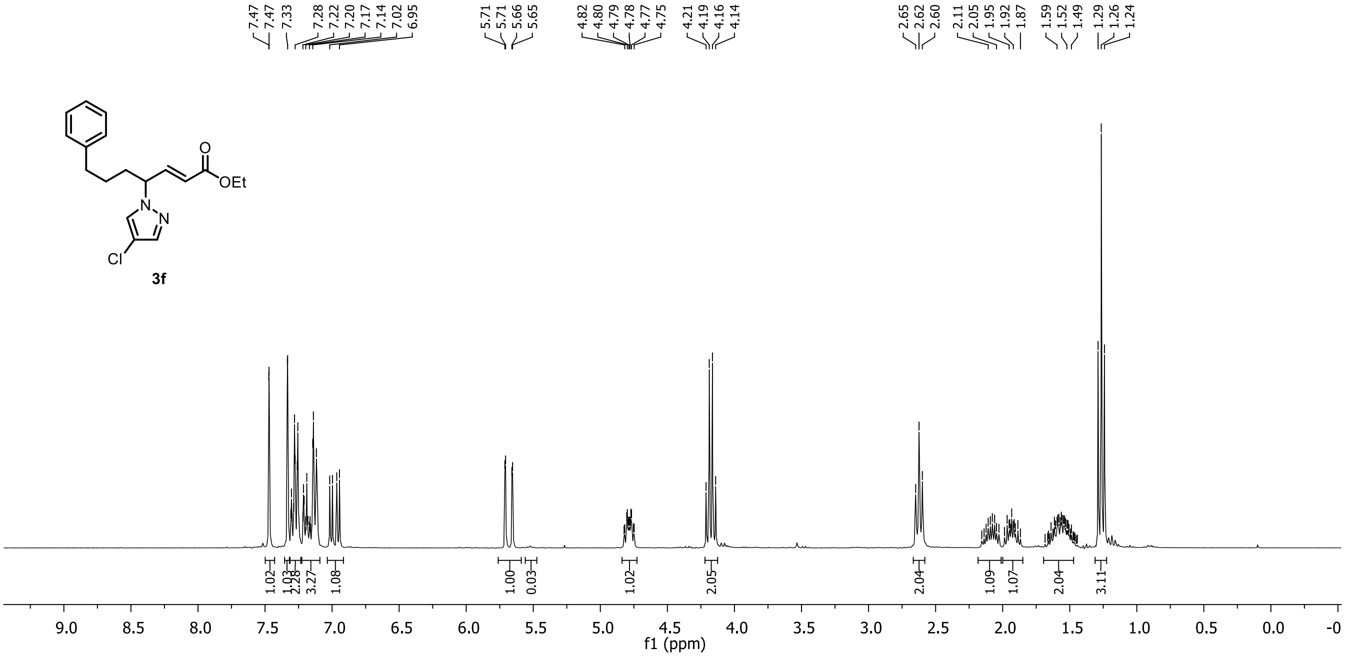

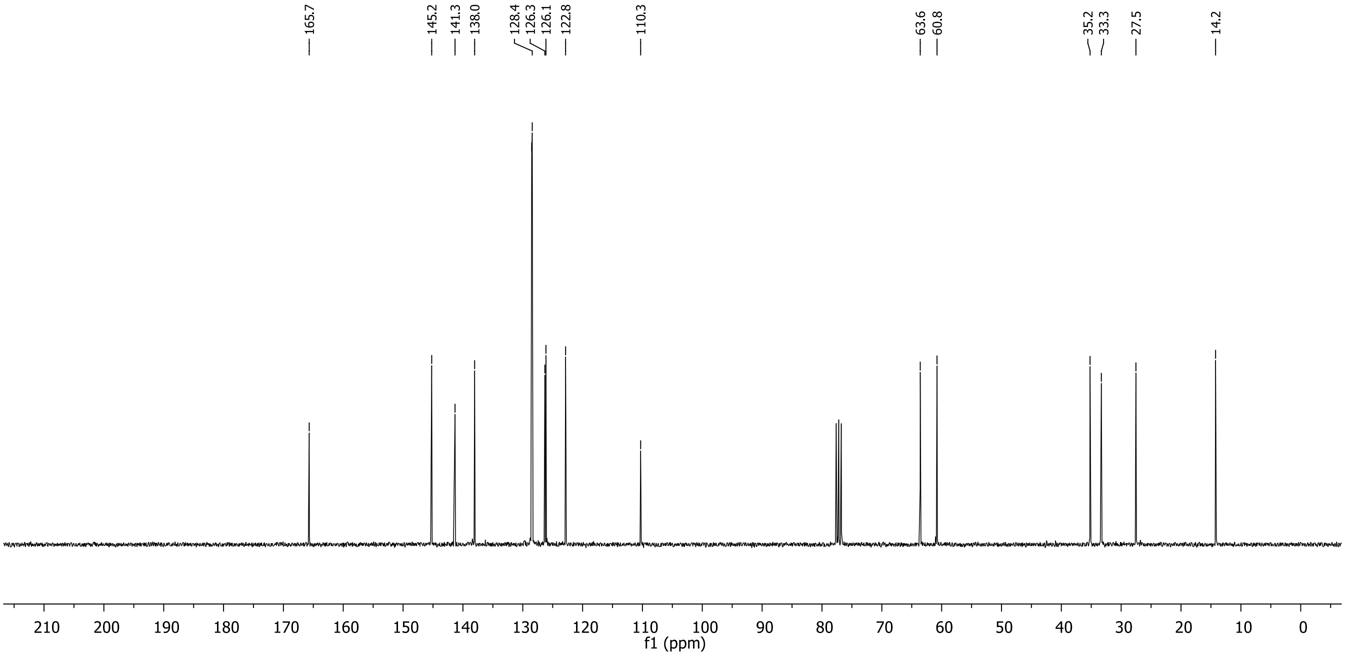

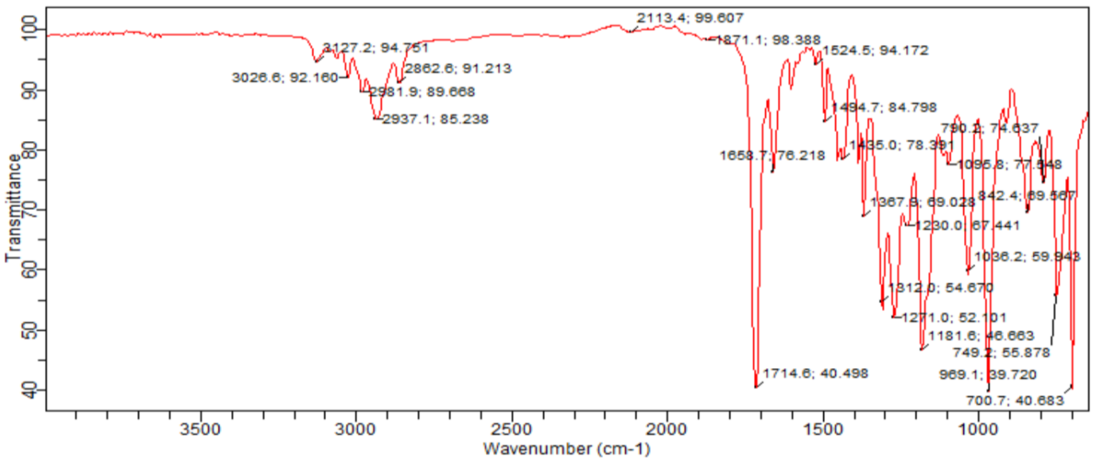

Ethyl (*E*)-7-((*tert*-butyldimethylsilyl)oxy)-4-(4-chloro-1H-pyrazol-1-yl)hept-2-enoate (3g) (^{1}H NMR: 300 MHz, ^{13}C NMR: 75 MHz, $CDCl_3$):

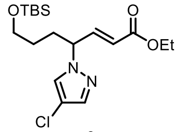

3g

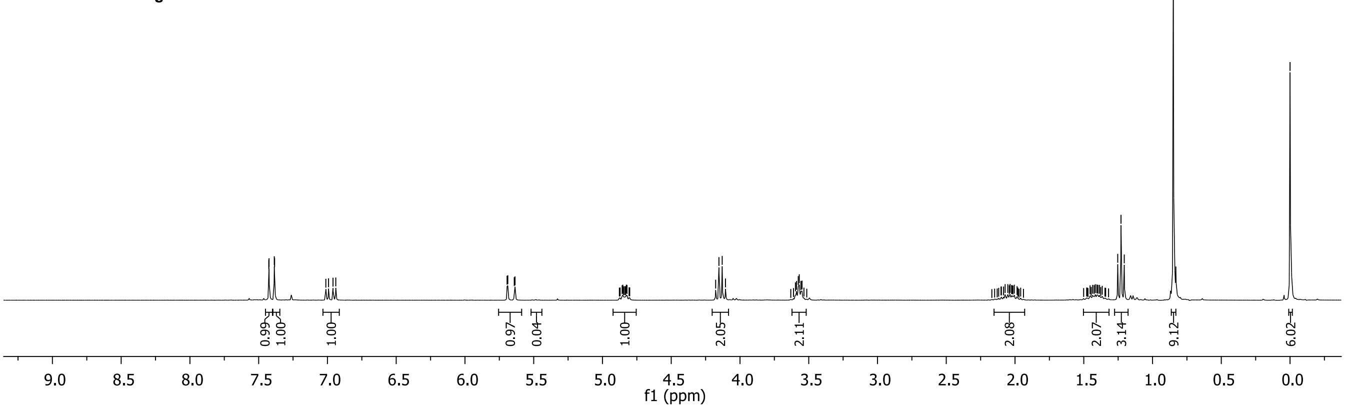

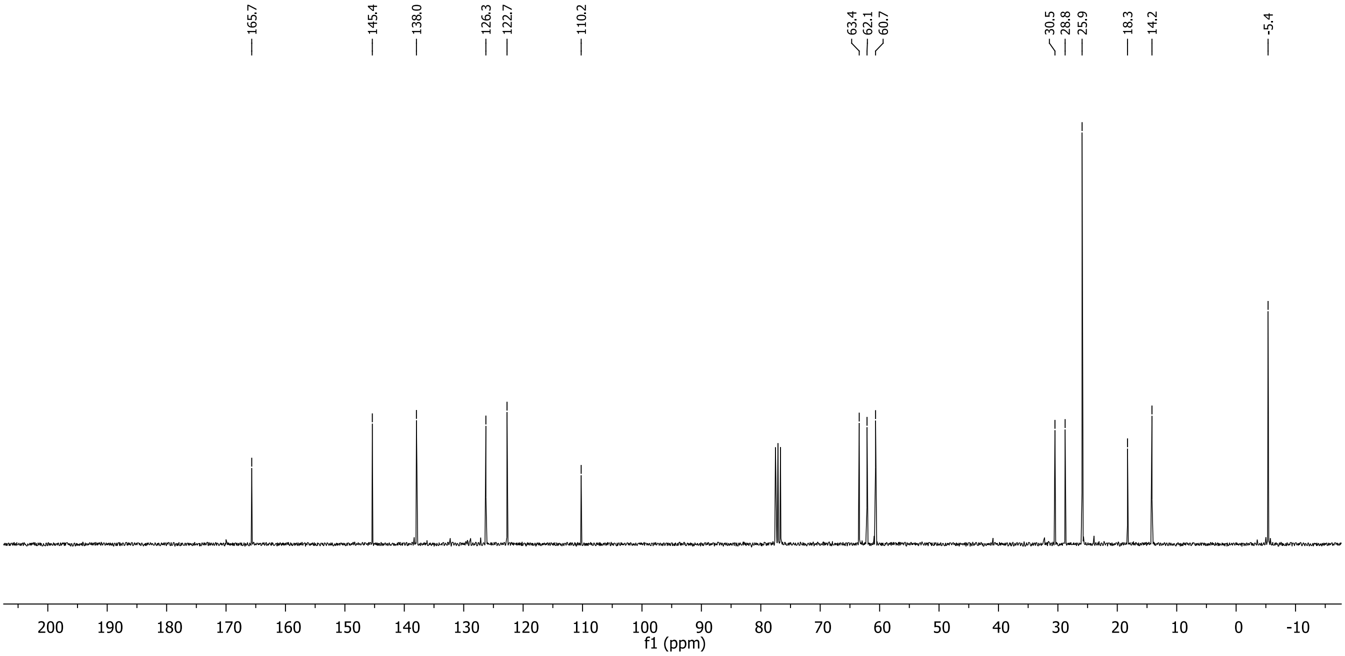

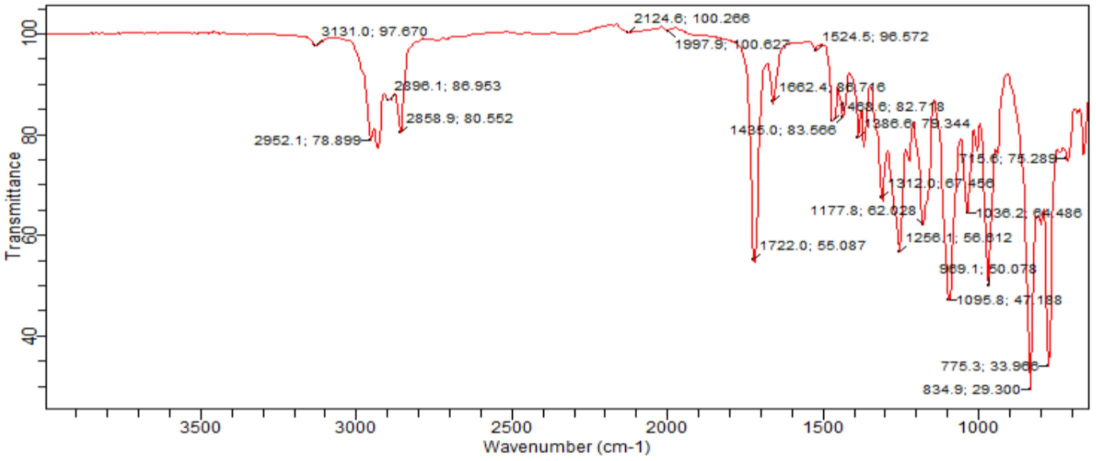

Ethyl (*E*)-4-(4-chloro-1H-pyrazol-1-yl)-7-((tetrahydro-2H-pyran-2-yl)oxy)hept-2-enoate (3h) (^{1}H NMR: 300 MHz, ^{13}C NMR: 75 MHz, $CDCl_3$):

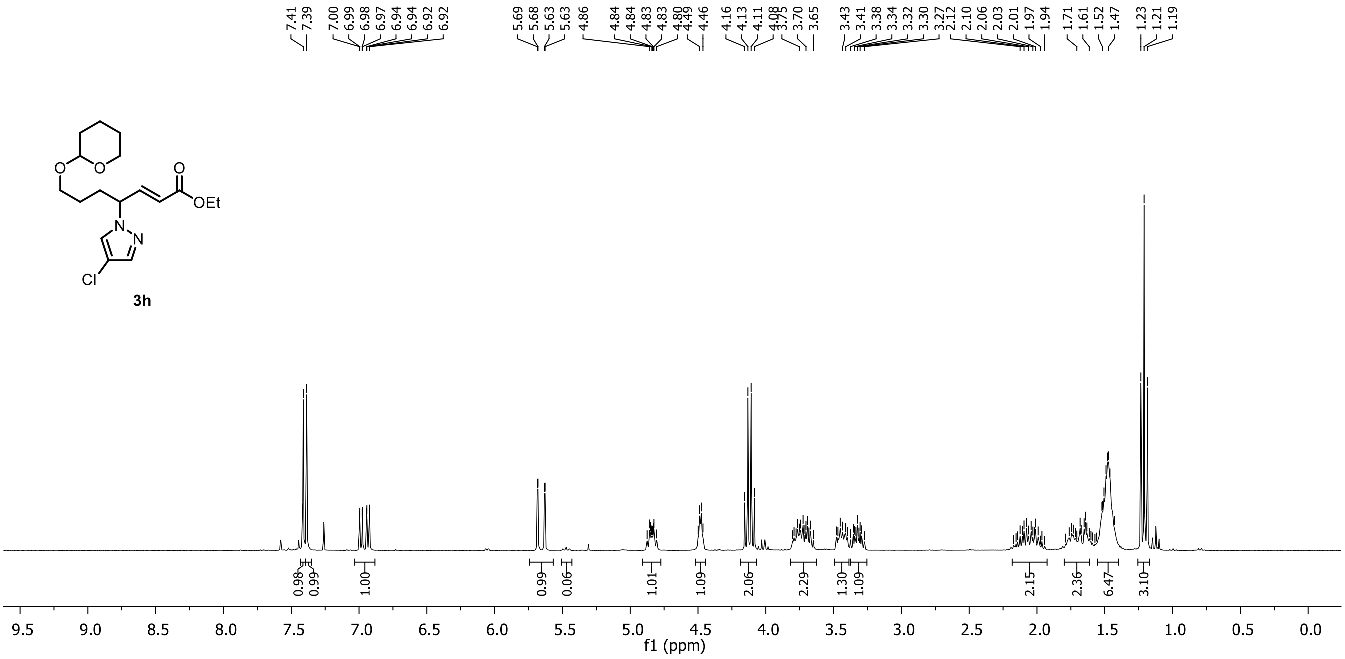

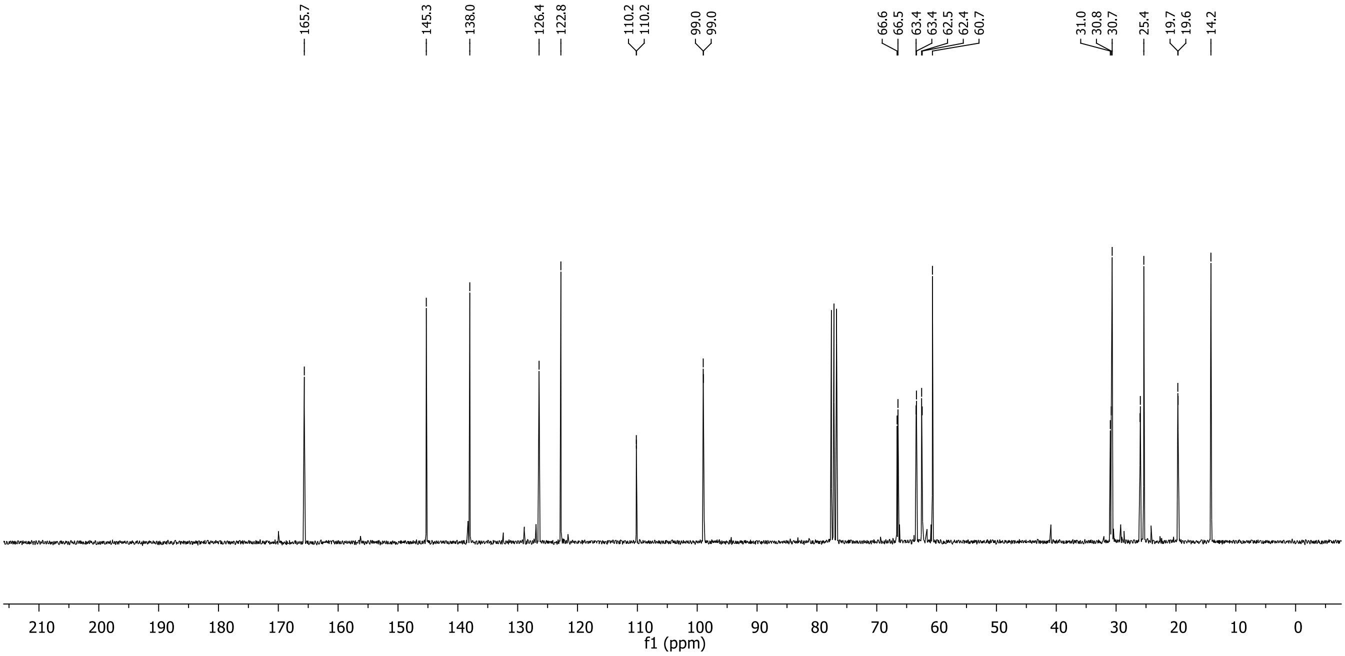

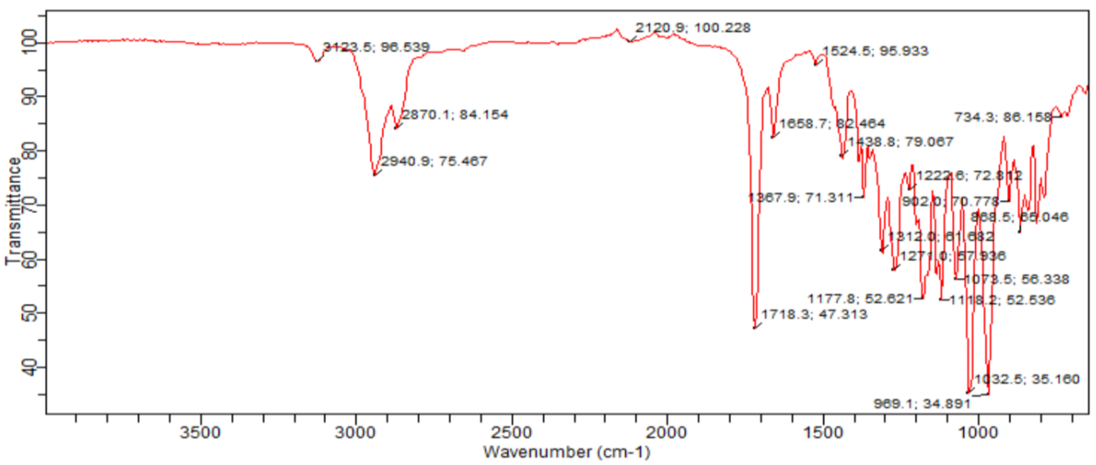

Ethyl (*E*)-6-(benzyloxy)-4-(4-chloro-1H-pyrazol-1-yl)hex-2-enoate (3i) (^{1}H NMR: 300 MHz, ^{13}C NMR: 75 MHz, $CDCl_3$):

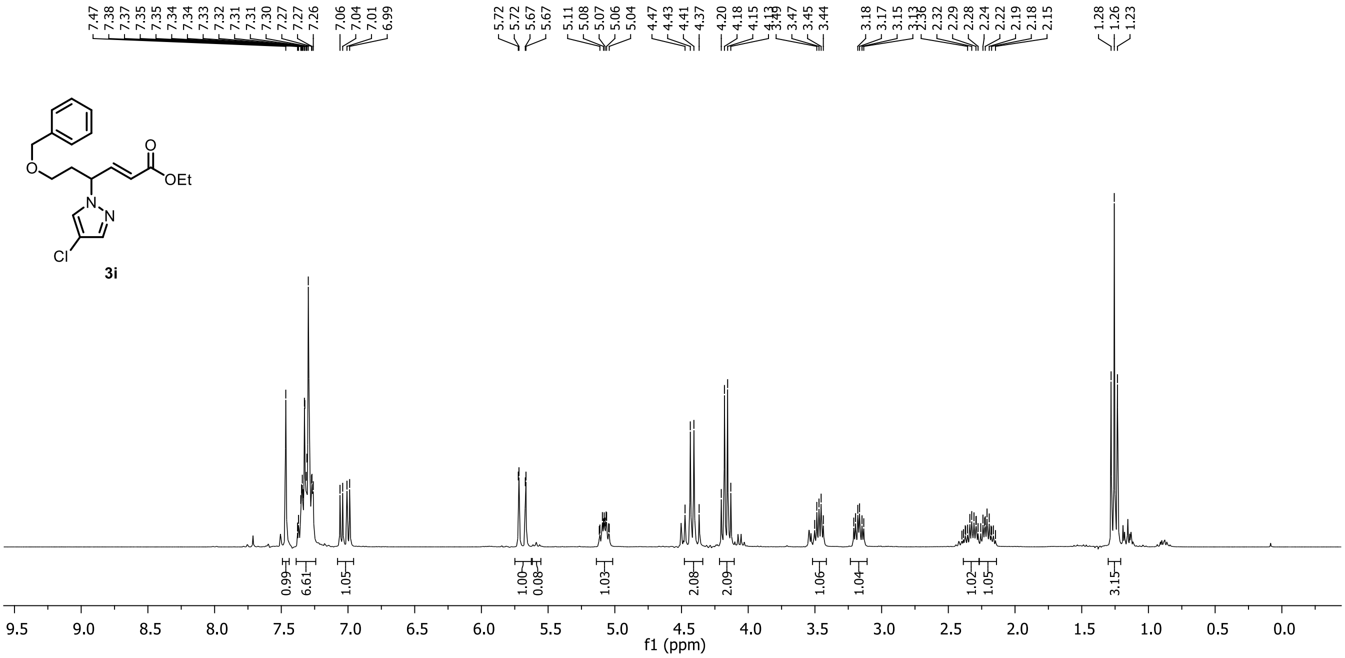

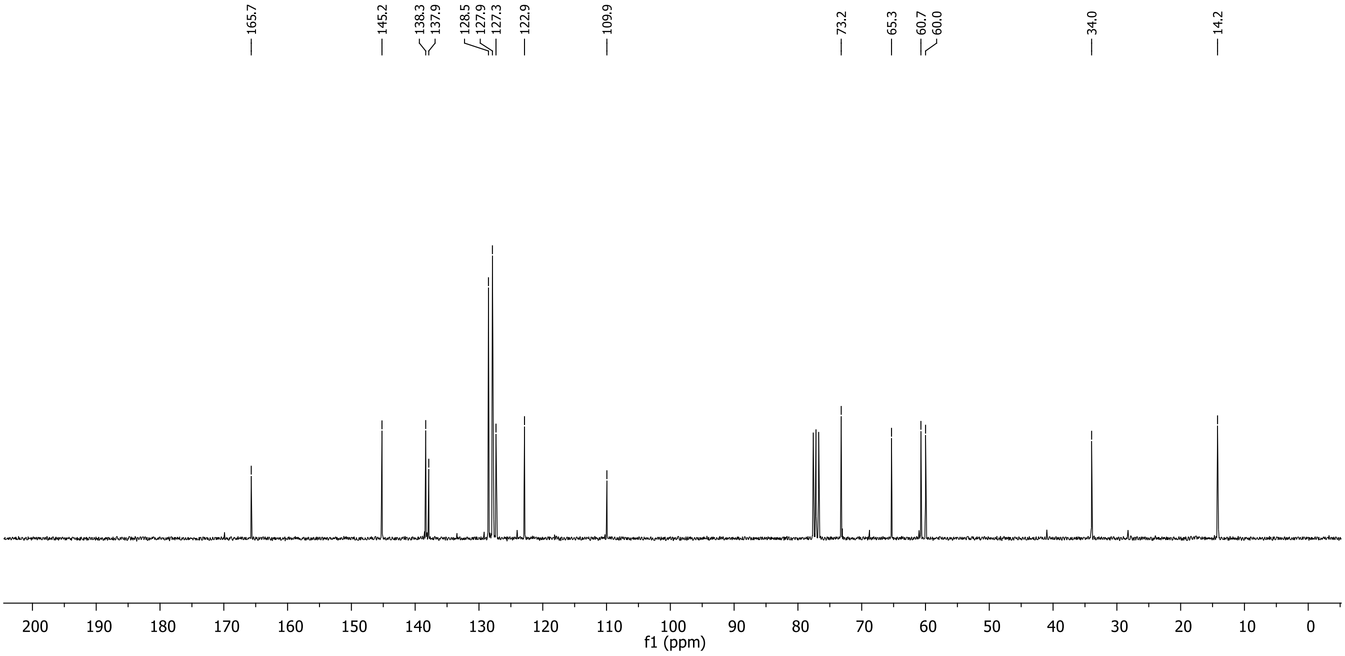

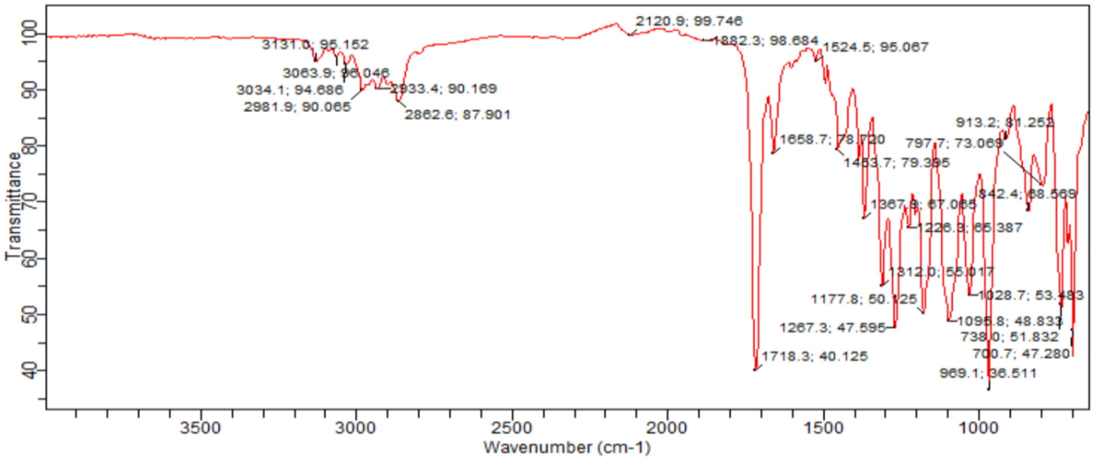

Ethyl (*E*)-4-(4-chloro-1H-pyrazol-1-yl)-5-(4-fluorophenyl)pent-2-enoate (3j) (^{1}H NMR: 400 MHz, ^{13}C NMR: 101 MHz, ^{19}F NMR 377 MHz, $CDCl_3$):

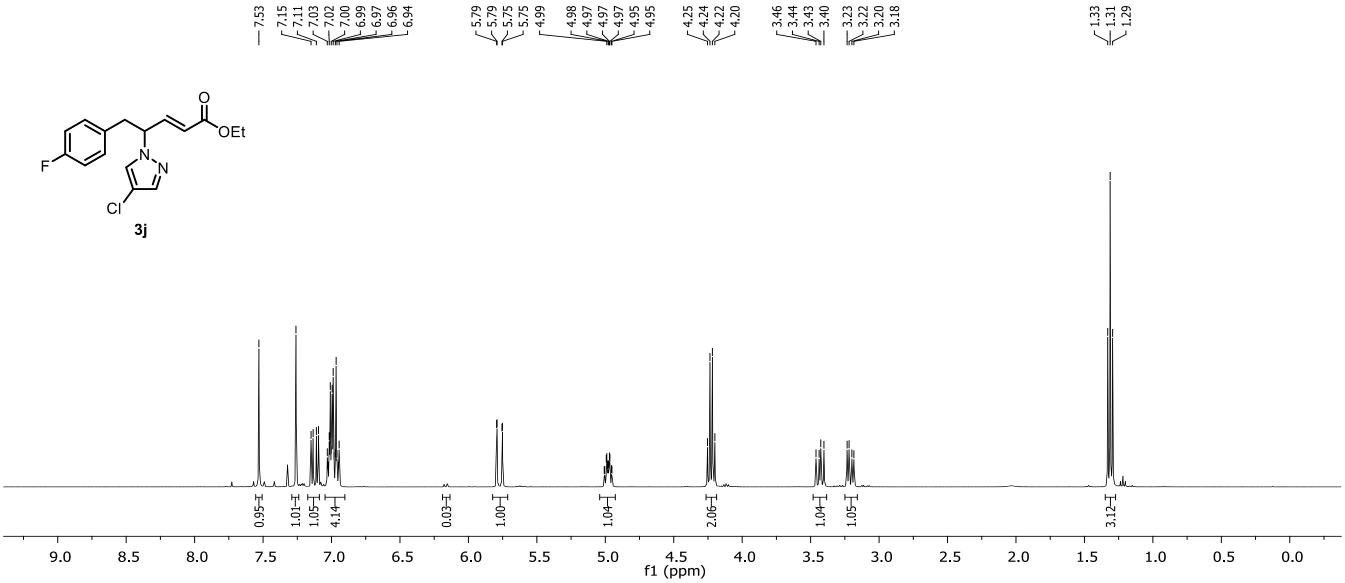

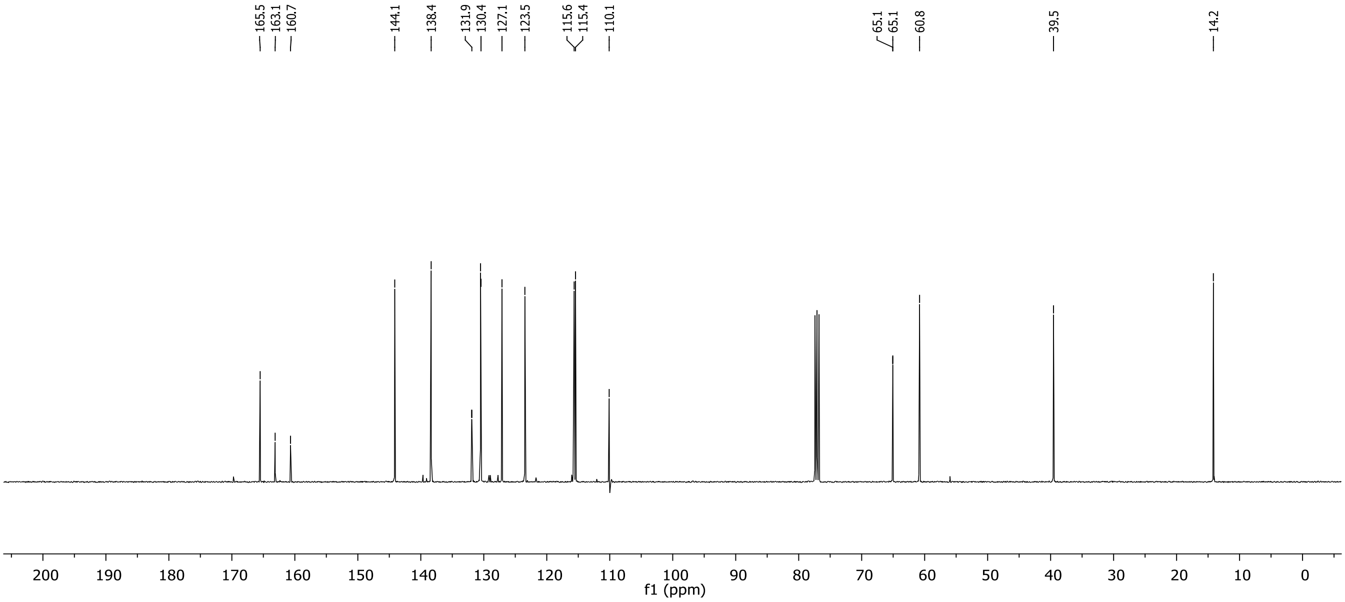

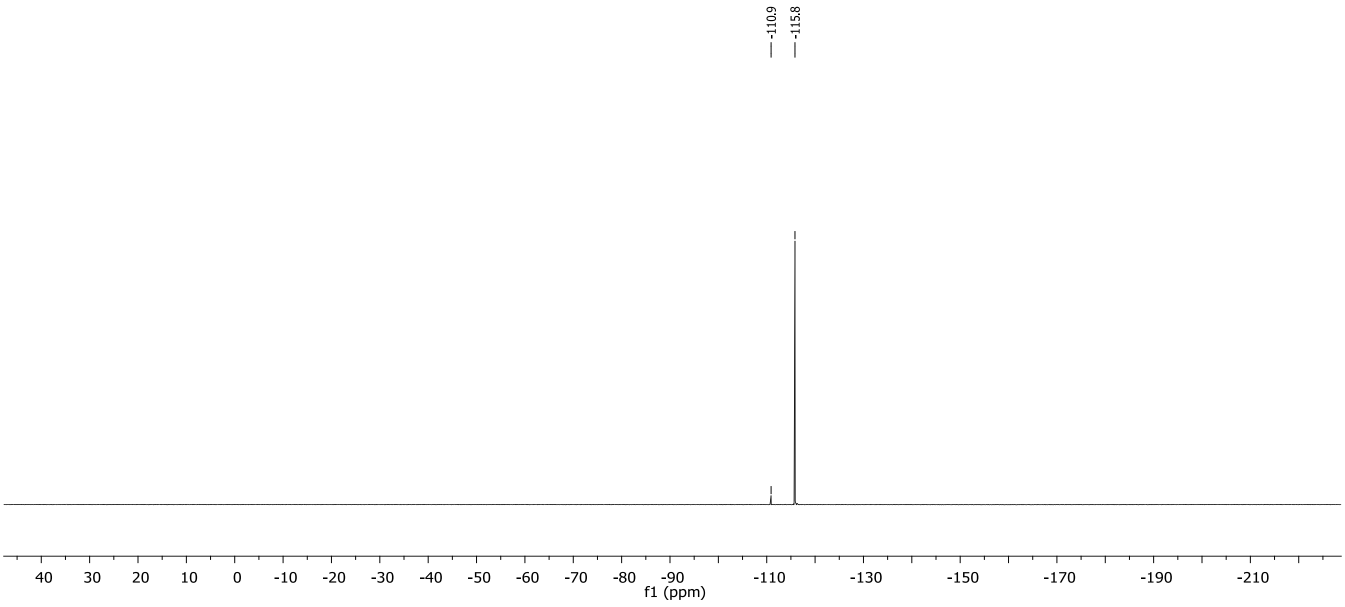

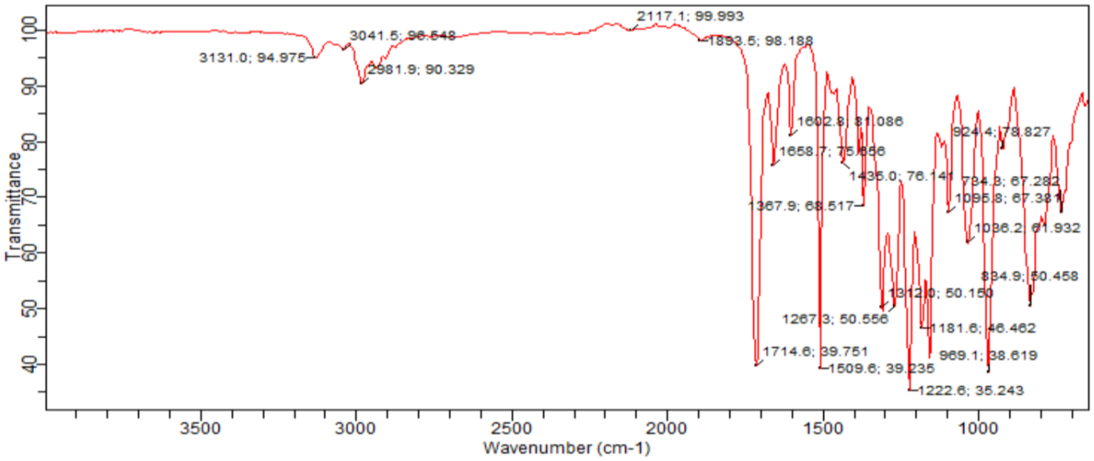

Transmittance
Wavenumber (cm-1)
2117.1; 99.993
3041.5; 96.548
3131.0; 94.975
1893.5; 98.188
2981.9; 90.329
1602.8; 81.086
1658.7; 75.656
1435.0; 76.141
1367.9; 68.517
924.4; 78.827
784.3; 67.282
1095.8; 67.381
1036.2; 61.932
834.9; 50.458
1312.0; 50.150
1267.3; 50.556
1181.6; 46.462
1714.6; 39.751
969.1; 38.619
1509.6; 39.235
1222.6; 35.243

1-Ethyl 8-methyl (*E*)-4-(4-chloro-1H-pyrazol-1-yl)oct-2-enedioate (3k) (^{1}H NMR: 300 MHz, ^{13}C NMR: 75 MHz, $CDCl_3$):

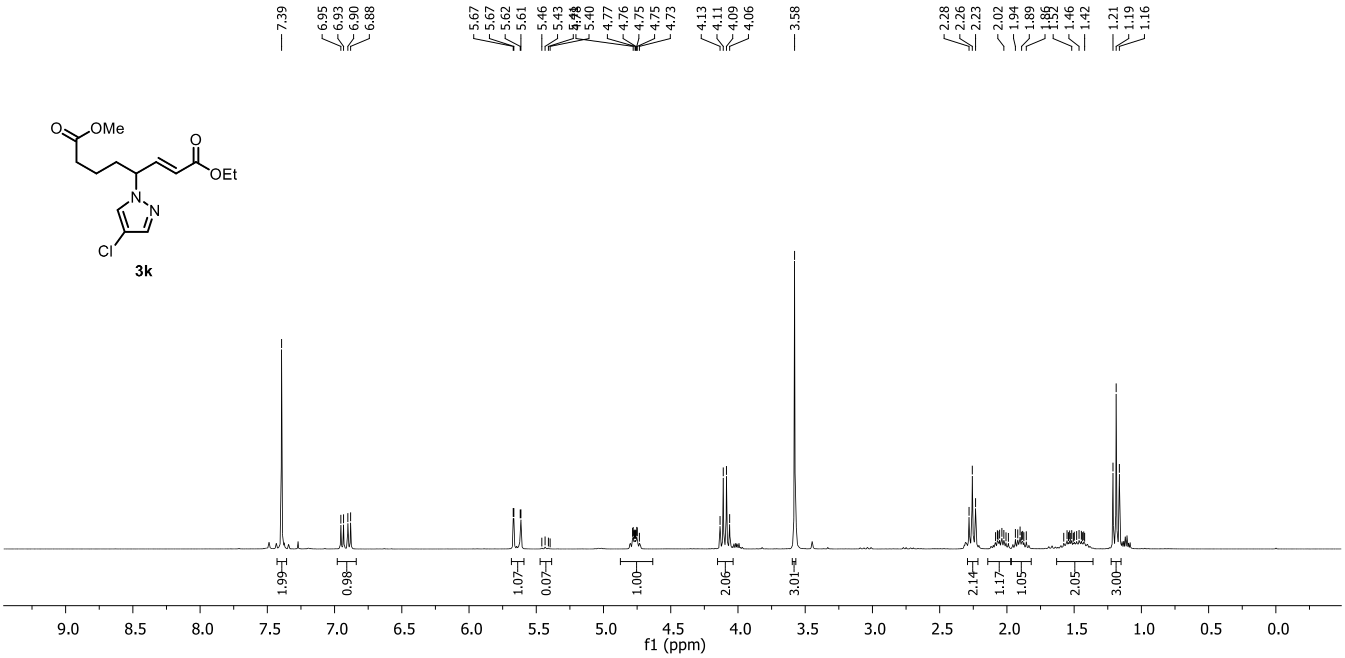

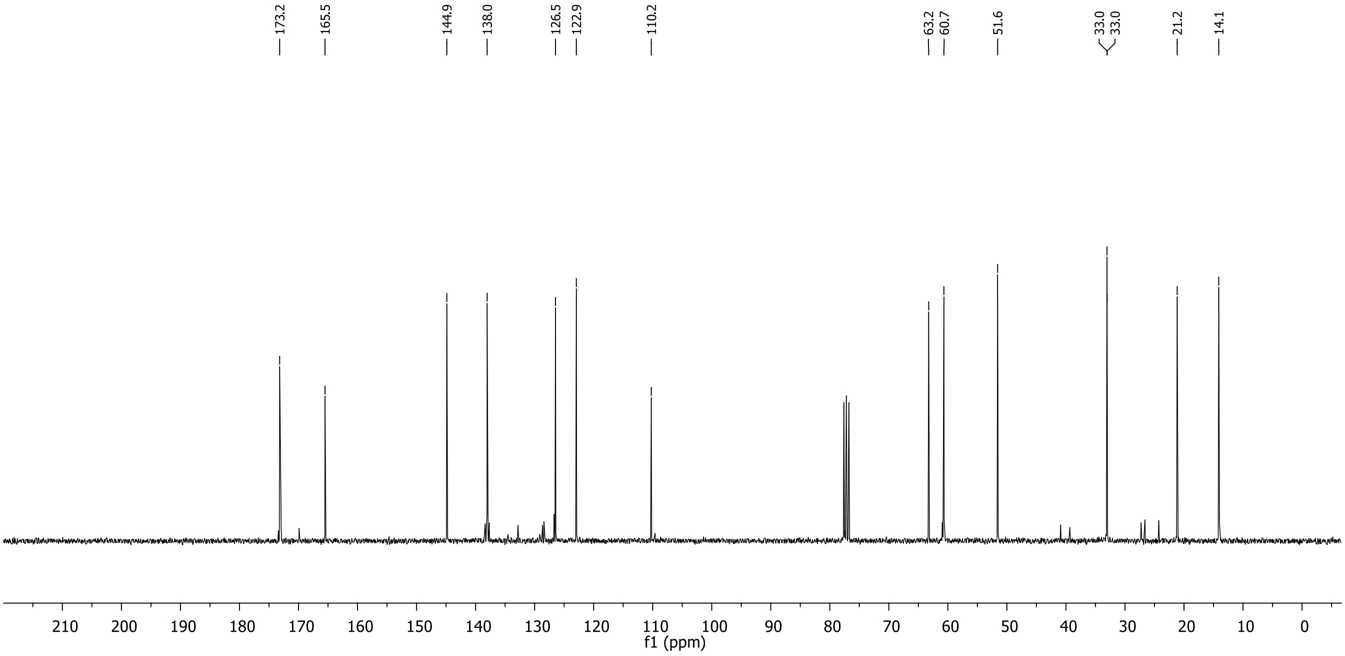

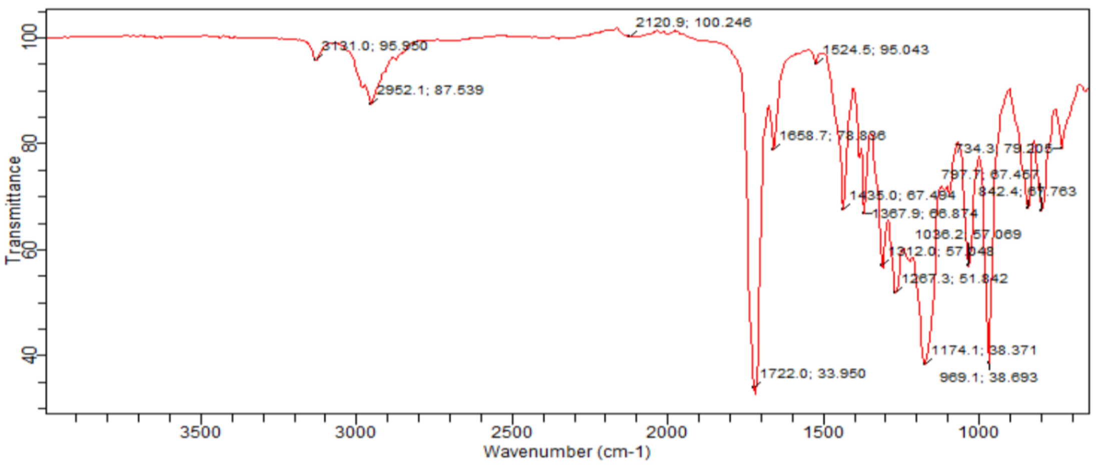

Dimethyl (*E*)-4-(4-chloro-1H-pyrazol-1-yl)hex-2-enedioate (3l) (^{1}H NMR: 300 MHz, ^{13}C NMR: 75 MHz, $CDCl_3$):

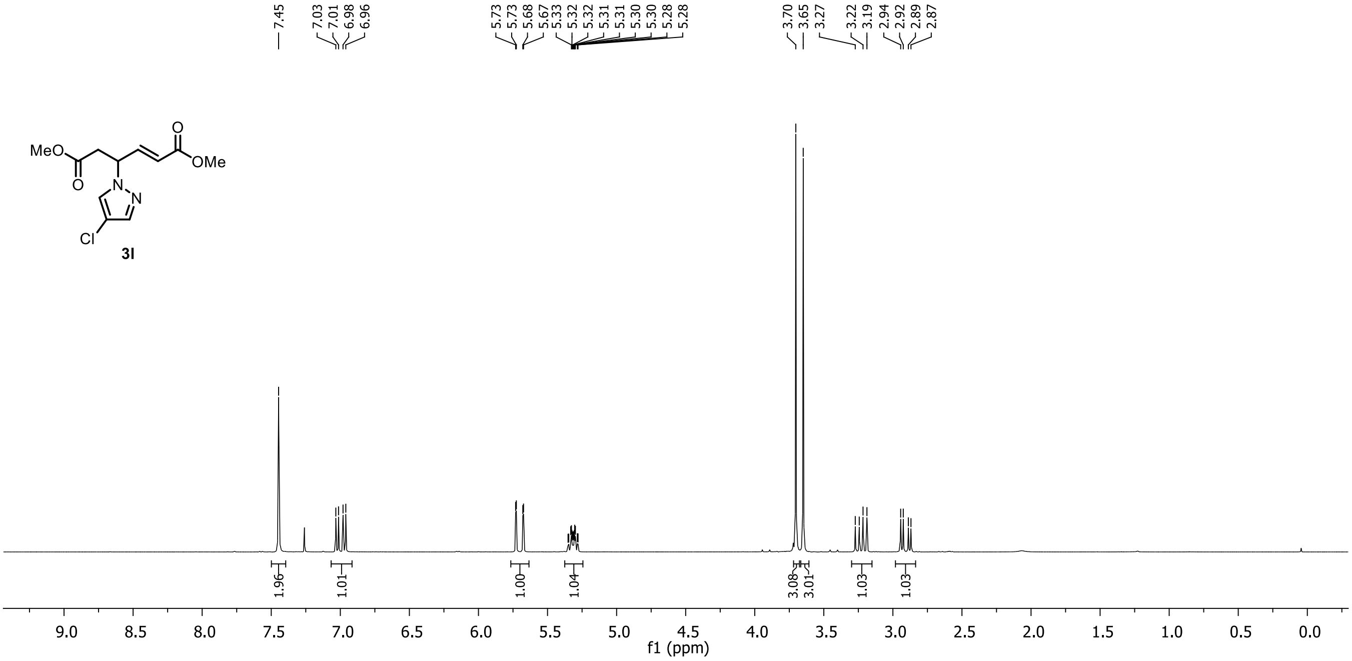

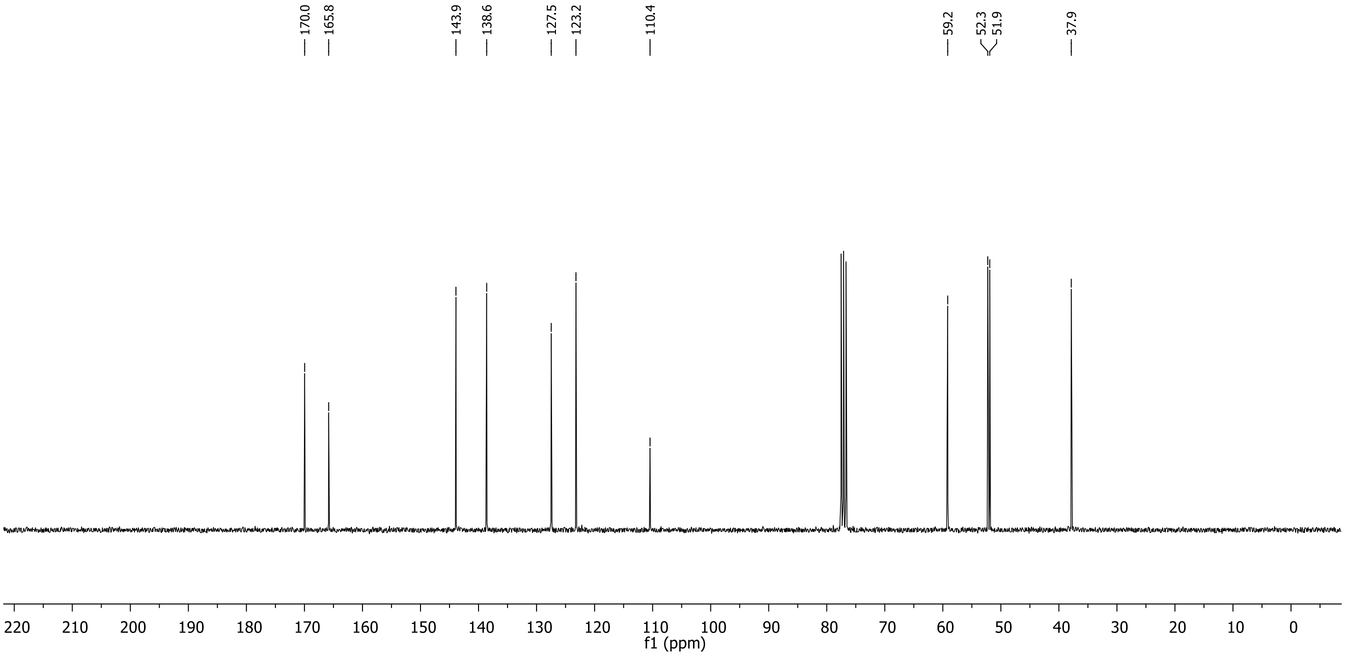

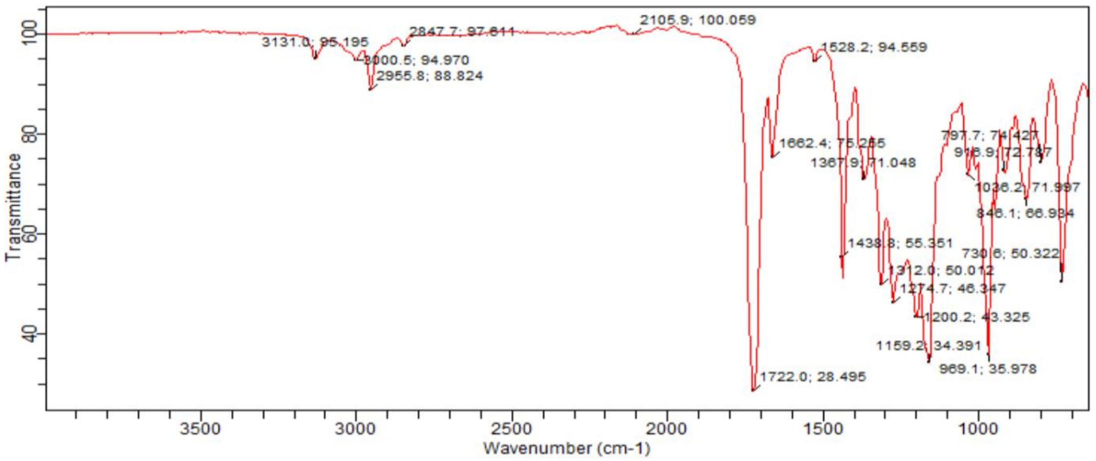

Diisopropyl (*E*)-4-(4-chloro-1H-pyrazol-1-yl)hex-2-enedioate (3m) (^{1}H NMR: 300 MHz, ^{13}C NMR: 75 MHz, $CDCl_3$):

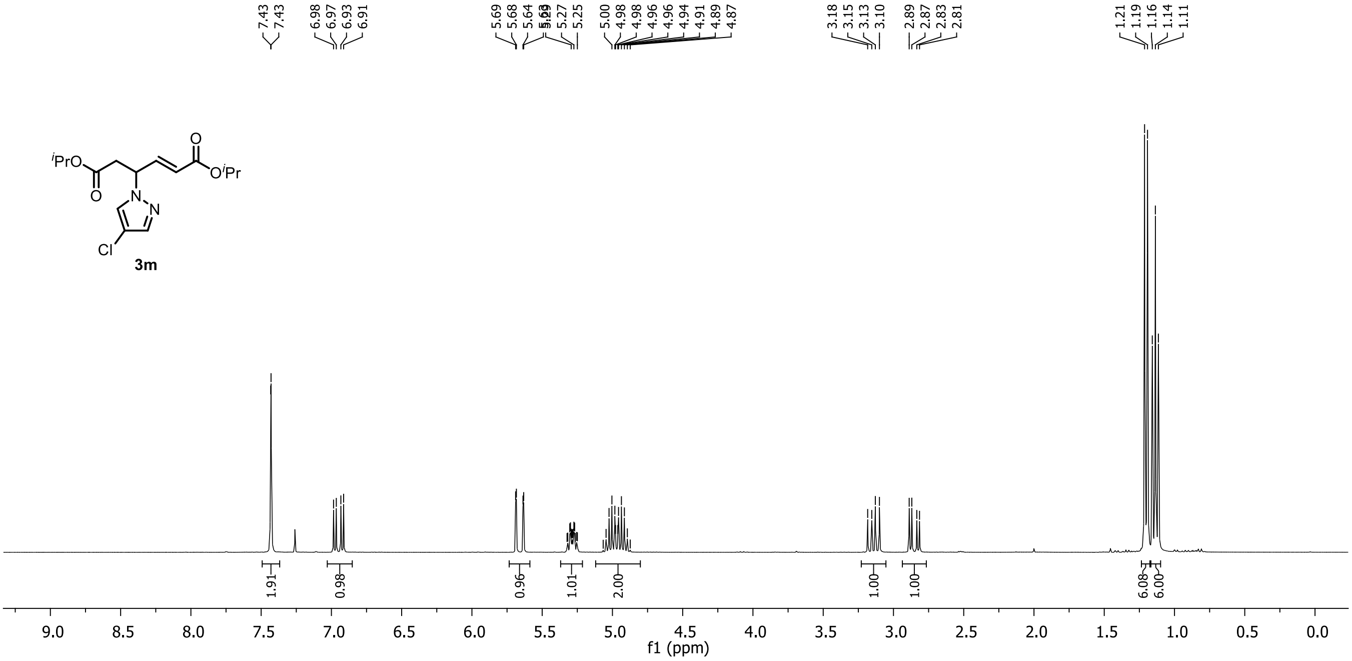

3m

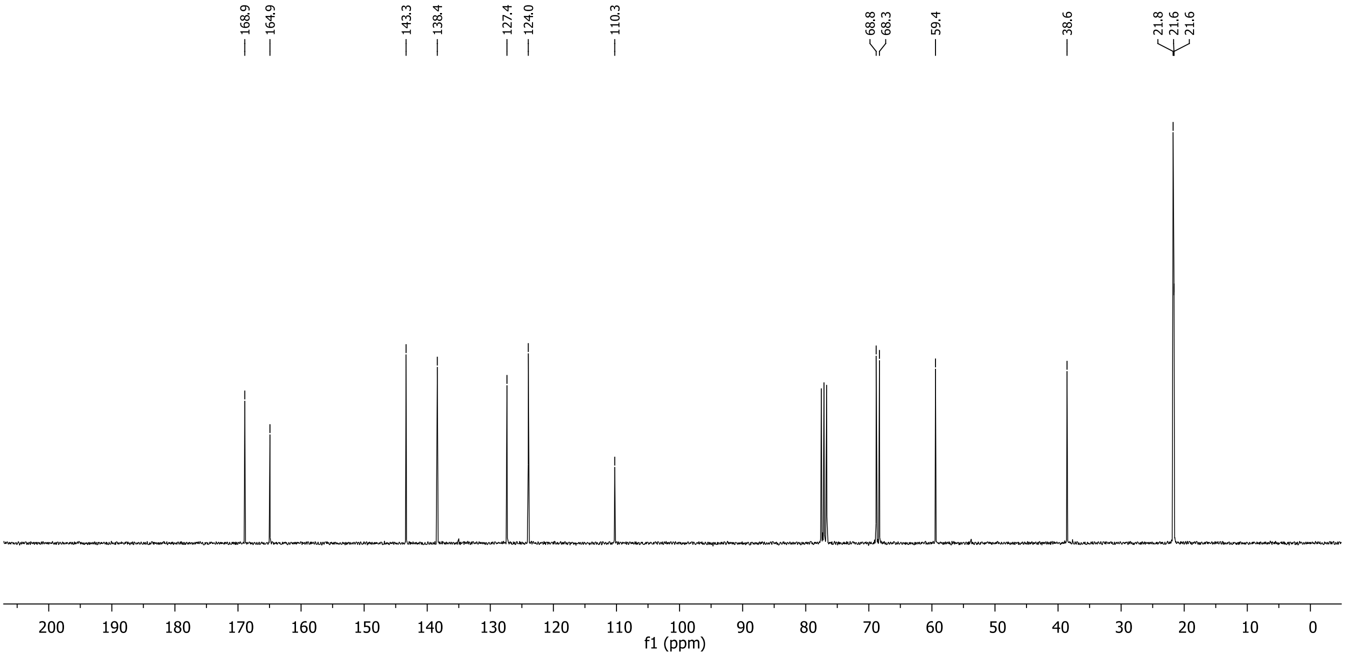

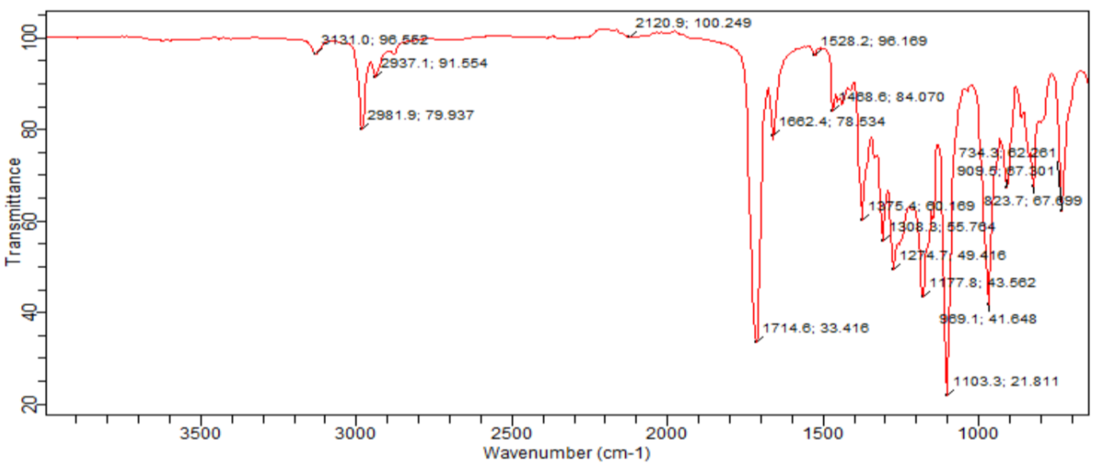

Dibenzyl (*E*)-4-(4-chloro-1H-pyrazol-1-yl)hex-2-enedioate (3n) (^{1}H NMR: 300 MHz, ^{13}C NMR: 75 MHz, CDCl₃):

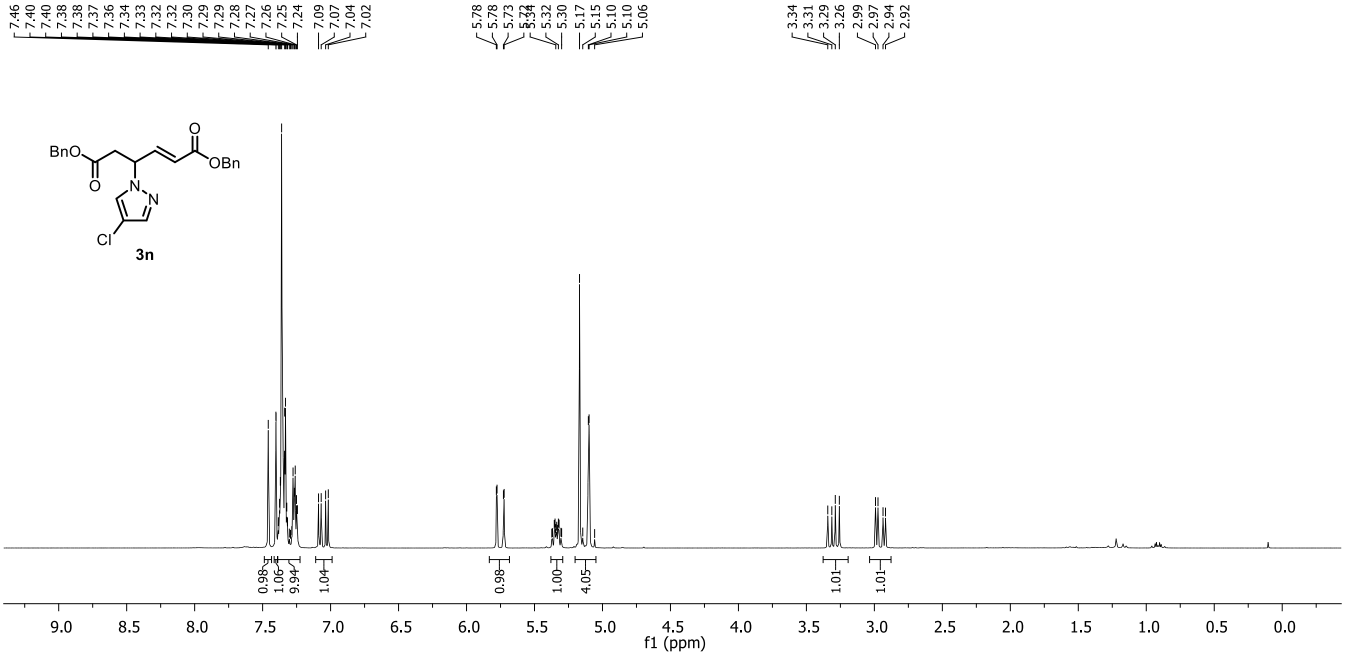

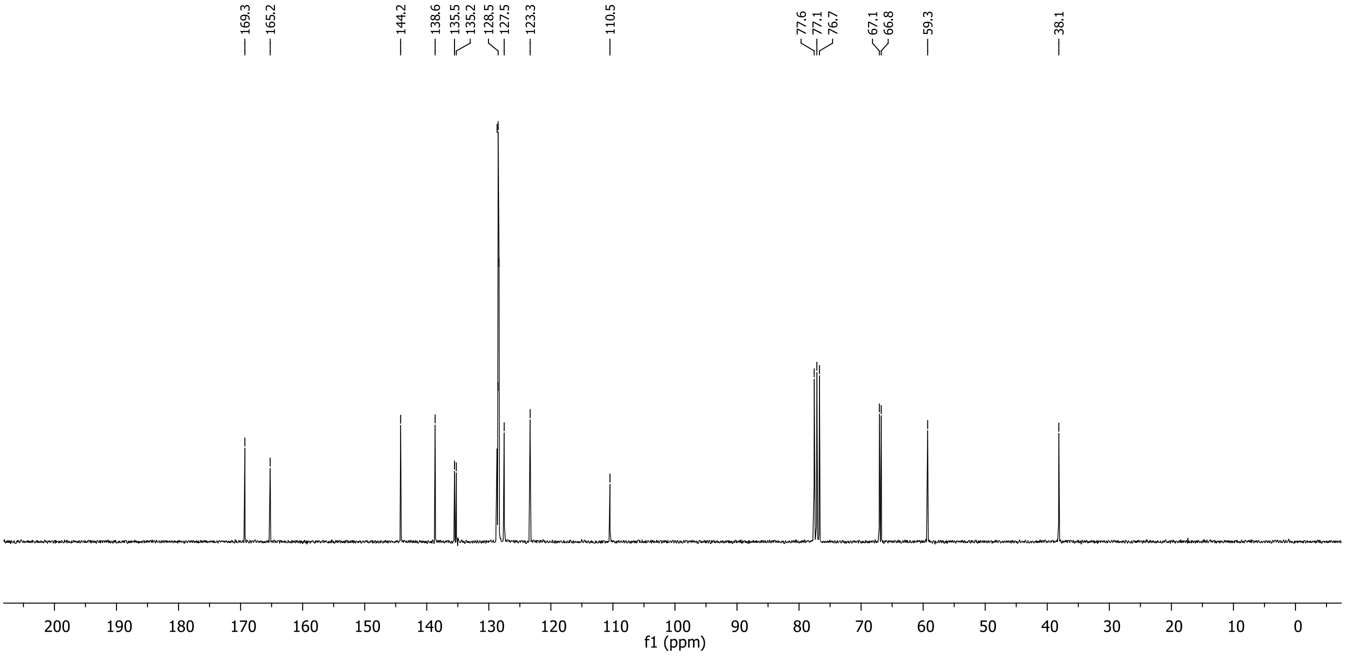

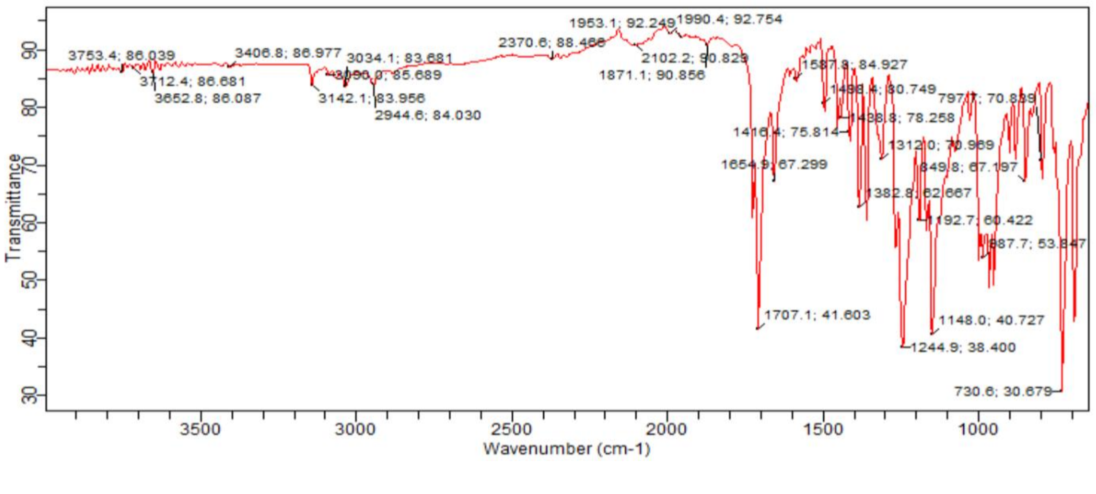

Dicyclohexyl (*E*)-4-(4-chloro-1H-pyrazol-1-yl)hex-2-enedioate (3o) (^{1}H NMR: 300 MHz, ^{13}C NMR: 75 MHz, $CDCl_3$):

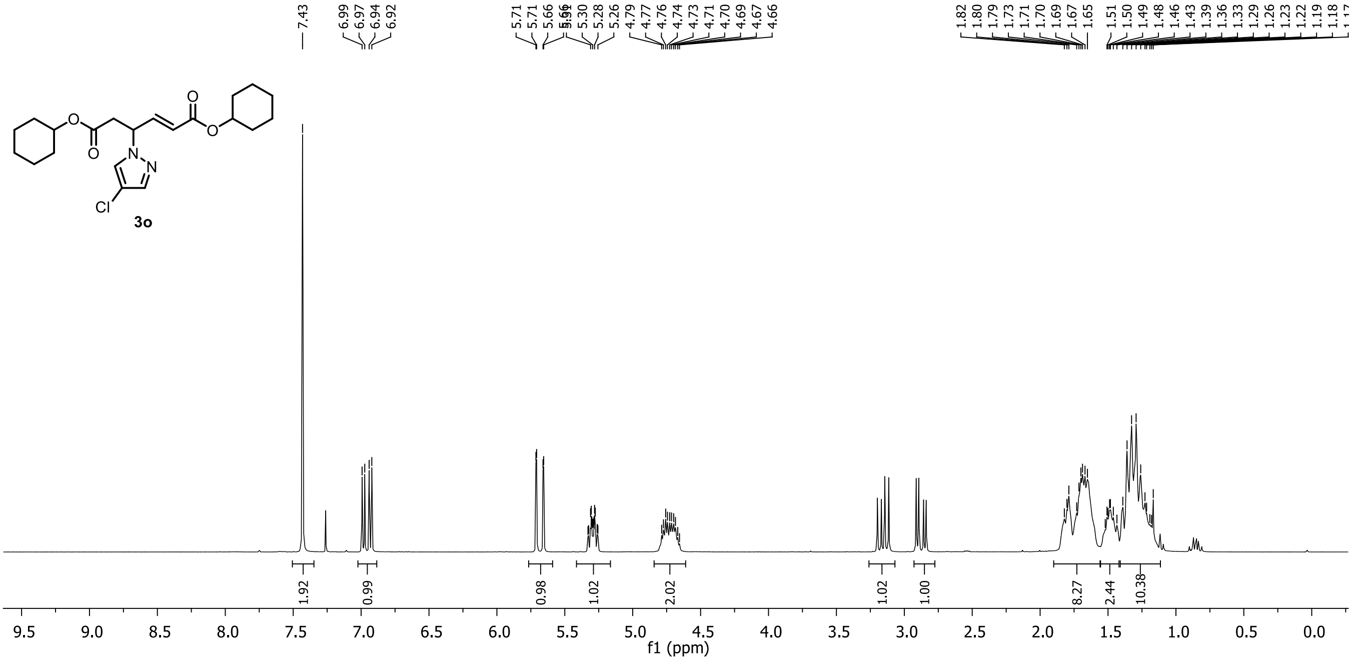

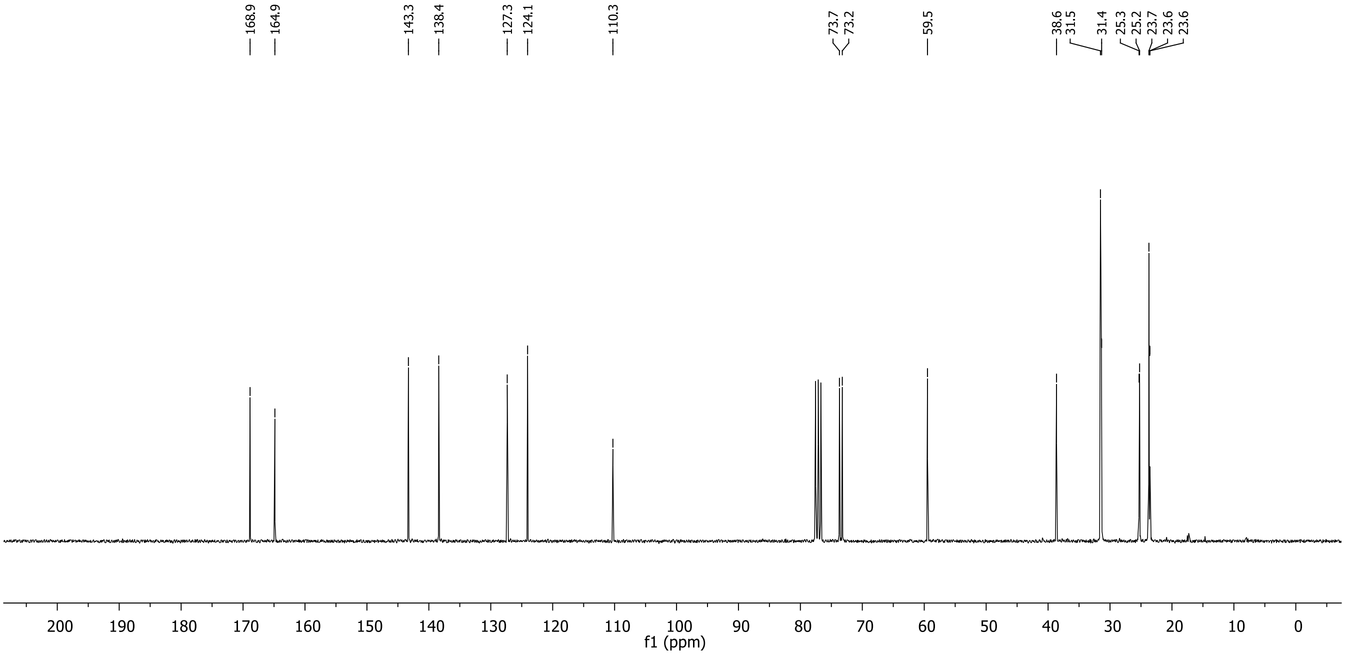

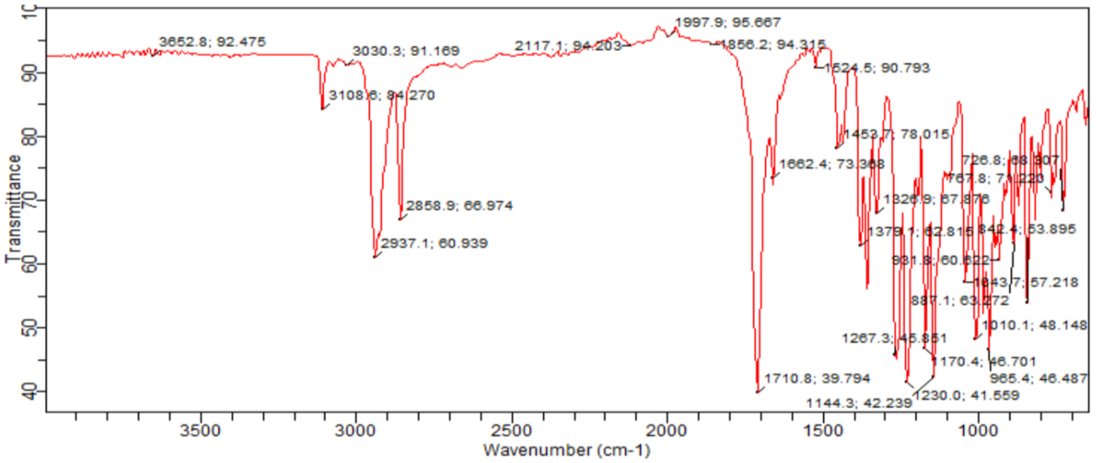

Diphenyl (*E*)-4-(4-chloro-1H-pyrazol-1-yl)hex-2-enedioate (3p) (^{1}H NMR: 300 MHz, ^{13}C NMR: 75 MHz, $CDCl_3$):

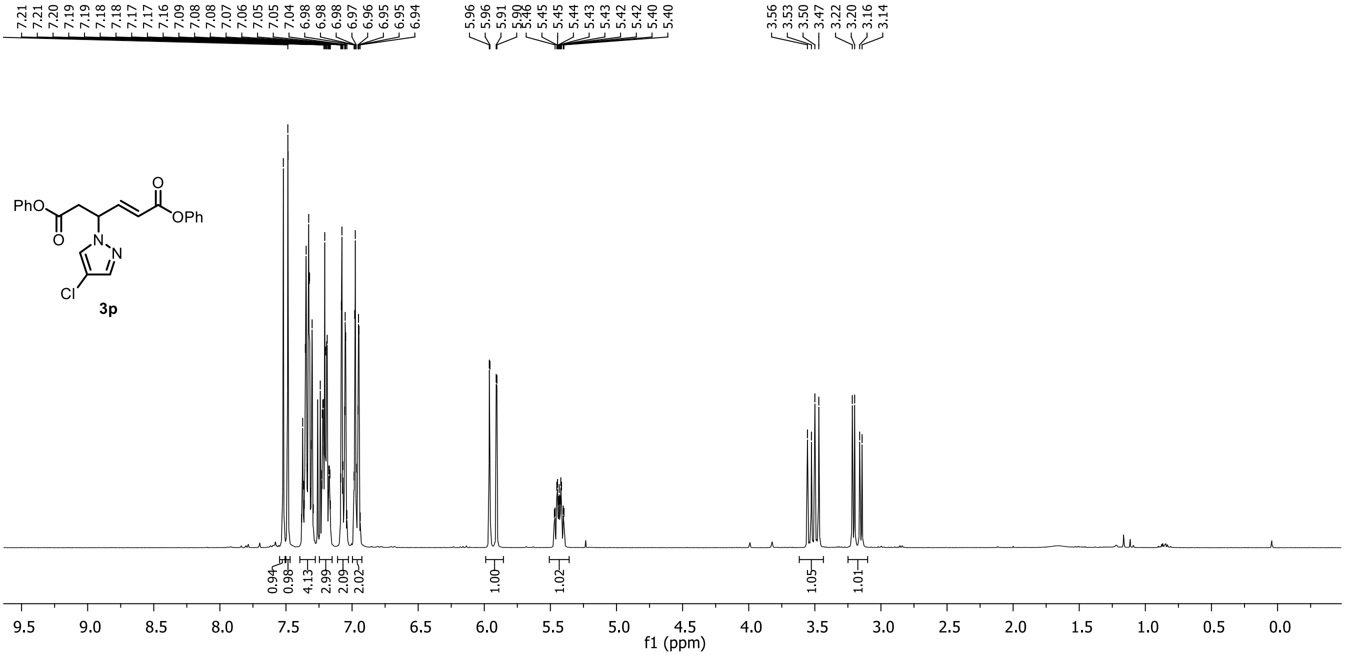

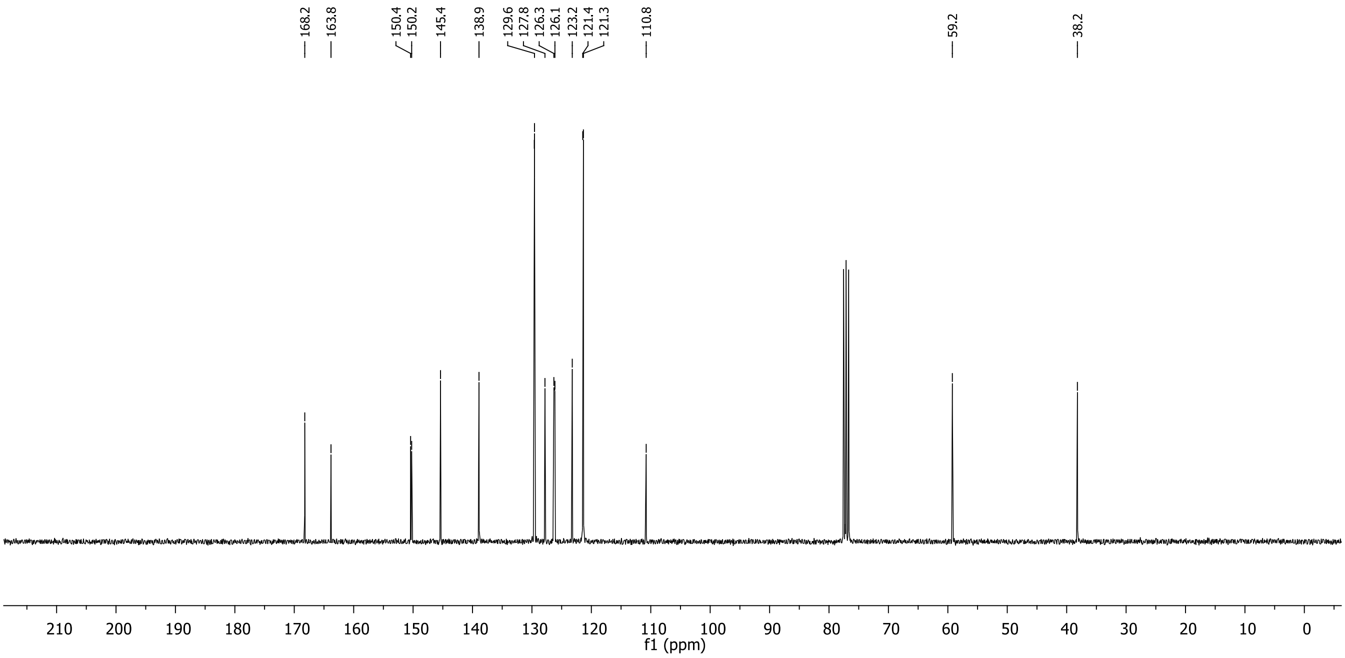

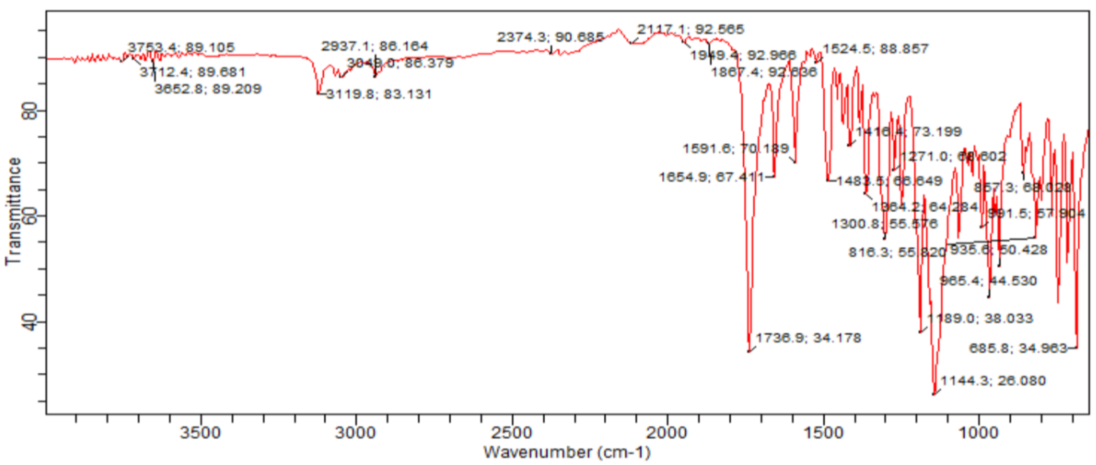

Diheptyl (*E*)-4-(4-chloro-1H-pyrazol-1-yl)hex-2-enedioate (3q) (^{1}H NMR: 300 MHz, ^{13}C NMR: 75 MHz, CDCl$_3$):

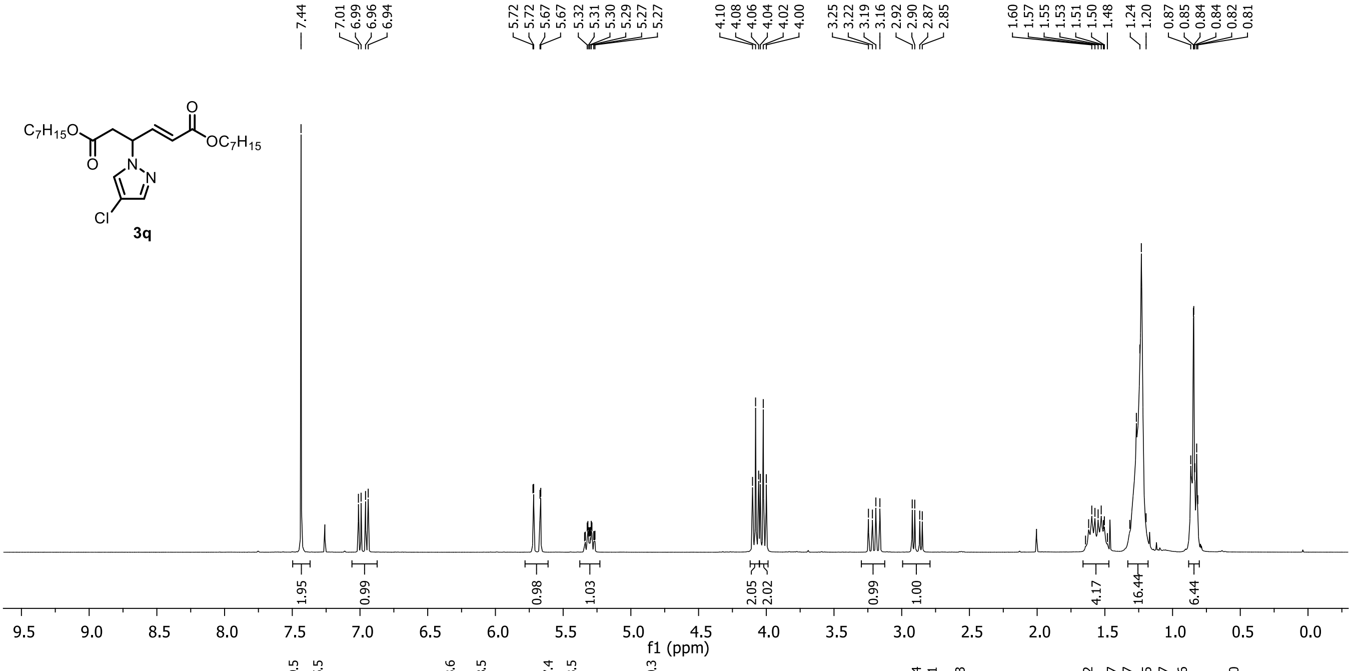

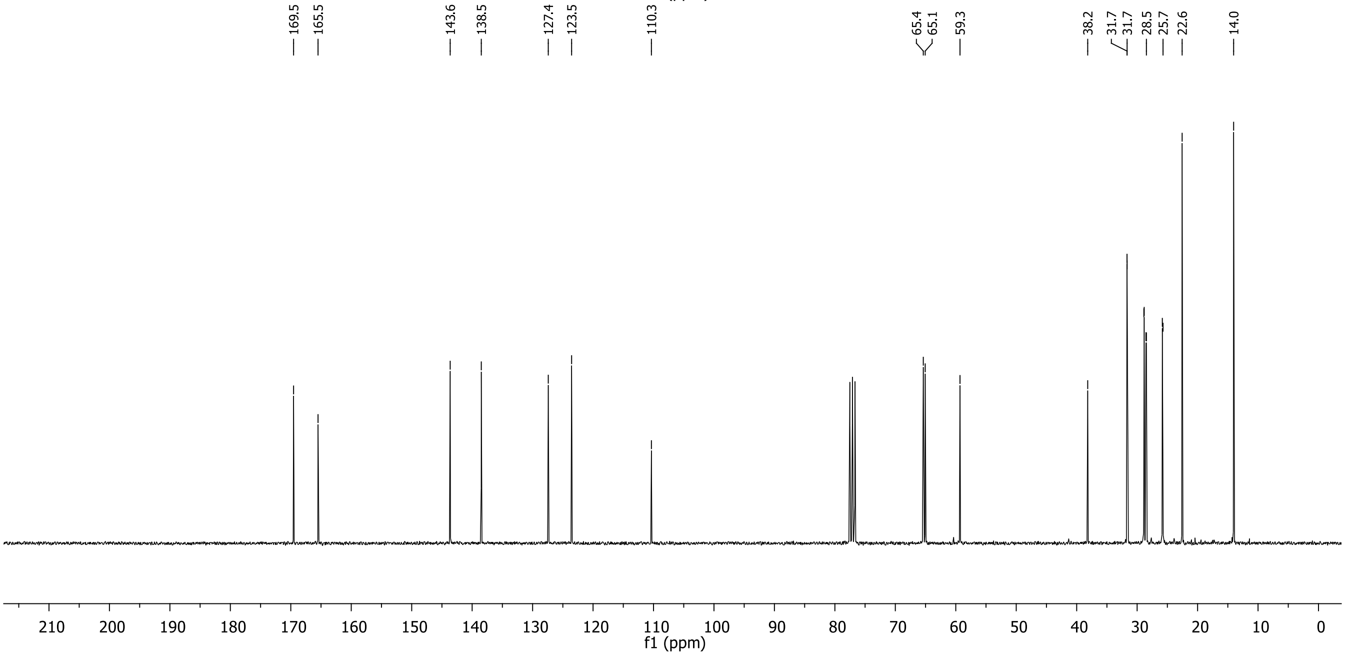

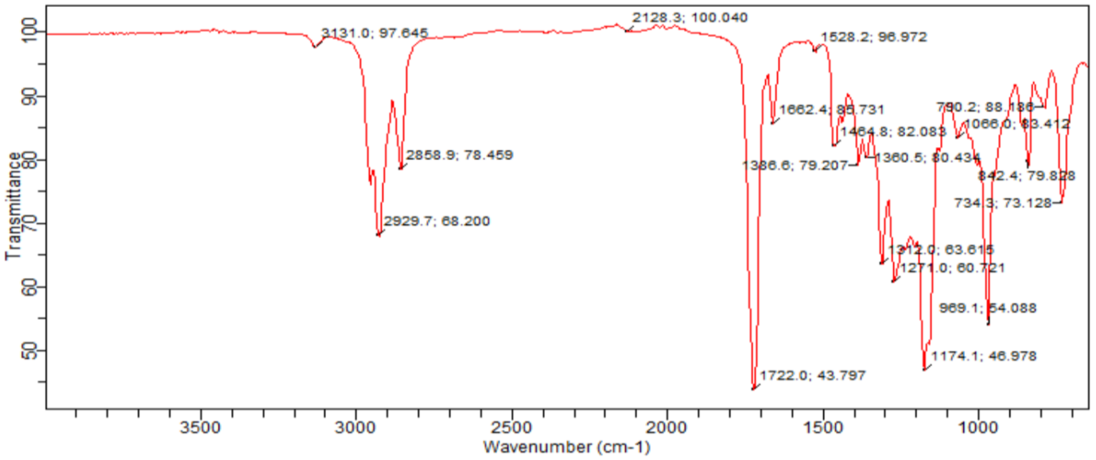

1-((1R,2S,5R)-2-isopropyl-5-methylcyclohexyl) 6-((1S,2R,5S)-2-isopropyl-5-methylcyclohexyl) (*E*)-4-(4-chloro-1H-pyrazol-1-yl)hex-2-enedioate (3r) (^{1}H NMR: 300 MHz, ^{13}C NMR: 75 MHz, $CDCl_3$):

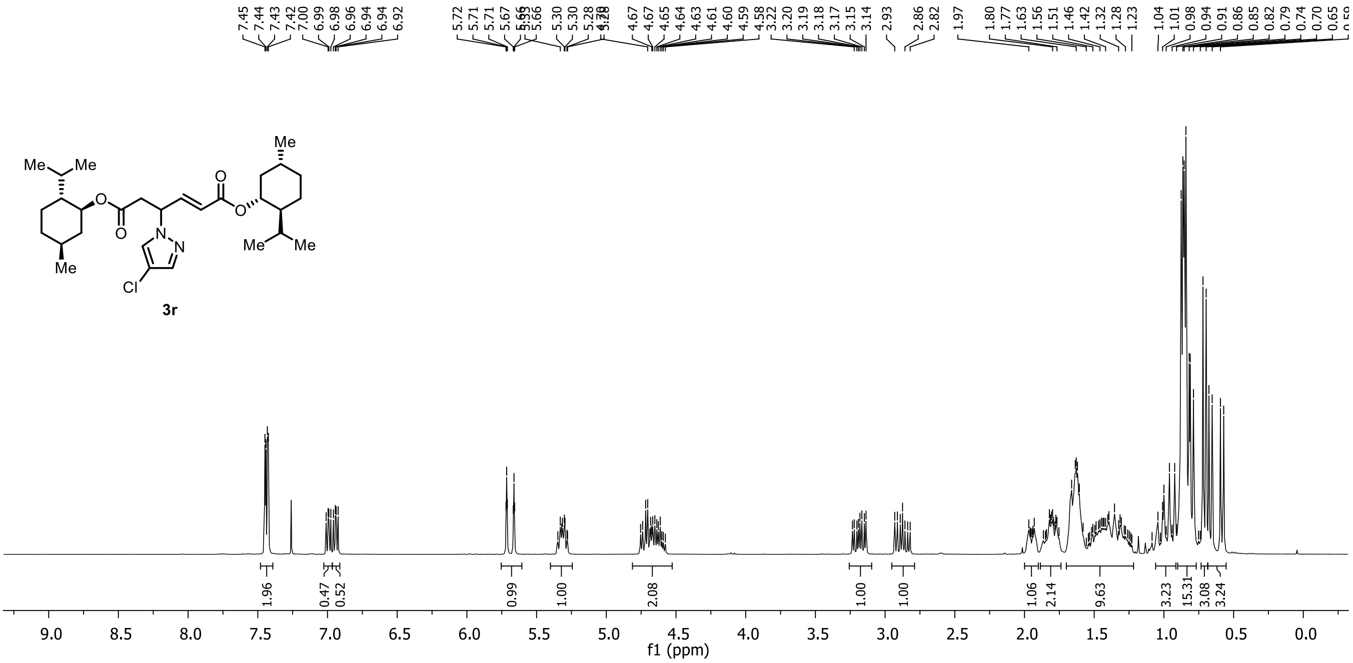

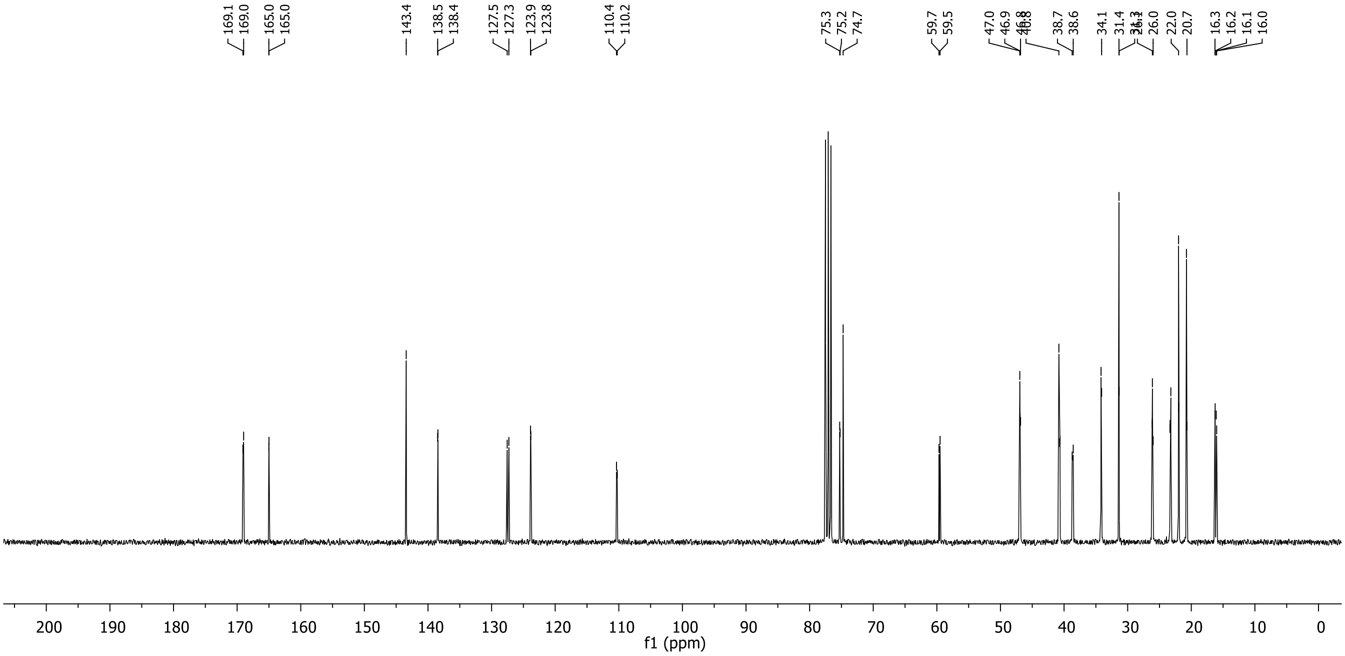

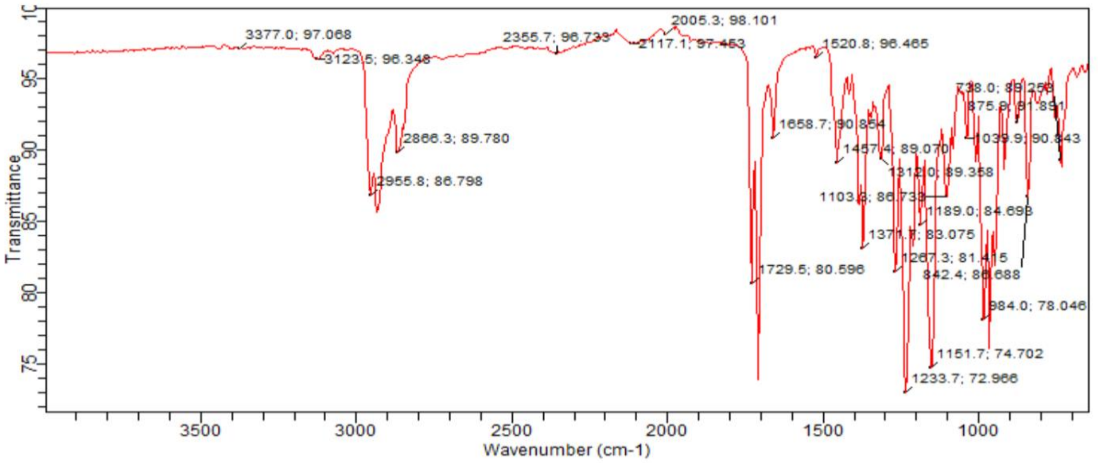

Benzyl (*E*)-4-(4-chloro-1H-pyrazol-1-yl)hex-2-enoate (3s) (^{1}H NMR: 300 MHz, ^{13}C NMR: 75 MHz, $CDCl_3$):

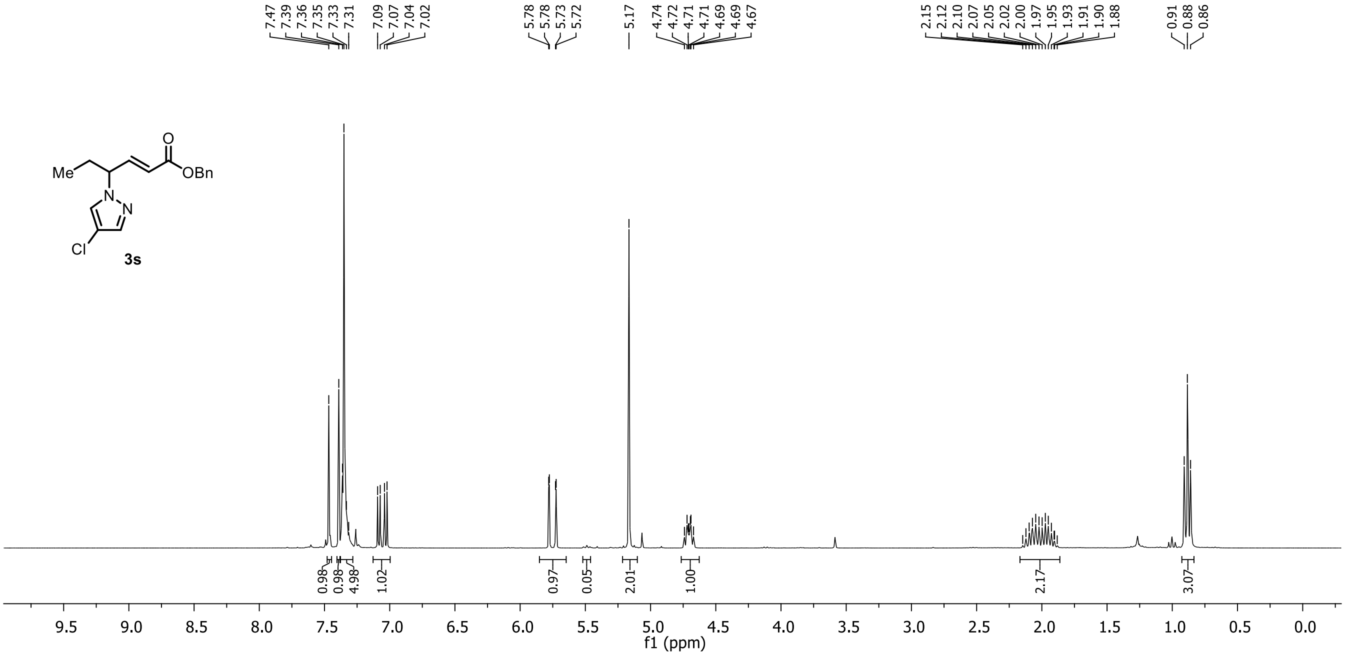

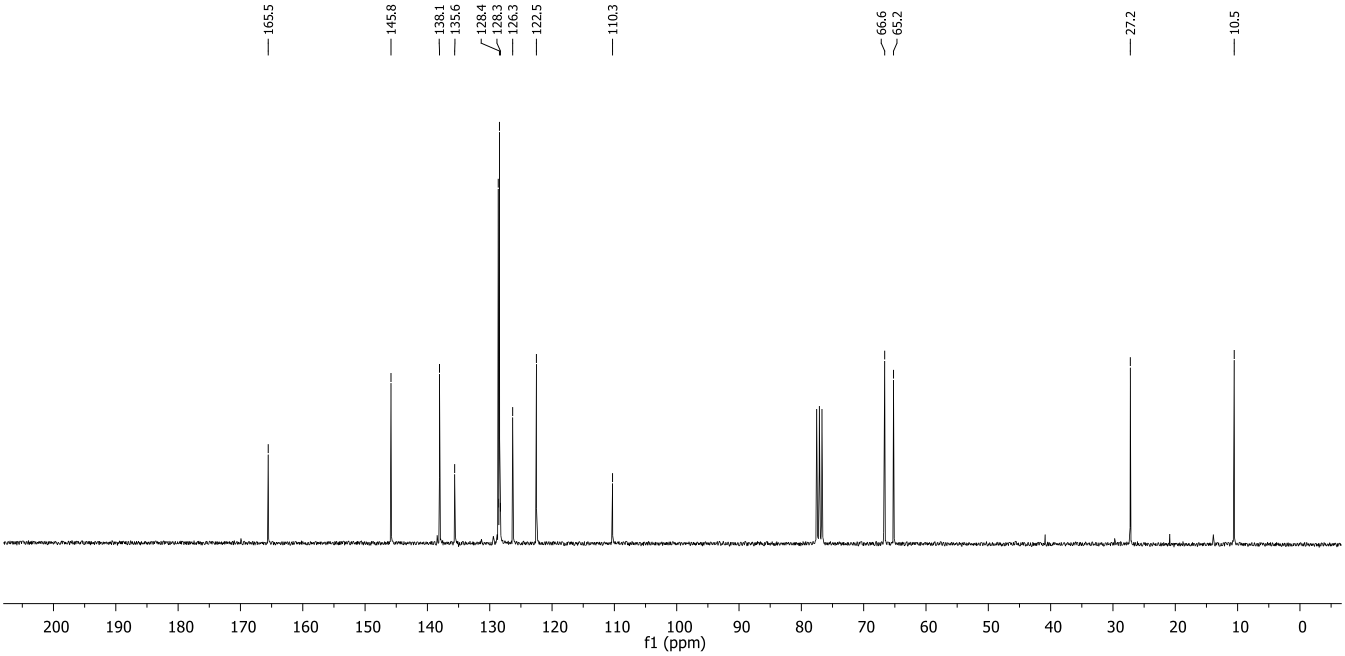

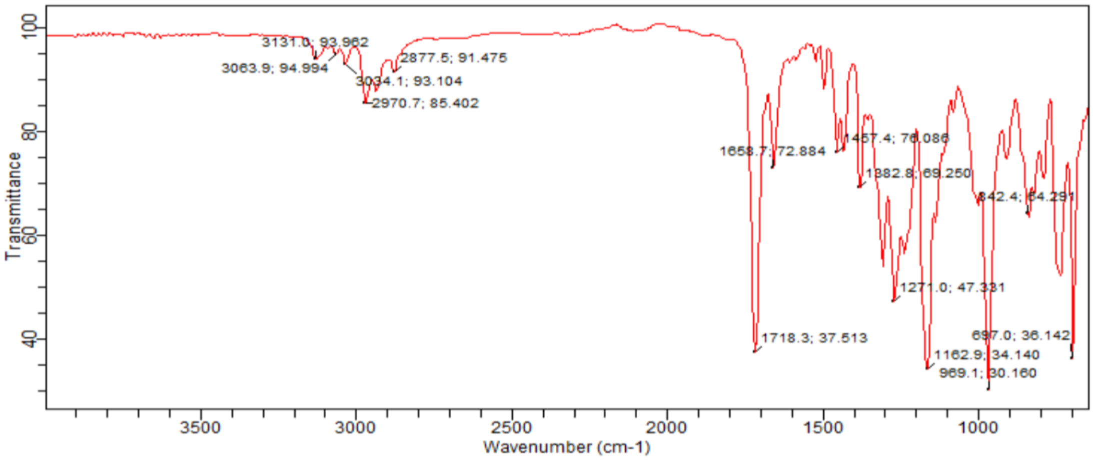

Phenyl (*E*)-4-(4-chloro-1H-pyrazol-1-yl)hex-2-enoate (3t) (^{1}H NMR: 400 MHz, ^{13}C NMR: 101 MHz, $CDCl_3$):

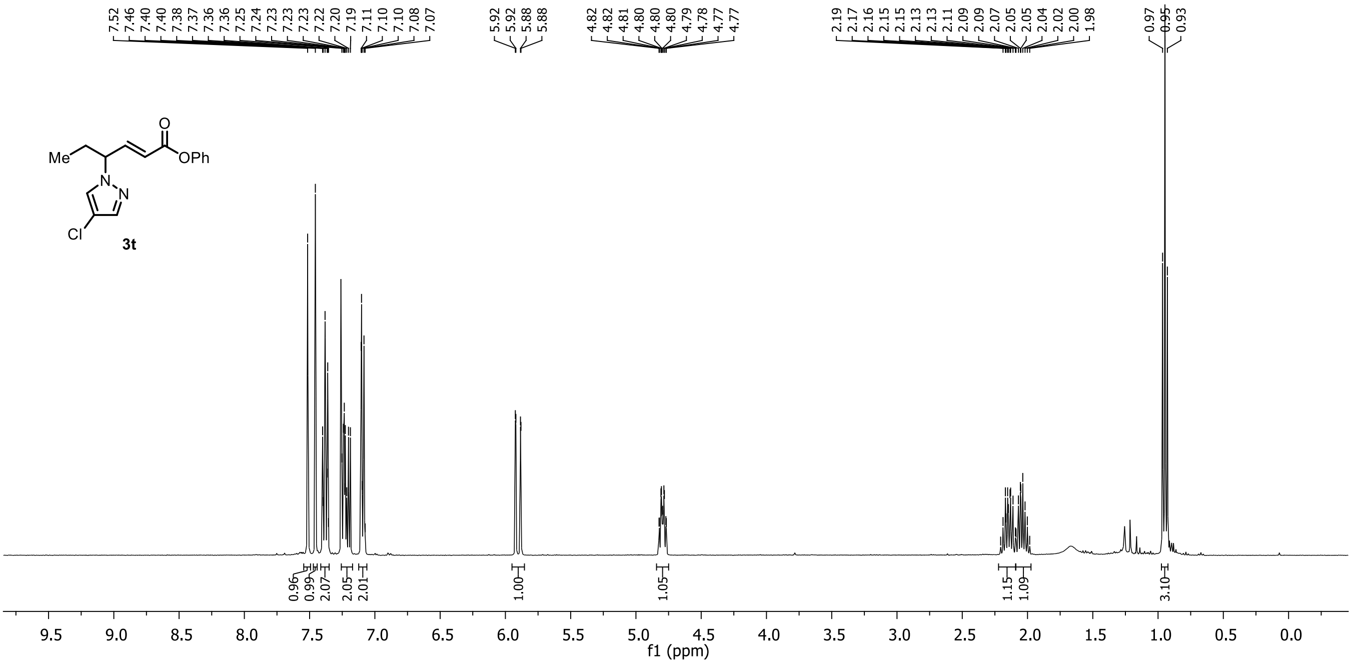

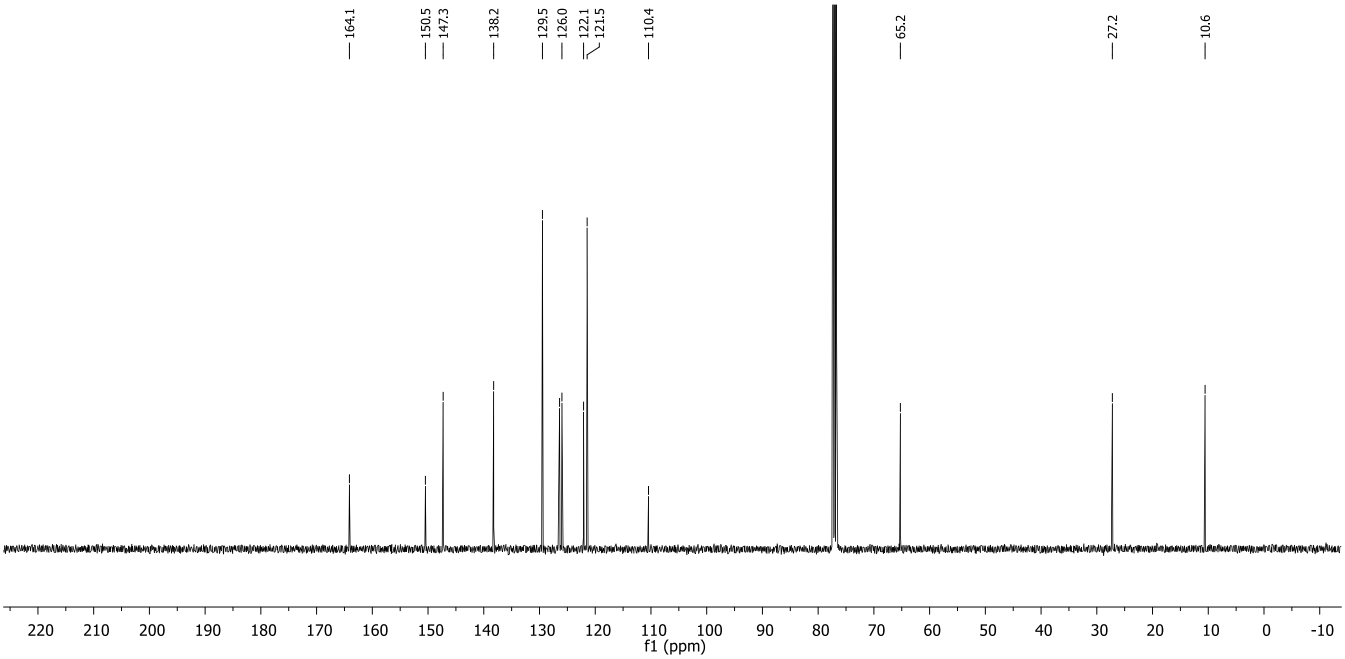

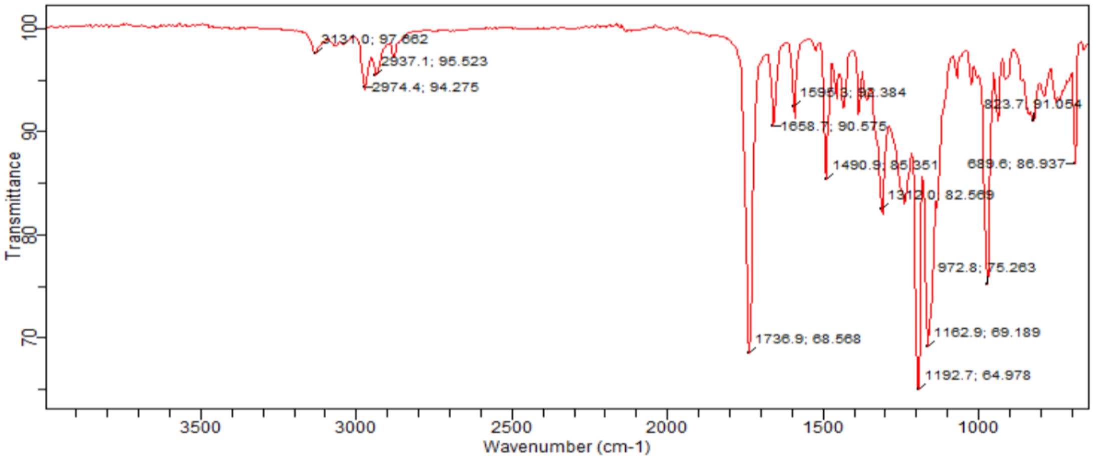

Cyclopropylmethyl (*E*)-4-(4-chloro-1H-pyrazol-1-yl)hex-2-enoate (3u) (^{1}H NMR: 400 MHz, ^{13}C NMR: 101 MHz, $CDCl_3$):

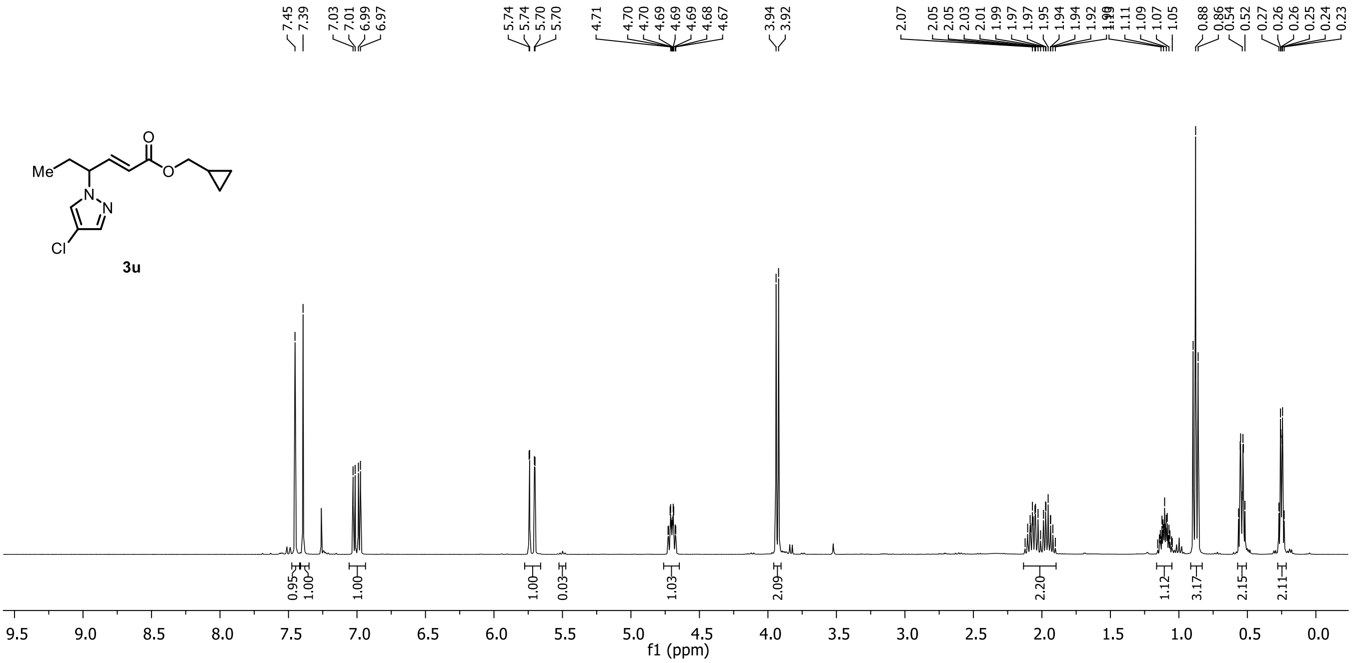

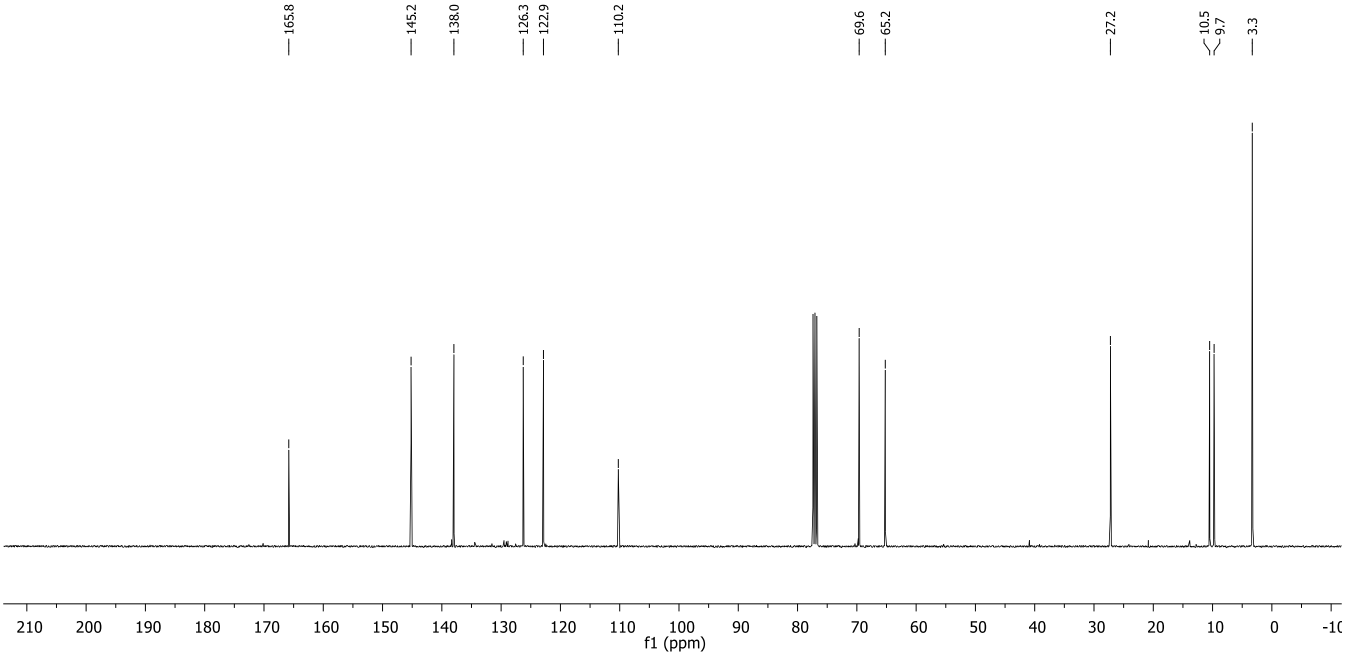

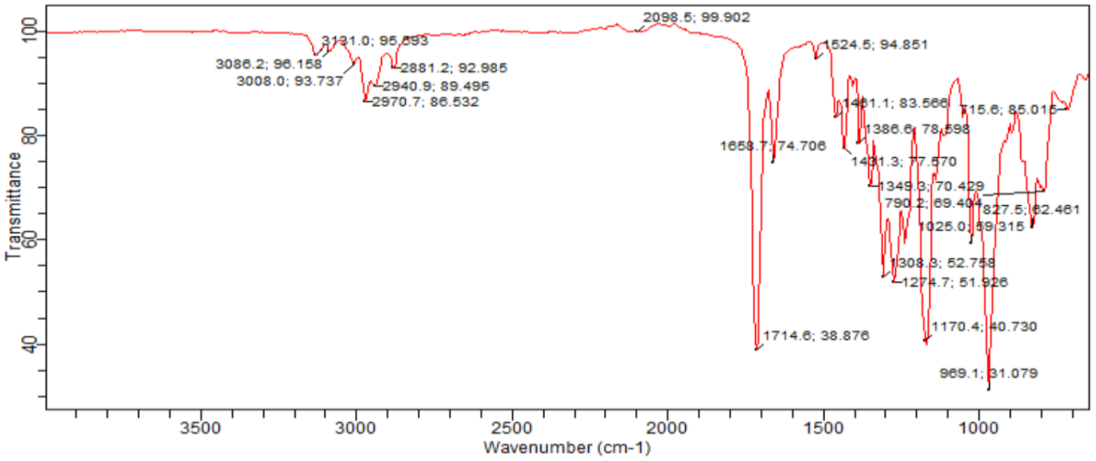

Oxiran-2-ylmethyl (*E*)-4-(4-chloro-1H-pyrazol-1-yl)hex-2-enoate (3v) (^{1}H NMR: 300 MHz, ^{13}C NMR: 75 MHz, $CDCl_3$):

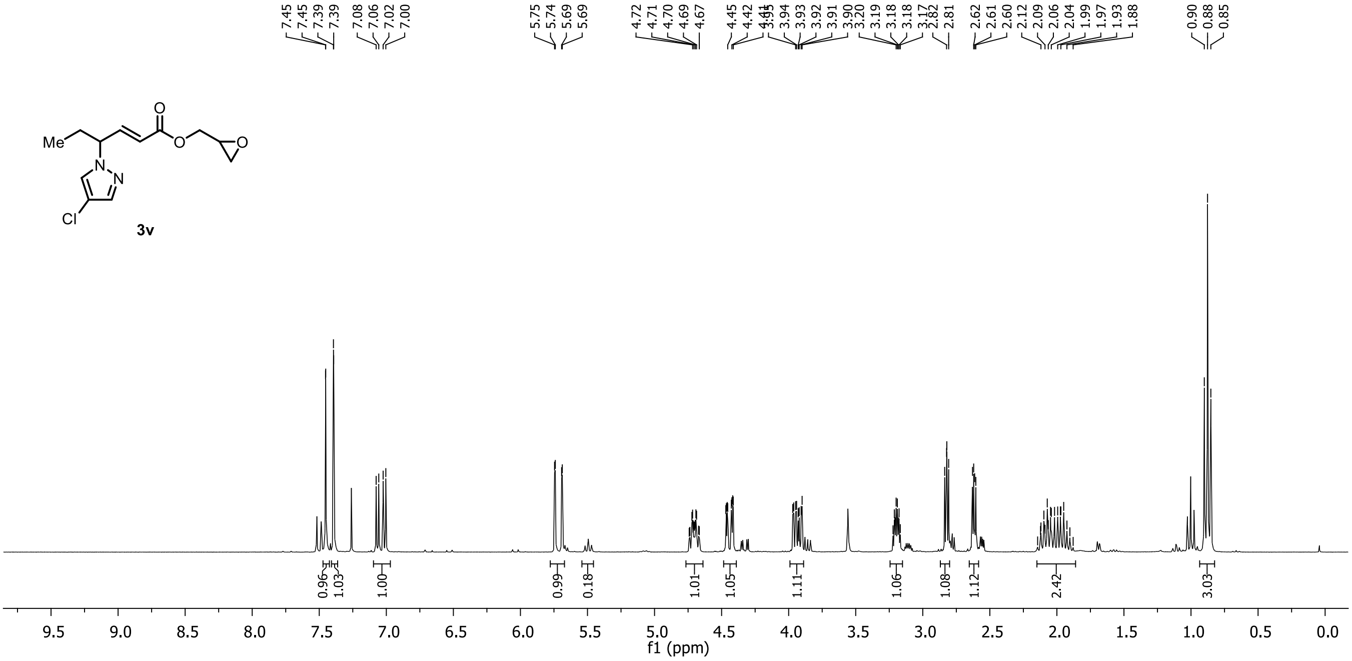

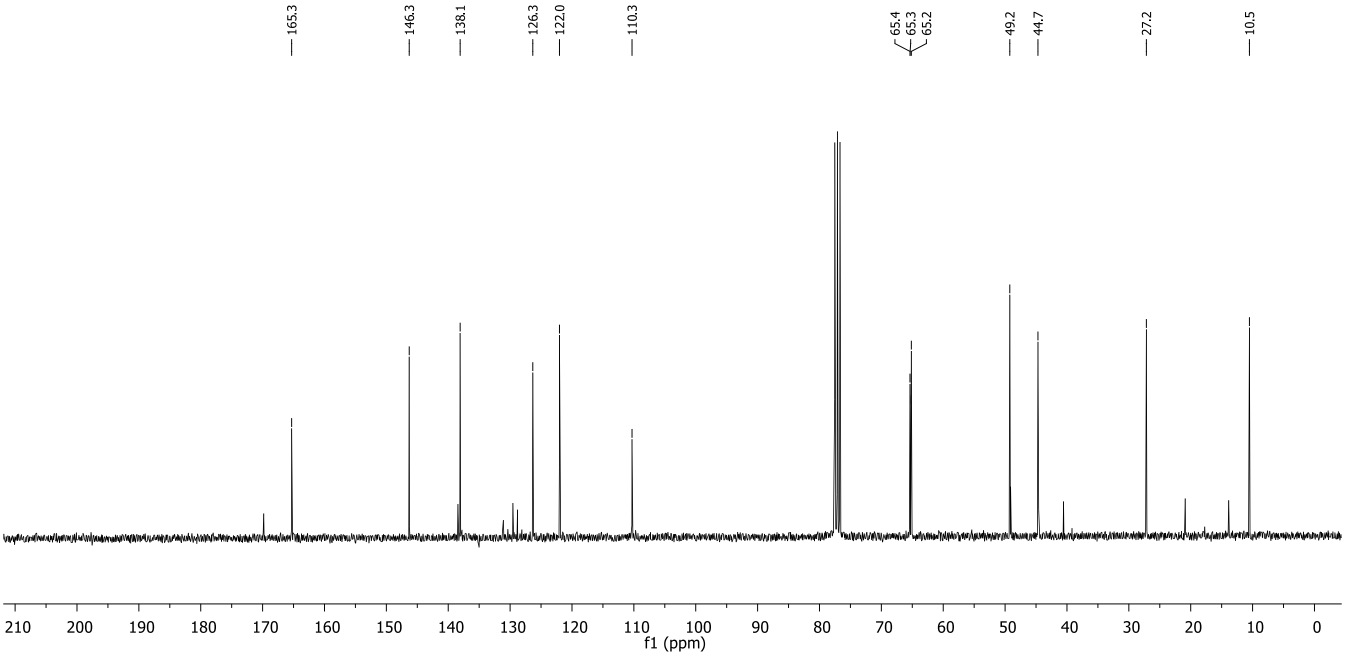

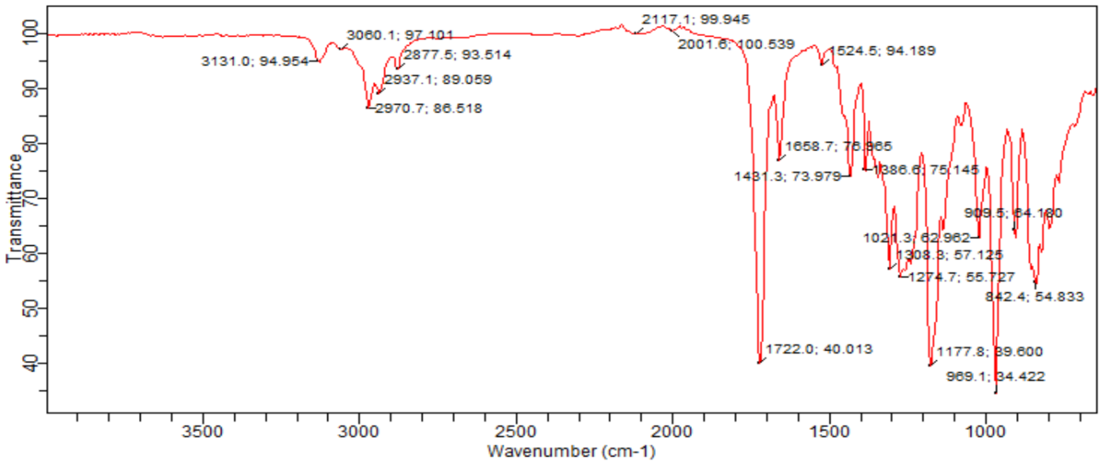

Pent-3-yn-1-yl (*E*)-4-(4-chloro-1H-pyrazol-1-yl)hex-2-enoate (3w) (^{1}H NMR: 300 MHz, ^{13}C NMR: 75 MHz, $CDCl_3$):

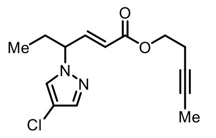

3w

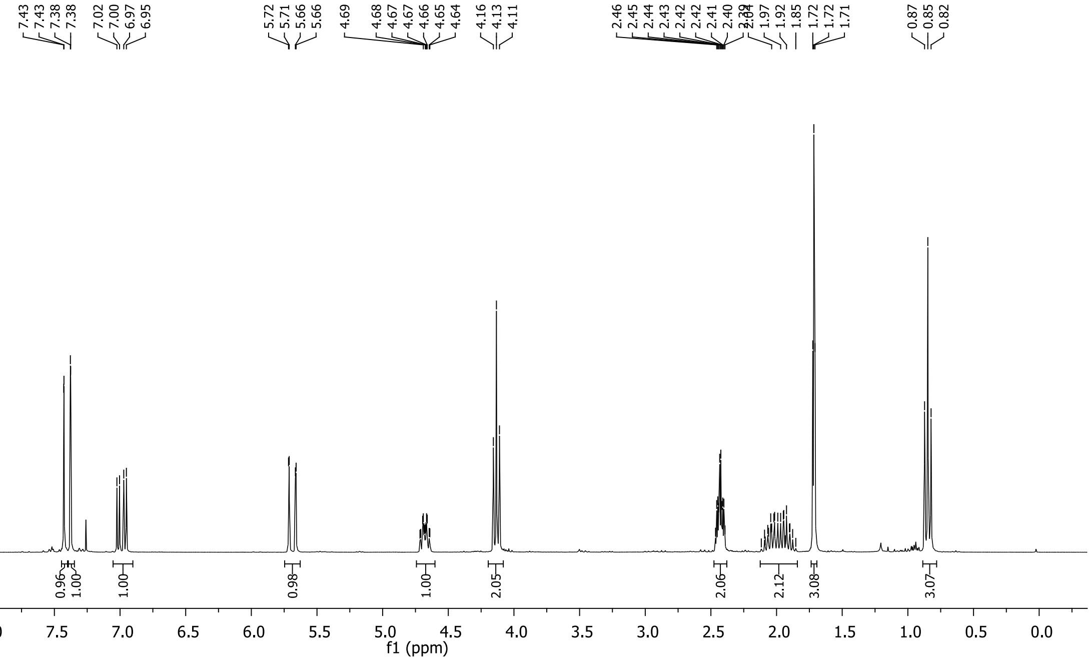

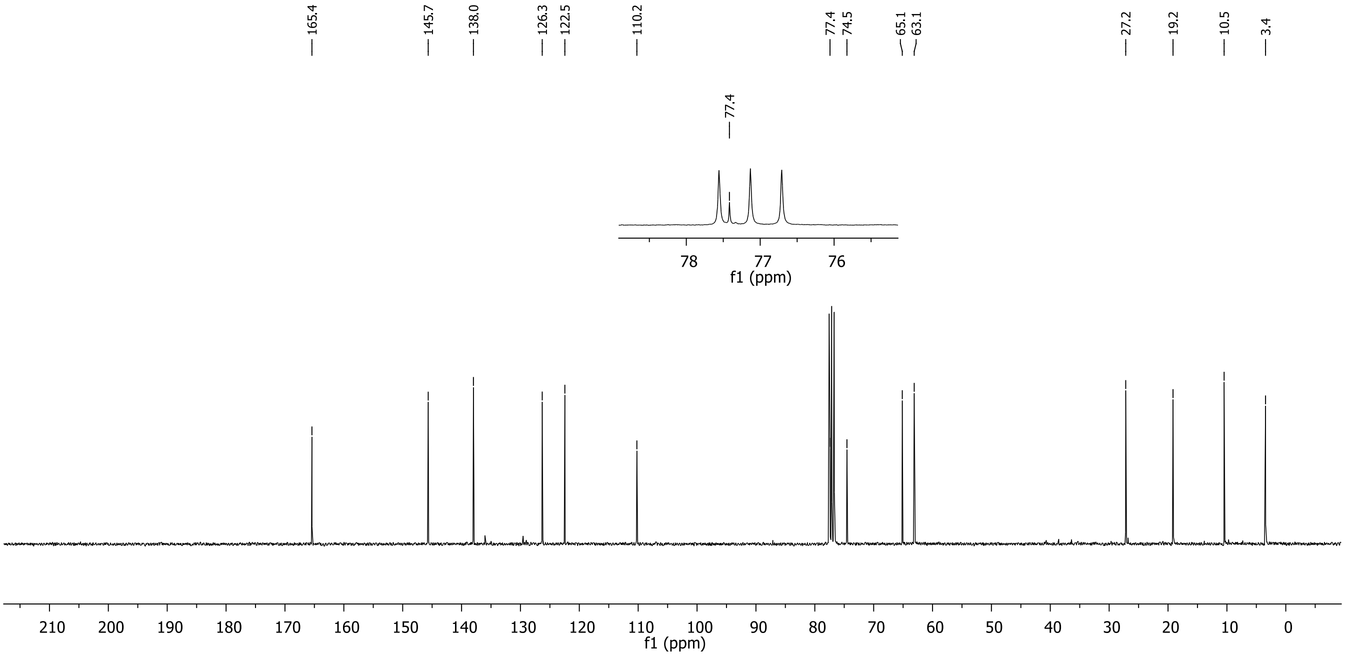

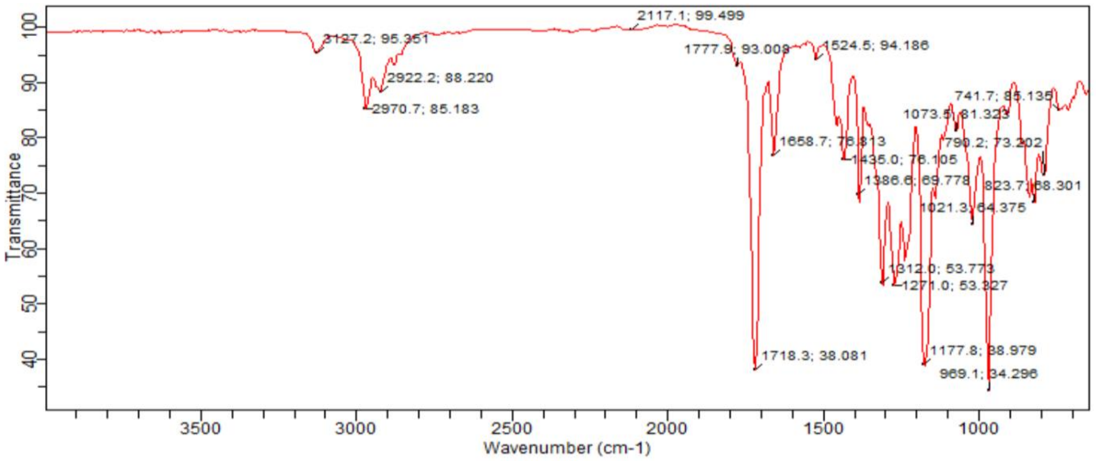

4-Oxocyclopent-2-en-1-yl (*E*)-4-(4-chloro-1H-pyrazol-1-yl)hex-2-enoate (3x) (^{1}H NMR: 300 MHz, ^{13}C NMR: 75 MHz, $CDCl_3$):

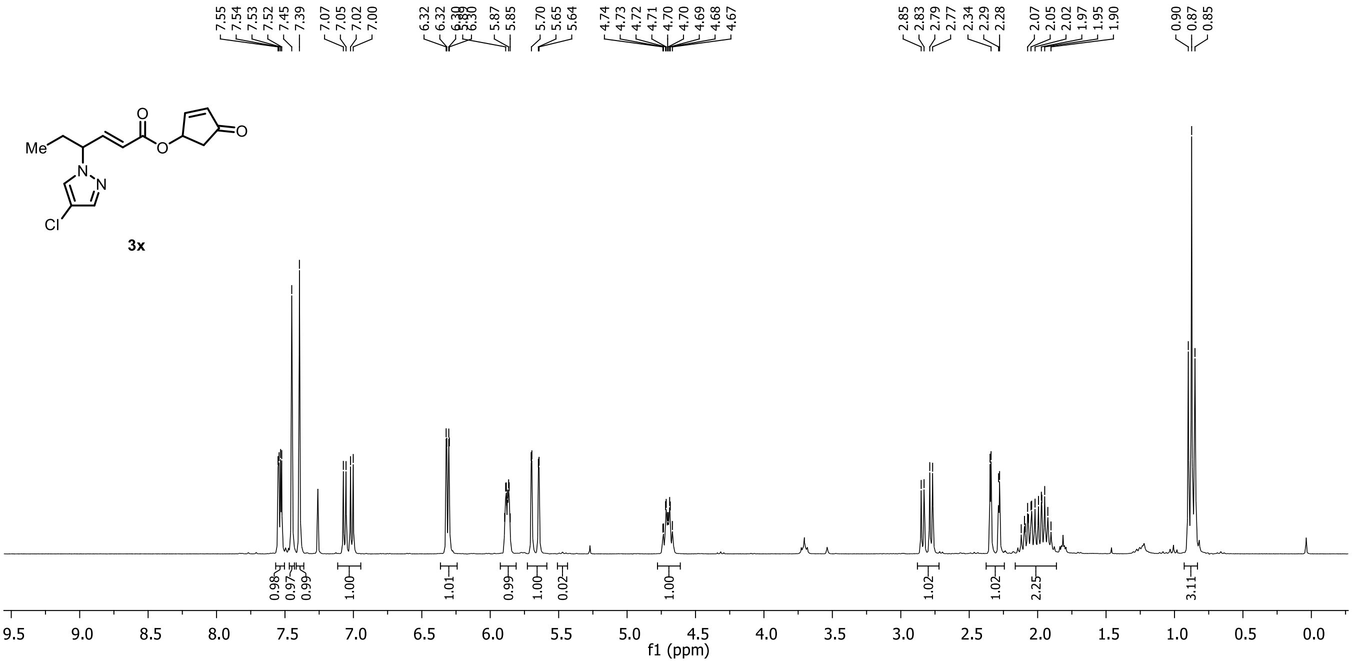

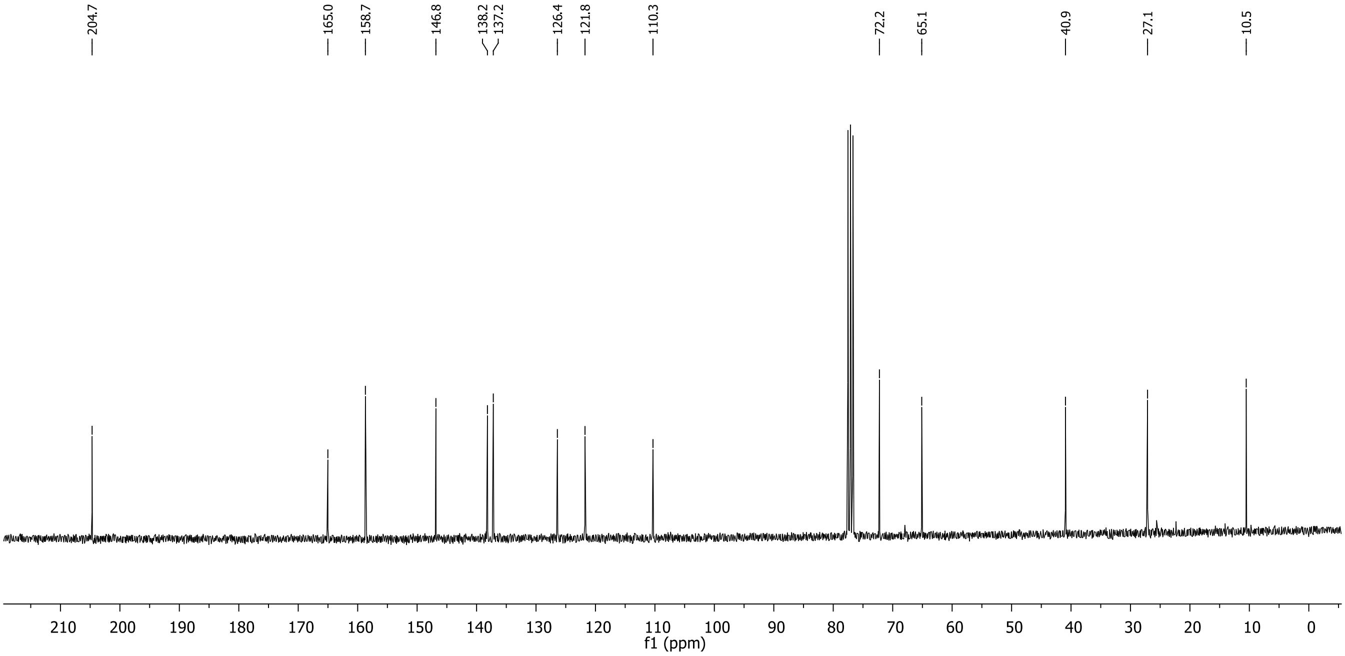

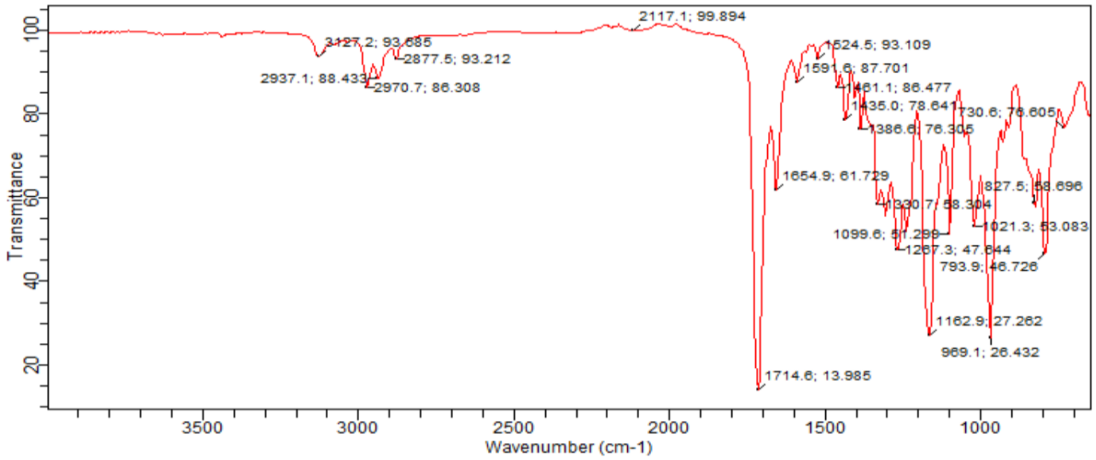

Cyclopent-3-en-1-yl (*E*)-4-(4-chloro-1H-pyrazol-1-yl)hex-2-enoate (3y) (^{1}H NMR: 300 MHz, ^{13}C NMR: 75 MHz, $CDCl_3$):

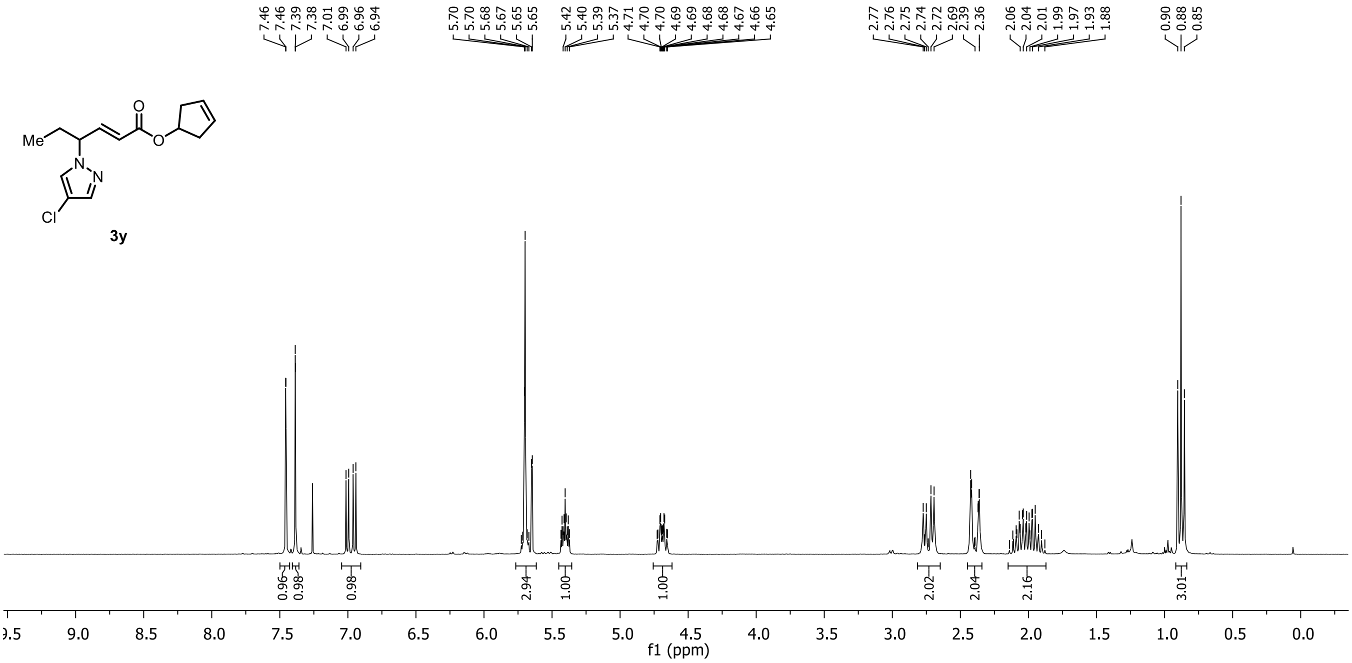

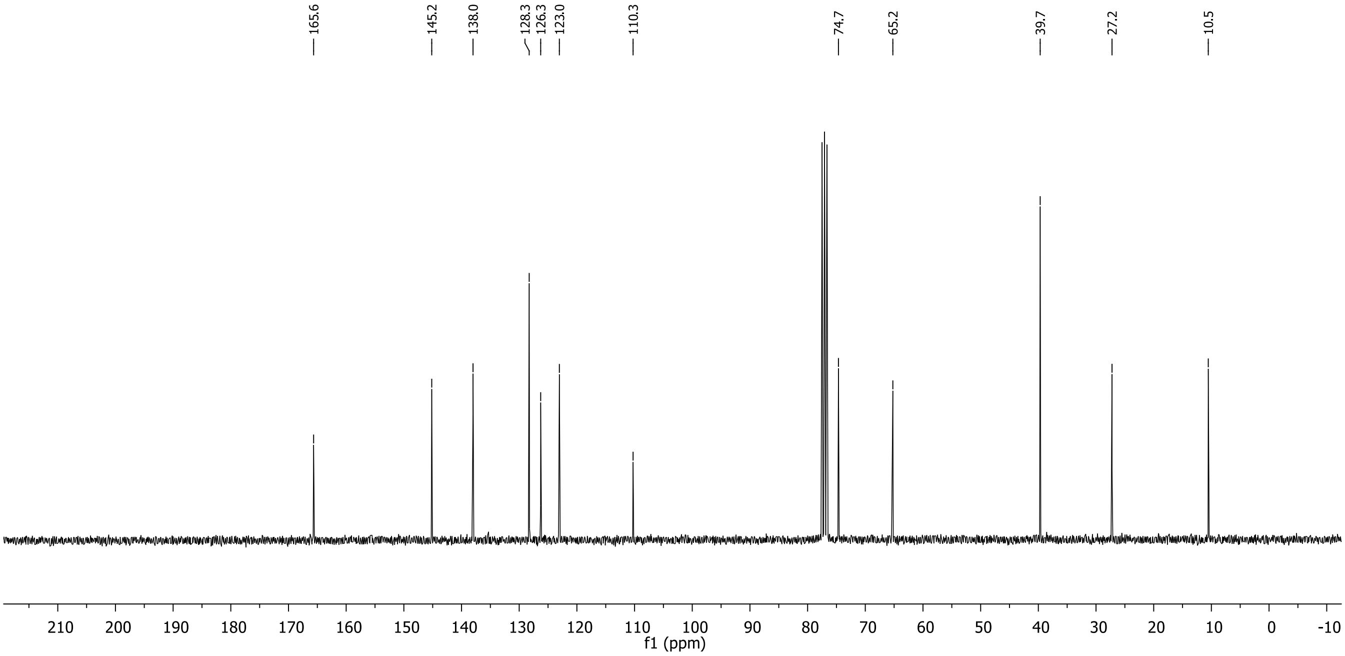

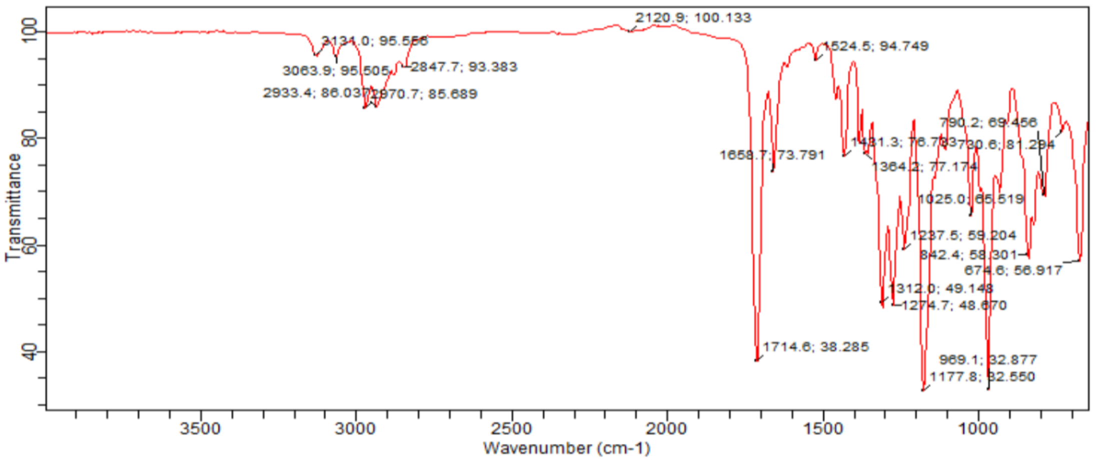

Ethyl (*E*)-4-(4-chloro-1H-pyrazol-1-yl)-2-ethylhex-2-enoate (3z) (^{1}H NMR: 400 MHz, ^{13}C NMR: 101 MHz, 2D NOESY NMR: 400 MHz, $CDCl_3$):

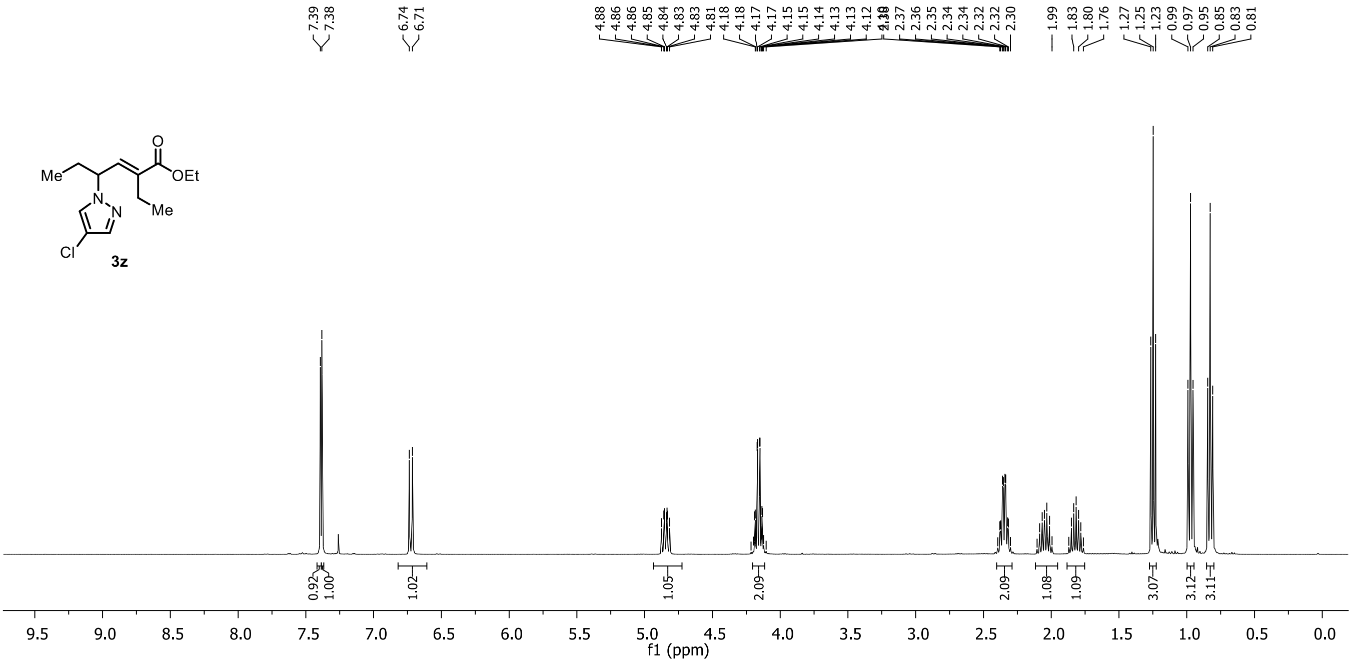

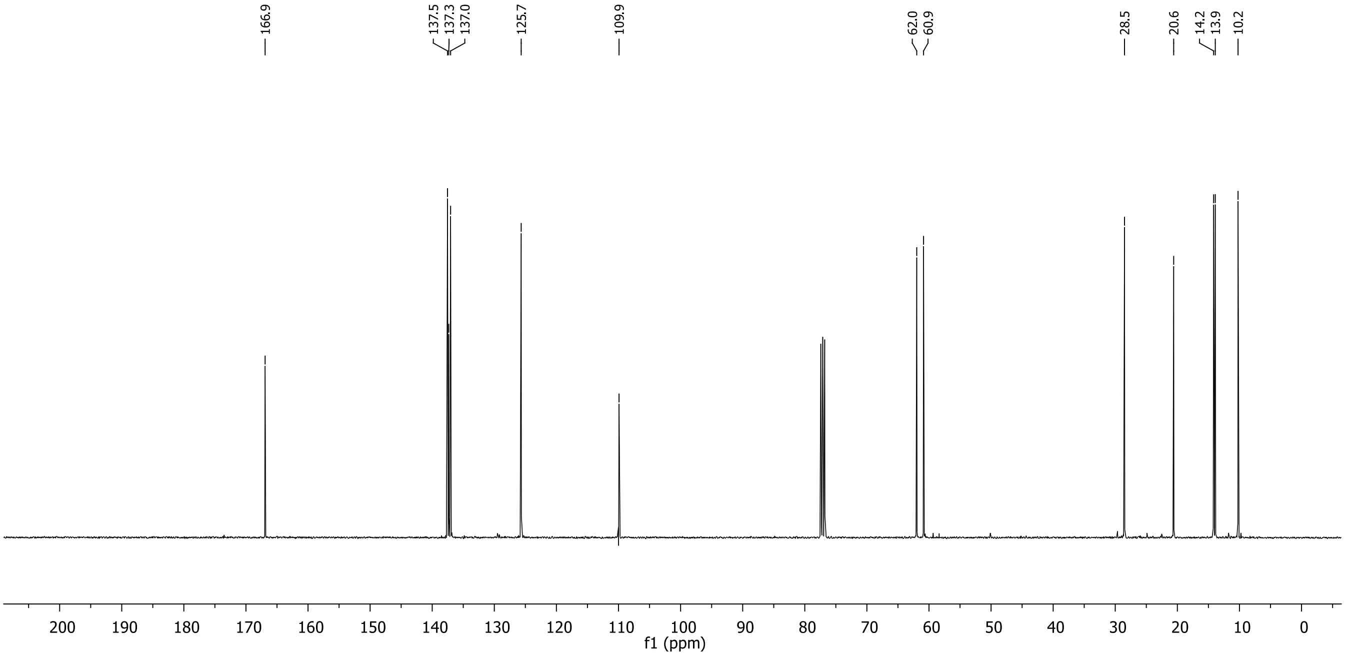

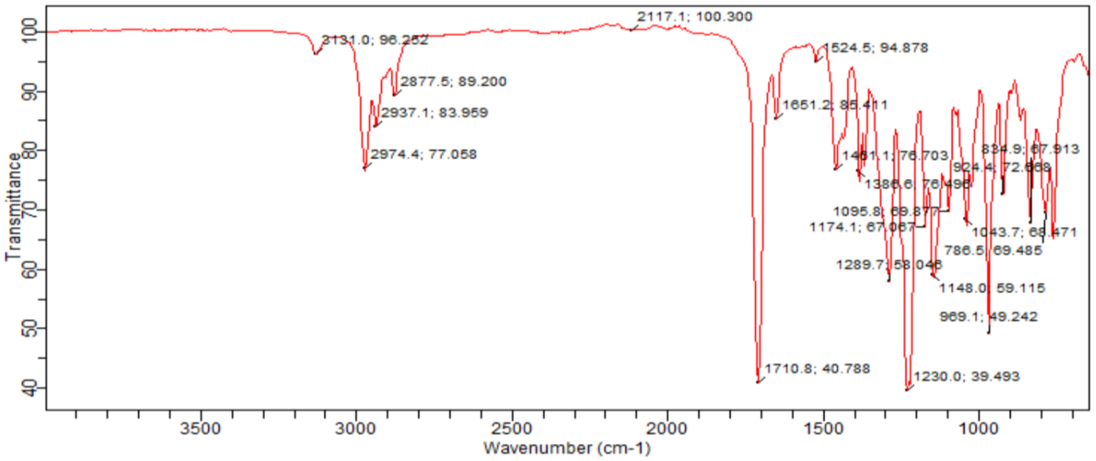

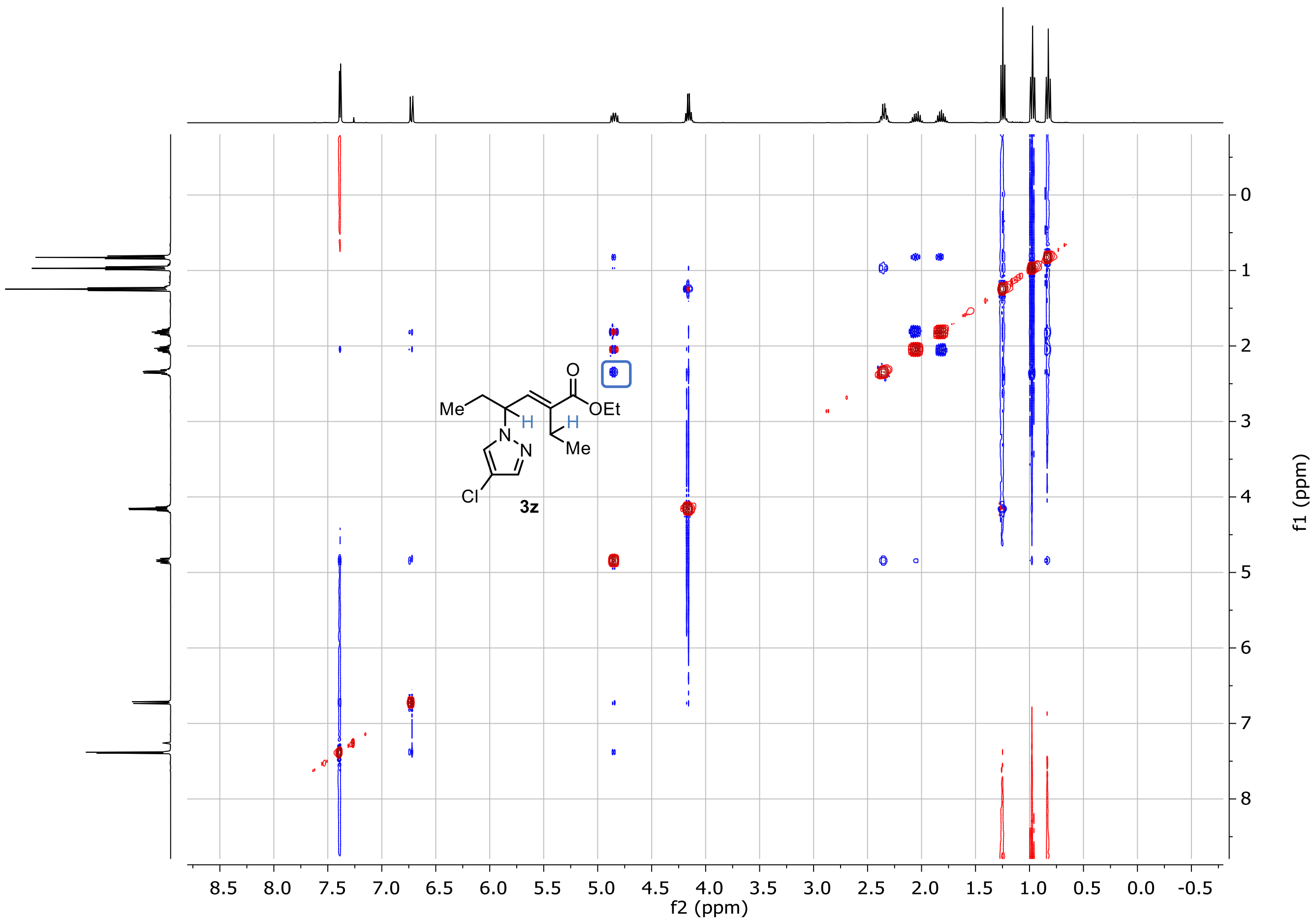
Me
H
O
OEt
H
N
N
Me
Cl
3z
f2 (ppm)
f1 (ppm)

Butyl (*E*)-4-(4-chloro-1H-pyrazol-1-yl)-2-methylbut-2-enoate (3aa) (^{1}H NMR: 400 MHz, ^{13}C NMR: 101 MHz, 2D NOESY NMR: 400 MHz, $CDCl_3$):

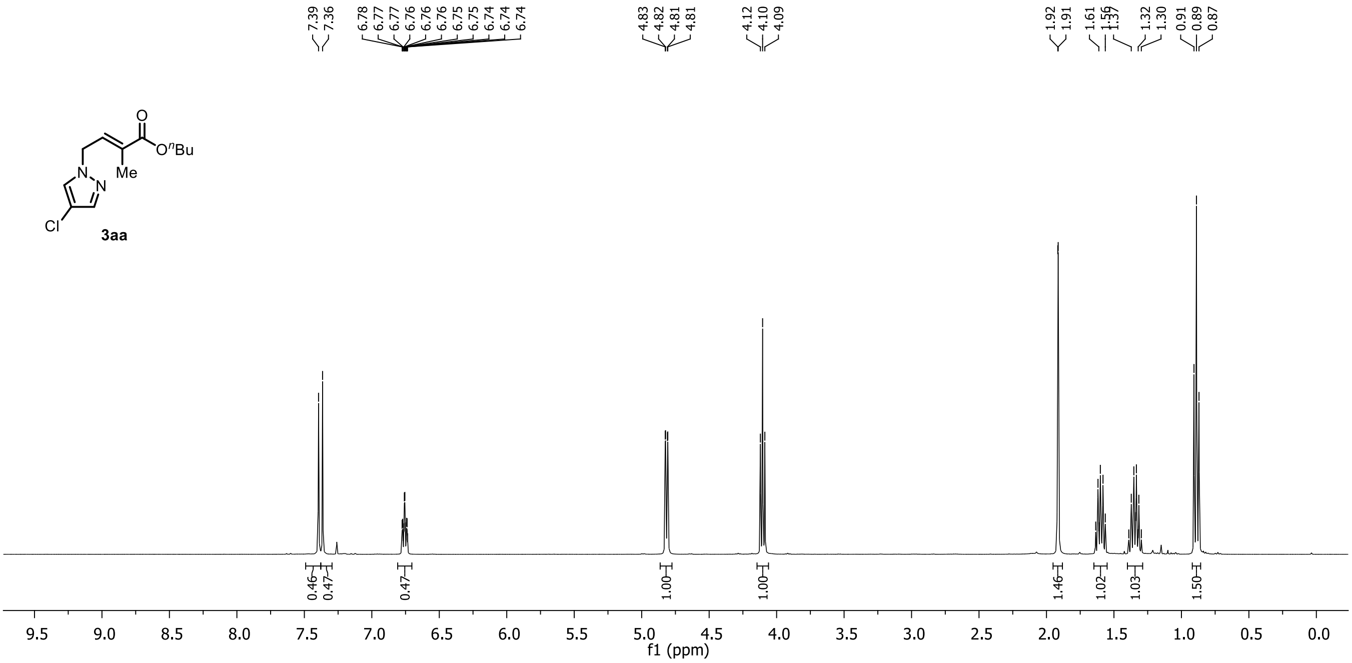

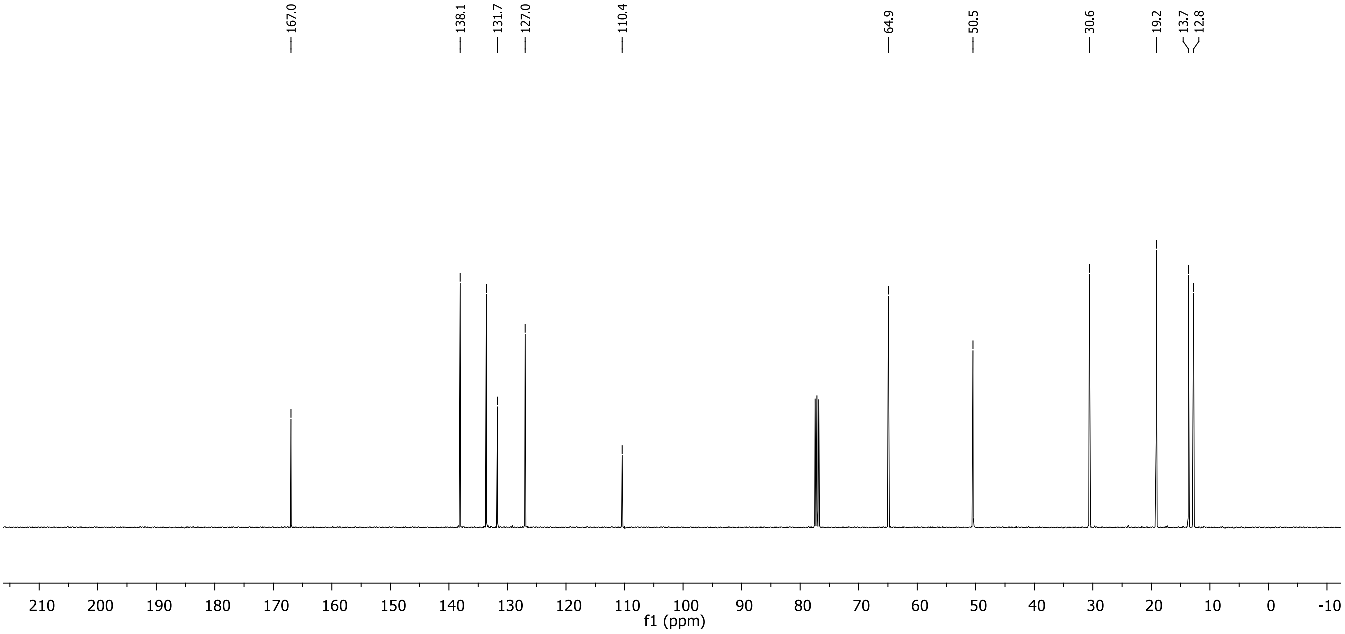

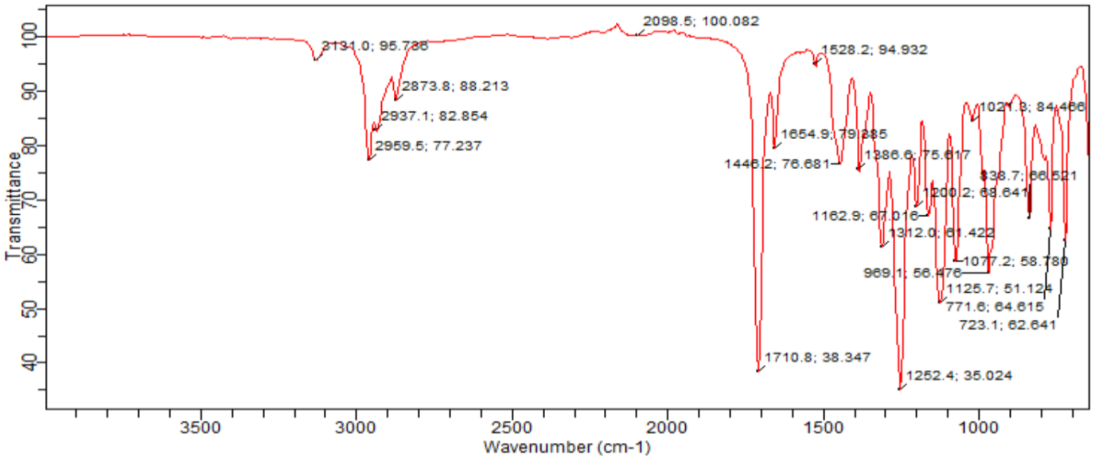

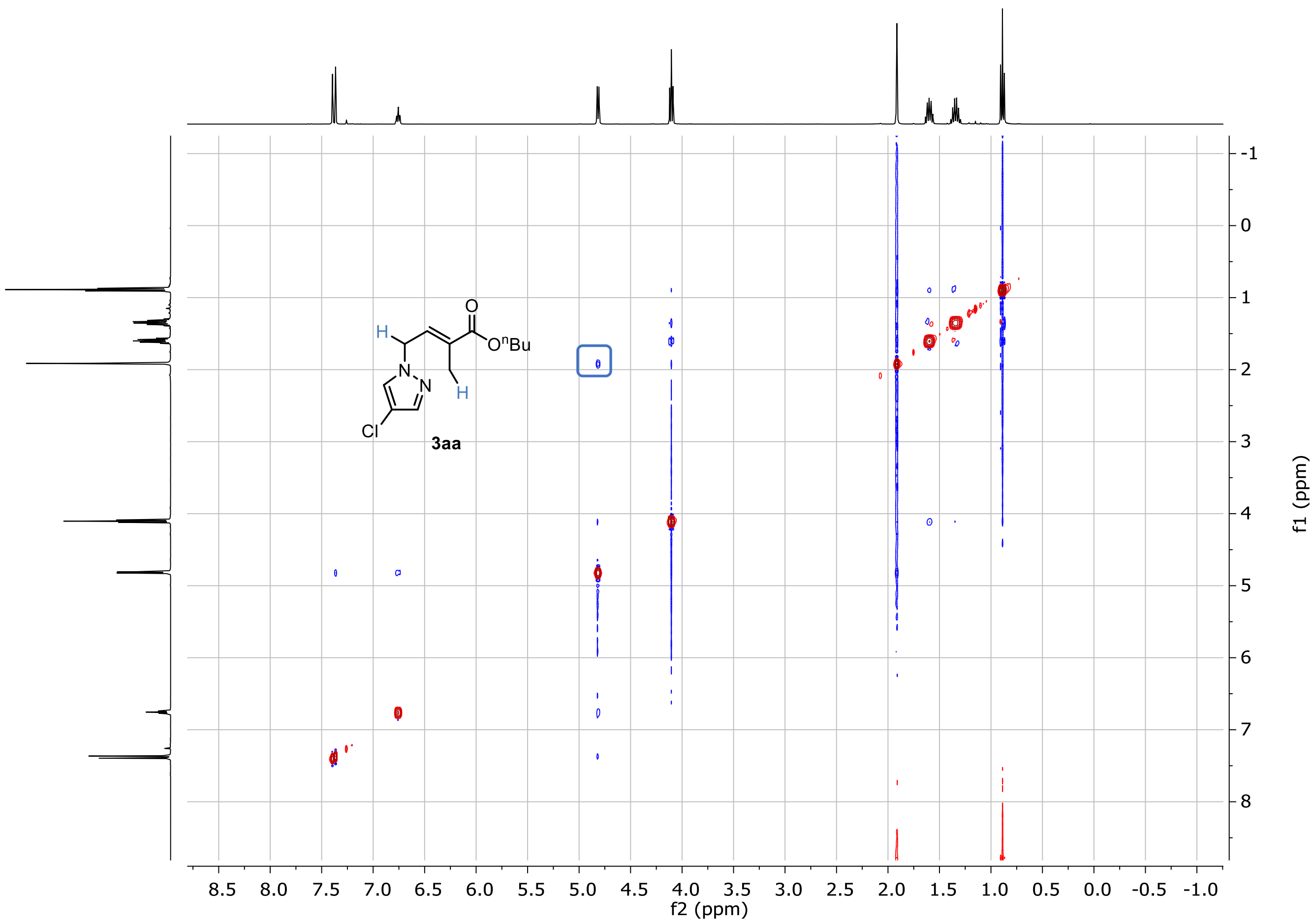
H
O
O^{n}Bu
N
N
H
Cl
3aa
8.5 8.0 7.5 7.0 6.5 6.0 5.5 5.0 4.5 4.0 3.5 3.0 2.5 2.0 1.5 1.0 0.5 0.0 -0.5 -1.0
f2 (ppm)
-1 0 1 2 3 4 5 6 7 8
f1 (ppm)

Diethyl 2-((1R,2R)-1,2-bis(4-chloro-1H-pyrazol-1-yl)hexyl)malonate (3ab) (^{1}H NMR: 300 MHz, ^{13}C NMR: 75 MHz, $CDCl_3$):

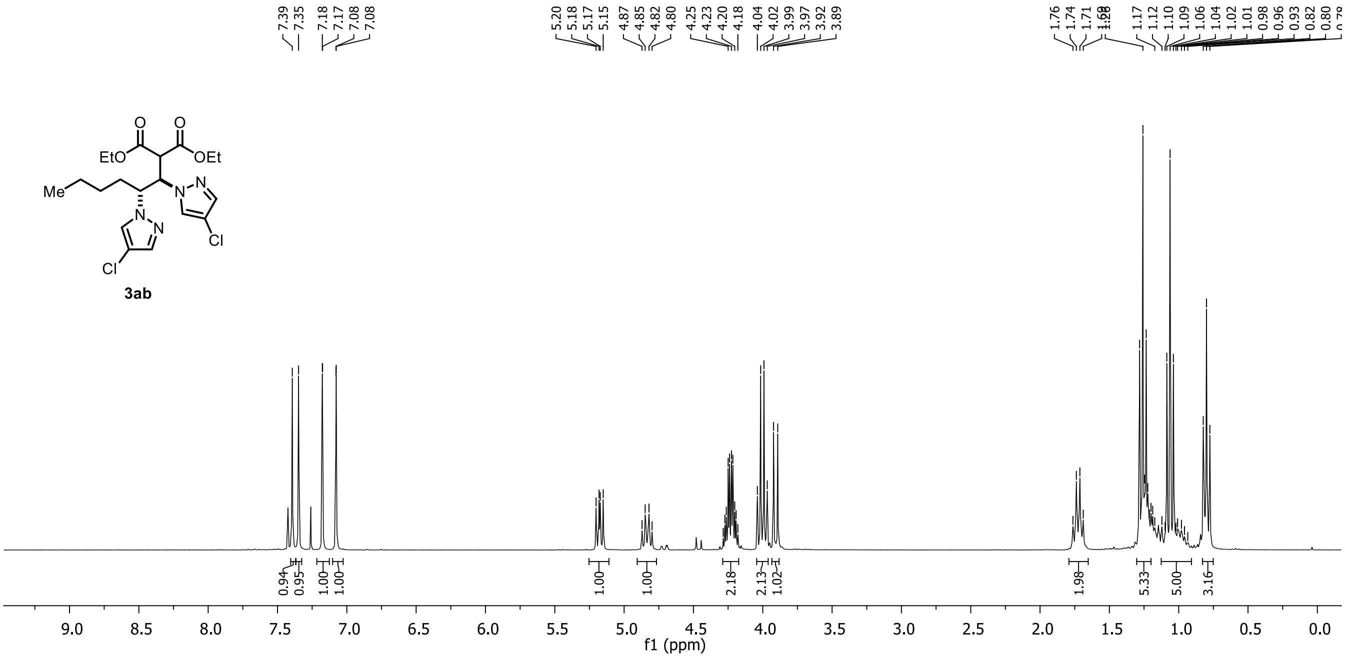

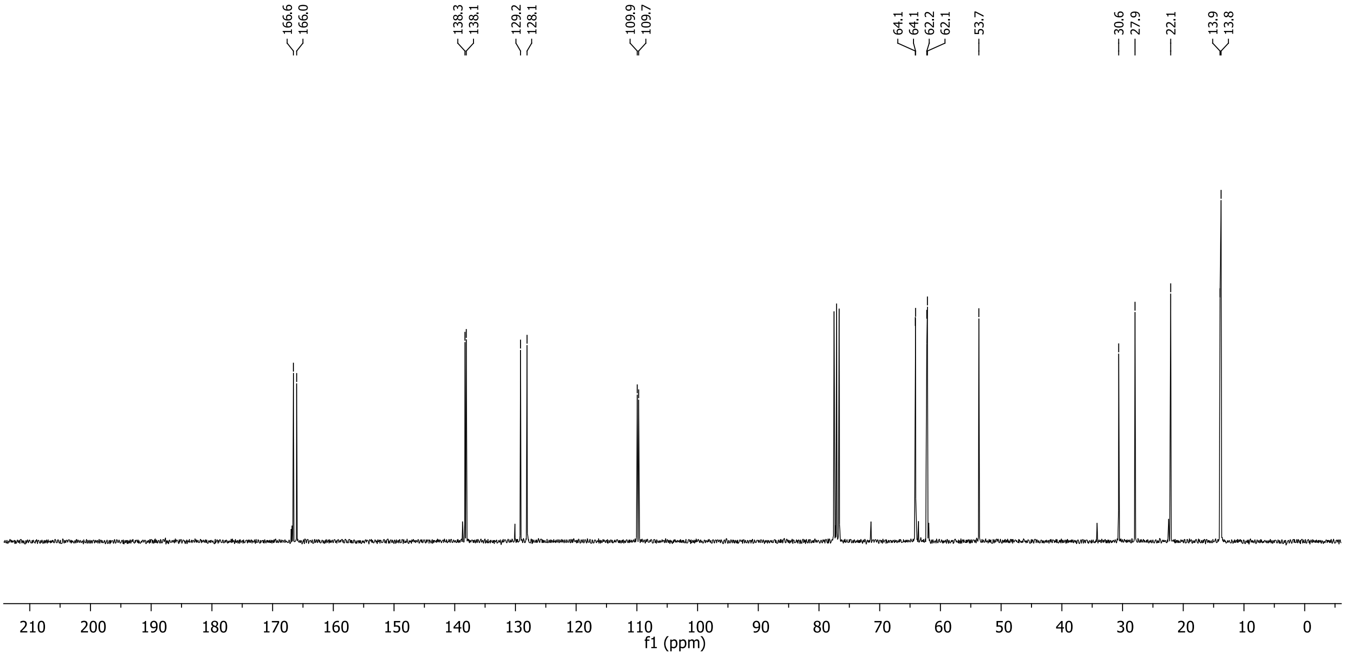

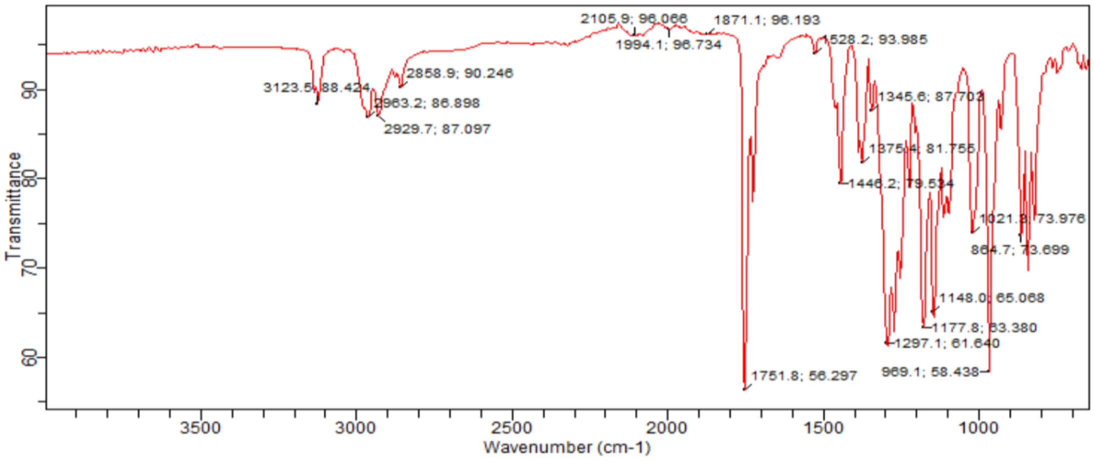

Ethyl (*E*)-4-(4-chloro-1H-pyrazol-1-yl)-4-methylpent-2-enoate (3ac) (^{1}H NMR: 300 MHz, ^{13}C NMR: 75 MHz, $CDCl_3$):

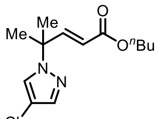

3ac

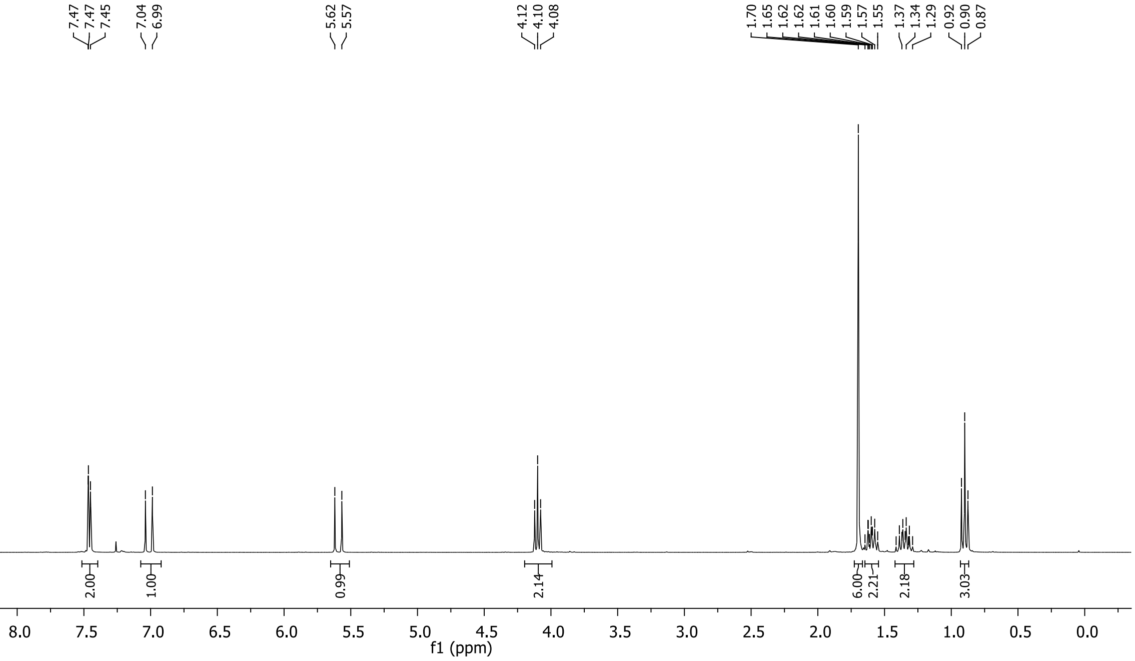

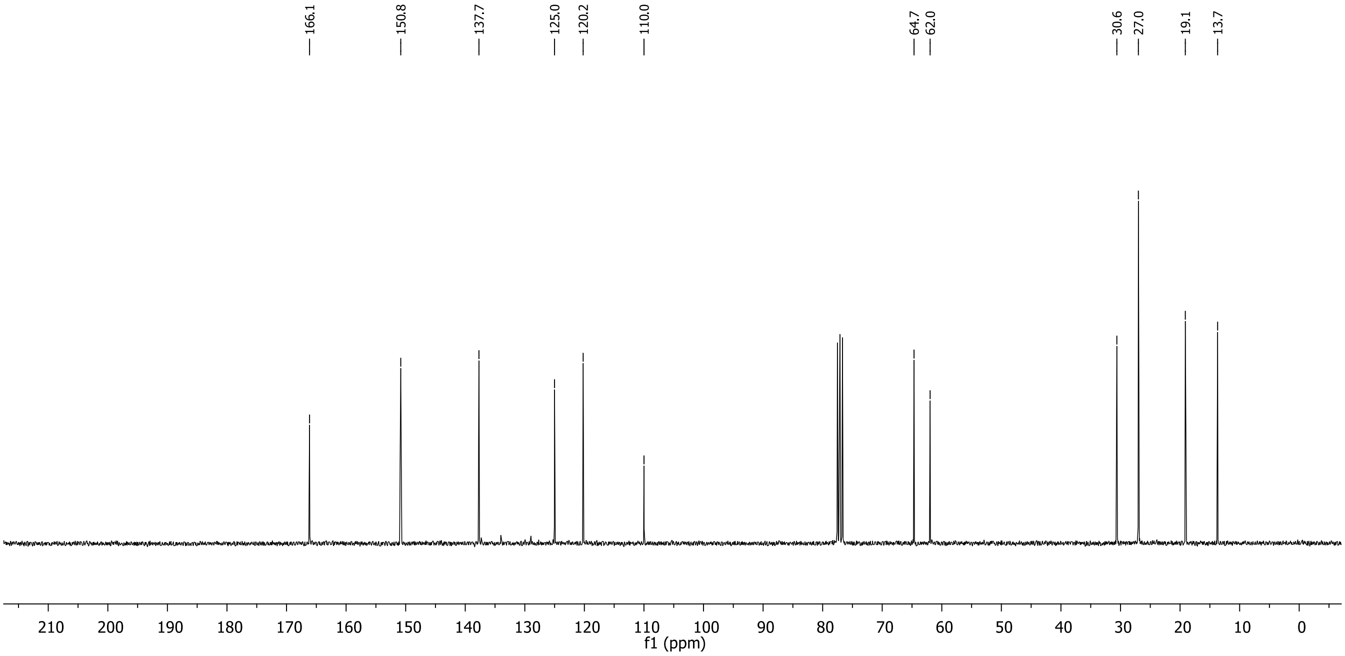

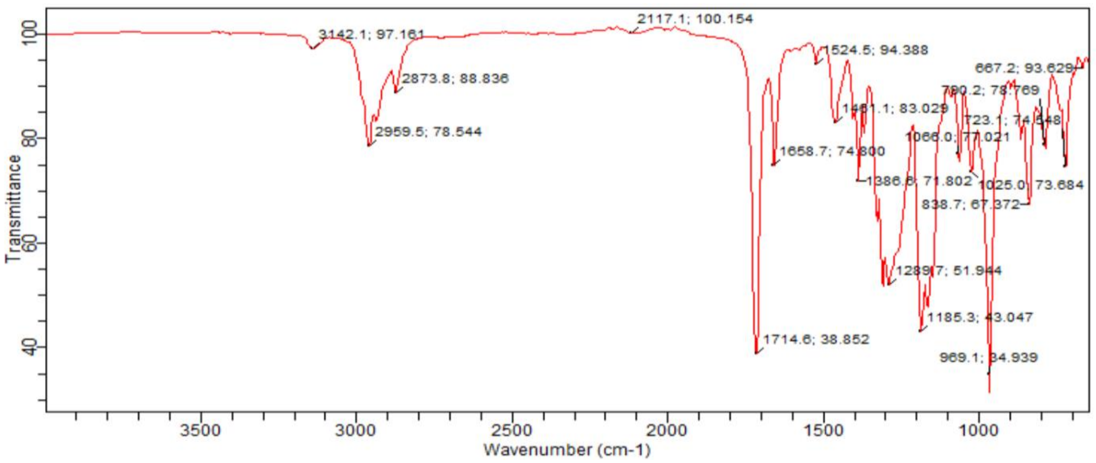

***tert*-Butyl (*E*)-4-(4-chloro-1H-pyrazol-1-yl)but-2-enoate (3ad) (^{1}H NMR: 300 MHz, ^{13}C NMR: 75 MHz, CDCl₃):**

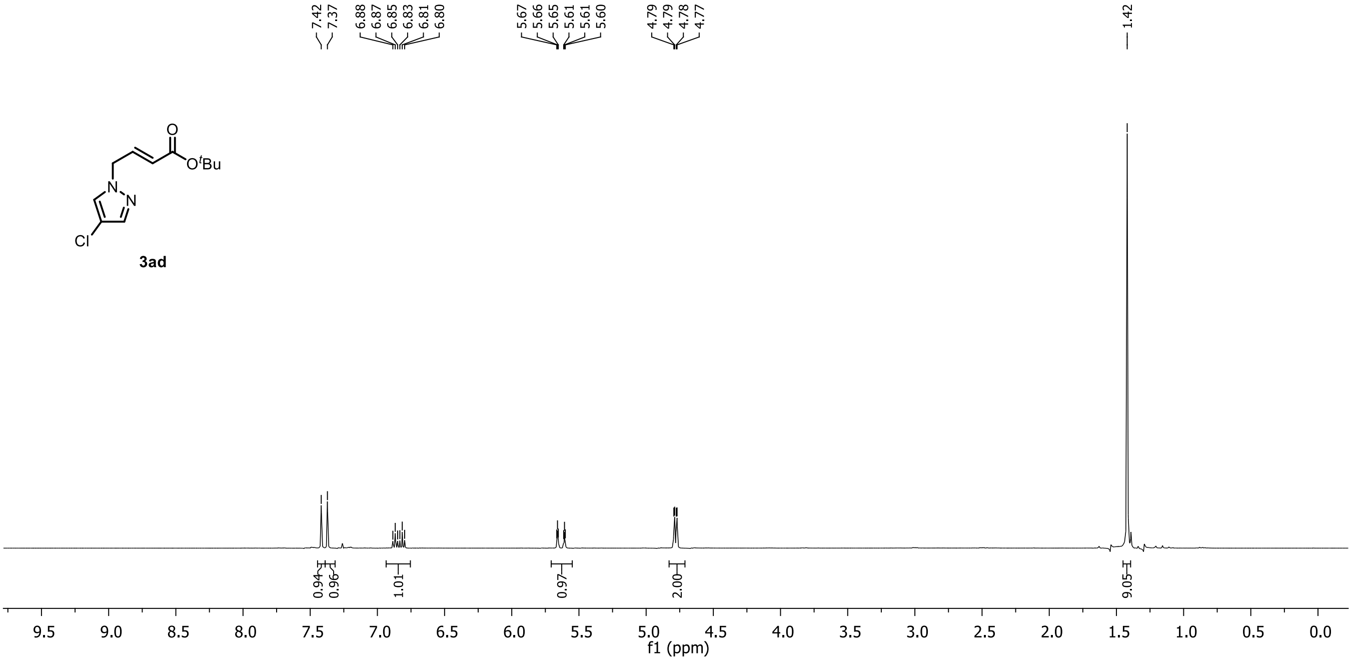

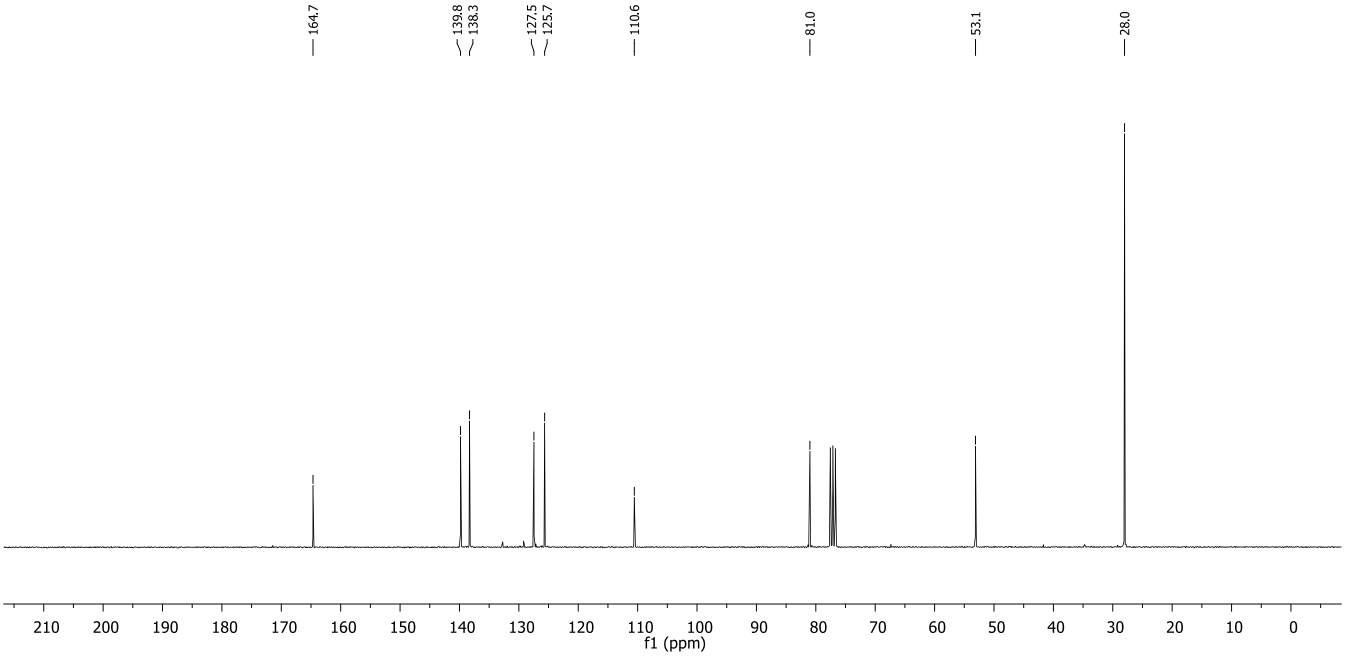

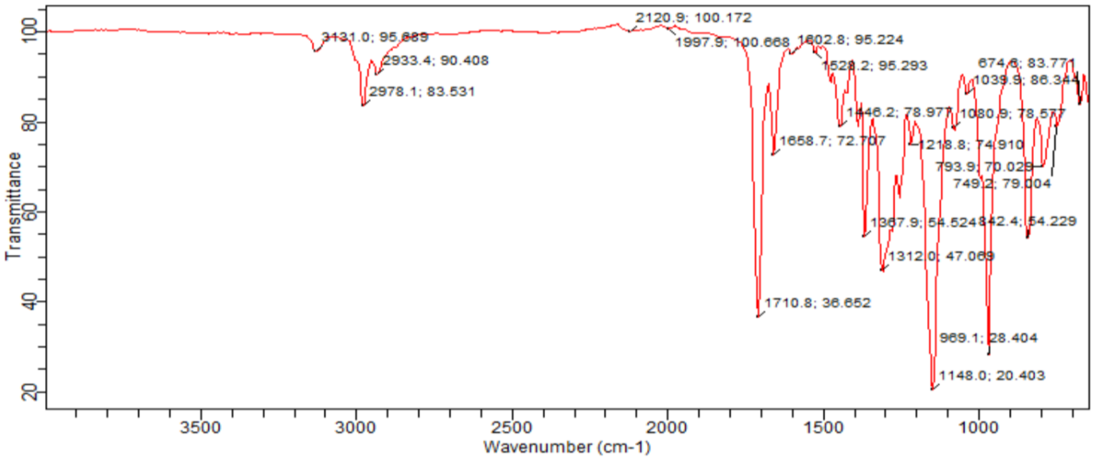

(*E*)-4-Chloro-1-(4-(phenylsulfonyl)but-3-en-2-yl)-1H-pyrazole (3ae) (^{1}H NMR: 300 MHz, ^{13}C NMR: 75 MHz, CDCl$_3$):

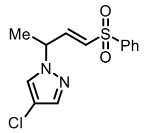

3ae

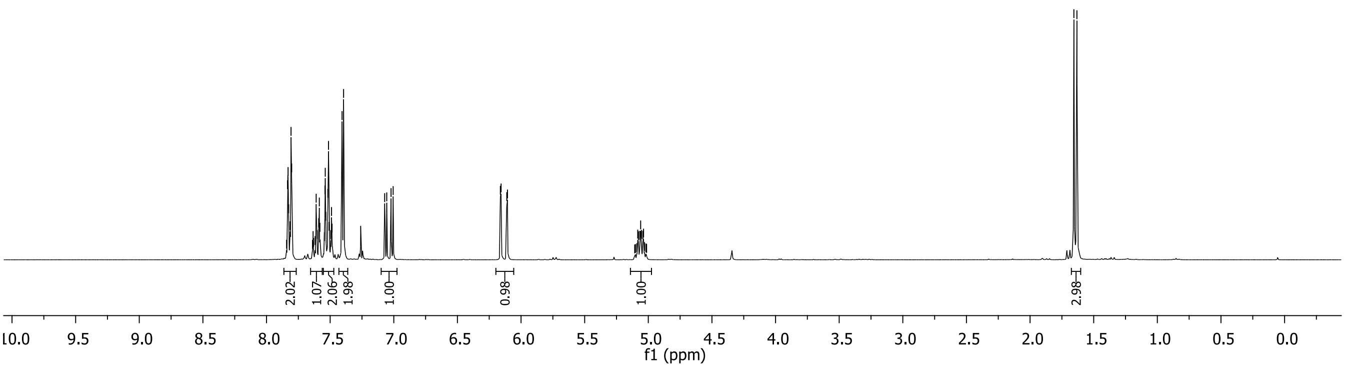

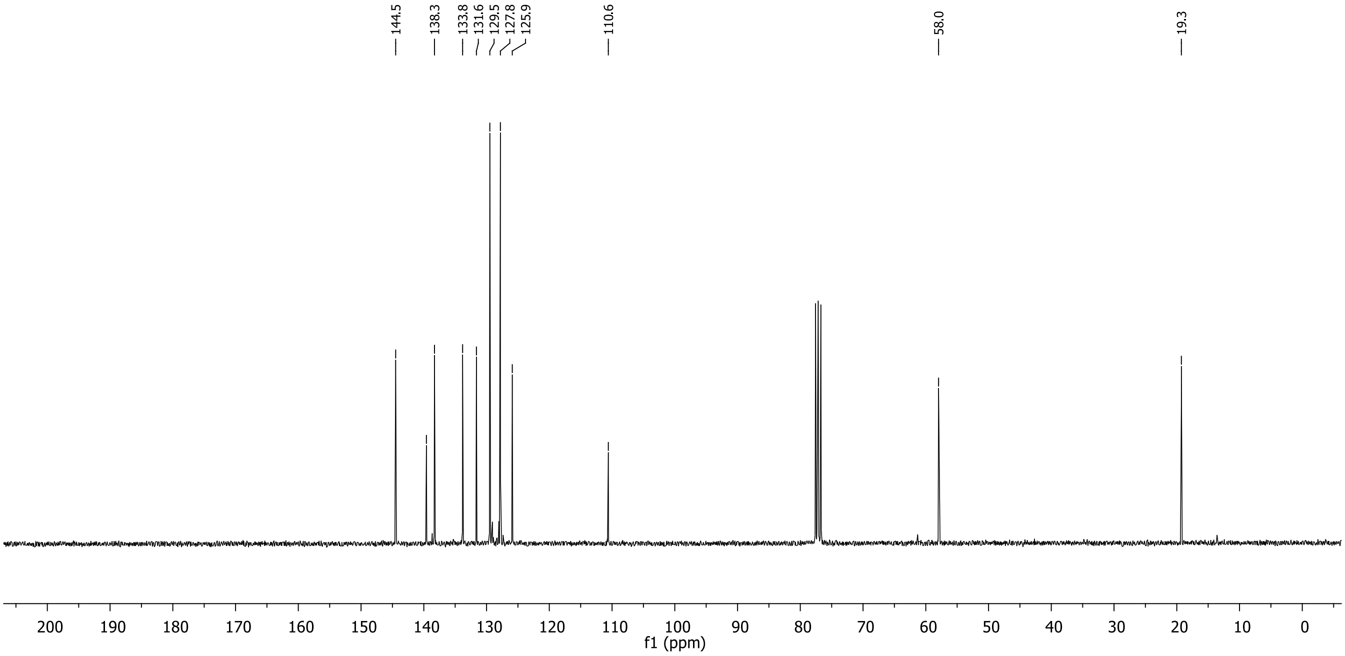

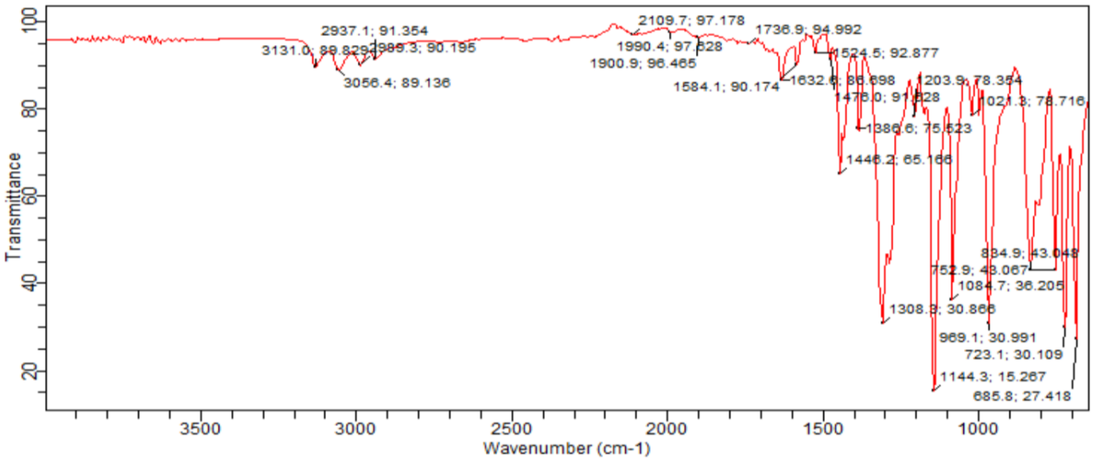

(*E*)-4-Chloro-1-(3-(phenylsulfonyl)allyl)-1H-pyrazole (3af) (^{1}H NMR: 300 MHz, ^{13}C NMR: 75 MHz, $CDCl_3$):

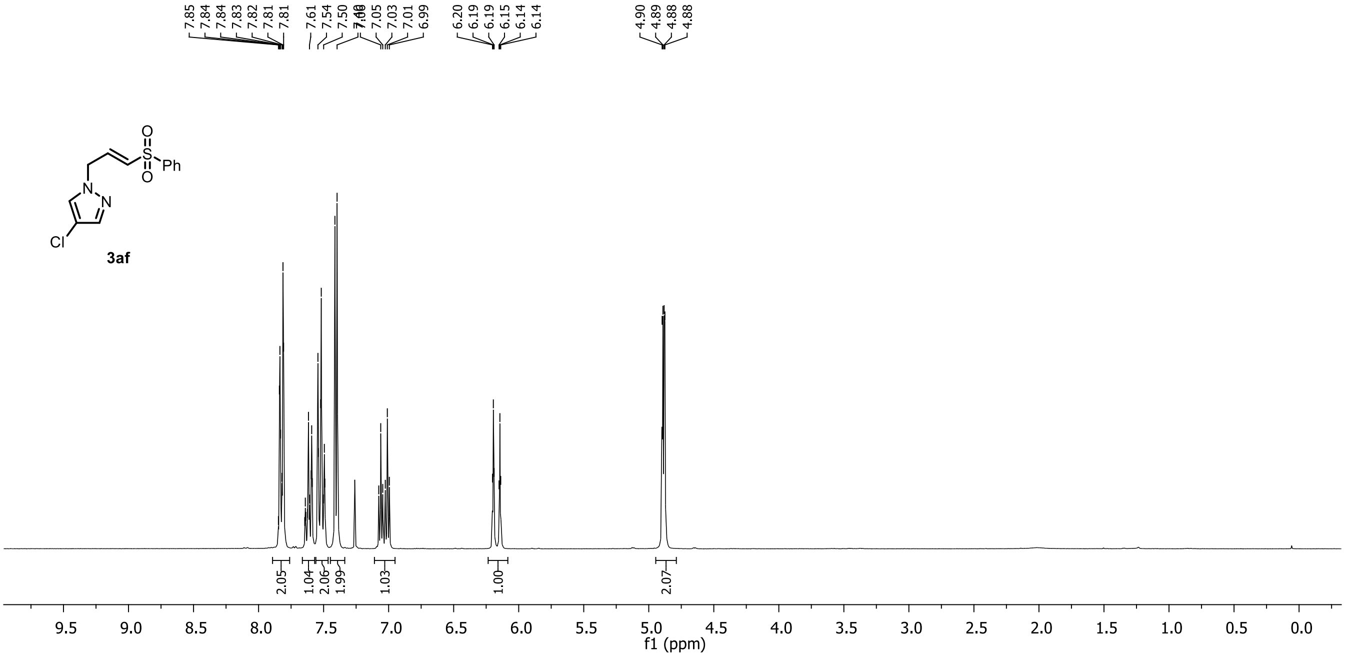

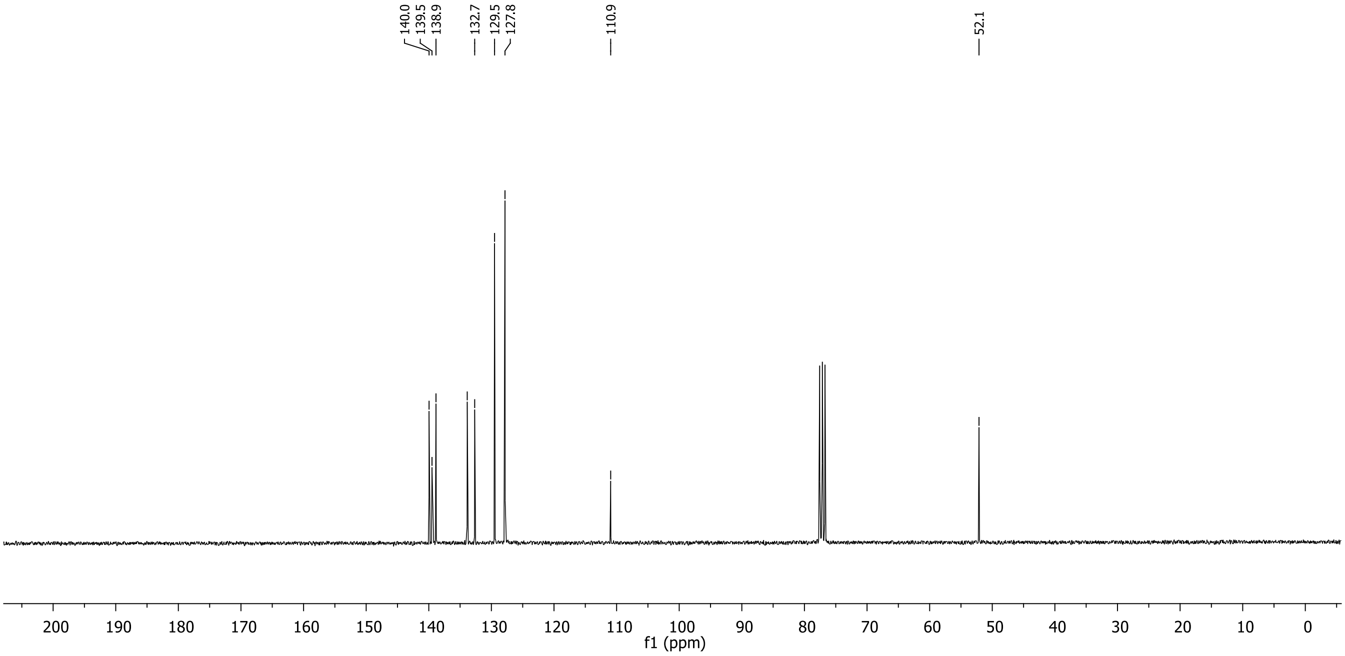

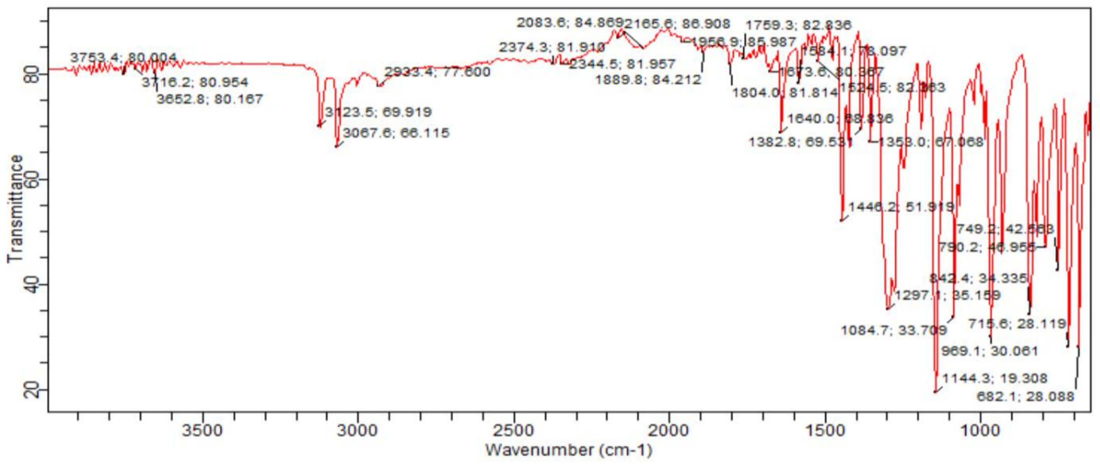

4-(4-Chloro-1H-pyrazol-1-yl)pent-2-enenitrile (3ag) (^{1}H NMR: 300 MHz, ^{13}C NMR: 75 MHz, $CDCl_3$):

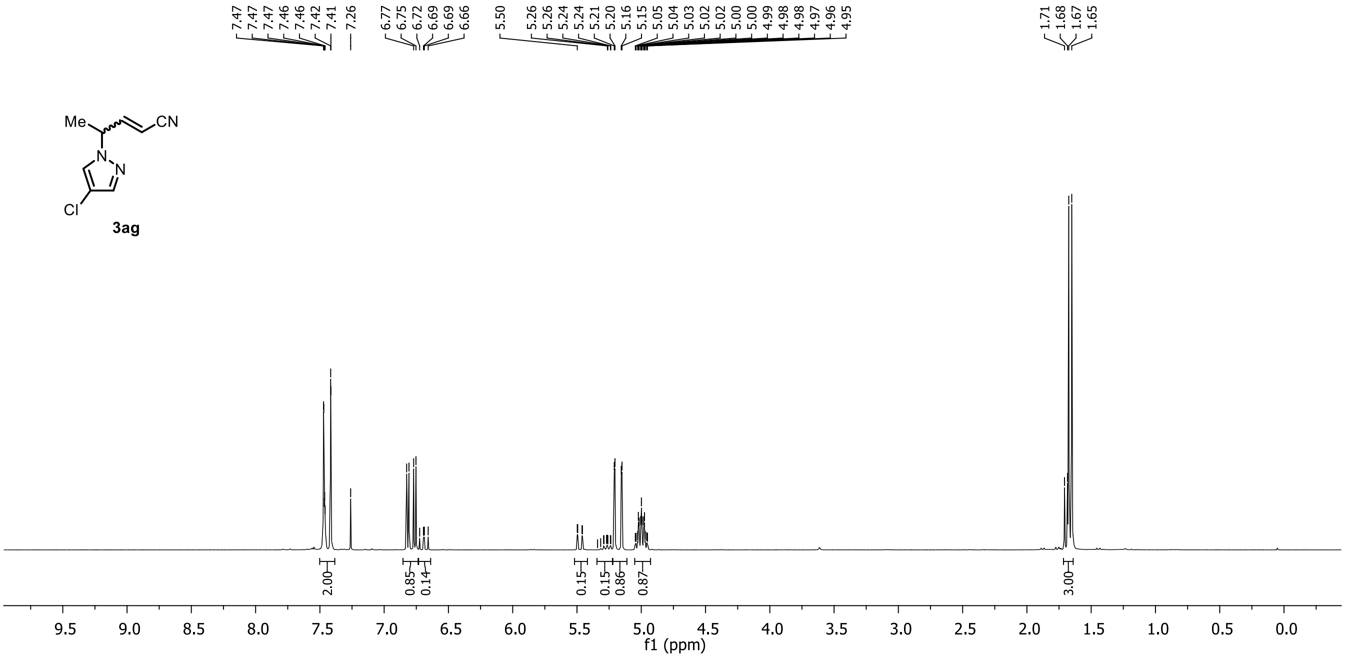

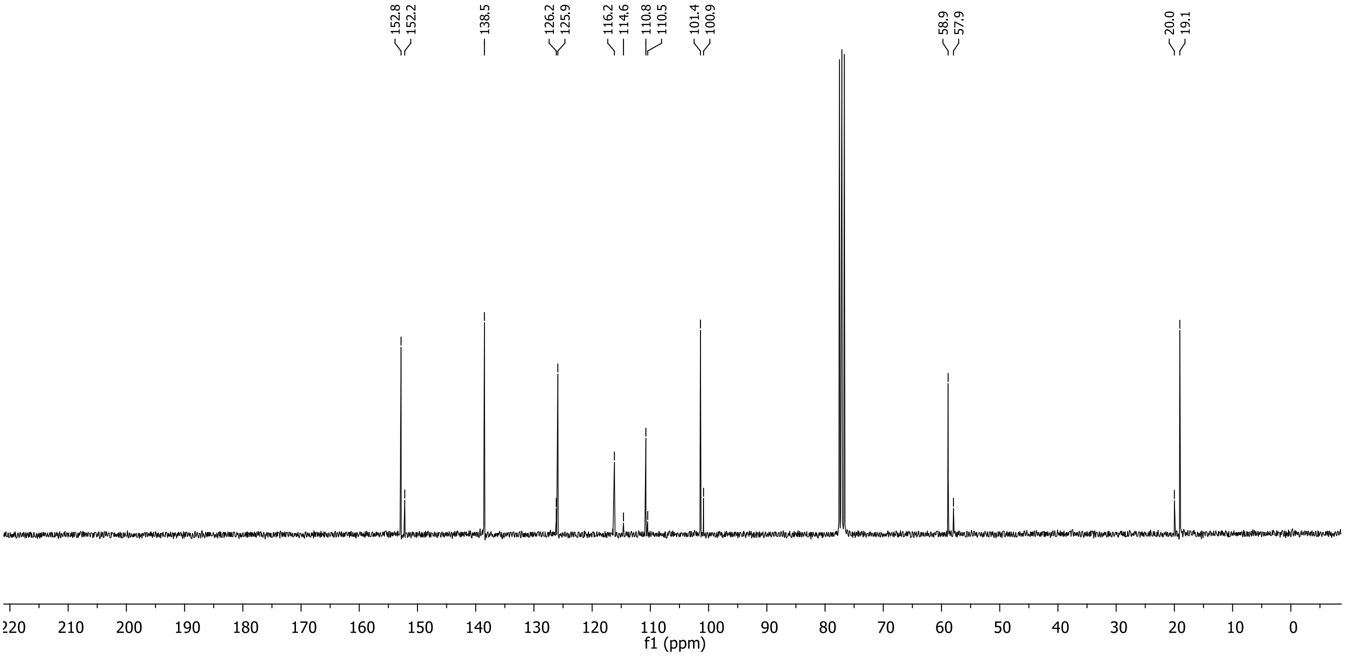

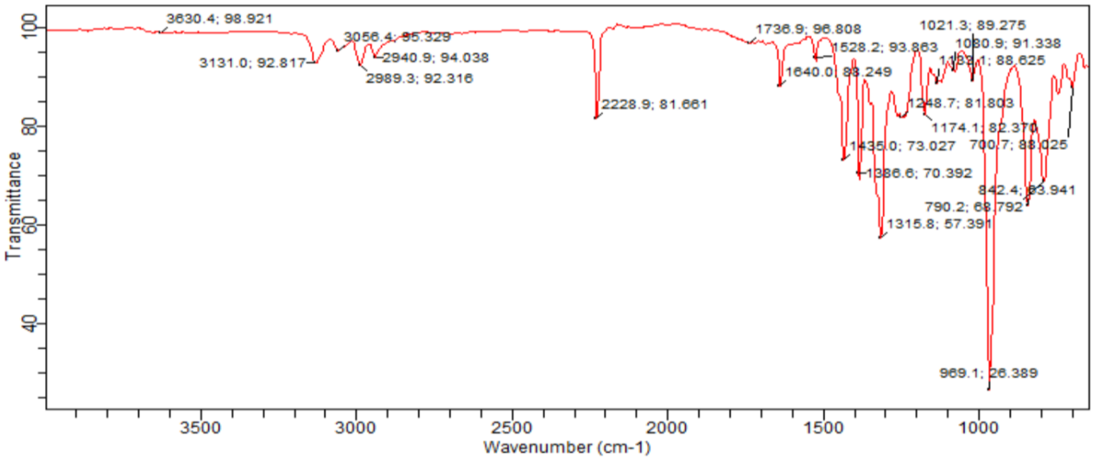

4-(4-Chloro-1H-pyrazol-1-yl)but-2-enenitrile (3ah) (^{1}H NMR: 300 MHz, ^{13}C NMR: 75 MHz, $CDCl_3$):

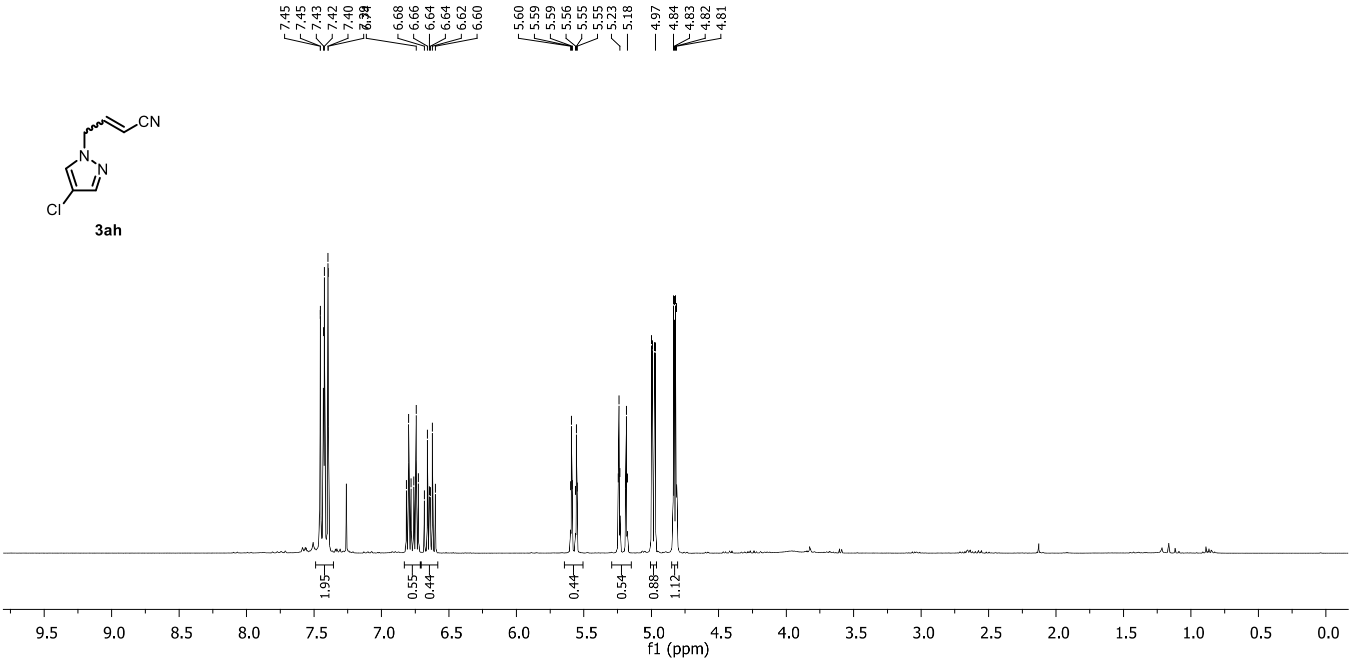

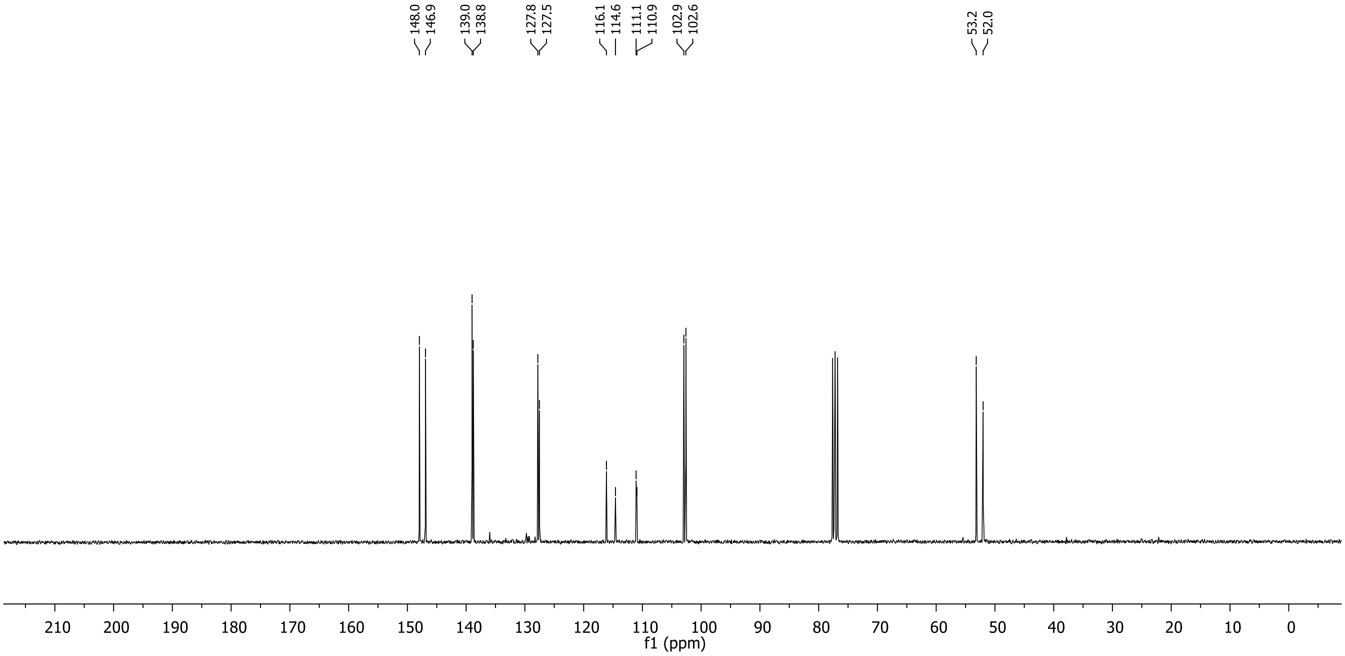

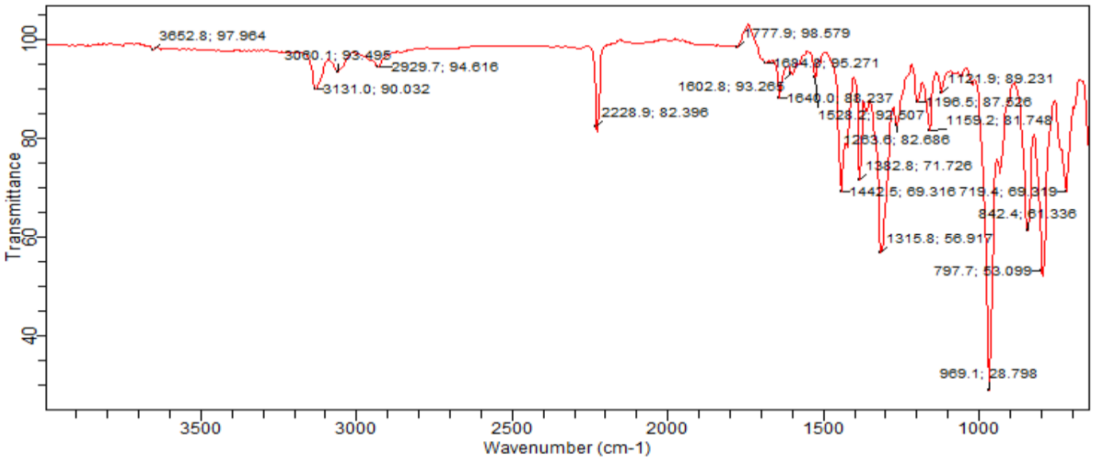

(*Z*)-4-(4-Chloro-1H-pyrazol-1-yl)-2-methylbut-2-enenitrile (3ai) (^{1}H NMR: 300 MHz, ^{13}C NMR: 75 MHz, 2D NOESY NMR: 400 MHz, $CDCl_3$):

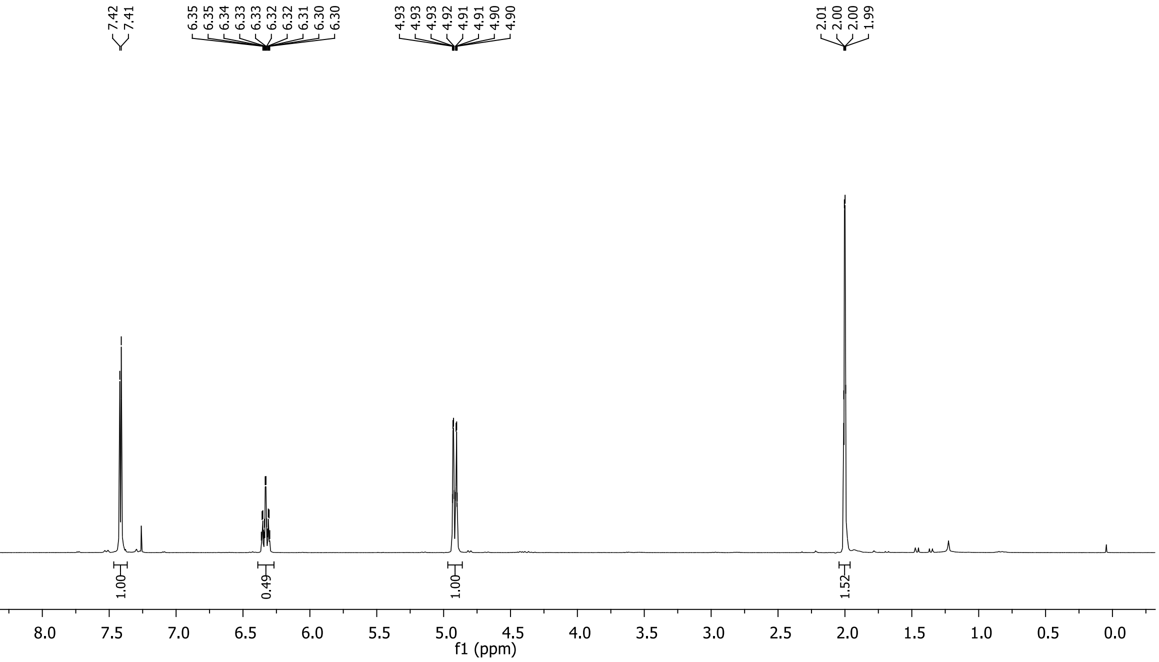

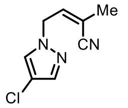

3ai

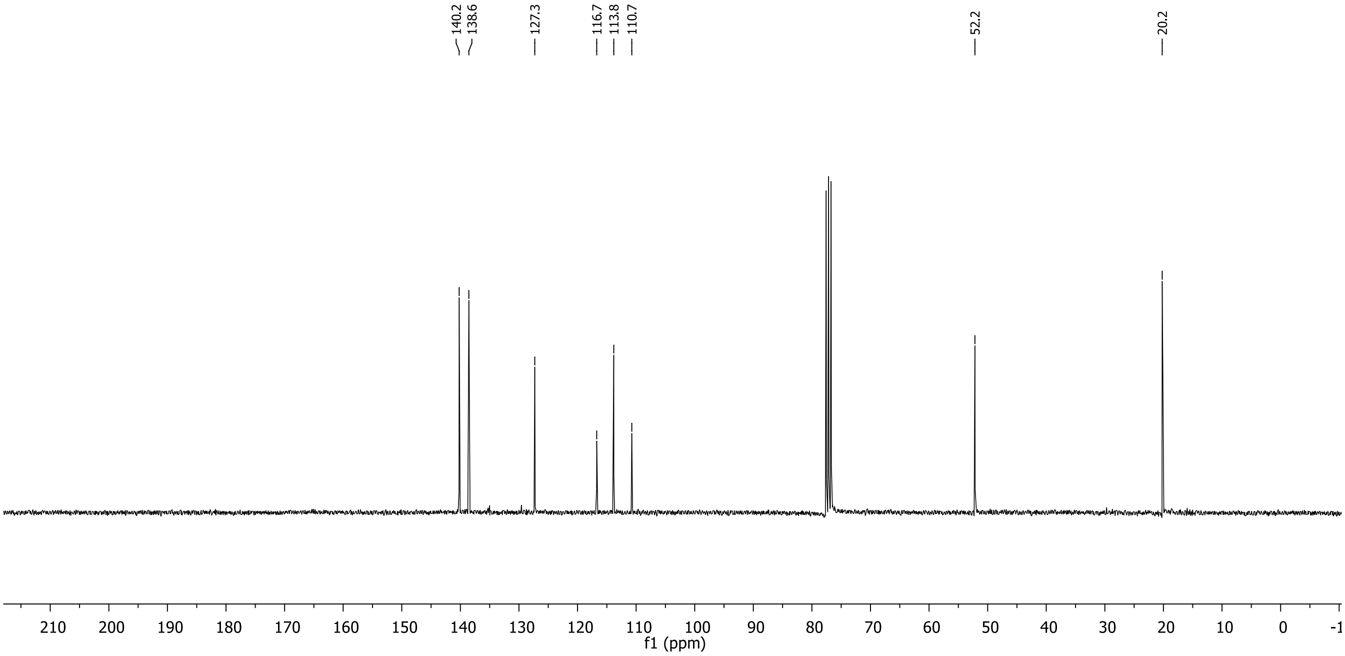

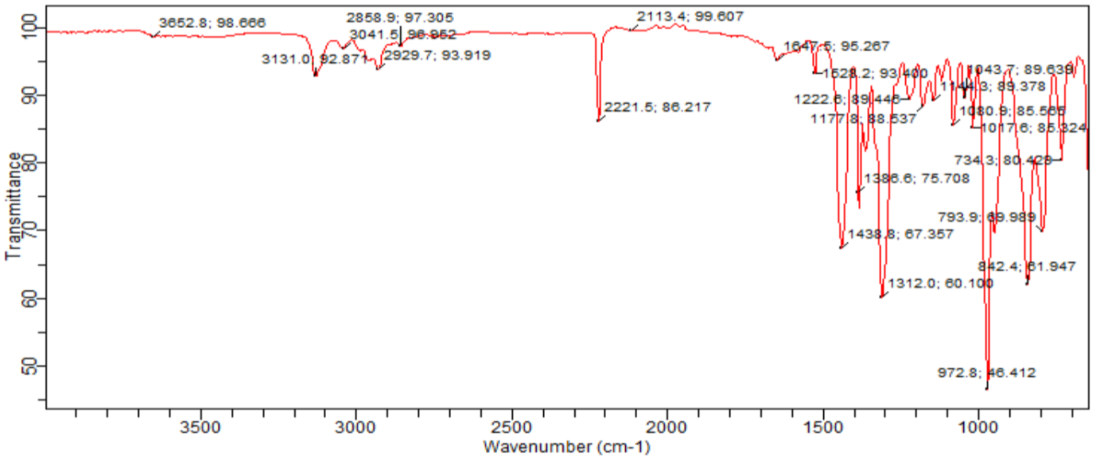

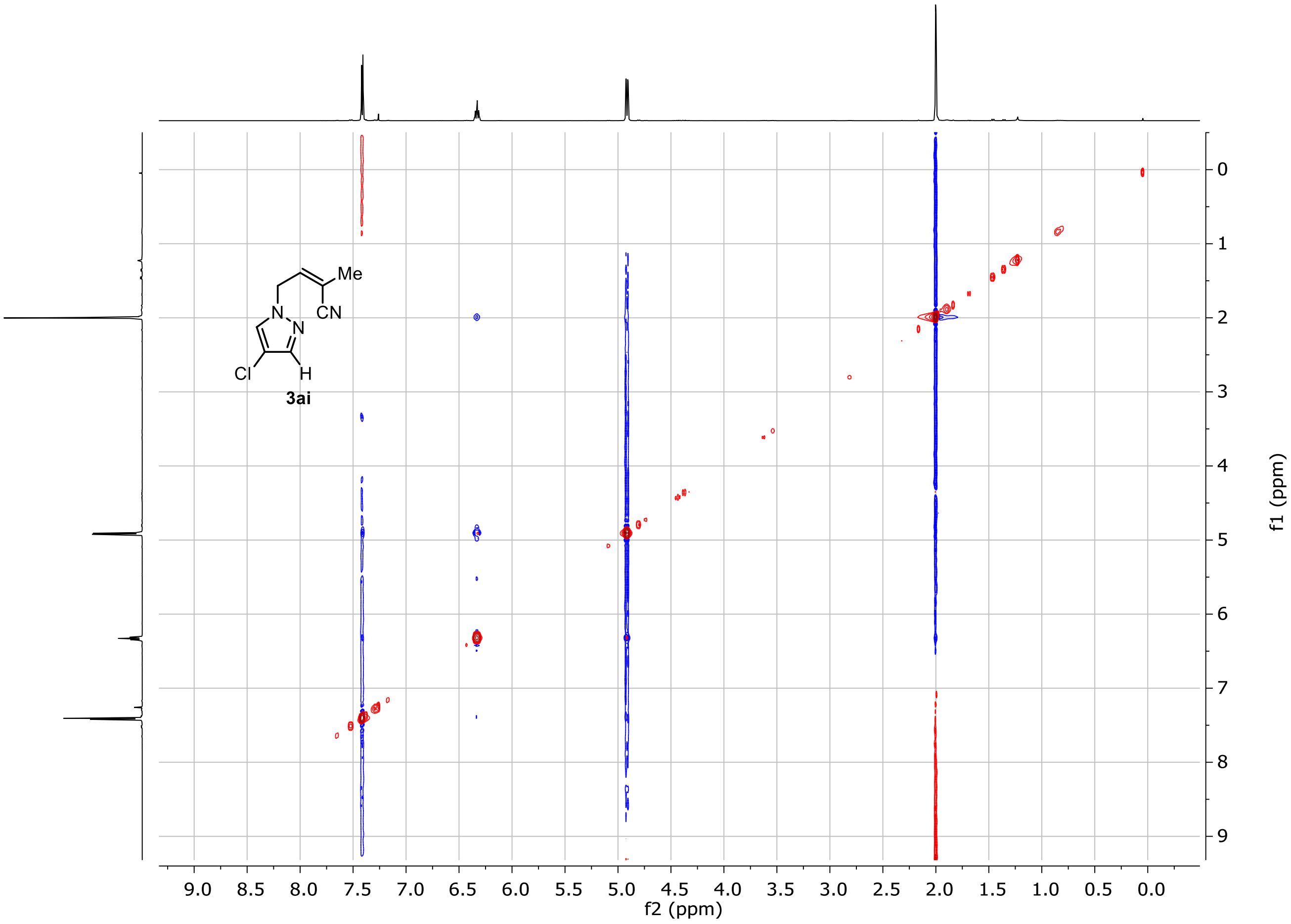

Me
CN
N
N
Cl
H
3ai
f1 (ppm)
f2 (ppm)
9.0 8.5 8.0 7.5 7.0 6.5 6.0 5.5 5.0 4.5 4.0 3.5 3.0 2.5 2.0 1.5 1.0 0.5 0.0
0 1 2 3 4 5 6 7 8 9

(*E*)-(3-(4-Chloro-1H-pyrazol-1-yl)prop-1-en-1-yl)diphenylphosphine oxide (3aj) (^{1}H NMR: 300 MHz, ^{13}C NMR: 75 MHz, ^{31}P NMR 162 MHz, CDCl$_3$):

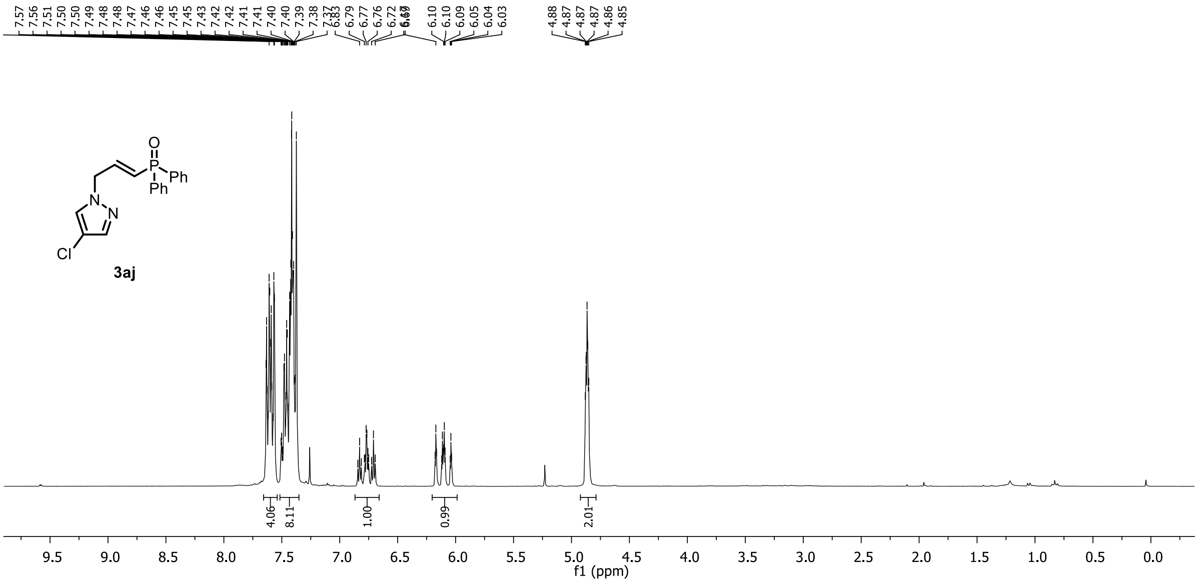

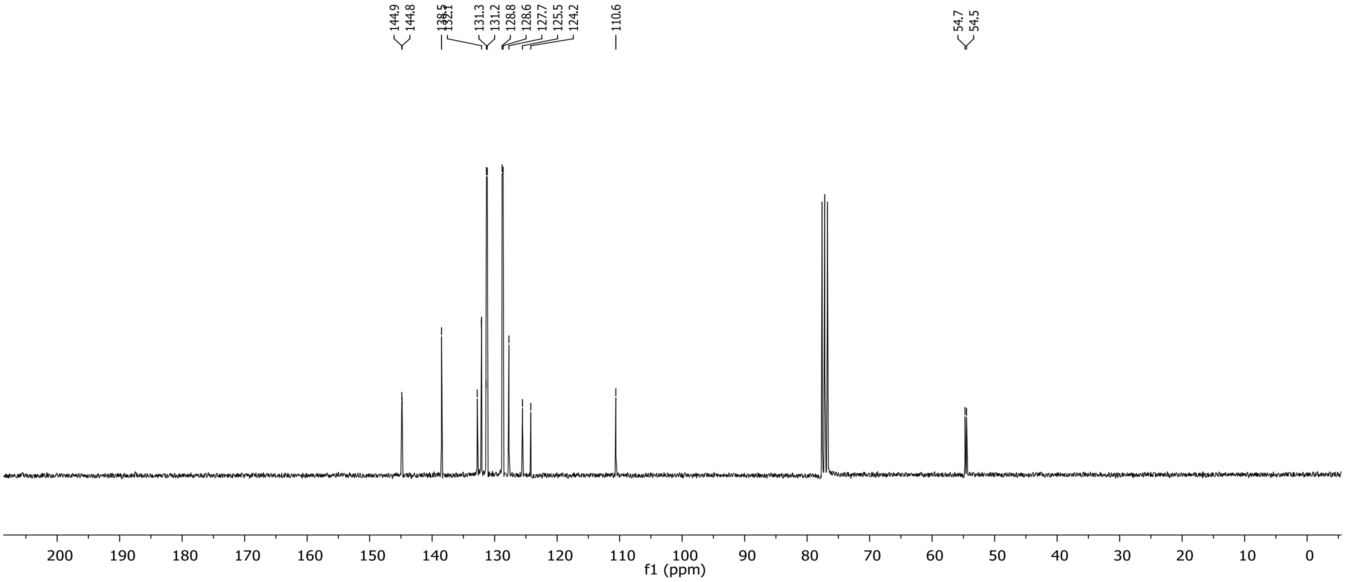

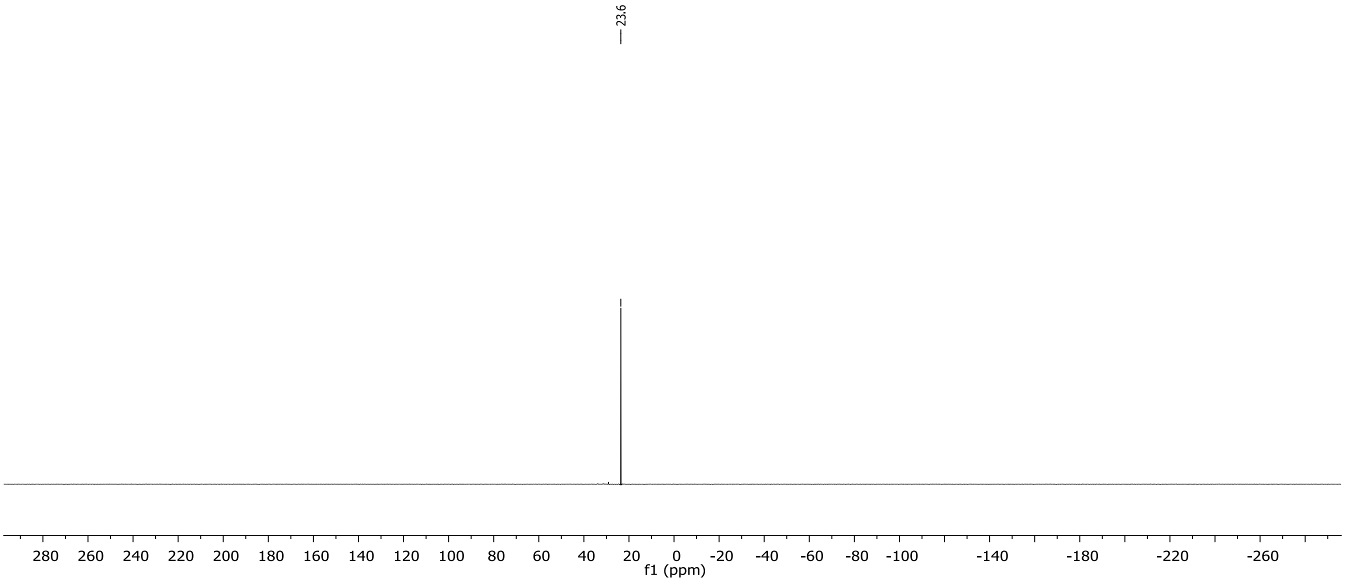

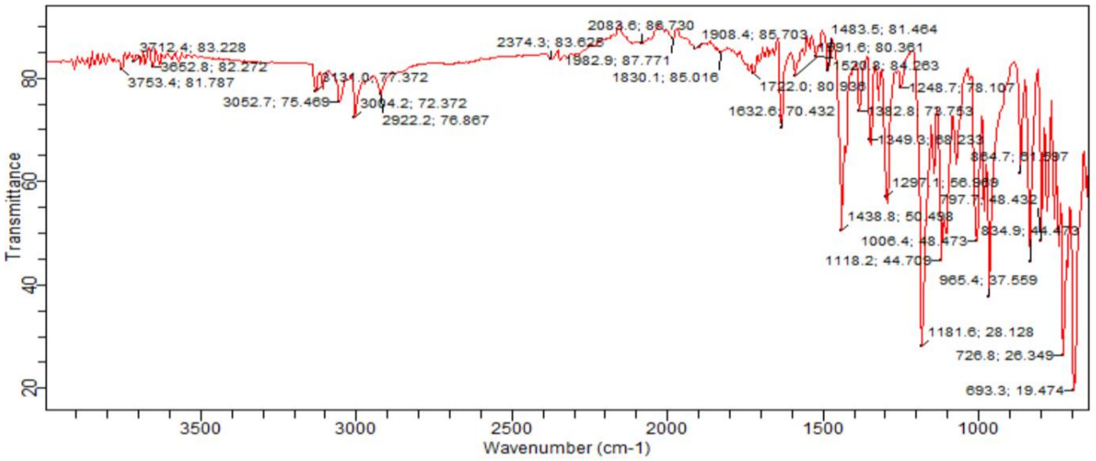
Transmittance
Wavenumber (cm-1)
3712.4; 83.228
3652.8; 82.272
3753.4; 81.787
3131.0; 77.372
3052.7; 75.469
3004.2; 72.372
2922.2; 76.867
2374.3; 83.625
2083.6; 88.730
1982.9; 87.771
1908.4; 85.703
1830.1; 85.016
1722.0; 80.936
1632.6; 70.432
1483.5; 81.464
1520.8; 84.263
1248.7; 78.107
1349.3; 68.233
1297.1; 56.969
1438.8; 50.498
1006.4; 48.473
1118.2; 44.709
965.4; 37.559
797.7; 48.432
834.9; 44.473
1181.6; 28.128
726.8; 26.349
693.3; 19.474

(*E*)-4-Chloro-1-(tetradec-8-en-7-yl)-1H-pyrazole and (*Z*)-4-chloro-1-(tetradec-7-en-7-yl)-1H-pyrazole (3ak) (^{1}H NMR: 300 MHz, ^{13}C NMR: 75 MHz, $CDCl_3$):

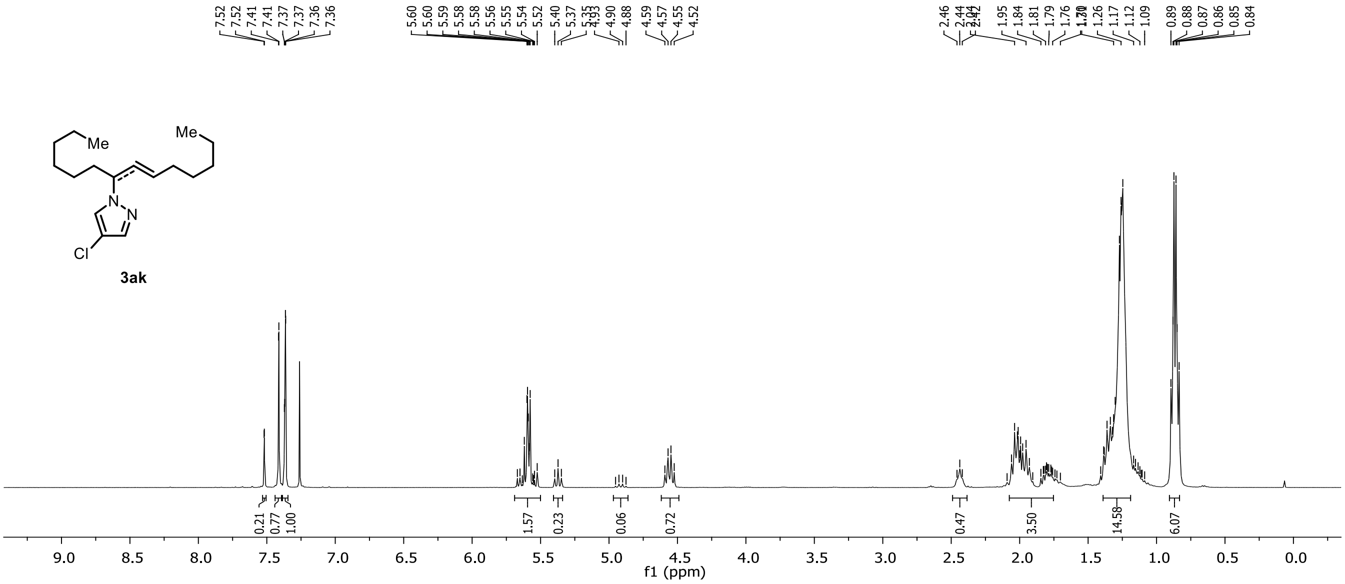

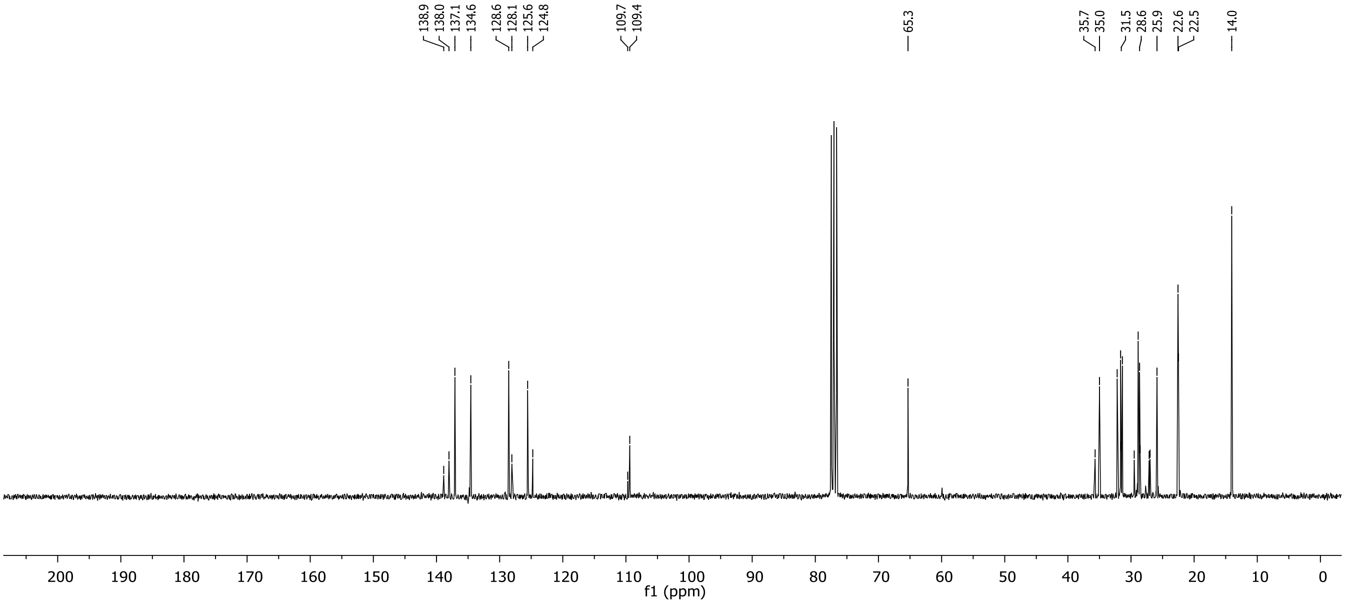

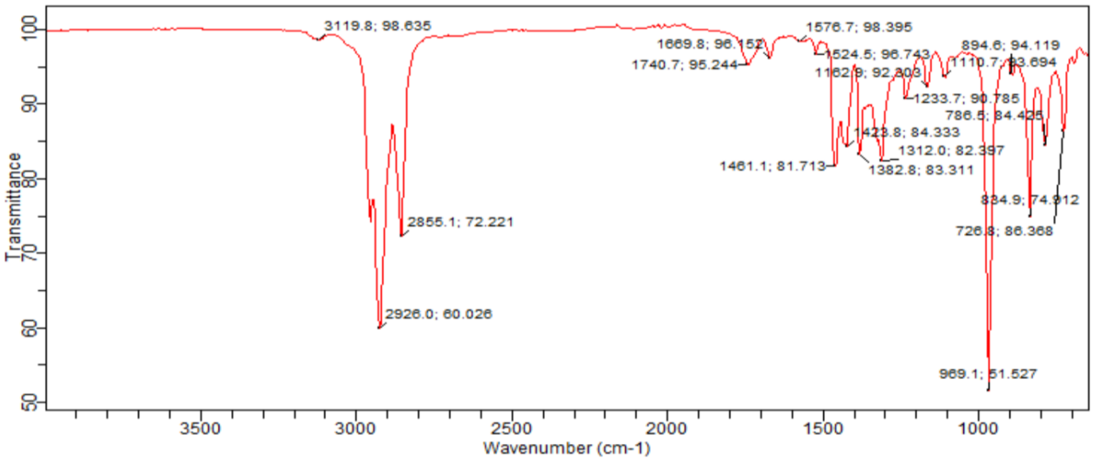

(E)-1-(Tetradec-8-en-7-yl)-1H-benzo[d][1,2,3]triazole (3al) (^{1}H NMR: 300 MHz, ^{13}C NMR: 75 MHz, CDCl$_3$):

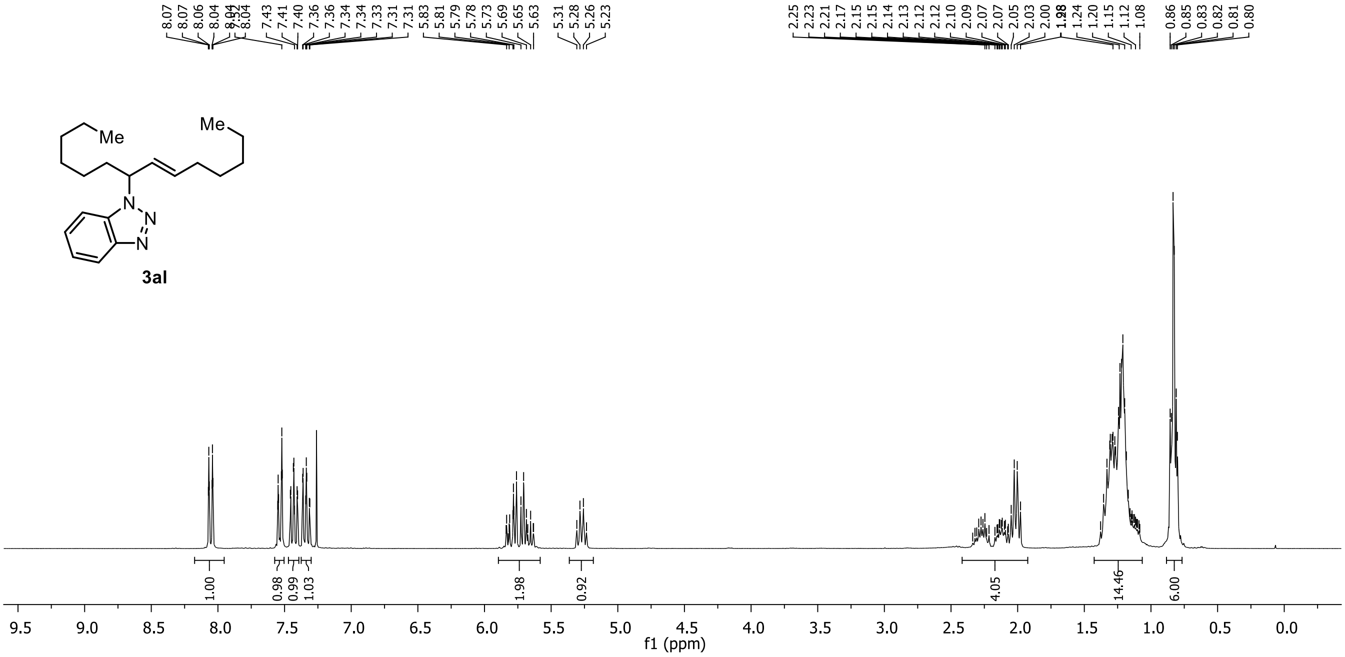

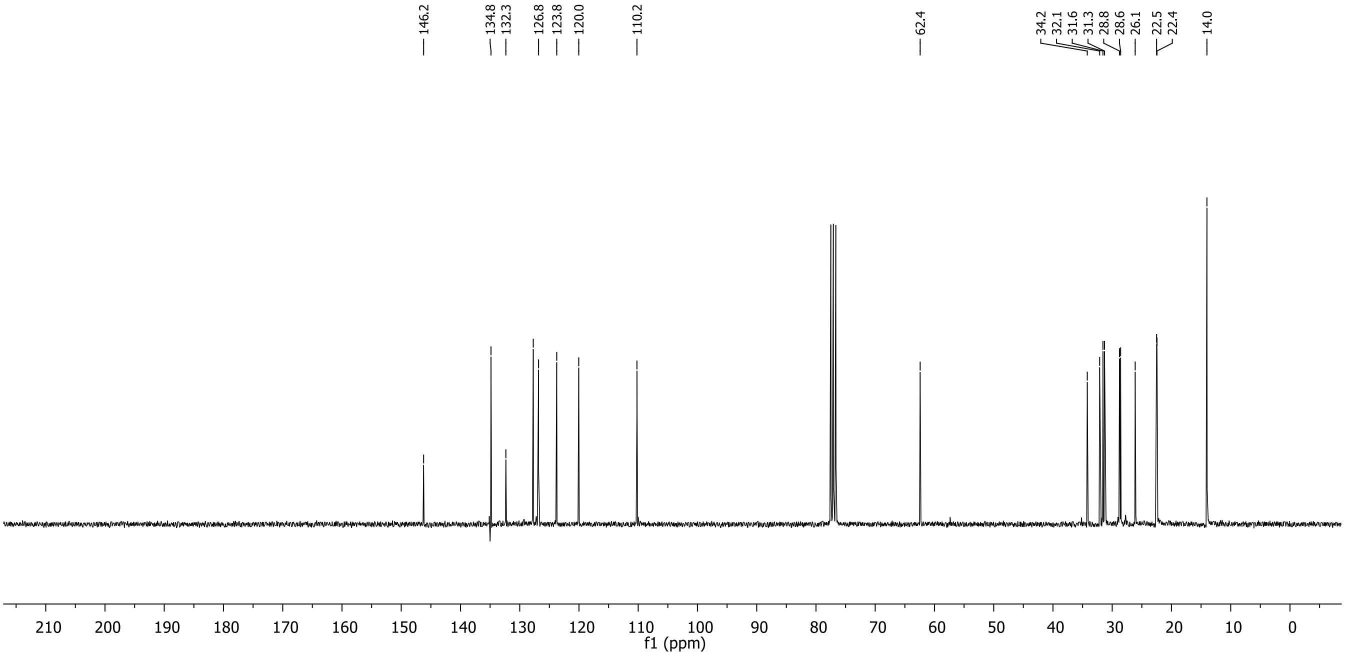

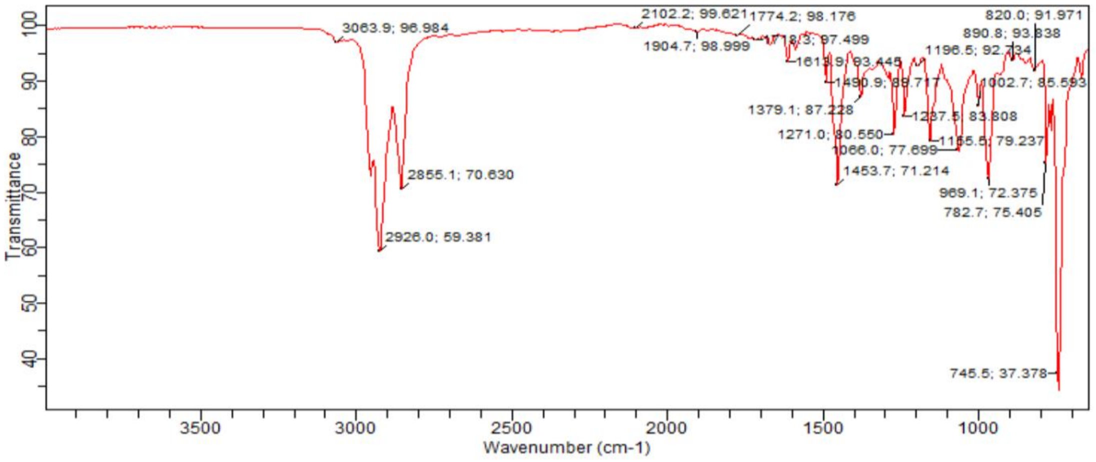

(*Z*)-1-(Tetradec-7-en-7-yl)-1H-benzo[d][1,2,3]triazole (3al’) (^{1}H NMR: 300 MHz, ^{13}C NMR: 75 MHz, $CDCl_3$):

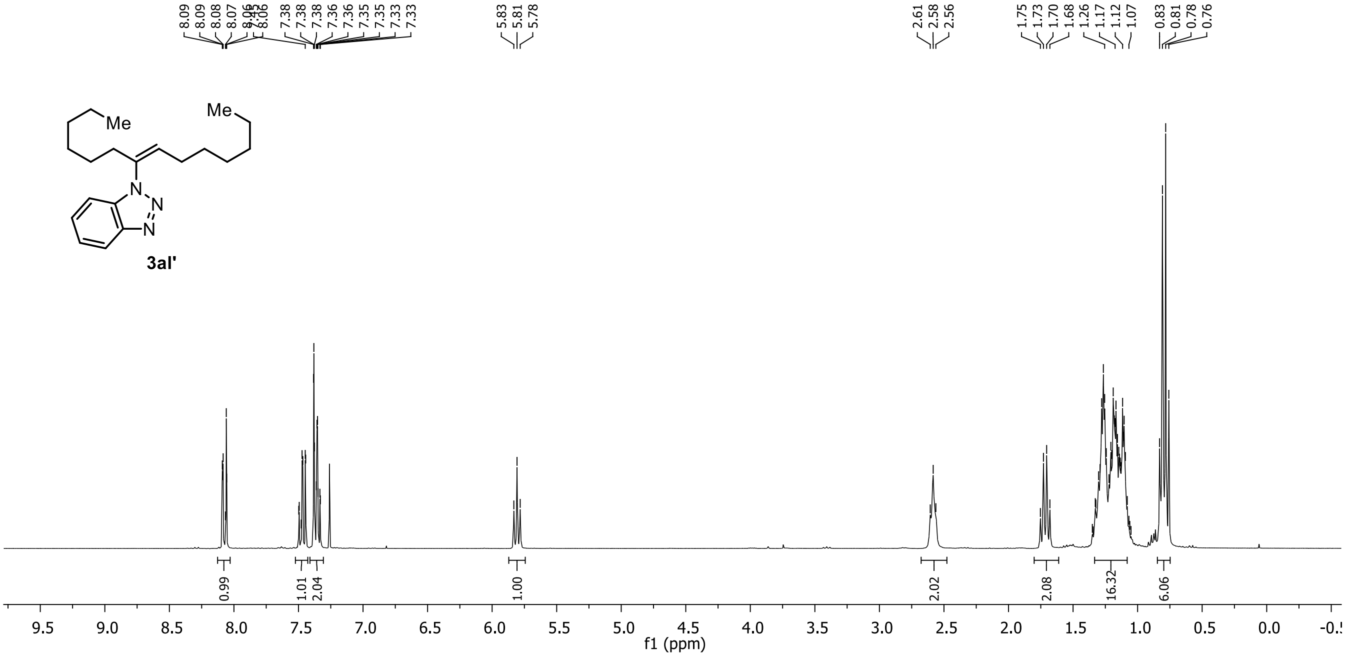

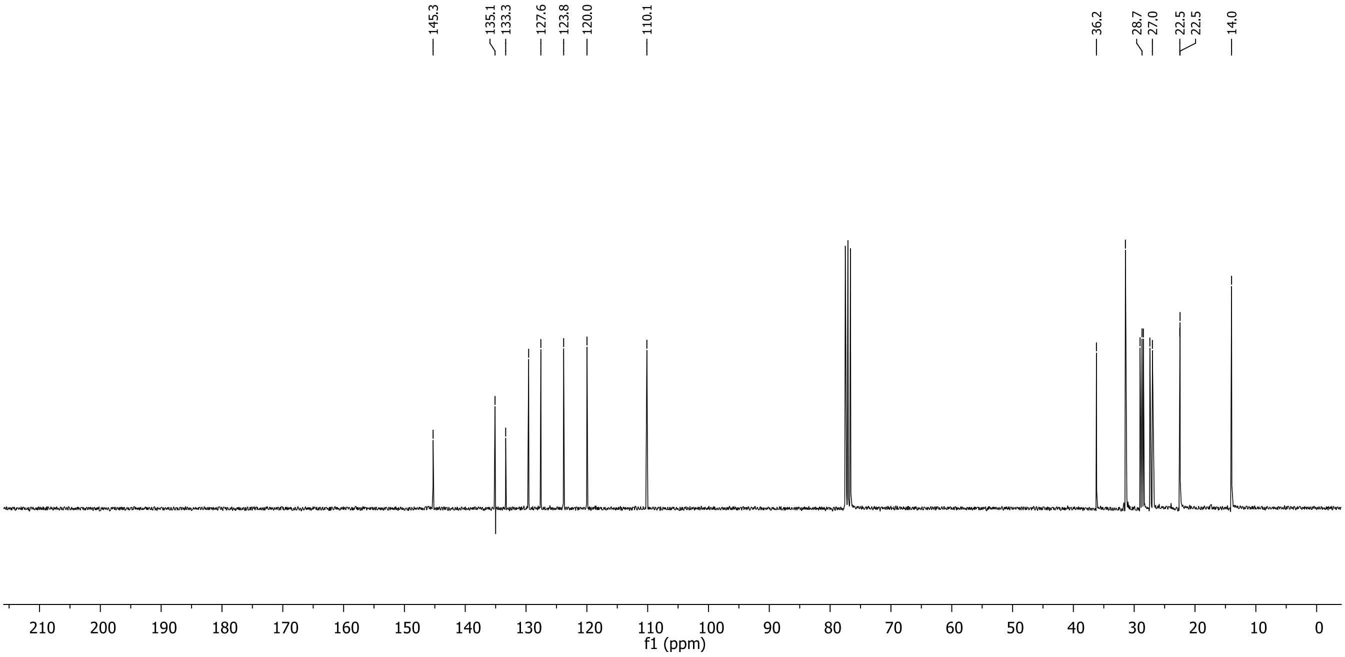

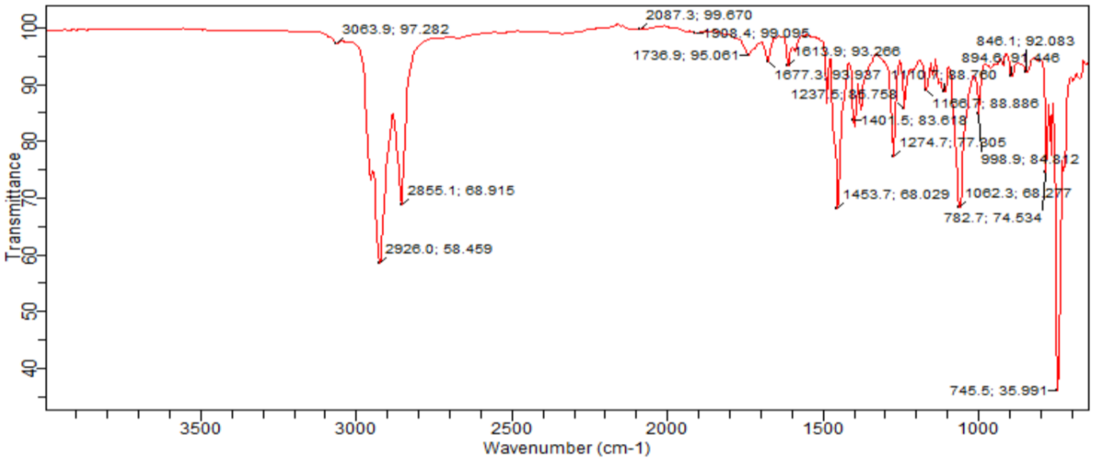

(*E*)-*N*-(Dec-6-en-5-yl)-N,4-dimethylbenzenesolfonamide (3am) (^{1}H NMR: 400 MHz, ^{13}C NMR: 101 MHz, $CDCl_3$):

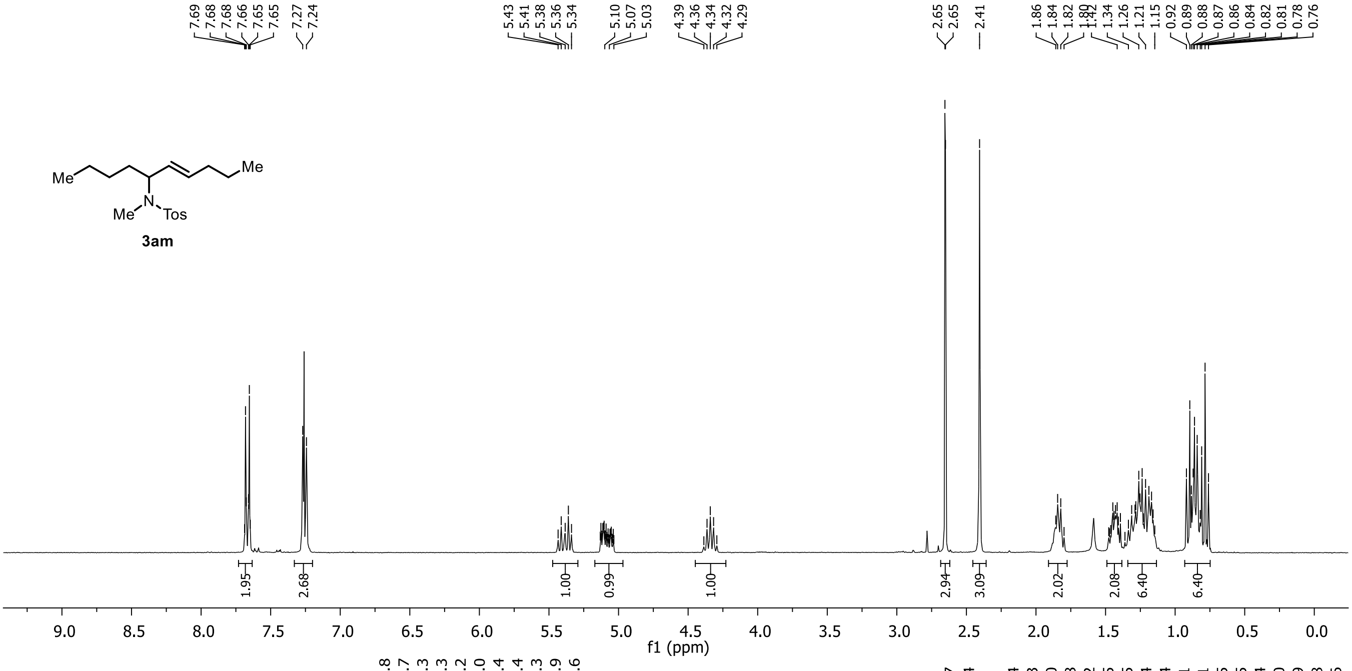

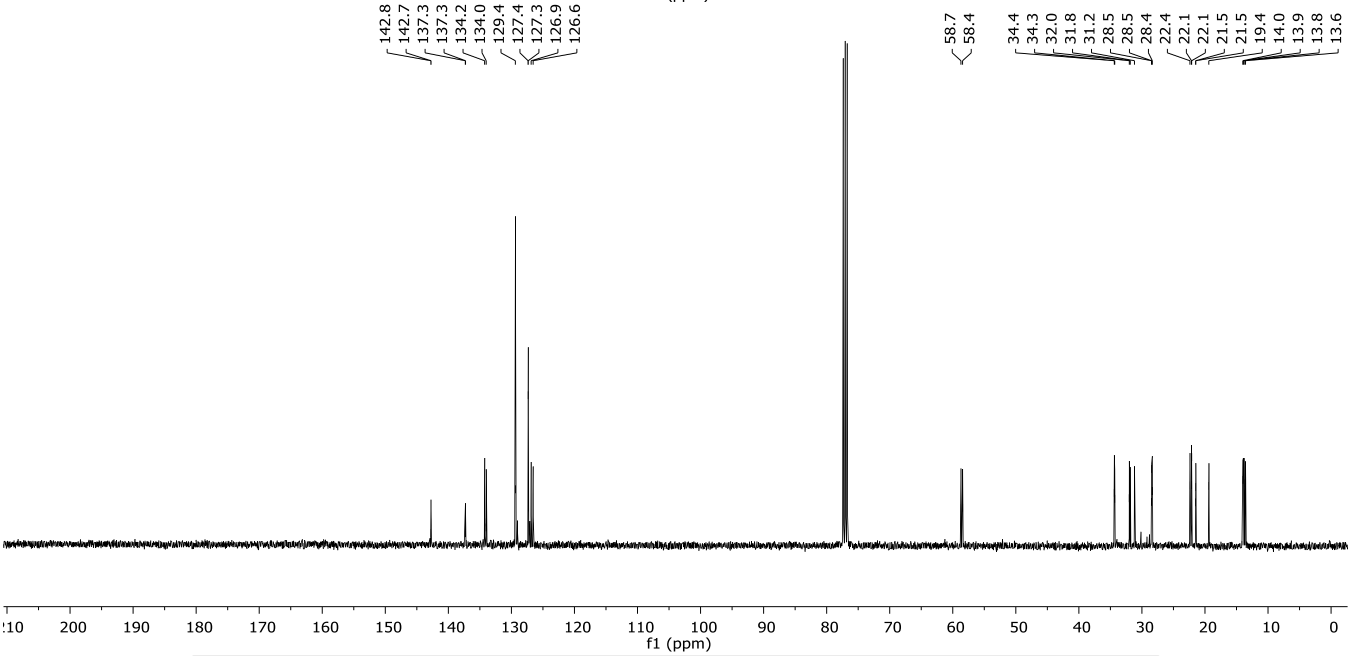

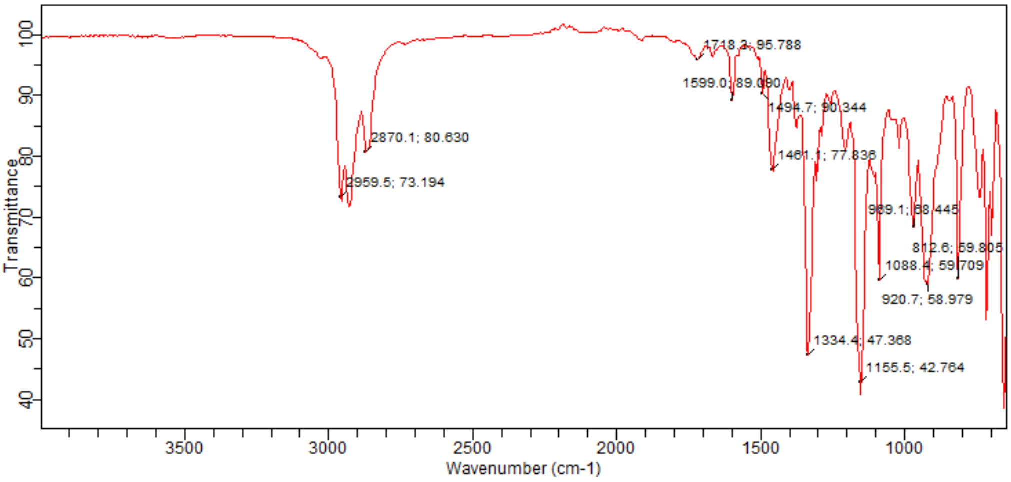

(*E*)-4-Chloro-1-(cyclododec-2-en-1-yl)-1H-pyrazole (3an) (^{1}H NMR: 400 MHz, ^{13}C NMR: 101 MHz, CDCl$_3$):

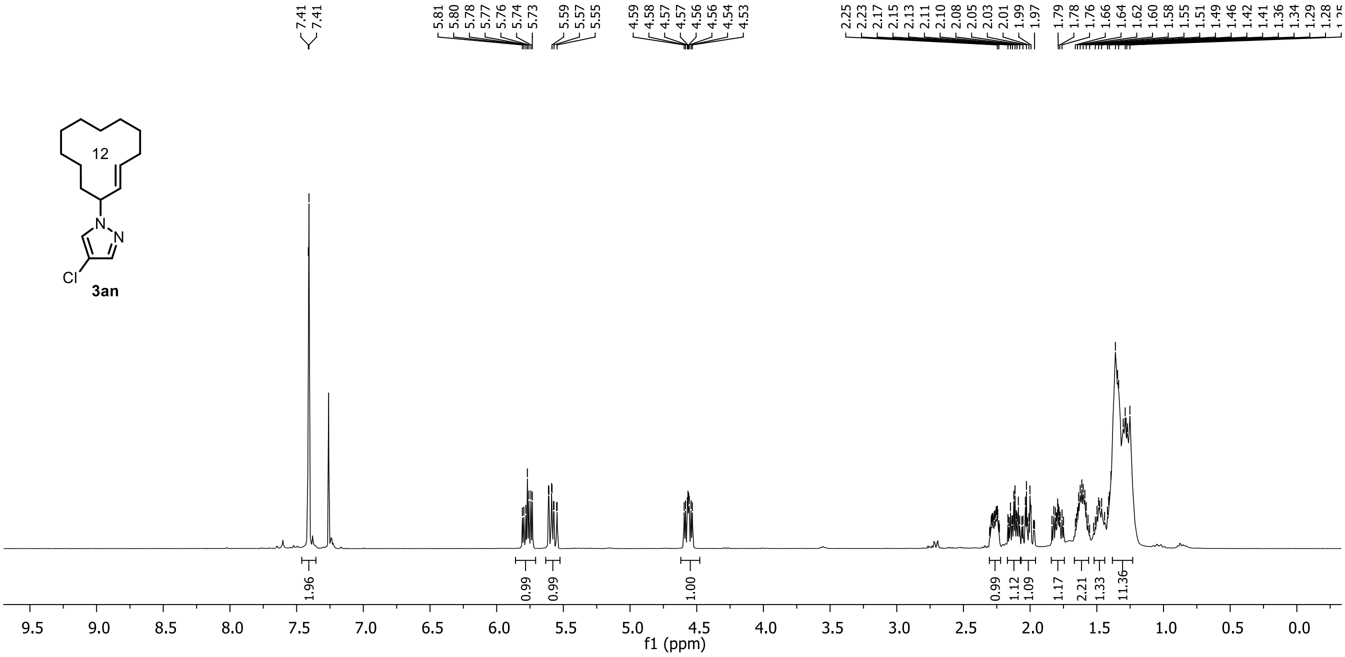

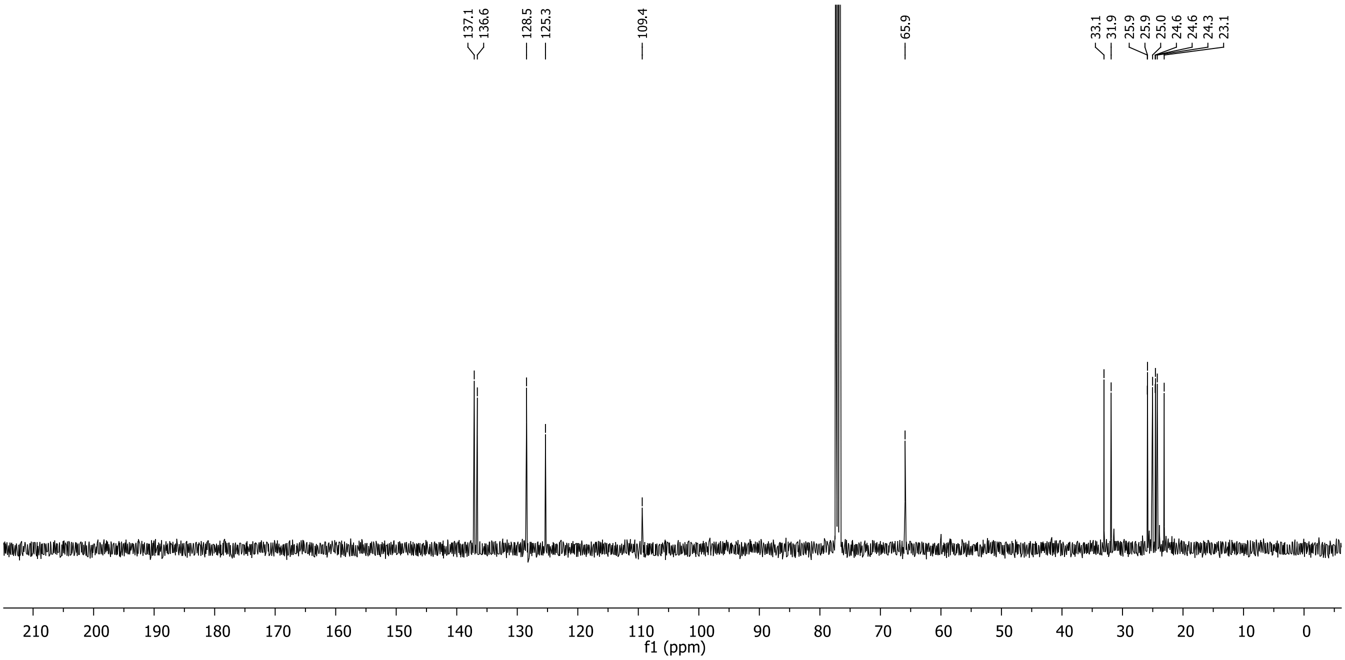

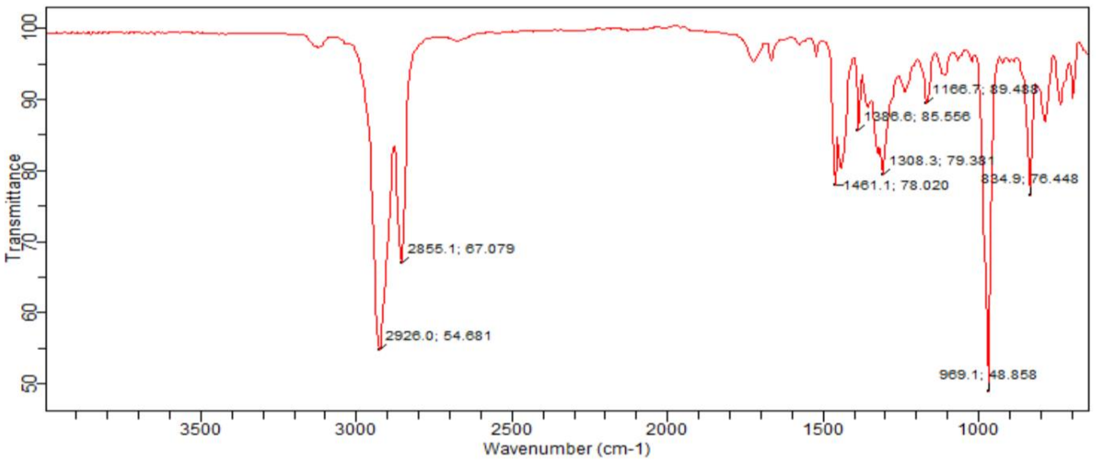

(*E*)-1-(Cyclododec-2-en-1-yl)-1H-benzo[d][1,2,3]triazole (3ao) (^{1}H NMR: 400 MHz, ^{13}C NMR: 101 MHz, $CDCl_3$):

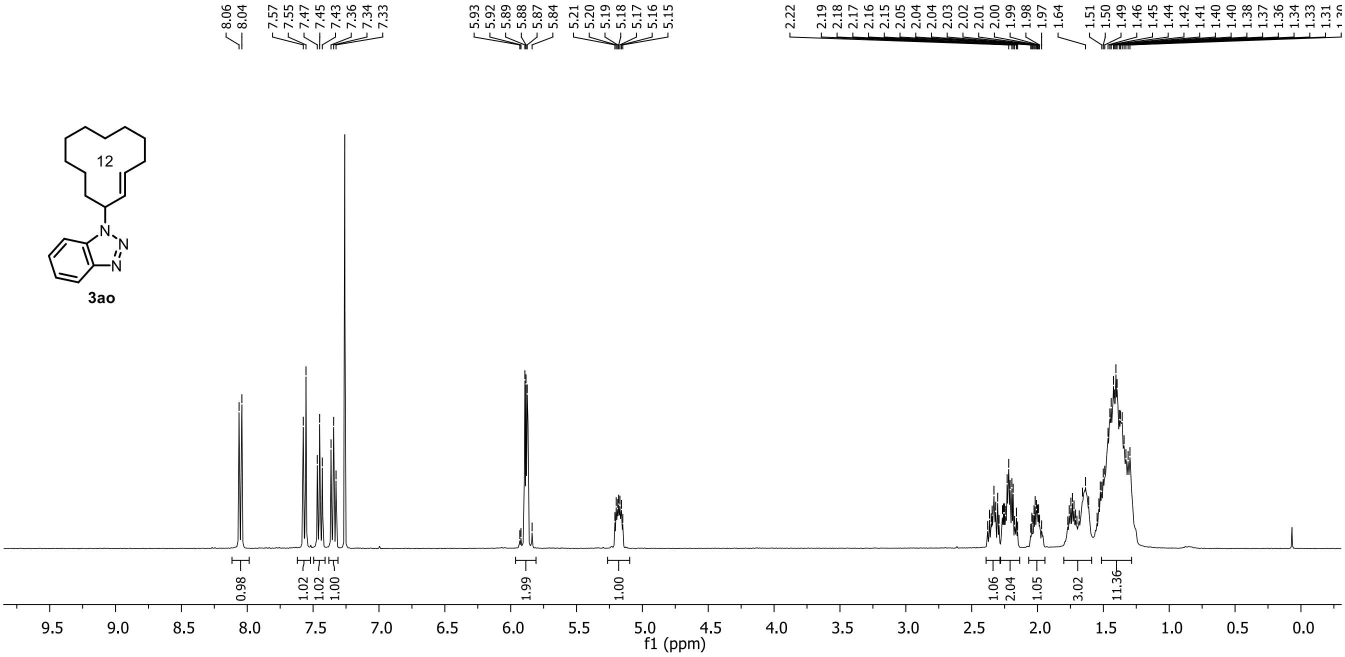

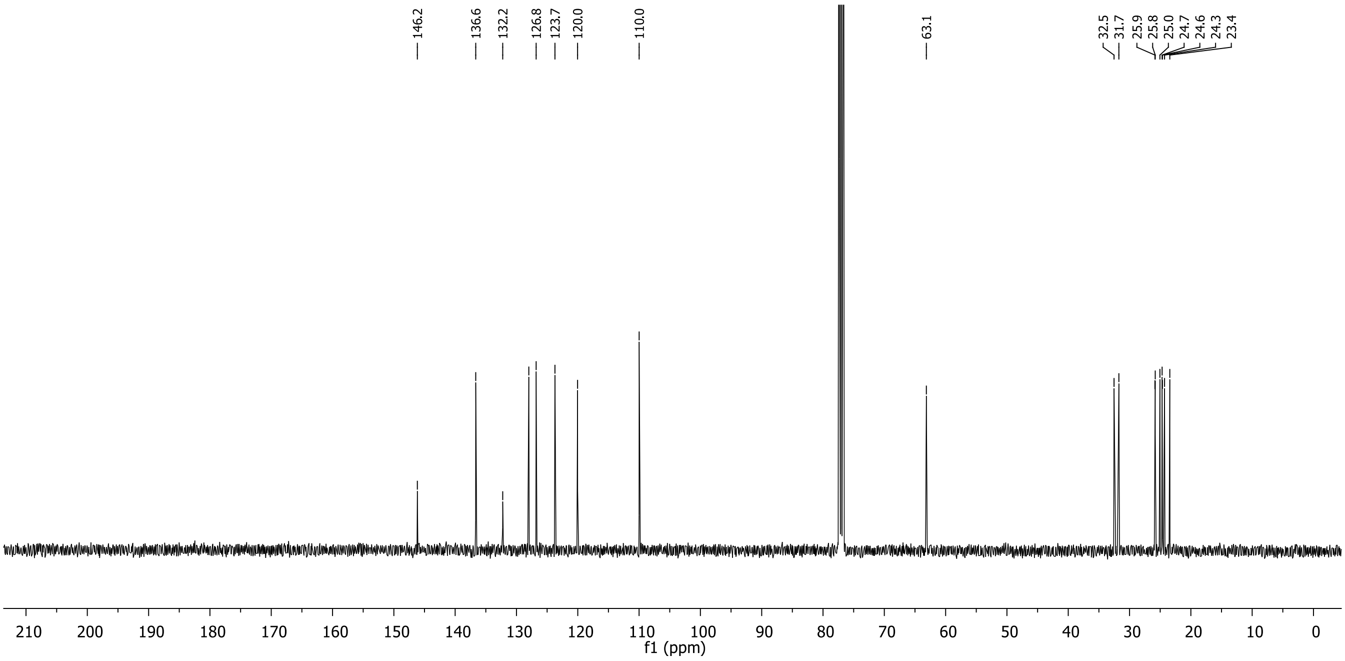

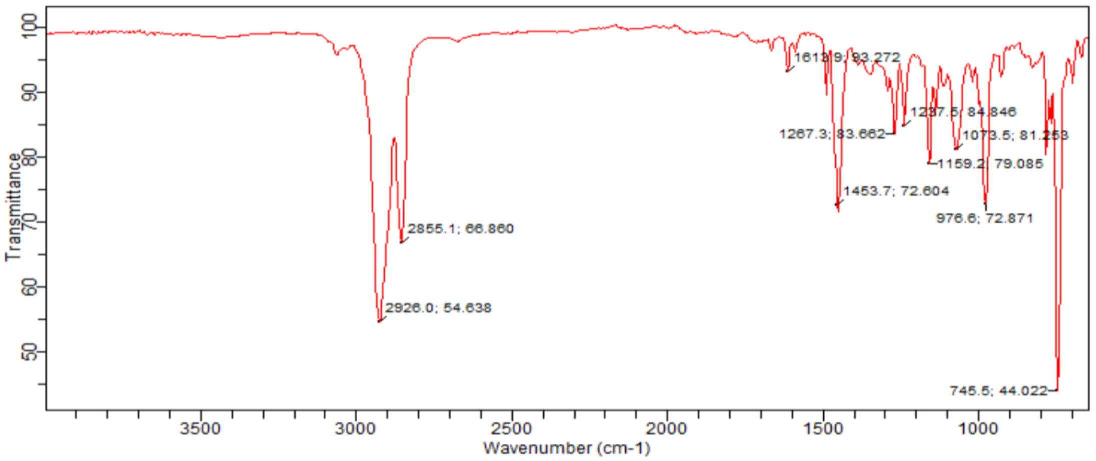

(*E*)-4-Chloro-1-(2-methylhex-3-en-2-yl)-1H-pyrazole (3ap) (^{1}H NMR: 300 MHz, ^{13}C NMR: 101 MHz, CDCl$_3$):

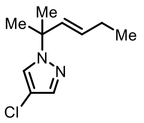

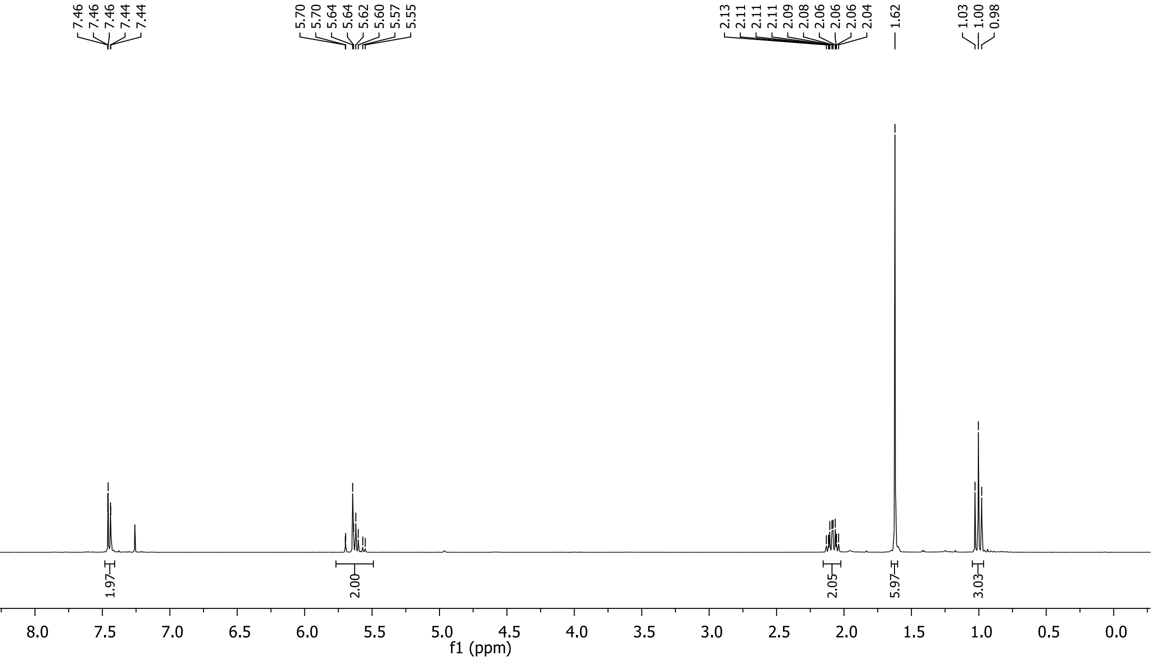

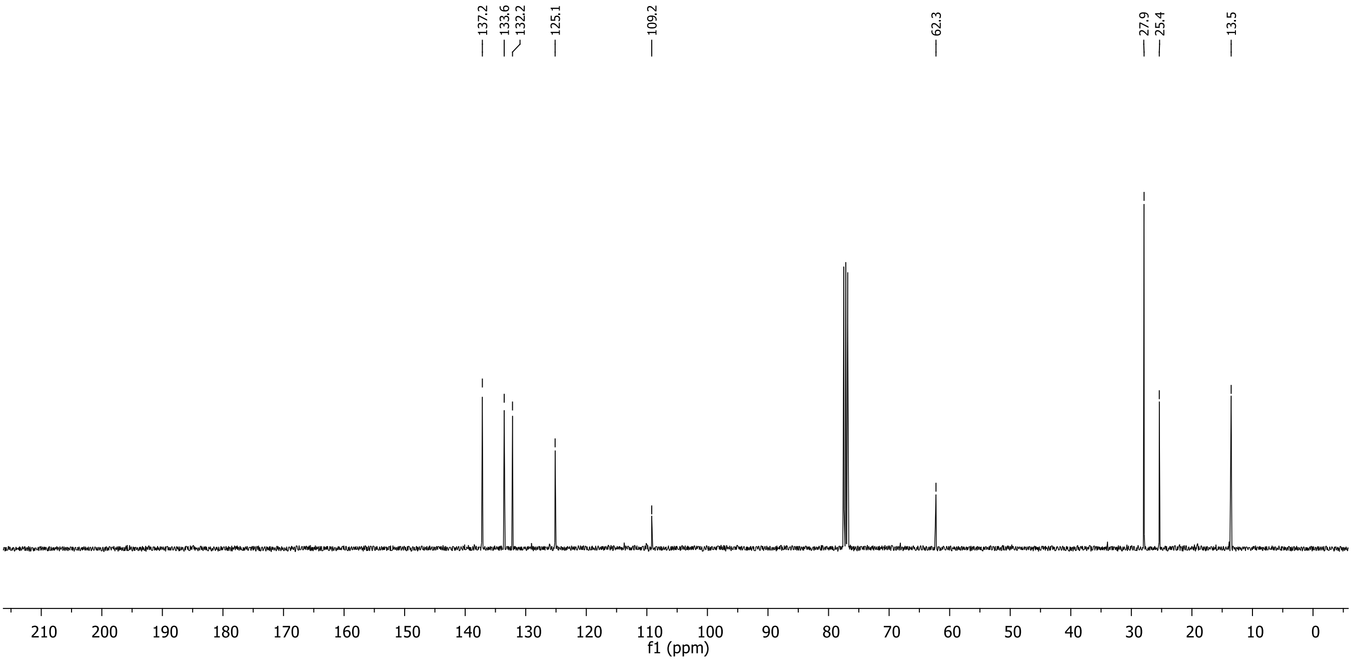

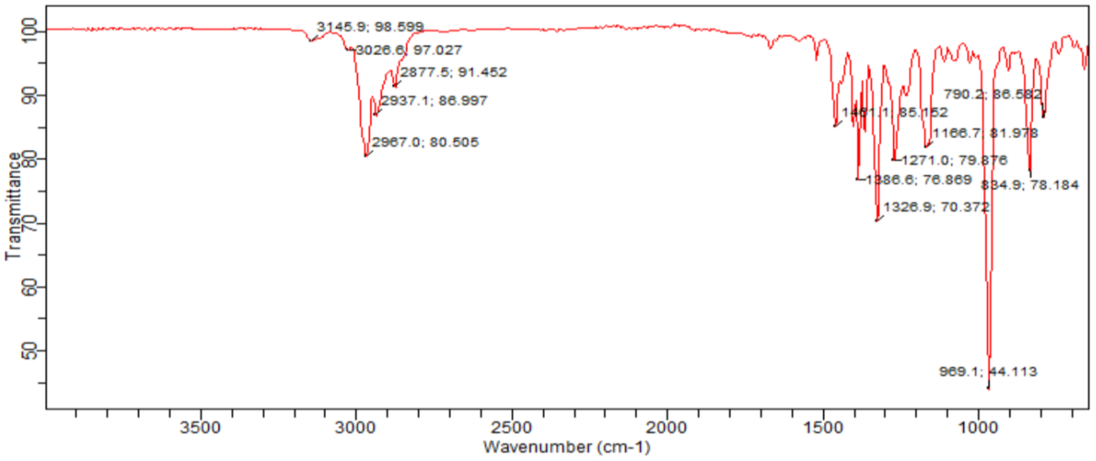

(*E*)-1-(8-((*tert*-Butyldimethylsilyl)oxy)-2,6-dimethyloct-3-en-2-yl)-4-chloro-1H-pyrazole (3aq) (^{1}H NMR: 300 MHz, ^{13}C NMR: 75 MHz, CDCl$_3$):

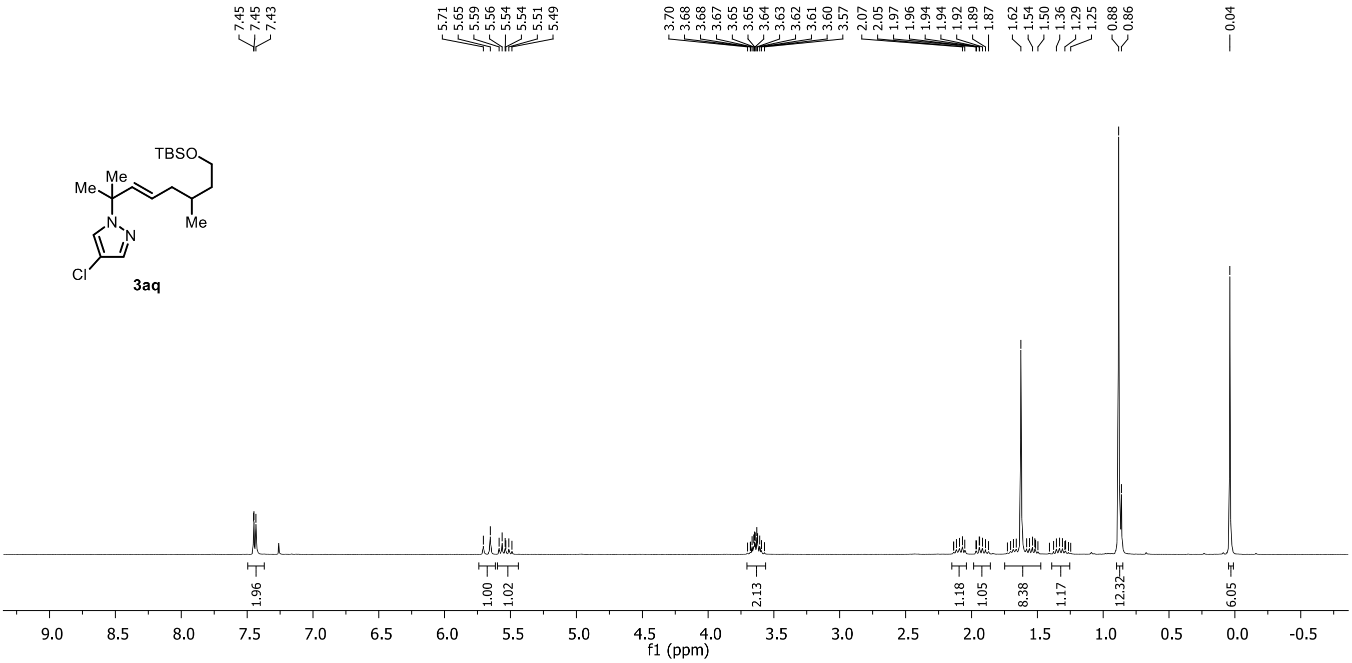

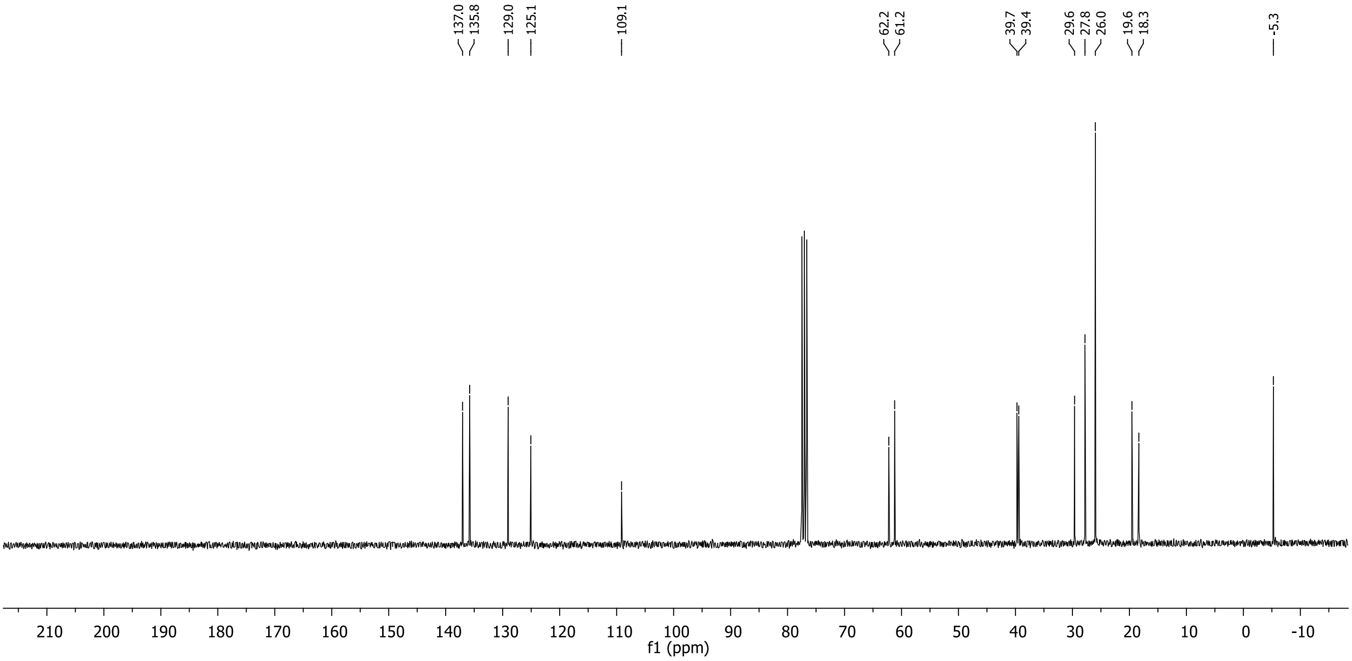

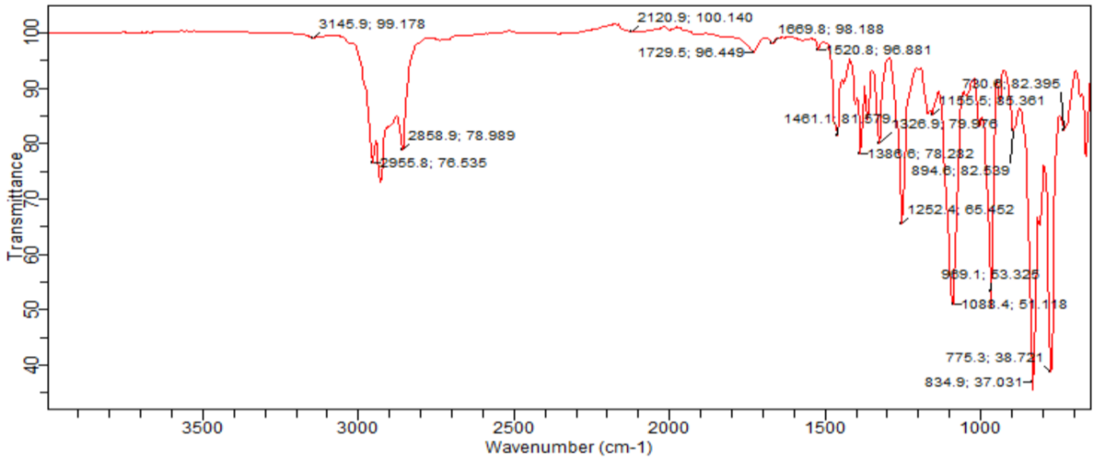

Ethyl (*E*)-4-(4-iodo-1H-pyrazol-1-yl)hex-2-enoate (3ar) (^{1}H NMR: 300 MHz, ^{13}C NMR: 101 MHz, $CDCl_3$):

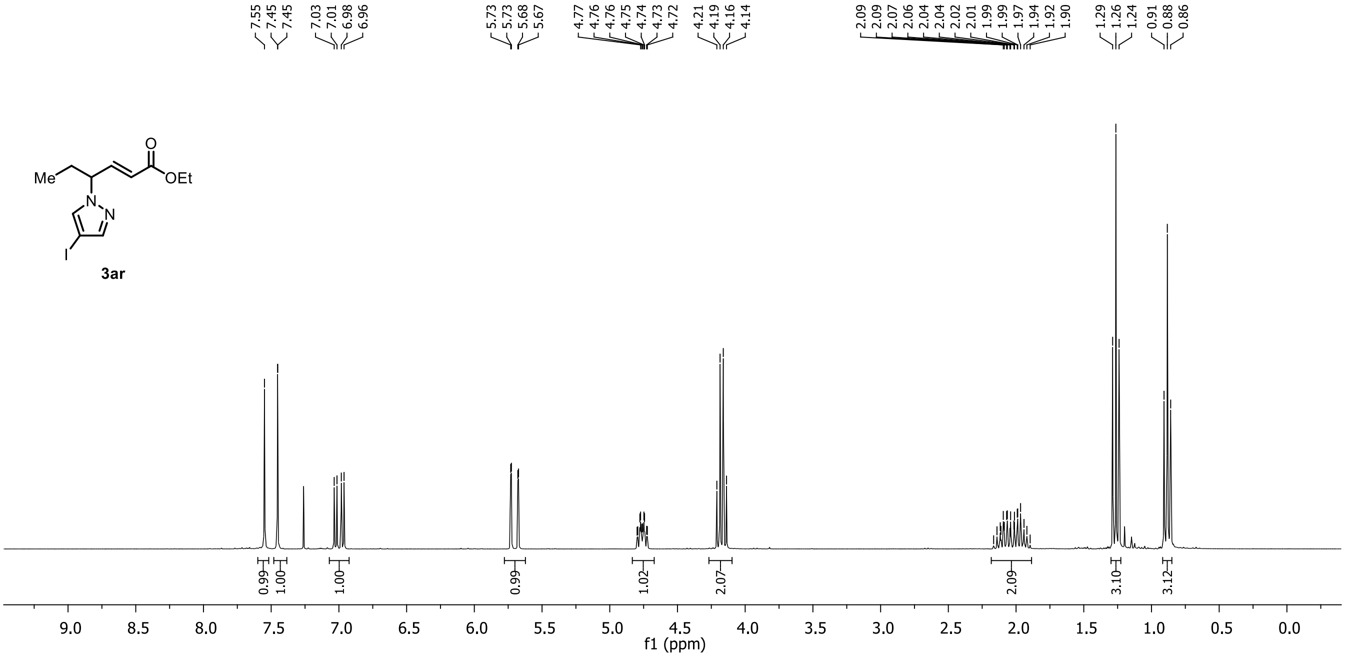

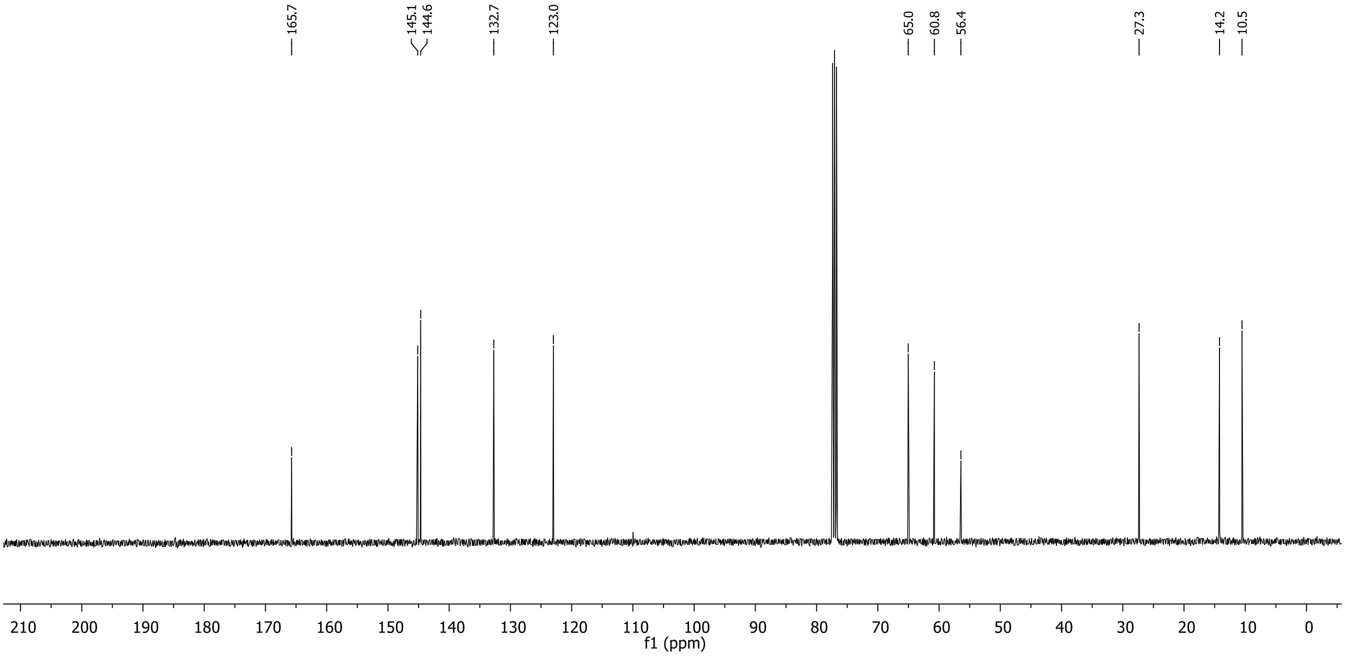

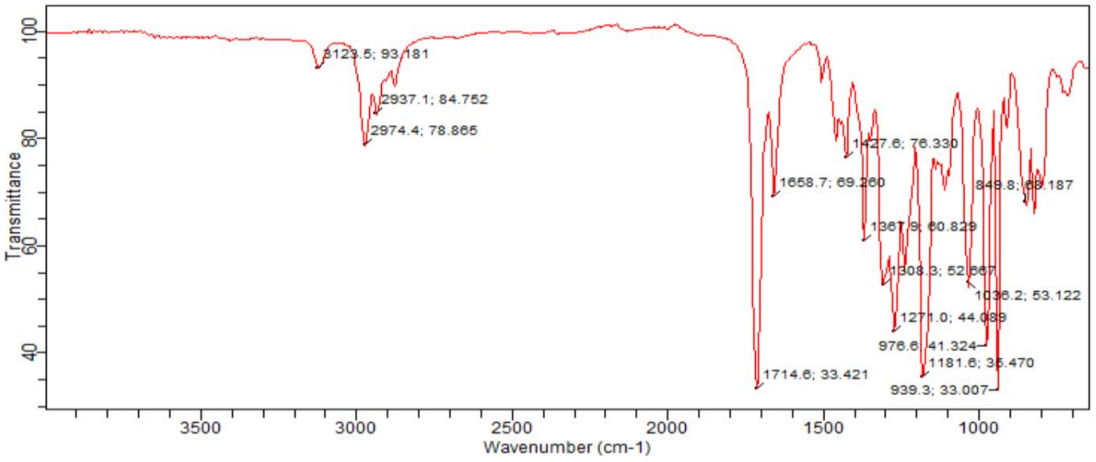

Ethyl (*E*)-1-(6-ethoxy-6-oxohex-4-en-3-yl)-1H-pyrazole-4-carboxylate (3as) (^{1}H NMR: 300 MHz, ^{13}C NMR: 75 MHz, $CDCl_3$):

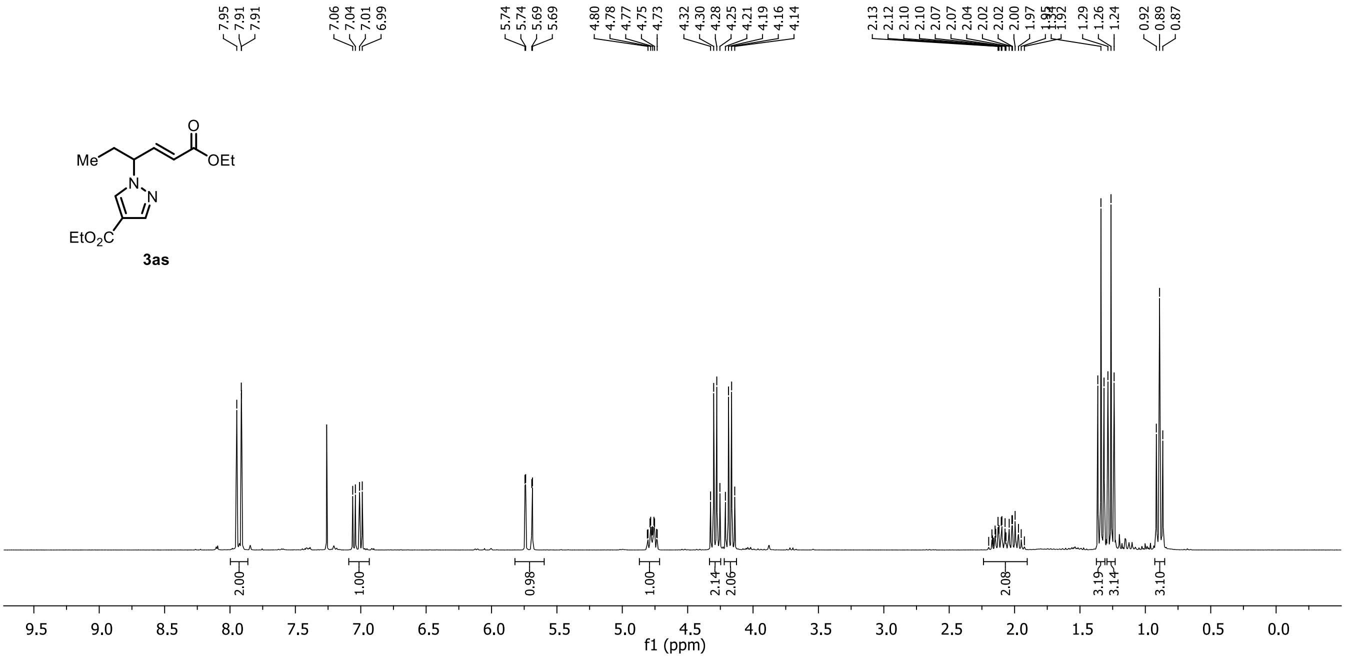

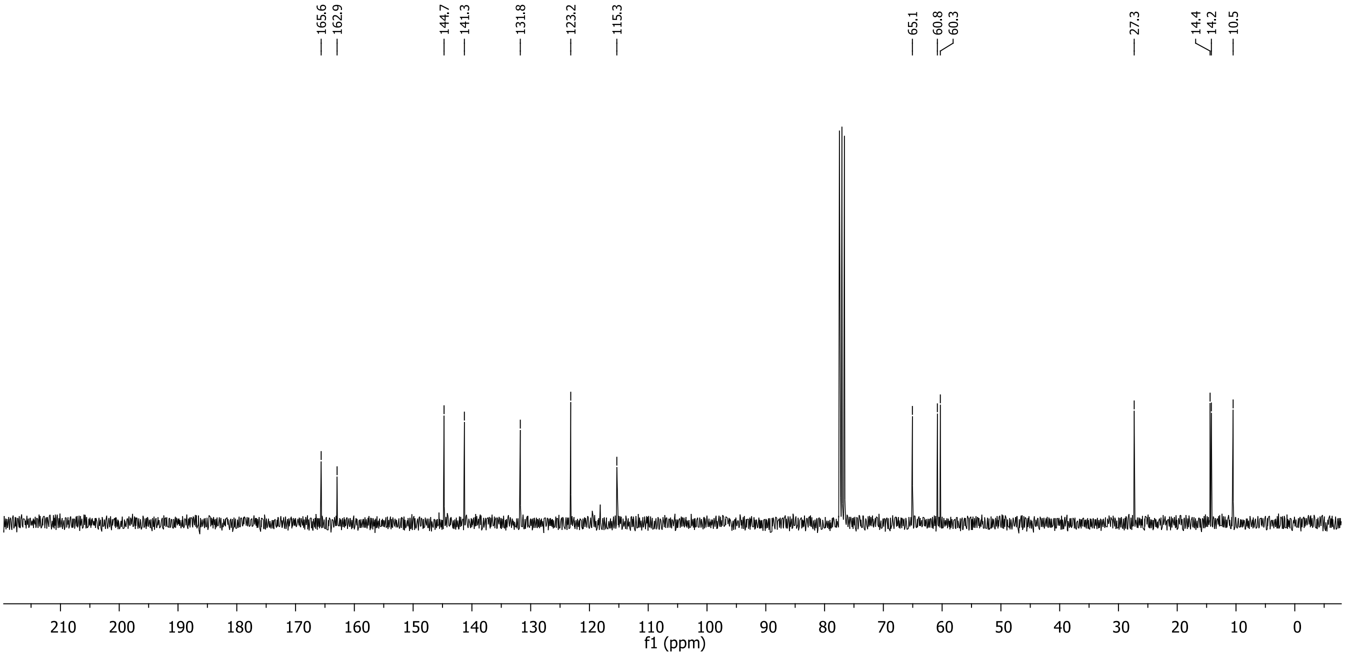

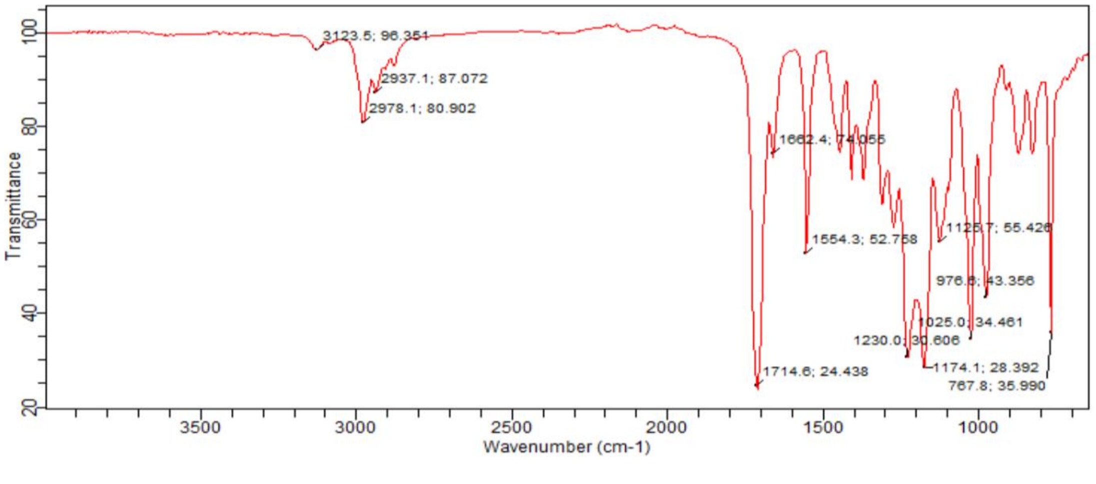

Ethyl (*E*)-4-(3-phenyl-1H-pyrazol-1-yl)hex-2-enoate (*N*1-isomer) and ethyl (*E*)-4-(5-phenyl-1H-pyrazol-1-yl)hex-2-enoate (*N*2-isomer) (3at) (^{1}H NMR: 400 MHz, ^{13}C NMR: 101 MHz, CDCl$_3$):

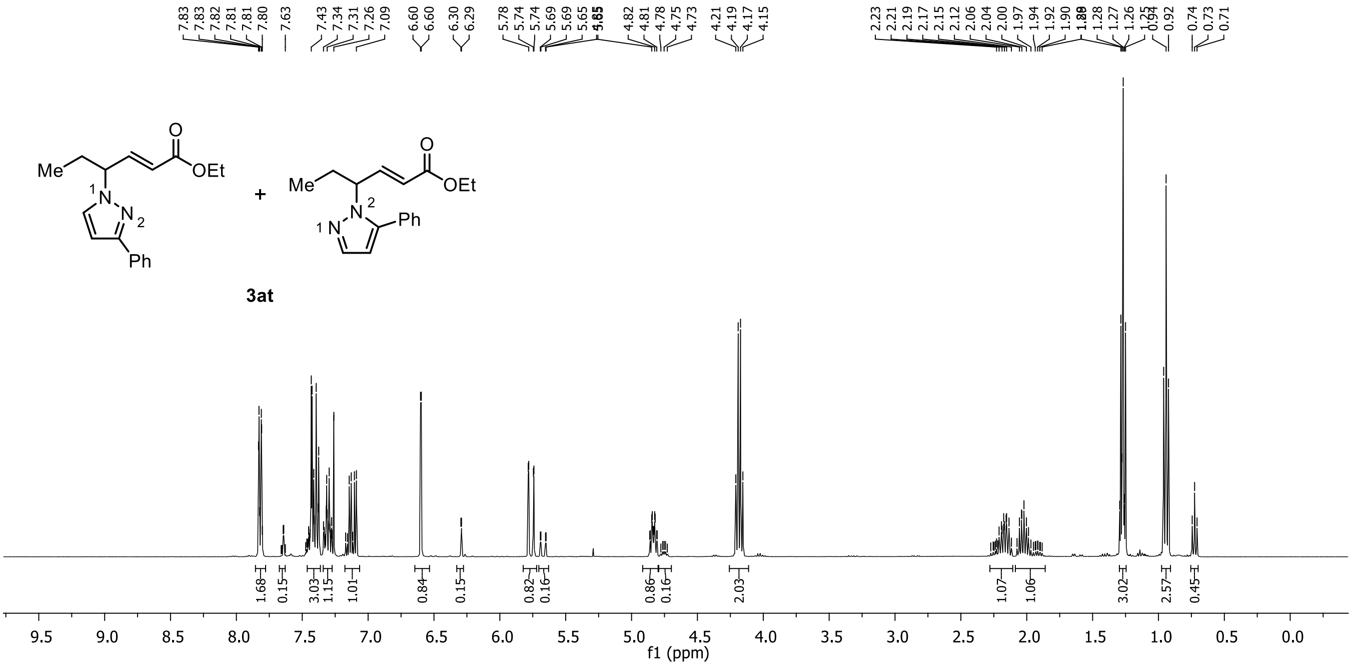

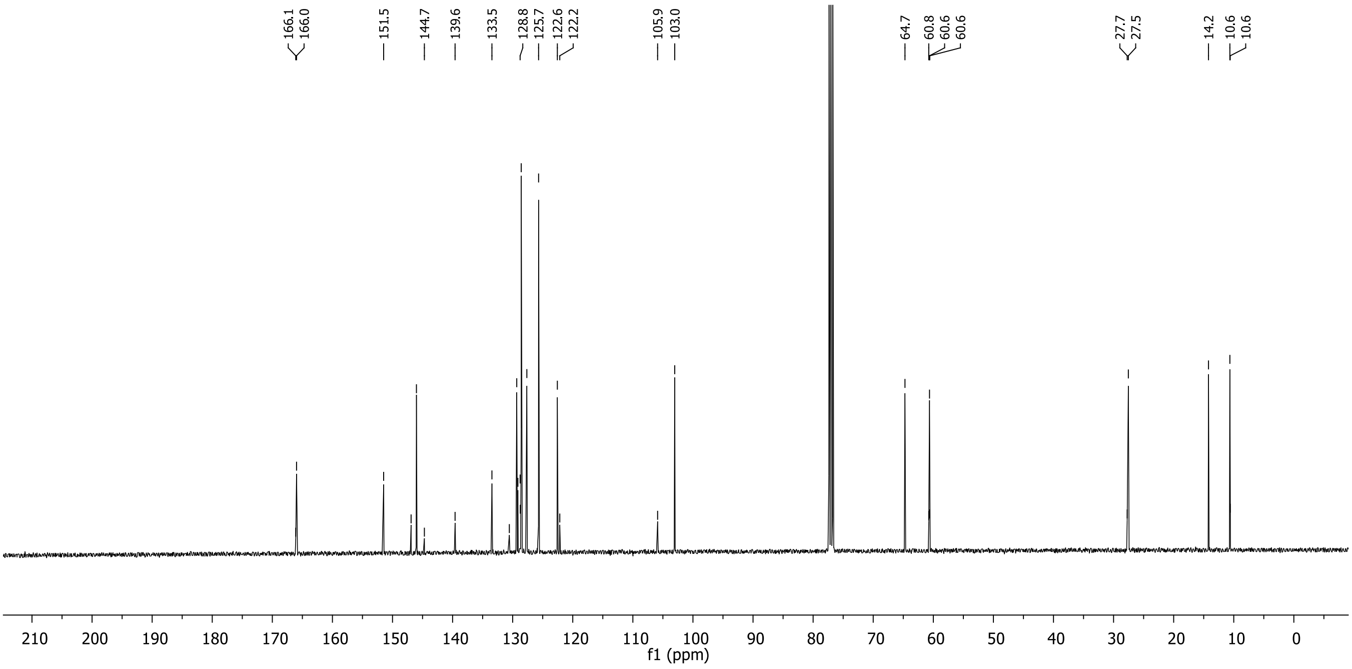

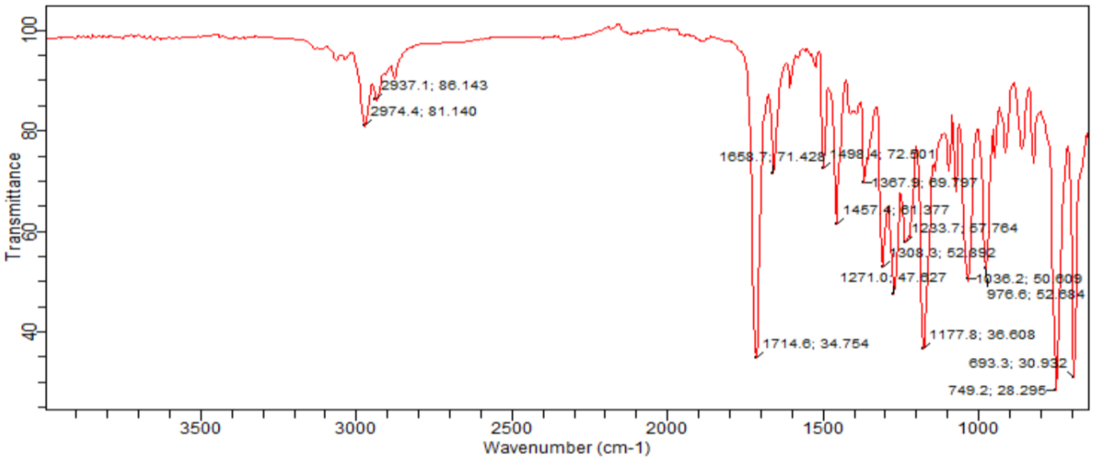

Ethyl (*E*)-4-(4-bromo-3-methyl-1H-pyrazol-1-yl)hex-2-enoate (*N*1-isomer) and ethyl (E)-4-(4-bromo-5-methyl-1H-pyrazol-1-yl)hex-2-enoate (*N*2-isomer) (3au) (^{1}H NMR: 400 MHz, ^{13}C NMR: 101 MHz, $CDCl_3$):

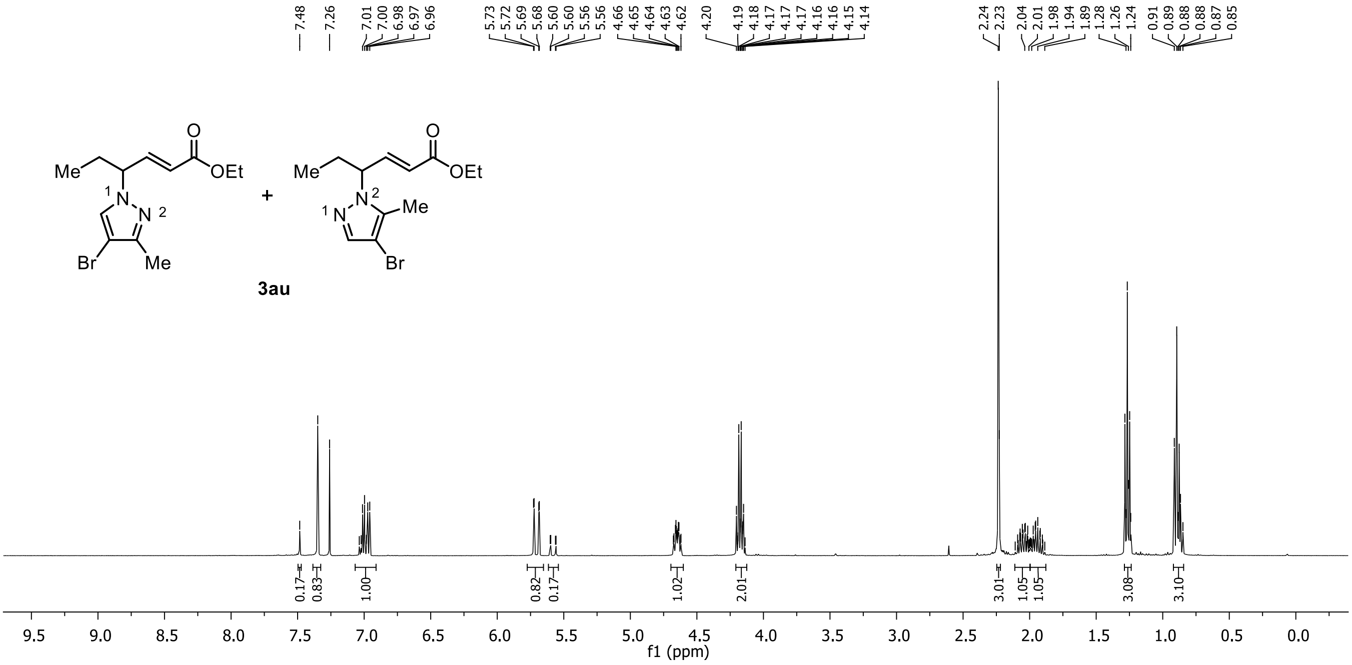
Me
OEt
+
Me
OEt
Br
Me
Br
Me
3au
7.48
7.26
7.01
7.00
6.98
6.97
6.96
5.73
5.72
5.69
5.68
5.60
5.60
5.56
5.56
4.66
4.65
4.64
4.63
4.62
4.20
4.19
4.18
4.17
4.17
4.17
4.16
4.15
4.14
2.24
2.23
2.04
2.01
1.98
1.94
1.89
1.28
1.26
1.24
0.91
0.89
0.88
0.88
0.87
0.85
0.17
0.83
1.00
0.82
0.17
1.02
2.01
3.01
1.05
1.05
3.08
3.10
f1 (ppm)

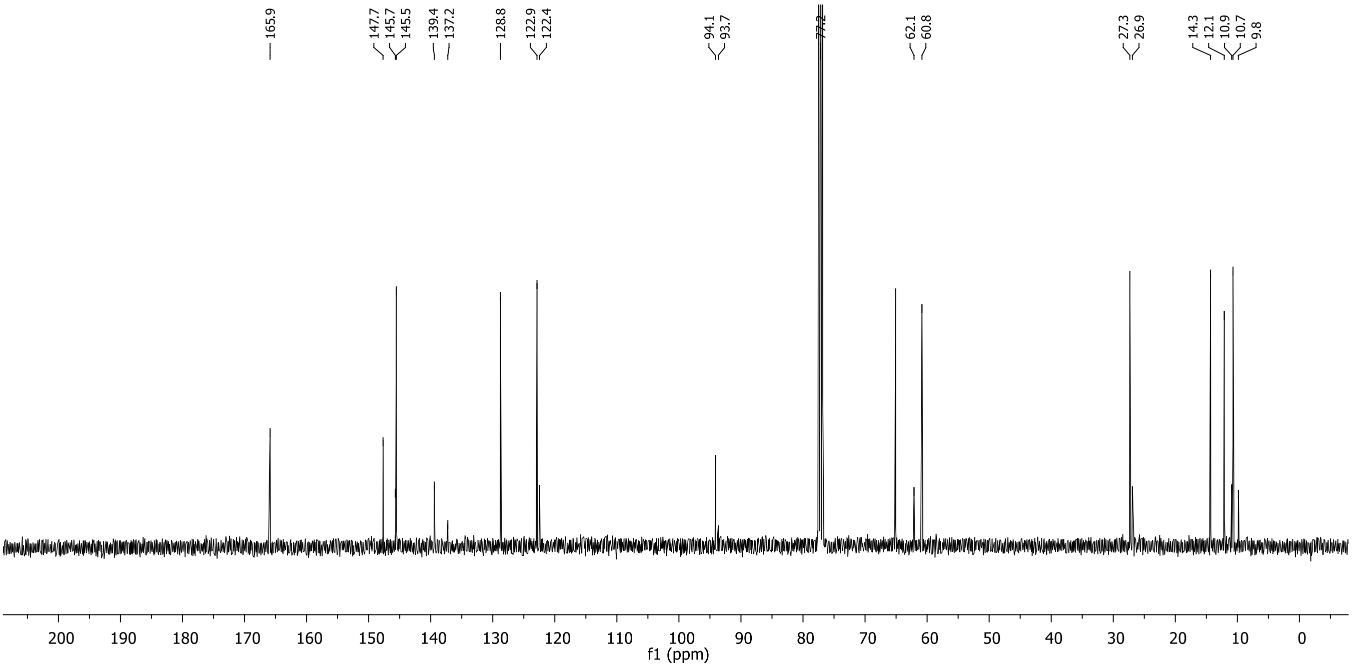
165.9
147.7
145.7
145.5
139.4
137.2
128.8
122.9
122.4
94.1
93.7
62.1
60.8
27.3
26.9
14.3
12.1
10.9
10.7
9.8
f1 (ppm)

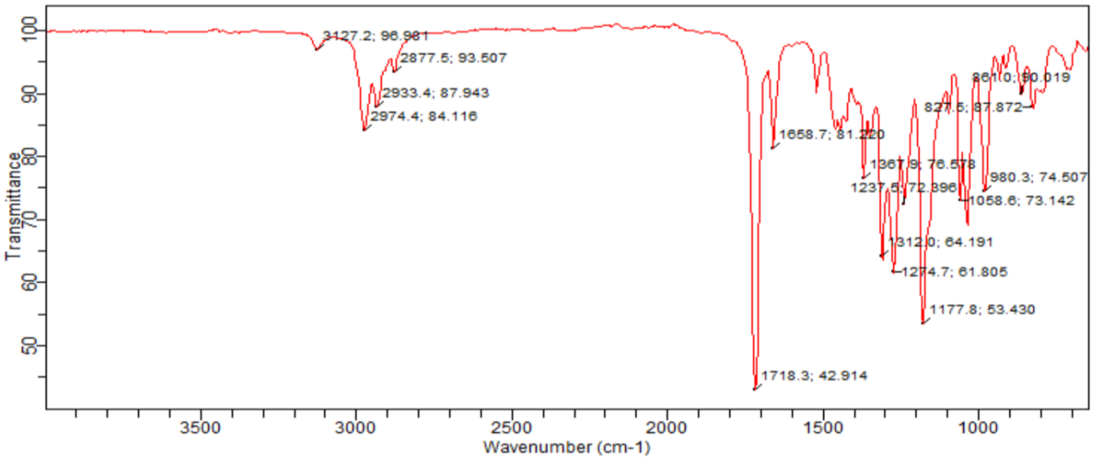
3127.2; 96.981
2877.5; 93.507
2933.4; 87.943
2974.4; 84.116
1658.7; 81.220
1367.9; 76.578
1237.5; 72.896
980.3; 74.507
1058.6; 73.142
1312.0; 64.191
1274.7; 61.805
1177.8; 53.430
1718.3; 42.914
861.0; 90.019
827.5; 87.872
Transmittance
Wavenumber (cm-1)

Ethyl (*E*)-4-(1H-benzo[d][1,2,3]triazol-1-yl)hex-2-enoate (3av) (^{1}H NMR: 300 MHz, ^{13}C NMR: 101 MHz, $CDCl_3$):

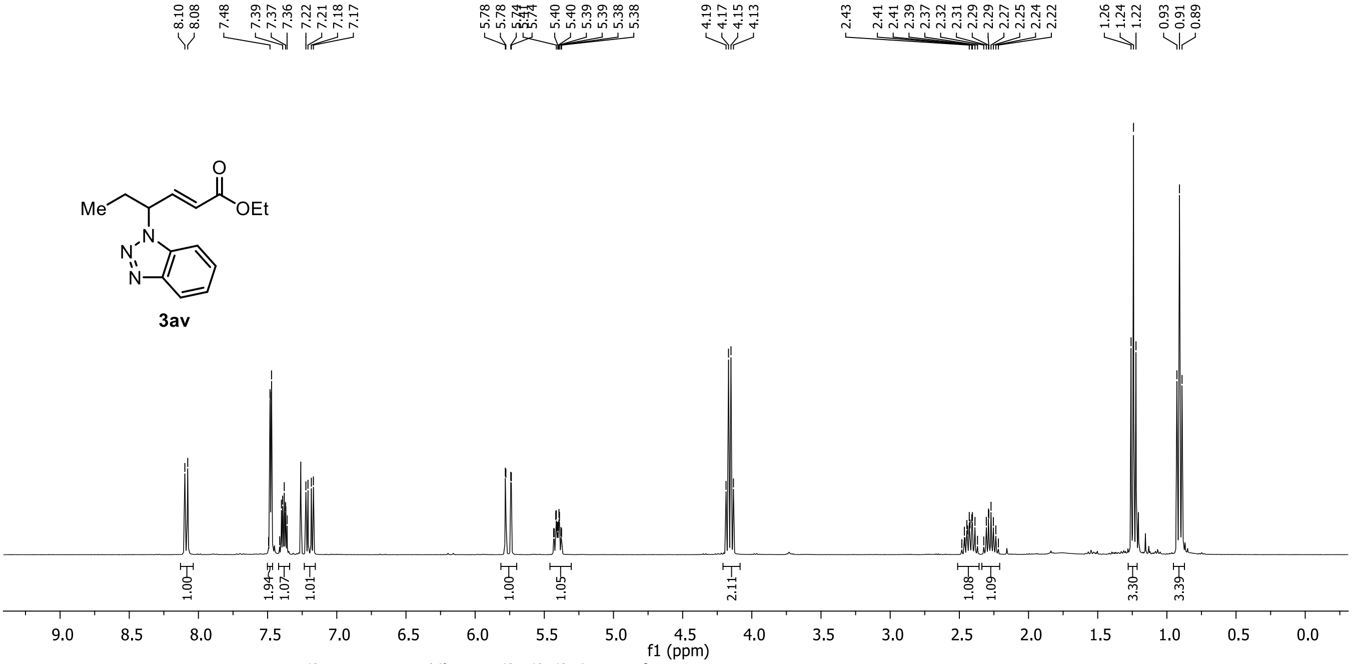

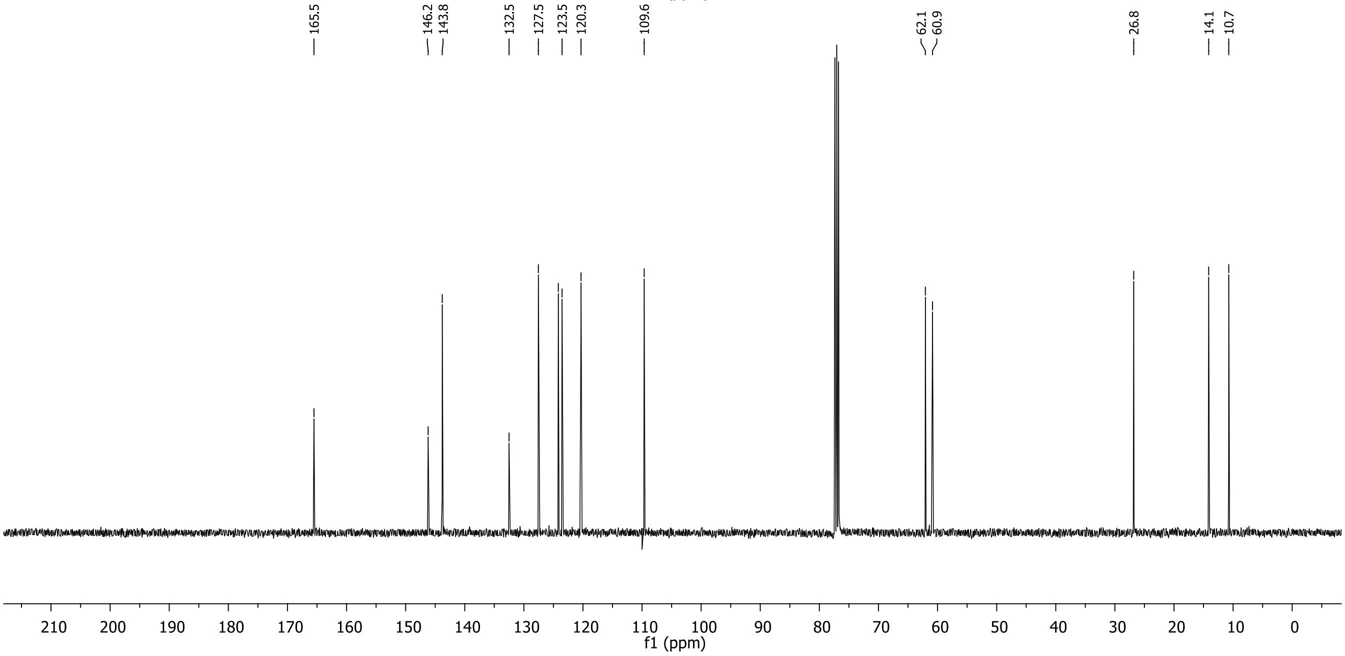

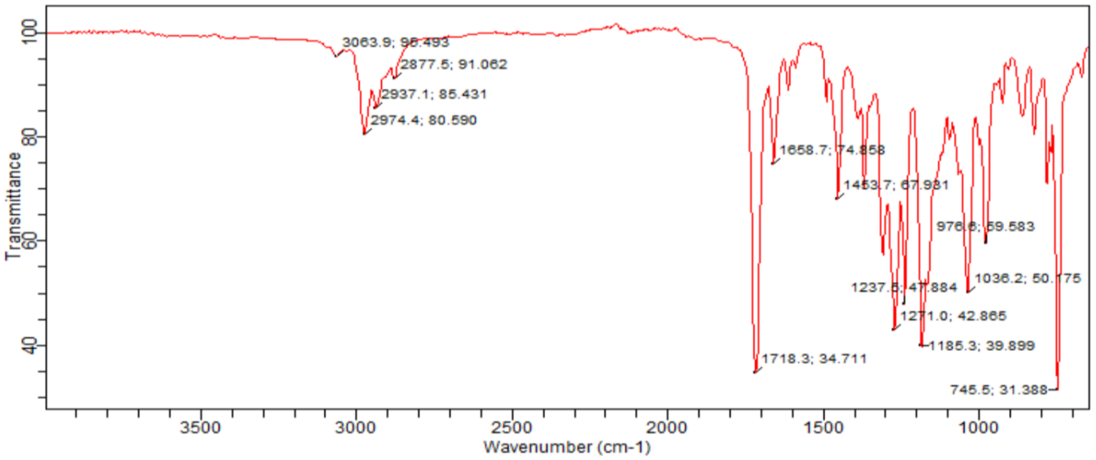

Ethyl (*E*)-4-(5-methyl-1H-benzo[d][1,2,3]triazol-1-yl)hex-2-enoate (C5-isomer) and ethyl (*E*)-4-(6-methyl-1H-benzo[d][1,2,3]triazol-1-yl)hex-2-enoate (C6-isomer) (3aw) (^{1}H NMR: 400 MHz, ^{13}C NMR: 101 MHz, $CDCl_3$):

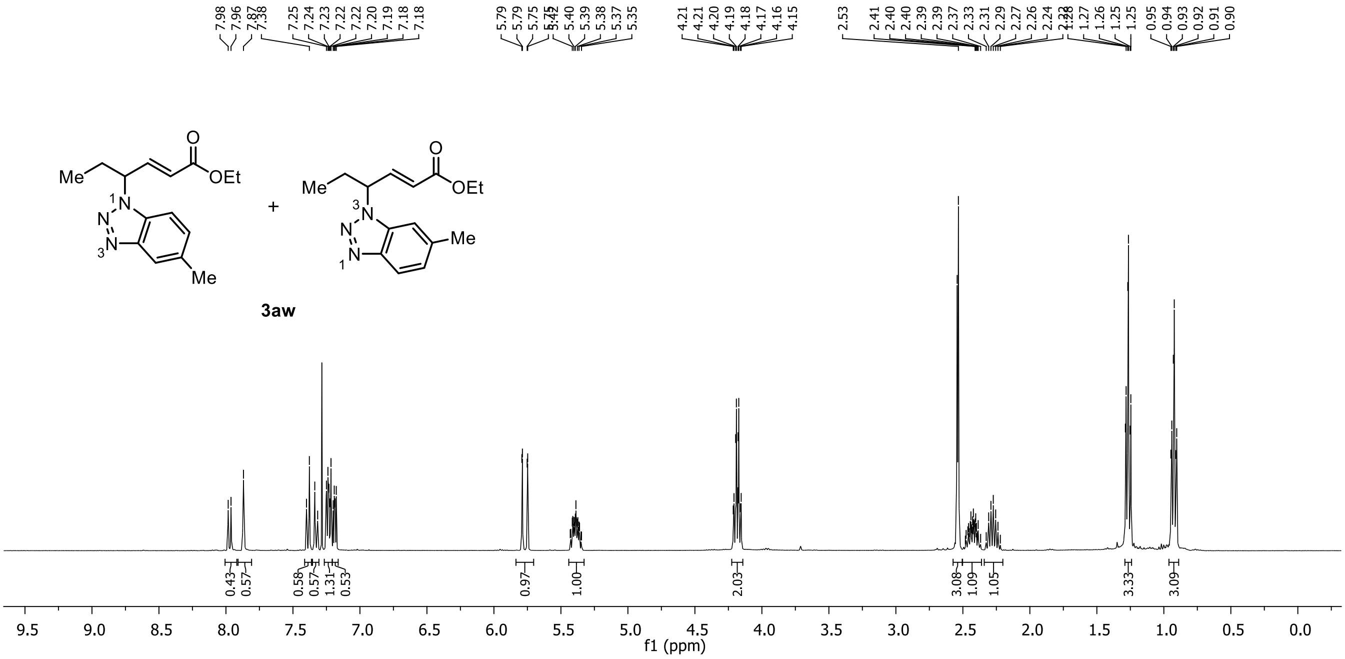

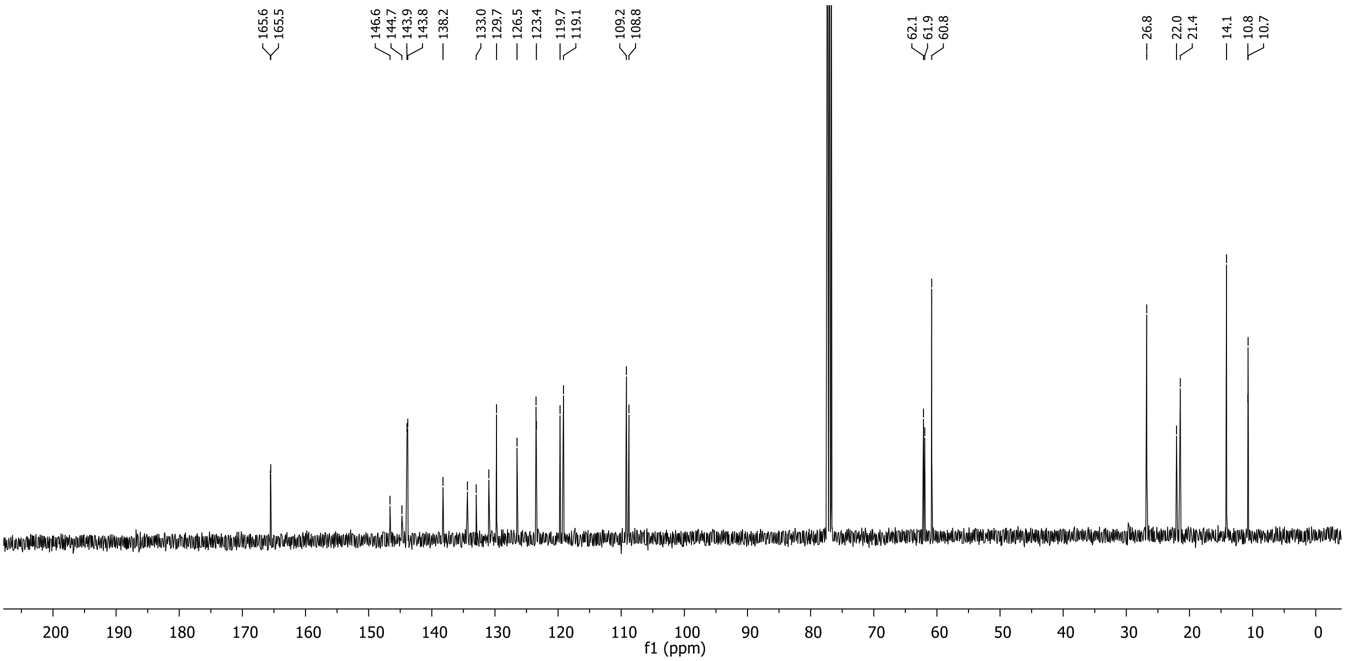

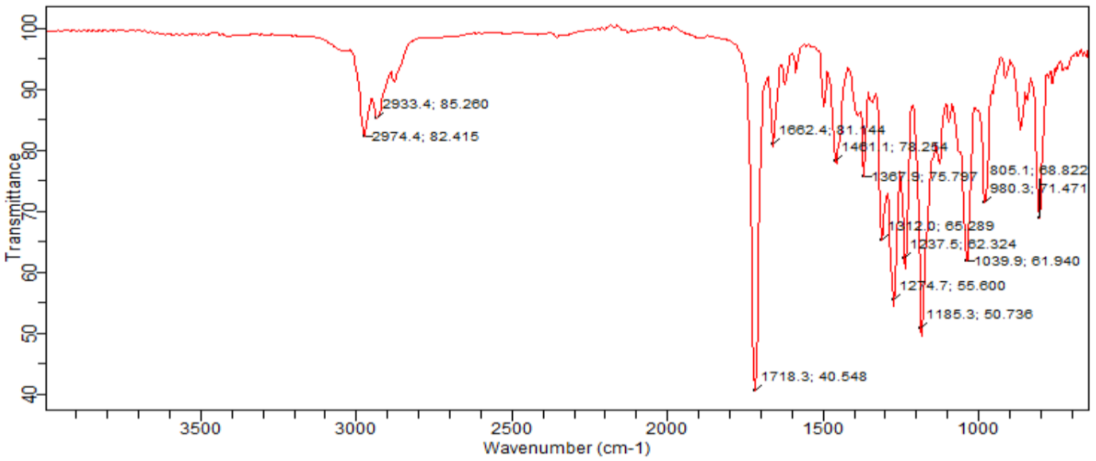

Diethyl (*E*)-4-(1H-pyrazol-1-yl)hex-2-enedioate (3ax) (^{1}H NMR: 400 MHz, ^{13}C NMR: 101 MHz, $CDCl_3$):

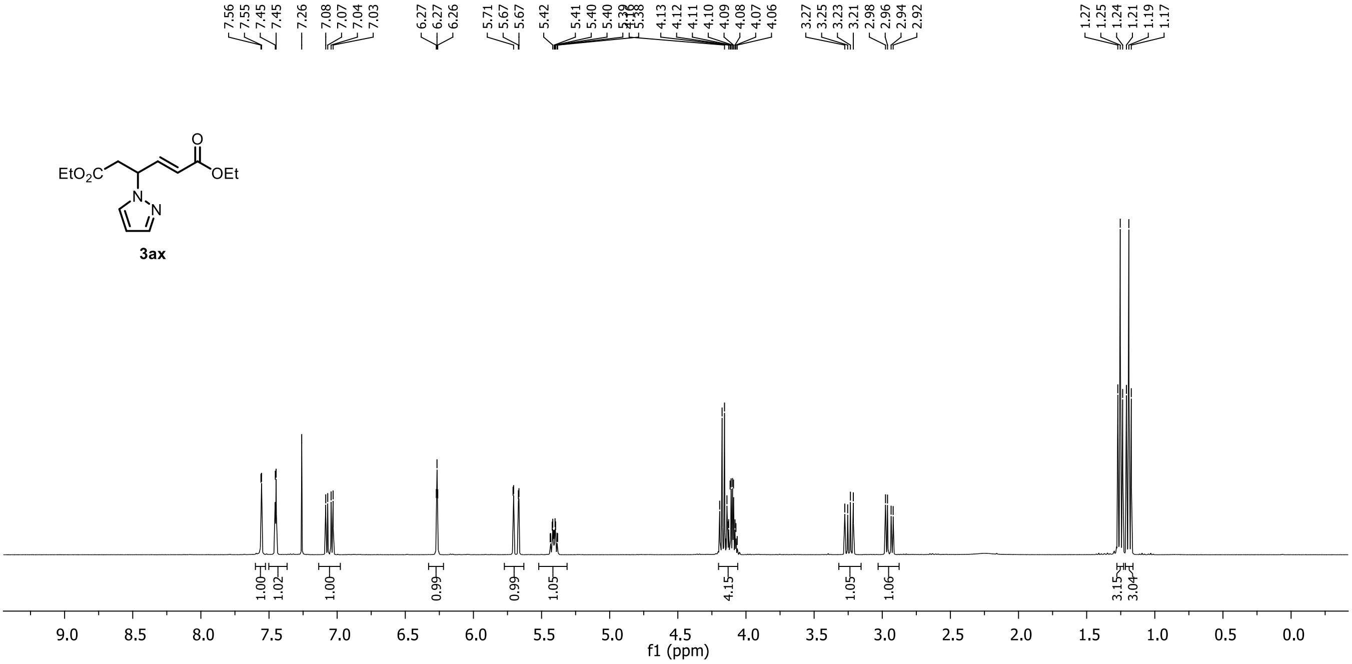

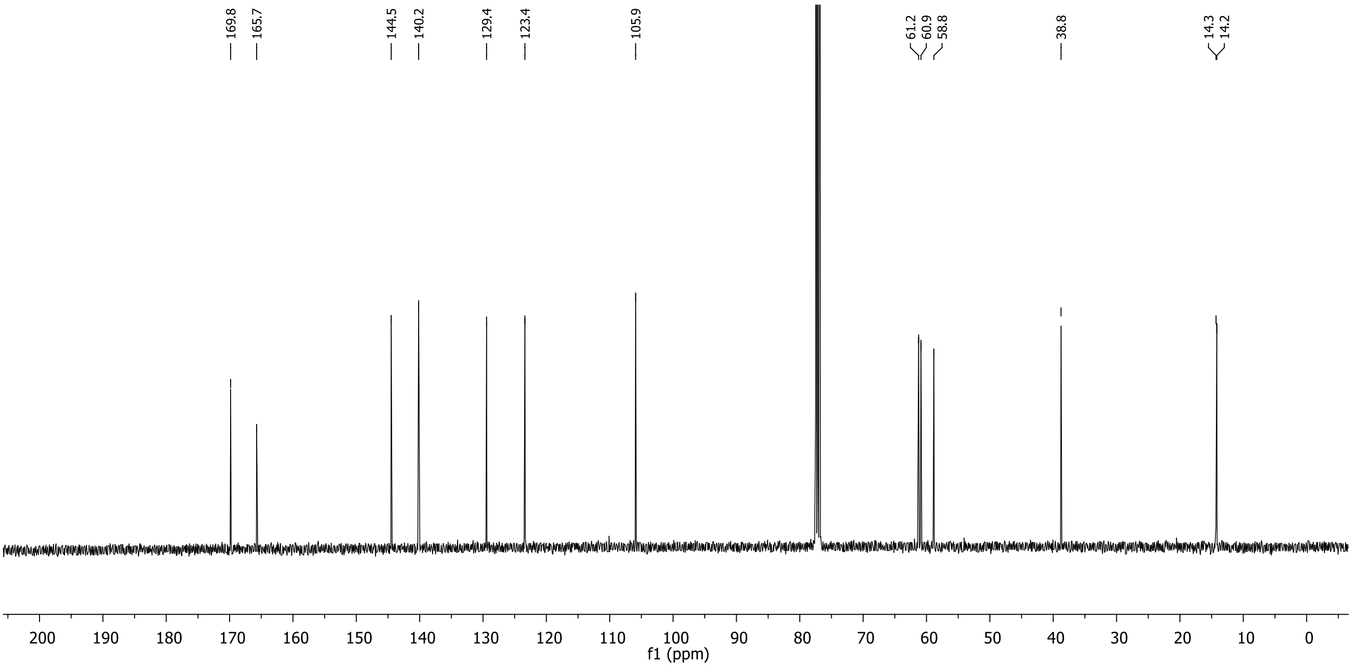

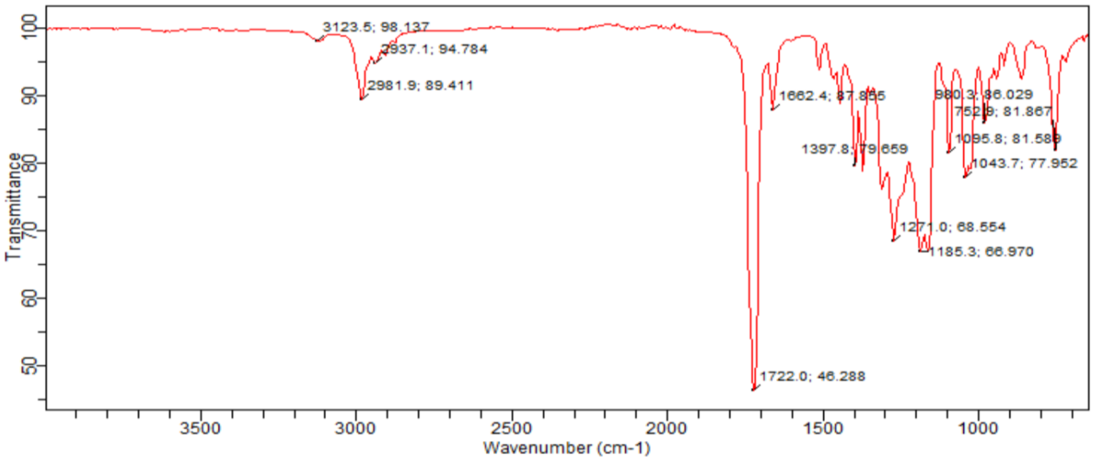

Diethyl (*E*)-4-(4-methyl-1H-pyrazol-1-yl)hex-2-enedioate (3ay) (^{1}H NMR: 400 MHz, ^{13}C NMR: 101 MHz, $CDCl_3$):

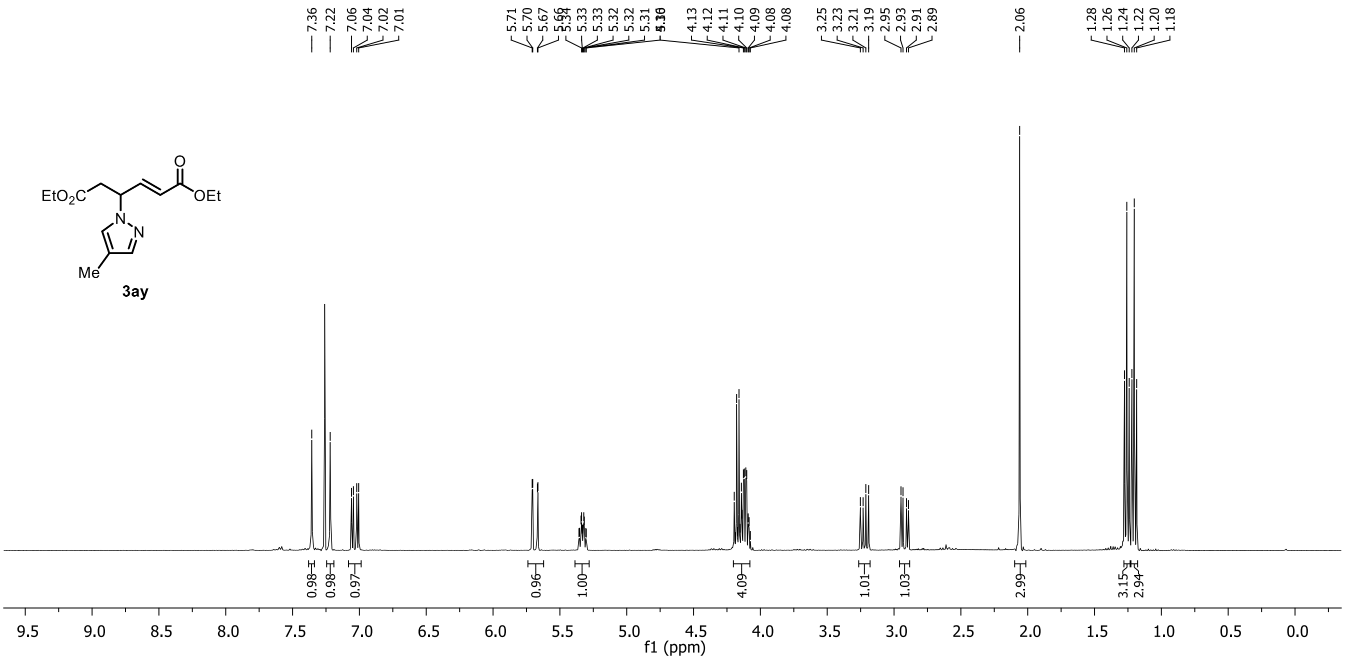

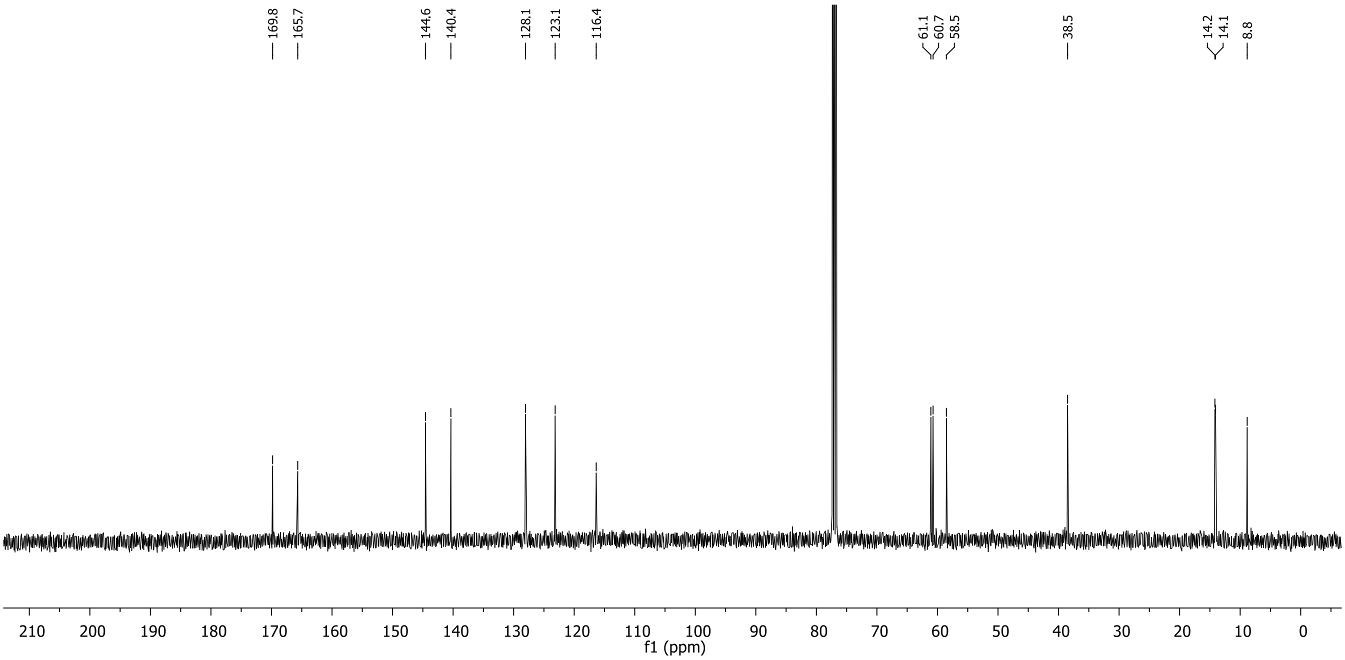

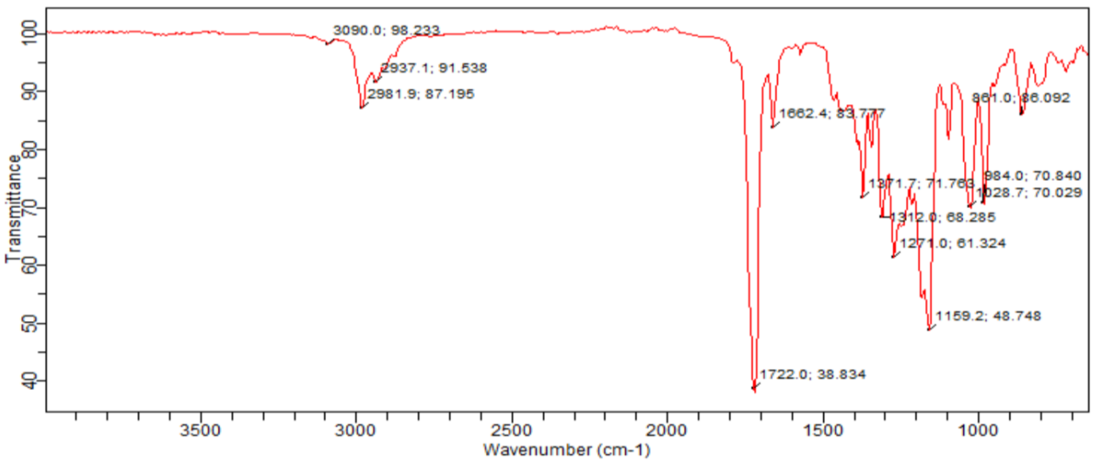

Diethyl (*E*)-4-(4-(trifluoromethyl)-1H-pyrazol-1-yl)hex-2-enedioate (3az) (^{1}H NMR: 400 MHz, ^{13}C NMR: 101 MHz, ^{19}F NMR: 376 MHz, $CDCl_3$):

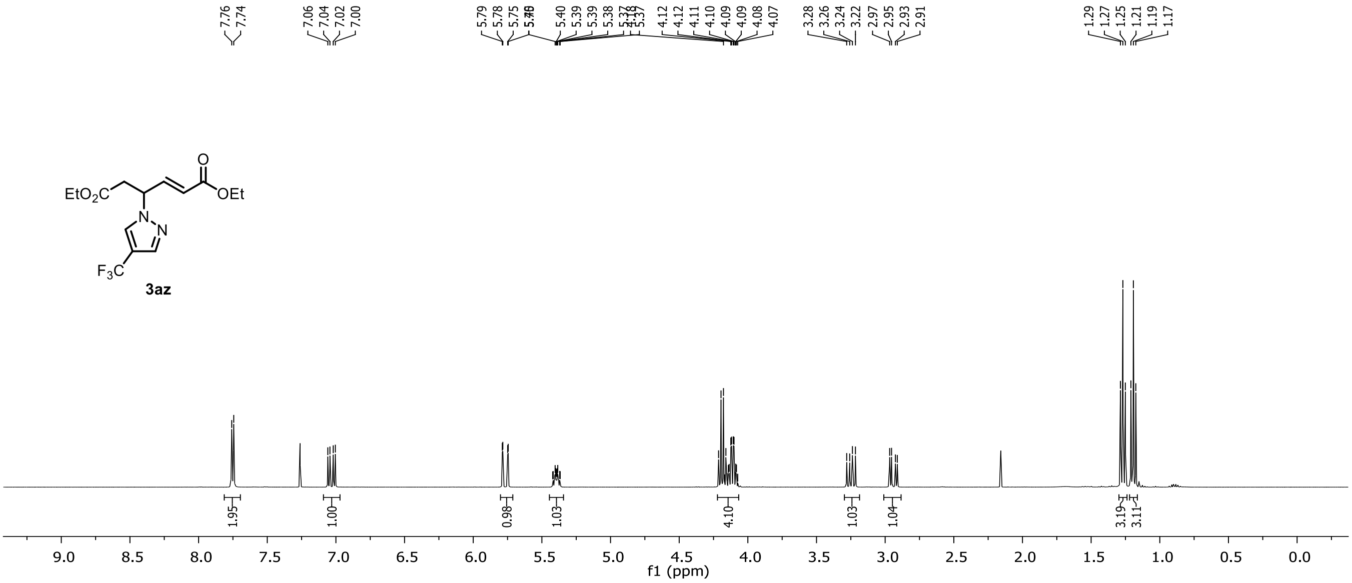

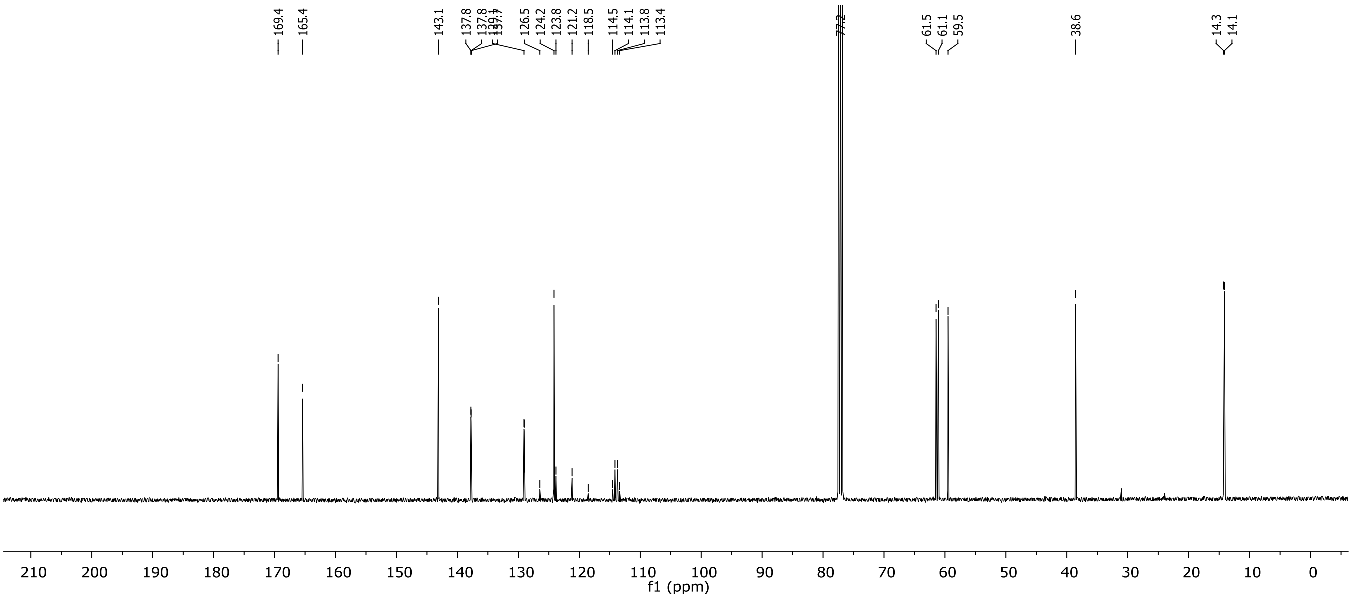

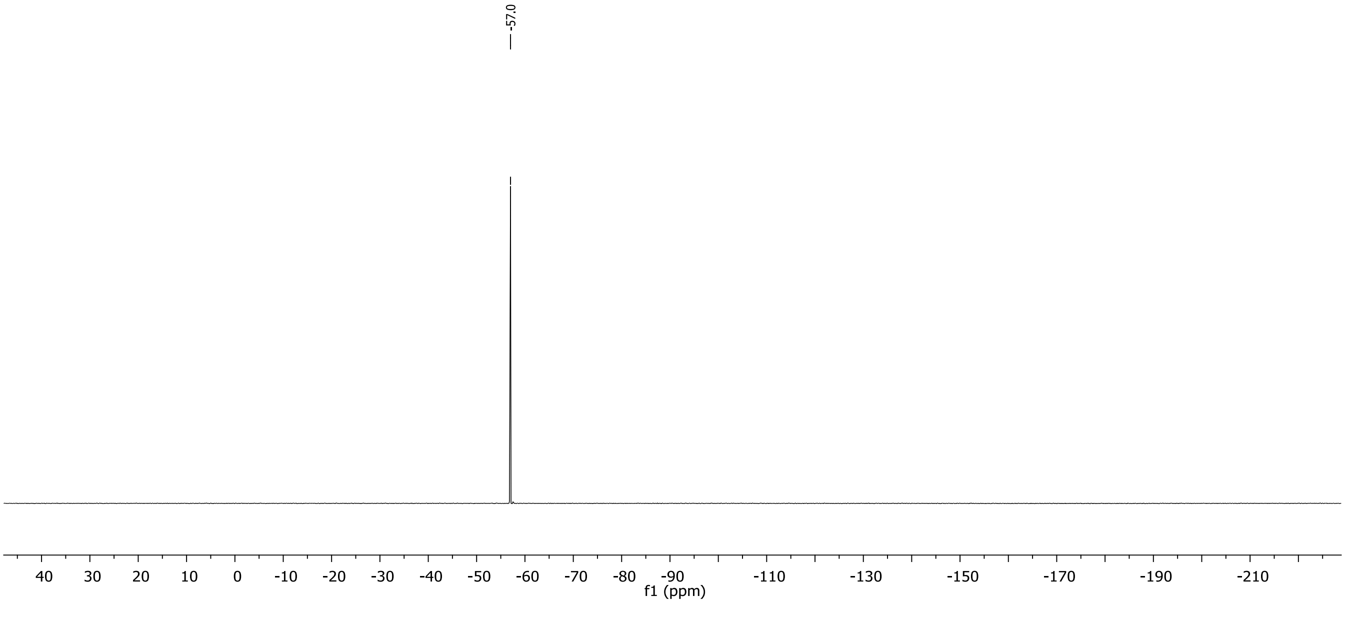

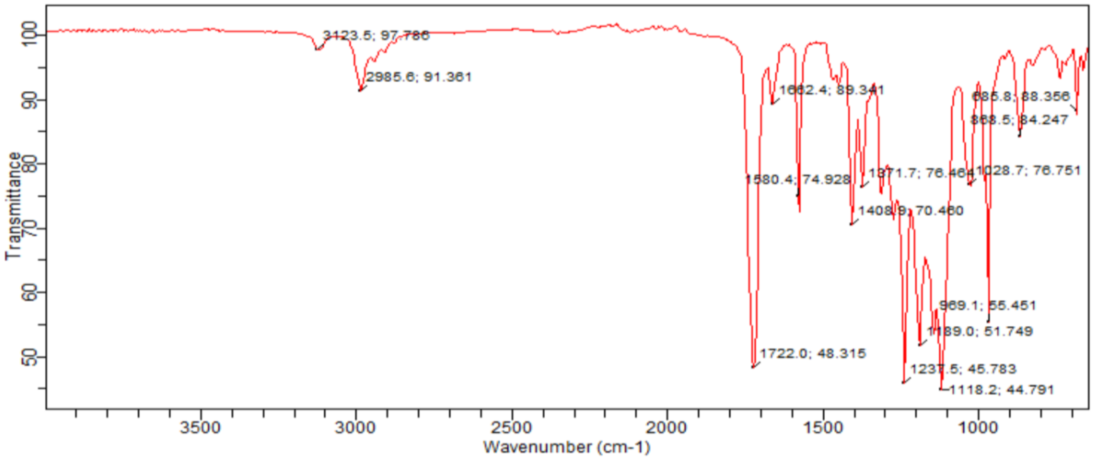

3123.5; 97.786
2985.6; 91.361
1662.4; 89.341
1580.4; 74.928
1371.7; 76.464
1408.9; 70.460
1028.7; 76.751
868.5; 84.247
969.1; 55.451
1189.0; 51.749
1722.0; 48.315
1237.5; 45.783
1118.2; 44.791
Transmittance
Wavenumber (cm-1)

Diethyl (*E*)-4-(4-(methoxycarbonyl)-1H-imidazol-1-yl)hex-2-enedioate (3ba) (^{1}H NMR: 300 MHz, ^{13}C NMR: 75 MHz, $CDCl_3$):

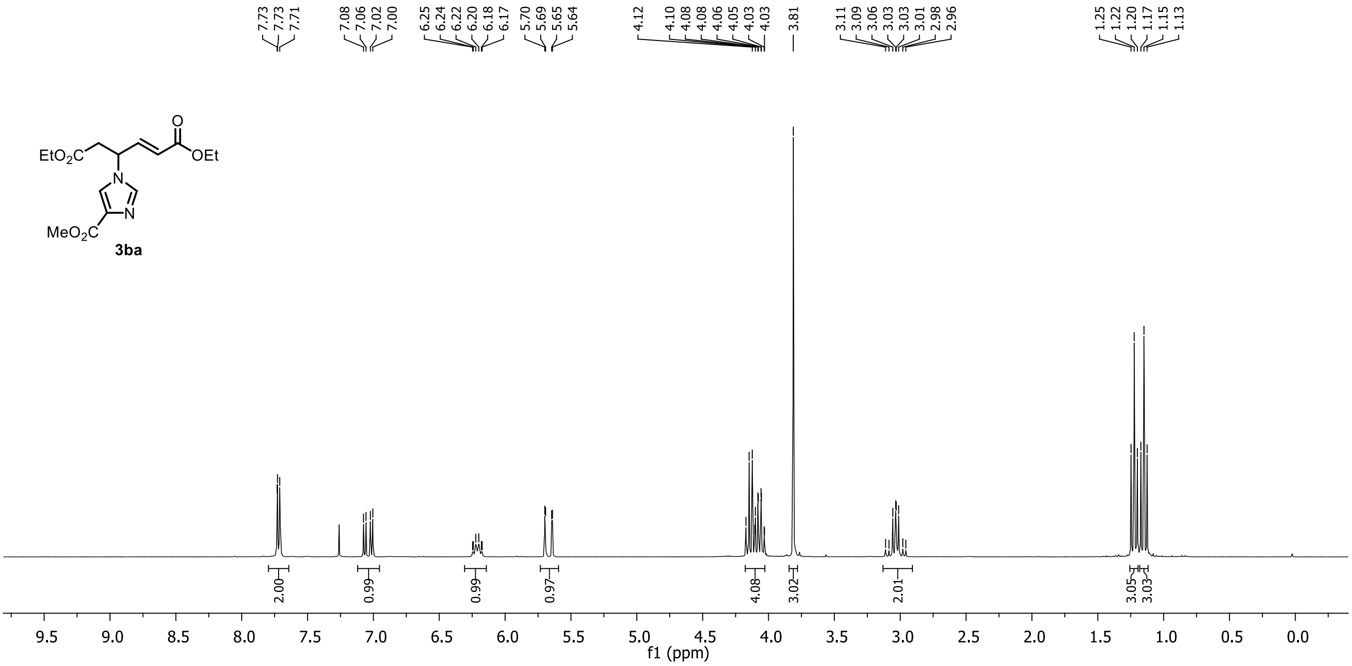

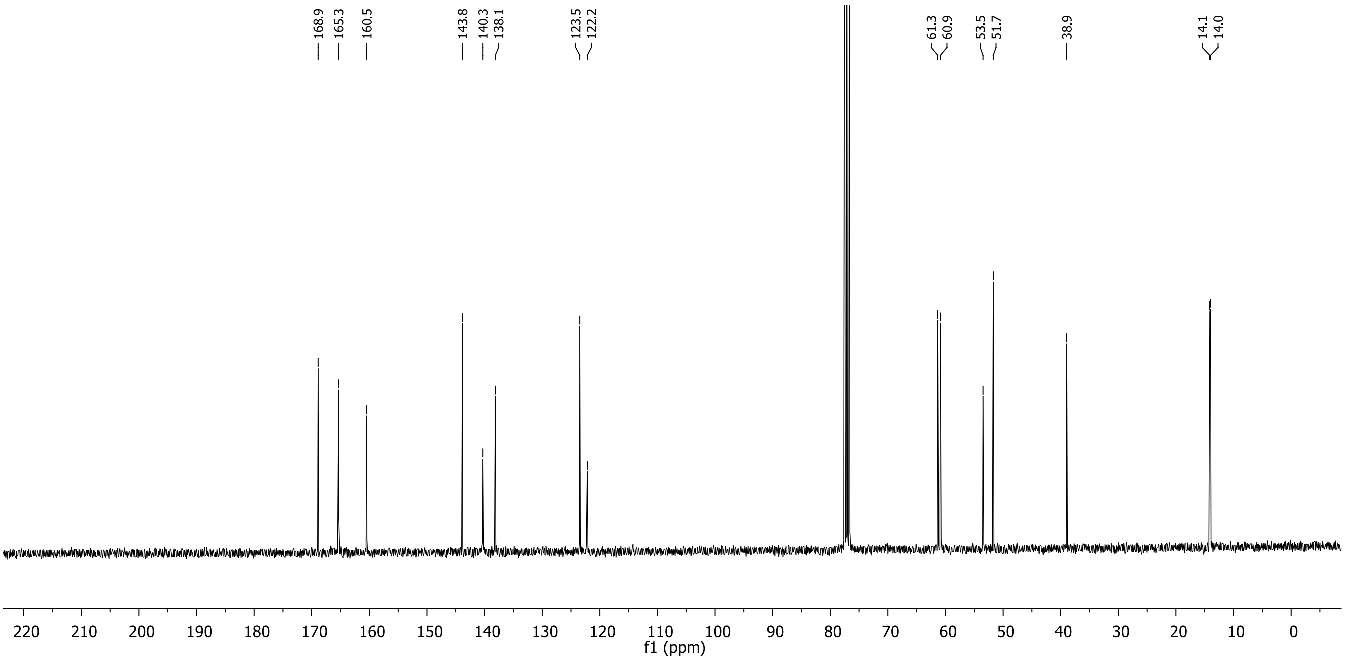

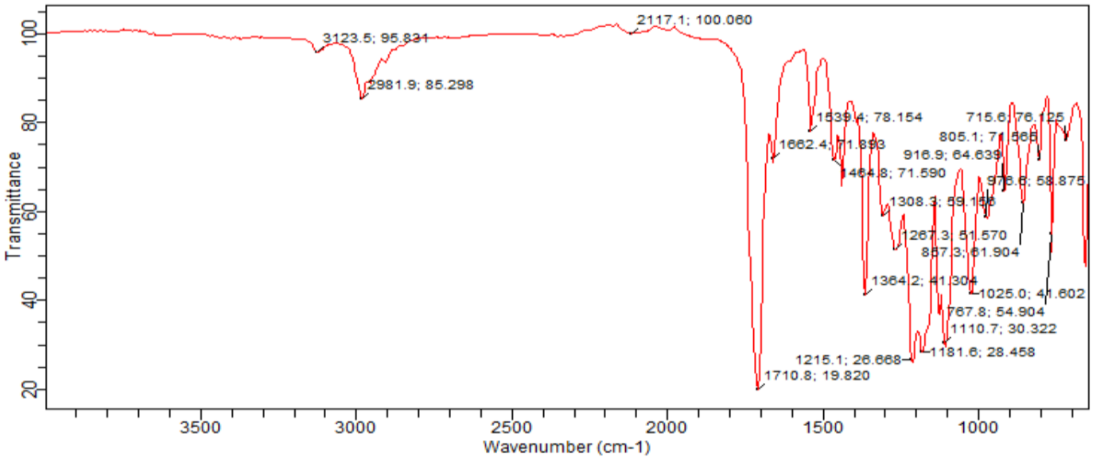

Diethyl (*E*)-4-(1H-1,2,4-triazol-1-yl)hex-2-enedioate (3bb) (^{1}H NMR: 300 MHz, ^{13}C NMR: 75 MHz, $CDCl_3$):

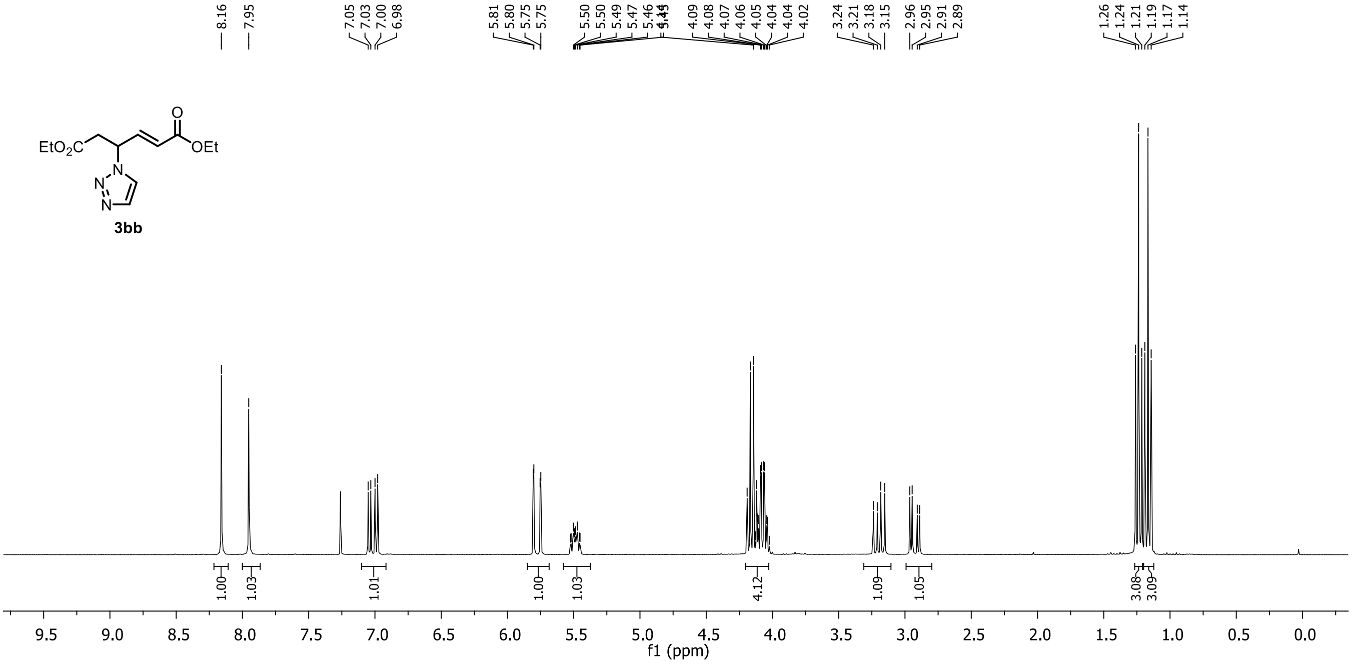

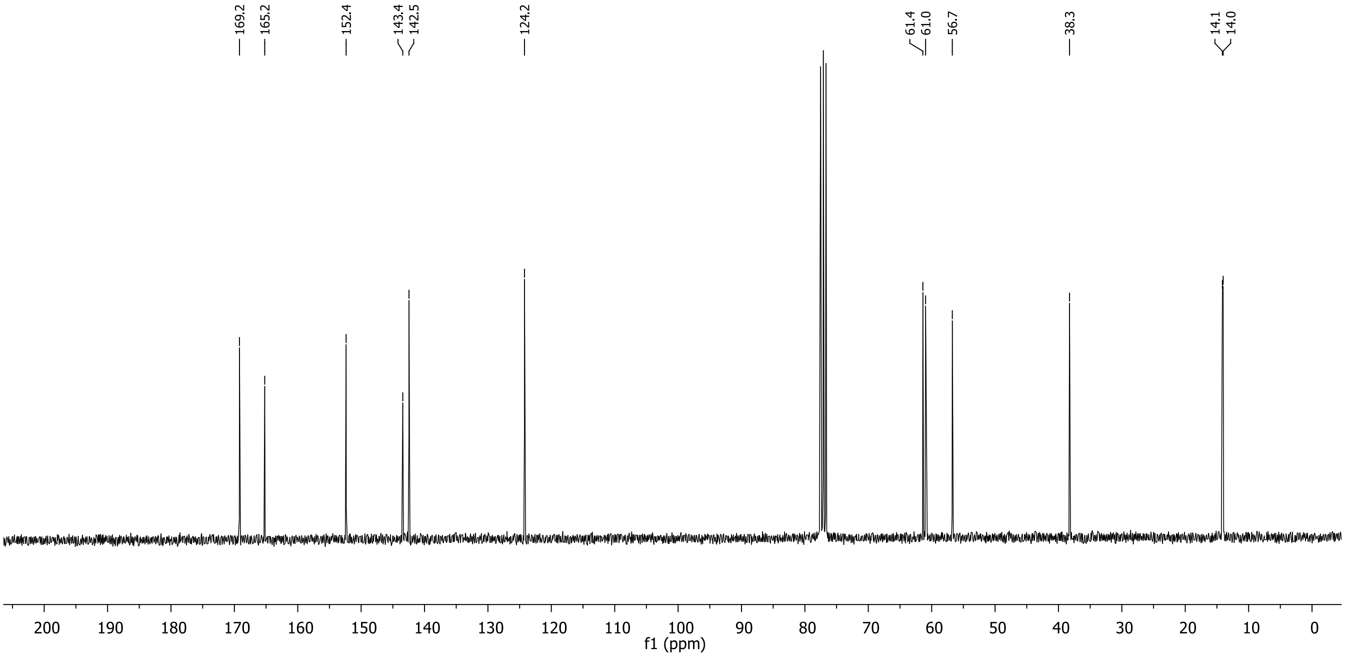

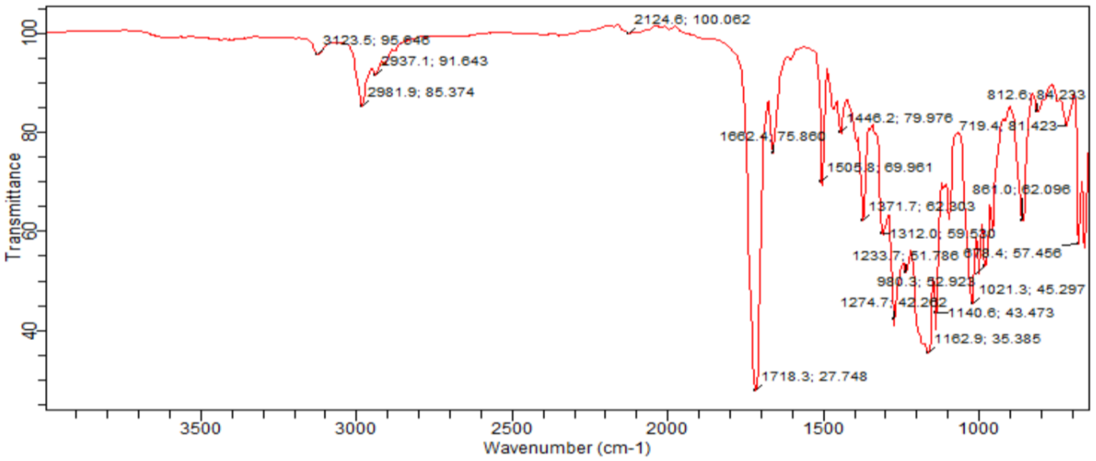

Diethyl (*E*)-4-(1H-1,2,3-triazol-1-yl)hex-2-enedioate (3bc) (^{1}H NMR: 300 MHz, ^{13}C NMR: 75 MHz, $CDCl_3$):

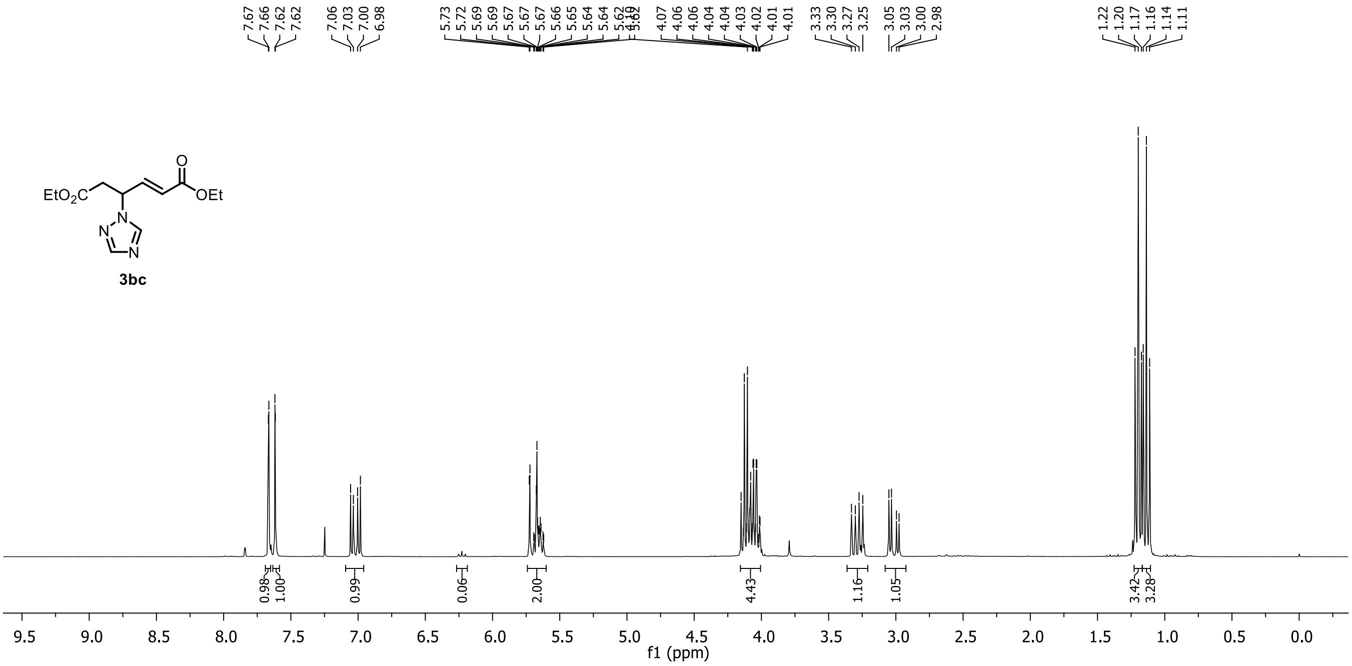

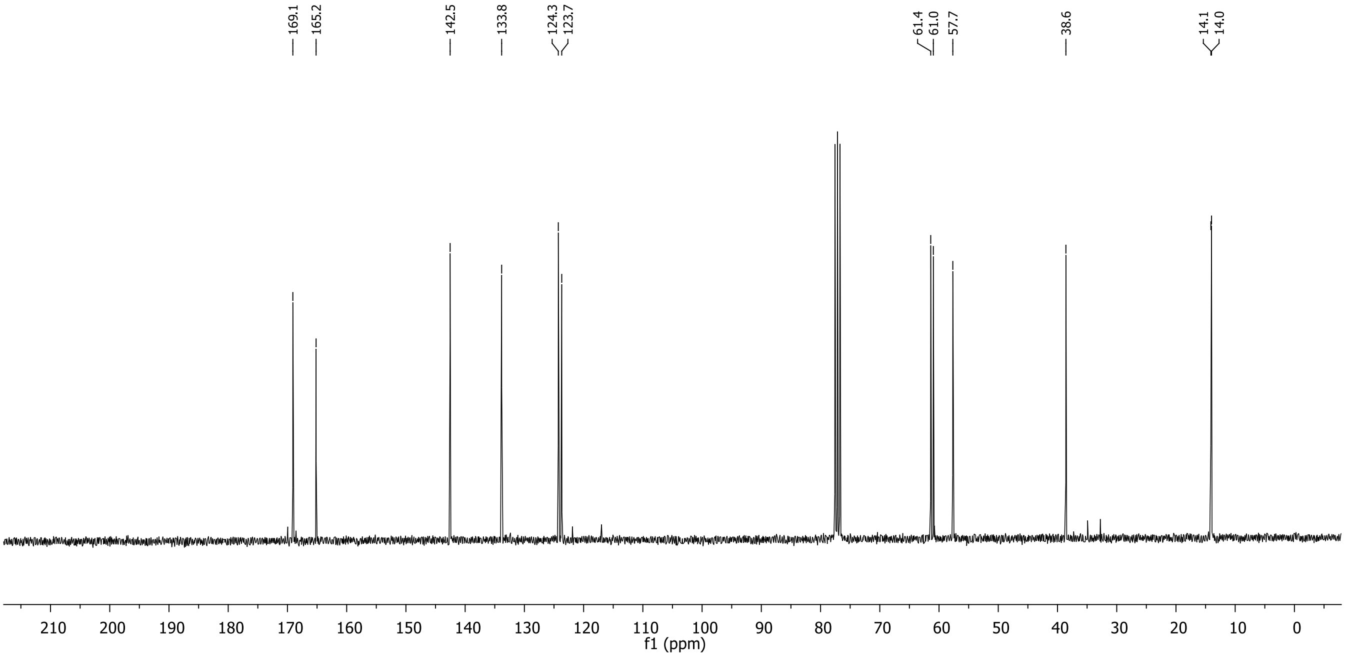

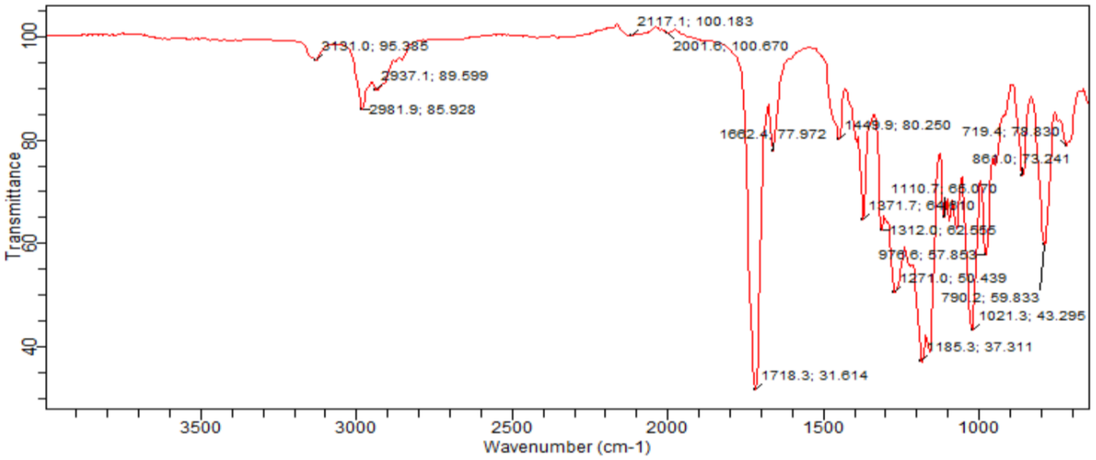

Diethyl (*E*)-4-(5-methyl-2H-tetrazol-2-yl)hex-2-enedioate (3bd) (^{1}H NMR: 400 MHz, ^{13}C NMR: 101 MHz, 2D NOESY NMR: 400 MHz, CDCl$_3$):

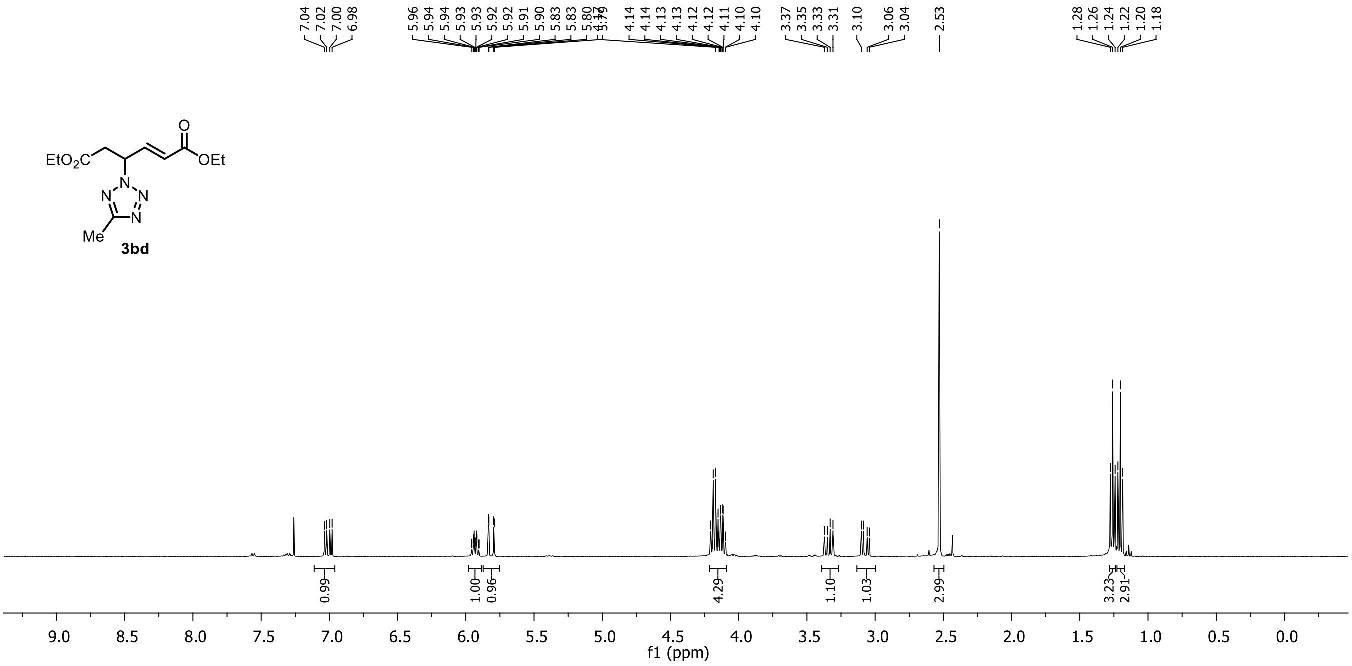

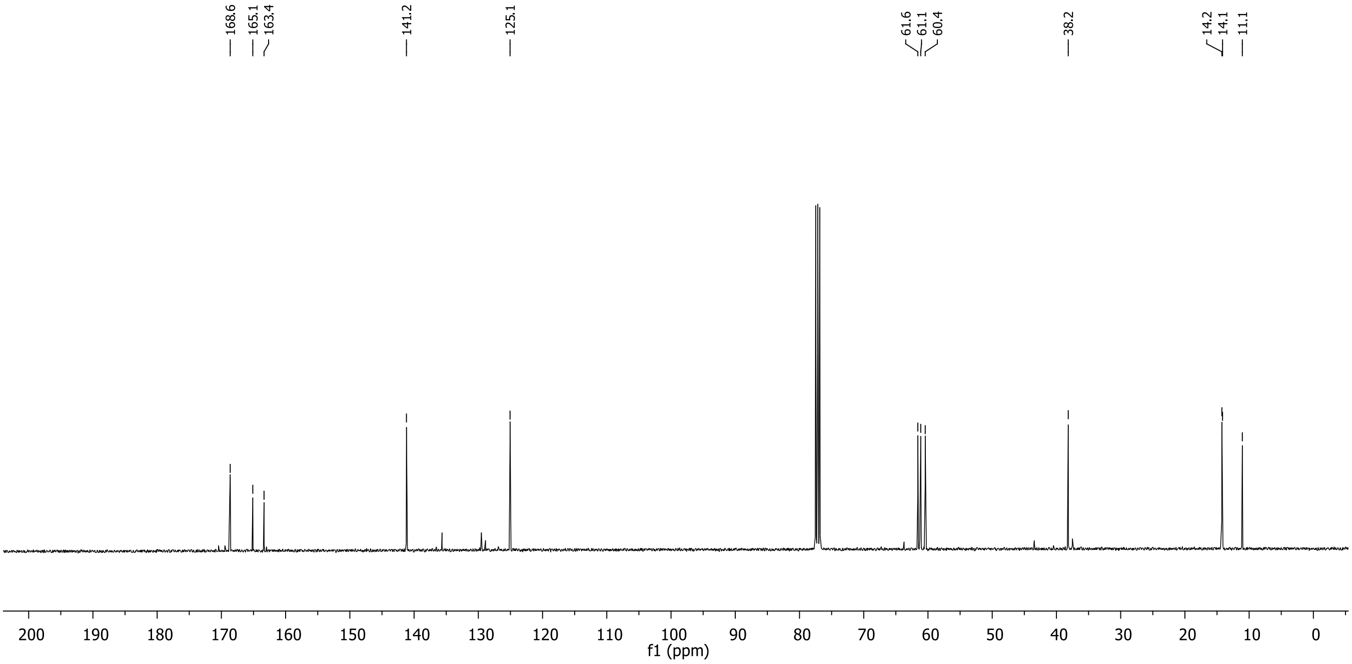

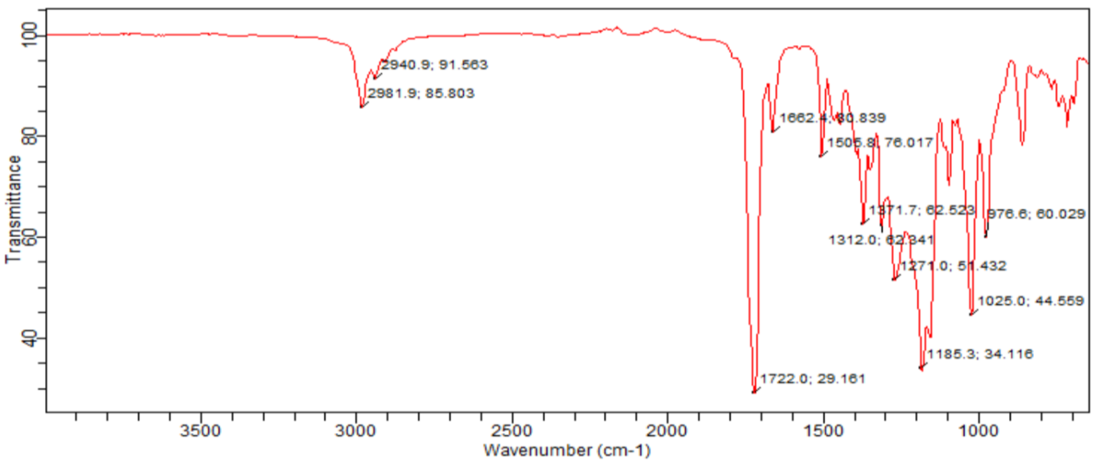

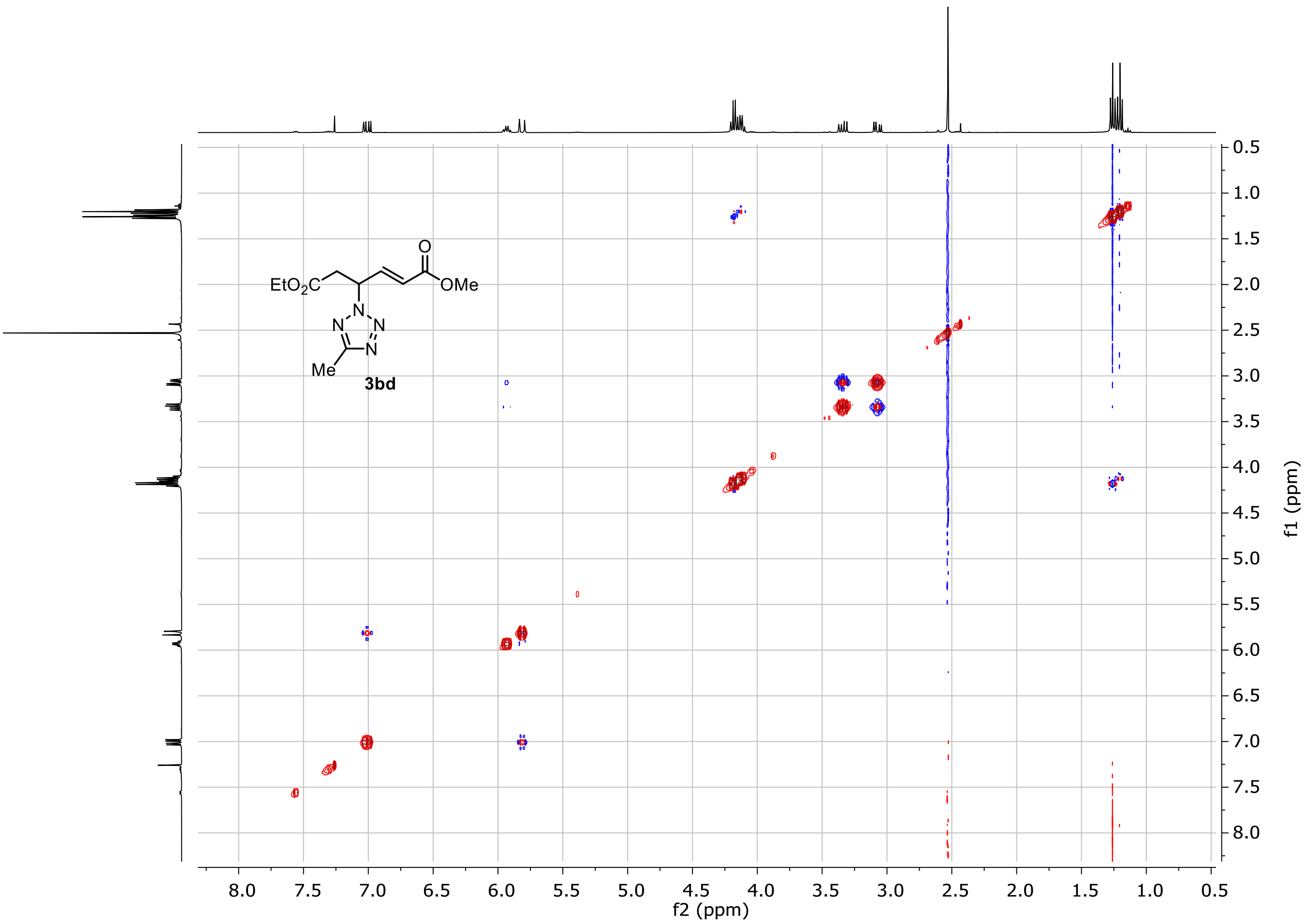
EtO2C
OMe
O
N
Me
3bd
f2 (ppm)
f1 (ppm)
8.0
7.5
7.0
6.5
6.0
5.5
5.0
4.5
4.0
3.5
3.0
2.5
2.0
1.5
1.0
0.5

Diethyl (*E*)-4-(5-phenyl-2H-tetrazol-2-yl)hex-2-enedioate (3be) (^{1}H NMR: 400 MHz, ^{13}C NMR: 101 MHz, 2D NOESY NMR: 400 MHz, $CDCl_3$):

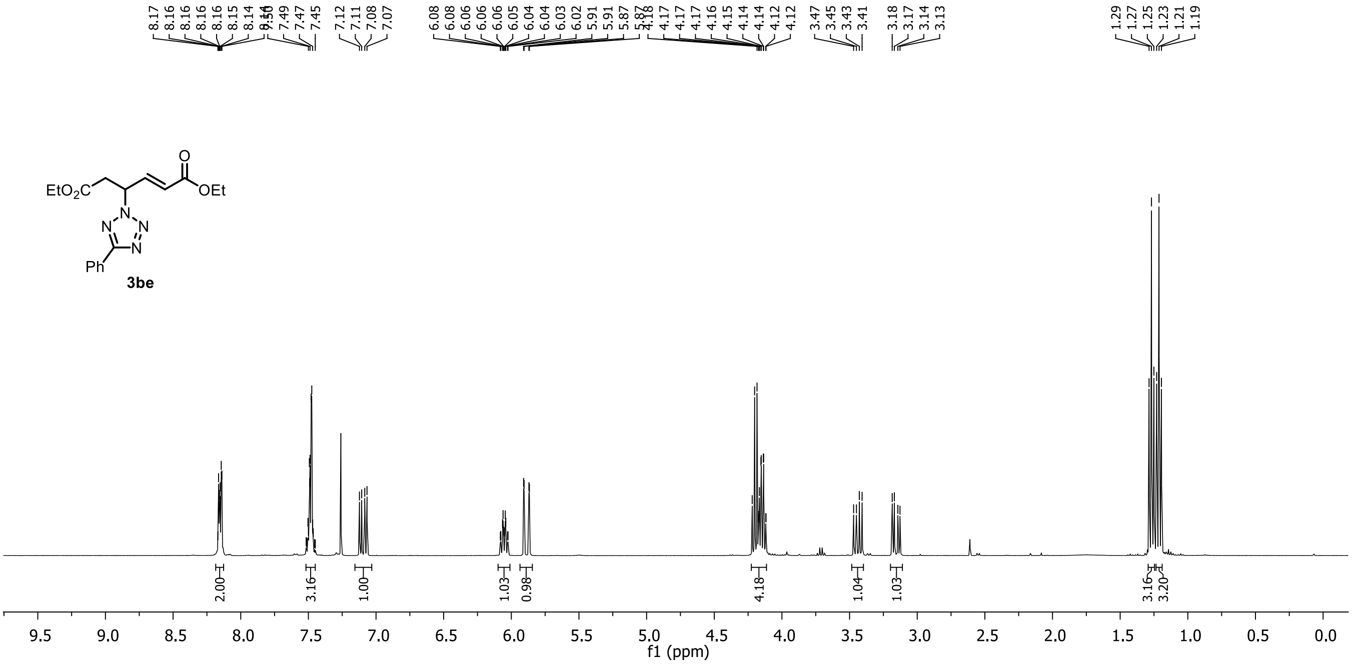

EtO2C
OEt
O
N
Ph
3be
2.00
3.16
1.00
1.03
0.98
4.18
1.04
1.03
3.16
3.20
f1 (ppm)

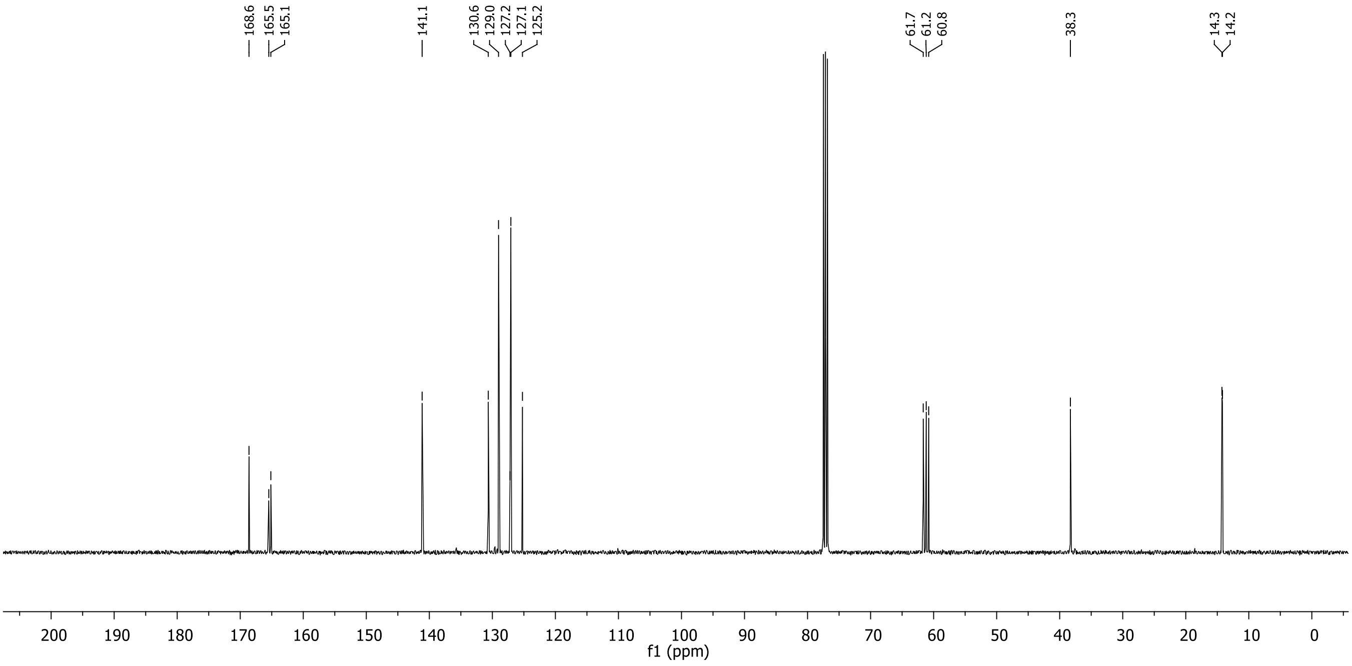

168.6
165.5
165.1
141.1
130.6
129.0
127.2
127.1
125.2
61.7
61.2
60.8
38.3
14.3
14.2
f1 (ppm)

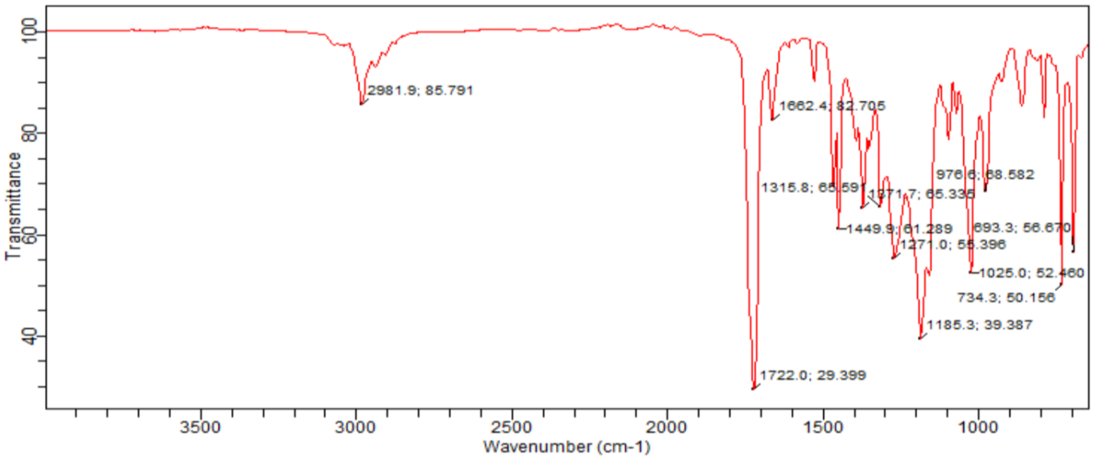

Transmittance
Wavenumber (cm-1)
2981.9; 85.791
1662.4; 82.705
1722.0; 29.399
1185.3; 39.387
734.3; 50.156
1025.0; 52.460

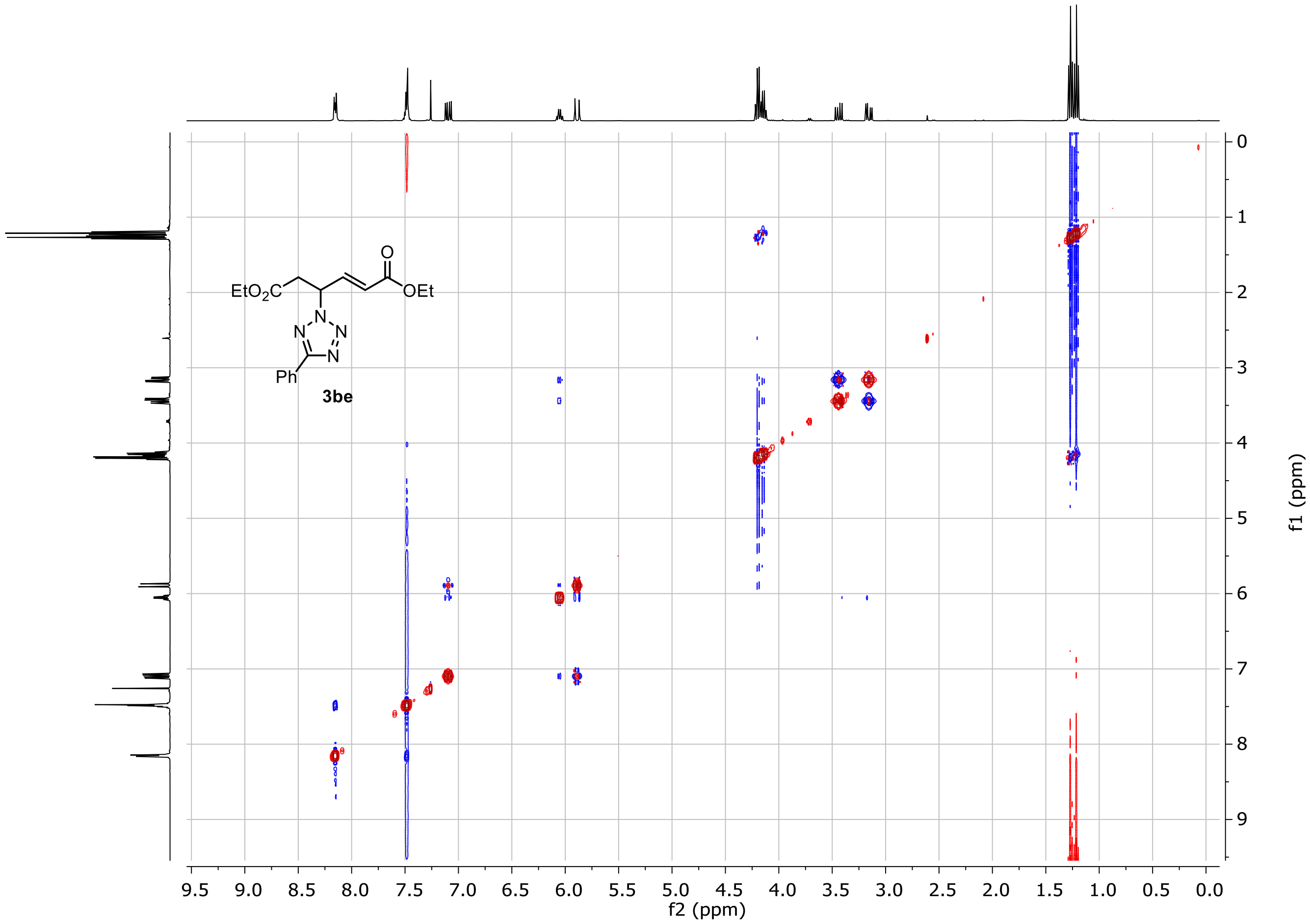
EtO2C
O
OEt
N
N
N
N
Ph
3be
9.5 9.0 8.5 8.0 7.5 7.0 6.5 6.0 5.5 5.0 4.5 4.0 3.5 3.0 2.5 2.0 1.5 1.0 0.5 0.0
f2 (ppm)
0 1 2 3 4 5 6 7 8 9
f1 (ppm)

Diethyl (*E*)-4-(3-(methoxycarbonyl)-1H-indazol-1-yl)hex-2-enedioate (3af) (^{1}H NMR: 300 MHz, ^{13}C NMR: 75 MHz, $CDCl_3$):

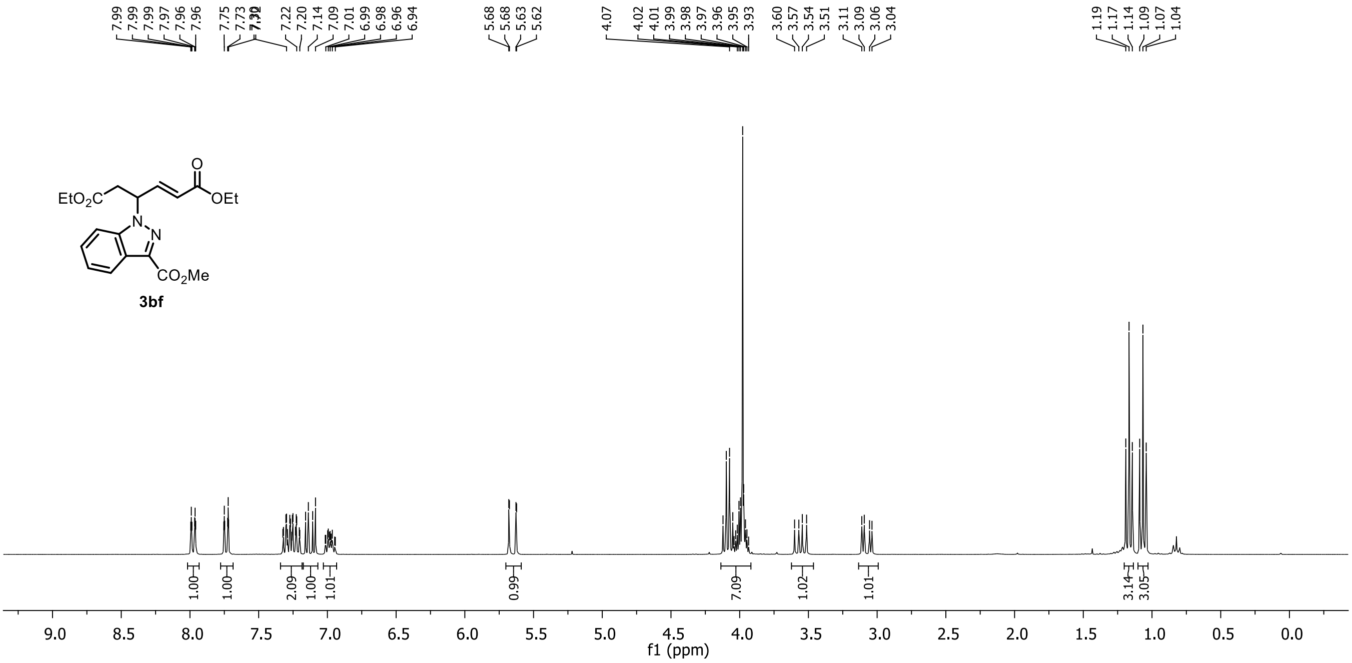

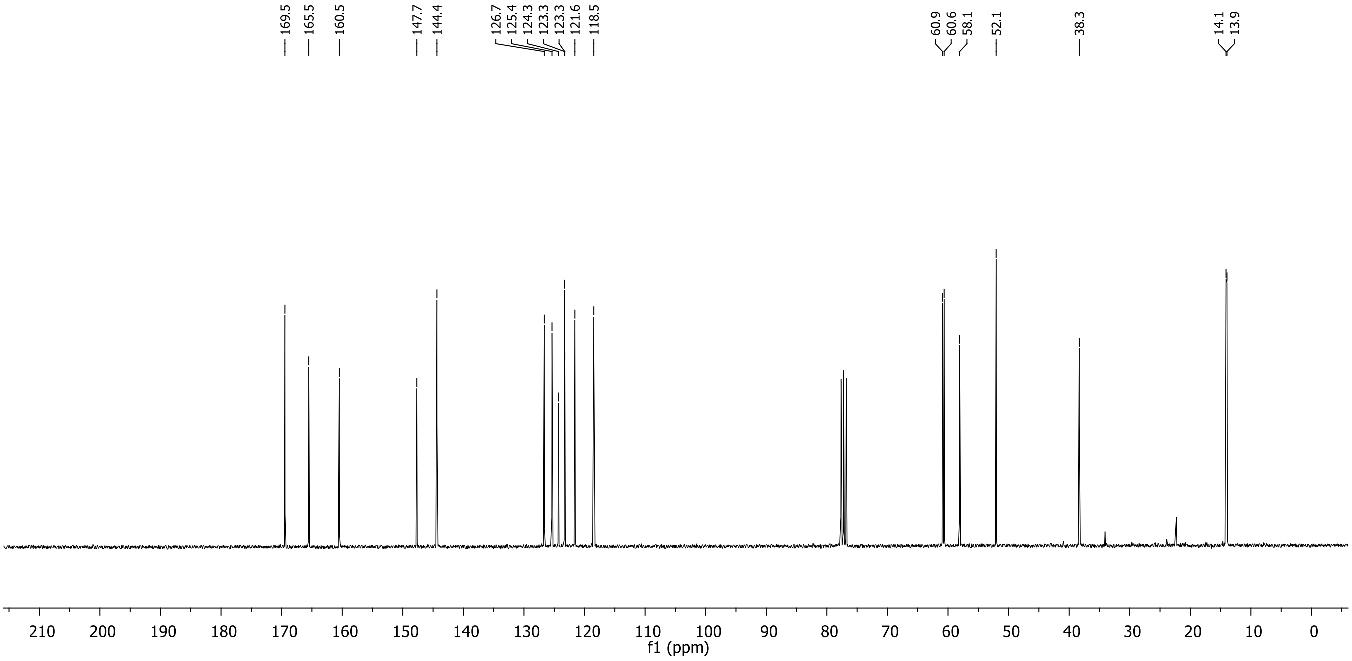

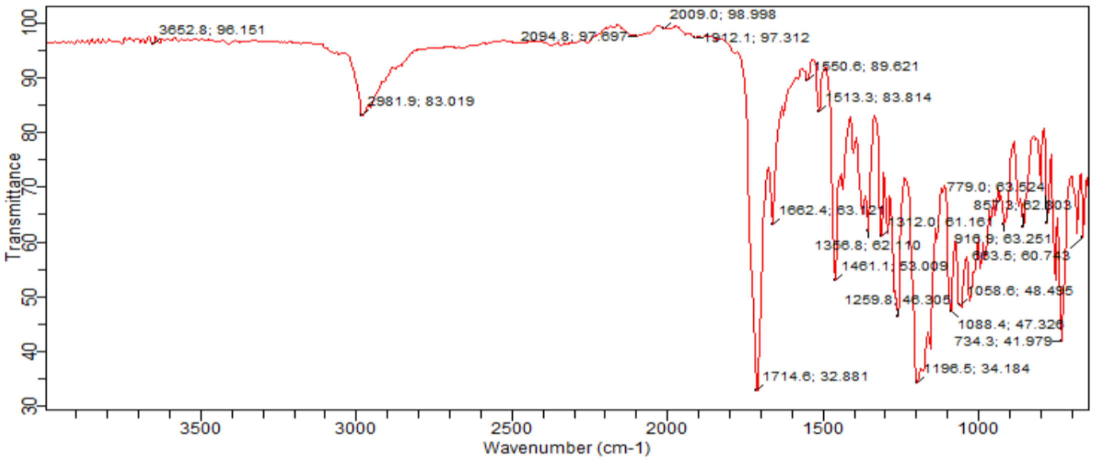

Diethyl (*E*)-4-(2-(methoxycarbonyl)-1H-benzo[d]imidazol-1-yl)hex-2-enedioate (3bg) (^{1}H NMR: 300 MHz, ^{13}C NMR: 75 MHz, $CDCl_3$):

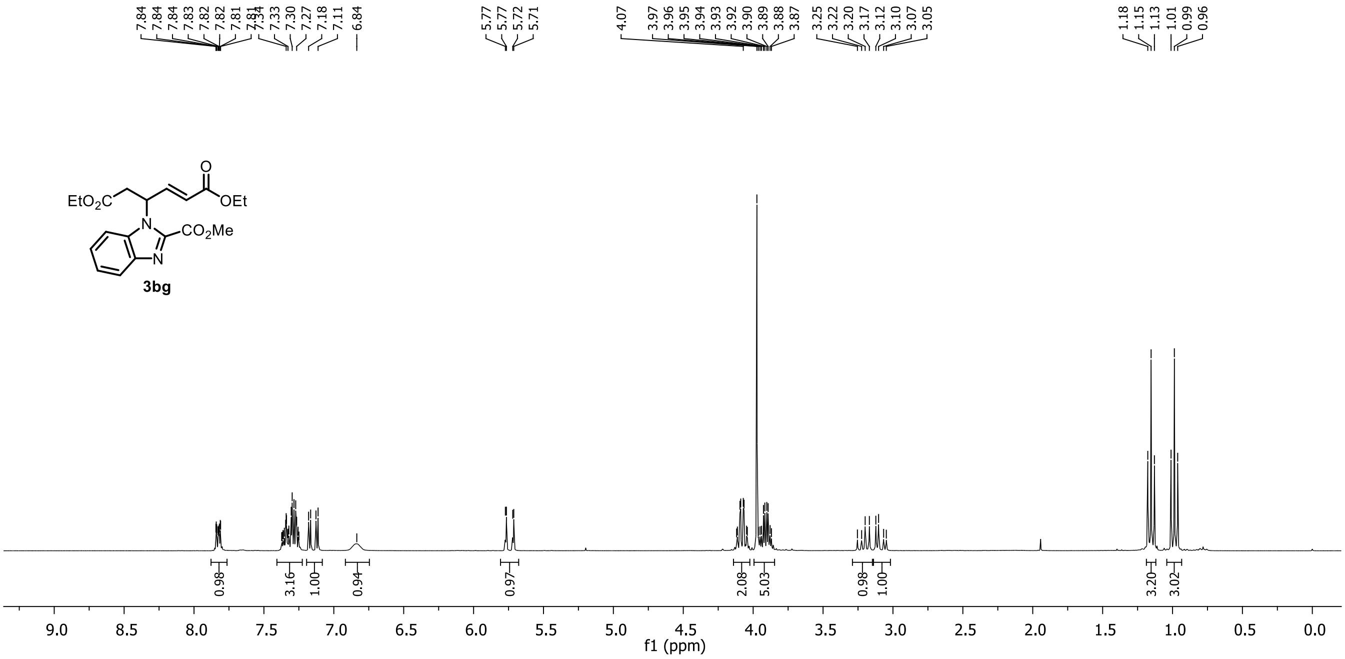

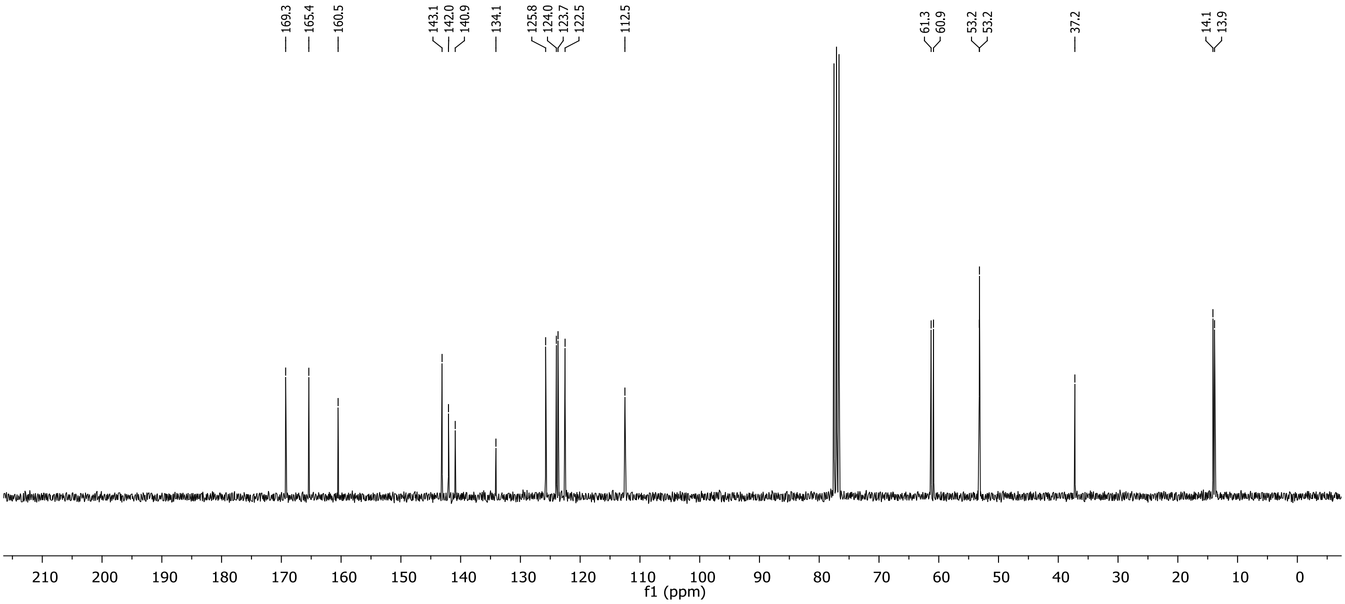

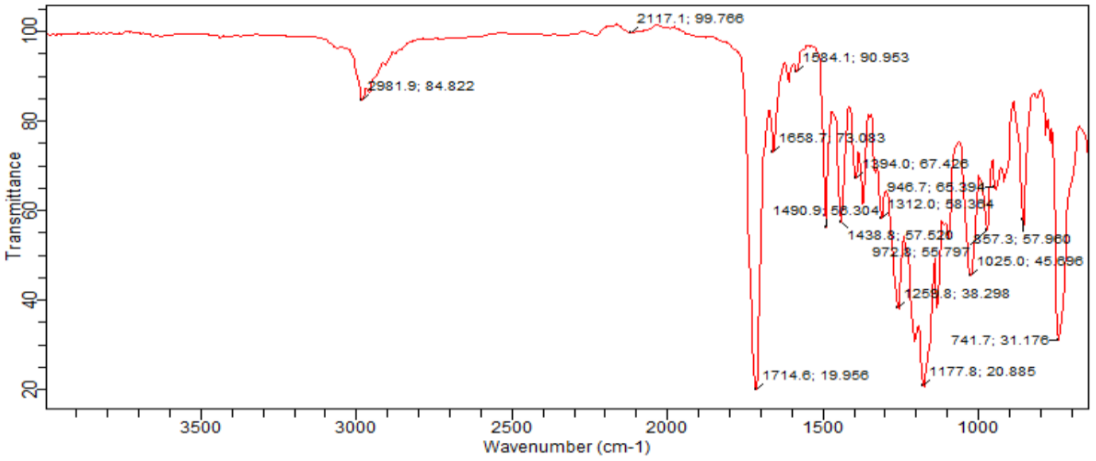

Diethyl (*E*)-4-(1H-benzo[d][1,2,3]triazol-1-yl)hex-2-enedioate (3bh) (^{1}H NMR: 400 MHz, ^{13}C NMR: 101 MHz, $CDCl_3$):

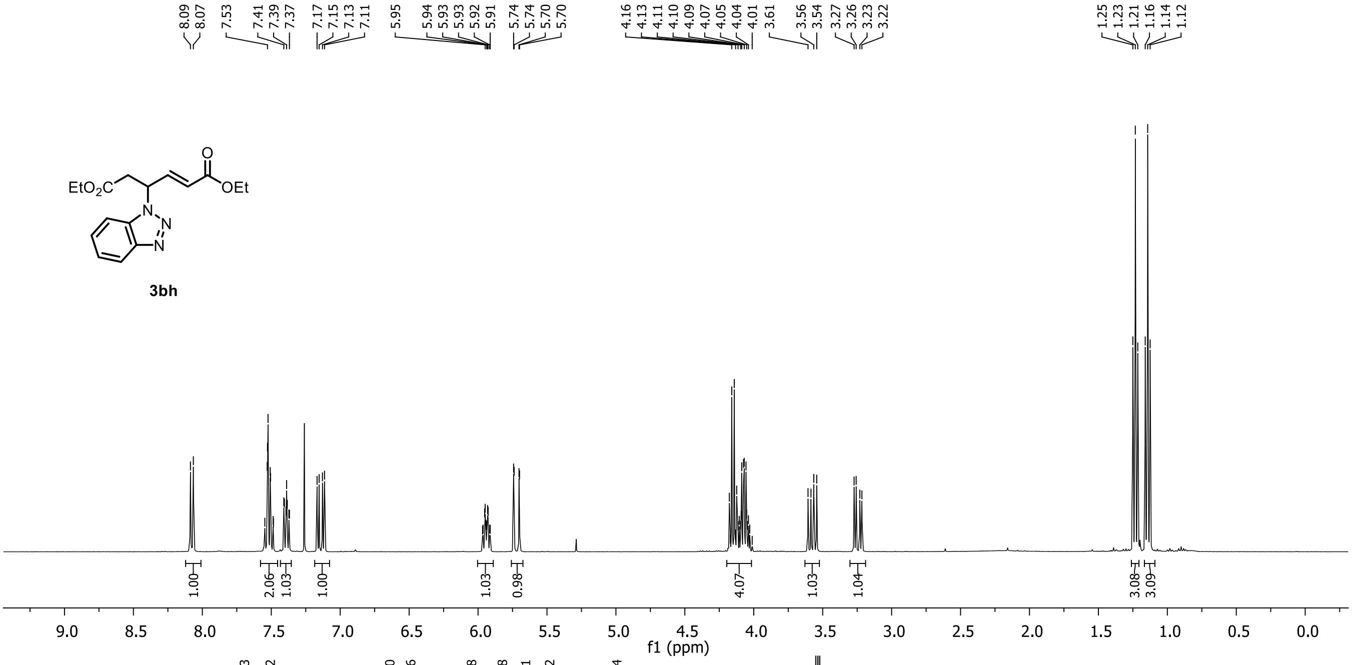

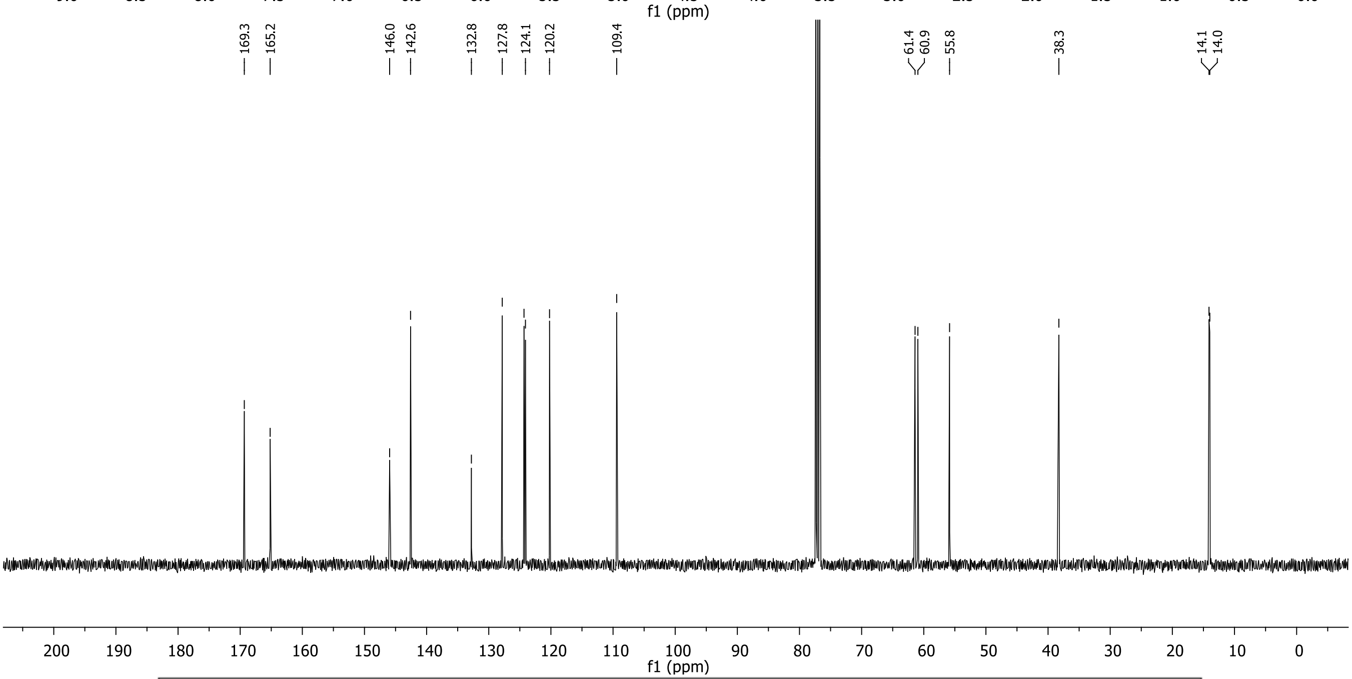

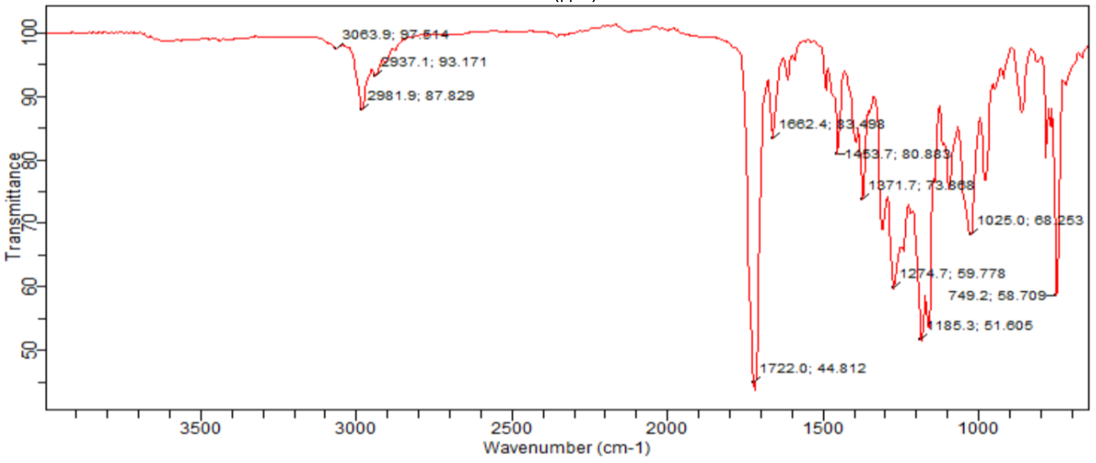

Diethyl (*E*)-4-(4-oxoquinazolin-3(4H)-yl)hex-2-enedioate (3bi) (^{1}H NMR: 300 MHz, ^{13}C NMR: 75 MHz, $CDCl_3$):

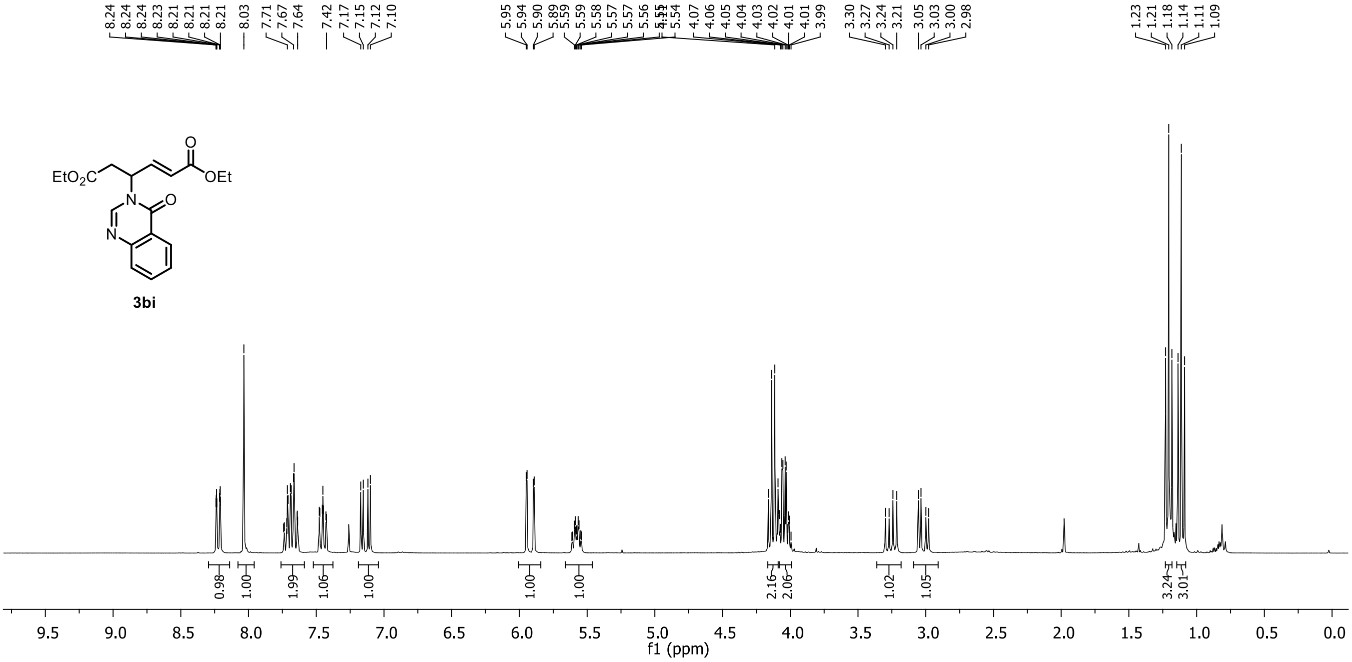

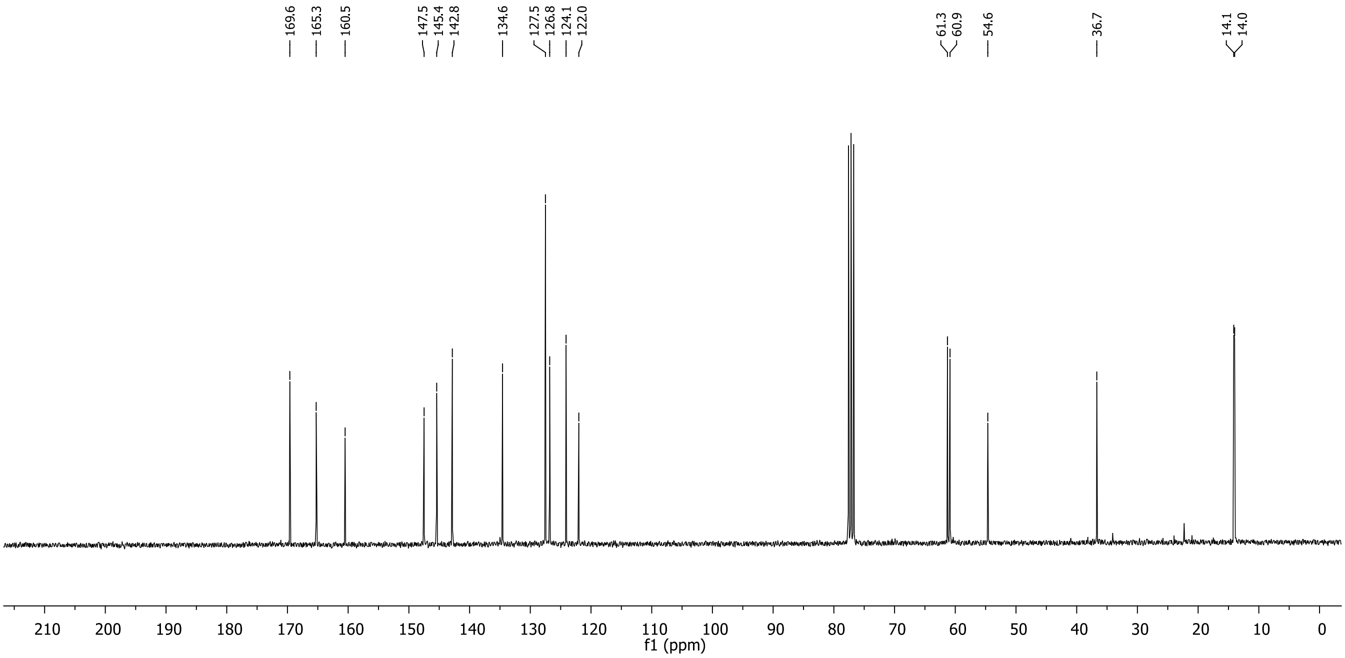

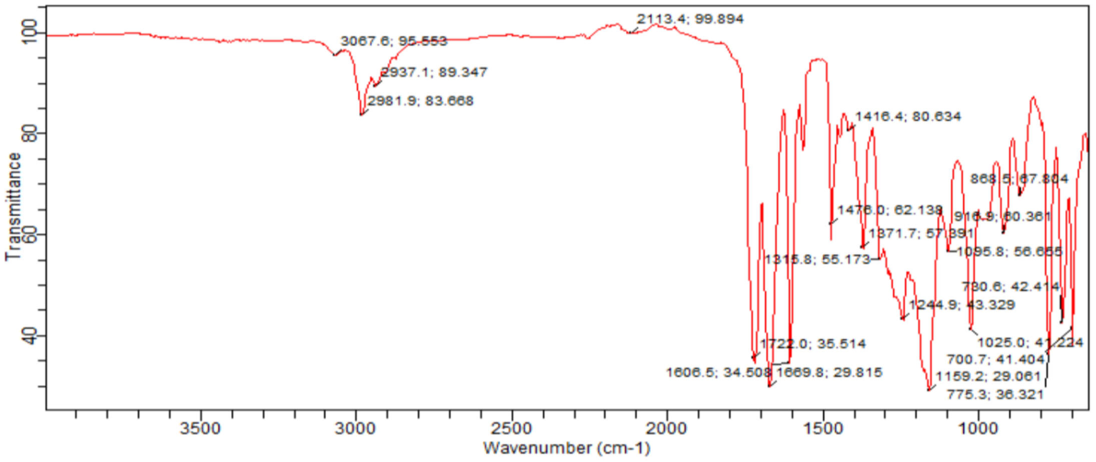

Diethyl (*E*)-4-(4-oxobenzo[d][1,2,3]triazin-3(4H)-yl)hex-2-enedioate (3bj) (^{1}H NMR: 300 MHz, ^{13}C NMR: 75 MHz, $CDCl_3$):

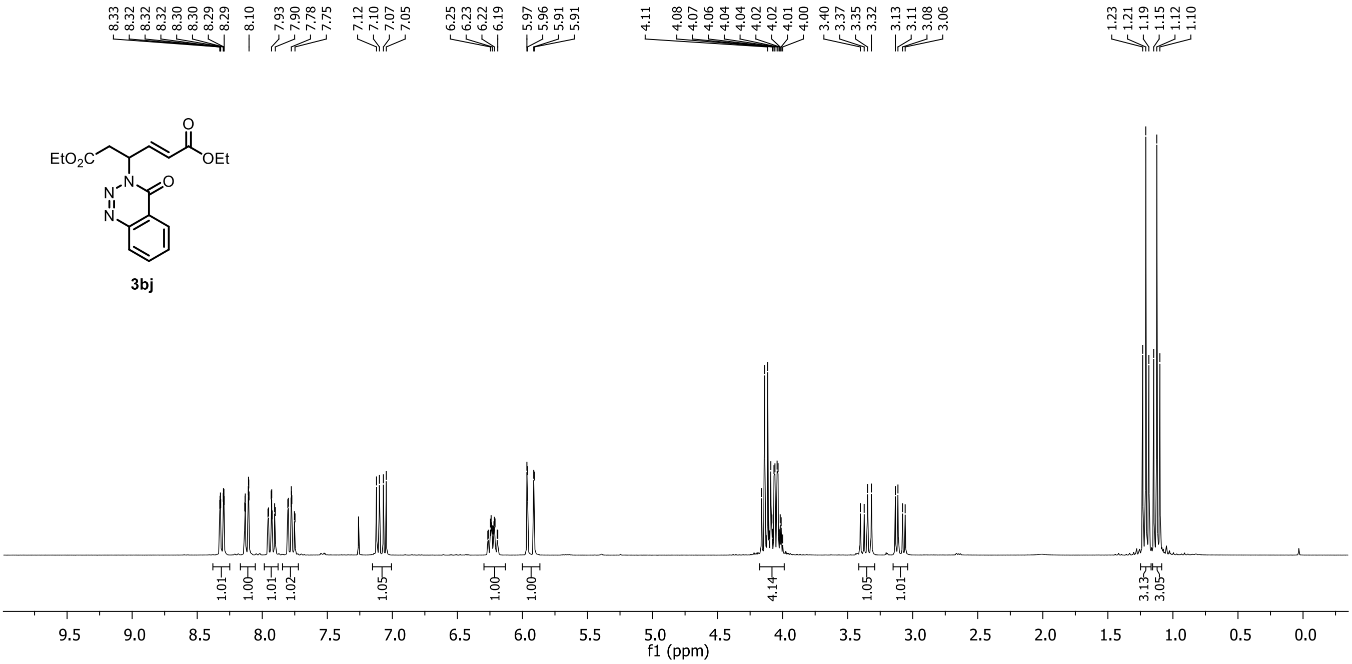

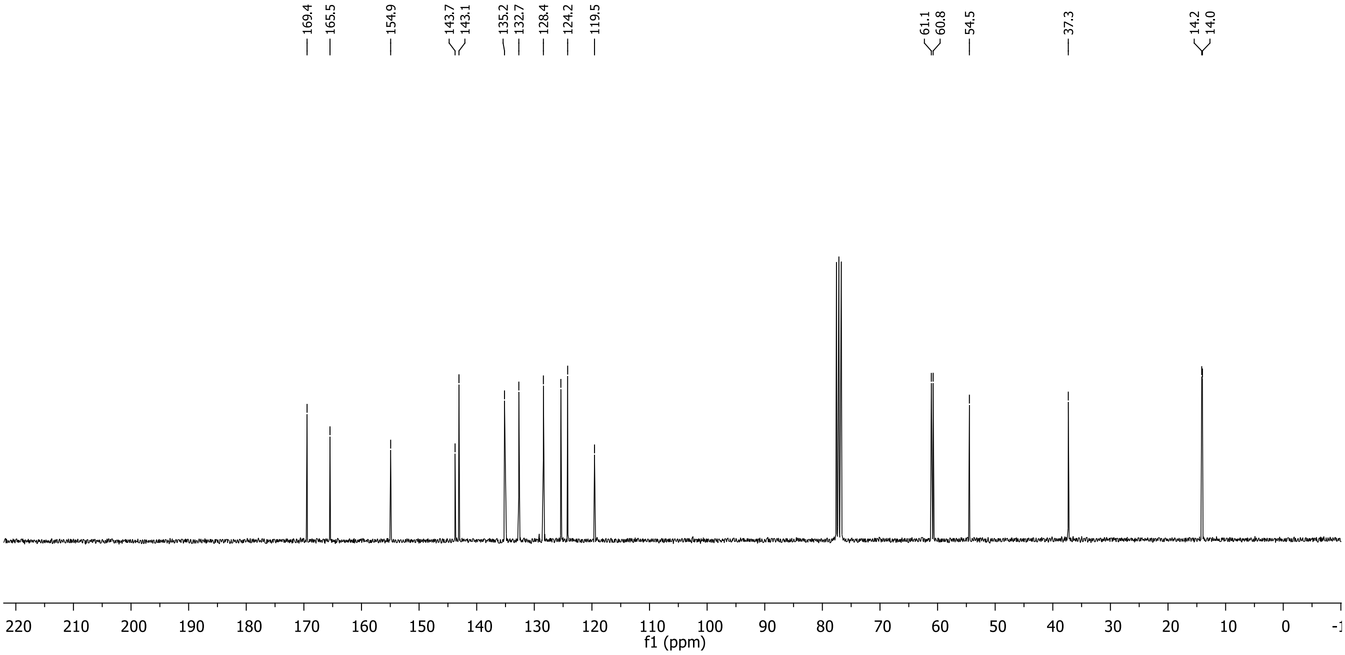

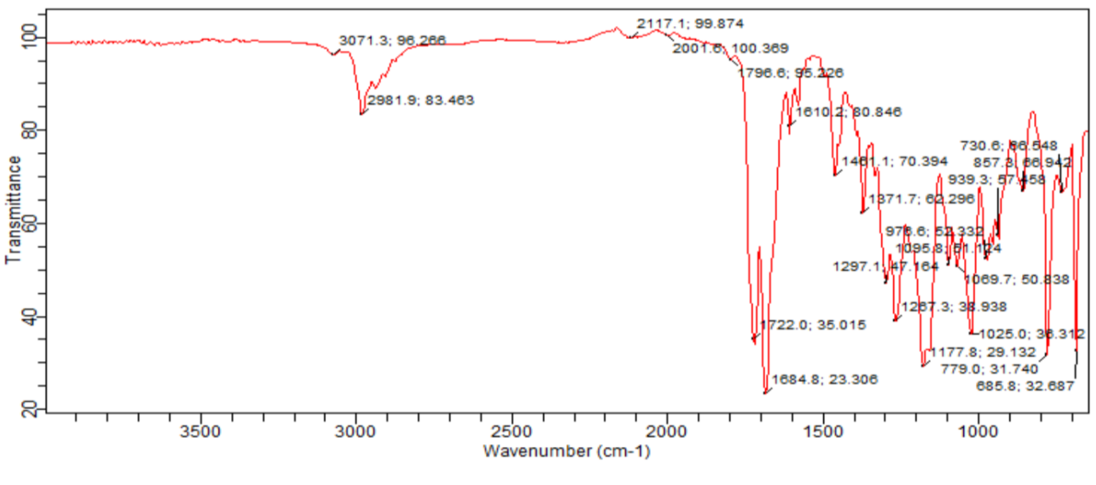

(8R,9S,10R,13S,14S,17S)-10,13-Dimethyl-3-oxo-2,3,6,7,8,9,10,11,12,13,14,15,16,17-tetradecahydro-1H-cyclopenta[a]phenanthren-17-yl (E)-4-(4-chloro-1H-pyrazol-1-yl)hex-2-enoate (3bk) (^{1}H NMR: 300 MHz, ^{13}C NMR: 75 MHz, $CDCl_3$):

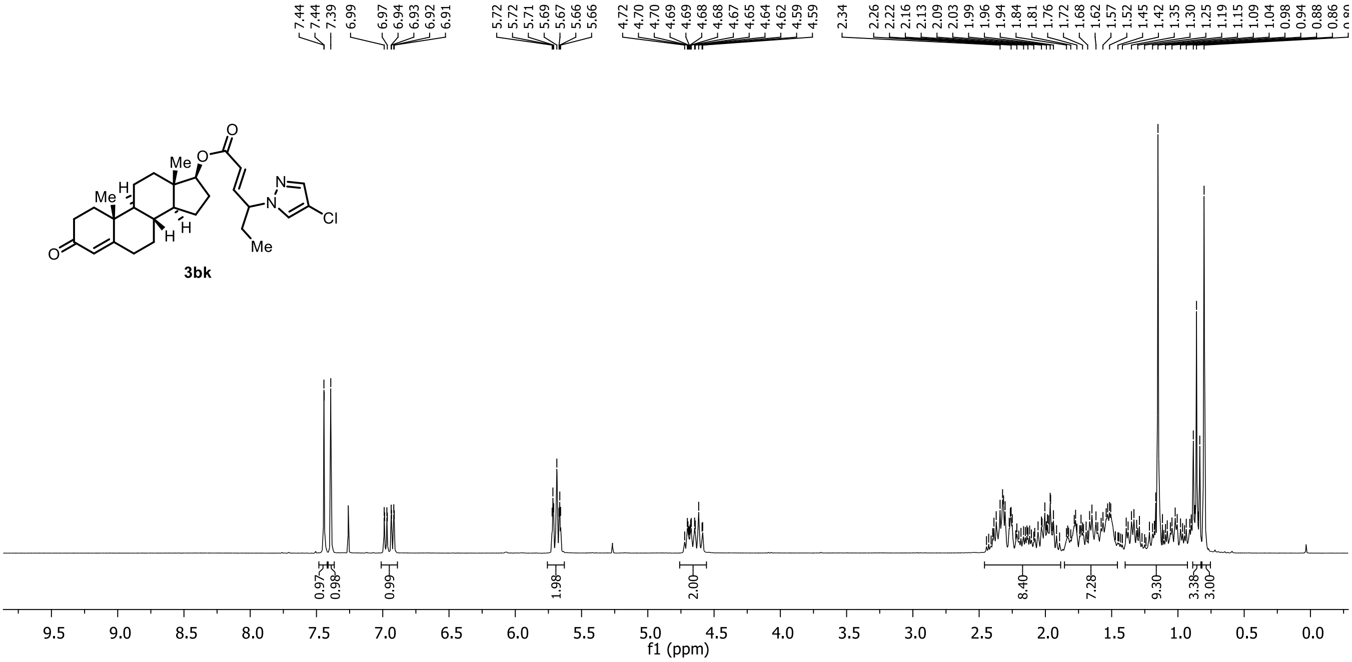

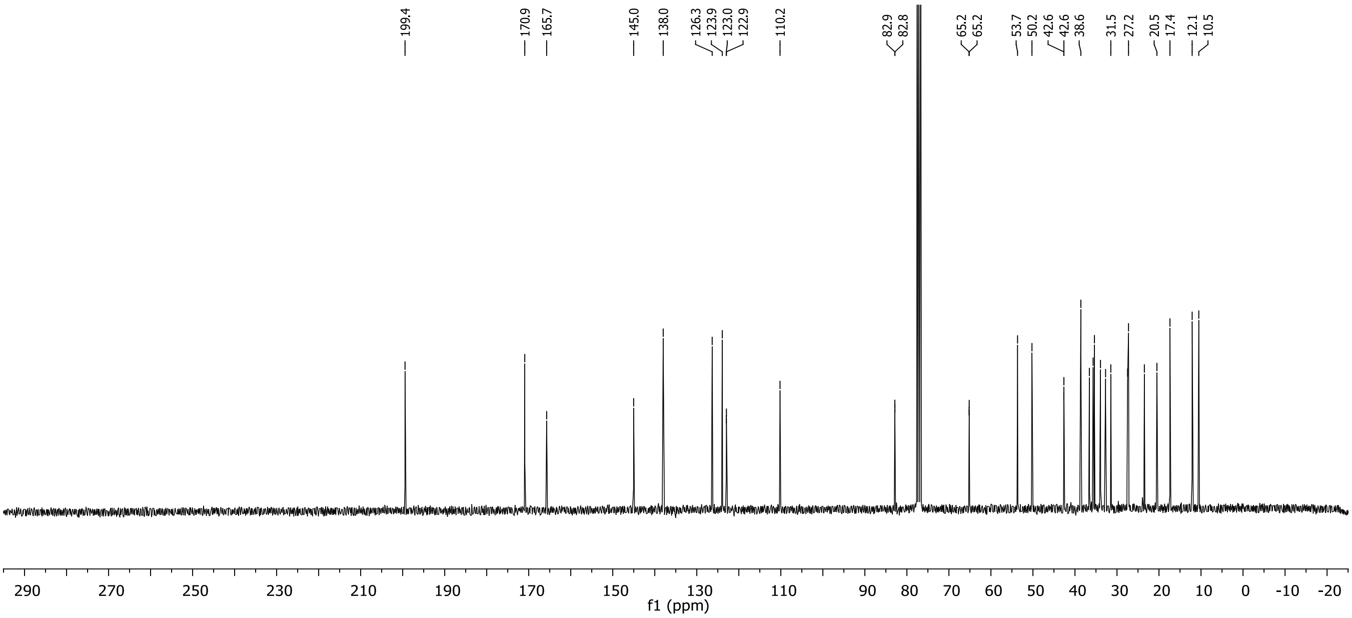

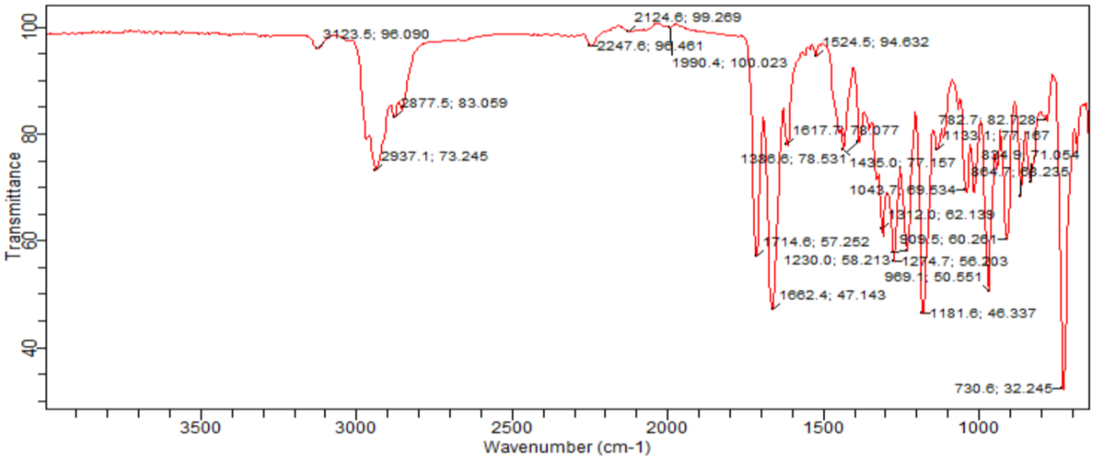

(8*R*,9*S*,10*R*,13*S*,14*S*,17*S*)-10,13-Dimethyl-3-oxo-2,3,6,7,8,9,10,11,12,13,14,15,16,17-tetradecahydro-1H-cyclopenta[a]phenanthren-17-yl (*E*)-4-(1H-benzo[d][1,2,3]triazol-1-yl)hex-2-enoate (3bl) (^{1}H NMR: 300 MHz, ^{13}C NMR: 75 MHz, $CDCl_3$):

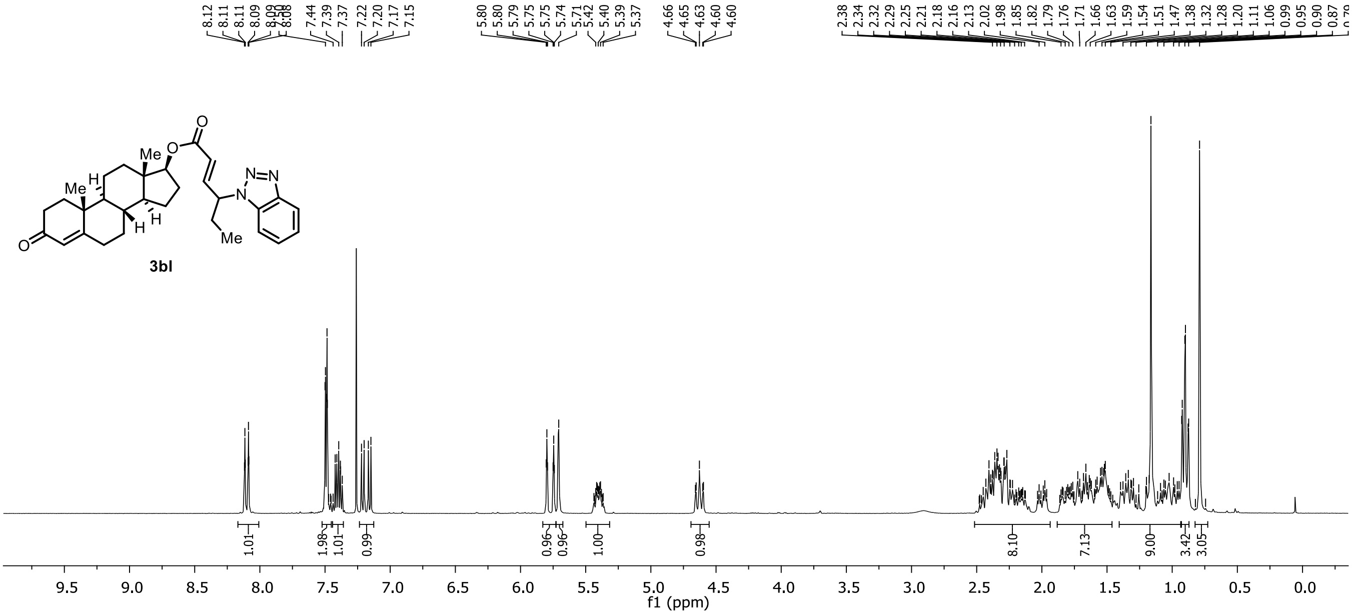

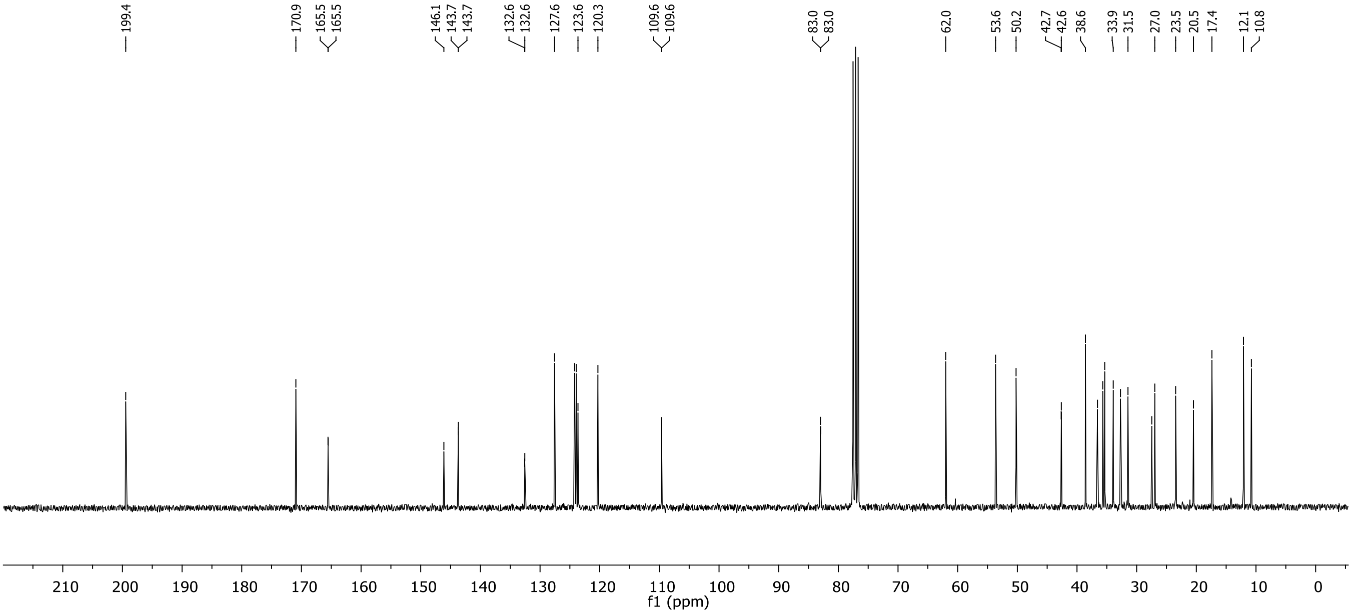

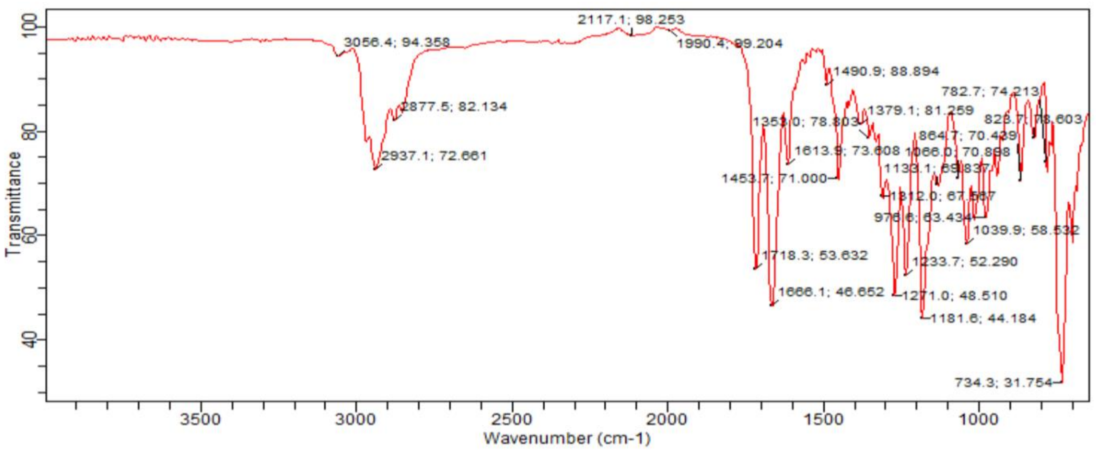

Diethyl (*E*)-4-(5-(4'-((2-butyl-4-oxo-1,3-diazaspiro[4.4]non-1-en-3-yl)methyl)-[1,1'-biphenyl]-2-yl)-2H-tetrazol-2-yl)hex-2-enedioate (3bm) (^{1}H NMR: 400 MHz, ^{13}C NMR: 101 MHz, $CDCl_3$):

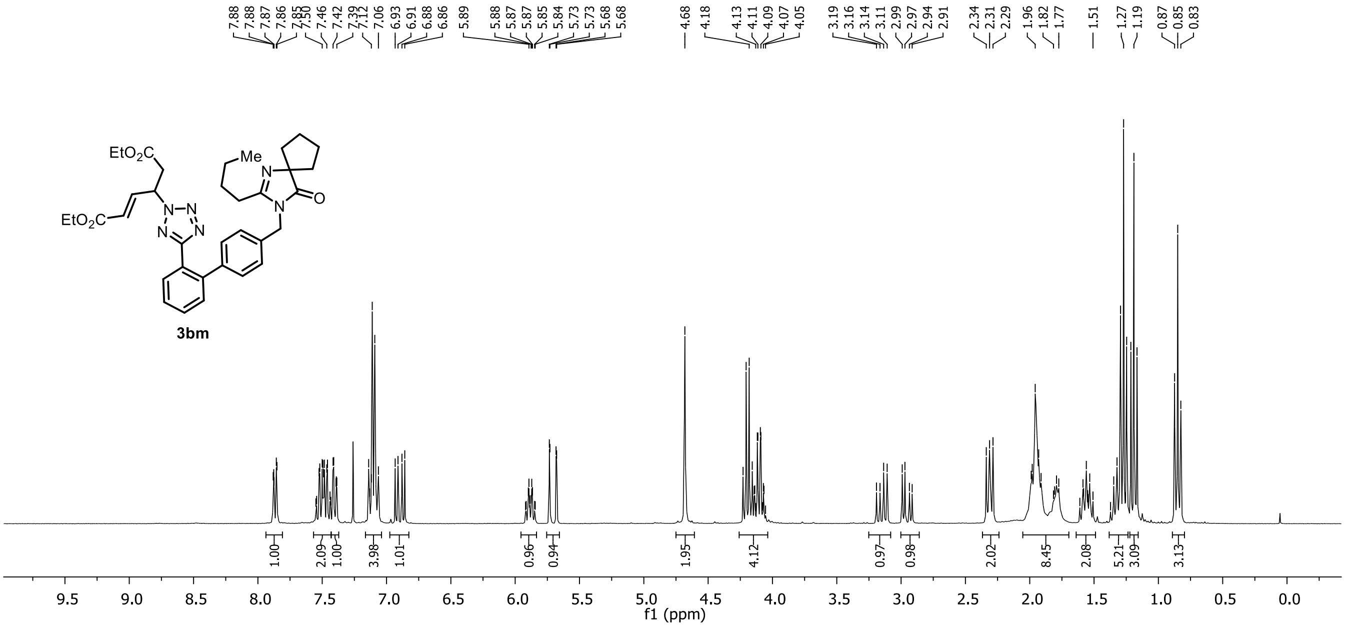

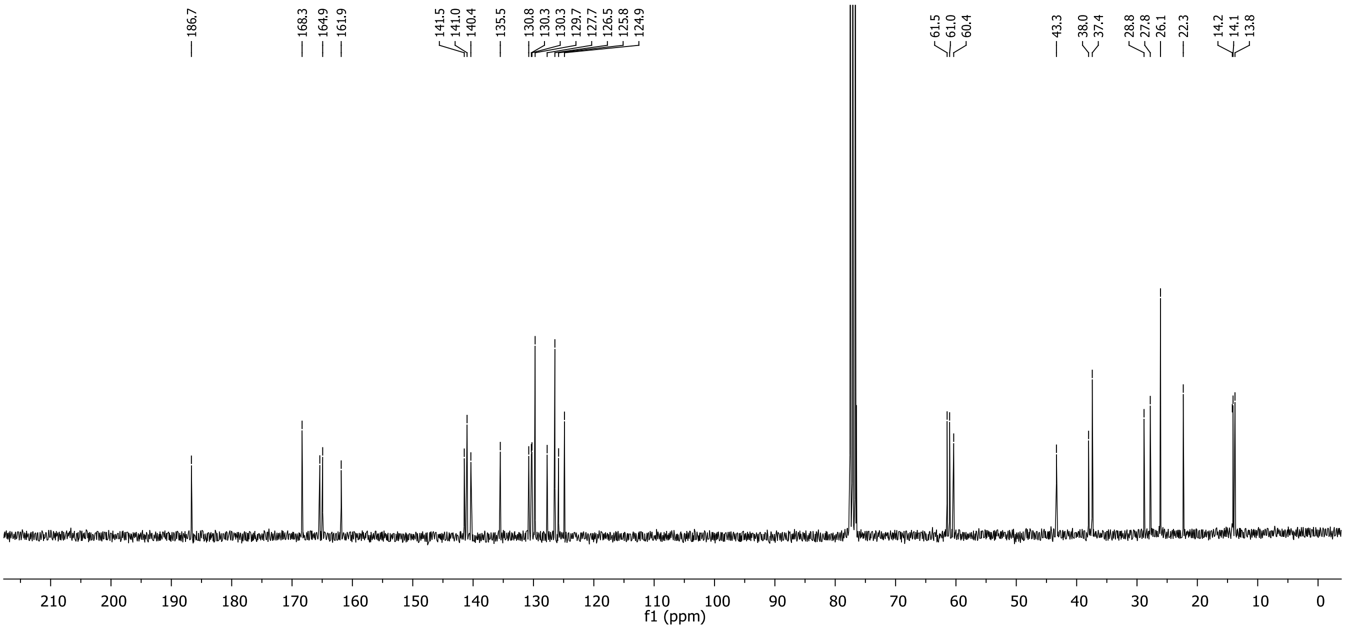

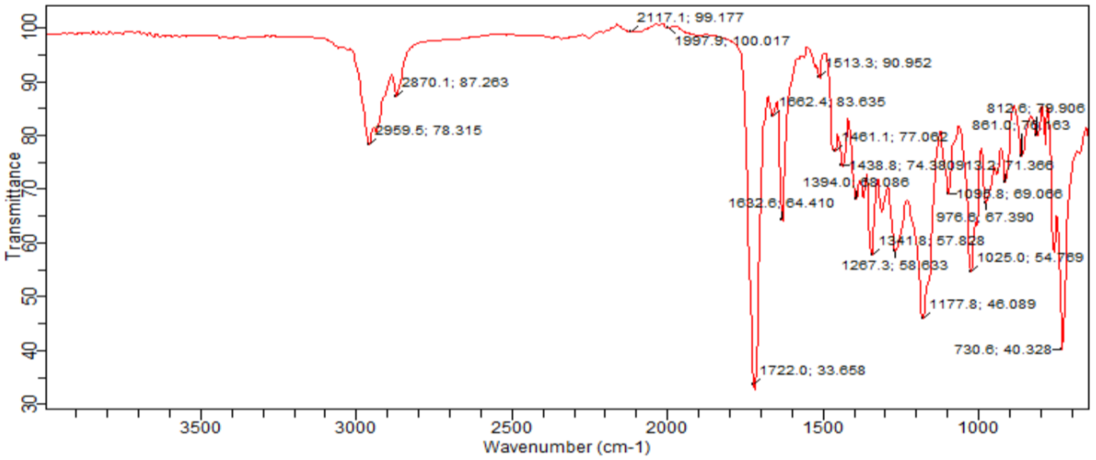

Diethyl (*E*)-4-((1S,3aS,3bR,5aS,10aS,10bS,12aS)-1-hydroxy-1,10a,12a-trimethyl-2,3,3a,3b,4,5,5a,6,10,10a,10b,11,12,12a-tetradecahydrocyclopenta[5,6]naphtho[1,2-f]indazol-8(1H)-yl)hex-2-enedioate (3bn (*N*1-isomer)) (^{1}H NMR: 400 MHz, ^{13}C NMR: 101 MHz, 2D NOESY NMR: 400 MHz, $CDCl_3$):

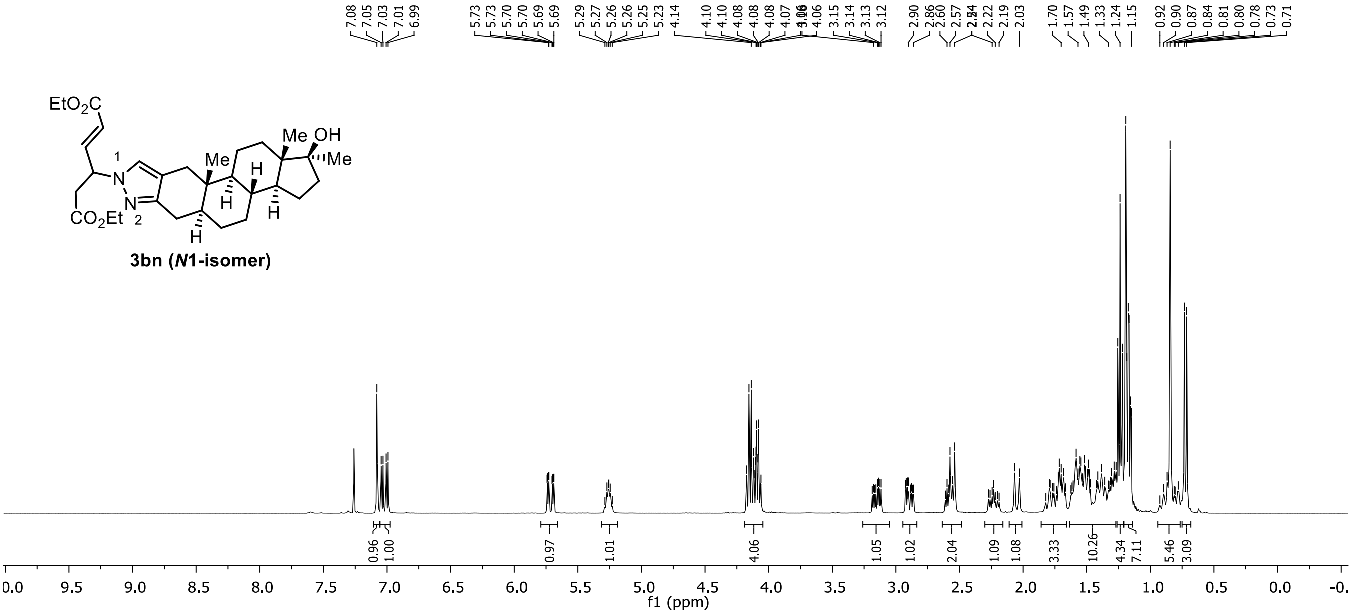

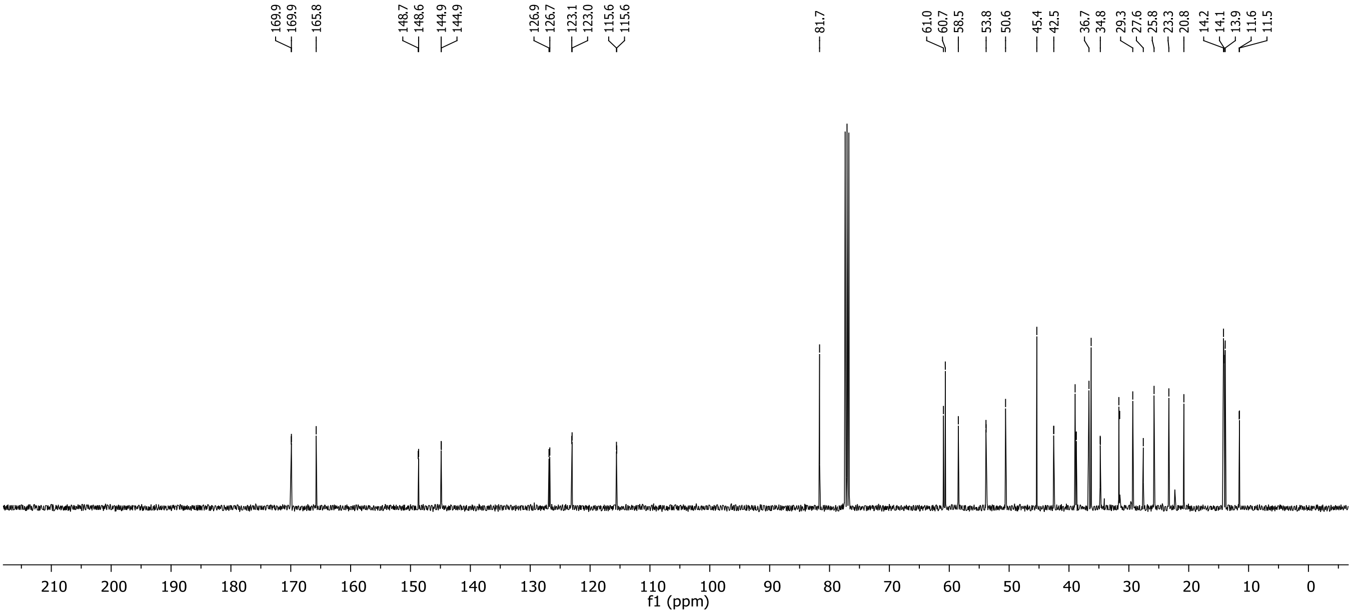

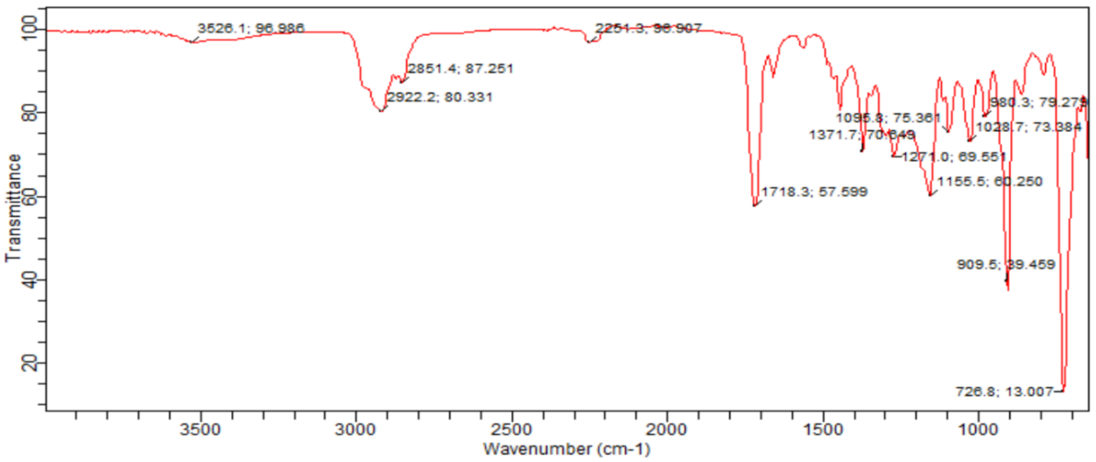

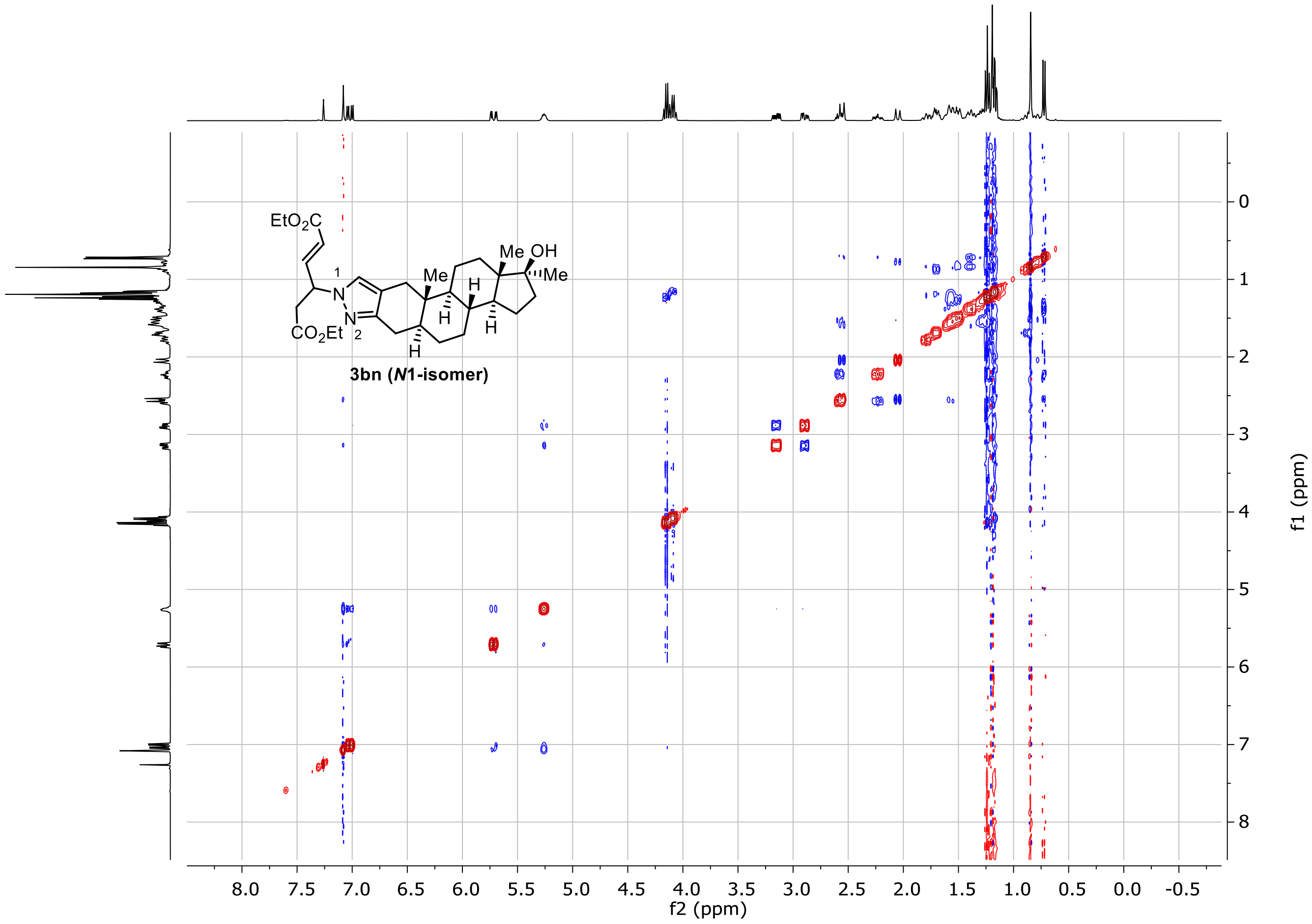
EtO2C
1
N
N
2
CO2Et
Me
H
H
H
Me OH
Me
3bn (N1-isomer)
8.0 7.5 7.0 6.5 6.0 5.5 5.0 4.5 4.0 3.5 3.0 2.5 2.0 1.5 1.0 0.5 0.0 -0.5
f2 (ppm)
f1 (ppm)
0 1 2 3 4 5 6 7 8

Diethyl (E)-4-((1S,3aS,3bR,5aS,10aS,10bS,12aS)-1-hydroxy-1,10a,12a-trimethyl-2,3,3a,3b,4,5,5a,6,10,10a,10b,11,12,12a-tetradecahydrocyclopenta[5,6]naphtho[1,2-f]indazol-7(1H)-yl)hex-2-enedioate (3bn (*N*2-isomer)) (^{1}H NMR: 400 MHz, ^{13}C NMR: 101 MHz, 2D NOESY NMR: 400 MHz, $CDCl_3$):

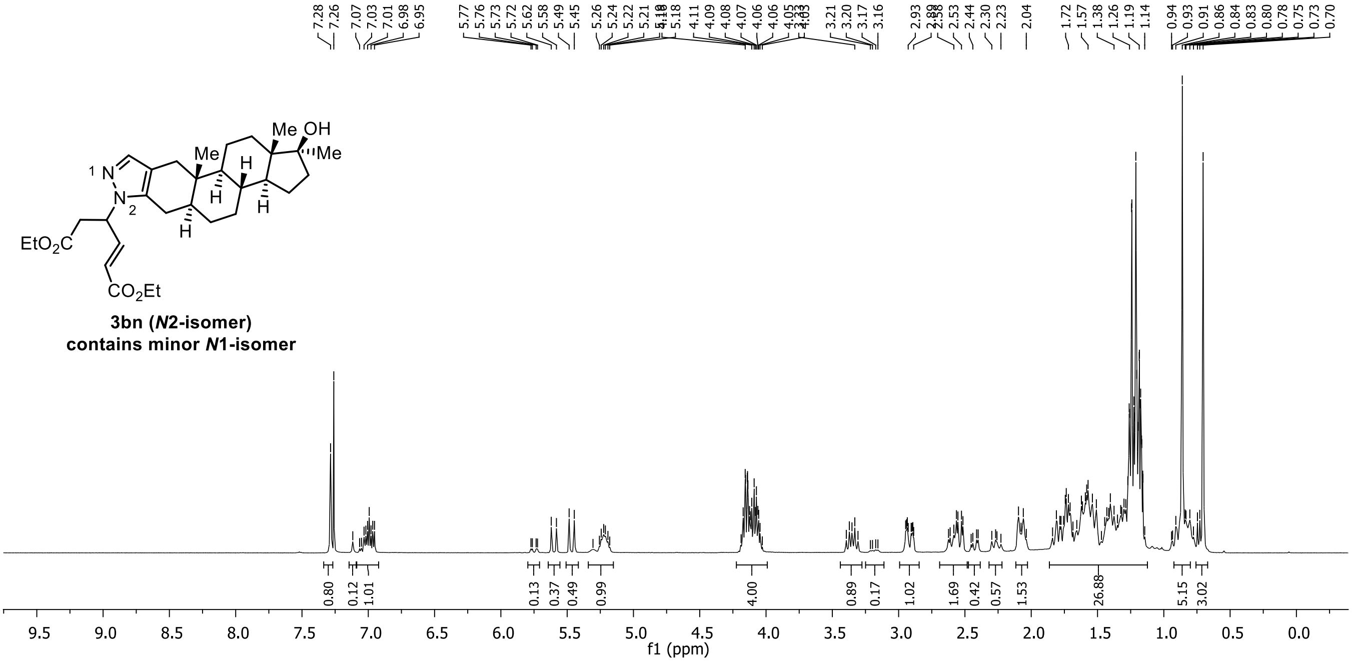

3bn (*N*2-isomer)
contains minor *N*1-isomer

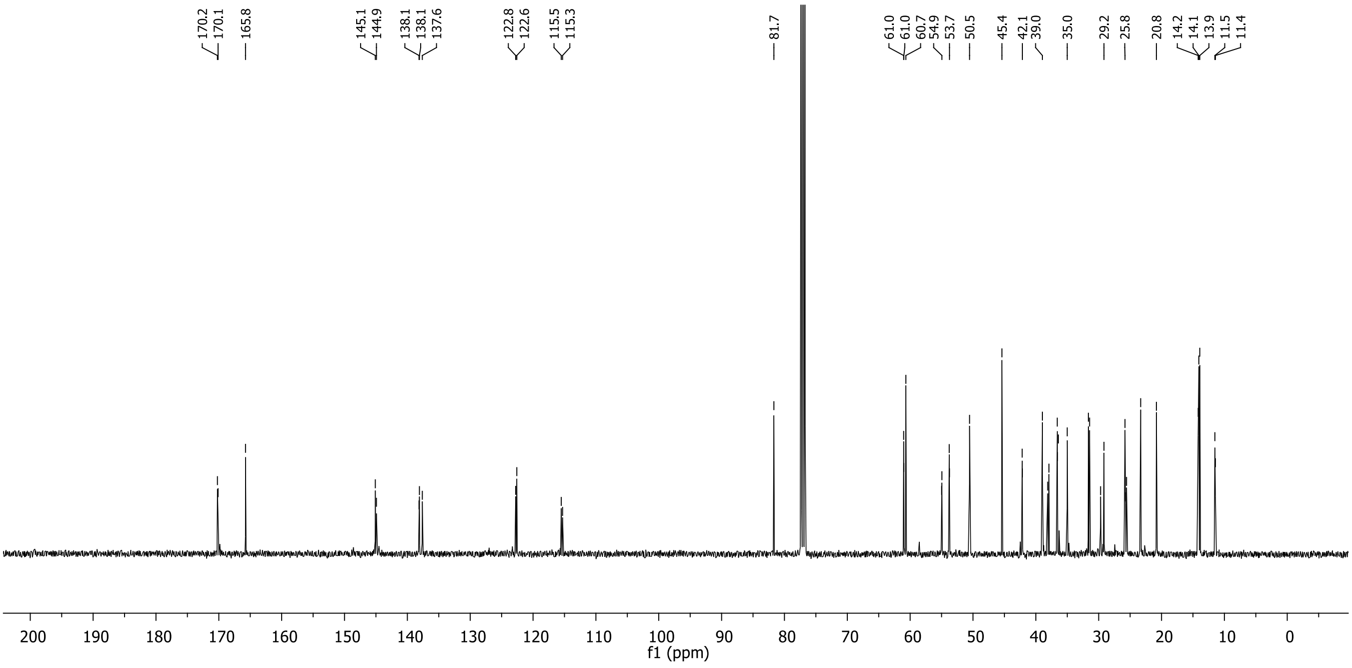

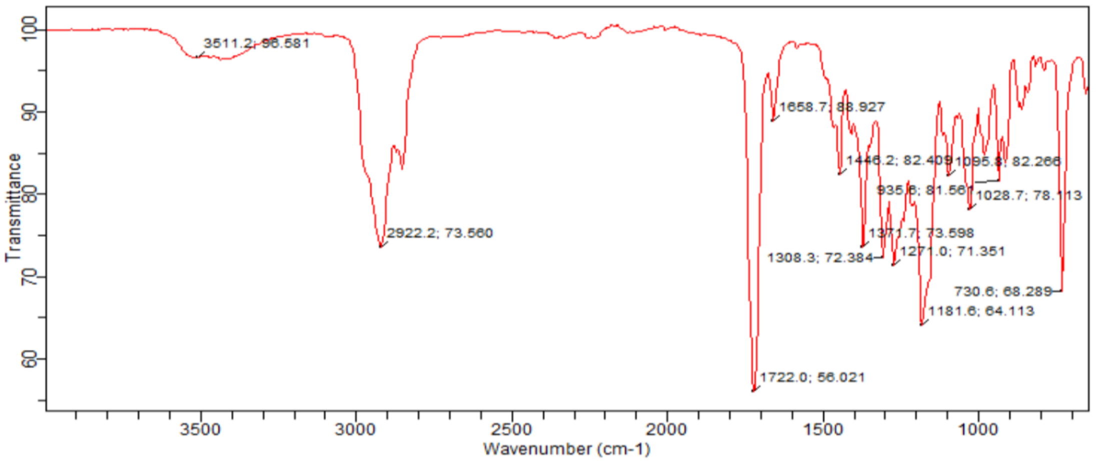

Transmittance
Wavenumber (cm-1)
3511.2; 96.581
2922.2; 73.560
1658.7; 88.927
1722.0; 56.021
1446.2; 82.409
1308.3; 72.384
1371.7; 73.598
1271.0; 71.351
1181.6; 64.113
935.8; 81.561
1095.8; 82.266
1028.7; 78.113
730.6; 68.289

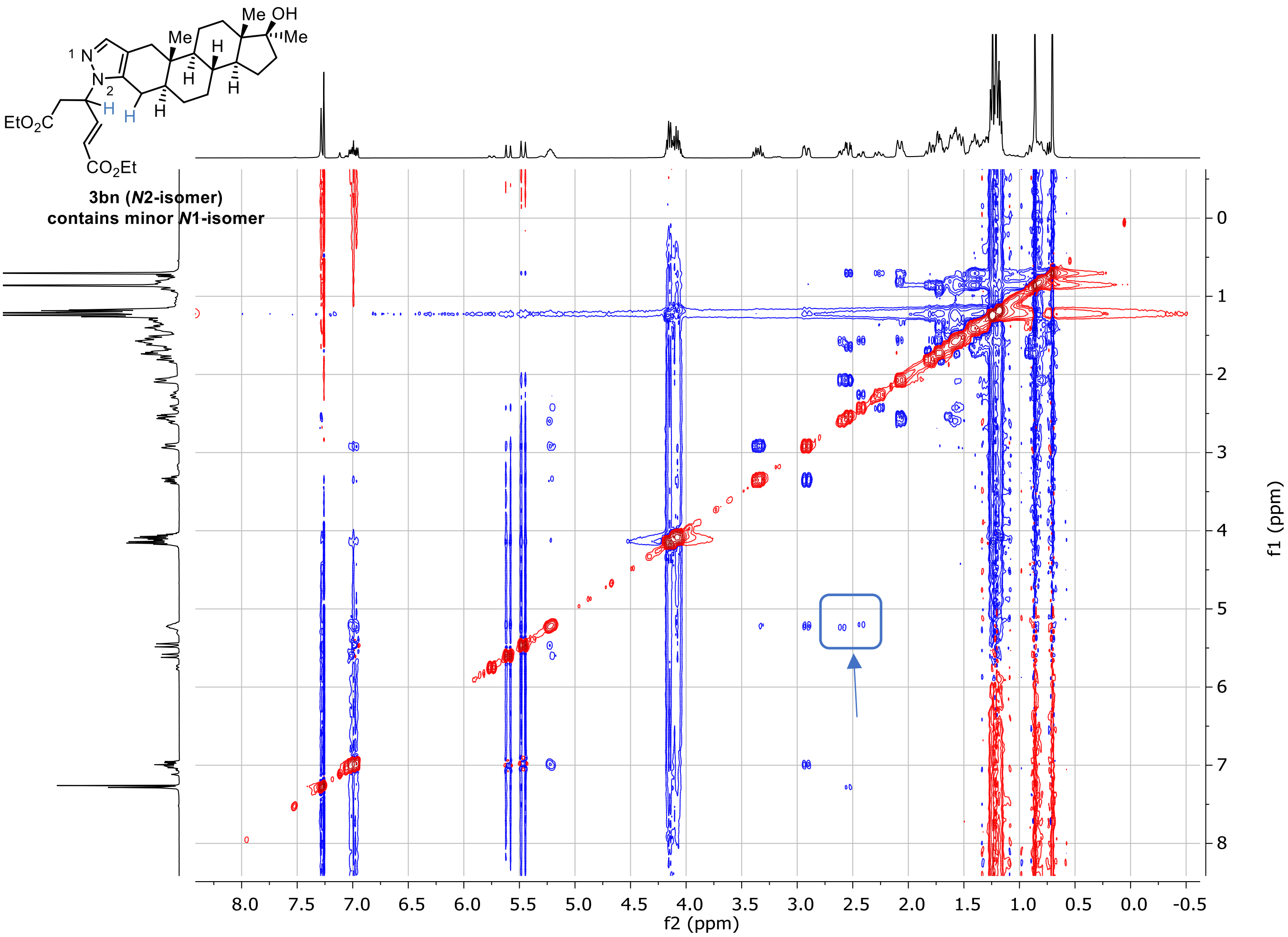

EtO2C
CO2Et
Me
OH
3bn (N2-isomer)
contains minor N1-isomer
f2 (ppm)
f1 (ppm)

Ethyl 4-(4-chloro-1H-pyrazol-1-yl)hex-3-enoate (3a'') (^{1}H NMR: 300 MHz, ^{13}C NMR: 75 MHz, 2D NOESY NMR: 400 MHz, $CDCl_3$):

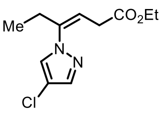

3a''

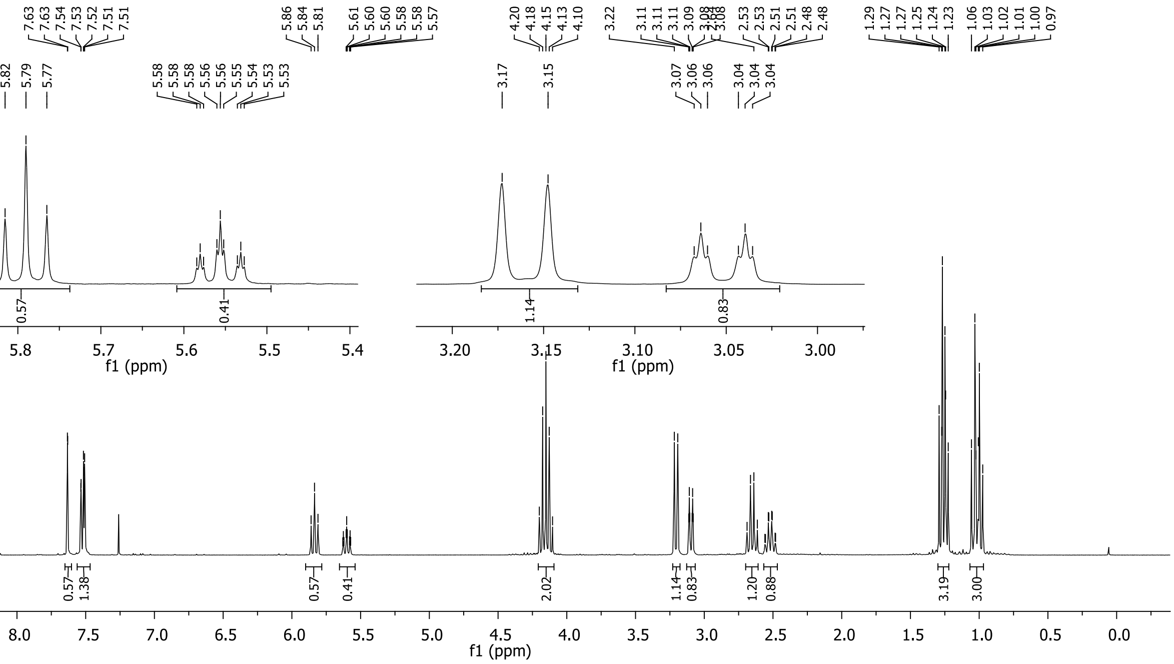

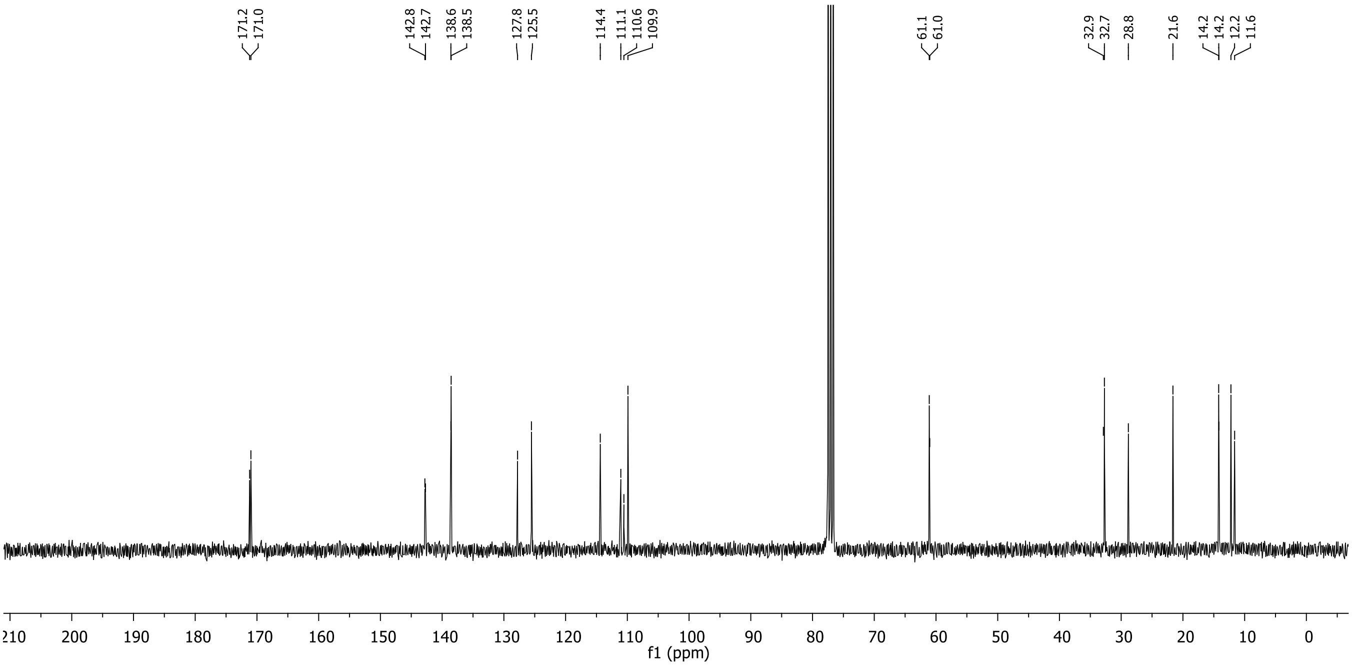

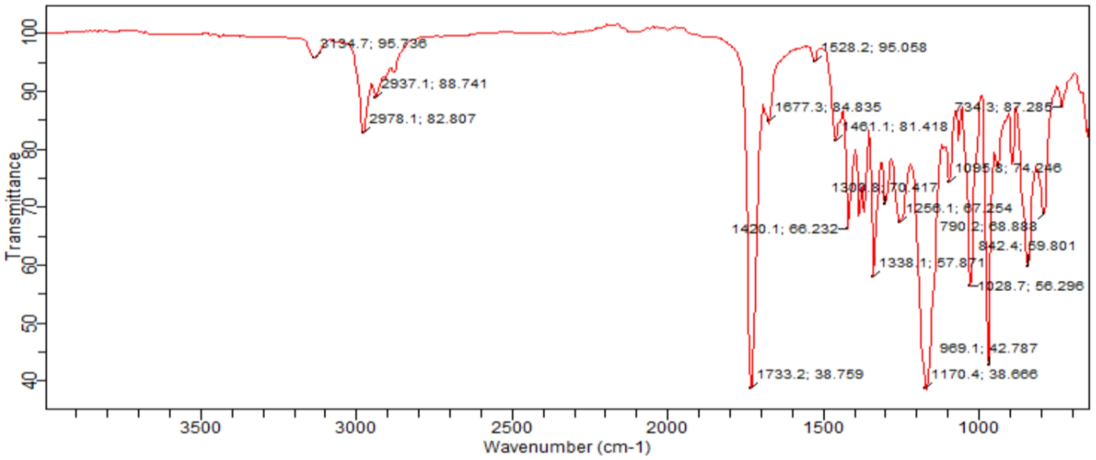
3134.7; 95.736
2937.1; 88.741
2978.1; 82.807
1528.2; 95.058
1677.3; 84.835
1461.1; 81.418
734.3; 87.285
1095.8; 74.246
1309.8; 70.417
1256.1; 67.254
790.2; 68.888
1420.1; 66.232
842.4; 59.801
1338.1; 57.871
1028.7; 56.296
969.1; 42.787
1733.2; 38.759
1170.4; 38.666
Transmittance
Wavenumber (cm-1)

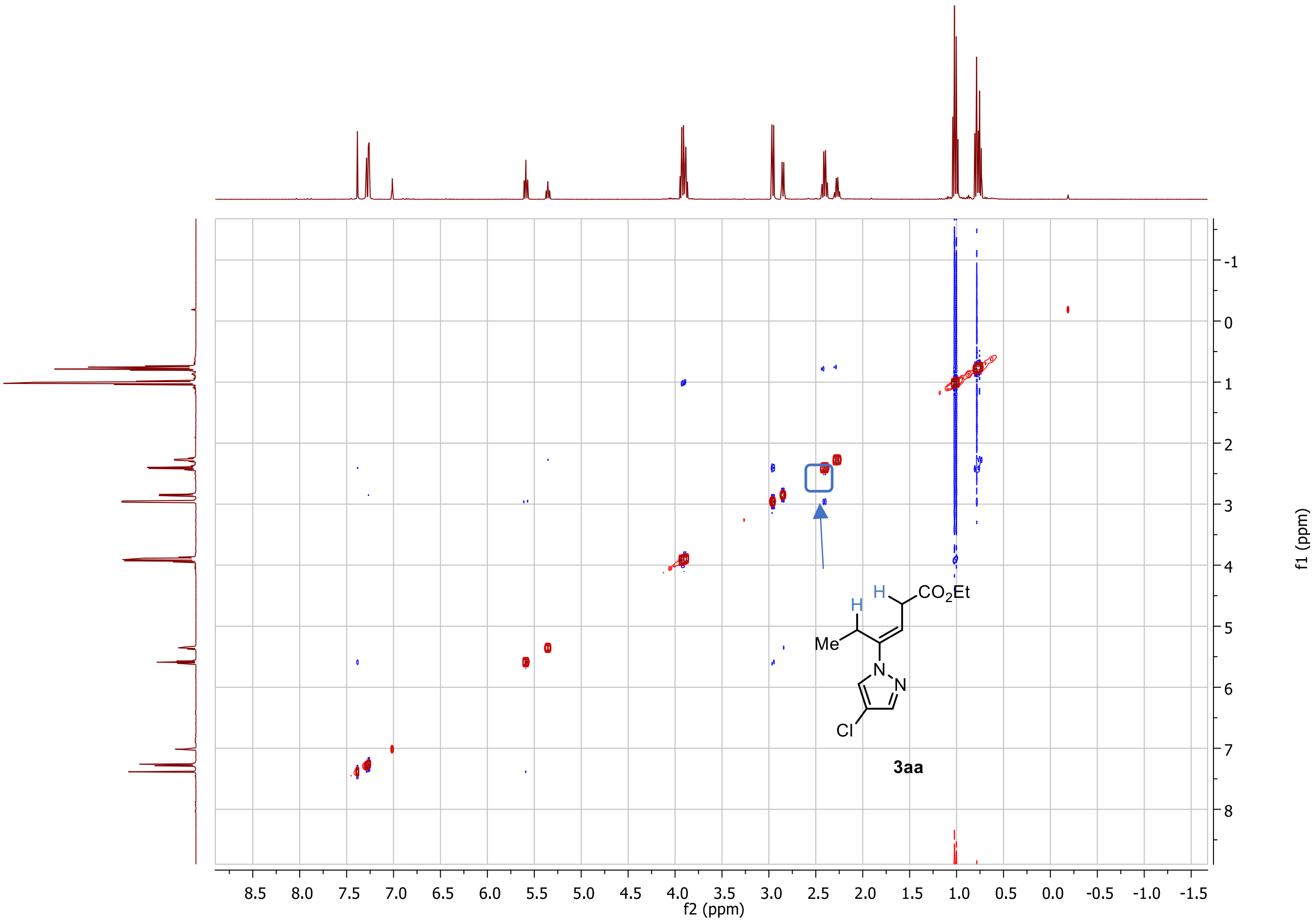
f1 (ppm)
f2 (ppm)
CO2Et
Me
Cl
3aa

Pent-3-yn-1-yl 4-(4-chloro-1H-pyrazol-1-yl)hex-3-enoate (3w'') (^{1}H NMR: 300 MHz, ^{13}C NMR: 75 MHz, CDCl$_3$):

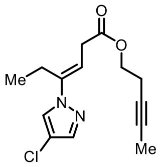

3w''

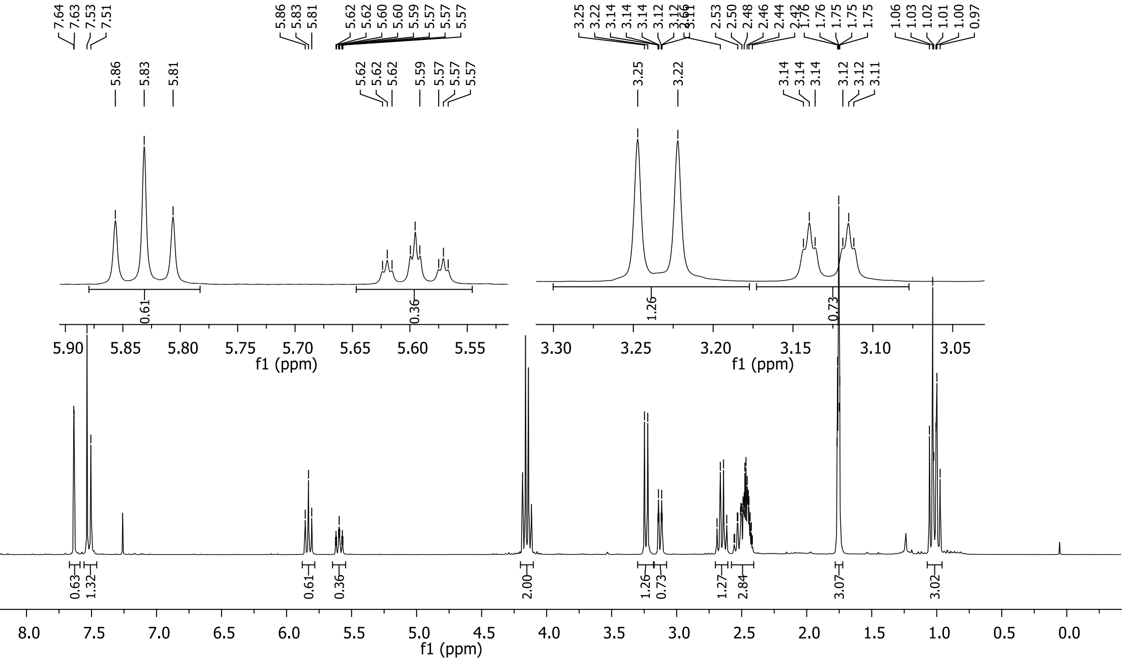

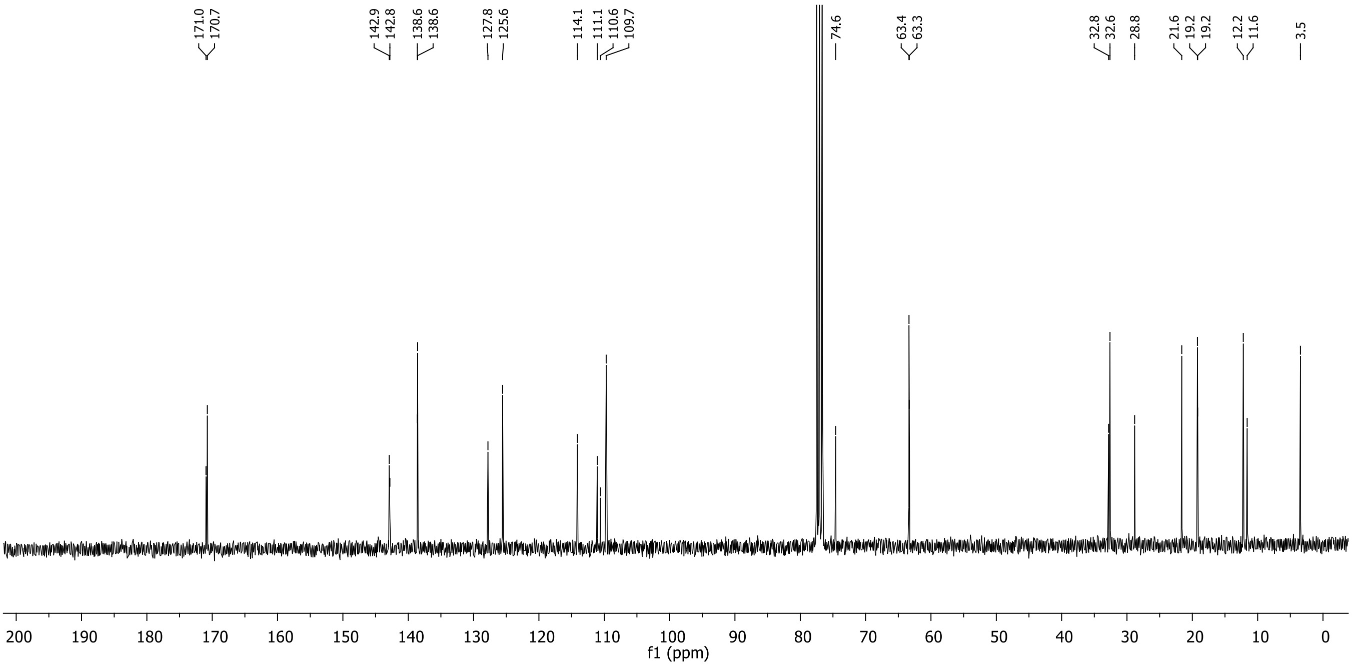

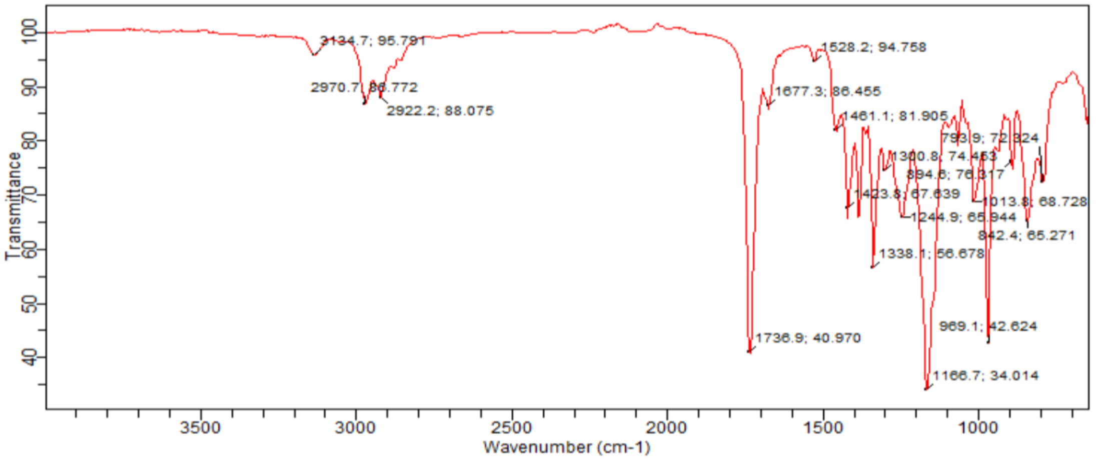

Ethyl 4-(1H-benzo[d][1,2,3]triazol-1-yl)hex-3-enoate (3av'') (^{1}H NMR: 300 MHz, ^{13}C NMR: 75 MHz, $CDCl_3$):

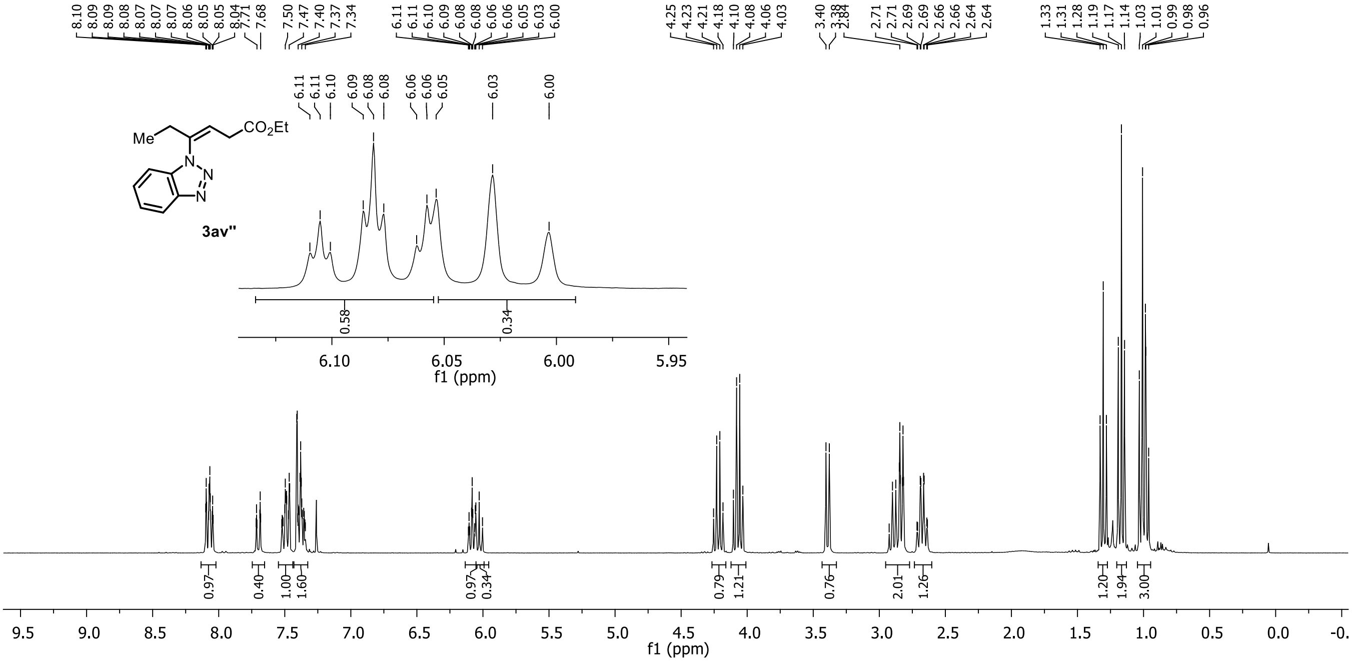

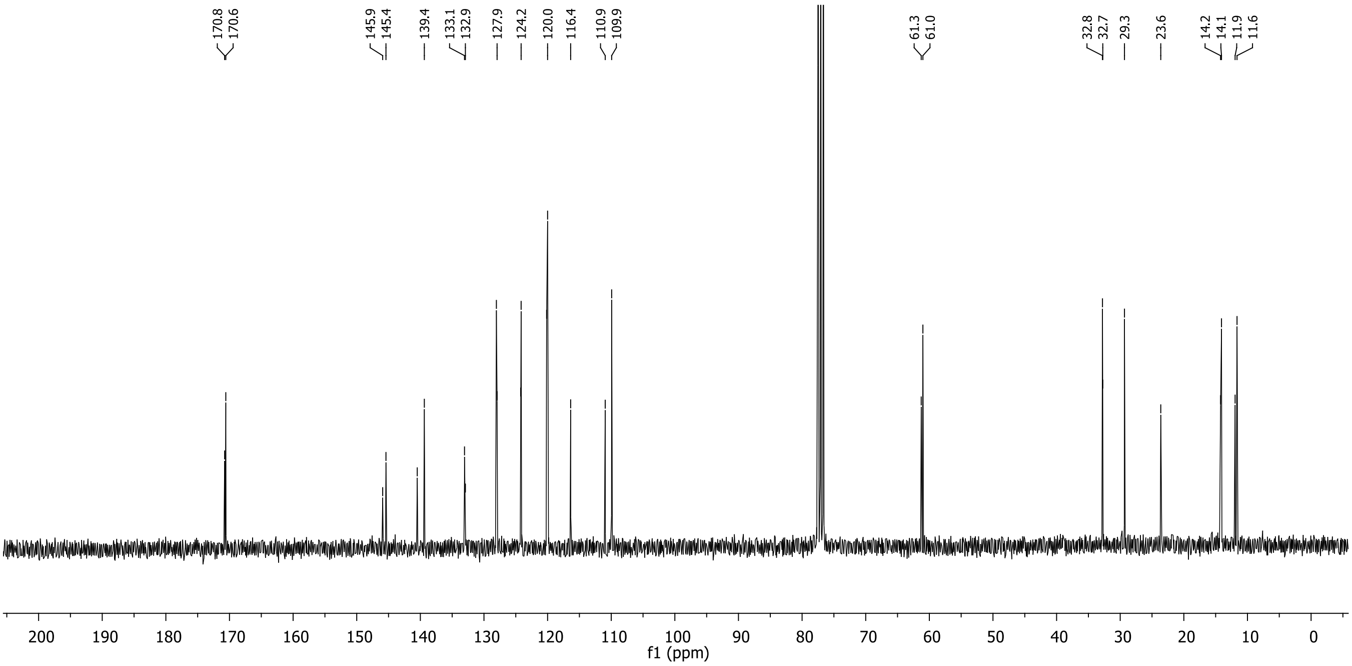

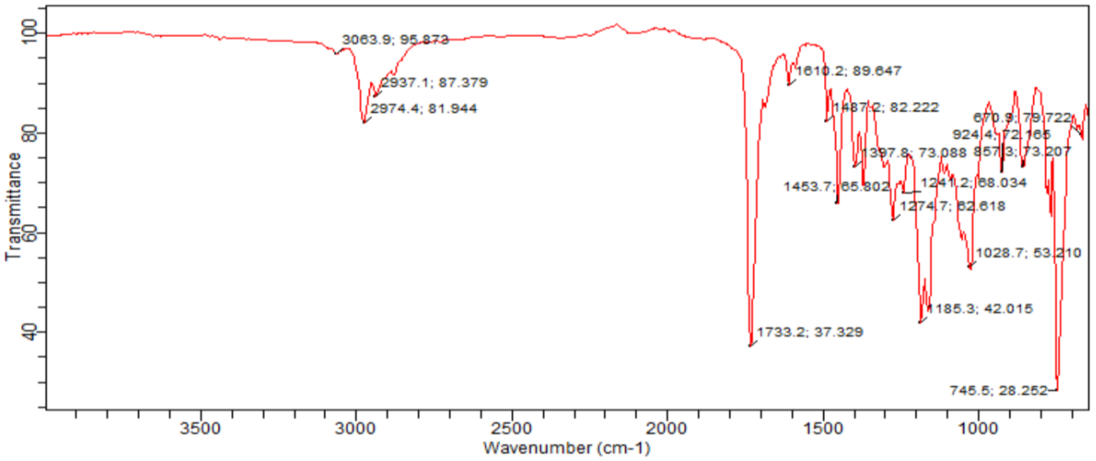

4-Chloro-1-(4-(phenylsulfonyl)but-2-en-2-yl)-1H-pyrazole (3ae'') (^{1}H NMR: 300 MHz, ^{13}C NMR: 75 MHz, $CDCl_3$):

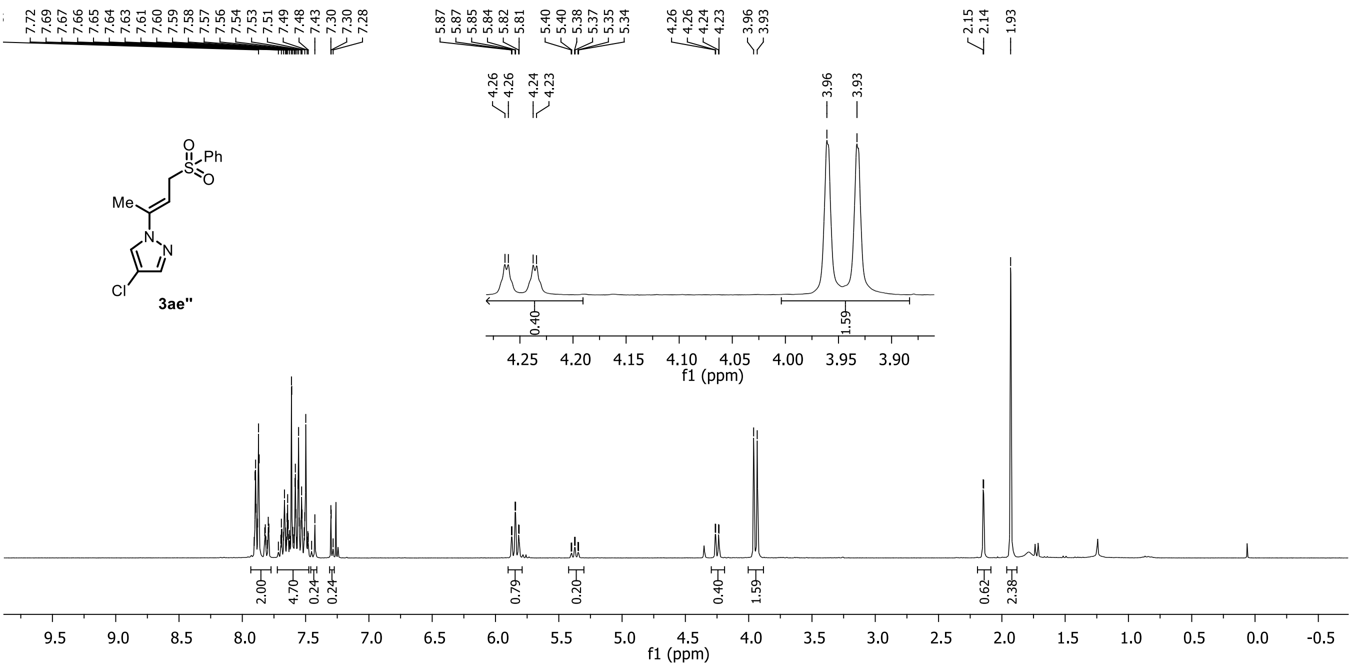

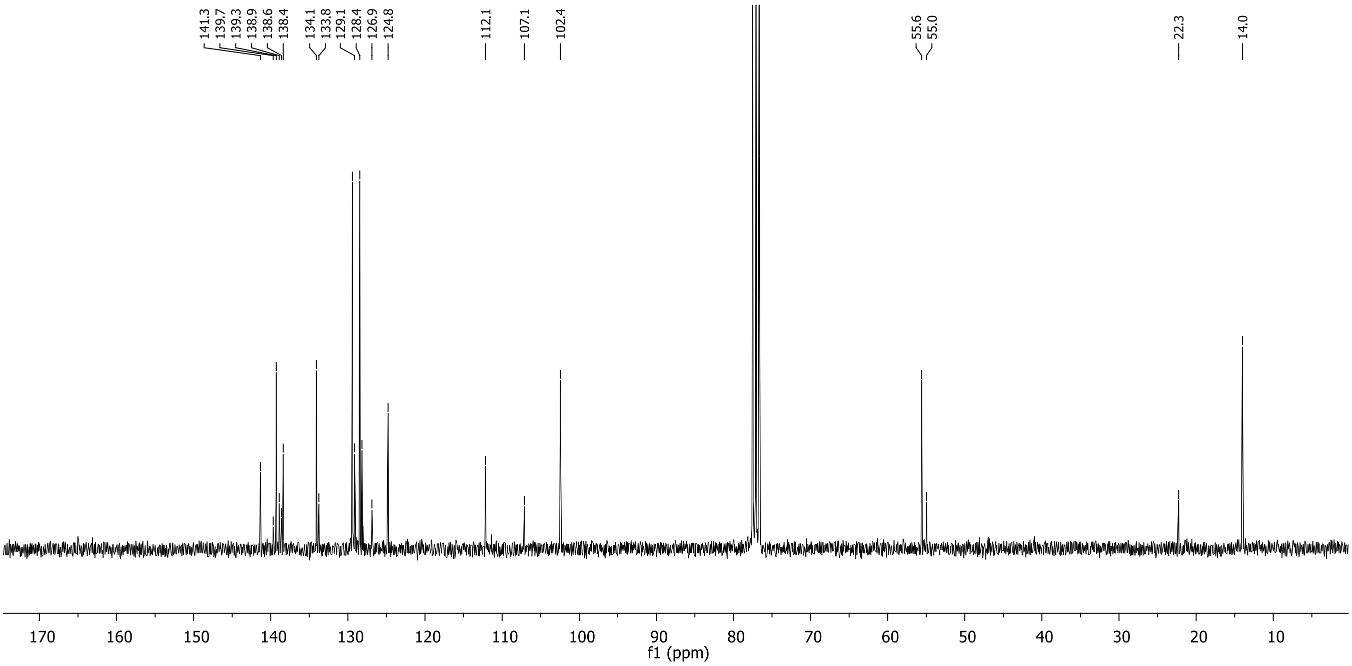

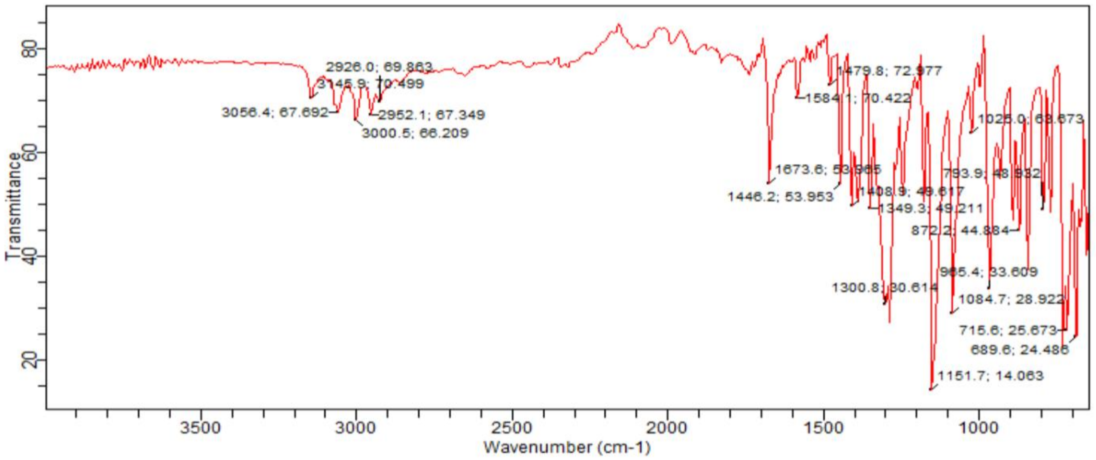

(*E*)-4-(4-Chloro-1H-pyrazol-1-yl)pent-3-enenitrile (3ag’’) (^{1}H NMR: 300 MHz, ^{13}C NMR: 75 MHz, $CDCl_3$):

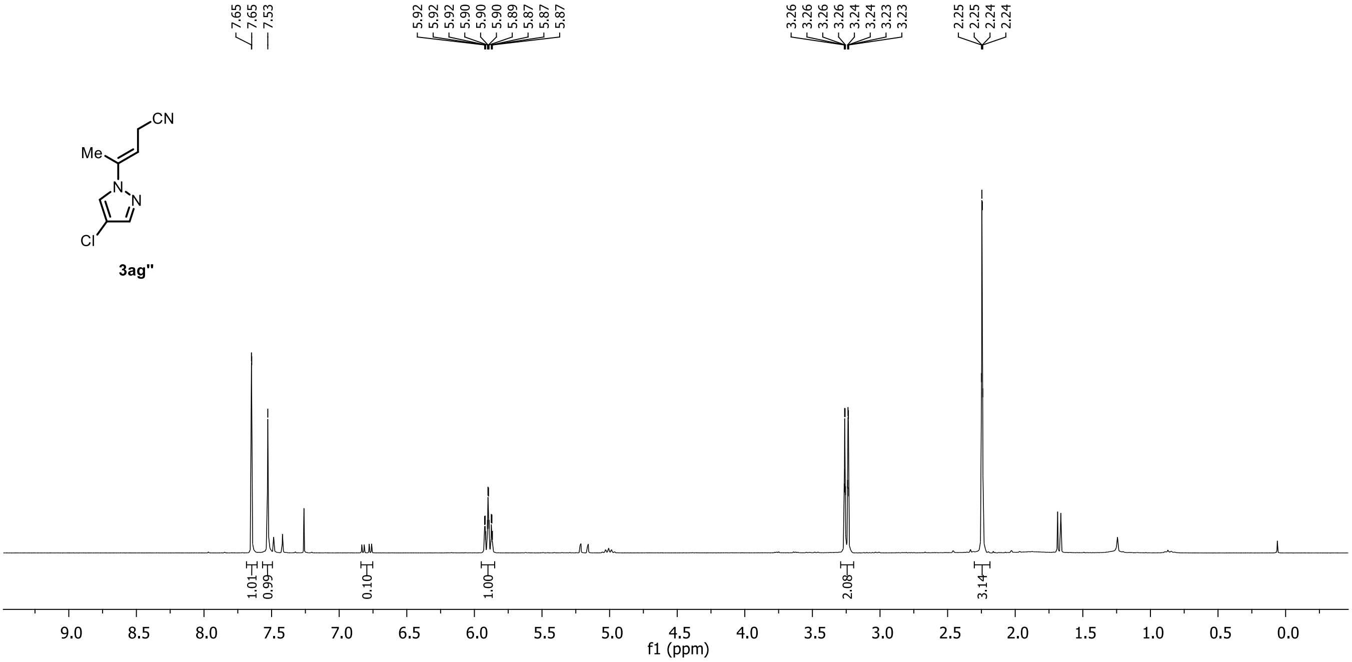

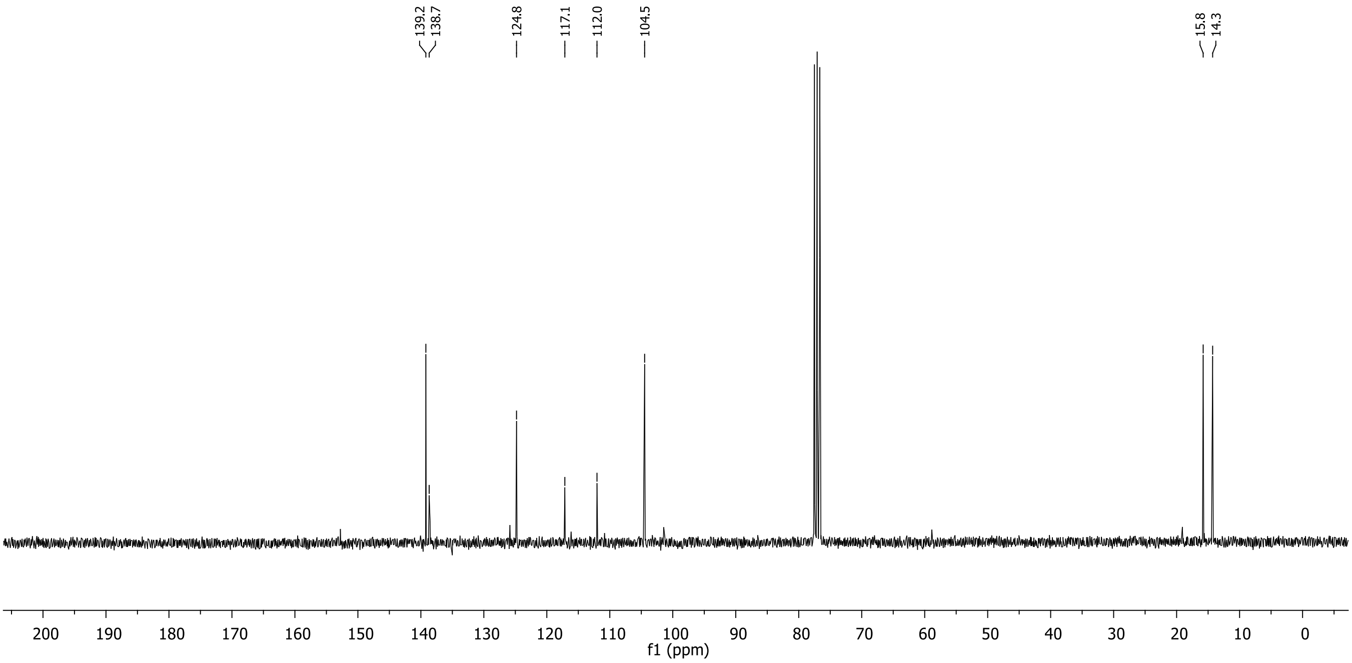

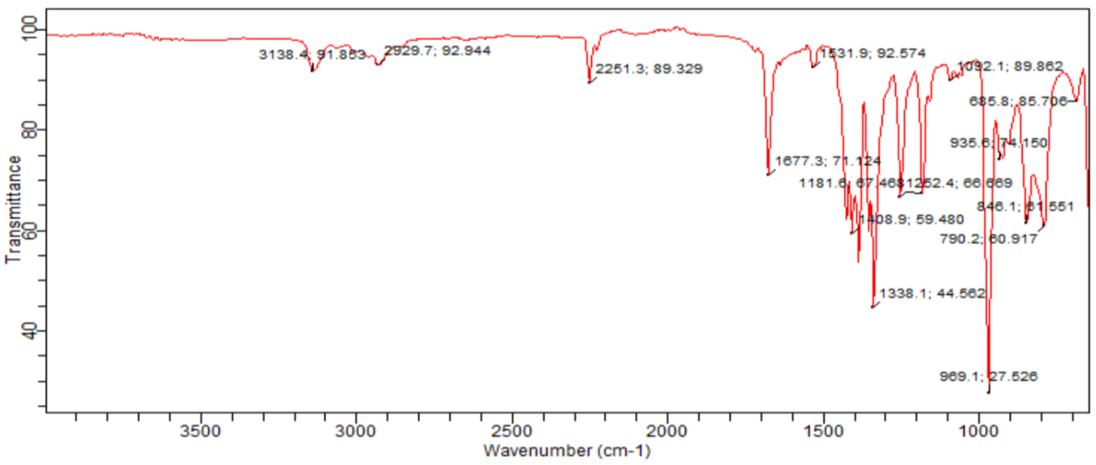

(3-(4-Chloro-1H-pyrazol-1-yl)allyl)diphenylphosphine oxide (3aj'') (^{1}H NMR: 300 MHz, ^{13}C NMR: 75 MHz, ^{31}P NMR: 162 MHz, $CDCl_3$)

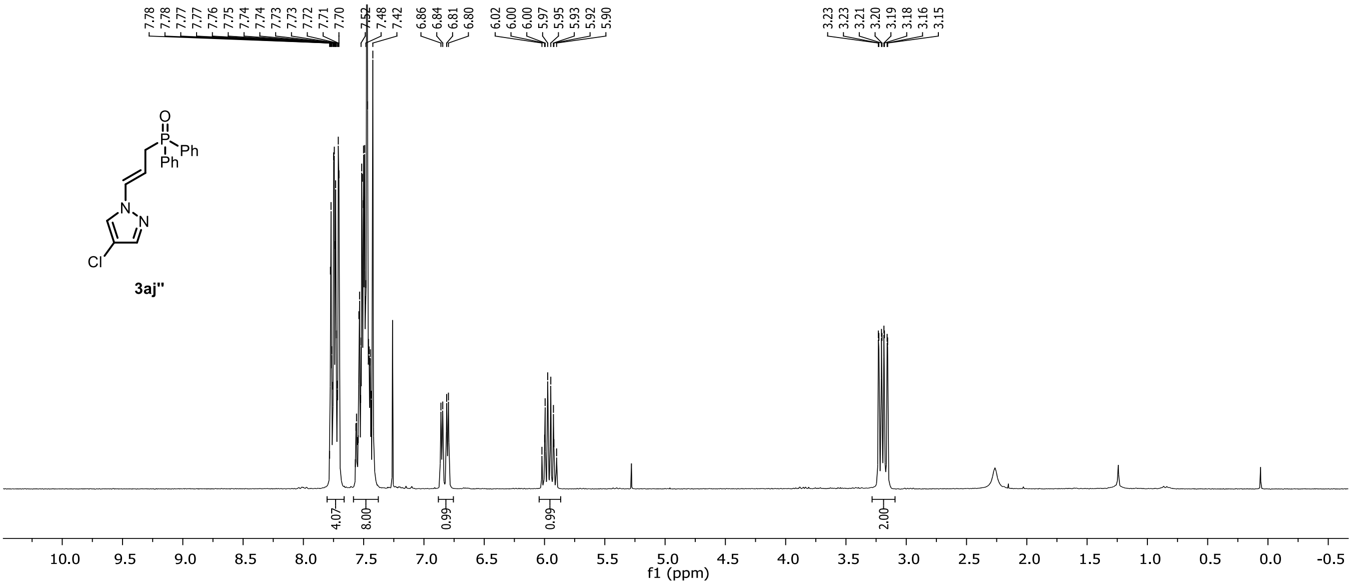

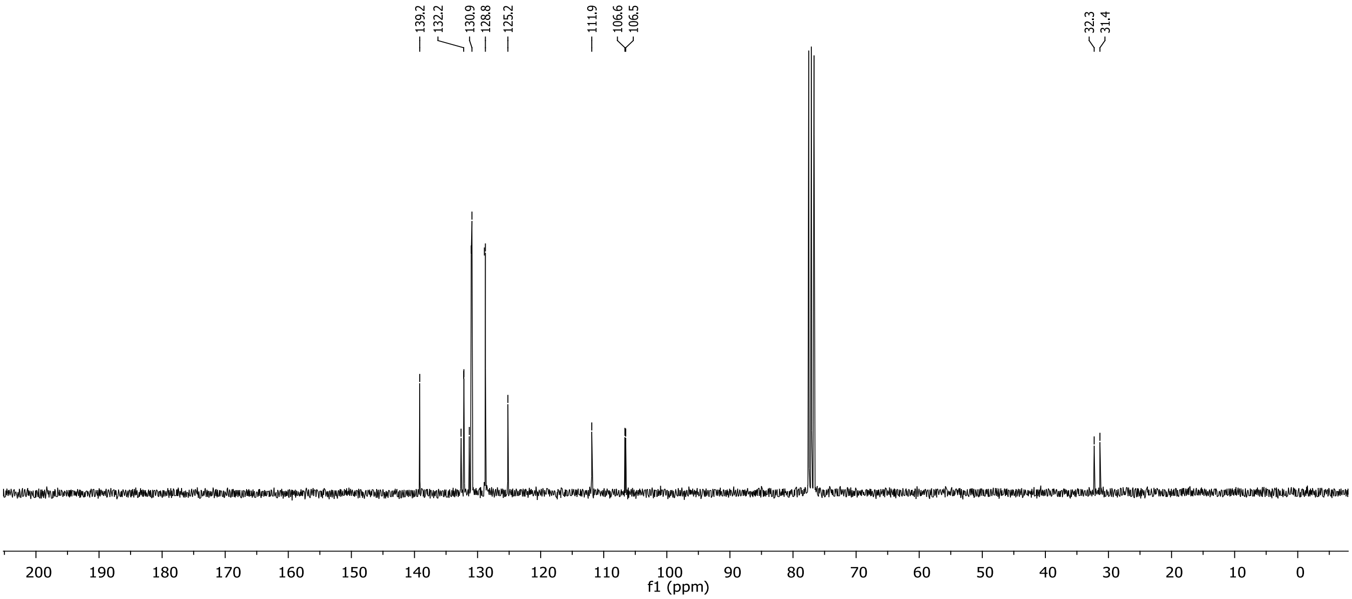

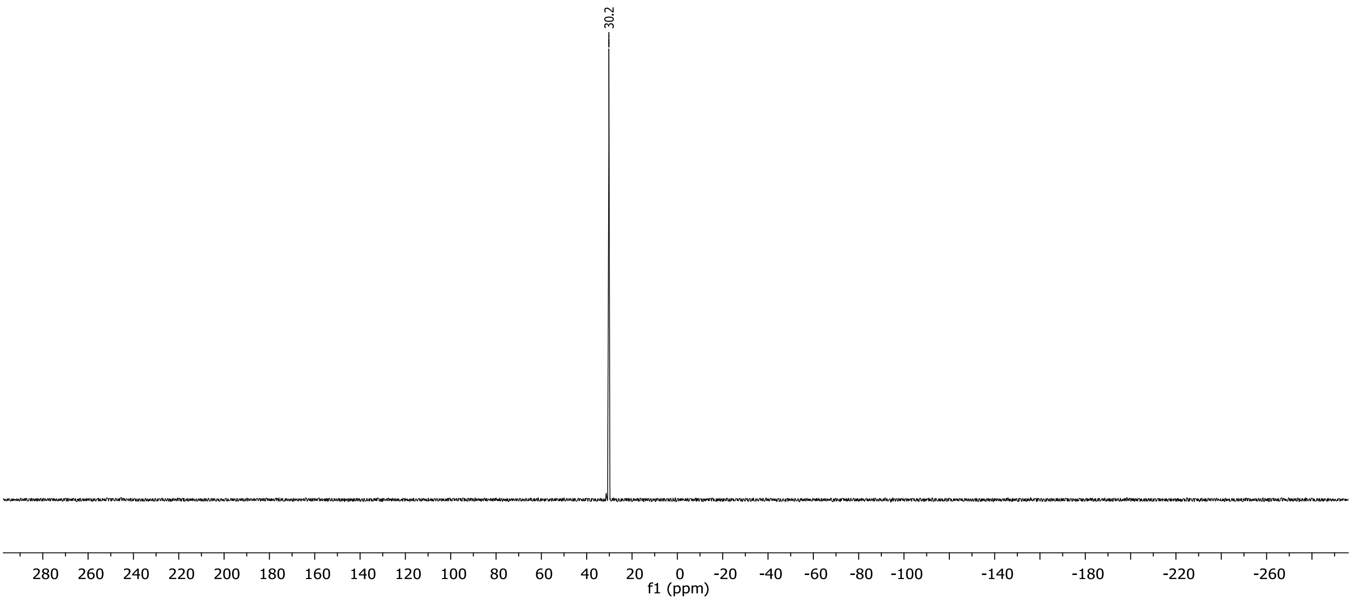

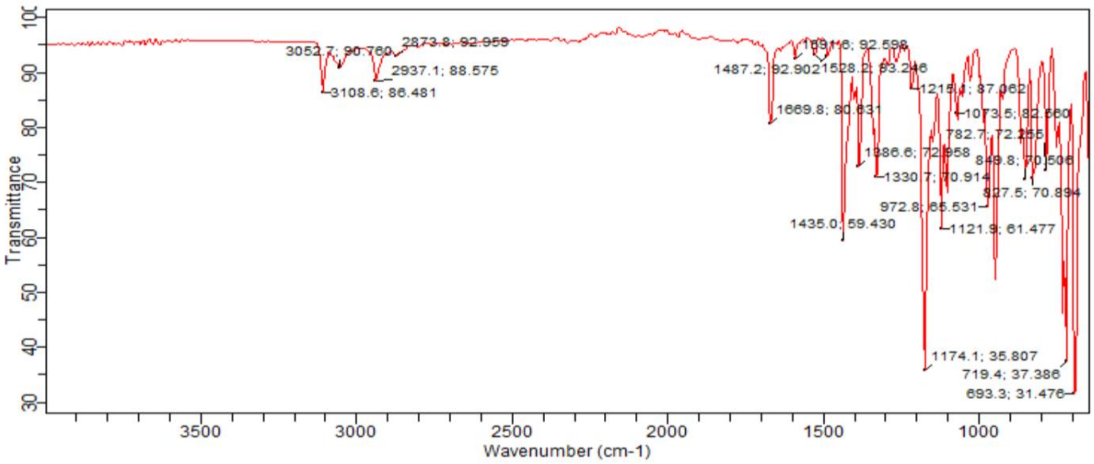
Transmittance
Wavenumber (cm-1)
3108.6; 86.481
3052.7; 90.760
2937.1; 88.575
2873.8; 92.959
1669.8; 80.631
1487.2; 92.902
1528.2; 93.246
1435.0; 59.430
1386.6; 72.958
1330.7; 70.914
1215.1; 87.062
1174.1; 35.807
1121.9; 61.477
1073.5; 82.660
972.8; 65.531
927.5; 70.894
849.8; 70.506
782.7; 72.255
719.4; 37.386
693.3; 31.476

Diethyl (*E*)-3-(4-chloro-1H-pyrazol-1-yl)hex-2-enedioate (C_{12}) (3c'') (^{1}H NMR: 400 MHz, ^{13}C NMR: 101 MHz, 2D NOESY NMR: 400 MHz, $CDCl_3$):

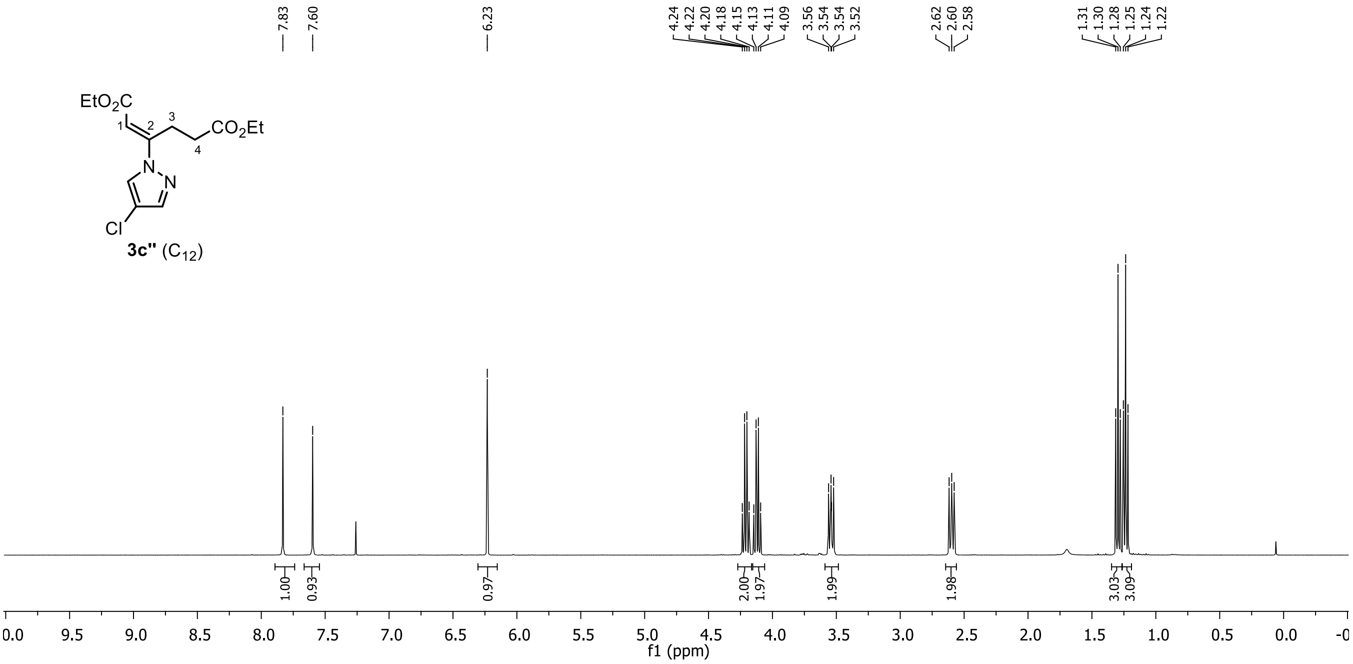

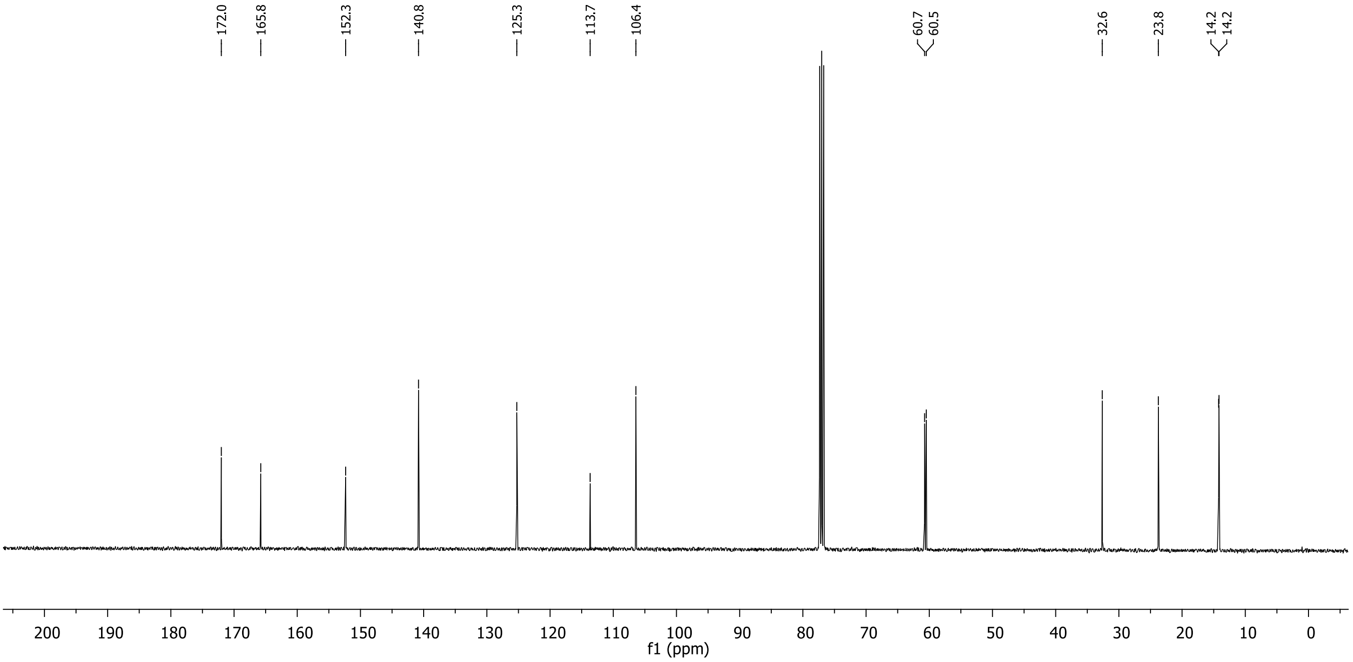

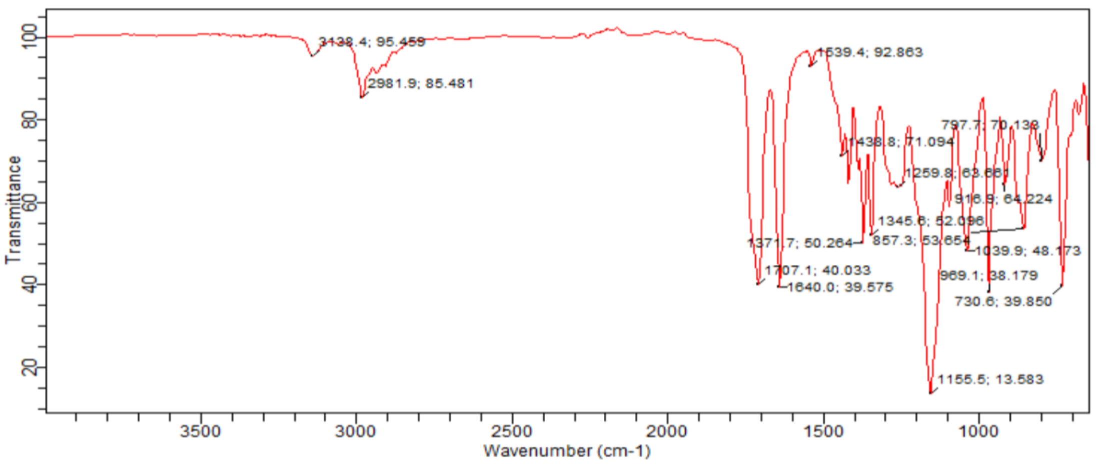

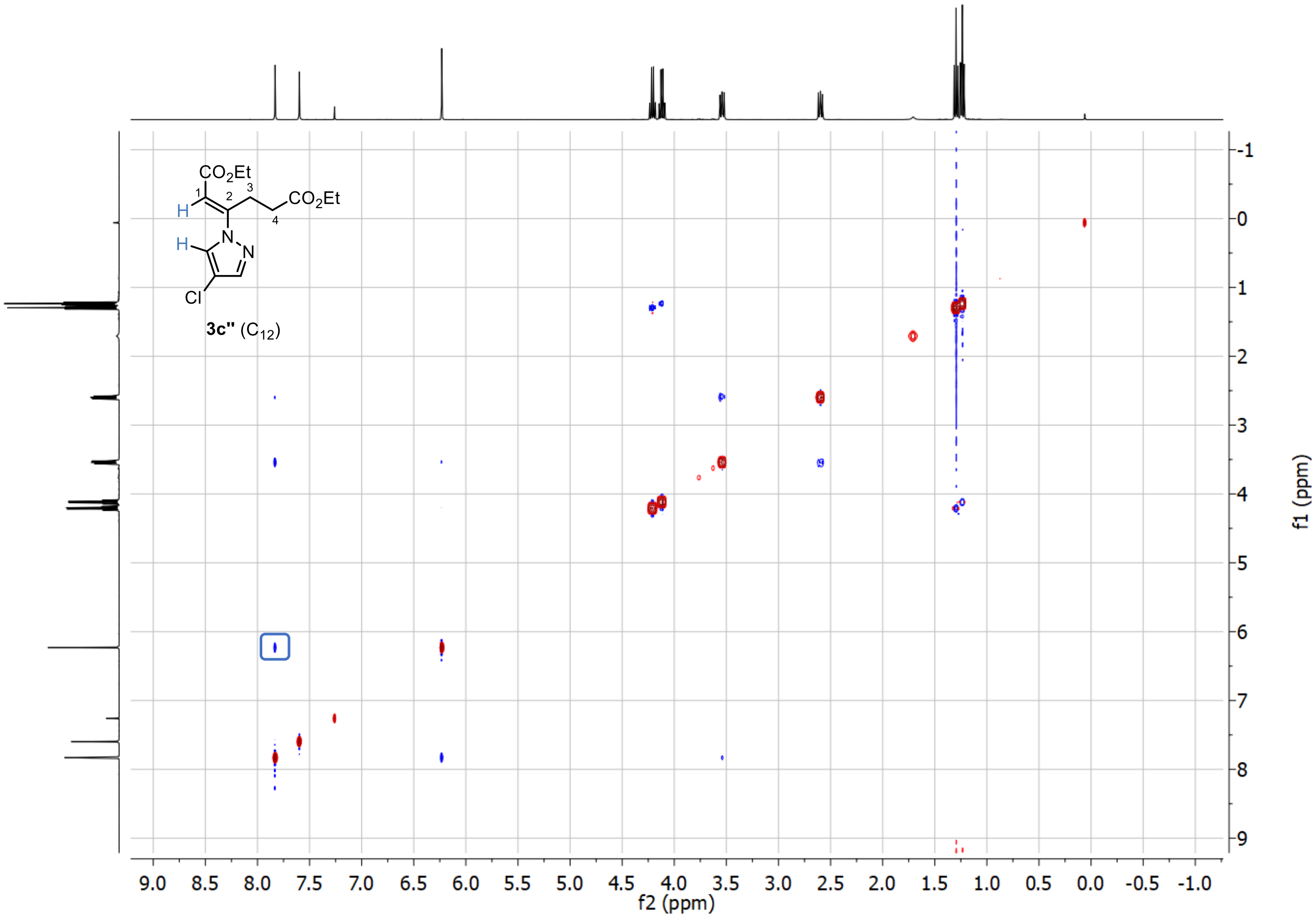
CO2Et
CO2Et
H
H
N
N
Cl
1
2
3
4
3c'' (C12)
f2 (ppm)
f1 (ppm)

Diethyl (*E*)-3-(4-chloro-1H-pyrazol-1-yl)hex-3-enedioate (C_{23}) (3c'') (^{1}H NMR: 400 MHz, ^{13}C NMR: 101 MHz, 2D NOESY NMR: 400 MHz, $CDCl_3$):

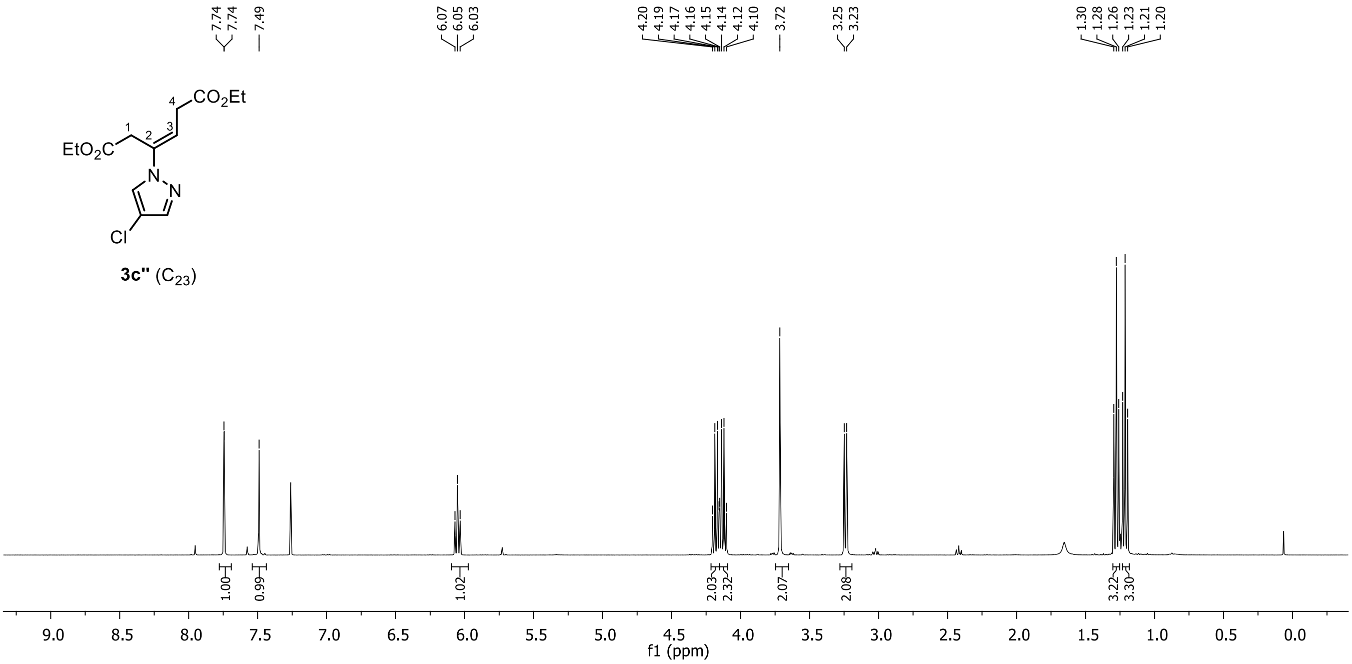

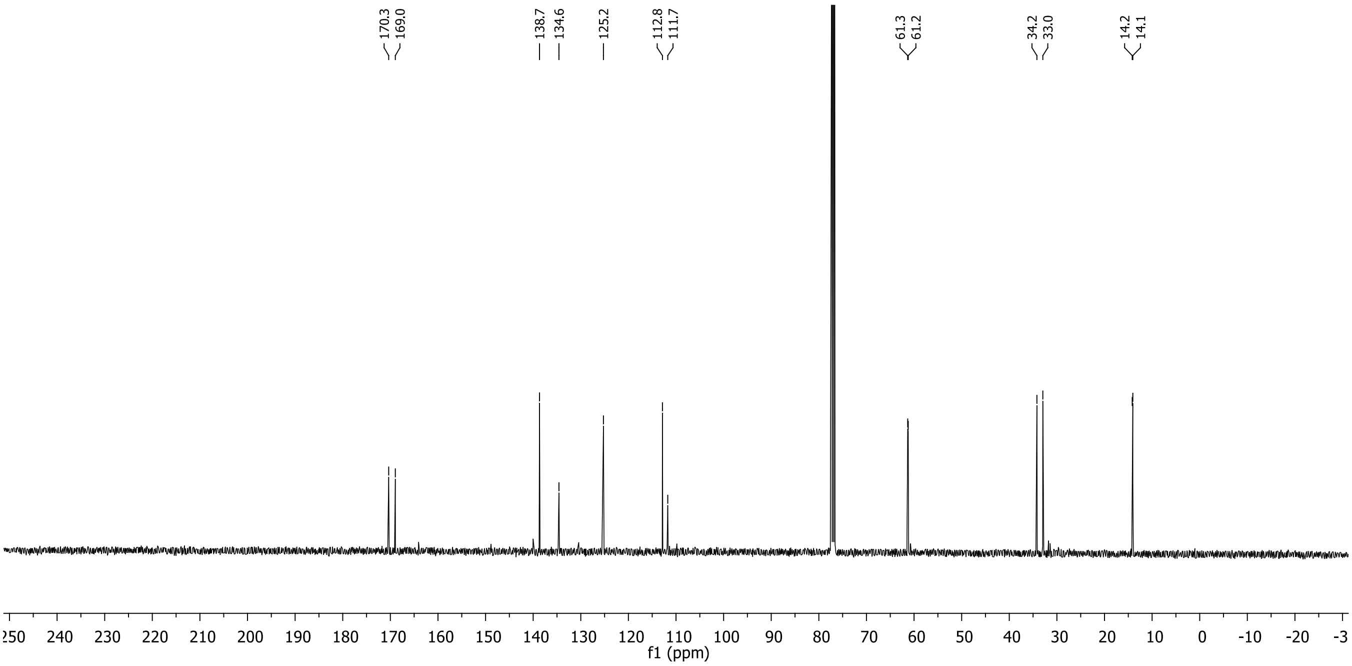

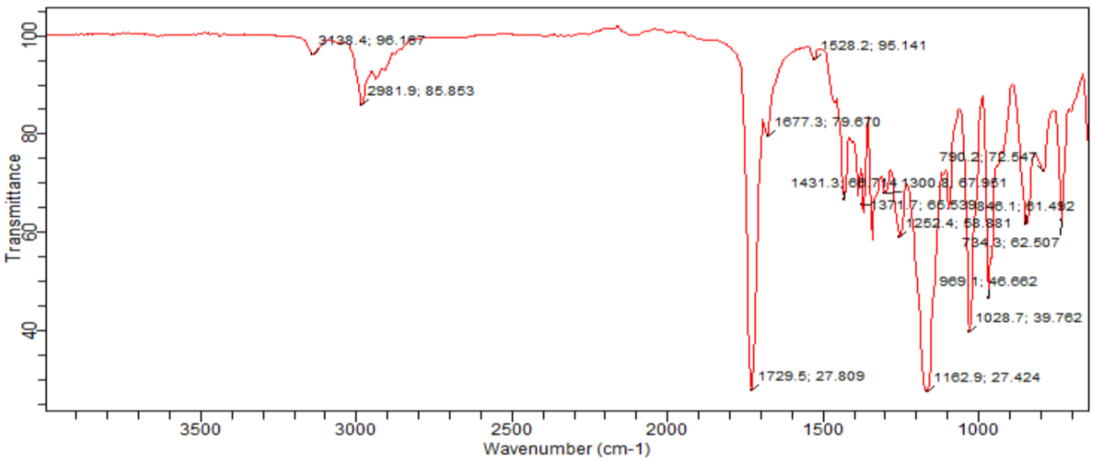

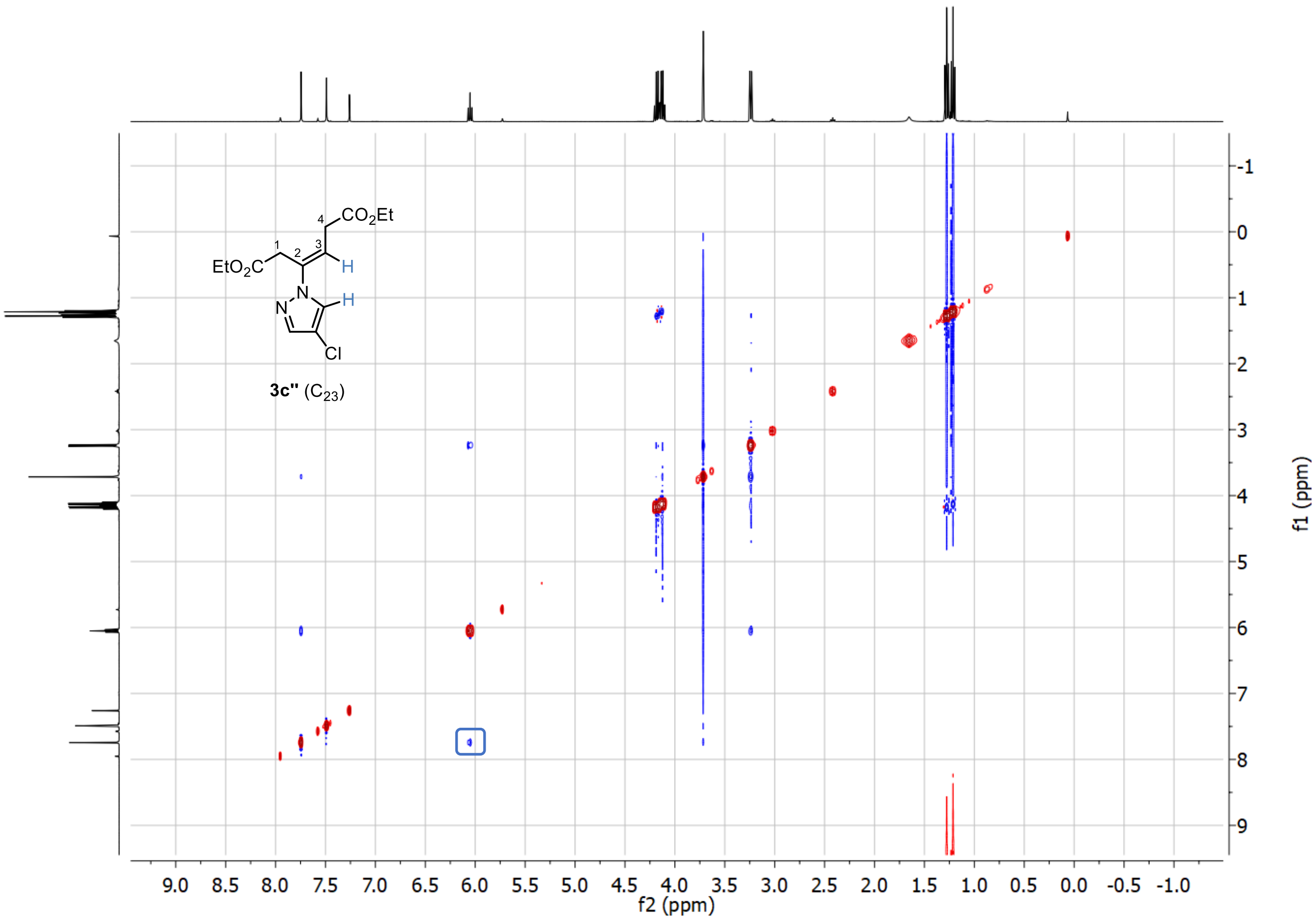

CO2Et
EtO2C
3c'' (C23)
f2 (ppm)
f1 (ppm)

(*E*)-4-Chloro-1-(3-(phenylsulfonyl)prop-1-en-1-yl)-1H-pyrazole (3af'') (^{1}H NMR: 300 MHz, ^{13}C NMR: 75 MHz, $CDCl_3$):

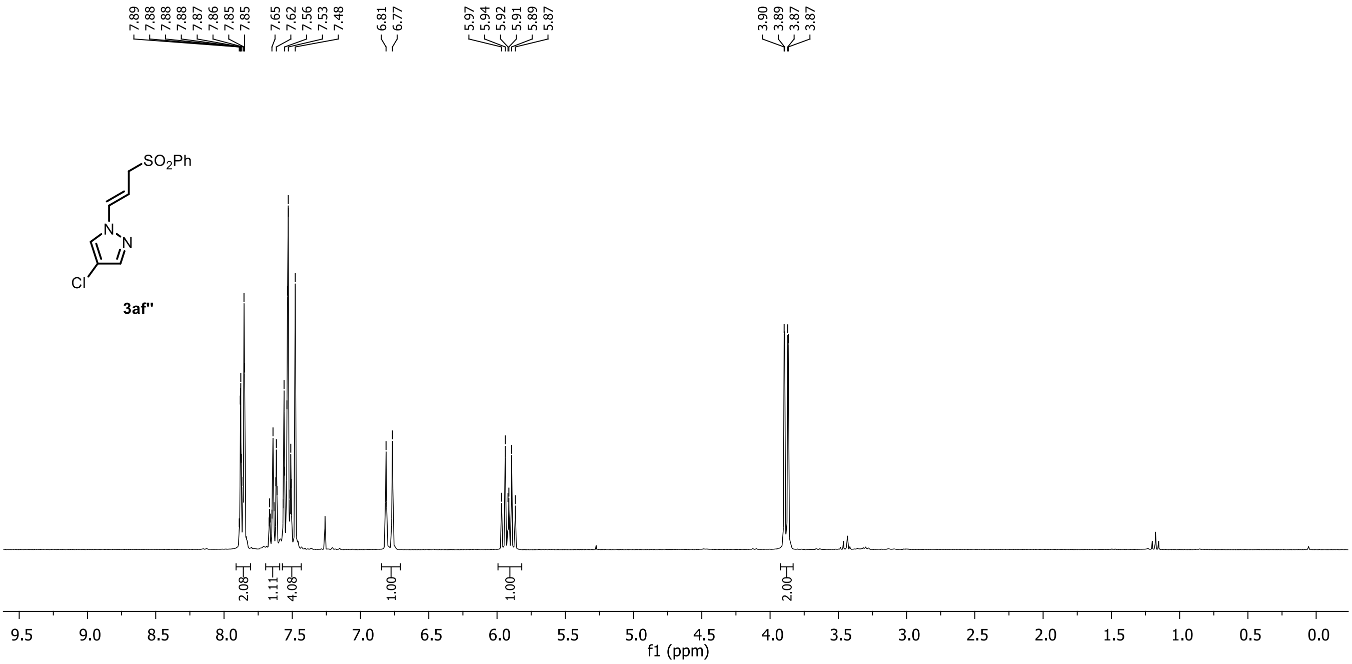

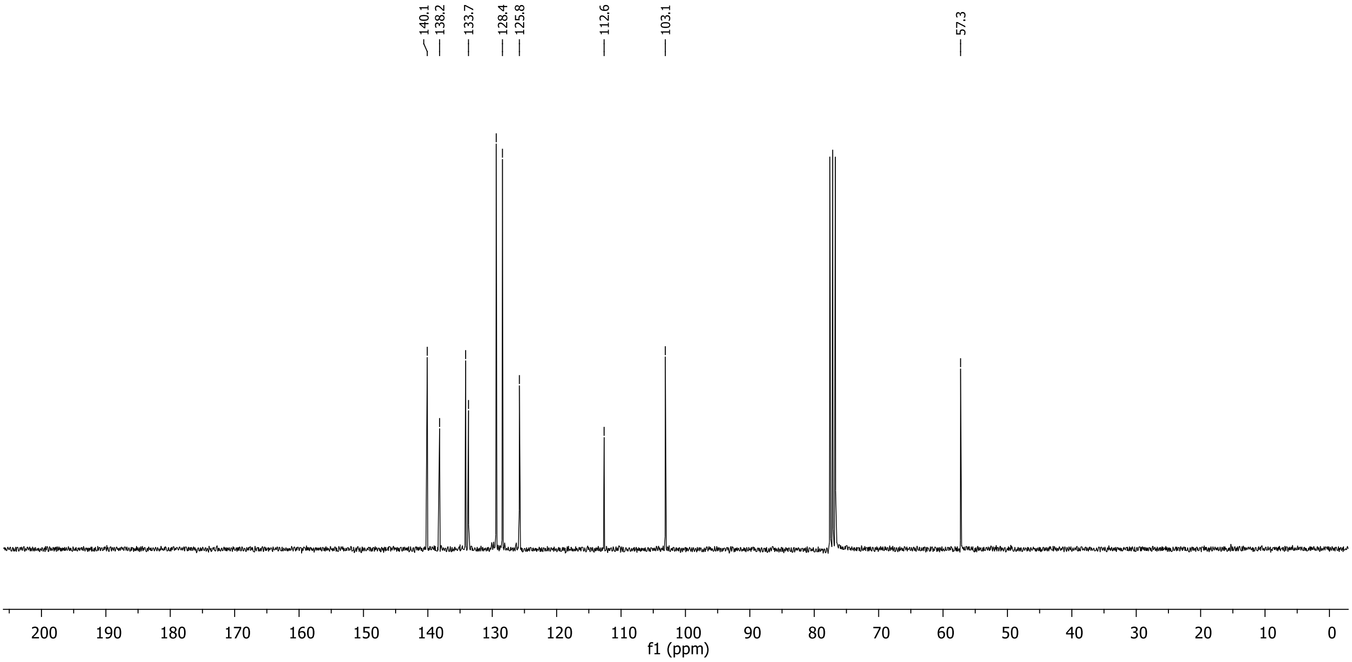

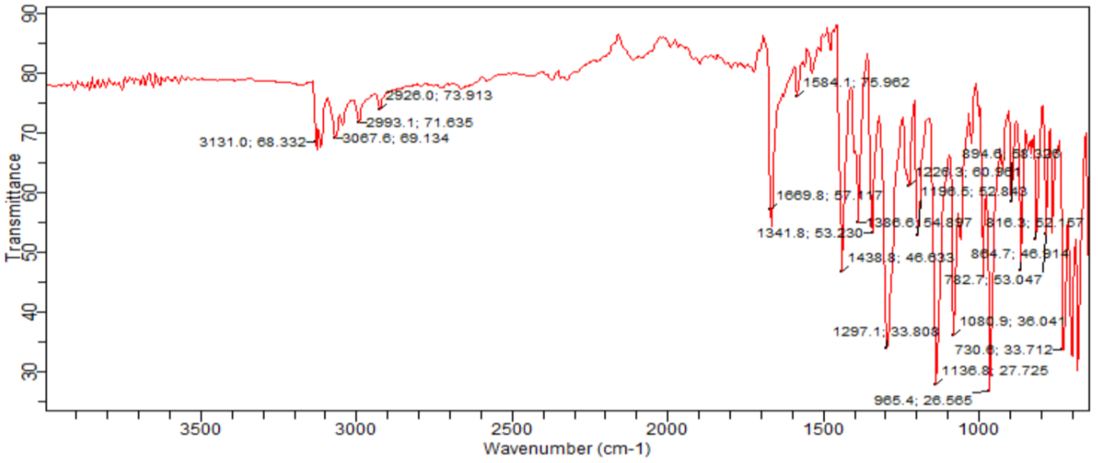

Ethyl 4-(4-chloro-1H-pyrazol-1-yl)-2-oxocyclopent-3-ene-1-carboxylate (4a) (^{1}H NMR: 400 MHz, ^{13}C NMR: 101 MHz, $CDCl_3$):

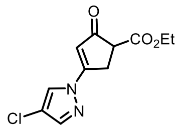

4a

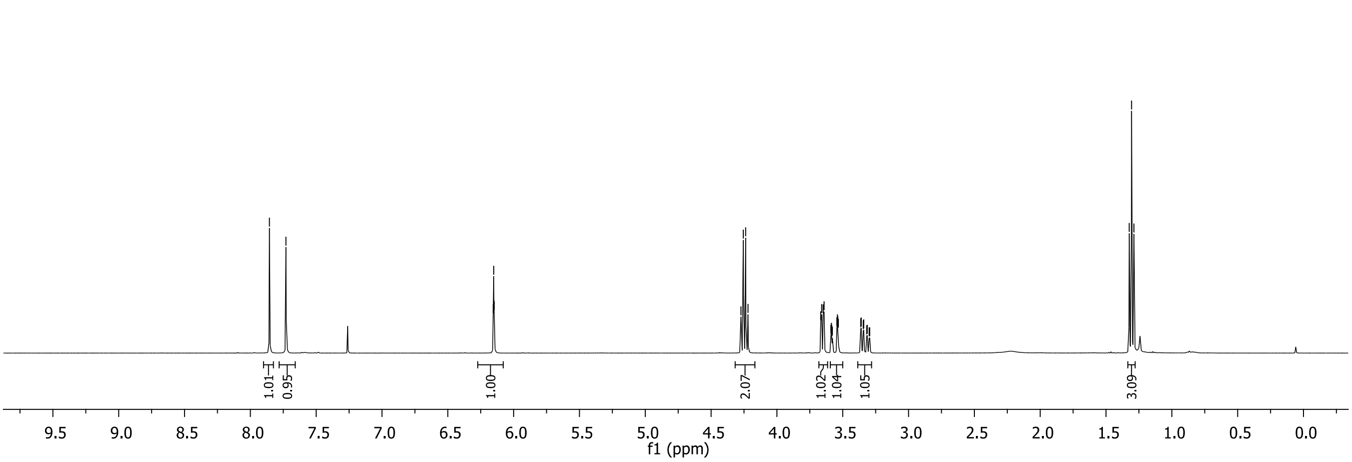

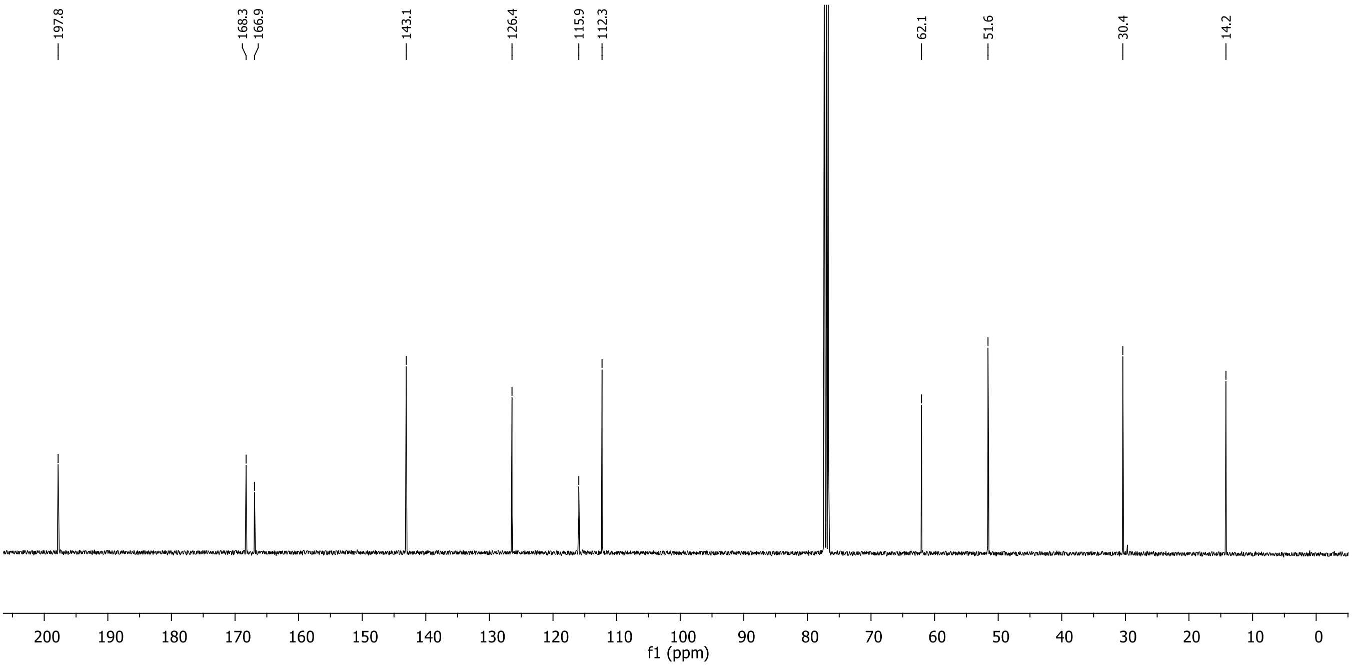

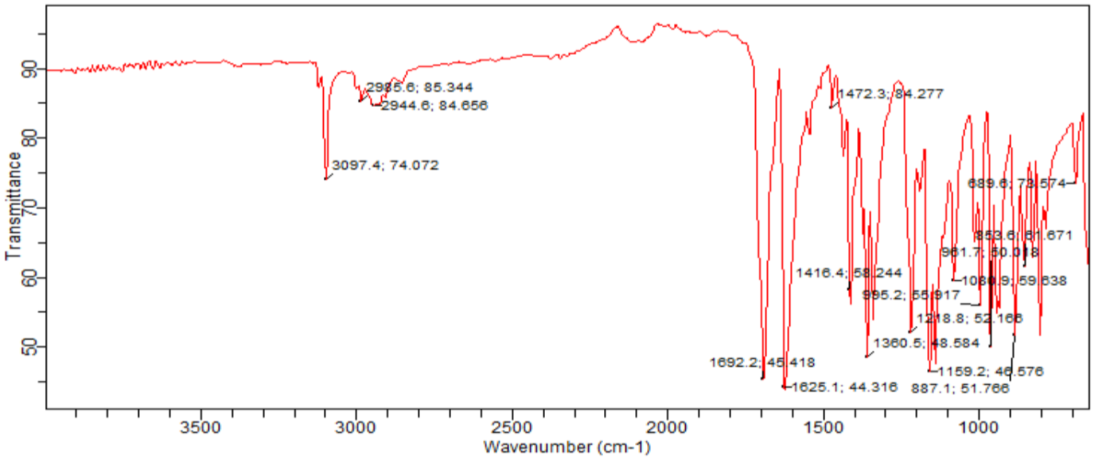

Ethyl 4-(1H-benzo[d][1,2,3]triazol-1-yl)-2-oxocyclopent-3-ene-1-carboxylate (4b) (^{1}H NMR: 400 MHz, ^{13}C NMR: 101 MHz, $CDCl_3$):

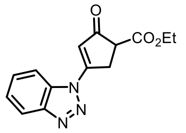

4b

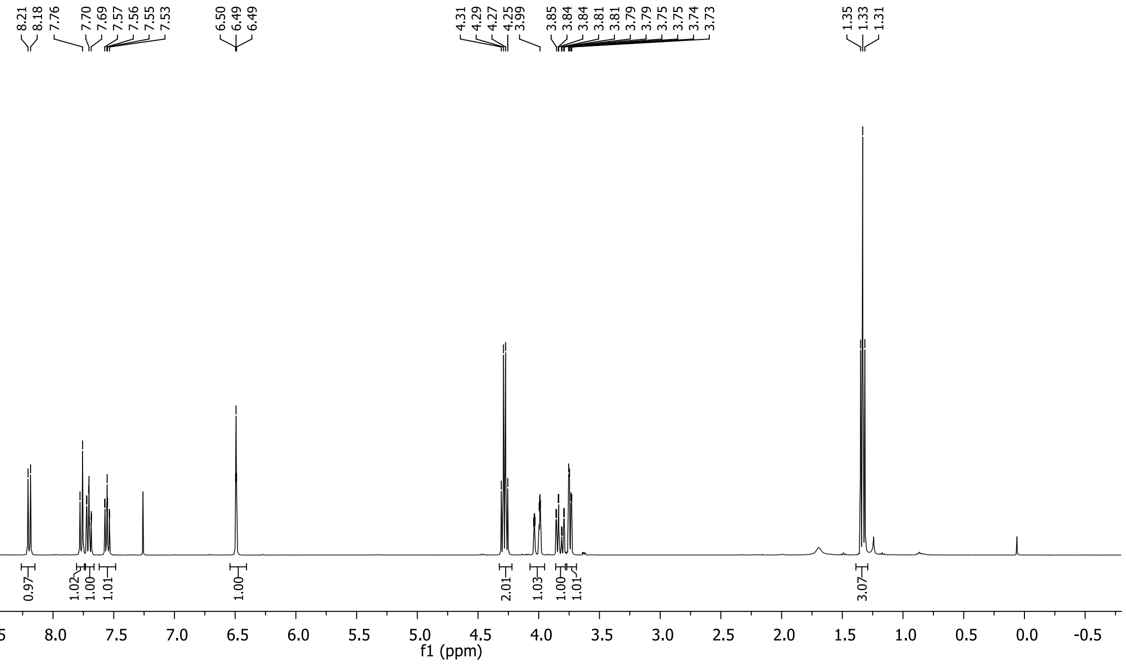

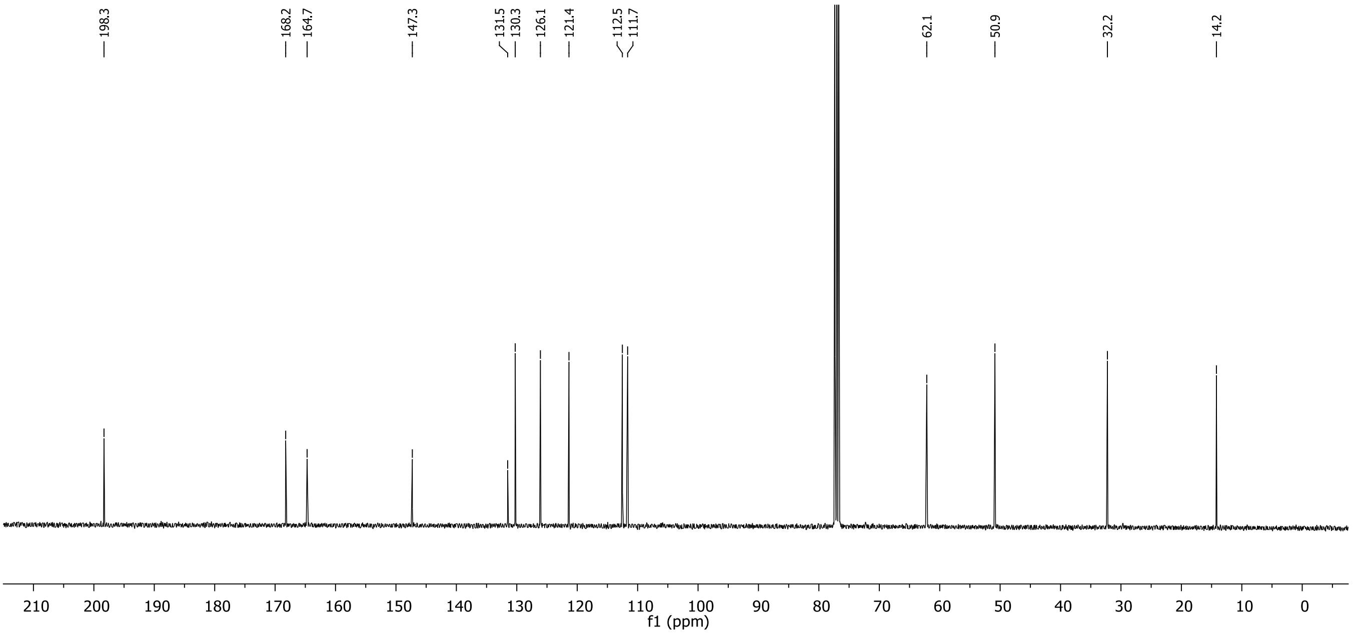

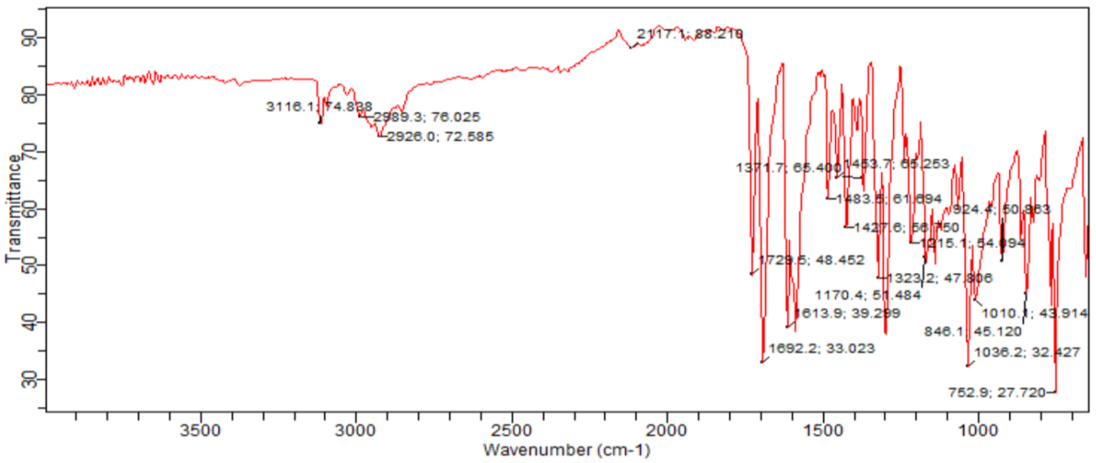

HPLC Data of Chiral Products

Diethyl (*E*)-4-(4-chloro-1H-pyrazol-1-yl)hex-2-enedioate (3c): (IC-3, hexane/^{i}PrOH 90:10, flow rate 0.5 mL/min, 25 °C)

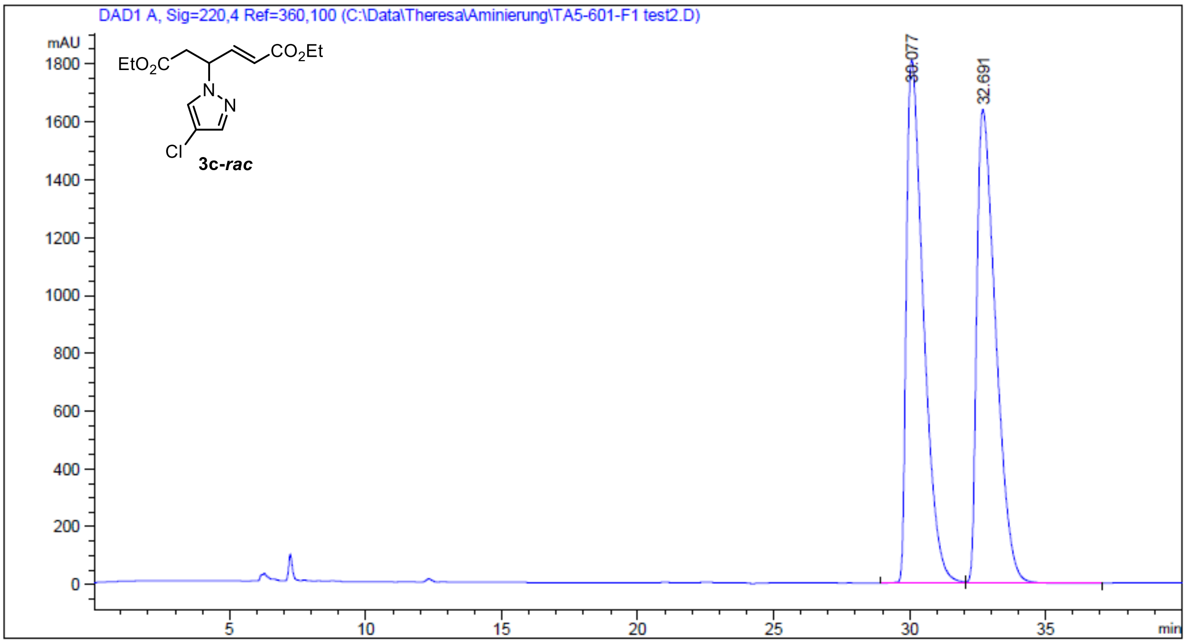

Signal 1: DAD1 A, Sig=220,4 Ref=360,100

Peak #	RetTime [min]	Type	Width [min]	Area [mAU*s]	Height [mAU]	Area %
1	30.077	BV	0.6560	7.87765e4	1809.01611	49.9147
2	32.691	VB	0.7357	7.90459e4	1636.04053	50.0853

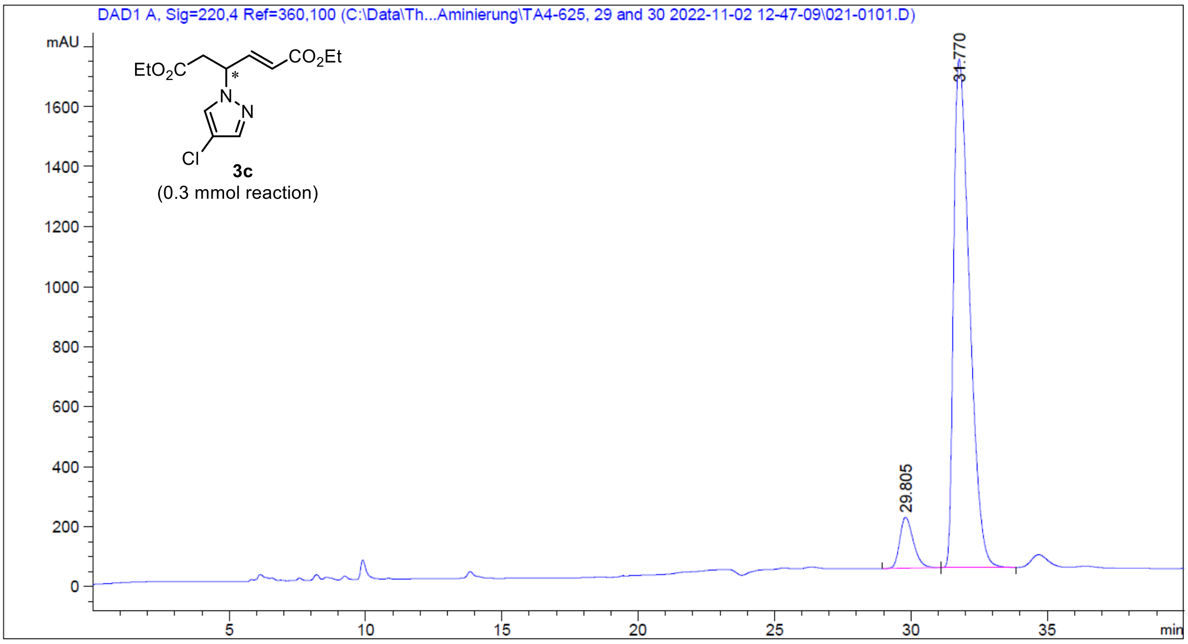

Signal 1: DAD1 A, Sig=220,4 Ref=360,100

Peak #	RetTime [min]	Type	Width [min]	Area [mAU*s]	Height [mAU]	Area %
1	29.805	BB	0.5367	5895.06396	169.93805	7.9279
2	31.770	BB	0.6273	6.84630e4	1694.85132	92.0721

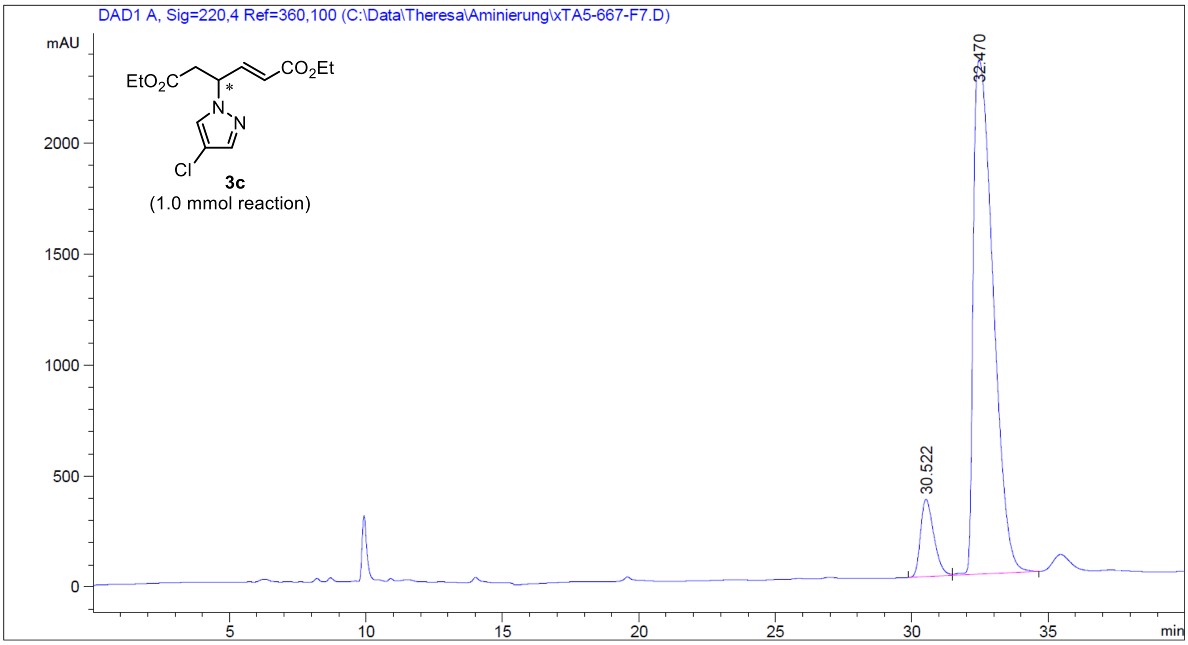

Signal 1: DAD1 A, Sig=220,4 Ref=360,100

Peak #	RetTime [min]	Type	Width [min]	Area [mAU*s]	Height [mAU]	Area %
1	30.522	BV	0.5246	1.18518e4	348.65198	9.2378
2	32.470	VB	0.7945	1.16445e5	2316.18677	90.7622

Diethyl (*E*)-4-(1H-benzo[d][1,2,3]triazol-1-yl)hex-2-enedioate (3bh): (IB-3, hexane/^{i}PrOH 90:10, flow rate 0.8 mL/min, 25 °C)

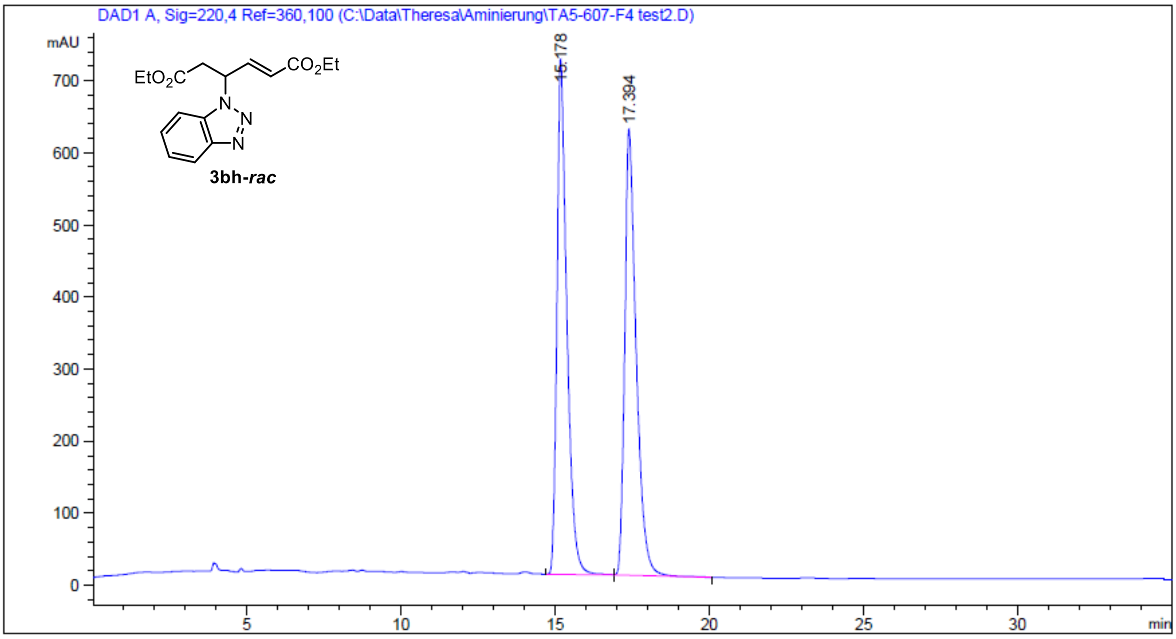

Signal 1: DAD1 A, Sig=220,4 Ref=360,100

Peak #	RetTime [min]	Type	Width [min]	Area [mAU*s]	Height [mAU]	Area %
1	15.178	BB	0.3212	1.58630e4	713.93103	49.9996
2	17.394	BB	0.3691	1.58632e4	618.74188	50.0004

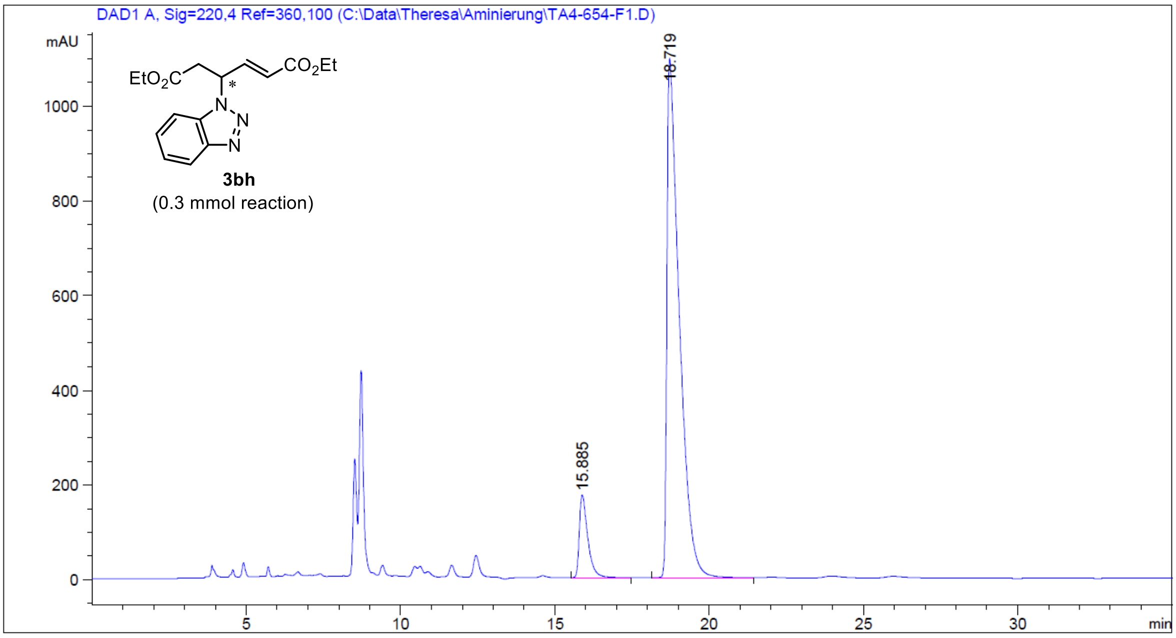

Signal 1: DAD1 A, Sig=220,4 Ref=360,100

Peak #	RetTime [min]	Type	Width [min]	Area [mAU*s]	Height [mAU]	Area %
1	15.885	BB	0.2843	3335.45313	175.05074	9.8562
2	18.719	BB	0.4028	3.05059e4	1095.61487	90.1438

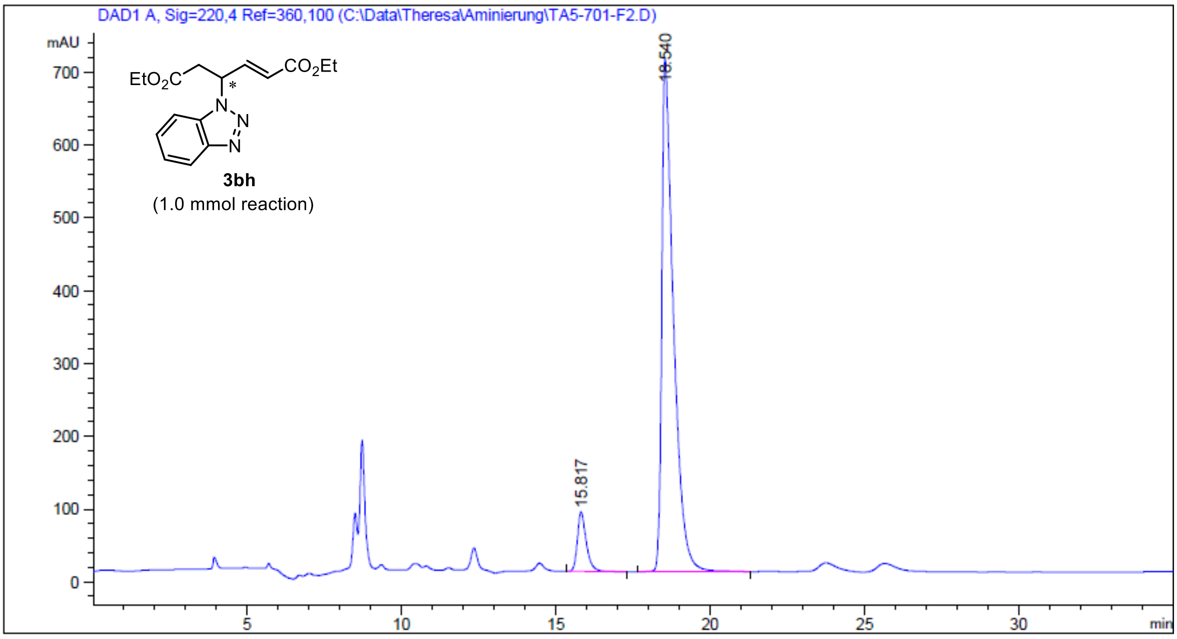

Signal 1: DAD1 A, Sig=220,4 Ref=360,100

Peak #	RetTime [min]	Type	Width [min]	Area [mAU*s]	Height [mAU]	Area %
1	15.817	BB	0.3004	1649.01099	82.07240	8.1749
2	18.540	BB	0.3790	1.85227e4	703.94849	91.8251

Author Contributions

A.B. conceived and designed the photo-aerobic regioselective intermolecular *N*-Allylation reaction. T.L. and T.A. performed the condition opitimzation, provided the products for the substrate scopes. T.A. wrote the manuscript. T.L. and T.A. wrote the Supporting Information.
